# Predator Communities With Invasive Pike Are Associated With Changes in Parental Care and Brain Gene Expression in Wild Alaskan Stickleback

**DOI:** 10.1093/icb/icag130

**Published:** 2026-07-28

**Authors:** Eric Arredondo, Brendon Byrd, Laura Stein

**Affiliations:** School of Biological Sciences, University of Oklahoma, 730 Van Vleet Oval, Norman, OK 73019, United States; School of Biological Sciences, University of Oklahoma, 730 Van Vleet Oval, Norman, OK 73019, United States; School of Biological Sciences, University of Oklahoma, 730 Van Vleet Oval, Norman, OK 73019, United States

## Abstract

Invasive predators can influence prey through direct predation but also via nonconsumptive effects that alter behavior and physiology. Parental care may be particularly sensitive to these effects, as parents must balance offspring investment with survival under predation risk. Here, we examine how predator exposure and predator community composition influence paternal behavior and brain gene expression in wild populations of threespined stickleback (*Gasterosteus aculeatus*) in southcentral Alaska. We conducted field behavioral assays in lakes with either native predators only or both native predators and invasive northern pike (*Esox lucius*), by recording changes in male fanning behavior following predator exposure. We then analyzed whole-brain gene expression using RNA sequencing on a subset of males. Stickleback males parented more in lakes with invasive predators compared to lakes with only native predators. However, after exposure to a model predator, neither predator treatment nor lake type significantly explained short-term changes in paternal behavior, suggesting that behavioral responses to acute predation risk are variable or constrained in natural settings. In contrast, transcriptomic analyses revealed 47 differentially expressed genes associated with predator community. Males from lakes with invasive pike showed enrichment of pathways related to protein synthesis and folding, whereas males from native predator-only lakes showed enrichment of immune response pathways. Parenting state showed minimal transcriptional differentiation. These results suggest that while invasive predator effects were less pronounced in short-term behavioral responses, they may be causing cumulative impacts over time, as evidenced by the baseline fanning behavior and gene expression patterns.

## Introduction

Invasive predators are a major driver of biodiversity loss, with an estimated 738 vertebrate species becoming extinct or endangered due to the impacts of just 30 introduced predators worldwide (Doherty et al. 2016). While the impacts of direct consumptive effects of predators on prey populations are clear, predators can also exert strong nonconsumptive effects that disrupt behavior, physiology, and reproduction, without direct predation (Preisser et al. 2005). These effects are frequently described within the framework of a “landscape of fear,” in which prey adjust behavior according to perceived predation risk across space and time (Gaynor et al. 2019). Since such responses can hinder growth, survival, and reproductive success, nonconsumptive effects represent a critical but often underappreciated means through which invasive predators impact native populations. Additionally, if non-consumptive effects depend on evolved recognition, then invasive predators may not be recognized as threatening, which in turn can impact native populations (Benkwitt 2017).

Parental care is likely to be especially sensitive to nonconsumptive effects because parents must simultaneously protect themselves while caring for their offspring. In many systems, anti-predator responses during reproduction involve tradeoffs between current reproductive investment and parental survival, and these tradeoffs can vary depending on environmental context, offspring state, and predator type (Fuiman and Magurran 1994; Ghalambor et al. 2013; Sheriff et al. 2017). For nest-tending species, predator exposure may influence how quickly parents resume care following predator exposure, how much effort they allocate to caring for offspring, and how they balance competing risks to themselves and offspring.

Tradeoffs between parental investment and survival may be amplified in the presence of invasive predators. Native prey are expected to have evolutionary history with established predators, resulting in adaptive antipredator responses. Invasive predators, however, may introduce unfamiliar cues or alter predation environments in ways that disrupt evolved decision-making processes (Sih et al. 2010). As a result, responses to invasive predators may be weak, generalized, or mismatched relative to responses to native predators. There is an increasing amount of work describing nonconsumptive effects of predation on parental effects, showing predators can impact populations across time (MacLeod et al. 2022); yet we know less about short-term effects of invasive predators on parenting. This represents an important gap, as changes in parental behavior can have cascading consequences for offspring and thus species survival (Ghalambor and Martin 2001; Fontaine and Martin 2006).

Understanding the consequences of nonconsumptive effects by invasive predators can be strengthened by integrating behavioral assays with gene expression analyses to link predator exposure to underlying molecular processes. By combining behavioral and transcriptomic approaches, we can better understand how organisms respond to invasive predator cues, as different predator environments may be associated with variation in the molecular mechanisms underlying those responses in the brain. Further, combining field behavioral experiments with transcriptomic analyses can reveal mechanisms linking ecological context to phenotypic responses.

Here, we investigate how predator exposure and predator community composition influence parental behavior and brain gene expression in parenting threespined stickleback fish (*Gasterosteus aculeatus*). In southcentral Alaska, invasive northern pike (*Esox lucius*) introduced in the mid-20th century have altered predator communities in many lakes that historically contained only native predators, such as trout (*Oncorhynchus spp*.) (Alaska Department of Fish and Game, Hendry et al. 2024). Stickleback provide obligate paternal care in the form of fanning that is known to dynamically respond to predation risk in both the lab and field, typically by reducing fanning during and after predation risk (Stein and Bell 2012, 2014, 2015, 2019; Hellmann et al. 2021). As we are interested in whether invasive predators impact the parenting brain, we compared a subset of parenting males to a sample of non-parenting males (males that had nests but no embryos in them). We predicted that predator exposure and lake predator community structure would influence both fanning behavior and brain gene expression, where stickleback would be more variable in their reduction of parental care after being exposed to an invasive predator model compared to models of a native predator, trout (*Oncorhynchus spp*.), and a non-predatory fish, Arctic char (*Salvelinus alpinus*).

## Materials and methods

### Study sites

**Fig. 1:**
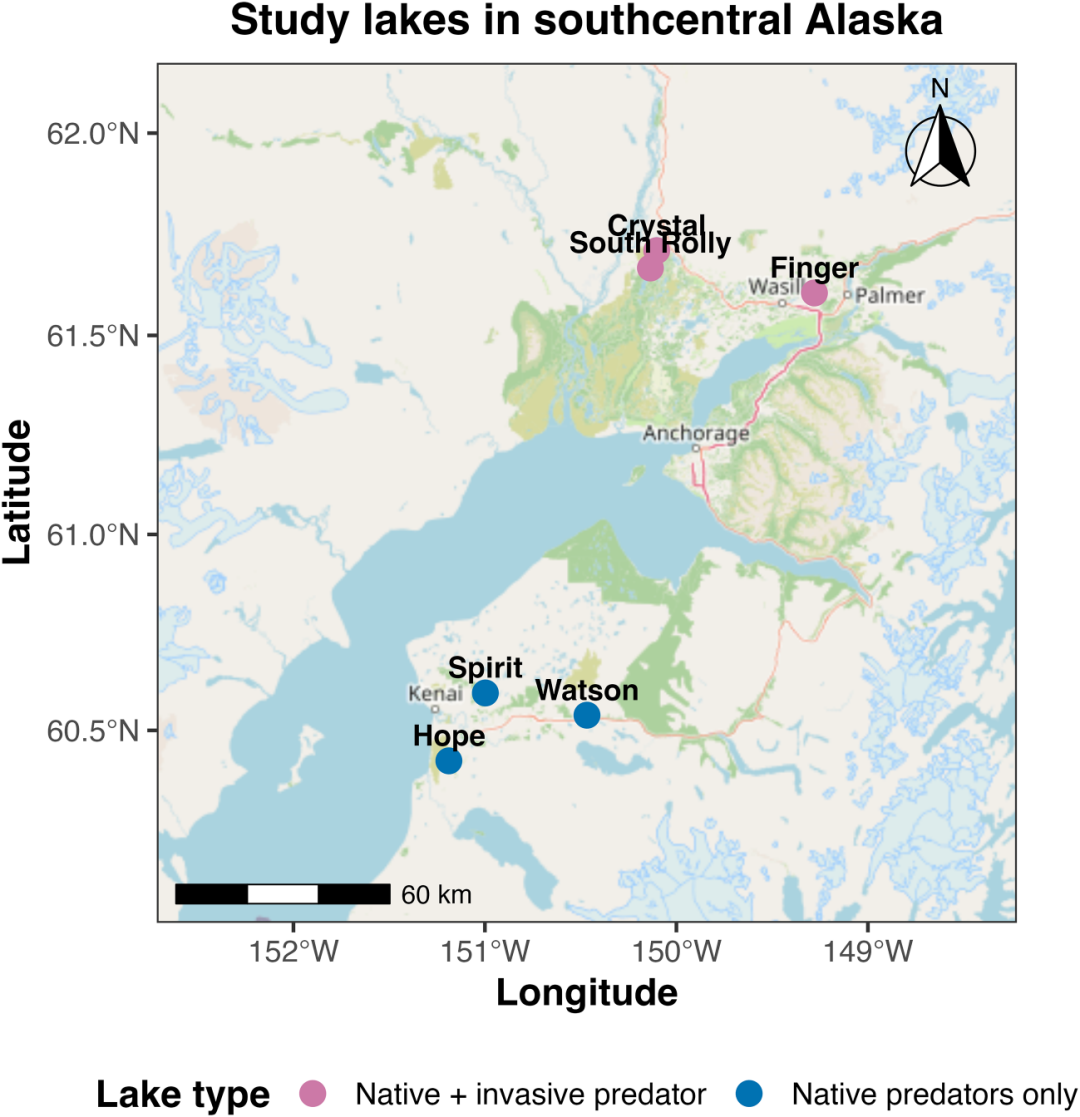
Map of the six study lakes in southcentral Alaska sampled for stickleback. The three northernmost points indicate lakes containing both native predators and invasive northern pike, whereas the three southernmost points indicate lakes containing only native predators. Labels identify each study lake, and the scale bar represents 60 km.

We sampled threespined stickleback in 6 lakes in southcentral Alaska under aquatic research permits from the Alaska Department of Fish and Game (P-24–014) and University of Oklahoma IACUC 2022–0190. Three lakes have current records of the presence of invasive pike populations in the Mat-Su Valley (Finger, South Rolly, Crystal; Table 1) (Alaska Department of Fish and Game), while the other three do not have populations of invasive pike in the Kenai region (Hope, Spirit, Watson; Fig. 1, Table 1).

**Table 1:** Study lakes in southcentral Alaska, including lake type classification, geographic coordinates, and sample sizes for behavioral assays and RNA sequencing.

Lake	Lake type	Coordinates	n per lake (behavior, RNA sequencing)
South Rolly	Native + invasive predator	61.667545, -150.135689	4, 5
Crystal	Native + invasive predator	61.710514, -150.102865	9, 3
Finger	Native + invasive predator	61.605600, -149.279200	9, 2
Watson	Native predators only	60.539000, -150.467000	5, 3
Hope	Native predators only	60.421528, -151.188278	6, 2
Spirit	Native predators only	60.596104, -150.997664	5, 4

Lakes are categorized as either containing only native predators or both native and invasive predators (northern pike). Sample sizes are reported as the number of individuals included in behavioral analyses and transcriptomic analyses for each lake.

### Experimental overview

#### Behavioral assays

Sampling was conducted between May 24 and June 18, 2024, between 1100 and 1505 h. Nesting three-spined stickleback males were located via snorkeling. Following a 2-min acclimation period to the snorkeler and video camera (AKASO EK7000) placed 10 cm from a male’s nest, 5-min baseline videos were taken. Another 2-min acclimation period to the snorkeler occurred before the male was exposed to a randomly assigned treatment of a model (char: non-predatory fish, Collectible Wildlife Gifts, 14.29 × 3.49 cm model; trout: native predator, Savage Gear 20.32 × 4.45 cm model; pike: invasive predator, Savage Gear, 26.35 × 3.81 cm model). Using a clear rod glued to the top of the models, the models were moved 10cm right of the nest mimicking fish swimming behavior for approximately 10 s. We recorded behavior for an additional 5 min following treatment exposure. The snorkeler was not present at the nest aside from setting up, acclimating, and moving the model. One hour after the model predator was removed, males were collected via dip net. Once the snorkeler was at the nest to retrieve the male, it took typically less than a minute to retrieve the male and then it took about 1–2 min to get back to shore. Males were sacrificed within 1–2 min once they reached shore. We understand there may be gene-expression changes if there were prolonged periods of stress or change for the fish, but given that each fish was sacrificed quickly and there were no major differences in timing between males, we feel confident our methods did not influence results. Standard length and mass were measured before males were euthanized via decapitation. Nest microhabitat data (depth and cover) were measured and nests collected to determine embryo developmental stage (early, late, fry, or mixed). All observations and video scoring was conducted using BORIS by a sole observer (EA) (Friard and Gamba 2016). Sample sizes for behavioral assays ranged from 4 to 9 per lake (see Table 1 for details).

#### RNA extraction and sequencing

Whole brains of a subset of sampled males (*n* = 19, see Table 1 for details) were stored in RNALater, then kept in a cooler with ice packs while at the lakes before being kept frozen in the field in a kitchen freezer (~ -18°C) before being shipped to the University of Oklahoma, where they were stored at −80°C. RNA was extracted using Zymo Research Direct-zol RNA Miniprep kits and checked using FastQC. All extractions were done by one extractor (EA). Paired-end sequencing was conducted by the Clinical Genomics Center at the Oklahoma Medical Research Foundation (Oklahoma City, OK). Prior to mRNA-seq analysis, quality control measures were implemented. Concentration of RNA was ascertained via fluorometric analysis on a Thermo Fisher Qubit 4 fluorometer. Overall quality of RNA was verified using an Agilent Tapestation 4200 instrument. Following initial QC steps, mRNA was isolated and libraries were generated using the Watchmaker Genomics mRNA kit according to the manufacturers protocol. Briefly, mature mRNA was enriched total RNA via pull-down with beads coated with oligo-dT homopolymers. The mRNA molecules were then chemically fragmented and the first strand of cDNA was generated using random primers followed by second strand synthesis and adapter ligation. Libraries were then indexed using IDT xGen Unique Dual Indexing primers. Final libraries for each sample were assayed on the Agilent Tapestation 4200 for appropriate size and quantity. These libraries were then pooled in equimolar amounts as ascertained via fluorometric analyses. Final pools were absolutely quantified using qPCR on an ABI QuantStudio 5 instrument with NEB Illumina Library Quantification reagents. Sequencing of the libraries was performed on an Illumina NovaSeq X Plus with PE150 reads. Samples had a median sequencing depth of 13.4 million paired-end reads (range: 7.8–21.4 million reads) and a median RNA integrity number (RIN) of 9.1 (range: 5.8–9.8).

### Statistical analyses

#### Behavioral analyses

Behavioral analyses were conducted in R (v4.5.2; R Core Team 2025). Behavioral analyses were restricted to males with embryos in their nest. The main behavioral response of interest was quantified as the change in male fanning time, calculated as the difference between fanning duration during the predator treatment period and baseline observations.

To evaluate ecological and environmental predictors of changes in fanning, a set of linear mixed-effects models (LMMs) were fit using the lme4 package (Bates et al. 2003). Candidate fixed effects included predator treatment (Char, Pike, Trout), lake type (native predator only vs. native + invasive predator lakes), body condition, nest depth, % cover, latency to return to the nest, and embryo developmental stage (Supplementary Table S1). Lake identity was included as a random intercept to account for non-independence of observations within lakes. As our main question of interest for behavior involves investigating how lake type and predator treatments influences changes in parenting, all the models we formed, therefore, include them as they are the main effects we hypothesized a priori would have the largest effect on parenting. To avoid step-wise selection and including models we did not have a priori hypotheses for, a null model was not included in candidate models, as null models may assume the effect of each of the variables we measured to be zero, which we did not view as biologically realistic in our system, and thus compared more maximal versions of models (Whittingham et al. 2006; Bolker 2024).

We initially sampled *n* = 54 males but removed 16 as they did not have embryos in their nest. The dataset for model selection had *n* = 38 males (native predator-only lakes: Char = 5, Pike = 7, Trout = 4; native + invasive predator lakes: Char = 7, Pike = 6, Trout = 9). These sample sizes provide us with 39% power to detect treatment effects, and 27% power to detect lake type (pike/no pike) effects at the alpha = 0.05 level. Power calculations were conducted using the “simr” package (Green and MacLeod 2016).

Model selection was performed using AICc-based model comparison using the MuMIn package (Bartoń 2010). All candidate models were fit using maximum likelihood (REML = FALSE) to ensure valid comparison of model likelihoods. The model with the lowest AICc value was selected as the final model and was subsequently refit using restricted maximum likelihood (REML = TRUE) to obtain final parameter estimates and inference.

Model diagnostics were evaluated using residual histograms, quantile–quantile plots, and residual-versus-fitted plots. Residual normality was additionally assessed using the Shapiro–Wilk test, and multicollinearity among predictors was evaluated using variance inflation diagnostics from the performance package (Lüdecke 2021). Standardized effect sizes for fixed effects were calculated using the effectsize package (Ben-Shachar et al. 2020). Estimated marginal means and pairwise comparisons among predator treatments were calculated using the emmeans package (Lenth et al. 2026), with Tukey-adjusted post hoc tests to control for multiple comparisons.

#### Transcriptomic analyses

##### Differential expression

Raw RNA-seq reads were quality filtered and adapter trimmed using fastp. Filtered reads were aligned to the *Gasterosteus aculeatus* reference genome (GAculeatus_UGA_version5) (Nath et al. 2021) using STAR. Gene annotations from Ensembl (release 115) were used for transcript annotation. Gene-level counts were generated using featureCounts. RNA sequencing analyses were conducted in R (v4.5.2; R Core Team 2025) using the DESeq2 package (Love et al. 2014). Since we were interested in comparing gene expression between parental state, our sequencing subset (*n* = 19) is a subset from the original total behavioral dataset (*n* = 54). Raw gene count matrices were imported and matched to sample metadata containing information on lake type and batch (*n* = 19; native predator-only lakes = 9; native + invasive predator lakes = 10; Parenting = 13, Non-parenting = 6). Genes with low counts were filtered prior to analysis, retaining genes with at least 10 counts in at least 3 samples. Differential expression was analyzed using negative binomial generalized linear models. The model included extraction batch as a covariate and lake type and parenting state (parenting vs. non-parenting) as the biological predictors in an additive model (Leek et al. 2010). To assess pathway-level shifts, genes were ranked by DESeq2 Wald statistics and analyzed using Gene Set Enrichment Analysis (GSEA) using the clusterprofiler package and Gene Ontology (Subramanian et al. 2005). were considered differentially expressed if they satisfied both: False discovery rate (FDR) < 0.05 (Benjamini–Hochberg correction) and an Absolute log2 fold change ≥ 0.58, equivalent to about 1.5-fold change. Variance-stabilized transformation (vst) was applied to normalized counts for downstream visualization and exploratory analyses. Principal component analysis (PCA) was used to examine overall transcriptomic structure, assess potential batch and lake effects.

## Results

### Differences in baseline fanning

Baseline fanning behavior differed between lake types prior to predator-model exposure. Males from lakes containing invasive northern pike exhibited higher baseline fanning rates than males from lakes containing only native predators (β = 29.67, SE = 13.66, t = 2.17, *P* = 0.037). In contrast, ecotype (benthic, *n* = 14, or limnetic, *n* = 24) did not significantly predict baseline fanning behavior, as limnetic males did not differ from benthic males in baseline fanning rates (β = −18.22, SE = 13.73, t = −1.33, *P* = 0.194). The random effect of lake explained negligible variance indicating that little additional variation was attributable to lake identity after accounting for lake type and ecotype.

### Differences in short-term fanning

When analyzing factors influencing the difference in fanning post-predator treatment, AICc model selection found the model including predator treatment and lake type as additive fixed effects, with lake as a random intercept, as the top-supported behavioral model (AICc = 381.94, model weight = 0.55). Models including additional predictors such as nest depth or embryo stage were less supported (ΔAICc = 2.37 and 2.52, respectively), and a model including a treatment × lake type interaction was also less supported (ΔAICc = 3.92). The best supported behavioral model was Difference in acute fanning ~ Treatment + Lake_type + (1|Lake). The estimated variance associated with the lake random effect added negligible additional variance attributable to lake identity beyond the fixed effects.

**Fig. 2:**
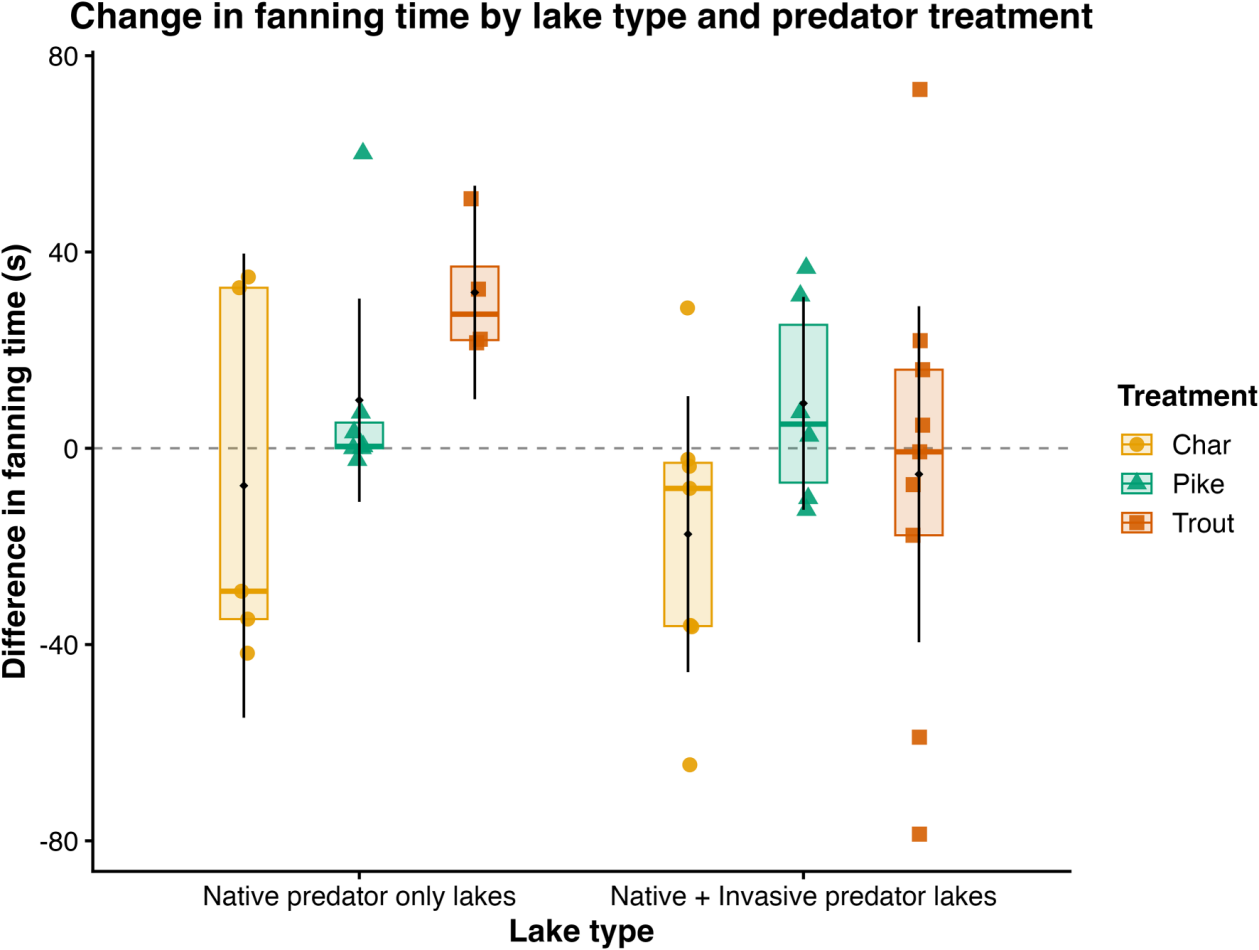
Change in male fanning behavior across lake types and predator treatments. Boxplots show the change in fanning time (s) in response to a model predator across lakes containing only native predators and lakes containing both native predators and invasive northern pike. Points represent individual males; shapes and colors indicate predator treatment (char, pike, trout). Boxes represent the interquartile range with the median indicated by the horizontal line. The diamond represents mean and the vertical black error bars show the 95% confidence interval of the mean.

In the best-supported model, neither predator treatment nor lake type significantly predicted change in short-term fanning behavior. Relative to char, the effect of pike was positive but not statistically significant (β = 21.06, SE = 12.91, t = 1.63, *P* = 0.112; standardized β = 0.63, 95% CI [−0.16, 1.43]), and the effect of trout was similarly positive and non-significant (β = 21.12, SE = 12.90, t = 1.64, *P* = 0.111; standardized β = 0.64, 95% CI [−0.16, 1.43], Table 2, Fig. 2).

**Table 2:** Parameter estimates from the top-supported linear mixed-effects model examining the effects of predator treatment and lake type on changes in male fanning behavior.

Term	Estimate	SE	df	t_value	*P*_value	Std_beta	CI_low_95	CI_high_95
Intercept	-4.652	11.186	34	-0.416	0.680	-0.174	-0.86	0.513
Treatment: pike vs. char	21.057	12.914	34	1.631	0.112			
Treatment: trout vs. char	21.120	12.901	34	1.637	0.111			
Lake type: native + invasive vs. native only	-14.967	10.747	34	-1.393	0.173			

Estimates are shown with standard errors (SE), degrees of freedom (df), t-values, *p-*values, standardized effect sizes (β), and 95% confidence intervals. Predator treatments are presented relative to the non-predatory char reference group. Lake type compares native + invasive predator lakes to native predator-only lakes.

Lake type had a negative but non-significant effect, indicating lower overall fanning responses in invasive-pike lakes compared to native predator-only lakes (β = −14.97, SE = 10.75, t = −1.39, *P* = 0.173; standardized β = −0.45, 95% CI [−1.11, 0.21]). Confidence intervals for all predictors overlapped zero.

Mean fanning responses varied across treatments but followed consistent patterns across lake types. In native predator-only lakes, mean fanning change was negative under char but positive under predator treatments (char = −4.65, pike = 16.41, trout = 16.47), whereas in invasive-pike lakes, mean responses were lower across all treatments (char = −19.62, pike = 1.44, trout = 1.50).

Tukey-adjusted pairwise comparisons among treatments were not statistically significant (char vs. pike: estimate = −21.06, SE = 13.0, *P* = 0.252; char vs. trout: estimate = −21.12, SE = 13.1, *P* = 0.256; pike vs. trout: estimate = −0.06, SE = 13.2, *P* = 1.000).

### Differential expression

**Fig. 3:**
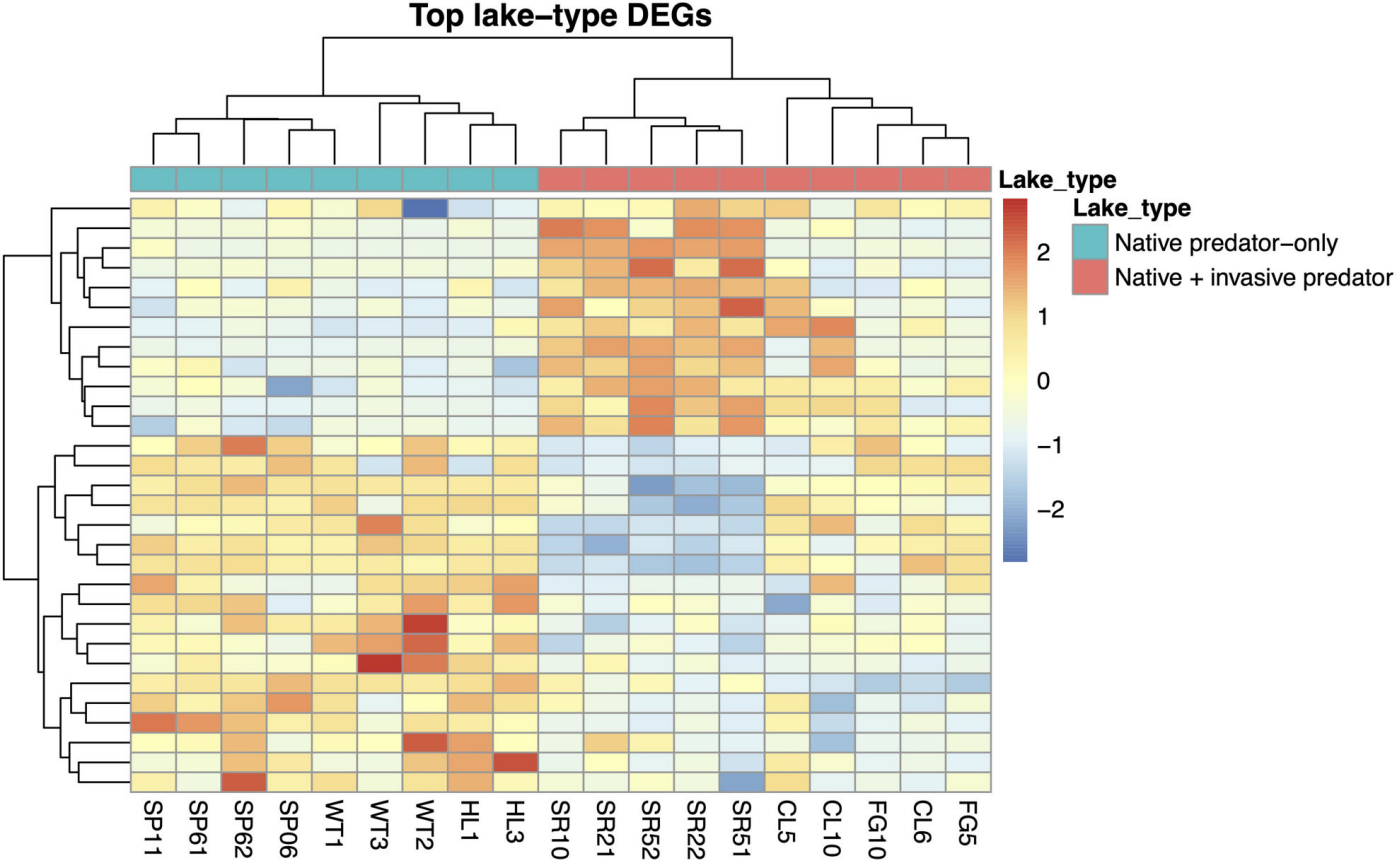
Expression patterns of top differentially expressed genes associated with lake type. Heatmap showing expression values of genes significantly differentially expressed between lake types. Rows represent genes and columns represent individual samples. Values represent relative expression levels (positive = higher expression, negative = lower expression).

**Fig. 4:**
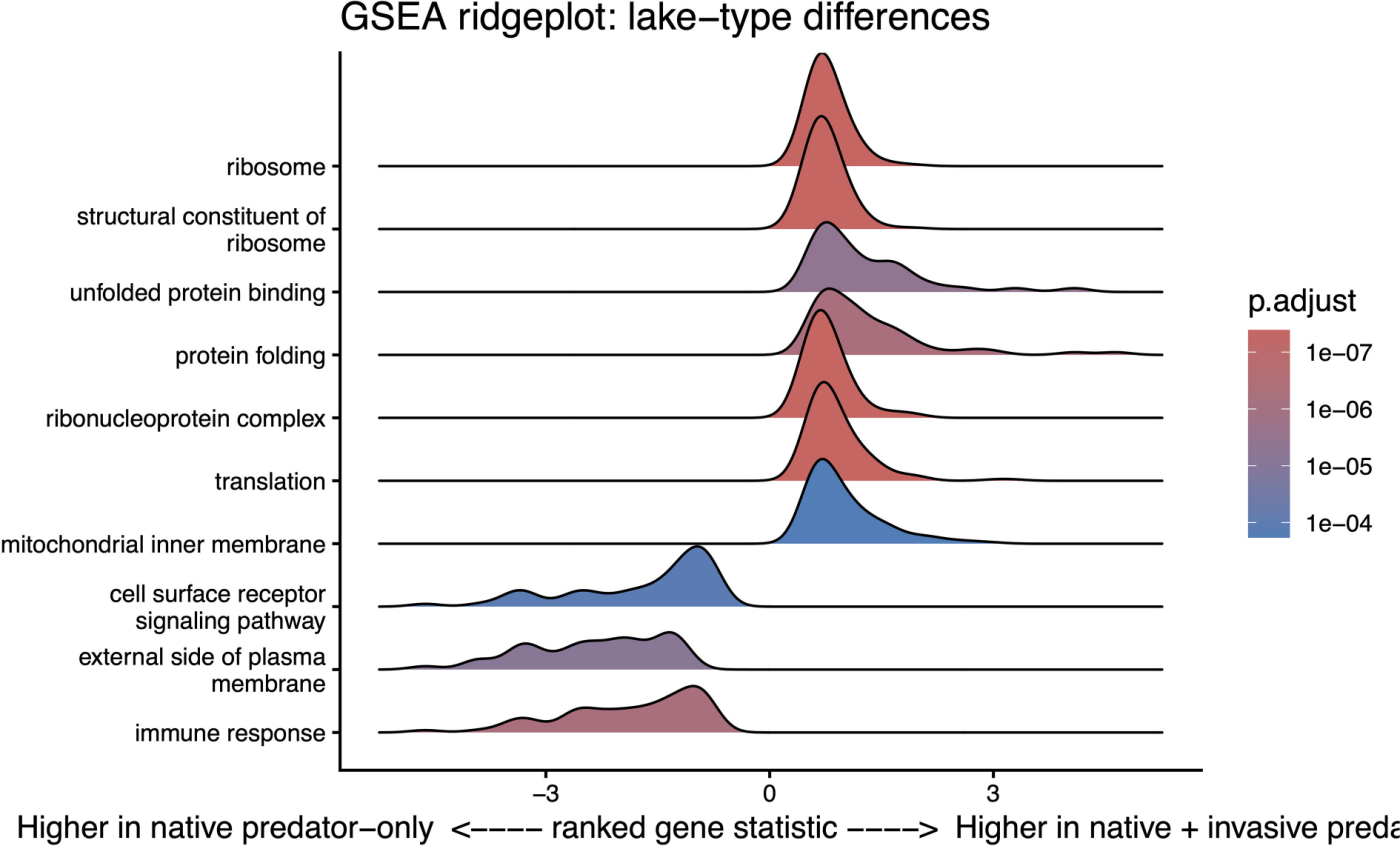
Gene set enrichment analysis of lake-type transcriptomic differences. Ridgeline plots showing significantly enriched gene ontology (GO) pathways associated with transcriptomic differences between lake types. The x-axis shows the ranked gene statistic from differential expression analysis. Negative values indicate higher expression in native predator-only lakes, whereas positive values indicate higher expression in lakes containing invasive northern pike. Color intensity indicates adjusted *p*-values.

For the transcriptomic analysis, out of 22,125 genes with nonzero total read counts, lake-type contrast identified 47 differentially expressed genes with clustering between lake-type showin in the top 30 differentially expressed genes (Fig. 3.). Principal component analysis of variance-stabilized gene expression values indicated that the first two principal components explained 26% and 18% of total transcriptomic variance, respectively. Batch effects detected 9 differentially expressed genes, while parenting state showed 1 differentially expressed gene. At the pathway level, GSEA revealed functional differences in brain gene expression between lake types (Fig. 4). Multiple pathways were significantly enriched (FDR < 0.05), with separation between native predator-only lakes and lakes containing both native and invasive predators. Pathways enriched in native + invasive predator lakes (positive NES values) were primarily associated with protein synthesis and cellular processing, including ribosome (NES = 2.52, FDR = 4.12 × 10⁻⁸), structural constituent of ribosome (NES = 2.41, FDR = 4.12 × 10⁻⁸), unfolded protein binding (NES = 2.30, FDR = 4.82 × 10⁻⁶), protein folding (NES = 2.29, FDR = 2.58 × 10⁻⁶), ribonucleoprotein complex (NES = 2.18, FDR = 4.12 × 10⁻⁸), and translation (NES = 2.17, FDR = 4.12 × 10⁻⁸). In contrast, pathways enriched in native predator-only lakes (negative NES values) were associated with immune and signaling processes, including immune response (NES = −2.10, FDR = 2.22 × 10⁻⁶), external side of plasma membrane (NES = −2.01, FDR = 5.75 × 10⁻⁶), cell surface receptor signaling pathway (NES = −1.98, FDR = 7.26 × 10⁻⁵), and transmembrane signaling receptor activity (NES = −1.82, FDR = 2.34 × 10^-4^).

## Discussion

With biological invasions becoming more frequent, understanding the impacts invasive species have on native populations have been well documented (Dick et al. 2013; Gallardo et al. 2016; Laverty et al. 2017), yet less is known about the nonconsumptive effects invasive predators have on native prey populations through changing parenting behavior. Investigating invasive predator effects requires moving beyond direct predation to ask how altered predator environments shape the behavior and physiology of native prey. By combining a field behavioral assay with whole-brain transcriptomics, our study approached this question through a parental-care framework, which is especially relevant because parenting individuals must continually balance offspring investment while protecting themselves. Across wild stickleback populations, we found that males parented more at baseline in lakes with invasive predators and brain gene expression corroborated these results. We also observed a trend whereby short-term paternal behavior exhibited some differentiation across predator treatments and communities with low power. Taken together, these results suggest that indirect effects of invasive predators may be better detected in longer-term phenotypes than in short-term behavioral responses measured under natural conditions.

### Weak predator community and treatment level effects on parenting

Males from lakes containing invasive northern pike exhibited significantly higher baseline fanning rates before predator exposure than males from lakes containing only native predators. This finding may suggest that predator community composition is associated with differences in parental care that are present prior to acute predator exposure. While the absence of consistent differences in short-term changes in fanning following predator presentation, the baseline difference suggests that predator communities may influence the overall level of parental investment rather than immediate behavioral changes. The processes responsible for this pattern cannot be determined from the present study, but long-term ecological differences among predator communities may contribute to persistent variation in paternal care behavior.

Post predator exposure, neither predator treatment nor lake type strongly explained change in fanning behavior. Although we did not find a significant effect on fanning, it may not be entirely surprising considering lower statistical power, and this was done in natural systems, where these fish are constantly exposed to various natural stimuli. For example, it is likely behavioral responses are shaped by multiple overlapping sources of natural variation, including nest contents, microhabitat, recent social interactions, and ambient ecological conditions. That being said, this field study provided a unique opportunity to investigate the parental behavior of native prey populations while preserving the ecological context in which behavior normally occurs.

The lack of strong behavioral separation among predator treatments also fits broader ideas from predator-naïveté theory. Work on invasive predators has shown that prey responses to novel threats are often weak, generalized, or mismatched rather than absent or inherently adaptive (Sih et al. 2010; Carthey and Banks 2014) and emphasize that invasive predator impacts can emerge when prey do not appropriately recognize or differentiate threats. Our findings support previous work in Alaskan stickleback, where no evolved differences in anti-predator behavior in response to invasive pike were found (Stevens et al. 2022). Our results are consistent with that general framework in that males did not appear to behaviorally distinguish strongly between native trout and invasive pike during8 the short post-exposure period. However, another plausible explanation is that actively caring fathers in the wild may rely on generalized risk-sensitive responses while maintaining offspring care, especially when abandoning or largely reducing care could itself be costly. In this sense, our behavioral results suggest that any immediate parental adjustment following a predator encounter may be subtle, generalized, or buffered by the demands of ongoing care.

### Lake type effect on gene expression

In contrast to the behavior results, the transcriptomic analysis revealed differentiation associated with lake type, and some separation between parenting states. Males from lakes containing invasive pike differed in brain gene expression from males in native predator-only lake. Ecological transcriptomics often detects modest single-gene differences alongside stronger pathway-level organization, especially in wild population, where transcriptomic data from natural systems are particularly useful for identifying responses to environmental changes (Alvarez et al. 2015). Our results support this, as differences in lake type expression suggests that broader predator environment is associated with differences at the molecular level in parenting individuals.

Males from invasive-pike lakes showed enrichment of ribosome, translation, ribonucleoprotein, and protein-folding-related pathways, whereas males from native predator-only lakes showed enrichment of immune-response pathways. These patterns suggest that fish from different predator regimes differ in broader cellular functions. The enrichment of translation- and ribosome-related pathways in invasive-pike lakes points to differences in protein synthesis and cellular maintenance (Costa-Mattioli et al. 2009). In our study, a conservative interpretation is that brains of caring males from invasive-pike lakes differed in broad cellular processes linked to protein production that may reflect a different physiological or neural background state in which paternal decisions are occurring. This could be important for parenting in ecologically relevant ways, as paternal care is energetically demanding (Cooke et al. 2006). Any shift in neural cellular investment may influence how care is maintained, how risk is processed, or how readily behavior changes in response to disturbance. In that sense, translation and protein-folding-related pathways may be relevant to parenting indirectly if they contribute to the neural and physiological capacity that supports paternal care under different predator communities.

The enrichment of immune-related pathways in native predator-only lakes provides a complementary perspective. Males from native predator-only lakes showed relatively higher expression of immune-associated pathways than males from invasive-pike lakes. While we cannot infer health status directly, it does suggest that predator regime may be associated with differences in how individuals allocate among broad physiological processes. Immunology work has shown that immune activity is energetically expensive and often linked to broader life-history tradeoffs (Lochmiller and Deerenberg 2000). Although we cannot establish a direct allocation tradeoff, the contrast between immune-associated pathways in native predator-only lakes and translational or protein-synthesis pathways in invasive-pike lakes suggests that predator regimes may be associated with different baseline physiological properties. For parental animals, those physiological differences could matter when it comes to investment even if short-term care behavior remains relatively stable. Altogether, our results support the idea that invasive predator regimes may shape native organisms through indirect effects, including how parents maintain reproductive behavior and how their internal physiology is shaped during this process.

In contrast to the effects of predator community on gene expression, parental state was associated with minimal transcriptomic divergence, with only a single differentially expressed gene identified between parenting and non-parenting males. This weak signal suggests that, at the whole-brain level, parental care in the wild for these populations may not be structured by large shifts in gene expression, but instead may be regulated through more localized, transient, or circuit-specific mechanisms. Previous work in stickleback and other vertebrates has shown that parental behaviors are often mediated by changes in specific neuroendocrine and expression within discrete brain regions across the breeding cycle rather than whole-brain changes (O’Connell and Hofmann 2011; Bukhari et al. 2017, 2019). In contrast, the strong and consistent differences observed across predator environments suggest that ecological context exerts a more sustained influence on baseline non-parenting state. Together, these findings imply that while parental care is a critical behavioral phenotype, its molecular regulation may be subtle or context-dependent relative to broader environmental pressures, highlighting the importance of considering both spatial and temporal scales when linking behavior to gene expression.

Several limitations of this study should be acknowledged. First, the RNA-seq sample size was modest, as we had a subset of males for sequencing. Second, since this was a wild field study, lake type may covary with other ecological differences among lakes that were not measured or modeled, as power is limited in our gene-expression dataset and within-lake correlated expression is not able to be fully overcome as compared to our behavioral work in terms of model building for gene-expression analyses. These limitations also point clearly to future work. Sampling pike-invaded versus non-invaded lakes in this system is inherently associated with geography, as invasive northern pike are established in the Mat-Su Valley but are not present in the Kenai Peninsula (Alaska Department of Fish & Game, personal communication). Consequently, predator regime cannot be fully separated from regional environmental differences, including baseline paternal behavioral between lake type prior to predator exposure in the current field design, which we found to be different prior to exposure of our model predators. It is possible that unmeasured variation in limnology, microbial communities, or other ecological characteristics among regions could contribute to some of the observed transcriptomic or behavioral differences. Future work could help disentangle predator-associated and geographic sources of variation by incorporating additional invaded and non-invaded systems across broader spatial scales, integrating environmental measurements such as water chemistry and microbiome composition, and conducting common-garden or controlled predator-exposure experiments across populations. Additionally, transcriptomic analyses are correlational and cannot, on their own, establish mechanistic causation between predator regime and behavioral or physiological responses. Controlled common-garden experiments, repeated predator exposure manipulations, longitudinal sampling across reproductive stages, and targeted functional studies of candidate genes or pathways could help disentangle environmentally induced versus evolved responses and more directly test mechanistic links between predator exposure, gene expression, and parental behavior. Experimental manipulations involving exposure to native versus invasive predator cues across generations may be particularly useful for identifying whether observed transcriptomic responses contribute directly to altered parental behavior. This is particularly relevant because recent work in stickleback suggests that predator effects on parental systems can extend beyond the immediately exposed generation (Hellmann et al. 2024). Repeated behavioral observations across embryo development or across multiple predator encounters would help determine whether predator regime primarily affects acute responses, longer-term paternal effort, or the variability of care itself. Brain-region-specific expression analyses would also be especially informative, because whole-brain data may dilute stronger region-specific signals related to parenting, stress, and risk assessment.

In conclusion, our study suggests that invasive predator effects in this system are more evident in brain gene expression and baseline parent care compared to short-term changes in paternal behavior. Paternal behavior in the wild appeared variable and weakly impacted by acute predator exposure, whereas the transcriptome revealed organization associated with predator regime, particularly in pathways related to translation, protein folding, and immune function. These findings support the idea that indirect effects of invasive predators may be expressed differently across levels of biological organization and are a useful framework for studying those effects in natural systems.

## Supplementary Material

icag130_Supplemental_Files

## Data Availability

The data and code are available as supplementary material. Any updates on the code can be found on GitHub at the following link: https://github.com/earred3/Predator-communities-stickleback-RNAseq-Behavior-ICB2026/tree/main Sequencing data can be found on the NCBI BioProject database under the BioProject accession PRJNA1506108.
