# Supplementary material for: Predator Communities With Invasive Pike Are Associated With Changes in Parental Care and Brain Gene Expression in Wild Alaskan Stickleback
Source: Integr Comp Biol. 2026 Jul 28;66:icag130. doi: 10.1093/icb/icag130 (PMC13452083; doi:10.1093/icb/icag130)
Supplement: Supplementary file 1 [file icag130_supplemental_files.zip › icb-2026-0120-File006.docx]

| Model_code | Formula | K | logLik | AICc | delta | weight |
| --- | --- | --- | --- | --- | --- | --- |
| model12 | Fan_dif ~ Treatment + Lake_type + (1 \| Lake) | 6 | -183.614 | 381.938 | 0.000 | 0.550 |
| model15 | Fan_dif ~ Treatment + Lake_type + Nest_depth + (1 \| Lake) | 7 | -183.285 | 384.303 | 2.365 | 0.168 |
| model14 | Fan_dif ~ Treatment + Lake_type + Embryos_stage_simple + (1 \| Lake) | 8 | -181.744 | 384.454 | 2.516 | 0.156 |
| model13 | Fan_dif ~ Treatment * Lake_type + (1 \| Lake) | 8 | -182.446 | 385.857 | 3.920 | 0.077 |
| model6 | Fan_dif ~ Treatment * Lake_type + Embryos_stage_simple + (1 \| Lake) | 10 | -179.830 | 387.807 | 5.870 | 0.029 |
| model11 | Fan_dif ~ Treatment + Lake_type + Nest_depth + Percent_cover + Embryos_stage_simple + (1 \| Lake) | 10 | -180.967 | 390.082 | 8.144 | 0.009 |
| model10 | Fan_dif ~ Treatment + Lake_type + Bodycondition + Nest_depth + Percent_cover + Embryos_stage_simple + (1 \| Lake) | 11 | -179.709 | 391.572 | 9.635 | 0.004 |
| model7 | Fan_dif ~ Treatment * Lake_type + Nest_depth + Percent_cover + Embryos_stage_simple + (1 \| Lake) | 12 | -178.384 | 393.249 | 11.311 | 0.002 |
| model9 | Fan_dif ~ Treatment + Lake_type + Nest_depth + Percent_cover + Latency_to_return + Embryos_stage_simple + (1 \| Lake) | 11 | -180.710 | 393.573 | 11.635 | 0.002 |
| model8 | Fan_dif ~ Treatment + Lake_type + Bodycondition + Nest_depth + Percent_cover + Latency_to_return + Embryos_stage_simple + (1 \| Lake) | 12 | -179.281 | 395.042 | 13.104 | 0.001 |
| model5 | Fan_dif ~ Treatment * Lake_type + Bodycondition + Nest_depth + Percent_cover + Embryos_stage_simple + (1 \| Lake) | 13 | -177.583 | 396.333 | 14.396 | 0.000 |
| model4 | Fan_dif ~ Treatment * Lake_type + Bodycondition + Nest_depth + Latency_to_return + Embryos_stage_simple + (1 \| Lake) | 13 | -178.225 | 397.616 | 15.678 | 0.000 |
| model2 | Fan_dif ~ Treatment * Lake_type + Nest_depth + Percent_cover + Latency_to_return + Embryos_stage_simple + (1 \| Lake) | 13 | -178.303 | 397.772 | 15.835 | 0.000 |
| model3 | Fan_dif ~ Treatment * Lake_type + Bodycondition + Percent_cover + Latency_to_return + Embryos_stage_simple + (1 \| Lake) | 13 | -178.518 | 398.203 | 16.265 | 0.000 |
| Bigmodel | Fan_dif ~ Treatment * Lake_type + Bodycondition + Nest_depth + Percent_cover + Latency_to_return + Embryos_stage_simple + (1 \| Lake) | 14 | -177.401 | 401.062 | 19.125 | 0.000 |

Table S1. Model selection results for linear mixed-effects models evaluating predictors of change in male fanning behavior following predator exposure. Candidate models included biological meaningful combinations of predator treatment, lake type, nest depth, percent cover, embryo developmental stage, latency to return, and body condition, with lake included as a random intercept. Models are ranked by Akaike Information Criterion corrected for small sample size (AICc). ΔAICc represents the difference from the top-supported model, and model weights indicate relative support among candidate models.

Figure S1

Principal component analysis of transcriptomic variation across all samples.

Principal component analysis of normalized brain gene expression across all samples. Points represent individuals and are colored by parental state. Shapes indicate lake type (No pike lakes: native predator-only lakes vs. Pike lakes: lakes containing invasive pike). The first two principal components explain 26% and 18% of the total variance, respectively.

Figure S2. Fixed effects estimates from the top behavioral mixed-effects model.

Estimated effects and 95% confidence intervals for predictors included in the best-supported model explaining variation in male fanning behavior. Predictors include predator treatment, and lake type. The dashed vertical line indicates no effect.

Figure S3. Volcano plot of differential gene expression between lake types (native predator-only vs. native + invasive predator lakes). Each point represents a gene, with log2 fold change on the x-axis and –log10 adjusted p-value on the y-axis. Horizontal and vertical dashed lines indicate significance thresholds (adjusted p-value < 0.05 and |log2 fold change| ≥ 0.58). Genes exceeding both thresholds are highlighted in green.

Figure S4. Principal components analysis (PCA) of whole-brain gene expression profiles colored by lake. Each point represents an individual male stickleback. PC1 explained 26% of the total variance and PC2 explained 18% of the total variance in transcriptomic expression.

Figure S5. Latency for male threespined stickleback to return to the nest following presentation of predator models (char, northern pike, or trout). This measure provides an indicator of the immediate behavioral response to model presentation. Points represent individual males, with colors and shapes indicating predator treatment. Boxes represent the interquartile range with the median indicated by the horizontal line. Diamonds indicate the mean. The dashed horizontal line indicates immediate return to the nest (0 s).
