# Supplementary material for: Predator Communities With Invasive Pike Are Associated With Changes in Parental Care and Brain Gene Expression in Wild Alaskan Stickleback
Source: Integr Comp Biol. 2026 Jul 28;66:icag130. doi: 10.1093/icb/icag130 (PMC13452083; doi:10.1093/icb/icag130)
Supplement: Supplementary file 1 [file icag130_supplemental_files.zip › icb-2026-0120-File010.html]

Predator communities with invasive pike are associated with changes in parental care and brain gene expression in wild Alaskan stickleback


# Predator communities with invasive pike are associated with changes in parental care and brain gene expression in wild Alaskan stickleback

#### E. Arredondo, B. Byrd, L.R. Stein

#### 2026-07-17

# Behavioral data

## Overview

This section of code analyzes parental care behaviors performed by
stickleback males both before and after exposure to both native and
invasive predator models, across lakes with and without invasive
pike.

## Loading necessary packages and creating themes and labels

```
##Clear environment
rm(list = ls()); gc()
```

```
##           used (Mb) gc trigger (Mb) limit (Mb) max used (Mb)
## Ncells  625762 33.5    1430010 76.4         NA   715762 38.3
## Vcells 1190477  9.1    8388608 64.0      16384  2010648 15.4
```

```
# packages
library(librarian)
shelf(tidyverse, ggplot2, tibble, dplyr, tidyr, knitr, kableExtra, broom, broom.mixed, lme4, lmerTest, emmeans, car, performance, MuMIn, effectsize, sjPlot, ggeffects)
```

```
## 
##   The 'cran_repo' argument in shelf() was not set, so it will use
##   cran_repo = 'https://cran.r-project.org' by default.
## 
##   To avoid this message, set the 'cran_repo' argument to a CRAN
##   mirror URL (see https://cran.r-project.org/mirrors.html) or set
##   'quiet = TRUE'.
```

```
# theme / labels / colors
theme_paper <- function() {
  theme_classic(base_size = 14) +
    theme(
      plot.title = element_text(face = "bold", hjust = 0.5, size = 16),
      axis.title = element_text(face = "bold", size = 14),
      axis.text = element_text(size = 12, color = "black"),
      legend.title = element_text(face = "bold", size = 13),
      legend.text = element_text(size = 12),
      strip.background = element_rect(fill = "grey95", color = "grey70"),
      strip.text = element_text(face = "bold", size = 12)
    )
}

# Char = orange, Pike = green, Trout = darker salmon
pal_treatment <- c(
  "Char"  = "#E69F00",
  "Pike"  = "#009E73",
  "Trout" = "#D55E00"
)

pal_lake <- c(
  "Native predator only lakes" = "#0072B2",
  "Native + Invasive predator lakes" = "#CC79A7"
)

lake_label_map <- c(
  "No Pike" = "Native predator only lakes",
  "Pike"    = "Native + Invasive predator lakes"
)

treatment_shape_map <- c(
  "Char"  = 16,
  "Pike"  = 17,
  "Trout" = 15
)


# theme / labels / colors
theme_paper <- function() {
  theme_classic(base_size = 14) +
    theme(
      plot.title = element_text(face = "bold", hjust = 0.5, size = 16),
      axis.title = element_text(face = "bold", size = 14),
      axis.text = element_text(size = 12, color = "black"),
      legend.title = element_text(face = "bold", size = 13),
      legend.text = element_text(size = 12),
      strip.background = element_rect(fill = "grey95", color = "grey70"),
      strip.text = element_text(face = "bold", size = 12)
    )
}

# Char = orange, Pike = green, Trout = darker salmon
pal_treatment <- c(
  "Char"  = "#E69F00",
  "Pike"  = "#009E73",
  "Trout" = "#D55E00"
)

pal_lake <- c(
  "Native predator only lakes" = "#0072B2",
  "Native + Invasive predator lakes" = "#CC79A7"
)

lake_label_map <- c(
  "No Pike" = "Native predator only lakes",
  "Pike"    = "Native + Invasive predator lakes"
)

treatment_shape_map <- c(
  "Char"  = 16,
  "Pike"  = 17,
  "Trout" = 15
)
```

## Entering and cleaning data

```
# load in behavior data
behavior <- read.csv("AK2024_Data.csv")
```

```
#Cleaning behavior data

behavior <- behavior %>%
  dplyr::mutate(
    ID = trimws(as.character(ID)),
    Lake = trimws(as.character(Lake)),
    Stage = trimws(as.character(Stage)),
    Treatment = trimws(as.character(Treatment)),
    Lake_type = trimws(as.character(Lake_type)),
    Ecotype = trimws(as.character(Ecotype)),
    Embryos_stage = trimws(as.character(Embryos_stage)),
    Fanning_time = suppressWarnings(as.numeric(Fanning_time)),
    Bodycondition = suppressWarnings(as.numeric(Bodycondition)),
    Nest_depth = suppressWarnings(as.numeric(Nest_depth)),
    Percent_cover = suppressWarnings(as.numeric(Percent_cover)),
    Latency_return = suppressWarnings(as.numeric(Latency_return))
  ) %>%
  dplyr::mutate(
    Lake_type = dplyr::case_when(
      Lake_type %in% c("No Pike", "No pike", "no pike") ~ "No Pike",
      Lake_type %in% c("Pike", "pike") ~ "Pike",
      TRUE ~ Lake_type
    ),
    Treatment = dplyr::case_when(
      Treatment %in% c("Char", "char") ~ "Char",
      Treatment %in% c("Pike", "pike") ~ "Pike",
      Treatment %in% c("Trout", "trout") ~ "Trout",
      TRUE ~ Treatment
    ),
    Ecotype = dplyr::na_if(Ecotype, ""),
    Embryos_stage = dplyr::na_if(Embryos_stage, "")
  )

print(
  behavior %>%
    dplyr::count(Lake_type, Ecotype, Treatment, Stage, name = "count") %>%
    dplyr::arrange(Stage, Lake_type, Ecotype, Treatment)
)
```

```
##    Lake_type  Ecotype Treatment    Stage count
## 1    No Pike  Benthic      Char Baseline     2
## 2    No Pike  Benthic      Pike Baseline     4
## 3    No Pike  Benthic     Trout Baseline     4
## 4    No Pike Limnetic      Char Baseline     7
## 5    No Pike Limnetic      Pike Baseline     7
## 6    No Pike Limnetic     Trout Baseline     6
## 7       Pike  Benthic      Char Baseline     3
## 8       Pike  Benthic      Pike Baseline     3
## 9       Pike  Benthic     Trout Baseline     3
## 10      Pike Limnetic      Char Baseline     7
## 11      Pike Limnetic      Pike Baseline     7
## 12      Pike Limnetic     Trout Baseline     8
## 13   No Pike  Benthic      Char Predator     2
## 14   No Pike  Benthic      Pike Predator     4
## 15   No Pike  Benthic     Trout Predator     4
## 16   No Pike Limnetic      Char Predator     6
## 17   No Pike Limnetic      Pike Predator     6
## 18   No Pike Limnetic     Trout Predator     6
## 19      Pike  Benthic      Char Predator     3
## 20      Pike  Benthic      Pike Predator     3
## 21      Pike  Benthic     Trout Predator     3
## 22      Pike Limnetic      Char Predator     7
## 23      Pike Limnetic      Pike Predator     6
## 24      Pike Limnetic     Trout Predator     8
```

```
#We are interested in parenting behavior, so remove males that were fanning but did not have eggs in the nest

behavior %>%
  dplyr::filter(!is.na(Embryos_stage), Embryos_stage != "None") %>%
  droplevels() -> behavior
```

## Behaviora data inspection

Inspecting the distribution of time fanning, latency to return to the
nest, difference in time fanning (after - before model predator
exposure), including sample sizes.

```
# raw fanning histogram
p_raw_fanning_hist <- ggplot(
  behavior %>% dplyr::filter(!is.na(Fanning_time)),
  aes(x = Fanning_time)
) +
  geom_histogram(bins = 20, fill = "grey70", color = "black") +
  labs(
    title = "Histogram of raw fanning time",
    x = "Fanning time (s)",
    y = "Count"
  ) +
  theme_paper()

print(p_raw_fanning_hist)
```

```
# raw latency to return histogram
p_raw_return_hist <- ggplot(
  behavior %>% dplyr::filter(!is.na(Latency_return)),
  aes(x = Latency_return)
) +
  geom_histogram(bins = 20, fill = "grey70", color = "black") +
  labs(
    title = "Histogram of raw latency to return",
    x = "Latency to return (s)",
    y = "Count"
  ) +
  theme_paper()

print(p_raw_return_hist)
```

```
# one row per ID for ID-level covariates
id_info <- behavior %>%
  dplyr::group_by(ID) %>%
  dplyr::summarise(
    Lake_type = dplyr::first(Lake_type[!is.na(Lake_type) & Lake_type != ""]),
    Ecotype = dplyr::first(Ecotype[!is.na(Ecotype) & Ecotype != ""]),
    Treatment = dplyr::first(Treatment[!is.na(Treatment) & Treatment != ""]),
    Lake = dplyr::first(Lake[!is.na(Lake) & Lake != ""]),
    Embryos_stage = dplyr::first(Embryos_stage[!is.na(Embryos_stage) & Embryos_stage != ""]),
    Bodycondition = dplyr::first(Bodycondition[!is.na(Bodycondition)]),
    Nest_depth = dplyr::first(Nest_depth[!is.na(Nest_depth)]),
    Percent_cover = dplyr::first(Percent_cover[!is.na(Percent_cover)]),
    Latency_to_return = dplyr::first(Latency_return[Stage == "Predator" & !is.na(Latency_return)]),
    .groups = "drop"
  )

# Create difference in fanning in dataframe
Fan_Dif <- behavior %>%
  tidyr::pivot_wider(
    id_cols = ID,
    names_from = Stage,
    values_from = Fanning_time
  ) %>%
  dplyr::left_join(id_info, by = "ID") %>%
  dplyr::filter(!is.na(Baseline), !is.na(Predator)) %>%
  dplyr::mutate(
    Fan_dif = Predator - Baseline,
    Embryos_stage_simple = dplyr::case_when(
      Embryos_stage %in% c("Fry", "Mixed") ~ "Other",
      is.na(Embryos_stage) ~ NA_character_,
      TRUE ~ Embryos_stage
    ),
    Lake_type_label = dplyr::recode(Lake_type, !!!lake_label_map)
  ) %>%
  dplyr::mutate(
    Lake_type = factor(Lake_type, levels = c("No Pike", "Pike")),
    Lake_type_label = factor(
      Lake_type_label,
      levels = c("Native predator only lakes", "Native + Invasive predator lakes")
    ),
    Ecotype = factor(Ecotype),
    Treatment = factor(Treatment, levels = c("Char", "Pike", "Trout")),
    Embryos_stage_simple = factor(Embryos_stage_simple),
    Lake = factor(Lake)
  )

print(
  Fan_Dif %>%
    dplyr::count(Lake_type, Ecotype, Treatment, name = "count") %>%
    dplyr::arrange(Lake_type, Ecotype, Treatment)
)
```

```
## # A tibble: 12 × 4
##    Lake_type Ecotype  Treatment count
##    <fct>     <fct>    <fct>     <int>
##  1 No Pike   Benthic  Char          1
##  2 No Pike   Benthic  Pike          2
##  3 No Pike   Benthic  Trout         2
##  4 No Pike   Limnetic Char          4
##  5 No Pike   Limnetic Pike          5
##  6 No Pike   Limnetic Trout         2
##  7 Pike      Benthic  Char          3
##  8 Pike      Benthic  Pike          3
##  9 Pike      Benthic  Trout         3
## 10 Pike      Limnetic Char          5
## 11 Pike      Limnetic Pike          3
## 12 Pike      Limnetic Trout         7
```

```
# histogram of Fan_dif
p_fandif_hist <- ggplot(
  Fan_Dif %>% dplyr::filter(!is.na(Fan_dif)),
  aes(x = Fan_dif)
) +
  geom_histogram(bins = 20, fill = "grey70", color = "black") +
  labs(
    title = "Histogram of change in fanning time",
    x = "Predator-induced change in fanning time (s)",
    y = "Count"
  ) +
  theme_paper()

print(p_fandif_hist)
```

```
# egg-only behavior dataset
aicc_data <- Fan_Dif %>%
  dplyr::filter(
    !is.na(Fan_dif),
    !is.na(Treatment),
    !is.na(Lake_type),
    !is.na(Ecotype),
    !is.na(Lake),
    !is.na(Bodycondition),
    !is.na(Nest_depth),
    !is.na(Percent_cover),
    !is.na(Embryos_stage_simple),
    Embryos_stage_simple != "None"
  ) %>%
  droplevels()

# sample sizes
nrow(aicc_data)
```

```
## [1] 38
```

```
table(aicc_data$Embryos_stage_simple)
```

```
## 
##    Eyes No eyes   Other 
##      11      18       9
```

```
table(aicc_data$Lake_type, aicc_data$Treatment)
```

```
##          
##           Char Pike Trout
##   No Pike    5    7     4
##   Pike       7    6     9
```

```
table(aicc_data$Ecotype)
```

```
## 
##  Benthic Limnetic 
##       14       24
```

```
table(aicc_data$Ecotype, aicc_data$Lake_type)
```

```
##           
##            No Pike Pike
##   Benthic        5    9
##   Limnetic      11   13
```

```
table(aicc_data$Ecotype, aicc_data$Treatment)
```

```
##           
##            Char Pike Trout
##   Benthic     4    5     5
##   Limnetic    8    8     8
```

## Checking effect of ecotype

```
# Quick ecotype models


# simple model: does ecotype predict Fan_dif?
eco_lm1 <- lm(Fan_dif ~ Ecotype, data = aicc_data)
summary(eco_lm1)
```

```
## 
## Call:
## lm(formula = Fan_dif ~ Ecotype, data = aicc_data)
## 
## Residuals:
##     Min      1Q  Median      3Q     Max 
## -76.670 -15.464   1.605  22.093  66.734 
## 
## Coefficients:
##                 Estimate Std. Error t value Pr(>|t|)
## (Intercept)        6.390      8.926   0.716    0.479
## EcotypeLimnetic   -8.357     11.232  -0.744    0.462
## 
## Residual standard error: 33.4 on 36 degrees of freedom
## Multiple R-squared:  0.01515,    Adjusted R-squared:  -0.01221 
## F-statistic: 0.5536 on 1 and 36 DF,  p-value: 0.4617
```

```
anova(eco_lm1)
```

```
## Analysis of Variance Table
## 
## Response: Fan_dif
##           Df Sum Sq Mean Sq F value Pr(>F)
## Ecotype    1    618  617.57  0.5536 0.4617
## Residuals 36  40157 1115.46
```

```
# ecotype + treatment
eco_lm2 <- lm(Fan_dif ~ Ecotype + Treatment, data = aicc_data)
summary(eco_lm2)
```

```
## 
## Call:
## lm(formula = Fan_dif ~ Ecotype + Treatment, data = aicc_data)
## 
## Residuals:
##     Min      1Q  Median      3Q     Max 
## -81.910 -18.743  -1.578  16.194  62.484 
## 
## Coefficients:
##                 Estimate Std. Error t value Pr(>|t|)  
## (Intercept)       -8.471     11.987  -0.707   0.4846  
## EcotypeLimnetic   -7.367     11.038  -0.667   0.5090  
## TreatmentPike     22.502     13.135   1.713   0.0958 .
## TreatmentTrout    19.111     13.135   1.455   0.1549  
## ---
## Signif. codes:  0 '***' 0.001 '**' 0.01 '*' 0.05 '.' 0.1 ' ' 1
## 
## Residual standard error: 32.78 on 34 degrees of freedom
## Multiple R-squared:  0.1039, Adjusted R-squared:  0.02488 
## F-statistic: 1.315 on 3 and 34 DF,  p-value: 0.2856
```

```
anova(eco_lm2)
```

```
## Analysis of Variance Table
## 
## Response: Fan_dif
##           Df Sum Sq Mean Sq F value Pr(>F)
## Ecotype    1    618  617.57  0.5747 0.4536
## Treatment  2   3621 1810.25  1.6846 0.2006
## Residuals 34  36536 1074.59
```

```
# ecotype + lake type + treatment
eco_lm3 <- lm(Fan_dif ~ Ecotype + Lake_type + Treatment, data = aicc_data)
summary(eco_lm3)
```

```
## 
## Call:
## lm(formula = Fan_dif ~ Ecotype + Lake_type + Treatment, data = aicc_data)
## 
## Residuals:
##     Min      1Q  Median      3Q     Max 
## -76.406 -16.370  -2.171  19.044  66.374 
## 
## Coefficients:
##                 Estimate Std. Error t value Pr(>|t|)
## (Intercept)        1.863     13.748   0.136    0.893
## EcotypeLimnetic   -8.981     10.913  -0.823    0.416
## Lake_typePike    -15.871     10.854  -1.462    0.153
## TreatmentPike     20.486     12.994   1.577    0.124
## TreatmentTrout    20.758     12.970   1.600    0.119
## 
## Residual standard error: 32.25 on 33 degrees of freedom
## Multiple R-squared:  0.1585, Adjusted R-squared:  0.05645 
## F-statistic: 1.553 on 4 and 33 DF,  p-value: 0.2098
```

```
anova(eco_lm3)
```

```
## Analysis of Variance Table
## 
## Response: Fan_dif
##           Df Sum Sq Mean Sq F value Pr(>F)
## Ecotype    1    618  617.57  0.5939 0.4464
## Lake_type  1   2360 2360.10  2.2698 0.1414
## Treatment  2   3483 1741.68  1.6750 0.2028
## Residuals 33  34313 1039.79
```

```
# mixed model version with lake as random intercept
eco_lmer1 <- lmerTest::lmer(Fan_dif ~ Ecotype + (1 | Lake), data = aicc_data)
```

```
## boundary (singular) fit: see help('isSingular')
```

```
summary(eco_lmer1)
```

```
## Linear mixed model fit by REML. t-tests use Satterthwaite's method [
## lmerModLmerTest]
## Formula: Fan_dif ~ Ecotype + (1 | Lake)
##    Data: aicc_data
## 
## REML criterion at convergence: 360.6
## 
## Scaled residuals: 
##      Min       1Q   Median       3Q      Max 
## -2.29561 -0.46303  0.04807  0.66150  1.99810 
## 
## Random effects:
##  Groups   Name        Variance Std.Dev.
##  Lake     (Intercept)    0      0.0    
##  Residual             1115     33.4    
## Number of obs: 38, groups:  Lake, 6
## 
## Fixed effects:
##                 Estimate Std. Error     df t value Pr(>|t|)
## (Intercept)        6.390      8.926 36.000   0.716    0.479
## EcotypeLimnetic   -8.357     11.232 36.000  -0.744    0.462
## 
## Correlation of Fixed Effects:
##             (Intr)
## EcotypLmntc -0.795
## optimizer (nloptwrap) convergence code: 0 (OK)
## boundary (singular) fit: see help('isSingular')
```

```
eco_lmer2 <- lmerTest::lmer(Fan_dif ~ Ecotype + Treatment + (1 | Lake), data = aicc_data)
```

```
## boundary (singular) fit: see help('isSingular')
```

```
summary(eco_lmer2)
```

```
## Linear mixed model fit by REML. t-tests use Satterthwaite's method [
## lmerModLmerTest]
## Formula: Fan_dif ~ Ecotype + Treatment + (1 | Lake)
##    Data: aicc_data
## 
## REML criterion at convergence: 343.6
## 
## Scaled residuals: 
##      Min       1Q   Median       3Q      Max 
## -2.49871 -0.57175 -0.04813  0.49401  1.90612 
## 
## Random effects:
##  Groups   Name        Variance Std.Dev.
##  Lake     (Intercept)    0      0.00   
##  Residual             1075     32.78   
## Number of obs: 38, groups:  Lake, 6
## 
## Fixed effects:
##                 Estimate Std. Error     df t value Pr(>|t|)  
## (Intercept)       -8.471     11.987 34.000  -0.707   0.4846  
## EcotypeLimnetic   -7.367     11.038 34.000  -0.667   0.5090  
## TreatmentPike     22.502     13.135 34.000   1.713   0.0958 .
## TreatmentTrout    19.111     13.135 34.000   1.455   0.1549  
## ---
## Signif. codes:  0 '***' 0.001 '**' 0.01 '*' 0.05 '.' 0.1 ' ' 1
## 
## Correlation of Fixed Effects:
##             (Intr) EctypL TrtmnP
## EcotypLmntc -0.614              
## TreatmentPk -0.595  0.043       
## TreatmntTrt -0.595  0.043  0.521
## optimizer (nloptwrap) convergence code: 0 (OK)
## boundary (singular) fit: see help('isSingular')
```

```
eco_lmer3 <- lmerTest::lmer(Fan_dif ~ Ecotype + Lake_type + Treatment + (1 | Lake), data = aicc_data)
```

```
## boundary (singular) fit: see help('isSingular')
```

```
summary(eco_lmer3)
```

```
## Linear mixed model fit by REML. t-tests use Satterthwaite's method [
## lmerModLmerTest]
## Formula: Fan_dif ~ Ecotype + Lake_type + Treatment + (1 | Lake)
##    Data: aicc_data
## 
## REML criterion at convergence: 334.9
## 
## Scaled residuals: 
##      Min       1Q   Median       3Q      Max 
## -2.36947 -0.50766 -0.06733  0.59058  2.05837 
## 
## Random effects:
##  Groups   Name        Variance Std.Dev.
##  Lake     (Intercept)    0      0.00   
##  Residual             1040     32.25   
## Number of obs: 38, groups:  Lake, 6
## 
## Fixed effects:
##                 Estimate Std. Error      df t value Pr(>|t|)
## (Intercept)        1.863     13.748  33.000   0.136    0.893
## EcotypeLimnetic   -8.981     10.913  33.000  -0.823    0.416
## Lake_typePike    -15.871     10.854  33.000  -1.462    0.153
## TreatmentPike     20.486     12.994  33.000   1.577    0.124
## TreatmentTrout    20.758     12.970  33.000   1.600    0.119
## 
## Correlation of Fixed Effects:
##             (Intr) EctypL Lk_tyP TrtmnP
## EcotypLmntc -0.576                     
## Lake_typePk -0.514  0.101              
## TreatmentPk -0.562  0.053  0.106       
## TreatmntTrt -0.464  0.034 -0.087  0.507
## optimizer (nloptwrap) convergence code: 0 (OK)
## boundary (singular) fit: see help('isSingular')
```

```
# compare quick models
AIC(eco_lm1, eco_lm2, eco_lm3, eco_lmer1, eco_lmer2, eco_lmer3)
```

```
##           df      AIC
## eco_lm1    3 378.4317
## eco_lm2    5 378.8413
## eco_lm3    6 378.4559
## eco_lmer1  4 368.5936
## eco_lmer2  6 355.5894
## eco_lmer3  7 348.8632
```

```
# Quick ecotype plots


# boxplot: Fan_dif by ecotype
p_ecotype_box <- ggplot(
  aicc_data,
  aes(x = Ecotype, y = Fan_dif)
) +
  geom_hline(yintercept = 0, linetype = "dashed", color = "grey40") +
  geom_boxplot(outlier.shape = NA, fill = "grey80", color = "black") +
  geom_jitter(width = 0.12, size = 2.5, alpha = 0.8) +
  labs(
    title = "Change in fanning time by ecotype",
    x = "Ecotype",
    y = "change in fanning time (s)"
  ) +
  theme_paper()

print(p_ecotype_box)
```

```
# boxplot: Fan_dif by ecotype, colored by treatment
p_ecotype_treatment_box <- ggplot(
  aicc_data,
  aes(x = Ecotype, y = Fan_dif, color = Treatment)
) +
  geom_hline(yintercept = 0, linetype = "dashed", color = "grey40") +
  geom_boxplot(outlier.shape = NA) +
  geom_jitter(
    aes(shape = Treatment),
    position = position_jitterdodge(jitter.width = 0.12, dodge.width = 0.75),
    size = 2.5,
    alpha = 0.8
  ) +
  scale_color_manual(values = pal_treatment) +
  scale_shape_manual(values = treatment_shape_map) +
  labs(
    title = "Change in fanning time by ecotype and treatment",
    x = "Ecotype",
    y = "Predator-induced change in fanning time (s)",
    color = "Treatment",
    shape = "Treatment"
  ) +
  theme_paper()

print(p_ecotype_treatment_box)
```

```
# boxplot: Fan_dif by ecotype, faceted by lake type
p_ecotype_laketype_box <- ggplot(
  aicc_data,
  aes(x = Ecotype, y = Fan_dif, color = Treatment)
) +
  geom_hline(yintercept = 0, linetype = "dashed", color = "grey40") +
  geom_boxplot(outlier.shape = NA) +
  geom_jitter(
    aes(shape = Treatment),
    position = position_jitterdodge(jitter.width = 0.12, dodge.width = 0.75),
    size = 2.5,
    alpha = 0.8
  ) +
  scale_color_manual(values = pal_treatment) +
  scale_shape_manual(values = treatment_shape_map) +
  facet_wrap(~ Lake_type_label) +
  labs(
    title = "Change in fanning time by ecotype, treatment, and lake type",
    x = "Ecotype",
    y = "Predator-induced change in fanning time (s)",
    color = "Treatment",
    shape = "Treatment"
  ) +
  theme_paper()

print(p_ecotype_laketype_box)
```

```
# mixed model version with lake as random intercept
base_lmer1 <- lmerTest::lmer(Baseline ~ Ecotype + (1 | Lake), data = aicc_data)
summary(base_lmer1)
```

```
## Linear mixed model fit by REML. t-tests use Satterthwaite's method [
## lmerModLmerTest]
## Formula: Baseline ~ Ecotype + (1 | Lake)
##    Data: aicc_data
## 
## REML criterion at convergence: 379.9
## 
## Scaled residuals: 
##      Min       1Q   Median       3Q      Max 
## -1.49900 -0.83325 -0.02229  0.71829  2.02617 
## 
## Random effects:
##  Groups   Name        Variance Std.Dev.
##  Lake     (Intercept)  272.5   16.51   
##  Residual             1776.7   42.15   
## Number of obs: 38, groups:  Lake, 6
## 
## Fixed effects:
##                 Estimate Std. Error      df t value Pr(>|t|)  
## (Intercept)       68.528     16.393   2.785    4.18   0.0288 *
## EcotypeLimnetic  -19.134     20.352   2.937   -0.94   0.4179  
## ---
## Signif. codes:  0 '***' 0.001 '**' 0.01 '*' 0.05 '.' 0.1 ' ' 1
## 
## Correlation of Fixed Effects:
##             (Intr)
## EcotypLmntc -0.806
```

```
base_lmer2 <- lmerTest::lmer(Baseline ~ Ecotype + Treatment + (1 | Lake), data = aicc_data)
summary(base_lmer2)
```

```
## Linear mixed model fit by REML. t-tests use Satterthwaite's method [
## lmerModLmerTest]
## Formula: Baseline ~ Ecotype + Treatment + (1 | Lake)
##    Data: aicc_data
## 
## REML criterion at convergence: 361.5
## 
## Scaled residuals: 
##      Min       1Q   Median       3Q      Max 
## -1.83970 -0.69216 -0.01935  0.63594  1.70632 
## 
## Random effects:
##  Groups   Name        Variance Std.Dev.
##  Lake     (Intercept)   88.84   9.425  
##  Residual             1768.08  42.049  
## Number of obs: 38, groups:  Lake, 6
## 
## Fixed effects:
##                 Estimate Std. Error      df t value Pr(>|t|)   
## (Intercept)       68.948     16.892   5.637   4.082  0.00741 **
## EcotypeLimnetic  -20.073     16.478   2.344  -1.218  0.33150   
## TreatmentPike    -15.696     16.883  29.909  -0.930  0.35996   
## TreatmentTrout    17.589     16.937  31.426   1.038  0.30697   
## ---
## Signif. codes:  0 '***' 0.001 '**' 0.01 '*' 0.05 '.' 0.1 ' ' 1
## 
## Correlation of Fixed Effects:
##             (Intr) EctypL TrtmnP
## EcotypLmntc -0.652              
## TreatmentPk -0.545  0.038       
## TreatmntTrt -0.545  0.043  0.514
```

```
base_lmer3 <- lmerTest::lmer(Baseline ~ Ecotype + Lake_type + Treatment + (1 | Lake), data = aicc_data)
```

```
## boundary (singular) fit: see help('isSingular')
```

```
summary(base_lmer3)
```

```
## Linear mixed model fit by REML. t-tests use Satterthwaite's method [
## lmerModLmerTest]
## Formula: Baseline ~ Ecotype + Lake_type + Treatment + (1 | Lake)
##    Data: aicc_data
## 
## REML criterion at convergence: 350
## 
## Scaled residuals: 
##     Min      1Q  Median      3Q     Max 
## -2.0385 -0.7086  0.1287  0.6043  1.7583 
## 
## Random effects:
##  Groups   Name        Variance Std.Dev.
##  Lake     (Intercept)    0      0.00   
##  Residual             1647     40.58   
## Number of obs: 38, groups:  Lake, 6
## 
## Fixed effects:
##                 Estimate Std. Error     df t value Pr(>|t|)   
## (Intercept)        50.71      17.30  33.00   2.931  0.00609 **
## EcotypeLimnetic   -18.22      13.73  33.00  -1.326  0.19382   
## Lake_typePike      29.67      13.66  33.00   2.172  0.03712 * 
## TreatmentPike     -12.71      16.35  33.00  -0.777  0.44241   
## TreatmentTrout     15.26      16.32  33.00   0.935  0.35656   
## ---
## Signif. codes:  0 '***' 0.001 '**' 0.01 '*' 0.05 '.' 0.1 ' ' 1
## 
## Correlation of Fixed Effects:
##             (Intr) EctypL Lk_tyP TrtmnP
## EcotypLmntc -0.576                     
## Lake_typePk -0.514  0.101              
## TreatmentPk -0.562  0.053  0.106       
## TreatmntTrt -0.464  0.034 -0.087  0.507
## optimizer (nloptwrap) convergence code: 0 (OK)
## boundary (singular) fit: see help('isSingular')
```

```
# compare quick models
AIC(eco_lm1, eco_lm2, eco_lm3, eco_lmer1, eco_lmer2, eco_lmer3)
```

```
##           df      AIC
## eco_lm1    3 378.4317
## eco_lm2    5 378.8413
## eco_lm3    6 378.4559
## eco_lmer1  4 368.5936
## eco_lmer2  6 355.5894
## eco_lmer3  7 348.8632
```

```
# Quick ecotype plots


# boxplot: Fan_dif by ecotype
p_ecotype_box <- ggplot(
  aicc_data,
  aes(x = Ecotype, y = Fan_dif)
) +
  geom_hline(yintercept = 0, linetype = "dashed", color = "grey40") +
  geom_boxplot(outlier.shape = NA, fill = "grey80", color = "black") +
  geom_jitter(width = 0.12, size = 2.5, alpha = 0.8) +
  labs(
    title = "Change in fanning time by ecotype",
    x = "Ecotype",
    y = "change in fanning time (s)"
  ) +
  theme_paper()

print(p_ecotype_box)
```

```
# boxplot: Fan_dif by ecotype, colored by treatment
p_ecotype_treatment_box <- ggplot(
  aicc_data,
  aes(x = Ecotype, y = Fan_dif, color = Treatment)
) +
  geom_hline(yintercept = 0, linetype = "dashed", color = "grey40") +
  geom_boxplot(outlier.shape = NA) +
  geom_jitter(
    aes(shape = Treatment),
    position = position_jitterdodge(jitter.width = 0.12, dodge.width = 0.75),
    size = 2.5,
    alpha = 0.8
  ) +
  scale_color_manual(values = pal_treatment) +
  scale_shape_manual(values = treatment_shape_map) +
  labs(
    title = "Change in fanning time by ecotype and treatment",
    x = "Ecotype",
    y = "Predator-induced change in fanning time (s)",
    color = "Treatment",
    shape = "Treatment"
  ) +
  theme_paper()

print(p_ecotype_treatment_box)
```

```
# boxplot: Fan_dif by ecotype, faceted by lake type
baseline_ecotype_laketype_box <- ggplot(
  aicc_data,
  aes(x = Ecotype, y = Baseline, color = Treatment)
) +
  geom_hline(yintercept = 0, linetype = "dashed", color = "grey40") +
  geom_boxplot(outlier.shape = NA) +
  geom_jitter(
    aes(shape = Treatment),
    position = position_jitterdodge(jitter.width = 0.12, dodge.width = 0.75),
    size = 2.5,
    alpha = 0.8
  ) +
  scale_color_manual(values = pal_treatment) +
  scale_shape_manual(values = treatment_shape_map) +
  facet_wrap(~ Lake_type_label) +
  labs(
    title = "Change in fanning time by ecotype, treatment, and lake type",
    x = "Ecotype",
    y = "change in fanning time (s)",
    color = "Treatment",
    shape = "Treatment"
  ) +
  theme_paper()

print(baseline_ecotype_laketype_box)
```

## AICc Model Comparison: Difference in time fanning

```
# behavior model set
# all models use same dataset
# each model is biologically meaningful and each model has lake type and treatment as those two effects are crucial for the main question

Bigmodel <- lmer(
  Fan_dif ~ Treatment * Lake_type + Bodycondition + Nest_depth +
    Percent_cover + Latency_to_return + Embryos_stage_simple + (1 | Lake),
  data = aicc_data,
  REML = FALSE
)
```

```
## boundary (singular) fit: see help('isSingular')
```

```
model2 <- lmer(
  Fan_dif ~ Treatment * Lake_type + Nest_depth + Percent_cover +
    Latency_to_return + Embryos_stage_simple + (1 | Lake),
  data = aicc_data, REML = FALSE
)
```

```
## boundary (singular) fit: see help('isSingular')
```

```
model3 <- lmer(
  Fan_dif ~ Treatment * Lake_type + Bodycondition + Percent_cover +
    Latency_to_return + Embryos_stage_simple + (1 | Lake),
  data = aicc_data, REML = FALSE
)
```

```
## boundary (singular) fit: see help('isSingular')
```

```
model4 <- lmer(
  Fan_dif ~ Treatment * Lake_type + Bodycondition + Nest_depth +
    Latency_to_return + Embryos_stage_simple + (1 | Lake),
  data = aicc_data, REML = FALSE
)
```

```
## boundary (singular) fit: see help('isSingular')
```

```
model5 <- lmer(
  Fan_dif ~ Treatment * Lake_type + Bodycondition + Nest_depth +
    Percent_cover + Embryos_stage_simple + (1 | Lake),
  data = aicc_data, REML = FALSE
)
```

```
## boundary (singular) fit: see help('isSingular')
```

```
model6 <- lmer(
  Fan_dif ~ Treatment * Lake_type + Embryos_stage_simple + (1 | Lake),
  data = aicc_data, REML = FALSE
)
```

```
## boundary (singular) fit: see help('isSingular')
```

```
model7 <- lmer(
  Fan_dif ~ Treatment * Lake_type + Nest_depth + Percent_cover +
    Embryos_stage_simple + (1 | Lake),
  data = aicc_data, REML = FALSE
)
```

```
## boundary (singular) fit: see help('isSingular')
```

```
model8 <- lmer(
  Fan_dif ~ Treatment + Lake_type + Bodycondition + Nest_depth +
    Percent_cover + Latency_to_return + Embryos_stage_simple + (1 | Lake),
  data = aicc_data, REML = FALSE
)
```

```
## boundary (singular) fit: see help('isSingular')
```

```
model9 <- lmer(
  Fan_dif ~ Treatment + Lake_type + Nest_depth + Percent_cover +
    Latency_to_return + Embryos_stage_simple + (1 | Lake),
  data = aicc_data, REML = FALSE
)
```

```
## boundary (singular) fit: see help('isSingular')
```

```
model10 <- lmer(
  Fan_dif ~ Treatment + Lake_type + Bodycondition + Nest_depth +
    Percent_cover + Embryos_stage_simple + (1 | Lake),
  data = aicc_data, REML = FALSE
)
```

```
## boundary (singular) fit: see help('isSingular')
```

```
model11 <- lmer(
  Fan_dif ~ Treatment + Lake_type + Nest_depth + Percent_cover +
    Embryos_stage_simple + (1 | Lake),
  data = aicc_data, REML = FALSE
)
```

```
## boundary (singular) fit: see help('isSingular')
```

```
model12 <- lmer(
  Fan_dif ~ Treatment + Lake_type + (1 | Lake),
  data = aicc_data, REML = FALSE
)
```

```
## boundary (singular) fit: see help('isSingular')
```

```
model13 <- lmer(
  Fan_dif ~ Treatment * Lake_type + (1 | Lake),
  data = aicc_data, REML = FALSE
)
```

```
## boundary (singular) fit: see help('isSingular')
```

```
model14 <- lmer(
  Fan_dif ~ Treatment + Lake_type + Embryos_stage_simple + (1 | Lake),
  data = aicc_data, REML = FALSE
)
```

```
## boundary (singular) fit: see help('isSingular')
```

```
  model15 <- lmer(
  Fan_dif ~ Treatment + Lake_type + Nest_depth + (1 | Lake),
  data = aicc_data, REML = FALSE
  )
```

```
## boundary (singular) fit: see help('isSingular')
```

```
  model16 <- lmer(
    Fan_dif ~1 + (1 | Lake),
  data = aicc_data,
  REML = FALSE
)
```

```
## boundary (singular) fit: see help('isSingular')
```

```
behavior_model_list <- list(
  Bigmodel = Bigmodel,
  model2 = model2,
  model3 = model3,
  model4 = model4,
  model5 = model5,
  model6 = model6,
  model7 = model7,
  model8 = model8,
  model9 = model9,
  model10 = model10,
  model11 = model11,
  model12 = model12,
  model13 = model13,
  model14 = model14,
  model15 = model15,
  model16 = model16
  
)

#Double check each model has same n
print(sapply(behavior_model_list, nobs))
```

```
## Bigmodel   model2   model3   model4   model5   model6   model7   model8 
##       38       38       38       38       38       38       38       38 
##   model9  model10  model11  model12  model13  model14  model15  model16 
##       38       38       38       38       38       38       38       38
```

```
aic_table <- MuMIn::model.sel(behavior_model_list) %>%
  as.data.frame() %>%
  tibble::rownames_to_column("Model_code") %>%
  dplyr::mutate(
    Formula = vapply(
      behavior_model_list,
      function(x) {
        f <- paste(deparse(stats::formula(x)), collapse = " ")
        f <- gsub("\\s+", " ", f)
        f
      },
      character(1)
    )[Model_code]
  ) %>%
  dplyr::select(Model_code, Formula, df, logLik, AICc, delta, weight) %>%
  dplyr::arrange(AICc)

print(
  knitr::kable(
    aic_table,
    digits = 3,
    escape = FALSE,
    caption = "AICc comparison of candidate behavioral models"
  ) %>%
    kableExtra::kable_styling(full_width = FALSE)
)
```

```
## <table class="table" style="color: black; width: auto !important; margin-left: auto; margin-right: auto;">
## <caption>AICc comparison of candidate behavioral models</caption>
##  <thead>
##   <tr>
##    <th style="text-align:left;"> Model_code </th>
##    <th style="text-align:left;"> Formula </th>
##    <th style="text-align:right;"> df </th>
##    <th style="text-align:right;"> logLik </th>
##    <th style="text-align:right;"> AICc </th>
##    <th style="text-align:right;"> delta </th>
##    <th style="text-align:right;"> weight </th>
##   </tr>
##  </thead>
## <tbody>
##   <tr>
##    <td style="text-align:left;"> model16 </td>
##    <td style="text-align:left;"> Fan_dif ~ 1 + (1 &amp;#124; Lake) </td>
##    <td style="text-align:right;"> 3 </td>
##    <td style="text-align:right;"> -186.506 </td>
##    <td style="text-align:right;"> 379.718 </td>
##    <td style="text-align:right;"> 0.000 </td>
##    <td style="text-align:right;"> 6.251392e-01 </td>
##   </tr>
##   <tr>
##    <td style="text-align:left;"> model12 </td>
##    <td style="text-align:left;"> Fan_dif ~ Treatment + Lake_type + (1 &amp;#124; Lake) </td>
##    <td style="text-align:right;"> 6 </td>
##    <td style="text-align:right;"> -183.614 </td>
##    <td style="text-align:right;"> 381.938 </td>
##    <td style="text-align:right;"> 2.220 </td>
##    <td style="text-align:right;"> 2.060203e-01 </td>
##   </tr>
##   <tr>
##    <td style="text-align:left;"> model15 </td>
##    <td style="text-align:left;"> Fan_dif ~ Treatment + Lake_type + Nest_depth + (1 &amp;#124; Lake) </td>
##    <td style="text-align:right;"> 7 </td>
##    <td style="text-align:right;"> -183.285 </td>
##    <td style="text-align:right;"> 384.303 </td>
##    <td style="text-align:right;"> 4.585 </td>
##    <td style="text-align:right;"> 6.313666e-02 </td>
##   </tr>
##   <tr>
##    <td style="text-align:left;"> model14 </td>
##    <td style="text-align:left;"> Fan_dif ~ Treatment + Lake_type + Embryos_stage_simple + (1 &amp;#124; Lake) </td>
##    <td style="text-align:right;"> 8 </td>
##    <td style="text-align:right;"> -181.744 </td>
##    <td style="text-align:right;"> 384.454 </td>
##    <td style="text-align:right;"> 4.736 </td>
##    <td style="text-align:right;"> 5.854331e-02 </td>
##   </tr>
##   <tr>
##    <td style="text-align:left;"> model13 </td>
##    <td style="text-align:left;"> Fan_dif ~ Treatment * Lake_type + (1 &amp;#124; Lake) </td>
##    <td style="text-align:right;"> 8 </td>
##    <td style="text-align:right;"> -182.446 </td>
##    <td style="text-align:right;"> 385.857 </td>
##    <td style="text-align:right;"> 6.140 </td>
##    <td style="text-align:right;"> 2.902331e-02 </td>
##   </tr>
##   <tr>
##    <td style="text-align:left;"> model6 </td>
##    <td style="text-align:left;"> Fan_dif ~ Treatment * Lake_type + Embryos_stage_simple + (1 &amp;#124; Lake) </td>
##    <td style="text-align:right;"> 10 </td>
##    <td style="text-align:right;"> -179.830 </td>
##    <td style="text-align:right;"> 387.807 </td>
##    <td style="text-align:right;"> 8.090 </td>
##    <td style="text-align:right;"> 1.094679e-02 </td>
##   </tr>
##   <tr>
##    <td style="text-align:left;"> model11 </td>
##    <td style="text-align:left;"> Fan_dif ~ Treatment + Lake_type + Nest_depth + Percent_cover + Embryos_stage_simple + (1 &amp;#124; Lake) </td>
##    <td style="text-align:right;"> 10 </td>
##    <td style="text-align:right;"> -180.967 </td>
##    <td style="text-align:right;"> 390.082 </td>
##    <td style="text-align:right;"> 10.364 </td>
##    <td style="text-align:right;"> 3.511261e-03 </td>
##   </tr>
##   <tr>
##    <td style="text-align:left;"> model10 </td>
##    <td style="text-align:left;"> Fan_dif ~ Treatment + Lake_type + Bodycondition + Nest_depth + Percent_cover + Embryos_stage_simple + (1 &amp;#124; Lake) </td>
##    <td style="text-align:right;"> 11 </td>
##    <td style="text-align:right;"> -179.709 </td>
##    <td style="text-align:right;"> 391.572 </td>
##    <td style="text-align:right;"> 11.855 </td>
##    <td style="text-align:right;"> 1.666265e-03 </td>
##   </tr>
##   <tr>
##    <td style="text-align:left;"> model7 </td>
##    <td style="text-align:left;"> Fan_dif ~ Treatment * Lake_type + Nest_depth + Percent_cover + Embryos_stage_simple + (1 &amp;#124; Lake) </td>
##    <td style="text-align:right;"> 12 </td>
##    <td style="text-align:right;"> -178.384 </td>
##    <td style="text-align:right;"> 393.249 </td>
##    <td style="text-align:right;"> 13.531 </td>
##    <td style="text-align:right;"> 7.206480e-04 </td>
##   </tr>
##   <tr>
##    <td style="text-align:left;"> model9 </td>
##    <td style="text-align:left;"> Fan_dif ~ Treatment + Lake_type + Nest_depth + Percent_cover + Latency_to_return + Embryos_stage_simple + (1 &amp;#124; Lake) </td>
##    <td style="text-align:right;"> 11 </td>
##    <td style="text-align:right;"> -180.710 </td>
##    <td style="text-align:right;"> 393.573 </td>
##    <td style="text-align:right;"> 13.855 </td>
##    <td style="text-align:right;"> 6.128150e-04 </td>
##   </tr>
##   <tr>
##    <td style="text-align:left;"> model8 </td>
##    <td style="text-align:left;"> Fan_dif ~ Treatment + Lake_type + Bodycondition + Nest_depth + Percent_cover + Latency_to_return + Embryos_stage_simple + (1 &amp;#124; Lake) </td>
##    <td style="text-align:right;"> 12 </td>
##    <td style="text-align:right;"> -179.281 </td>
##    <td style="text-align:right;"> 395.042 </td>
##    <td style="text-align:right;"> 15.324 </td>
##    <td style="text-align:right;"> 2.940083e-04 </td>
##   </tr>
##   <tr>
##    <td style="text-align:left;"> model5 </td>
##    <td style="text-align:left;"> Fan_dif ~ Treatment * Lake_type + Bodycondition + Nest_depth + Percent_cover + Embryos_stage_simple + (1 &amp;#124; Lake) </td>
##    <td style="text-align:right;"> 13 </td>
##    <td style="text-align:right;"> -177.583 </td>
##    <td style="text-align:right;"> 396.333 </td>
##    <td style="text-align:right;"> 16.616 </td>
##    <td style="text-align:right;"> 1.541309e-04 </td>
##   </tr>
##   <tr>
##    <td style="text-align:left;"> model4 </td>
##    <td style="text-align:left;"> Fan_dif ~ Treatment * Lake_type + Bodycondition + Nest_depth + Latency_to_return + Embryos_stage_simple + (1 &amp;#124; Lake) </td>
##    <td style="text-align:right;"> 13 </td>
##    <td style="text-align:right;"> -178.225 </td>
##    <td style="text-align:right;"> 397.616 </td>
##    <td style="text-align:right;"> 17.898 </td>
##    <td style="text-align:right;"> 8.117249e-05 </td>
##   </tr>
##   <tr>
##    <td style="text-align:left;"> model2 </td>
##    <td style="text-align:left;"> Fan_dif ~ Treatment * Lake_type + Nest_depth + Percent_cover + Latency_to_return + Embryos_stage_simple + (1 &amp;#124; Lake) </td>
##    <td style="text-align:right;"> 13 </td>
##    <td style="text-align:right;"> -178.303 </td>
##    <td style="text-align:right;"> 397.772 </td>
##    <td style="text-align:right;"> 18.055 </td>
##    <td style="text-align:right;"> 7.506052e-05 </td>
##   </tr>
##   <tr>
##    <td style="text-align:left;"> model3 </td>
##    <td style="text-align:left;"> Fan_dif ~ Treatment * Lake_type + Bodycondition + Percent_cover + Latency_to_return + Embryos_stage_simple + (1 &amp;#124; Lake) </td>
##    <td style="text-align:right;"> 13 </td>
##    <td style="text-align:right;"> -178.518 </td>
##    <td style="text-align:right;"> 398.203 </td>
##    <td style="text-align:right;"> 18.485 </td>
##    <td style="text-align:right;"> 6.053165e-05 </td>
##   </tr>
##   <tr>
##    <td style="text-align:left;"> Bigmodel </td>
##    <td style="text-align:left;"> Fan_dif ~ Treatment * Lake_type + Bodycondition + Nest_depth + Percent_cover + Latency_to_return + Embryos_stage_simple + (1 &amp;#124; Lake) </td>
##    <td style="text-align:right;"> 14 </td>
##    <td style="text-align:right;"> -177.401 </td>
##    <td style="text-align:right;"> 401.062 </td>
##    <td style="text-align:right;"> 21.345 </td>
##    <td style="text-align:right;"> 1.448779e-05 </td>
##   </tr>
## </tbody>
## </table>
```

### Evaluating final selected model

```
# final selected model from AICc
# refit winning model with REML = TRUE for final estimates

BestLMER_model <-  lmer(
  Fan_dif ~ Treatment + Lake_type + (1 | Lake),
  data = aicc_data, REML = TRUE
)
```

```
## boundary (singular) fit: see help('isSingular')
```

```
std_model <- standardize_parameters(
  BestLMER_model,
  method = "refit",
  ci = 0.95
)
```

```
## boundary (singular) fit: see help('isSingular')
```

```
# extract statistical outliers using IQR rule
outliers_fan <- boxplot.stats(aicc_data$Fan_dif)$out

# print outlier values
print(outliers_fan)
```

```
## [1] -78.637
```

```
# show rows corresponding to outliers
aicc_data[aicc_data$Fan_dif %in% outliers_fan, ]
```

```
## # A tibble: 1 × 15
##   ID    Baseline Predator Lake_type Ecotype  Treatment Lake        Embryos_stage
##   <chr>    <dbl>    <dbl> <fct>     <fct>    <fct>     <fct>       <chr>        
## 1 SR-22     103.     23.9 Pike      Limnetic Trout     South Rolly No eyes      
## # ℹ 7 more variables: Bodycondition <dbl>, Nest_depth <dbl>,
## #   Percent_cover <dbl>, Latency_to_return <dbl>, Fan_dif <dbl>,
## #   Embryos_stage_simple <fct>, Lake_type_label <fct>
```

```
# number of outliers
length(outliers_fan)
```

```
## [1] 1
```

```
# Make the exact model dataset
fan_model_data <- aicc_data %>%
  filter(
    !is.na(Fan_dif),
    !is.na(Treatment),
    !is.na(Lake_type),
    !is.na(Lake)
  ) %>%
  mutate(row_id = row_number())

# Fit full best model
bestLMER_model <- lmer(
  Fan_dif ~ Treatment + Lake_type + (1 | Lake),
  data = fan_model_data,
  REML = TRUE
)
```

```
## boundary (singular) fit: see help('isSingular')
```

```
summary(bestLMER_model)
```

```
## Linear mixed model fit by REML. t-tests use Satterthwaite's method [
## lmerModLmerTest]
## Formula: Fan_dif ~ Treatment + Lake_type + (1 | Lake)
##    Data: fan_model_data
## 
## REML criterion at convergence: 342.2
## 
## Scaled residuals: 
##     Min      1Q  Median      3Q     Max 
## -2.4971 -0.5129 -0.0176  0.5299  2.2318 
## 
## Random effects:
##  Groups   Name        Variance Std.Dev.
##  Lake     (Intercept)    0      0.00   
##  Residual             1030     32.09   
## Number of obs: 38, groups:  Lake, 6
## 
## Fixed effects:
##                Estimate Std. Error      df t value Pr(>|t|)
## (Intercept)      -4.652     11.186  34.000  -0.416    0.680
## TreatmentPike    21.057     12.914  34.000   1.631    0.112
## TreatmentTrout   21.120     12.901  34.000   1.637    0.111
## Lake_typePike   -14.967     10.747  34.000  -1.393    0.173
## 
## Correlation of Fixed Effects:
##             (Intr) TrtmnP TrtmnT
## TreatmentPk -0.651              
## TreatmntTrt -0.544  0.506       
## Lake_typePk -0.560  0.101 -0.091
## optimizer (nloptwrap) convergence code: 0 (OK)
## boundary (singular) fit: see help('isSingular')
```

```
# Identify the most extreme observations by absolute Fan_dif
extreme_fan <- fan_model_data %>%
  mutate(abs_Fan_dif = abs(Fan_dif)) %>%
  arrange(desc(abs_Fan_dif)) %>%
  select(row_id, Lake, Lake_type, Treatment, Fan_dif, abs_Fan_dif) %>%
  slice(1:5)

print(extreme_fan)
```

```
## # A tibble: 5 × 6
##   row_id Lake        Lake_type Treatment Fan_dif abs_Fan_dif
##    <int> <fct>       <fct>     <fct>       <dbl>       <dbl>
## 1     38 South Rolly Pike      Trout       -78.6        78.6
## 2     23 Finger      Pike      Trout        73.1        73.1
## 3     21 Finger      Pike      Char        -64.5        64.5
## 4     16 Hope        No Pike   Pike         60.1        60.1
## 5     29 Crystal     Pike      Trout       -58.9        58.9
```

```
# Remove the single most extreme observation and refit
fan_no_top1 <- fan_model_data %>%
  filter(row_id != extreme_fan$row_id[1])

bestLMER_no_top1 <- lmer(
  Fan_dif ~ Treatment + Lake_type + (1 | Lake),
  data = fan_no_top1,
  REML = TRUE
)
```

```
## boundary (singular) fit: see help('isSingular')
```

```
summary(bestLMER_no_top1)
```

```
## Linear mixed model fit by REML. t-tests use Satterthwaite's method [
## lmerModLmerTest]
## Formula: Fan_dif ~ Treatment + Lake_type + (1 | Lake)
##    Data: fan_no_top1
## 
## REML criterion at convergence: 325.9
## 
## Scaled residuals: 
##     Min      1Q  Median      3Q     Max 
## -2.3376 -0.5703 -0.1540  0.5041  2.1957 
## 
## Random effects:
##  Groups   Name        Variance Std.Dev.
##  Lake     (Intercept)   0.0     0.00   
##  Residual             847.9    29.12   
## Number of obs: 37, groups:  Lake, 6
## 
## Fixed effects:
##                Estimate Std. Error      df t value Pr(>|t|)  
## (Intercept)      -6.420     10.168  33.000  -0.631   0.5321  
## TreatmentPike    21.426     11.718  33.000   1.829   0.0765 .
## TreatmentTrout   27.546     11.915  33.000   2.312   0.0272 *
## Lake_typePike   -11.936      9.808  33.000  -1.217   0.2322  
## ---
## Signif. codes:  0 '***' 0.001 '**' 0.01 '*' 0.05 '.' 0.1 ' ' 1
## 
## Correlation of Fixed Effects:
##             (Intr) TrtmnP TrtmnT
## TreatmentPk -0.650              
## TreatmntTrt -0.545  0.499       
## Lake_typePk -0.563  0.102 -0.069
## optimizer (nloptwrap) convergence code: 0 (OK)
## boundary (singular) fit: see help('isSingular')
```

```
#  Remove the top 2 most extreme observations and refit
fan_no_top2 <- fan_model_data %>%
  filter(!row_id %in% extreme_fan$row_id[1:2])

bestLMER_no_top2 <- lmer(
  Fan_dif ~ Treatment + Lake_type + (1 | Lake),
  data = fan_no_top2,
  REML = TRUE
)
```

```
## boundary (singular) fit: see help('isSingular')
```

```
summary(bestLMER_no_top2)
```

```
## Linear mixed model fit by REML. t-tests use Satterthwaite's method [
## lmerModLmerTest]
## Formula: Fan_dif ~ Treatment + Lake_type + (1 | Lake)
##    Data: fan_no_top2
## 
## REML criterion at convergence: 311.5
## 
## Scaled residuals: 
##      Min       1Q   Median       3Q      Max 
## -2.26341 -0.60342 -0.04109  0.59636  1.77515 
## 
## Random effects:
##  Groups   Name        Variance Std.Dev.
##  Lake     (Intercept)   0.0     0.00   
##  Residual             733.1    27.08   
## Number of obs: 36, groups:  Lake, 6
## 
## Fixed effects:
##                Estimate Std. Error      df t value Pr(>|t|)  
## (Intercept)      -4.860      9.475  32.000  -0.513   0.6116  
## TreatmentPike    21.100     10.896  32.000   1.936   0.0617 .
## TreatmentTrout   21.875     11.312  32.000   1.934   0.0620 .
## Lake_typePike   -14.611      9.183  32.000  -1.591   0.1214  
## ---
## Signif. codes:  0 '***' 0.001 '**' 0.01 '*' 0.05 '.' 0.1 ' ' 1
## 
## Correlation of Fixed Effects:
##             (Intr) TrtmnP TrtmnT
## TreatmentPk -0.650              
## TreatmntTrt -0.546  0.491       
## Lake_typePk -0.565  0.103 -0.043
## optimizer (nloptwrap) convergence code: 0 (OK)
## boundary (singular) fit: see help('isSingular')
```

```
# Compare fixed effects side by side
coef_full <- coef(summary(bestLMER_model)) %>%
  as.data.frame() %>%
  mutate(term = rownames(.), model = "Full data")

coef_no1 <- coef(summary(bestLMER_no_top1)) %>%
  as.data.frame() %>%
  mutate(term = rownames(.), model = "Top 1 removed")

coef_no2 <- coef(summary(bestLMER_no_top2)) %>%
  as.data.frame() %>%
  mutate(term = rownames(.), model = "Top 2 removed")

coef_compare <- bind_rows(coef_full, coef_no1, coef_no2) %>%
  select(model, term, Estimate, `Std. Error`, df, `t value`, `Pr(>|t|)`)

print(coef_compare)
```

```
##                             model           term   Estimate Std. Error df
## (Intercept)...1         Full data    (Intercept)  -4.651812  11.186198 34
## TreatmentPike...2       Full data  TreatmentPike  21.056917  12.913768 34
## TreatmentTrout...3      Full data TreatmentTrout  21.119892  12.900530 34
## Lake_typePike...4       Full data  Lake_typePike -14.966894  10.747351 34
## (Intercept)...5     Top 1 removed    (Intercept)  -6.419709  10.167928 33
## TreatmentPike...6   Top 1 removed  TreatmentPike  21.426038  11.717547 33
## TreatmentTrout...7  Top 1 removed TreatmentTrout  27.545518  11.915408 33
## Lake_typePike...8   Top 1 removed  Lake_typePike -11.936214   9.807795 33
## (Intercept)...9     Top 2 removed    (Intercept)  -4.859601   9.475388 32
## TreatmentPike...10  Top 2 removed  TreatmentPike  21.100301  10.896229 32
## TreatmentTrout...11 Top 2 removed TreatmentTrout  21.875127  11.312230 32
## Lake_typePike...12  Top 2 removed  Lake_typePike -14.610684   9.183038 32
##                        t value   Pr(>|t|)
## (Intercept)...1     -0.4158528 0.68013088
## TreatmentPike...2    1.6305788 0.11220975
## TreatmentTrout...3   1.6371337 0.11082663
## Lake_typePike...4   -1.3926124 0.17277790
## (Intercept)...5     -0.6313684 0.53214898
## TreatmentPike...6    1.8285430 0.07651922
## TreatmentTrout...7   2.3117562 0.02717529
## Lake_typePike...8   -1.2170130 0.23223152
## (Intercept)...9     -0.5128656 0.61156779
## TreatmentPike...10   1.9364775 0.06168263
## TreatmentTrout...11  1.9337590 0.06202925
## Lake_typePike...12  -1.5910512 0.12143010
```

```
bind_rows(
  tidy(bestLMER_model) %>% mutate(Model="Full"),
  tidy(bestLMER_no_top1) %>% mutate(Model="Top1 removed"),
  tidy(bestLMER_no_top2) %>% mutate(Model="Top2 removed")
) %>%
filter(term!="(Intercept)") %>%
select(Model, term, estimate, std.error, p.value)
```

```
## # A tibble: 15 × 5
##    Model        term            estimate std.error p.value
##    <chr>        <chr>              <dbl>     <dbl>   <dbl>
##  1 Full         TreatmentPike       21.1     12.9   0.112 
##  2 Full         TreatmentTrout      21.1     12.9   0.111 
##  3 Full         Lake_typePike      -15.0     10.7   0.173 
##  4 Full         sd__(Intercept)      0       NA    NA     
##  5 Full         sd__Observation     32.1     NA    NA     
##  6 Top1 removed TreatmentPike       21.4     11.7   0.0765
##  7 Top1 removed TreatmentTrout      27.5     11.9   0.0272
##  8 Top1 removed Lake_typePike      -11.9      9.81  0.232 
##  9 Top1 removed sd__(Intercept)      0       NA    NA     
## 10 Top1 removed sd__Observation     29.1     NA    NA     
## 11 Top2 removed TreatmentPike       21.1     10.9   0.0617
## 12 Top2 removed TreatmentTrout      21.9     11.3   0.0620
## 13 Top2 removed Lake_typePike      -14.6      9.18  0.121 
## 14 Top2 removed sd__(Intercept)      0       NA    NA     
## 15 Top2 removed sd__Observation     27.1     NA    NA
```

```
# diagnostics/ check residuals 
best_resid <- resid(BestLMER_model)
best_fitted <- fitted(BestLMER_model)

hist(
  best_resid,
  breaks = 15,
  main = "Histogram of residuals: best behavioral model",
  xlab = "Residuals",
  col = "grey80",
  border = "white"
)
```

```
qqnorm(best_resid, main = "QQ plot of residuals: best behavioral model")
qqline(best_resid, col = "red", lwd = 2)
```

```
plot(
  best_fitted, best_resid,
  xlab = "Fitted values",
  ylab = "Residuals",
  main = "Residuals vs fitted: best behavioral model",
  pch = 16,
  col = "black"
)

abline(h = 0, lty = 2, col = "red")
```

```
print(shapiro.test(best_resid))
```

```
## 
##  Shapiro-Wilk normality test
## 
## data:  best_resid
## W = 0.98184, p-value = 0.7819
```

```
##Let's look at the model now 
print(summary(BestLMER_model))
```

```
## Linear mixed model fit by REML. t-tests use Satterthwaite's method [
## lmerModLmerTest]
## Formula: Fan_dif ~ Treatment + Lake_type + (1 | Lake)
##    Data: aicc_data
## 
## REML criterion at convergence: 342.2
## 
## Scaled residuals: 
##     Min      1Q  Median      3Q     Max 
## -2.4971 -0.5129 -0.0176  0.5299  2.2318 
## 
## Random effects:
##  Groups   Name        Variance Std.Dev.
##  Lake     (Intercept)    0      0.00   
##  Residual             1030     32.09   
## Number of obs: 38, groups:  Lake, 6
## 
## Fixed effects:
##                Estimate Std. Error      df t value Pr(>|t|)
## (Intercept)      -4.652     11.186  34.000  -0.416    0.680
## TreatmentPike    21.057     12.914  34.000   1.631    0.112
## TreatmentTrout   21.120     12.901  34.000   1.637    0.111
## Lake_typePike   -14.967     10.747  34.000  -1.393    0.173
## 
## Correlation of Fixed Effects:
##             (Intr) TrtmnP TrtmnT
## TreatmentPk -0.651              
## TreatmntTrt -0.544  0.506       
## Lake_typePk -0.560  0.101 -0.091
## optimizer (nloptwrap) convergence code: 0 (OK)
## boundary (singular) fit: see help('isSingular')
```

```
print(performance::check_collinearity(BestLMER_model))
```

```
## # Check for Multicollinearity
## 
## Low Correlation
## 
##       Term  VIF    VIF 95% CI adj. VIF Tolerance Tolerance 95% CI
##  Treatment 1.04 [1.00, 62.99]     1.02      0.96     [0.02, 1.00]
##  Lake_type 1.04 [1.00, 62.99]     1.02      0.96     [0.02, 1.00]
```

```
print(MuMIn::r.squaredGLMM(BestLMER_model))
```

```
##            R2m       R2c
## [1,] 0.1312427 0.1312427
```

```
print(confint(BestLMER_model, method = "profile"))
```

```
## Computing profile confidence intervals ...
```

```
##                     2.5 %    97.5 %
## .sig01           0.000000 13.248201
## .sigma          24.627044 38.706964
## (Intercept)    -25.926398 16.622744
## TreatmentPike   -3.502426 45.616259
## TreatmentTrout  -3.414274 45.654059
## Lake_typePike  -35.393193  5.480291
```

```
# fixed effects table
behavior_fixed_effects <- broom.mixed::tidy(
  BestLMER_model,
  effects = "fixed",
  conf.int = TRUE
)

print(behavior_fixed_effects)
```

```
## # A tibble: 4 × 9
##   effect term      estimate std.error statistic    df p.value conf.low conf.high
##   <chr>  <chr>        <dbl>     <dbl>     <dbl> <dbl>   <dbl>    <dbl>     <dbl>
## 1 fixed  (Interce…    -4.65      11.2    -0.416  34.0   0.680   -27.4      18.1 
## 2 fixed  Treatmen…    21.1       12.9     1.63   34.0   0.112    -5.19     47.3 
## 3 fixed  Treatmen…    21.1       12.9     1.64   34.0   0.111    -5.10     47.3 
## 4 fixed  Lake_typ…   -15.0       10.7    -1.39   34.0   0.173   -36.8       6.87
```

```
#standardrized effects for the best behavior model
behavior_std_effects <- effectsize::standardize_parameters(BestLMER_model)
```

```
## boundary (singular) fit: see help('isSingular')
```

```
print(behavior_std_effects)
```

```
## # Standardization method: refit
## 
## Parameter         | Std. Coef. |        95% CI
## ----------------------------------------------
## (Intercept)       |      -0.17 | [-0.86, 0.51]
## Treatment [Pike]  |       0.63 | [-0.16, 1.43]
## Treatment [Trout] |       0.64 | [-0.16, 1.43]
## Lake type [Pike]  |      -0.45 | [-1.11, 0.21]
```

```
MuMIn::r.squaredGLMM(BestLMER_model)
```

```
##            R2m       R2c
## [1,] 0.1312427 0.1312427
```

```
behavior_results_table <- behavior_fixed_effects %>%
  dplyr::mutate(
    term = dplyr::recode(
      term,
      `(Intercept)` = "Intercept",
      `TreatmentPike` = "Treatment: Pike vs Char",
      `TreatmentTrout` = "Treatment: Trout vs Char",
      `Lake_typePike` = "Lake type: Native + Invasive vs Native only",
  
    )
  ) %>%
  dplyr::select(term, estimate, std.error, statistic, df, p.value, conf.low, conf.high) %>%
  dplyr::rename(
    Term = term,
    Estimate = estimate,
    SE = std.error,
    t_value = statistic,
    DF = df,
    P_value = p.value,
    CI_low = conf.low,
    CI_high = conf.high
  )

print(
  knitr::kable(
    behavior_results_table,
    digits = 3,
    caption = "Fixed effects from the selected behavioral mixed-effects model"
  ) %>%
    kableExtra::kable_styling(full_width = FALSE)
)
```

```
## <table class="table" style="color: black; width: auto !important; margin-left: auto; margin-right: auto;">
## <caption>Fixed effects from the selected behavioral mixed-effects model</caption>
##  <thead>
##   <tr>
##    <th style="text-align:left;"> Term </th>
##    <th style="text-align:right;"> Estimate </th>
##    <th style="text-align:right;"> SE </th>
##    <th style="text-align:right;"> t_value </th>
##    <th style="text-align:right;"> DF </th>
##    <th style="text-align:right;"> P_value </th>
##    <th style="text-align:right;"> CI_low </th>
##    <th style="text-align:right;"> CI_high </th>
##   </tr>
##  </thead>
## <tbody>
##   <tr>
##    <td style="text-align:left;"> Intercept </td>
##    <td style="text-align:right;"> -4.652 </td>
##    <td style="text-align:right;"> 11.186 </td>
##    <td style="text-align:right;"> -0.416 </td>
##    <td style="text-align:right;"> 34 </td>
##    <td style="text-align:right;"> 0.680 </td>
##    <td style="text-align:right;"> -27.385 </td>
##    <td style="text-align:right;"> 18.081 </td>
##   </tr>
##   <tr>
##    <td style="text-align:left;"> Treatment: Pike vs Char </td>
##    <td style="text-align:right;"> 21.057 </td>
##    <td style="text-align:right;"> 12.914 </td>
##    <td style="text-align:right;"> 1.631 </td>
##    <td style="text-align:right;"> 34 </td>
##    <td style="text-align:right;"> 0.112 </td>
##    <td style="text-align:right;"> -5.187 </td>
##    <td style="text-align:right;"> 47.301 </td>
##   </tr>
##   <tr>
##    <td style="text-align:left;"> Treatment: Trout vs Char </td>
##    <td style="text-align:right;"> 21.120 </td>
##    <td style="text-align:right;"> 12.901 </td>
##    <td style="text-align:right;"> 1.637 </td>
##    <td style="text-align:right;"> 34 </td>
##    <td style="text-align:right;"> 0.111 </td>
##    <td style="text-align:right;"> -5.097 </td>
##    <td style="text-align:right;"> 47.337 </td>
##   </tr>
##   <tr>
##    <td style="text-align:left;"> Lake type: Native + Invasive vs Native only </td>
##    <td style="text-align:right;"> -14.967 </td>
##    <td style="text-align:right;"> 10.747 </td>
##    <td style="text-align:right;"> -1.393 </td>
##    <td style="text-align:right;"> 34 </td>
##    <td style="text-align:right;"> 0.173 </td>
##    <td style="text-align:right;"> -36.808 </td>
##    <td style="text-align:right;"> 6.874 </td>
##   </tr>
## </tbody>
## </table>
```

```
# let's try estimated marginal meansn for our treatments 
emmeans_treat_main <- emmeans::emmeans(BestLMER_model, ~ Treatment)
emmeans_treat_pairs <- summary(pairs(emmeans_treat_main, adjust = "tukey"))
print(emmeans_treat_pairs)
```

```
##  contrast     estimate   SE   df t.ratio p.value
##  Char - Pike   -21.057 13.0 30.8  -1.622  0.2518
##  Char - Trout  -21.120 13.1 32.2  -1.611  0.2558
##  Pike - Trout   -0.063 13.2 33.0  -0.005  1.0000
## 
## Results are averaged over the levels of: Lake_type 
## Degrees-of-freedom method: kenward-roger 
## P value adjustment: tukey method for comparing a family of 3 estimates
```

```
aicc_data %>%
  group_by(Treatment) %>%
  summarise(
    n = n(),
    mean = mean(Latency_to_return),
    sd = sd(Latency_to_return),
    p = t.test(Latency_to_return, mu = 0)$p.value,
    CI.low = t.test(Latency_to_return, mu = 0)$conf.int[1],
    CI.high = t.test(Latency_to_return, mu = 0)$conf.int[2]
  )
```

```
## # A tibble: 3 × 7
##   Treatment     n  mean    sd       p CI.low CI.high
##   <fct>     <int> <dbl> <dbl>   <dbl>  <dbl>   <dbl>
## 1 Char         12  10.6  8.94 0.00172   4.94    16.3
## 2 Pike         13  18.6 23.0  0.0127    4.76    32.5
## 3 Trout        13  13.1 13.0  0.00331   5.30    21.0
```

### Visualization

```
# behavior figures
# main boxplot
p_beh_lake_treat <- ggplot(
  aicc_data %>% dplyr::filter(!is.na(Lake_type_label), !is.na(Treatment), !is.na(Fan_dif)),
  aes(x = Lake_type_label, y = Fan_dif, fill = Treatment)
) +
  geom_boxplot(
    aes(group = interaction(Lake_type_label, Treatment)),
    outlier.shape = NA,
    width = 0.65,
    alpha = 0.85,
    position = position_dodge(width = 0.75)
  ) +
  geom_point(
    aes(shape = Treatment, group = interaction(Lake_type_label, Treatment)),
    color = "black",
    size = 2.4,
    alpha = 0.85,
    position = position_jitterdodge(
      jitter.width = 0.08,
      dodge.width = 0.75
    )
  ) +
  geom_hline(yintercept = 0, linewidth = 0.4) +
  scale_fill_manual(values = pal_treatment) +
  scale_shape_manual(values = treatment_shape_map) +
  labs(
    title = "Difference in fanning time by lake type and predator treatment",
    x = "Lake type",
    y = "Difference in fanning time(s)",
    fill = "Treatment",
    shape = "Treatment"
  ) +
  theme_paper()

print(p_beh_lake_treat)
```

```
# fixed-effect coefficient plot
coef_plot_df <- behavior_fixed_effects %>%
  dplyr::filter(term != "(Intercept)") %>%
  dplyr::mutate(
    term = dplyr::recode(
      term,
      `TreatmentPike` = "Treatment: Pike vs Char",
      `TreatmentTrout` = "Treatment: Trout vs Char",
      `Lake_typePike` = "Lake type: Native + Invasive vs Native only",
     
    )
  )

p_coef <- ggplot(
  coef_plot_df,
  aes(x = estimate, y = reorder(term, estimate))
) +
  geom_vline(xintercept = 0, linetype = "dashed", linewidth = 0.4) +
  geom_errorbarh(
    aes(xmin = conf.low, xmax = conf.high),
    height = 0.15,
    linewidth = 0.7
  ) +
  geom_point(size = 3) +
  labs(
    title = "Fixed effects from the best behavioral model",
    x = "Estimate (95% CI)",
    y = NULL
  ) +
  theme_paper()
```

```
## Warning: `geom_errorbarh()` was deprecated in ggplot2 4.0.0.
## ℹ Please use the `orientation` argument of `geom_errorbar()` instead.
## This warning is displayed once per session.
## Call `lifecycle::last_lifecycle_warnings()` to see where this warning was
## generated.
```

```
print(p_coef)
```

```
## `height` was translated to `width`.
```

```
# predicted marginal means plot
emm_plot_df <- as.data.frame(
  emmeans::emmeans(BestLMER_model, ~ Treatment + Lake_type)
) %>%
  dplyr::mutate(
    Lake_type_label = dplyr::case_when(
      Lake_type == "No Pike" ~ "Native predator only lakes",
      Lake_type == "Pike" ~ "Native + Invasive predator lakes",
      TRUE ~ as.character(Lake_type)
    )
  )

p_emm <- ggplot(
  emm_plot_df,
  aes(x = Treatment, y = emmean, color = Treatment, group = 1)
) +
  geom_point(size = 3) +
  geom_line(linewidth = 0.8) +
  geom_errorbar(
    aes(ymin = lower.CL, ymax = upper.CL),
    width = 0.12,
    linewidth = 0.8
  ) +
  facet_wrap(~ Lake_type_label) +
  scale_color_manual(values = pal_treatment) +
  labs(
    title = "Estimated marginal means of predator-induced change in fanning time",
    x = "Treatment",
    y = "Estimated mean change in fanning time (s)",
    color = "Treatment"
  ) +
  theme_paper()

print(p_emm)
```

```
# reaction norm: raw means
raw_means_df <- aicc_data %>%
  dplyr::group_by(Lake_type_label, Treatment) %>%
  dplyr::summarise(
    mean_Fan_dif = mean(Fan_dif, na.rm = TRUE),
    sd_Fan_dif = sd(Fan_dif, na.rm = TRUE),
    n = dplyr::n(),
    se_Fan_dif = sd_Fan_dif / sqrt(n),
    .groups = "drop"
  )

p_reaction_raw <- ggplot(
  raw_means_df,
  aes(x = Treatment, y = mean_Fan_dif, color = Lake_type_label, group = Lake_type_label)
) +
  geom_point(size = 3) +
  geom_line(linewidth = 0.9) +
  geom_errorbar(
    aes(ymin = mean_Fan_dif - se_Fan_dif, ymax = mean_Fan_dif + se_Fan_dif),
    width = 0.12,
    linewidth = 0.7
  ) +
  scale_color_manual(values = pal_lake) +
  labs(
    title = "Reaction norm plot of raw mean change in fanning time",
    x = "Treatment",
    y = "Mean change in fanning time (s)",
    color = "Lake type"
  ) +
  theme_paper()

print(p_reaction_raw)
```

```
# reaction norm: model means
p_reaction_emm <- ggplot(
  emm_plot_df,
  aes(x = Treatment, y = emmean, color = Lake_type_label, group = Lake_type_label)
) +
  geom_point(size = 3) +
  geom_line(linewidth = 0.9) +
  geom_errorbar(
    aes(ymin = lower.CL, ymax = upper.CL),
    width = 0.12,
    linewidth = 0.7
  ) +
  scale_color_manual(values = pal_lake) +
  labs(
    title = "Reaction norm plot of model-estimated mean change in fanning time",
    x = "Treatment",
    y = "Estimated mean change in fanning time (s)",
    color = "Lake type"
  ) +
  theme_paper()

print(p_reaction_emm)
```

```
plot_data <- aicc_data %>%
  filter(!is.na(Lake_type_label),
         !is.na(Treatment),
         !is.na(Fan_dif))

summary_fan <- plot_data %>%
  group_by(Lake_type_label, Treatment) %>%
  summarise(
    n = n(),
    mean = mean(Fan_dif, na.rm = TRUE),
    se = sd(Fan_dif, na.rm = TRUE) / sqrt(n),
    ci = qt(0.975, df = n - 1) * se,
    .groups = "drop"
  )

p_fan_lake_treat <- ggplot(
  plot_data,
  aes(x = Lake_type_label,
      y = Fan_dif,
      fill = Treatment,
      color = Treatment)
) +
  geom_hline(yintercept = 0, linetype = "dashed",
             color = "grey55", linewidth = 0.5) +

  # boxplot
  geom_crossbar(
    data = plot_data %>%
      group_by(Lake_type_label, Treatment) %>%
      summarise(
        q1 = quantile(Fan_dif, 0.25, na.rm = TRUE),
        med = median(Fan_dif, na.rm = TRUE),
        q3 = quantile(Fan_dif, 0.75, na.rm = TRUE),
        .groups = "drop"
      ),
    aes(
      x = Lake_type_label,
      y = med,
      ymin = q1,
      ymax = q3,
      group = Treatment,
      fill = Treatment,
      color = Treatment
    ),
    position = position_dodge(width = 0.78),
    width = 0.32,
    alpha = 0.18,
    linewidth = 0.45
  ) +

  # individual males
  geom_point(
    aes(shape = Treatment),
    position = position_jitterdodge(jitter.width = 0.07,
                                    dodge.width = 0.78),
    size = 2.7,
    alpha = 0.9
  ) +

  # mean ± 95% CI
  geom_pointrange(
    data = summary_fan,
    aes(x = Lake_type_label,
        y = mean,
        ymin = mean - ci,
        ymax = mean + ci,
        group = Treatment),
    inherit.aes = FALSE,
    position = position_dodge(width = 0.78),
    shape = 18,
    size = 0.25,
    linewidth = 0.55,
    color = "black"
  ) +

  scale_fill_manual(values = pal_treatment) +
  scale_color_manual(values = pal_treatment) +
  scale_shape_manual(values = treatment_shape_map) +

  labs(
    title = "Change in fanning time by lake type and predator treatment",
    x = "Lake type",
    y = "Difference in fanning time (s)",
    fill = "Treatment",
    color = "Treatment",
    shape = "Treatment"
  ) +
  theme_paper()

print(p_fan_lake_treat)
```

```
ggsave(
  filename = "Difference_fanning_time.png",
  plot = p_fan_lake_treat, 
  width = 8,
  height = 6,
  units = "in",
  dpi = 600,
  bg = "white"
)

###return to nest data
    ggplot(aicc_data,
       aes(x = Treatment,
           y = Latency_to_return,
           color = Treatment,
           fill = Treatment,
           shape = Treatment)) +


  geom_boxplot(width = 0.18,
               alpha = 0.25,
               outlier.shape = NA,
               linewidth = 0.5) +

  geom_jitter(width = 0.08,
              size = 2.2,
              alpha = 0.85) +

  stat_summary(fun = mean,
               geom = "point",
               shape = 18,
               size = 2.5,
               color = "black") +

  geom_hline(yintercept = 0,
             linetype = "dashed") +

  labs(
    x = "Predator treatment",
    y = "Latency to return to nest (s)"
  ) +

  theme_paper()
```

```
ggplot(aicc_data, aes(x = Lake_type_label, y = Baseline, fill = Lake_type)) +
  geom_boxplot(outlier.shape = NA, alpha = 0.6, width = 0.6) +
  geom_jitter(width = 0.25, height = 0, size = 2.5, color = "black", alpha = 0.8) +
  labs(x = "Lake type", y = "Baseline fanning time (s)") +
  theme_minimal(base_size = 14) +
  theme(
    legend.position = "none",
    panel.grid.minor = element_blank(),
    panel.grid.major.x = element_blank()
  )
```

#Transcriptomic data

## Overview

Analyze whole brain gene expression between predator communities, and
between parental state (parent vs non-parent)

## Load necessary libraries and data

```
shelf(genefilter, DESeq2, apeglm, vsn, tidyr, RColorBrewer, dplyr, tidyverse, ggrepel, ggplot2, stringr, AnnotationDbi, clusterProfiler, enrichplot, forcats, GO.db, biomaRt, tibble, pheatmap)
```

```
## 
##   The 'cran_repo' argument in shelf() was not set, so it will use
##   cran_repo = 'https://cran.r-project.org' by default.
## 
##   To avoid this message, set the 'cran_repo' argument to a CRAN
##   mirror URL (see https://cran.r-project.org/mirrors.html) or set
##   'quiet = TRUE'.
```

```
#######
# Loading in raw data and meta data

akdata <- read.csv("AK24_Predator_GeneCounts.csv", check.names = FALSE)
akmeta <- read.csv("AK24_Predator_metadata.csv", check.names = FALSE, stringsAsFactors = FALSE)
```

## Checking and cleaning data as necessary

```
# Confirming that the data files are a matrix of count data.

akdata <- data.frame(akdata[, -1], row.names = akdata[, 1])

if ("sample" %in% names(akmeta)) {
  rownames(akmeta) <- akmeta$sample
} else {
  rownames(akmeta) <- akmeta[, 1]
  akmeta <- akmeta[, -1, drop = FALSE]
}

head(akdata)
```

```
##                    CL10 CL5  CL6 FG10 FG5  HL1  HL3 SP06 SP11 SP61 SP62 SR10
## ENSGACG00000026949    0   2    2    0   3    3    6    2    1    4    4    0
## ENSGACG00000001074  723 547  986 1462 808  995 1178 1172  945  882  727  865
## ENSGACG00000001071   34   6   19   24  10    7   25   76   60   43   48    9
## ENSGACG00000001068  996 804  828 1694 935  946 1562 1829 1400 1266 1192  903
## ENSGACG00000025125  300 609 1069 1398 915 1052 1608  937  758 1047  854  720
## ENSGACG00000019934  129 285  368  393 265  323  477  230  599  422  332  357
##                    SR21 SR22 SR51 SR52  WT1 WT2  WT3
## ENSGACG00000026949    0    0    4    0    1   2    1
## ENSGACG00000001074  684  843  913 1008  948 566 1120
## ENSGACG00000001071    2   12   10    9    9   2   13
## ENSGACG00000001068  619  937  758  815 1474 931 1393
## ENSGACG00000025125  876  856  954 1100 1188 502 1088
## ENSGACG00000019934  218  309  407  377  460 187  416
```

```
head(akmeta)
```

```
##         Lake Treatment Lake_type BodyCondition Embryo_stage Parent_state Batch
## CL10 Crystal     Trout      Pike      1.303807      No eyes    Parenting     3
## CL5  Crystal      Pike      Pike      0.971860        Other    Parenting     2
## CL6  Crystal      Char      Pike      1.137431      No eyes    Parenting     3
## FG10  Finger     Trout      Pike      1.389248      No eyes    Parenting     3
## FG5   Finger      Char      Pike      1.167908        Other    Parenting     3
## HL1     Hope     Trout   No pike      1.588722         Eyes    Parenting     4
```

```
# Make sure the sample names are in the same order between the two files.

all(colnames(akdata) %in% rownames(akmeta))
```

```
## [1] TRUE
```

```
akdata <- akdata[, rownames(akmeta), drop = FALSE]
all(colnames(akdata) == rownames(akmeta))
```

```
## [1] TRUE
```

```
# Cleaning metadata and setting factor levels

akmeta$Batch <- factor(akmeta$Batch)

akmeta$Lake_type <- trimws(as.character(akmeta$Lake_type))
akmeta$Lake_type[akmeta$Lake_type %in% c("No Pike", "No pike", "no pike")] <- "No Pike"
akmeta$Lake_type[akmeta$Lake_type %in% c("Pike", "pike")] <- "Pike"
akmeta$Lake_type <- factor(akmeta$Lake_type, levels = c("No Pike", "Pike"))

akmeta$Treatment <- trimws(as.character(akmeta$Treatment))
akmeta$Treatment <- factor(akmeta$Treatment, levels = c("Char", "Pike", "Trout"))

akmeta$Parent_state <- trimws(as.character(akmeta$Parent_state))
akmeta$Parent_state[akmeta$Parent_state %in% c("Parenting", "parenting")] <- "Parenting"
akmeta$Parent_state[akmeta$Parent_state %in% c("Non-parenting", "Non parenting", "non-parenting", "non parenting")] <- "Non-parenting"
akmeta$Parent_state <- factor(akmeta$Parent_state, levels = c("Non-parenting", "Parenting"))

akmeta$Embryo_stage <- trimws(as.character(akmeta$Embryo_stage))
akmeta$Embryo_stage[akmeta$Embryo_stage == ""] <- NA
akmeta$Embryo_stage <- factor(akmeta$Embryo_stage)

akmeta$Treatment <- relevel(akmeta$Treatment, ref = "Char")
akmeta$Lake_type <- relevel(akmeta$Lake_type, ref = "No Pike")
akmeta$Parent_state <- relevel(akmeta$Parent_state, ref = "Non-parenting")

akmeta$Lake_type_label <- ifelse(
  akmeta$Lake_type == "No Pike",
  "Native predator-only",
  "Native + invasive predator"
)
akmeta$Lake_type_label <- factor(
  akmeta$Lake_type_label,
  levels = c("Native predator-only", "Native + invasive predator")
)

# Plotting histogram and mean-variance relationship

row_mean1 <- apply(akdata, 1, mean)
row_var1 <- apply(akdata, 1, var)
df <- data.frame(row_mean1, row_var1)

hist(log(df$row_mean1 + 1))
```

```
plot(log(df$row_mean1 + 1), log(df$row_var1 + 1), abline(0,1), ylim = c(0,30), xlim = c(0,30))
```

## Filtering genes and running analyses

```
# Creating dds object with batch, lake type, and parent state in the model

dds <- DESeqDataSetFromMatrix(countData = akdata, colData = akmeta, design = ~ Batch + Lake_type + Parent_state)
```

```
##   Note: levels of factors in the design contain characters other than
##   letters, numbers, '_' and '.'. It is recommended (but not required) to use
##   only letters, numbers, and delimiters '_' or '.', as these are safe characters
##   for column names in R. [This is a message, not a warning or an error]
```

```
# Pre-filtering out genes with less than 10 counts in fewer than 3 samples

keep <- rowSums(counts(dds) >= 10) >= 3
dds <- dds[keep, ]
names(dds)
```

```
##     [1] "ENSGACG00000001074" "ENSGACG00000001071" "ENSGACG00000001068"
##     [4] "ENSGACG00000025125" "ENSGACG00000019934" "ENSGACG00000019936"
##     [7] "ENSGACG00000019938" "ENSGACG00000019945" "ENSGACG00000034280"
##    [10] "ENSGACG00000019990" "ENSGACG00000028744" "ENSGACG00000019993"
##    [13] "ENSGACG00000023454" "ENSGACG00000001122" "ENSGACG00000001116"
##    [16] "ENSGACG00000001102" "ENSGACG00000019869" "ENSGACG00000019879"
##    [19] "ENSGACG00000024620" "ENSGACG00000019892" "ENSGACG00000019897"
##    [22] "ENSGACG00000019903" "ENSGACG00000019904" "ENSGACG00000019949"
##    [25] "ENSGACG00000019956" "ENSGACG00000029540" "ENSGACG00000019957"
##    [28] "ENSGACG00000019962" "ENSGACG00000019967" "ENSGACG00000019970"
##    [31] "ENSGACG00000019973" "ENSGACG00000019975" "ENSGACG00000025650"
##    [34] "ENSGACG00000019979" "ENSGACG00000019981" "ENSGACG00000019984"
##    [37] "ENSGACG00000030236" "ENSGACG00000001099" "ENSGACG00000034539"
##    [40] "ENSGACG00000001095" "ENSGACG00000001088" "ENSGACG00000001086"
##    [43] "ENSGACG00000001080" "ENSGACG00000019995" "ENSGACG00000019999"
##    [46] "ENSGACG00000020007" "ENSGACG00000037548" "ENSGACG00000019907"
##    [49] "ENSGACG00000026801" "ENSGACG00000019912" "ENSGACG00000019914"
##    [52] "ENSGACG00000019929" "ENSGACG00000027604" "ENSGACG00000020012"
##    [55] "ENSGACG00000035558" "ENSGACG00000020017" "ENSGACG00000020019"
##    [58] "ENSGACG00000020026" "ENSGACG00000020031" "ENSGACG00000020036"
##    [61] "ENSGACG00000020037" "ENSGACG00000020040" "ENSGACG00000035494"
##    [64] "ENSGACG00000020042" "ENSGACG00000027075" "ENSGACG00000020045"
##    [67] "ENSGACG00000020046" "ENSGACG00000020047" "ENSGACG00000036876"
##    [70] "ENSGACG00000028313" "ENSGACG00000020049" "ENSGACG00000020050"
##    [73] "ENSGACG00000020051" "ENSGACG00000020052" "ENSGACG00000020053"
##    [76] "ENSGACG00000020054" "ENSGACG00000032657" "ENSGACG00000020056"
##    [79] "ENSGACG00000031842" "ENSGACG00000020059" "ENSGACG00000020060"
##    [82] "ENSGACG00000020065" "ENSGACG00000020067" "ENSGACG00000020090"
##    [85] "ENSGACG00000020091" "ENSGACG00000020092" "ENSGACG00000020094"
##    [88] "ENSGACG00000020098" "ENSGACG00000020100" "ENSGACG00000020102"
##    [91] "ENSGACG00000020104" "ENSGACG00000020107" "ENSGACG00000020109"
##    [94] "ENSGACG00000020068" "ENSGACG00000020070" "ENSGACG00000020073"
##    [97] "ENSGACG00000020074" "ENSGACG00000023123" "ENSGACG00000020076"
##   [100] "ENSGACG00000020079" "ENSGACG00000020080" "ENSGACG00000020081"
##   [103] "ENSGACG00000020083" "ENSGACG00000020085" "ENSGACG00000020087"
##   [106] "ENSGACG00000028866" "ENSGACG00000019776" "ENSGACG00000019777"
##   [109] "ENSGACG00000019783" "ENSGACG00000019785" "ENSGACG00000019787"
##   [112] "ENSGACG00000019793" "ENSGACG00000029172" "ENSGACG00000019796"
##   [115] "ENSGACG00000023345" "ENSGACG00000019797" "ENSGACG00000019801"
##   [118] "ENSGACG00000019804" "ENSGACG00000019805" "ENSGACG00000024947"
##   [121] "ENSGACG00000019806" "ENSGACG00000019814" "ENSGACG00000019816"
##   [124] "ENSGACG00000019817" "ENSGACG00000034714" "ENSGACG00000019823"
##   [127] "ENSGACG00000019825" "ENSGACG00000019827" "ENSGACG00000019837"
##   [130] "ENSGACG00000019838" "ENSGACG00000019839" "ENSGACG00000019843"
##   [133] "ENSGACG00000019845" "ENSGACG00000019849" "ENSGACG00000019851"
##   [136] "ENSGACG00000019853" "ENSGACG00000019855" "ENSGACG00000019857"
##   [139] "ENSGACG00000019858" "ENSGACG00000019859" "ENSGACG00000019860"
##   [142] "ENSGACG00000019862" "ENSGACG00000019863" "ENSGACG00000031649"
##   [145] "ENSGACG00000032738" "ENSGACG00000019866" "ENSGACG00000000738"
##   [148] "ENSGACG00000000737" "ENSGACG00000000734" "ENSGACG00000000732"
##   [151] "ENSGACG00000000725" "ENSGACG00000000724" "ENSGACG00000000722"
##   [154] "ENSGACG00000000720" "ENSGACG00000000717" "ENSGACG00000000712"
##   [157] "ENSGACG00000000708" "ENSGACG00000034765" "ENSGACG00000000706"
##   [160] "ENSGACG00000028158" "ENSGACG00000028384" "ENSGACG00000016731"
##   [163] "ENSGACG00000000691" "ENSGACG00000031592" "ENSGACG00000018300"
##   [166] "ENSGACG00000000716" "ENSGACG00000000699" "ENSGACG00000027237"
##   [169] "ENSGACG00000000695" "ENSGACG00000000689" "ENSGACG00000032891"
##   [172] "ENSGACG00000000686" "ENSGACG00000016720" "ENSGACG00000016472"
##   [175] "ENSGACG00000024419" "ENSGACG00000016474" "ENSGACG00000000683"
##   [178] "ENSGACG00000000675" "ENSGACG00000000673" "ENSGACG00000000662"
##   [181] "ENSGACG00000032529" "ENSGACG00000016479" "ENSGACG00000025298"
##   [184] "ENSGACG00000000660" "ENSGACG00000036115" "ENSGACG00000017728"
##   [187] "ENSGACG00000000658" "ENSGACG00000000655" "ENSGACG00000016501"
##   [190] "ENSGACG00000016488" "ENSGACG00000000654" "ENSGACG00000016495"
##   [193] "ENSGACG00000018311" "ENSGACG00000016496" "ENSGACG00000016498"
##   [196] "ENSGACG00000018312" "ENSGACG00000000652" "ENSGACG00000016513"
##   [199] "ENSGACG00000026453" "ENSGACG00000030812" "ENSGACG00000000650"
##   [202] "ENSGACG00000016524" "ENSGACG00000035998" "ENSGACG00000031979"
##   [205] "ENSGACG00000000642" "ENSGACG00000018316" "ENSGACG00000018318"
##   [208] "ENSGACG00000018319" "ENSGACG00000018320" "ENSGACG00000030999"
##   [211] "ENSGACG00000034565" "ENSGACG00000016536" "ENSGACG00000023004"
##   [214] "ENSGACG00000030446" "ENSGACG00000000623" "ENSGACG00000016544"
##   [217] "ENSGACG00000016550" "ENSGACG00000016556" "ENSGACG00000016558"
##   [220] "ENSGACG00000018322" "ENSGACG00000018324" "ENSGACG00000018325"
##   [223] "ENSGACG00000036523" "ENSGACG00000018332" "ENSGACG00000018334"
##   [226] "ENSGACG00000000619" "ENSGACG00000018337" "ENSGACG00000000616"
##   [229] "ENSGACG00000032179" "ENSGACG00000000613" "ENSGACG00000000607"
##   [232] "ENSGACG00000000598" "ENSGACG00000018344" "ENSGACG00000016571"
##   [235] "ENSGACG00000018348" "ENSGACG00000016581" "ENSGACG00000031614"
##   [238] "ENSGACG00000019578" "ENSGACG00000016587" "ENSGACG00000025133"
##   [241] "ENSGACG00000000597" "ENSGACG00000027068" "ENSGACG00000000590"
##   [244] "ENSGACG00000000589" "ENSGACG00000018357" "ENSGACG00000000586"
##   [247] "ENSGACG00000000581" "ENSGACG00000018359" "ENSGACG00000000578"
##   [250] "ENSGACG00000018361" "ENSGACG00000000570" "ENSGACG00000018362"
##   [253] "ENSGACG00000000567" "ENSGACG00000000565" "ENSGACG00000018367"
##   [256] "ENSGACG00000019742" "ENSGACG00000019744" "ENSGACG00000019746"
##   [259] "ENSGACG00000016609" "ENSGACG00000019748" "ENSGACG00000037930"
##   [262] "ENSGACG00000016624" "ENSGACG00000019755" "ENSGACG00000019759"
##   [265] "ENSGACG00000019762" "ENSGACG00000019765" "ENSGACG00000019767"
##   [268] "ENSGACG00000019772" "ENSGACG00000027469" "ENSGACG00000033774"
##   [271] "ENSGACG00000037218" "ENSGACG00000023532" "ENSGACG00000029265"
##   [274] "ENSGACG00000018374" "ENSGACG00000038139" "ENSGACG00000035005"
##   [277] "ENSGACG00000028857" "ENSGACG00000033226" "ENSGACG00000034108"
##   [280] "ENSGACG00000024901" "ENSGACG00000036482" "ENSGACG00000035789"
##   [283] "ENSGACG00000033414" "ENSGACG00000025567" "ENSGACG00000028472"
##   [286] "ENSGACG00000025781" "ENSGACG00000032180" "ENSGACG00000024997"
##   [289] "ENSGACG00000031524" "ENSGACG00000032853" "ENSGACG00000026585"
##   [292] "ENSGACG00000026148" "ENSGACG00000035289" "ENSGACG00000018383"
##   [295] "ENSGACG00000030506" "ENSGACG00000016634" "ENSGACG00000028314"
##   [298] "ENSGACG00000026657" "ENSGACG00000028094" "ENSGACG00000018387"
##   [301] "ENSGACG00000026171" "ENSGACG00000033655" "ENSGACG00000018390"
##   [304] "ENSGACG00000032808" "ENSGACG00000036277" "ENSGACG00000026077"
##   [307] "ENSGACG00000018391" "ENSGACG00000026851" "ENSGACG00000022852"
##   [310] "ENSGACG00000027874" "ENSGACG00000019820" "ENSGACG00000018406"
##   [313] "ENSGACG00000018407" "ENSGACG00000016641" "ENSGACG00000025297"
##   [316] "ENSGACG00000029573" "ENSGACG00000018413" "ENSGACG00000018417"
##   [319] "ENSGACG00000016642" "ENSGACG00000037169" "ENSGACG00000018419"
##   [322] "ENSGACG00000016645" "ENSGACG00000016649" "ENSGACG00000018422"
##   [325] "ENSGACG00000018432" "ENSGACG00000030639" "ENSGACG00000032735"
##   [328] "ENSGACG00000018439" "ENSGACG00000018440" "ENSGACG00000029510"
##   [331] "ENSGACG00000016658" "ENSGACG00000018444" "ENSGACG00000018445"
##   [334] "ENSGACG00000018447" "ENSGACG00000016665" "ENSGACG00000018449"
##   [337] "ENSGACG00000016673" "ENSGACG00000018450" "ENSGACG00000016676"
##   [340] "ENSGACG00000024732" "ENSGACG00000016686" "ENSGACG00000032114"
##   [343] "ENSGACG00000018457" "ENSGACG00000024020" "ENSGACG00000034337"
##   [346] "ENSGACG00000032135" "ENSGACG00000018461" "ENSGACG00000016693"
##   [349] "ENSGACG00000016695" "ENSGACG00000016697" "ENSGACG00000016706"
##   [352] "ENSGACG00000033009" "ENSGACG00000029651" "ENSGACG00000018466"
##   [355] "ENSGACG00000018467" "ENSGACG00000016734" "ENSGACG00000031245"
##   [358] "ENSGACG00000016736" "ENSGACG00000023054" "ENSGACG00000016749"
##   [361] "ENSGACG00000018471" "ENSGACG00000016751" "ENSGACG00000016755"
##   [364] "ENSGACG00000018475" "ENSGACG00000016757" "ENSGACG00000018477"
##   [367] "ENSGACG00000026846" "ENSGACG00000024167" "ENSGACG00000018480"
##   [370] "ENSGACG00000018483" "ENSGACG00000018484" "ENSGACG00000018485"
##   [373] "ENSGACG00000018487" "ENSGACG00000019515" "ENSGACG00000018490"
##   [376] "ENSGACG00000018352" "ENSGACG00000018354" "ENSGACG00000018355"
##   [379] "ENSGACG00000016791" "ENSGACG00000018602" "ENSGACG00000016772"
##   [382] "ENSGACG00000016794" "ENSGACG00000018605" "ENSGACG00000018608"
##   [385] "ENSGACG00000016796" "ENSGACG00000016798" "ENSGACG00000016803"
##   [388] "ENSGACG00000018610" "ENSGACG00000018613" "ENSGACG00000016808"
##   [391] "ENSGACG00000018533" "ENSGACG00000036867" "ENSGACG00000016811"
##   [394] "ENSGACG00000016814" "ENSGACG00000016819" "ENSGACG00000016821"
##   [397] "ENSGACG00000016824" "ENSGACG00000018534" "ENSGACG00000032327"
##   [400] "ENSGACG00000016829" "ENSGACG00000024987" "ENSGACG00000016847"
##   [403] "ENSGACG00000016848" "ENSGACG00000016854" "ENSGACG00000037224"
##   [406] "ENSGACG00000017237" "ENSGACG00000017253" "ENSGACG00000017265"
##   [409] "ENSGACG00000017268" "ENSGACG00000017286" "ENSGACG00000017288"
##   [412] "ENSGACG00000031359" "ENSGACG00000028630" "ENSGACG00000028441"
##   [415] "ENSGACG00000018541" "ENSGACG00000017306" "ENSGACG00000018542"
##   [418] "ENSGACG00000017312" "ENSGACG00000017316" "ENSGACG00000017318"
##   [421] "ENSGACG00000030577" "ENSGACG00000017324" "ENSGACG00000018543"
##   [424] "ENSGACG00000018545" "ENSGACG00000017326" "ENSGACG00000029687"
##   [427] "ENSGACG00000017346" "ENSGACG00000017332" "ENSGACG00000017334"
##   [430] "ENSGACG00000017349" "ENSGACG00000017351" "ENSGACG00000017353"
##   [433] "ENSGACG00000017357" "ENSGACG00000017362" "ENSGACG00000031693"
##   [436] "ENSGACG00000016217" "ENSGACG00000016220" "ENSGACG00000025670"
##   [439] "ENSGACG00000018550" "ENSGACG00000032235" "ENSGACG00000016231"
##   [442] "ENSGACG00000016234" "ENSGACG00000016271" "ENSGACG00000027170"
##   [445] "ENSGACG00000016277" "ENSGACG00000018552" "ENSGACG00000028925"
##   [448] "ENSGACG00000018559" "ENSGACG00000016279" "ENSGACG00000030177"
##   [451] "ENSGACG00000023452" "ENSGACG00000018574" "ENSGACG00000028189"
##   [454] "ENSGACG00000030362" "ENSGACG00000016283" "ENSGACG00000037117"
##   [457] "ENSGACG00000016300" "ENSGACG00000018580" "ENSGACG00000018585"
##   [460] "ENSGACG00000032382" "ENSGACG00000018587" "ENSGACG00000016304"
##   [463] "ENSGACG00000018595" "ENSGACG00000018598" "ENSGACG00000018601"
##   [466] "ENSGACG00000018493" "ENSGACG00000016311" "ENSGACG00000016318"
##   [469] "ENSGACG00000026351" "ENSGACG00000016321" "ENSGACG00000016326"
##   [472] "ENSGACG00000030496" "ENSGACG00000016332" "ENSGACG00000016337"
##   [475] "ENSGACG00000016341" "ENSGACG00000016344" "ENSGACG00000027038"
##   [478] "ENSGACG00000016353" "ENSGACG00000026201" "ENSGACG00000016358"
##   [481] "ENSGACG00000016360" "ENSGACG00000016362" "ENSGACG00000016363"
##   [484] "ENSGACG00000016366" "ENSGACG00000016370" "ENSGACG00000033764"
##   [487] "ENSGACG00000016372" "ENSGACG00000016381" "ENSGACG00000017723"
##   [490] "ENSGACG00000016388" "ENSGACG00000023006" "ENSGACG00000016398"
##   [493] "ENSGACG00000027606" "ENSGACG00000016402" "ENSGACG00000024639"
##   [496] "ENSGACG00000029689" "ENSGACG00000016424" "ENSGACG00000016411"
##   [499] "ENSGACG00000027989" "ENSGACG00000018498" "ENSGACG00000018503"
##   [502] "ENSGACG00000031806" "ENSGACG00000023310" "ENSGACG00000016431"
##   [505] "ENSGACG00000016437" "ENSGACG00000016443" "ENSGACG00000018508"
##   [508] "ENSGACG00000018511" "ENSGACG00000028756" "ENSGACG00000016449"
##   [511] "ENSGACG00000016452" "ENSGACG00000018514" "ENSGACG00000025656"
##   [514] "ENSGACG00000016458" "ENSGACG00000033815" "ENSGACG00000017717"
##   [517] "ENSGACG00000017720" "ENSGACG00000016862" "ENSGACG00000016868"
##   [520] "ENSGACG00000016876" "ENSGACG00000016877" "ENSGACG00000016886"
##   [523] "ENSGACG00000016892" "ENSGACG00000036527" "ENSGACG00000026360"
##   [526] "ENSGACG00000023234" "ENSGACG00000016915" "ENSGACG00000016922"
##   [529] "ENSGACG00000028827" "ENSGACG00000016924" "ENSGACG00000016927"
##   [532] "ENSGACG00000035530" "ENSGACG00000031439" "ENSGACG00000025862"
##   [535] "ENSGACG00000016933" "ENSGACG00000016934" "ENSGACG00000016958"
##   [538] "ENSGACG00000016967" "ENSGACG00000016971" "ENSGACG00000030006"
##   [541] "ENSGACG00000029740" "ENSGACG00000033799" "ENSGACG00000016980"
##   [544] "ENSGACG00000023598" "ENSGACG00000017001" "ENSGACG00000023689"
##   [547] "ENSGACG00000017006" "ENSGACG00000034247" "ENSGACG00000031920"
##   [550] "ENSGACG00000017411" "ENSGACG00000017415" "ENSGACG00000017418"
##   [553] "ENSGACG00000034679" "ENSGACG00000032674" "ENSGACG00000017434"
##   [556] "ENSGACG00000017435" "ENSGACG00000017440" "ENSGACG00000035105"
##   [559] "ENSGACG00000032572" "ENSGACG00000028135" "ENSGACG00000017441"
##   [562] "ENSGACG00000018529" "ENSGACG00000018525" "ENSGACG00000029248"
##   [565] "ENSGACG00000018527" "ENSGACG00000017463" "ENSGACG00000017466"
##   [568] "ENSGACG00000017467" "ENSGACG00000030601" "ENSGACG00000028860"
##   [571] "ENSGACG00000017470" "ENSGACG00000017478" "ENSGACG00000033285"
##   [574] "ENSGACG00000017483" "ENSGACG00000018532" "ENSGACG00000027482"
##   [577] "ENSGACG00000018620" "ENSGACG00000018623" "ENSGACG00000018626"
##   [580] "ENSGACG00000018631" "ENSGACG00000018634" "ENSGACG00000018647"
##   [583] "ENSGACG00000018642" "ENSGACG00000018655" "ENSGACG00000026066"
##   [586] "ENSGACG00000018656" "ENSGACG00000035904" "ENSGACG00000033486"
##   [589] "ENSGACG00000018659" "ENSGACG00000027313" "ENSGACG00000018663"
##   [592] "ENSGACG00000017493" "ENSGACG00000018664" "ENSGACG00000018665"
##   [595] "ENSGACG00000029861" "ENSGACG00000018678" "ENSGACG00000018679"
##   [598] "ENSGACG00000029784" "ENSGACG00000018682" "ENSGACG00000025804"
##   [601] "ENSGACG00000018684" "ENSGACG00000018687" "ENSGACG00000018696"
##   [604] "ENSGACG00000018693" "ENSGACG00000018695" "ENSGACG00000018700"
##   [607] "ENSGACG00000018709" "ENSGACG00000018710" "ENSGACG00000018712"
##   [610] "ENSGACG00000018714" "ENSGACG00000018718" "ENSGACG00000018723"
##   [613] "ENSGACG00000018724" "ENSGACG00000018727" "ENSGACG00000017497"
##   [616] "ENSGACG00000028115" "ENSGACG00000018729" "ENSGACG00000018730"
##   [619] "ENSGACG00000018731" "ENSGACG00000018733" "ENSGACG00000018734"
##   [622] "ENSGACG00000018736" "ENSGACG00000018740" "ENSGACG00000025001"
##   [625] "ENSGACG00000017504" "ENSGACG00000018050" "ENSGACG00000029915"
##   [628] "ENSGACG00000018053" "ENSGACG00000028766" "ENSGACG00000017515"
##   [631] "ENSGACG00000018062" "ENSGACG00000017518" "ENSGACG00000017521"
##   [634] "ENSGACG00000017524" "ENSGACG00000017526" "ENSGACG00000017530"
##   [637] "ENSGACG00000036678" "ENSGACG00000017532" "ENSGACG00000018076"
##   [640] "ENSGACG00000033122" "ENSGACG00000018083" "ENSGACG00000017551"
##   [643] "ENSGACG00000017554" "ENSGACG00000018098" "ENSGACG00000018103"
##   [646] "ENSGACG00000031122" "ENSGACG00000018115" "ENSGACG00000018116"
##   [649] "ENSGACG00000017557" "ENSGACG00000018119" "ENSGACG00000023859"
##   [652] "ENSGACG00000018122" "ENSGACG00000029254" "ENSGACG00000031417"
##   [655] "ENSGACG00000017569" "ENSGACG00000017574" "ENSGACG00000017578"
##   [658] "ENSGACG00000017580" "ENSGACG00000018129" "ENSGACG00000017583"
##   [661] "ENSGACG00000018131" "ENSGACG00000017592" "ENSGACG00000017599"
##   [664] "ENSGACG00000017605" "ENSGACG00000034830" "ENSGACG00000032866"
##   [667] "ENSGACG00000017621" "ENSGACG00000017628" "ENSGACG00000017632"
##   [670] "ENSGACG00000017637" "ENSGACG00000017642" "ENSGACG00000017643"
##   [673] "ENSGACG00000017664" "ENSGACG00000017666" "ENSGACG00000017671"
##   [676] "ENSGACG00000032725" "ENSGACG00000017677" "ENSGACG00000027301"
##   [679] "ENSGACG00000017681" "ENSGACG00000017686" "ENSGACG00000018133"
##   [682] "ENSGACG00000029486" "ENSGACG00000031836" "ENSGACG00000018139"
##   [685] "ENSGACG00000035580" "ENSGACG00000018141" "ENSGACG00000024201"
##   [688] "ENSGACG00000018144" "ENSGACG00000018147" "ENSGACG00000017692"
##   [691] "ENSGACG00000017694" "ENSGACG00000023922" "ENSGACG00000018149"
##   [694] "ENSGACG00000018152" "ENSGACG00000018159" "ENSGACG00000018161"
##   [697] "ENSGACG00000033990" "ENSGACG00000017367" "ENSGACG00000018167"
##   [700] "ENSGACG00000017379" "ENSGACG00000017372" "ENSGACG00000034986"
##   [703] "ENSGACG00000017706" "ENSGACG00000018169" "ENSGACG00000027745"
##   [706] "ENSGACG00000018171" "ENSGACG00000017733" "ENSGACG00000018175"
##   [709] "ENSGACG00000017735" "ENSGACG00000017740" "ENSGACG00000018181"
##   [712] "ENSGACG00000017741" "ENSGACG00000038123" "ENSGACG00000018186"
##   [715] "ENSGACG00000025762" "ENSGACG00000018188" "ENSGACG00000018200"
##   [718] "ENSGACG00000033299" "ENSGACG00000033942" "ENSGACG00000018209"
##   [721] "ENSGACG00000018211" "ENSGACG00000018213" "ENSGACG00000018214"
##   [724] "ENSGACG00000017743" "ENSGACG00000017755" "ENSGACG00000018218"
##   [727] "ENSGACG00000017764" "ENSGACG00000017769" "ENSGACG00000017773"
##   [730] "ENSGACG00000017788" "ENSGACG00000017801" "ENSGACG00000023896"
##   [733] "ENSGACG00000017819" "ENSGACG00000017823" "ENSGACG00000017825"
##   [736] "ENSGACG00000017830" "ENSGACG00000017832" "ENSGACG00000031811"
##   [739] "ENSGACG00000032052" "ENSGACG00000036472" "ENSGACG00000017837"
##   [742] "ENSGACG00000018224" "ENSGACG00000026685" "ENSGACG00000032085"
##   [745] "ENSGACG00000018229" "ENSGACG00000017846" "ENSGACG00000018231"
##   [748] "ENSGACG00000027016" "ENSGACG00000017863" "ENSGACG00000035372"
##   [751] "ENSGACG00000027771" "ENSGACG00000026911" "ENSGACG00000017868"
##   [754] "ENSGACG00000017871" "ENSGACG00000018242" "ENSGACG00000026432"
##   [757] "ENSGACG00000018249" "ENSGACG00000017018" "ENSGACG00000017022"
##   [760] "ENSGACG00000018253" "ENSGACG00000017027" "ENSGACG00000035039"
##   [763] "ENSGACG00000028688" "ENSGACG00000024753" "ENSGACG00000018256"
##   [766] "ENSGACG00000017040" "ENSGACG00000018258" "ENSGACG00000025182"
##   [769] "ENSGACG00000018263" "ENSGACG00000026700" "ENSGACG00000033228"
##   [772] "ENSGACG00000018268" "ENSGACG00000017051" "ENSGACG00000026213"
##   [775] "ENSGACG00000034364" "ENSGACG00000032120" "ENSGACG00000017064"
##   [778] "ENSGACG00000018273" "ENSGACG00000017074" "ENSGACG00000023230"
##   [781] "ENSGACG00000017077" "ENSGACG00000023672" "ENSGACG00000020692"
##   [784] "ENSGACG00000017103" "ENSGACG00000017106" "ENSGACG00000017117"
##   [787] "ENSGACG00000017122" "ENSGACG00000017128" "ENSGACG00000018281"
##   [790] "ENSGACG00000018279" "ENSGACG00000018285" "ENSGACG00000018286"
##   [793] "ENSGACG00000018289" "ENSGACG00000028194" "ENSGACG00000026282"
##   [796] "ENSGACG00000018296" "ENSGACG00000019572" "ENSGACG00000019568"
##   [799] "ENSGACG00000019563" "ENSGACG00000019560" "ENSGACG00000019558"
##   [802] "ENSGACG00000019554" "ENSGACG00000019555" "ENSGACG00000018742"
##   [805] "ENSGACG00000018752" "ENSGACG00000018757" "ENSGACG00000018763"
##   [808] "ENSGACG00000018765" "ENSGACG00000018769" "ENSGACG00000018770"
##   [811] "ENSGACG00000025048" "ENSGACG00000018774" "ENSGACG00000018789"
##   [814] "ENSGACG00000018791" "ENSGACG00000018795" "ENSGACG00000018799"
##   [817] "ENSGACG00000036475" "ENSGACG00000019653" "ENSGACG00000019651"
##   [820] "ENSGACG00000024573" "ENSGACG00000019644" "ENSGACG00000029658"
##   [823] "ENSGACG00000019640" "ENSGACG00000027564" "ENSGACG00000024955"
##   [826] "ENSGACG00000019625" "ENSGACG00000032436" "ENSGACG00000019621"
##   [829] "ENSGACG00000019619" "ENSGACG00000019617" "ENSGACG00000019611"
##   [832] "ENSGACG00000030378" "ENSGACG00000019609" "ENSGACG00000019604"
##   [835] "ENSGACG00000019602" "ENSGACG00000019599" "ENSGACG00000019596"
##   [838] "ENSGACG00000019594" "ENSGACG00000019588" "ENSGACG00000019586"
##   [841] "ENSGACG00000026933" "ENSGACG00000018351" "ENSGACG00000017915"
##   [844] "ENSGACG00000029626" "ENSGACG00000030940" "ENSGACG00000036234"
##   [847] "ENSGACG00000032839" "ENSGACG00000034203" "ENSGACG00000034379"
##   [850] "ENSGACG00000034170" "ENSGACG00000029240" "ENSGACG00000017920"
##   [853] "ENSGACG00000017921" "ENSGACG00000017925" "ENSGACG00000017928"
##   [856] "ENSGACG00000017934" "ENSGACG00000017939" "ENSGACG00000017940"
##   [859] "ENSGACG00000017942" "ENSGACG00000017135" "ENSGACG00000017944"
##   [862] "ENSGACG00000031732" "ENSGACG00000017946" "ENSGACG00000017954"
##   [865] "ENSGACG00000017146" "ENSGACG00000017958" "ENSGACG00000017960"
##   [868] "ENSGACG00000017153" "ENSGACG00000017962" "ENSGACG00000025609"
##   [871] "ENSGACG00000023568" "ENSGACG00000017963" "ENSGACG00000017966"
##   [874] "ENSGACG00000017971" "ENSGACG00000017972" "ENSGACG00000017170"
##   [877] "ENSGACG00000017174" "ENSGACG00000017976" "ENSGACG00000031393"
##   [880] "ENSGACG00000017981" "ENSGACG00000036586" "ENSGACG00000031088"
##   [883] "ENSGACG00000017183" "ENSGACG00000017982" "ENSGACG00000017988"
##   [886] "ENSGACG00000017990" "ENSGACG00000017993" "ENSGACG00000017995"
##   [889] "ENSGACG00000017998" "ENSGACG00000028573" "ENSGACG00000018005"
##   [892] "ENSGACG00000018007" "ENSGACG00000017190" "ENSGACG00000017195"
##   [895] "ENSGACG00000017204" "ENSGACG00000017211" "ENSGACG00000017212"
##   [898] "ENSGACG00000017216" "ENSGACG00000017218" "ENSGACG00000017010"
##   [901] "ENSGACG00000017013" "ENSGACG00000017015" "ENSGACG00000033342"
##   [904] "ENSGACG00000017907" "ENSGACG00000036991" "ENSGACG00000034903"
##   [907] "ENSGACG00000017877" "ENSGACG00000018011" "ENSGACG00000028906"
##   [910] "ENSGACG00000018016" "ENSGACG00000018023" "ENSGACG00000018027"
##   [913] "ENSGACG00000017881" "ENSGACG00000018029" "ENSGACG00000018030"
##   [916] "ENSGACG00000017882" "ENSGACG00000018033" "ENSGACG00000017883"
##   [919] "ENSGACG00000027065" "ENSGACG00000018035" "ENSGACG00000025526"
##   [922] "ENSGACG00000018036" "ENSGACG00000017891" "ENSGACG00000037628"
##   [925] "ENSGACG00000031016" "ENSGACG00000018046" "ENSGACG00000018420"
##   [928] "ENSGACG00000036174" "ENSGACG00000036810" "ENSGACG00000035672"
##   [931] "ENSGACG00000030516" "ENSGACG00000037468" "ENSGACG00000031357"
##   [934] "ENSGACG00000036962" "ENSGACG00000030278" "ENSGACG00000035912"
##   [937] "ENSGACG00000033072" "ENSGACG00000024460" "ENSGACG00000017896"
##   [940] "ENSGACG00000026137" "ENSGACG00000036341" "ENSGACG00000027026"
##   [943] "ENSGACG00000037519" "ENSGACG00000023166" "ENSGACG00000033165"
##   [946] "ENSGACG00000024315" "ENSGACG00000030731" "ENSGACG00000034034"
##   [949] "ENSGACG00000029733" "ENSGACG00000027115" "ENSGACG00000034698"
##   [952] "ENSGACG00000035369" "ENSGACG00000029993" "ENSGACG00000034024"
##   [955] "ENSGACG00000035545" "ENSGACG00000026898" "ENSGACG00000026893"
##   [958] "ENSGACG00000030742" "ENSGACG00000017901" "ENSGACG00000025658"
##   [961] "ENSGACG00000024959" "ENSGACG00000017910" "ENSGACG00000029639"
##   [964] "ENSGACG00000017912" "ENSGACG00000027139" "ENSGACG00000017913"
##   [967] "ENSGACG00000033533" "ENSGACG00000027036" "ENSGACG00000034783"
##   [970] "ENSGACG00000036818" "ENSGACG00000032115" "ENSGACG00000023814"
##   [973] "ENSGACG00000032983" "ENSGACG00000026400" "ENSGACG00000017914"
##   [976] "ENSGACG00000036669" "ENSGACG00000035604" "ENSGACG00000026589"
##   [979] "ENSGACG00000017697" "ENSGACG00000031270" "ENSGACG00000027659"
##   [982] "ENSGACG00000033043" "ENSGACG00000034896" "ENSGACG00000032830"
##   [985] "ENSGACG00000018556" "ENSGACG00000024601" "ENSGACG00000033430"
##   [988] "ENSGACG00000028218" "ENSGACG00000036221" "ENSGACG00000035058"
##   [991] "ENSGACG00000031831" "ENSGACG00000028097" "ENSGACG00000033979"
##   [994] "ENSGACG00000024771" "ENSGACG00000023802" "ENSGACG00000024003"
##   [997] "ENSGACG00000033059" "ENSGACG00000036242" "ENSGACG00000035980"
##  [1000] "ENSGACG00000037338" "ENSGACG00000027726" "ENSGACG00000028530"
##  [1003] "ENSGACG00000036544" "ENSGACG00000032801" "ENSGACG00000030199"
##  [1006] "ENSGACG00000034536" "ENSGACG00000028118" "ENSGACG00000033849"
##  [1009] "ENSGACG00000030022" "ENSGACG00000038044" "ENSGACG00000037808"
##  [1012] "ENSGACG00000035280" "ENSGACG00000023140" "ENSGACG00000033559"
##  [1015] "ENSGACG00000029017" "ENSGACG00000028346" "ENSGACG00000030607"
##  [1018] "ENSGACG00000032667" "ENSGACG00000027123" "ENSGACG00000032697"
##  [1021] "ENSGACG00000025361" "ENSGACG00000033949" "ENSGACG00000029457"
##  [1024] "ENSGACG00000035741" "ENSGACG00000023266" "ENSGACG00000027353"
##  [1027] "ENSGACG00000034282" "ENSGACG00000035802" "ENSGACG00000023584"
##  [1030] "ENSGACG00000031301" "ENSGACG00000032574" "ENSGACG00000034529"
##  [1033] "ENSGACG00000032468" "ENSGACG00000024493" "ENSGACG00000024298"
##  [1036] "ENSGACG00000033716" "ENSGACG00000034832" "ENSGACG00000032125"
##  [1039] "ENSGACG00000034472" "ENSGACG00000035181" "ENSGACG00000034893"
##  [1042] "ENSGACG00000031383" "ENSGACG00000025829" "ENSGACG00000025718"
##  [1045] "ENSGACG00000024018" "ENSGACG00000023940" "ENSGACG00000024096"
##  [1048] "ENSGACG00000028342" "ENSGACG00000018853" "ENSGACG00000034199"
##  [1051] "ENSGACG00000029446" "ENSGACG00000018852" "ENSGACG00000018851"
##  [1054] "ENSGACG00000018960" "ENSGACG00000031779" "ENSGACG00000028650"
##  [1057] "ENSGACG00000027220" "ENSGACG00000033599" "ENSGACG00000025586"
##  [1060] "ENSGACG00000037234" "ENSGACG00000018408" "ENSGACG00000025380"
##  [1063] "ENSGACG00000018958" "ENSGACG00000018509" "ENSGACG00000025600"
##  [1066] "ENSGACG00000034514" "ENSGACG00000031011" "ENSGACG00000028976"
##  [1069] "ENSGACG00000028480" "ENSGACG00000034294" "ENSGACG00000029462"
##  [1072] "ENSGACG00000016763" "ENSGACG00000026424" "ENSGACG00000018929"
##  [1075] "ENSGACG00000033623" "ENSGACG00000017702" "ENSGACG00000017810"
##  [1078] "ENSGACG00000034983" "ENSGACG00000029599" "ENSGACG00000026086"
##  [1081] "ENSGACG00000017083" "ENSGACG00000017885" "ENSGACG00000018923"
##  [1084] "ENSGACG00000017905" "ENSGACG00000018919" "ENSGACG00000018918"
##  [1087] "ENSGACG00000036936" "ENSGACG00000025850" "ENSGACG00000023316"
##  [1090] "ENSGACG00000018910" "ENSGACG00000018894" "ENSGACG00000018891"
##  [1093] "ENSGACG00000028280" "ENSGACG00000023507" "ENSGACG00000018884"
##  [1096] "ENSGACG00000018882" "ENSGACG00000018875" "ENSGACG00000018865"
##  [1099] "ENSGACG00000018864" "ENSGACG00000018862" "ENSGACG00000033094"
##  [1102] "ENSGACG00000025057" "ENSGACG00000018857" "ENSGACG00000018854"
##  [1105] "ENSGACG00000019108" "ENSGACG00000019105" "ENSGACG00000019107"
##  [1108] "ENSGACG00000019104" "ENSGACG00000036743" "ENSGACG00000019092"
##  [1111] "ENSGACG00000025227" "ENSGACG00000019084" "ENSGACG00000019082"
##  [1114] "ENSGACG00000019075" "ENSGACG00000019071" "ENSGACG00000019068"
##  [1117] "ENSGACG00000025508" "ENSGACG00000032502" "ENSGACG00000019066"
##  [1120] "ENSGACG00000019037" "ENSGACG00000019063" "ENSGACG00000019061"
##  [1123] "ENSGACG00000019036" "ENSGACG00000023272" "ENSGACG00000019034"
##  [1126] "ENSGACG00000019033" "ENSGACG00000019027" "ENSGACG00000028204"
##  [1129] "ENSGACG00000025330" "ENSGACG00000019025" "ENSGACG00000019024"
##  [1132] "ENSGACG00000019020" "ENSGACG00000019018" "ENSGACG00000019016"
##  [1135] "ENSGACG00000019014" "ENSGACG00000031910" "ENSGACG00000019009"
##  [1138] "ENSGACG00000019008" "ENSGACG00000019007" "ENSGACG00000019005"
##  [1141] "ENSGACG00000019001" "ENSGACG00000034450" "ENSGACG00000018998"
##  [1144] "ENSGACG00000018997" "ENSGACG00000018981" "ENSGACG00000018983"
##  [1147] "ENSGACG00000018969" "ENSGACG00000018967" "ENSGACG00000018950"
##  [1150] "ENSGACG00000018956" "ENSGACG00000018953" "ENSGACG00000018951"
##  [1153] "ENSGACG00000028220" "ENSGACG00000018942" "ENSGACG00000018938"
##  [1156] "ENSGACG00000023440" "ENSGACG00000018935" "ENSGACG00000025481"
##  [1159] "ENSGACG00000018930" "ENSGACG00000019150" "ENSGACG00000019148"
##  [1162] "ENSGACG00000019147" "ENSGACG00000019145" "ENSGACG00000019139"
##  [1165] "ENSGACG00000019136" "ENSGACG00000032712" "ENSGACG00000019125"
##  [1168] "ENSGACG00000027070" "ENSGACG00000019123" "ENSGACG00000019121"
##  [1171] "ENSGACG00000019113" "ENSGACG00000019120" "ENSGACG00000019116"
##  [1174] "ENSGACG00000019115" "ENSGACG00000019114" "ENSGACG00000029101"
##  [1177] "ENSGACG00000019542" "ENSGACG00000019540" "ENSGACG00000019538"
##  [1180] "ENSGACG00000019534" "ENSGACG00000024651" "ENSGACG00000019532"
##  [1183] "ENSGACG00000027058" "ENSGACG00000019527" "ENSGACG00000019525"
##  [1186] "ENSGACG00000031852" "ENSGACG00000019522" "ENSGACG00000033679"
##  [1189] "ENSGACG00000019520" "ENSGACG00000029646" "ENSGACG00000018489"
##  [1192] "ENSGACG00000002171" "ENSGACG00000019509" "ENSGACG00000028267"
##  [1195] "ENSGACG00000019508" "ENSGACG00000019506" "ENSGACG00000025218"
##  [1198] "ENSGACG00000019505" "ENSGACG00000019497" "ENSGACG00000019496"
##  [1201] "ENSGACG00000019495" "ENSGACG00000019490" "ENSGACG00000019485"
##  [1204] "ENSGACG00000019481" "ENSGACG00000019472" "ENSGACG00000019468"
##  [1207] "ENSGACG00000019467" "ENSGACG00000019463" "ENSGACG00000019461"
##  [1210] "ENSGACG00000019459" "ENSGACG00000019457" "ENSGACG00000038102"
##  [1213] "ENSGACG00000019447" "ENSGACG00000019445" "ENSGACG00000019441"
##  [1216] "ENSGACG00000034749" "ENSGACG00000019422" "ENSGACG00000019413"
##  [1219] "ENSGACG00000019404" "ENSGACG00000019399" "ENSGACG00000019389"
##  [1222] "ENSGACG00000019386" "ENSGACG00000019383" "ENSGACG00000019373"
##  [1225] "ENSGACG00000019367" "ENSGACG00000035156" "ENSGACG00000019364"
##  [1228] "ENSGACG00000019363" "ENSGACG00000019348" "ENSGACG00000019346"
##  [1231] "ENSGACG00000019345" "ENSGACG00000023708" "ENSGACG00000018846"
##  [1234] "ENSGACG00000018843" "ENSGACG00000036138" "ENSGACG00000018841"
##  [1237] "ENSGACG00000018838" "ENSGACG00000018836" "ENSGACG00000023138"
##  [1240] "ENSGACG00000034033" "ENSGACG00000018829" "ENSGACG00000037944"
##  [1243] "ENSGACG00000018824" "ENSGACG00000018821" "ENSGACG00000018820"
##  [1246] "ENSGACG00000031956" "ENSGACG00000018818" "ENSGACG00000018815"
##  [1249] "ENSGACG00000018814" "ENSGACG00000018812" "ENSGACG00000036284"
##  [1252] "ENSGACG00000023247" "ENSGACG00000034264" "ENSGACG00000018804"
##  [1255] "ENSGACG00000018803" "ENSGACG00000019654" "ENSGACG00000019657"
##  [1258] "ENSGACG00000034580" "ENSGACG00000032317" "ENSGACG00000019660"
##  [1261] "ENSGACG00000019662" "ENSGACG00000019663" "ENSGACG00000019666"
##  [1264] "ENSGACG00000019667" "ENSGACG00000024090" "ENSGACG00000019673"
##  [1267] "ENSGACG00000019674" "ENSGACG00000019679" "ENSGACG00000019680"
##  [1270] "ENSGACG00000019683" "ENSGACG00000019685" "ENSGACG00000019690"
##  [1273] "ENSGACG00000029723" "ENSGACG00000019692" "ENSGACG00000019694"
##  [1276] "ENSGACG00000024864" "ENSGACG00000019698" "ENSGACG00000019702"
##  [1279] "ENSGACG00000019703" "ENSGACG00000019705" "ENSGACG00000019706"
##  [1282] "ENSGACG00000019709" "ENSGACG00000019712" "ENSGACG00000019718"
##  [1285] "ENSGACG00000019720" "ENSGACG00000019721" "ENSGACG00000035645"
##  [1288] "ENSGACG00000019725" "ENSGACG00000024062" "ENSGACG00000019732"
##  [1291] "ENSGACG00000019740" "ENSGACG00000019741" "ENSGACG00000019344"
##  [1294] "ENSGACG00000019342" "ENSGACG00000019341" "ENSGACG00000037303"
##  [1297] "ENSGACG00000019336" "ENSGACG00000019334" "ENSGACG00000019333"
##  [1300] "ENSGACG00000019330" "ENSGACG00000019325" "ENSGACG00000019323"
##  [1303] "ENSGACG00000019321" "ENSGACG00000019320" "ENSGACG00000019313"
##  [1306] "ENSGACG00000019311" "ENSGACG00000019309" "ENSGACG00000019302"
##  [1309] "ENSGACG00000019293" "ENSGACG00000019291" "ENSGACG00000019287"
##  [1312] "ENSGACG00000032660" "ENSGACG00000031404" "ENSGACG00000019271"
##  [1315] "ENSGACG00000019270" "ENSGACG00000025721" "ENSGACG00000019269"
##  [1318] "ENSGACG00000035217" "ENSGACG00000019264" "ENSGACG00000035523"
##  [1321] "ENSGACG00000019259" "ENSGACG00000019257" "ENSGACG00000019253"
##  [1324] "ENSGACG00000019248" "ENSGACG00000019242" "ENSGACG00000019247"
##  [1327] "ENSGACG00000019240" "ENSGACG00000031889" "ENSGACG00000019236"
##  [1330] "ENSGACG00000019233" "ENSGACG00000019230" "ENSGACG00000023470"
##  [1333] "ENSGACG00000019227" "ENSGACG00000019223" "ENSGACG00000019221"
##  [1336] "ENSGACG00000001120" "ENSGACG00000037511" "ENSGACG00000037907"
##  [1339] "ENSGACG00000026257" "ENSGACG00000034457" "ENSGACG00000037008"
##  [1342] "ENSGACG00000001041" "ENSGACG00000035845" "ENSGACG00000031415"
##  [1345] "ENSGACG00000001005" "ENSGACG00000001000" "ENSGACG00000000995"
##  [1348] "ENSGACG00000028828" "ENSGACG00000025988" "ENSGACG00000019216"
##  [1351] "ENSGACG00000037072" "ENSGACG00000019214" "ENSGACG00000019212"
##  [1354] "ENSGACG00000019208" "ENSGACG00000019206" "ENSGACG00000019204"
##  [1357] "ENSGACG00000019202" "ENSGACG00000019193" "ENSGACG00000019192"
##  [1360] "ENSGACG00000036238" "ENSGACG00000014055" "ENSGACG00000019186"
##  [1363] "ENSGACG00000019185" "ENSGACG00000019173" "ENSGACG00000019184"
##  [1366] "ENSGACG00000019176" "ENSGACG00000019174" "ENSGACG00000019171"
##  [1369] "ENSGACG00000019169" "ENSGACG00000019163" "ENSGACG00000019160"
##  [1372] "ENSGACG00000019159" "ENSGACG00000019156" "ENSGACG00000019155"
##  [1375] "ENSGACG00000033797" "ENSGACG00000033614" "ENSGACG00000024649"
##  [1378] "ENSGACG00000029498" "ENSGACG00000030778" "ENSGACG00000023744"
##  [1381] "ENSGACG00000035023" "ENSGACG00000024309" "ENSGACG00000027402"
##  [1384] "ENSGACG00000024336" "ENSGACG00000030614" "ENSGACG00000028996"
##  [1387] "ENSGACG00000023496" "ENSGACG00000033484" "ENSGACG00000035896"
##  [1390] "ENSGACG00000023742" "ENSGACG00000033901" "ENSGACG00000030549"
##  [1393] "ENSGACG00000025101" "ENSGACG00000024172" "ENSGACG00000031451"
##  [1396] "ENSGACG00000028222" "ENSGACG00000028270" "ENSGACG00000026970"
##  [1399] "ENSGACG00000029959" "ENSGACG00000034925" "ENSGACG00000035038"
##  [1402] "ENSGACG00000026105" "ENSGACG00000031221" "ENSGACG00000036662"
##  [1405] "ENSGACG00000024347" "ENSGACG00000028261" "ENSGACG00000024754"
##  [1408] "ENSGACG00000027783" "ENSGACG00000023810" "ENSGACG00000023093"
##  [1411] "ENSGACG00000037787" "ENSGACG00000018911" "ENSGACG00000018945"
##  [1414] "ENSGACG00000037070" "ENSGACG00000019519" "ENSGACG00000020890"
##  [1417] "ENSGACG00000020892" "ENSGACG00000033922" "ENSGACG00000020907"
##  [1420] "ENSGACG00000032825" "ENSGACG00000033058" "ENSGACG00000020895"
##  [1423] "ENSGACG00000035301" "ENSGACG00000020898" "ENSGACG00000020899"
##  [1426] "ENSGACG00000020900" "ENSGACG00000020901" "ENSGACG00000020902"
##  [1429] "ENSGACG00000020903" "ENSGACG00000036914" "ENSGACG00000020905"
##  [1432] "ENSGACG00000026156" "ENSGACG00000020908" "ENSGACG00000031685"
##  [1435] "ENSGACG00000020910" "ENSGACG00000027872" "ENSGACG00000020913"
##  [1438] "ENSGACG00000020914" "ENSGACG00000020915" "ENSGACG00000020916"
##  [1441] "ENSGACG00000020917" "ENSGACG00000024431" "ENSGACG00000020918"
##  [1444] "ENSGACG00000001953" "ENSGACG00000020888" "ENSGACG00000020889"
##  [1447] "ENSGACG00000031875" "ENSGACG00000027737" "ENSGACG00000029165"
##  [1450] "ENSGACG00000020240" "ENSGACG00000020043" "ENSGACG00000020057"
##  [1453] "ENSGACG00000020061" "ENSGACG00000020062" "ENSGACG00000020063"
##  [1456] "ENSGACG00000020066" "ENSGACG00000020069" "ENSGACG00000020071"
##  [1459] "ENSGACG00000020072" "ENSGACG00000020077" "ENSGACG00000020078"
##  [1462] "ENSGACG00000020082" "ENSGACG00000035852" "ENSGACG00000035234"
##  [1465] "ENSGACG00000001601" "ENSGACG00000034676" "ENSGACG00000001623"
##  [1468] "ENSGACG00000001637" "ENSGACG00000032739" "ENSGACG00000027676"
##  [1471] "ENSGACG00000000974" "ENSGACG00000000966" "ENSGACG00000000961"
##  [1474] "ENSGACG00000032591" "ENSGACG00000027503" "ENSGACG00000000958"
##  [1477] "ENSGACG00000020212" "ENSGACG00000020213" "ENSGACG00000000753"
##  [1480] "ENSGACG00000000751" "ENSGACG00000024229" "ENSGACG00000000745"
##  [1483] "ENSGACG00000033678" "ENSGACG00000036956" "ENSGACG00000000735"
##  [1486] "ENSGACG00000000733" "ENSGACG00000000731" "ENSGACG00000020312"
##  [1489] "ENSGACG00000000941" "ENSGACG00000000932" "ENSGACG00000019877"
##  [1492] "ENSGACG00000036825" "ENSGACG00000019881" "ENSGACG00000000929"
##  [1495] "ENSGACG00000019884" "ENSGACG00000019331" "ENSGACG00000035255"
##  [1498] "ENSGACG00000032080" "ENSGACG00000000917" "ENSGACG00000023505"
##  [1501] "ENSGACG00000019339" "ENSGACG00000019347" "ENSGACG00000019349"
##  [1504] "ENSGACG00000019350" "ENSGACG00000025224" "ENSGACG00000019354"
##  [1507] "ENSGACG00000019358" "ENSGACG00000000901" "ENSGACG00000019361"
##  [1510] "ENSGACG00000000892" "ENSGACG00000000890" "ENSGACG00000000877"
##  [1513] "ENSGACG00000000874" "ENSGACG00000019362" "ENSGACG00000000870"
##  [1516] "ENSGACG00000000867" "ENSGACG00000000862" "ENSGACG00000025461"
##  [1519] "ENSGACG00000000853" "ENSGACG00000019366" "ENSGACG00000000852"
##  [1522] "ENSGACG00000019370" "ENSGACG00000000850" "ENSGACG00000000848"
##  [1525] "ENSGACG00000019377" "ENSGACG00000000846" "ENSGACG00000033717"
##  [1528] "ENSGACG00000019378" "ENSGACG00000000827" "ENSGACG00000019384"
##  [1531] "ENSGACG00000000822" "ENSGACG00000000821" "ENSGACG00000000819"
##  [1534] "ENSGACG00000000817" "ENSGACG00000000812" "ENSGACG00000024374"
##  [1537] "ENSGACG00000000809" "ENSGACG00000000804" "ENSGACG00000000803"
##  [1540] "ENSGACG00000019393" "ENSGACG00000019396" "ENSGACG00000019398"
##  [1543] "ENSGACG00000019400" "ENSGACG00000019402" "ENSGACG00000029168"
##  [1546] "ENSGACG00000000799" "ENSGACG00000019405" "ENSGACG00000019408"
##  [1549] "ENSGACG00000019409" "ENSGACG00000034087" "ENSGACG00000019411"
##  [1552] "ENSGACG00000019414" "ENSGACG00000027523" "ENSGACG00000019314"
##  [1555] "ENSGACG00000000790" "ENSGACG00000019316" "ENSGACG00000000789"
##  [1558] "ENSGACG00000019318" "ENSGACG00000000782" "ENSGACG00000035192"
##  [1561] "ENSGACG00000023489" "ENSGACG00000025419" "ENSGACG00000028745"
##  [1564] "ENSGACG00000000770" "ENSGACG00000000768" "ENSGACG00000000759"
##  [1567] "ENSGACG00000037351" "ENSGACG00000027699" "ENSGACG00000020241"
##  [1570] "ENSGACG00000030697" "ENSGACG00000020243" "ENSGACG00000020245"
##  [1573] "ENSGACG00000020246" "ENSGACG00000020248" "ENSGACG00000020249"
##  [1576] "ENSGACG00000020250" "ENSGACG00000019886" "ENSGACG00000019888"
##  [1579] "ENSGACG00000030411" "ENSGACG00000020251" "ENSGACG00000024817"
##  [1582] "ENSGACG00000031027" "ENSGACG00000020253" "ENSGACG00000019834"
##  [1585] "ENSGACG00000019840" "ENSGACG00000019844" "ENSGACG00000019847"
##  [1588] "ENSGACG00000020254" "ENSGACG00000036361" "ENSGACG00000029005"
##  [1591] "ENSGACG00000019861" "ENSGACG00000020256" "ENSGACG00000020257"
##  [1594] "ENSGACG00000037552" "ENSGACG00000027216" "ENSGACG00000026391"
##  [1597] "ENSGACG00000020261" "ENSGACG00000020262" "ENSGACG00000023592"
##  [1600] "ENSGACG00000020264" "ENSGACG00000020265" "ENSGACG00000019824"
##  [1603] "ENSGACG00000019828" "ENSGACG00000034813" "ENSGACG00000030216"
##  [1606] "ENSGACG00000020593" "ENSGACG00000034356" "ENSGACG00000020594"
##  [1609] "ENSGACG00000019803" "ENSGACG00000036681" "ENSGACG00000029120"
##  [1612] "ENSGACG00000020595" "ENSGACG00000020597" "ENSGACG00000020598"
##  [1615] "ENSGACG00000023736" "ENSGACG00000020600" "ENSGACG00000034069"
##  [1618] "ENSGACG00000023479" "ENSGACG00000030892" "ENSGACG00000019745"
##  [1621] "ENSGACG00000019747" "ENSGACG00000020601" "ENSGACG00000023569"
##  [1624] "ENSGACG00000035406" "ENSGACG00000020602" "ENSGACG00000019753"
##  [1627] "ENSGACG00000029495" "ENSGACG00000019760" "ENSGACG00000020603"
##  [1630] "ENSGACG00000020605" "ENSGACG00000020604" "ENSGACG00000020606"
##  [1633] "ENSGACG00000019308" "ENSGACG00000020607" "ENSGACG00000020395"
##  [1636] "ENSGACG00000020396" "ENSGACG00000019764" "ENSGACG00000020397"
##  [1639] "ENSGACG00000020266" "ENSGACG00000020269" "ENSGACG00000020270"
##  [1642] "ENSGACG00000020271" "ENSGACG00000020398" "ENSGACG00000034906"
##  [1645] "ENSGACG00000020272" "ENSGACG00000033003" "ENSGACG00000019770"
##  [1648] "ENSGACG00000020273" "ENSGACG00000029139" "ENSGACG00000019794"
##  [1651] "ENSGACG00000035474" "ENSGACG00000019902" "ENSGACG00000033102"
##  [1654] "ENSGACG00000020275" "ENSGACG00000020277" "ENSGACG00000020278"
##  [1657] "ENSGACG00000020279" "ENSGACG00000020280" "ENSGACG00000034349"
##  [1660] "ENSGACG00000020282" "ENSGACG00000020284" "ENSGACG00000020285"
##  [1663] "ENSGACG00000020286" "ENSGACG00000020287" "ENSGACG00000034849"
##  [1666] "ENSGACG00000020288" "ENSGACG00000019913" "ENSGACG00000020289"
##  [1669] "ENSGACG00000020290" "ENSGACG00000020291" "ENSGACG00000020292"
##  [1672] "ENSGACG00000019917" "ENSGACG00000035738" "ENSGACG00000020293"
##  [1675] "ENSGACG00000019922" "ENSGACG00000019924" "ENSGACG00000020294"
##  [1678] "ENSGACG00000019925" "ENSGACG00000020295" "ENSGACG00000020296"
##  [1681] "ENSGACG00000027918" "ENSGACG00000019927" "ENSGACG00000020399"
##  [1684] "ENSGACG00000029526" "ENSGACG00000020298" "ENSGACG00000033991"
##  [1687] "ENSGACG00000020299" "ENSGACG00000020400" "ENSGACG00000020300"
##  [1690] "ENSGACG00000019931" "ENSGACG00000023227" "ENSGACG00000033134"
##  [1693] "ENSGACG00000020301" "ENSGACG00000020402" "ENSGACG00000030752"
##  [1696] "ENSGACG00000020403" "ENSGACG00000019935" "ENSGACG00000020404"
##  [1699] "ENSGACG00000026227" "ENSGACG00000020308" "ENSGACG00000019937"
##  [1702] "ENSGACG00000030363" "ENSGACG00000019943" "ENSGACG00000019946"
##  [1705] "ENSGACG00000031454" "ENSGACG00000019961" "ENSGACG00000037958"
##  [1708] "ENSGACG00000020303" "ENSGACG00000020304" "ENSGACG00000023160"
##  [1711] "ENSGACG00000020309" "ENSGACG00000026847" "ENSGACG00000022986"
##  [1714] "ENSGACG00000020311" "ENSGACG00000037502" "ENSGACG00000019980"
##  [1717] "ENSGACG00000019983" "ENSGACG00000019989" "ENSGACG00000019991"
##  [1720] "ENSGACG00000020306" "ENSGACG00000020151" "ENSGACG00000020152"
##  [1723] "ENSGACG00000020153" "ENSGACG00000020695" "ENSGACG00000028757"
##  [1726] "ENSGACG00000020155" "ENSGACG00000020405" "ENSGACG00000020406"
##  [1729] "ENSGACG00000020001" "ENSGACG00000020407" "ENSGACG00000020003"
##  [1732] "ENSGACG00000020005" "ENSGACG00000020408" "ENSGACG00000020006"
##  [1735] "ENSGACG00000020409" "ENSGACG00000025080" "ENSGACG00000020163"
##  [1738] "ENSGACG00000020156" "ENSGACG00000027037" "ENSGACG00000020158"
##  [1741] "ENSGACG00000020159" "ENSGACG00000032993" "ENSGACG00000020160"
##  [1744] "ENSGACG00000020161" "ENSGACG00000020162" "ENSGACG00000020164"
##  [1747] "ENSGACG00000035696" "ENSGACG00000032425" "ENSGACG00000020411"
##  [1750] "ENSGACG00000020412" "ENSGACG00000020166" "ENSGACG00000020413"
##  [1753] "ENSGACG00000031017" "ENSGACG00000020016" "ENSGACG00000020018"
##  [1756] "ENSGACG00000037297" "ENSGACG00000020022" "ENSGACG00000020024"
##  [1759] "ENSGACG00000020415" "ENSGACG00000020168" "ENSGACG00000023228"
##  [1762] "ENSGACG00000036712" "ENSGACG00000020170" "ENSGACG00000020027"
##  [1765] "ENSGACG00000020169" "ENSGACG00000037264" "ENSGACG00000034820"
##  [1768] "ENSGACG00000034035" "ENSGACG00000020418" "ENSGACG00000019418"
##  [1771] "ENSGACG00000020419" "ENSGACG00000020420" "ENSGACG00000019423"
##  [1774] "ENSGACG00000019425" "ENSGACG00000019426" "ENSGACG00000034600"
##  [1777] "ENSGACG00000019429" "ENSGACG00000031892" "ENSGACG00000020421"
##  [1780] "ENSGACG00000020422" "ENSGACG00000024453" "ENSGACG00000020423"
##  [1783] "ENSGACG00000020424" "ENSGACG00000020425" "ENSGACG00000026392"
##  [1786] "ENSGACG00000019430" "ENSGACG00000020426" "ENSGACG00000033926"
##  [1789] "ENSGACG00000019435" "ENSGACG00000019438" "ENSGACG00000019443"
##  [1792] "ENSGACG00000023571" "ENSGACG00000020429" "ENSGACG00000019458"
##  [1795] "ENSGACG00000020430" "ENSGACG00000019464" "ENSGACG00000036660"
##  [1798] "ENSGACG00000031158" "ENSGACG00000020172" "ENSGACG00000019471"
##  [1801] "ENSGACG00000020432" "ENSGACG00000020173" "ENSGACG00000019474"
##  [1804] "ENSGACG00000020174" "ENSGACG00000020433" "ENSGACG00000026005"
##  [1807] "ENSGACG00000031414" "ENSGACG00000028459" "ENSGACG00000019484"
##  [1810] "ENSGACG00000020176" "ENSGACG00000023986" "ENSGACG00000023753"
##  [1813] "ENSGACG00000020180" "ENSGACG00000020434" "ENSGACG00000020435"
##  [1816] "ENSGACG00000019489" "ENSGACG00000020436" "ENSGACG00000020608"
##  [1819] "ENSGACG00000019493" "ENSGACG00000035895" "ENSGACG00000020609"
##  [1822] "ENSGACG00000020439" "ENSGACG00000020610" "ENSGACG00000020612"
##  [1825] "ENSGACG00000020440" "ENSGACG00000020615" "ENSGACG00000028803"
##  [1828] "ENSGACG00000020441" "ENSGACG00000033336" "ENSGACG00000020443"
##  [1831] "ENSGACG00000020444" "ENSGACG00000032320" "ENSGACG00000023960"
##  [1834] "ENSGACG00000033503" "ENSGACG00000020617" "ENSGACG00000020618"
##  [1837] "ENSGACG00000020619" "ENSGACG00000020620" "ENSGACG00000025060"
##  [1840] "ENSGACG00000020183" "ENSGACG00000020185" "ENSGACG00000019498"
##  [1843] "ENSGACG00000028770" "ENSGACG00000034799" "ENSGACG00000020622"
##  [1846] "ENSGACG00000020623" "ENSGACG00000020624" "ENSGACG00000020625"
##  [1849] "ENSGACG00000020626" "ENSGACG00000020186" "ENSGACG00000020627"
##  [1852] "ENSGACG00000020628" "ENSGACG00000020629" "ENSGACG00000020187"
##  [1855] "ENSGACG00000020630" "ENSGACG00000020188" "ENSGACG00000020631"
##  [1858] "ENSGACG00000020632" "ENSGACG00000020634" "ENSGACG00000020633"
##  [1861] "ENSGACG00000020635" "ENSGACG00000020636" "ENSGACG00000026477"
##  [1864] "ENSGACG00000020638" "ENSGACG00000020639" "ENSGACG00000020640"
##  [1867] "ENSGACG00000020641" "ENSGACG00000020642" "ENSGACG00000037553"
##  [1870] "ENSGACG00000020644" "ENSGACG00000029954" "ENSGACG00000020189"
##  [1873] "ENSGACG00000020190" "ENSGACG00000020191" "ENSGACG00000030831"
##  [1876] "ENSGACG00000020192" "ENSGACG00000020647" "ENSGACG00000020649"
##  [1879] "ENSGACG00000020650" "ENSGACG00000020651" "ENSGACG00000023301"
##  [1882] "ENSGACG00000020193" "ENSGACG00000029270" "ENSGACG00000020194"
##  [1885] "ENSGACG00000020195" "ENSGACG00000020196" "ENSGACG00000020197"
##  [1888] "ENSGACG00000020198" "ENSGACG00000020199" "ENSGACG00000020200"
##  [1891] "ENSGACG00000020201" "ENSGACG00000020202" "ENSGACG00000020203"
##  [1894] "ENSGACG00000020204" "ENSGACG00000020205" "ENSGACG00000020206"
##  [1897] "ENSGACG00000019499" "ENSGACG00000020207" "ENSGACG00000020208"
##  [1900] "ENSGACG00000020209" "ENSGACG00000020210" "ENSGACG00000037422"
##  [1903] "ENSGACG00000020653" "ENSGACG00000020654" "ENSGACG00000035045"
##  [1906] "ENSGACG00000020658" "ENSGACG00000020313" "ENSGACG00000020314"
##  [1909] "ENSGACG00000030686" "ENSGACG00000020315" "ENSGACG00000026069"
##  [1912] "ENSGACG00000020317" "ENSGACG00000020318" "ENSGACG00000020319"
##  [1915] "ENSGACG00000020320" "ENSGACG00000037598" "ENSGACG00000023291"
##  [1918] "ENSGACG00000019503" "ENSGACG00000020322" "ENSGACG00000019528"
##  [1921] "ENSGACG00000020323" "ENSGACG00000026075" "ENSGACG00000020326"
##  [1924] "ENSGACG00000020327" "ENSGACG00000020328" "ENSGACG00000020330"
##  [1927] "ENSGACG00000020331" "ENSGACG00000020333" "ENSGACG00000020334"
##  [1930] "ENSGACG00000020335" "ENSGACG00000020336" "ENSGACG00000020337"
##  [1933] "ENSGACG00000020659" "ENSGACG00000020338" "ENSGACG00000020660"
##  [1936] "ENSGACG00000031613" "ENSGACG00000020661" "ENSGACG00000020662"
##  [1939] "ENSGACG00000020339" "ENSGACG00000020663" "ENSGACG00000020563"
##  [1942] "ENSGACG00000020564" "ENSGACG00000020565" "ENSGACG00000020665"
##  [1945] "ENSGACG00000025604" "ENSGACG00000020341" "ENSGACG00000020569"
##  [1948] "ENSGACG00000020342" "ENSGACG00000037812" "ENSGACG00000020344"
##  [1951] "ENSGACG00000020345" "ENSGACG00000020666" "ENSGACG00000020667"
##  [1954] "ENSGACG00000020570" "ENSGACG00000020668" "ENSGACG00000020571"
##  [1957] "ENSGACG00000020346" "ENSGACG00000020347" "ENSGACG00000034616"
##  [1960] "ENSGACG00000020669" "ENSGACG00000020671" "ENSGACG00000026875"
##  [1963] "ENSGACG00000020349" "ENSGACG00000020350" "ENSGACG00000020351"
##  [1966] "ENSGACG00000020352" "ENSGACG00000020353" "ENSGACG00000020673"
##  [1969] "ENSGACG00000020674" "ENSGACG00000020354" "ENSGACG00000035574"
##  [1972] "ENSGACG00000034712" "ENSGACG00000020677" "ENSGACG00000020356"
##  [1975] "ENSGACG00000033281" "ENSGACG00000020358" "ENSGACG00000027933"
##  [1978] "ENSGACG00000035425" "ENSGACG00000020365" "ENSGACG00000020362"
##  [1981] "ENSGACG00000032542" "ENSGACG00000020575" "ENSGACG00000020572"
##  [1984] "ENSGACG00000020573" "ENSGACG00000020574" "ENSGACG00000020576"
##  [1987] "ENSGACG00000020577" "ENSGACG00000020578" "ENSGACG00000020579"
##  [1990] "ENSGACG00000037915" "ENSGACG00000020580" "ENSGACG00000020363"
##  [1993] "ENSGACG00000020581" "ENSGACG00000020364" "ENSGACG00000020582"
##  [1996] "ENSGACG00000020678" "ENSGACG00000030716" "ENSGACG00000020583"
##  [1999] "ENSGACG00000020030" "ENSGACG00000038083" "ENSGACG00000020680"
##  [2002] "ENSGACG00000020584" "ENSGACG00000030662" "ENSGACG00000027736"
##  [2005] "ENSGACG00000020585" "ENSGACG00000020586" "ENSGACG00000020587"
##  [2008] "ENSGACG00000020588" "ENSGACG00000020589" "ENSGACG00000027832"
##  [2011] "ENSGACG00000020038" "ENSGACG00000020035" "ENSGACG00000020039"
##  [2014] "ENSGACG00000020214" "ENSGACG00000020215" "ENSGACG00000036496"
##  [2017] "ENSGACG00000020216" "ENSGACG00000028129" "ENSGACG00000030445"
##  [2020] "ENSGACG00000020451" "ENSGACG00000024992" "ENSGACG00000024672"
##  [2023] "ENSGACG00000020452" "ENSGACG00000020218" "ENSGACG00000020219"
##  [2026] "ENSGACG00000020220" "ENSGACG00000029262" "ENSGACG00000020222"
##  [2029] "ENSGACG00000027664" "ENSGACG00000020713" "ENSGACG00000020223"
##  [2032] "ENSGACG00000020224" "ENSGACG00000033240" "ENSGACG00000020703"
##  [2035] "ENSGACG00000036000" "ENSGACG00000020225" "ENSGACG00000020226"
##  [2038] "ENSGACG00000019533" "ENSGACG00000019535" "ENSGACG00000029362"
##  [2041] "ENSGACG00000019537" "ENSGACG00000020704" "ENSGACG00000019541"
##  [2044] "ENSGACG00000020705" "ENSGACG00000020706" "ENSGACG00000020707"
##  [2047] "ENSGACG00000020227" "ENSGACG00000019543" "ENSGACG00000020708"
##  [2050] "ENSGACG00000020709" "ENSGACG00000019544" "ENSGACG00000020710"
##  [2053] "ENSGACG00000020228" "ENSGACG00000019546" "ENSGACG00000019549"
##  [2056] "ENSGACG00000020229" "ENSGACG00000020230" "ENSGACG00000020231"
##  [2059] "ENSGACG00000020711" "ENSGACG00000029958" "ENSGACG00000020233"
##  [2062] "ENSGACG00000032225" "ENSGACG00000020235" "ENSGACG00000019569"
##  [2065] "ENSGACG00000024017" "ENSGACG00000020237" "ENSGACG00000029117"
##  [2068] "ENSGACG00000019574" "ENSGACG00000037193" "ENSGACG00000019587"
##  [2071] "ENSGACG00000019590" "ENSGACG00000020681" "ENSGACG00000020682"
##  [2074] "ENSGACG00000019593" "ENSGACG00000020683" "ENSGACG00000019601"
##  [2077] "ENSGACG00000020369" "ENSGACG00000019605" "ENSGACG00000020370"
##  [2080] "ENSGACG00000029244" "ENSGACG00000020239" "ENSGACG00000020371"
##  [2083] "ENSGACG00000020374" "ENSGACG00000019618" "ENSGACG00000019629"
##  [2086] "ENSGACG00000020375" "ENSGACG00000019638" "ENSGACG00000019649"
##  [2089] "ENSGACG00000019650" "ENSGACG00000034032" "ENSGACG00000000728"
##  [2092] "ENSGACG00000019655" "ENSGACG00000024984" "ENSGACG00000019658"
##  [2095] "ENSGACG00000000726" "ENSGACG00000000721" "ENSGACG00000000715"
##  [2098] "ENSGACG00000019672" "ENSGACG00000000704" "ENSGACG00000019677"
##  [2101] "ENSGACG00000000701" "ENSGACG00000023267" "ENSGACG00000034729"
##  [2104] "ENSGACG00000027110" "ENSGACG00000020088" "ENSGACG00000020376"
##  [2107] "ENSGACG00000020377" "ENSGACG00000019686" "ENSGACG00000020095"
##  [2110] "ENSGACG00000019688" "ENSGACG00000029641" "ENSGACG00000020378"
##  [2113] "ENSGACG00000020096" "ENSGACG00000020097" "ENSGACG00000037136"
##  [2116] "ENSGACG00000023369" "ENSGACG00000020381" "ENSGACG00000034470"
##  [2119] "ENSGACG00000020382" "ENSGACG00000020383" "ENSGACG00000020105"
##  [2122] "ENSGACG00000027992" "ENSGACG00000020385" "ENSGACG00000024762"
##  [2125] "ENSGACG00000020386" "ENSGACG00000020388" "ENSGACG00000027041"
##  [2128] "ENSGACG00000020390" "ENSGACG00000020392" "ENSGACG00000033920"
##  [2131] "ENSGACG00000020393" "ENSGACG00000020106" "ENSGACG00000020108"
##  [2134] "ENSGACG00000020394" "ENSGACG00000036515" "ENSGACG00000019701"
##  [2137] "ENSGACG00000025654" "ENSGACG00000019710" "ENSGACG00000033377"
##  [2140] "ENSGACG00000019319" "ENSGACG00000031802" "ENSGACG00000019324"
##  [2143] "ENSGACG00000020567" "ENSGACG00000019329" "ENSGACG00000024495"
##  [2146] "ENSGACG00000037566" "ENSGACG00000020568" "ENSGACG00000001192"
##  [2149] "ENSGACG00000029703" "ENSGACG00000020684" "ENSGACG00000001189"
##  [2152] "ENSGACG00000024599" "ENSGACG00000001181" "ENSGACG00000020689"
##  [2155] "ENSGACG00000020690" "ENSGACG00000032336" "ENSGACG00000024588"
##  [2158] "ENSGACG00000020693" "ENSGACG00000020694" "ENSGACG00000029491"
##  [2161] "ENSGACG00000027632" "ENSGACG00000001175" "ENSGACG00000001173"
##  [2164] "ENSGACG00000025147" "ENSGACG00000001165" "ENSGACG00000001161"
##  [2167] "ENSGACG00000027093" "ENSGACG00000020111" "ENSGACG00000020112"
##  [2170] "ENSGACG00000020113" "ENSGACG00000020446" "ENSGACG00000020447"
##  [2173] "ENSGACG00000020449" "ENSGACG00000020117" "ENSGACG00000020450"
##  [2176] "ENSGACG00000020366" "ENSGACG00000033410" "ENSGACG00000020119"
##  [2179] "ENSGACG00000020120" "ENSGACG00000020121" "ENSGACG00000020123"
##  [2182] "ENSGACG00000033088" "ENSGACG00000020124" "ENSGACG00000027621"
##  [2185] "ENSGACG00000020126" "ENSGACG00000020127" "ENSGACG00000028557"
##  [2188] "ENSGACG00000020128" "ENSGACG00000020129" "ENSGACG00000001158"
##  [2191] "ENSGACG00000001154" "ENSGACG00000001147" "ENSGACG00000001144"
##  [2194] "ENSGACG00000001141" "ENSGACG00000034071" "ENSGACG00000035892"
##  [2197] "ENSGACG00000020135" "ENSGACG00000037431" "ENSGACG00000001133"
##  [2200] "ENSGACG00000035195" "ENSGACG00000020139" "ENSGACG00000001131"
##  [2203] "ENSGACG00000001125" "ENSGACG00000020145" "ENSGACG00000027246"
##  [2206] "ENSGACG00000024168" "ENSGACG00000020696" "ENSGACG00000027646"
##  [2209] "ENSGACG00000029011" "ENSGACG00000036440" "ENSGACG00000029161"
##  [2212] "ENSGACG00000001113" "ENSGACG00000031985" "ENSGACG00000024881"
##  [2215] "ENSGACG00000025800" "ENSGACG00000031868" "ENSGACG00000036565"
##  [2218] "ENSGACG00000032552" "ENSGACG00000032361" "ENSGACG00000031972"
##  [2221] "ENSGACG00000001106" "ENSGACG00000037658" "ENSGACG00000033230"
##  [2224] "ENSGACG00000027639" "ENSGACG00000030487" "ENSGACG00000032650"
##  [2227] "ENSGACG00000020698" "ENSGACG00000025623" "ENSGACG00000025949"
##  [2230] "ENSGACG00000029269" "ENSGACG00000033063" "ENSGACG00000029407"
##  [2233] "ENSGACG00000036239" "ENSGACG00000028493" "ENSGACG00000030641"
##  [2236] "ENSGACG00000035128" "ENSGACG00000033981" "ENSGACG00000029776"
##  [2239] "ENSGACG00000029459" "ENSGACG00000036619" "ENSGACG00000036175"
##  [2242] "ENSGACG00000026090" "ENSGACG00000026364" "ENSGACG00000024626"
##  [2245] "ENSGACG00000026101" "ENSGACG00000034508" "ENSGACG00000032714"
##  [2248] "ENSGACG00000036710" "ENSGACG00000031171" "ENSGACG00000030357"
##  [2251] "ENSGACG00000037410" "ENSGACG00000031211" "ENSGACG00000031246"
##  [2254] "ENSGACG00000033952" "ENSGACG00000032106" "ENSGACG00000030073"
##  [2257] "ENSGACG00000030106" "ENSGACG00000031269" "ENSGACG00000034309"
##  [2260] "ENSGACG00000029274" "ENSGACG00000036233" "ENSGACG00000033807"
##  [2263] "ENSGACG00000034023" "ENSGACG00000030346" "ENSGACG00000026941"
##  [2266] "ENSGACG00000037394" "ENSGACG00000025591" "ENSGACG00000001606"
##  [2269] "ENSGACG00000023724" "ENSGACG00000025029" "ENSGACG00000020777"
##  [2272] "ENSGACG00000020778" "ENSGACG00000020779" "ENSGACG00000036932"
##  [2275] "ENSGACG00000024784" "ENSGACG00000020781" "ENSGACG00000005434"
##  [2278] "ENSGACG00000000969" "ENSGACG00000000826" "ENSGACG00000020783"
##  [2281] "ENSGACG00000020244" "ENSGACG00000020247" "ENSGACG00000020263"
##  [2284] "ENSGACG00000020305" "ENSGACG00000028948" "ENSGACG00000001100"
##  [2287] "ENSGACG00000000304" "ENSGACG00000000314" "ENSGACG00000020332"
##  [2290] "ENSGACG00000025719" "ENSGACG00000020784" "ENSGACG00000020785"
##  [2293] "ENSGACG00000024244" "ENSGACG00000000320" "ENSGACG00000034331"
##  [2296] "ENSGACG00000035840" "ENSGACG00000000330" "ENSGACG00000017967"
##  [2299] "ENSGACG00000000356" "ENSGACG00000033294" "ENSGACG00000026874"
##  [2302] "ENSGACG00000036303" "ENSGACG00000020786" "ENSGACG00000000360"
##  [2305] "ENSGACG00000000362" "ENSGACG00000000368" "ENSGACG00000020787"
##  [2308] "ENSGACG00000020788" "ENSGACG00000028144" "ENSGACG00000000373"
##  [2311] "ENSGACG00000000377" "ENSGACG00000035014" "ENSGACG00000034959"
##  [2314] "ENSGACG00000000380" "ENSGACG00000031161" "ENSGACG00000020791"
##  [2317] "ENSGACG00000020361" "ENSGACG00000020236" "ENSGACG00000020793"
##  [2320] "ENSGACG00000020101" "ENSGACG00000020794" "ENSGACG00000023531"
##  [2323] "ENSGACG00000020795" "ENSGACG00000037505" "ENSGACG00000020367"
##  [2326] "ENSGACG00000018669" "ENSGACG00000018668" "ENSGACG00000018660"
##  [2329] "ENSGACG00000018658" "ENSGACG00000020797" "ENSGACG00000026010"
##  [2332] "ENSGACG00000018652" "ENSGACG00000024041" "ENSGACG00000030802"
##  [2335] "ENSGACG00000018640" "ENSGACG00000018638" "ENSGACG00000018633"
##  [2338] "ENSGACG00000018630" "ENSGACG00000018629" "ENSGACG00000020799"
##  [2341] "ENSGACG00000036937" "ENSGACG00000018625" "ENSGACG00000018618"
##  [2344] "ENSGACG00000024463" "ENSGACG00000032428" "ENSGACG00000035473"
##  [2347] "ENSGACG00000018611" "ENSGACG00000032932" "ENSGACG00000018606"
##  [2350] "ENSGACG00000032971" "ENSGACG00000018600" "ENSGACG00000018596"
##  [2353] "ENSGACG00000018594" "ENSGACG00000030827" "ENSGACG00000018590"
##  [2356] "ENSGACG00000018588" "ENSGACG00000030035" "ENSGACG00000018577"
##  [2359] "ENSGACG00000018567" "ENSGACG00000018565" "ENSGACG00000033677"
##  [2362] "ENSGACG00000027816" "ENSGACG00000018560" "ENSGACG00000018555"
##  [2365] "ENSGACG00000018553" "ENSGACG00000018546" "ENSGACG00000026553"
##  [2368] "ENSGACG00000018544" "ENSGACG00000018697" "ENSGACG00000024879"
##  [2371] "ENSGACG00000018702" "ENSGACG00000026280" "ENSGACG00000018704"
##  [2374] "ENSGACG00000018711" "ENSGACG00000018715" "ENSGACG00000028252"
##  [2377] "ENSGACG00000025065" "ENSGACG00000018720" "ENSGACG00000018737"
##  [2380] "ENSGACG00000018743" "ENSGACG00000037622" "ENSGACG00000035485"
##  [2383] "ENSGACG00000025056" "ENSGACG00000018753" "ENSGACG00000031870"
##  [2386] "ENSGACG00000018761" "ENSGACG00000018764" "ENSGACG00000035476"
##  [2389] "ENSGACG00000027253" "ENSGACG00000018775" "ENSGACG00000018781"
##  [2392] "ENSGACG00000018784" "ENSGACG00000018786" "ENSGACG00000018788"
##  [2395] "ENSGACG00000018793" "ENSGACG00000018796" "ENSGACG00000036002"
##  [2398] "ENSGACG00000018805" "ENSGACG00000020368" "ENSGACG00000018808"
##  [2401] "ENSGACG00000018809" "ENSGACG00000018813" "ENSGACG00000026892"
##  [2404] "ENSGACG00000027126" "ENSGACG00000035061" "ENSGACG00000018825"
##  [2407] "ENSGACG00000036902" "ENSGACG00000018830" "ENSGACG00000018855"
##  [2410] "ENSGACG00000018866" "ENSGACG00000030068" "ENSGACG00000018870"
##  [2413] "ENSGACG00000018872" "ENSGACG00000018876" "ENSGACG00000020800"
##  [2416] "ENSGACG00000018883" "ENSGACG00000030980" "ENSGACG00000034260"
##  [2419] "ENSGACG00000020803" "ENSGACG00000018893" "ENSGACG00000025911"
##  [2422] "ENSGACG00000020805" "ENSGACG00000028209" "ENSGACG00000020808"
##  [2425] "ENSGACG00000018896" "ENSGACG00000018897" "ENSGACG00000025209"
##  [2428] "ENSGACG00000018902" "ENSGACG00000035222" "ENSGACG00000018904"
##  [2431] "ENSGACG00000037302" "ENSGACG00000020810" "ENSGACG00000028585"
##  [2434] "ENSGACG00000020812" "ENSGACG00000018908" "ENSGACG00000018909"
##  [2437] "ENSGACG00000020813" "ENSGACG00000018913" "ENSGACG00000023529"
##  [2440] "ENSGACG00000018916" "ENSGACG00000020814" "ENSGACG00000020545"
##  [2443] "ENSGACG00000031078" "ENSGACG00000018921" "ENSGACG00000037703"
##  [2446] "ENSGACG00000027961" "ENSGACG00000020549" "ENSGACG00000018924"
##  [2449] "ENSGACG00000020550" "ENSGACG00000037550" "ENSGACG00000018931"
##  [2452] "ENSGACG00000020551" "ENSGACG00000018932" "ENSGACG00000028671"
##  [2455] "ENSGACG00000038131" "ENSGACG00000031744" "ENSGACG00000034323"
##  [2458] "ENSGACG00000020552" "ENSGACG00000020553" "ENSGACG00000020554"
##  [2461] "ENSGACG00000020555" "ENSGACG00000020556" "ENSGACG00000018954"
##  [2464] "ENSGACG00000018963" "ENSGACG00000018970" "ENSGACG00000018971"
##  [2467] "ENSGACG00000018972" "ENSGACG00000018979" "ENSGACG00000018976"
##  [2470] "ENSGACG00000018980" "ENSGACG00000018982" "ENSGACG00000036995"
##  [2473] "ENSGACG00000034588" "ENSGACG00000026288" "ENSGACG00000034569"
##  [2476] "ENSGACG00000024117" "ENSGACG00000018986" "ENSGACG00000018988"
##  [2479] "ENSGACG00000018991" "ENSGACG00000020558" "ENSGACG00000020559"
##  [2482] "ENSGACG00000036374" "ENSGACG00000020560" "ENSGACG00000037845"
##  [2485] "ENSGACG00000020562" "ENSGACG00000032447" "ENSGACG00000023206"
##  [2488] "ENSGACG00000026370" "ENSGACG00000020717" "ENSGACG00000029843"
##  [2491] "ENSGACG00000019012" "ENSGACG00000020718" "ENSGACG00000020719"
##  [2494] "ENSGACG00000019019" "ENSGACG00000019021" "ENSGACG00000020720"
##  [2497] "ENSGACG00000037432" "ENSGACG00000019023" "ENSGACG00000020722"
##  [2500] "ENSGACG00000020723" "ENSGACG00000020724" "ENSGACG00000020725"
##  [2503] "ENSGACG00000020726" "ENSGACG00000026531" "ENSGACG00000020728"
##  [2506] "ENSGACG00000020729" "ENSGACG00000020731" "ENSGACG00000020732"
##  [2509] "ENSGACG00000025715" "ENSGACG00000020734" "ENSGACG00000028465"
##  [2512] "ENSGACG00000024436" "ENSGACG00000020737" "ENSGACG00000026028"
##  [2515] "ENSGACG00000020739" "ENSGACG00000020741" "ENSGACG00000020742"
##  [2518] "ENSGACG00000020743" "ENSGACG00000020744" "ENSGACG00000020745"
##  [2521] "ENSGACG00000037533" "ENSGACG00000019029" "ENSGACG00000032137"
##  [2524] "ENSGACG00000024208" "ENSGACG00000019031" "ENSGACG00000019038"
##  [2527] "ENSGACG00000019040" "ENSGACG00000019042" "ENSGACG00000019044"
##  [2530] "ENSGACG00000020746" "ENSGACG00000019049" "ENSGACG00000020747"
##  [2533] "ENSGACG00000019052" "ENSGACG00000019055" "ENSGACG00000019057"
##  [2536] "ENSGACG00000019058" "ENSGACG00000019069" "ENSGACG00000019072"
##  [2539] "ENSGACG00000019076" "ENSGACG00000019078" "ENSGACG00000032788"
##  [2542] "ENSGACG00000019085" "ENSGACG00000025766" "ENSGACG00000030388"
##  [2545] "ENSGACG00000030065" "ENSGACG00000028486" "ENSGACG00000019096"
##  [2548] "ENSGACG00000019099" "ENSGACG00000019101" "ENSGACG00000019111"
##  [2551] "ENSGACG00000023290" "ENSGACG00000019119" "ENSGACG00000019124"
##  [2554] "ENSGACG00000029614" "ENSGACG00000026379" "ENSGACG00000031000"
##  [2557] "ENSGACG00000019138" "ENSGACG00000019137" "ENSGACG00000019142"
##  [2560] "ENSGACG00000019152" "ENSGACG00000019161" "ENSGACG00000034367"
##  [2563] "ENSGACG00000019167" "ENSGACG00000023146" "ENSGACG00000031917"
##  [2566] "ENSGACG00000023422" "ENSGACG00000019180" "ENSGACG00000019194"
##  [2569] "ENSGACG00000019196" "ENSGACG00000020748" "ENSGACG00000033877"
##  [2572] "ENSGACG00000020749" "ENSGACG00000033482" "ENSGACG00000019199"
##  [2575] "ENSGACG00000017731" "ENSGACG00000019201" "ENSGACG00000028328"
##  [2578] "ENSGACG00000037259" "ENSGACG00000019205" "ENSGACG00000037473"
##  [2581] "ENSGACG00000023863" "ENSGACG00000026583" "ENSGACG00000019211"
##  [2584] "ENSGACG00000019213" "ENSGACG00000020751" "ENSGACG00000020752"
##  [2587] "ENSGACG00000023155" "ENSGACG00000020754" "ENSGACG00000020755"
##  [2590] "ENSGACG00000019232" "ENSGACG00000023564" "ENSGACG00000033481"
##  [2593] "ENSGACG00000019234" "ENSGACG00000020757" "ENSGACG00000019244"
##  [2596] "ENSGACG00000020758" "ENSGACG00000019250" "ENSGACG00000019258"
##  [2599] "ENSGACG00000020759" "ENSGACG00000019261" "ENSGACG00000019265"
##  [2602] "ENSGACG00000019273" "ENSGACG00000019275" "ENSGACG00000020760"
##  [2605] "ENSGACG00000029311" "ENSGACG00000020761" "ENSGACG00000020762"
##  [2608] "ENSGACG00000020763" "ENSGACG00000027425" "ENSGACG00000020765"
##  [2611] "ENSGACG00000020766" "ENSGACG00000036266" "ENSGACG00000020768"
##  [2614] "ENSGACG00000020769" "ENSGACG00000020770" "ENSGACG00000020771"
##  [2617] "ENSGACG00000020773" "ENSGACG00000033047" "ENSGACG00000027867"
##  [2620] "ENSGACG00000019286" "ENSGACG00000019289" "ENSGACG00000037796"
##  [2623] "ENSGACG00000035975" "ENSGACG00000019294" "ENSGACG00000019295"
##  [2626] "ENSGACG00000019297" "ENSGACG00000019299" "ENSGACG00000026579"
##  [2629] "ENSGACG00000019301" "ENSGACG00000019305" "ENSGACG00000028897"
##  [2632] "ENSGACG00000029125" "ENSGACG00000034647" "ENSGACG00000029214"
##  [2635] "ENSGACG00000023854" "ENSGACG00000031023" "ENSGACG00000000379"
##  [2638] "ENSGACG00000033839" "ENSGACG00000029834" "ENSGACG00000030743"
##  [2641] "ENSGACG00000025425" "ENSGACG00000037133" "ENSGACG00000029589"
##  [2644] "ENSGACG00000035269" "ENSGACG00000032523" "ENSGACG00000031189"
##  [2647] "ENSGACG00000029912" "ENSGACG00000034498" "ENSGACG00000035441"
##  [2650] "ENSGACG00000030371" "ENSGACG00000023404" "ENSGACG00000029470"
##  [2653] "ENSGACG00000037950" "ENSGACG00000034873" "ENSGACG00000035198"
##  [2656] "ENSGACG00000023656" "ENSGACG00000035815" "ENSGACG00000038091"
##  [2659] "ENSGACG00000025104" "ENSGACG00000029461" "ENSGACG00000035657"
##  [2662] "ENSGACG00000035648" "ENSGACG00000031482" "ENSGACG00000023989"
##  [2665] "ENSGACG00000037659" "ENSGACG00000029131" "ENSGACG00000023249"
##  [2668] "ENSGACG00000037430" "ENSGACG00000027891" "ENSGACG00000030714"
##  [2671] "ENSGACG00000029763" "ENSGACG00000031183" "ENSGACG00000019699"
##  [2674] "ENSGACG00000024607" "ENSGACG00000032033" "ENSGACG00000033833"
##  [2677] "ENSGACG00000029186" "ENSGACG00000032323" "ENSGACG00000024005"
##  [2680] "ENSGACG00000023510" "ENSGACG00000029565" "ENSGACG00000031333"
##  [2683] "ENSGACG00000033436" "ENSGACG00000030166" "ENSGACG00000024479"
##  [2686] "ENSGACG00000034392" "ENSGACG00000029653" "ENSGACG00000030958"
##  [2689] "ENSGACG00000030998" "ENSGACG00000026860" "ENSGACG00000026991"
##  [2692] "ENSGACG00000023339" "ENSGACG00000026397" "ENSGACG00000028383"
##  [2695] "ENSGACG00000036172" "ENSGACG00000037893" "ENSGACG00000024543"
##  [2698] "ENSGACG00000029849" "ENSGACG00000033358" "ENSGACG00000026909"
##  [2701] "ENSGACG00000032182" "ENSGACG00000032939" "ENSGACG00000035594"
##  [2704] "ENSGACG00000025562" "ENSGACG00000023462" "ENSGACG00000023362"
##  [2707] "ENSGACG00000034477" "ENSGACG00000032069" "ENSGACG00000020815"
##  [2710] "ENSGACG00000022390" "ENSGACG00000037443" "ENSGACG00000034326"
##  [2713] "ENSGACG00000020819" "ENSGACG00000023320" "ENSGACG00000020821"
##  [2716] "ENSGACG00000030293" "ENSGACG00000035730" "ENSGACG00000020823"
##  [2719] "ENSGACG00000018835" "ENSGACG00000000348" "ENSGACG00000000344"
##  [2722] "ENSGACG00000029316" "ENSGACG00000020824" "ENSGACG00000020825"
##  [2725] "ENSGACG00000034671" "ENSGACG00000030172" "ENSGACG00000035315"
##  [2728] "ENSGACG00000030823" "ENSGACG00000024135" "ENSGACG00000020826"
##  [2731] "ENSGACG00000020827" "ENSGACG00000020829" "ENSGACG00000020830"
##  [2734] "ENSGACG00000020832" "ENSGACG00000037031" "ENSGACG00000027054"
##  [2737] "ENSGACG00000020837" "ENSGACG00000020838" "ENSGACG00000020839"
##  [2740] "ENSGACG00000024409" "ENSGACG00000023966" "ENSGACG00000029804"
##  [2743] "ENSGACG00000024390" "ENSGACG00000020842" "ENSGACG00000020843"
##  [2746] "ENSGACG00000020844" "ENSGACG00000028555" "ENSGACG00000028692"
##  [2749] "ENSGACG00000020845" "ENSGACG00000020846" "ENSGACG00000020850"
##  [2752] "ENSGACG00000031262" "ENSGACG00000020847" "ENSGACG00000020848"
##  [2755] "ENSGACG00000023226" "ENSGACG00000023792" "ENSGACG00000020454"
##  [2758] "ENSGACG00000020456" "ENSGACG00000019083" "ENSGACG00000019130"
##  [2761] "ENSGACG00000037108" "ENSGACG00000020457" "ENSGACG00000020458"
##  [2764] "ENSGACG00000020459" "ENSGACG00000020460" "ENSGACG00000020461"
##  [2767] "ENSGACG00000023570" "ENSGACG00000020463" "ENSGACG00000020464"
##  [2770] "ENSGACG00000020465" "ENSGACG00000020466" "ENSGACG00000020467"
##  [2773] "ENSGACG00000020468" "ENSGACG00000025314" "ENSGACG00000020470"
##  [2776] "ENSGACG00000020471" "ENSGACG00000020473" "ENSGACG00000020474"
##  [2779] "ENSGACG00000032424" "ENSGACG00000020477" "ENSGACG00000033164"
##  [2782] "ENSGACG00000020479" "ENSGACG00000020483" "ENSGACG00000020485"
##  [2785] "ENSGACG00000020486" "ENSGACG00000037625" "ENSGACG00000020488"
##  [2788] "ENSGACG00000020489" "ENSGACG00000028485" "ENSGACG00000033227"
##  [2791] "ENSGACG00000020493" "ENSGACG00000020494" "ENSGACG00000020496"
##  [2794] "ENSGACG00000020497" "ENSGACG00000035202" "ENSGACG00000020507"
##  [2797] "ENSGACG00000020508" "ENSGACG00000035244" "ENSGACG00000020511"
##  [2800] "ENSGACG00000034368" "ENSGACG00000020513" "ENSGACG00000020514"
##  [2803] "ENSGACG00000020515" "ENSGACG00000020516" "ENSGACG00000020517"
##  [2806] "ENSGACG00000020519" "ENSGACG00000020520" "ENSGACG00000020521"
##  [2809] "ENSGACG00000033792" "ENSGACG00000029047" "ENSGACG00000020524"
##  [2812] "ENSGACG00000020525" "ENSGACG00000030152" "ENSGACG00000023482"
##  [2815] "ENSGACG00000025472" "ENSGACG00000020853" "ENSGACG00000020854"
##  [2818] "ENSGACG00000033809" "ENSGACG00000020855" "ENSGACG00000026881"
##  [2821] "ENSGACG00000020857" "ENSGACG00000035259" "ENSGACG00000020858"
##  [2824] "ENSGACG00000027705" "ENSGACG00000025083" "ENSGACG00000020862"
##  [2827] "ENSGACG00000020863" "ENSGACG00000026215" "ENSGACG00000034236"
##  [2830] "ENSGACG00000020865" "ENSGACG00000020867" "ENSGACG00000032248"
##  [2833] "ENSGACG00000020870" "ENSGACG00000020871" "ENSGACG00000020872"
##  [2836] "ENSGACG00000034029" "ENSGACG00000029768" "ENSGACG00000020876"
##  [2839] "ENSGACG00000020877" "ENSGACG00000020878" "ENSGACG00000020880"
##  [2842] "ENSGACG00000026819" "ENSGACG00000020883" "ENSGACG00000025212"
##  [2845] "ENSGACG00000020884" "ENSGACG00000027361" "ENSGACG00000020885"
##  [2848] "ENSGACG00000027136" "ENSGACG00000035347" "ENSGACG00000001755"
##  [2851] "ENSGACG00000023548" "ENSGACG00000001828" "ENSGACG00000033494"
##  [2854] "ENSGACG00000001855" "ENSGACG00000028251" "ENSGACG00000001861"
##  [2857] "ENSGACG00000001880" "ENSGACG00000001894" "ENSGACG00000001918"
##  [2860] "ENSGACG00000001916" "ENSGACG00000026722" "ENSGACG00000001942"
##  [2863] "ENSGACG00000020657" "ENSGACG00000020712" "ENSGACG00000030189"
##  [2866] "ENSGACG00000034662" "ENSGACG00000025970" "ENSGACG00000027394"
##  [2869] "ENSGACG00000026980" "ENSGACG00000032856" "ENSGACG00000030634"
##  [2872] "ENSGACG00000033986" "ENSGACG00000028676" "ENSGACG00000033607"
##  [2875] "ENSGACG00000026223" "ENSGACG00000025289" "ENSGACG00000037025"
##  [2878] "ENSGACG00000034275" "ENSGACG00000025253" "ENSGACG00000027286"
##  [2881] "ENSGACG00000033962" "ENSGACG00000033759" "ENSGACG00000034391"
##  [2884] "ENSGACG00000035993" "ENSGACG00000026285" "ENSGACG00000035714"
##  [2887] "ENSGACG00000025984" "ENSGACG00000034910" "ENSGACG00000037706"
##  [2890] "ENSGACG00000037111" "ENSGACG00000023331" "ENSGACG00000027483"
##  [2893] "ENSGACG00000037877" "ENSGACG00000035088" "ENSGACG00000036647"
##  [2896] "ENSGACG00000029331" "ENSGACG00000023709" "ENSGACG00000037666"
##  [2899] "ENSGACG00000034641" "ENSGACG00000024033" "ENSGACG00000030190"
##  [2902] "ENSGACG00000029853" "ENSGACG00000038114" "ENSGACG00000028538"
##  [2905] "ENSGACG00000027374" "ENSGACG00000034948" "ENSGACG00000033375"
##  [2908] "ENSGACG00000031073" "ENSGACG00000028917" "ENSGACG00000032201"
##  [2911] "ENSGACG00000030394" "ENSGACG00000026676" "ENSGACG00000033851"
##  [2914] "ENSGACG00000031276" "ENSGACG00000030292" "ENSGACG00000022381"
##  [2917] "ENSGACG00000020592" "ENSGACG00000020613" "ENSGACG00000020652"
##  [2920] "ENSGACG00000020379" "ENSGACG00000020387" "ENSGACG00000020796"
##  [2923] "ENSGACG00000020820" "ENSGACG00000020472" "ENSGACG00000028575"
##  [2926] "ENSGACG00000020879" "ENSGACG00000007144" "ENSGACG00000006546"
##  [2929] "ENSGACG00000006550" "ENSGACG00000006559" "ENSGACG00000006573"
##  [2932] "ENSGACG00000006586" "ENSGACG00000006591" "ENSGACG00000033700"
##  [2935] "ENSGACG00000006619" "ENSGACG00000006628" "ENSGACG00000006635"
##  [2938] "ENSGACG00000037719" "ENSGACG00000028870" "ENSGACG00000006679"
##  [2941] "ENSGACG00000036759" "ENSGACG00000006697" "ENSGACG00000036235"
##  [2944] "ENSGACG00000006708" "ENSGACG00000006784" "ENSGACG00000006790"
##  [2947] "ENSGACG00000006808" "ENSGACG00000006818" "ENSGACG00000006839"
##  [2950] "ENSGACG00000006866" "ENSGACG00000006869" "ENSGACG00000006874"
##  [2953] "ENSGACG00000030420" "ENSGACG00000024456" "ENSGACG00000011343"
##  [2956] "ENSGACG00000011346" "ENSGACG00000011369" "ENSGACG00000010517"
##  [2959] "ENSGACG00000006890" "ENSGACG00000010555" "ENSGACG00000029317"
##  [2962] "ENSGACG00000006900" "ENSGACG00000034834" "ENSGACG00000006917"
##  [2965] "ENSGACG00000010573" "ENSGACG00000010582" "ENSGACG00000010645"
##  [2968] "ENSGACG00000024045" "ENSGACG00000010665" "ENSGACG00000010675"
##  [2971] "ENSGACG00000010684" "ENSGACG00000028579" "ENSGACG00000010693"
##  [2974] "ENSGACG00000006936" "ENSGACG00000010697" "ENSGACG00000029611"
##  [2977] "ENSGACG00000006981" "ENSGACG00000033573" "ENSGACG00000007009"
##  [2980] "ENSGACG00000007016" "ENSGACG00000007027" "ENSGACG00000007046"
##  [2983] "ENSGACG00000007053" "ENSGACG00000007095" "ENSGACG00000007060"
##  [2986] "ENSGACG00000007068" "ENSGACG00000007071" "ENSGACG00000007101"
##  [2989] "ENSGACG00000007117" "ENSGACG00000029126" "ENSGACG00000028049"
##  [2992] "ENSGACG00000008673" "ENSGACG00000008685" "ENSGACG00000008722"
##  [2995] "ENSGACG00000023627" "ENSGACG00000008742" "ENSGACG00000008749"
##  [2998] "ENSGACG00000008759" "ENSGACG00000008790" "ENSGACG00000008792"
##  [3001] "ENSGACG00000008361" "ENSGACG00000033940" "ENSGACG00000008448"
##  [3004] "ENSGACG00000008468" "ENSGACG00000008485" "ENSGACG00000008504"
##  [3007] "ENSGACG00000008566" "ENSGACG00000008571" "ENSGACG00000008584"
##  [3010] "ENSGACG00000008602" "ENSGACG00000008604" "ENSGACG00000008610"
##  [3013] "ENSGACG00000008619" "ENSGACG00000008624" "ENSGACG00000008799"
##  [3016] "ENSGACG00000005876" "ENSGACG00000005880" "ENSGACG00000008820"
##  [3019] "ENSGACG00000008832" "ENSGACG00000008883" "ENSGACG00000008901"
##  [3022] "ENSGACG00000008962" "ENSGACG00000008979" "ENSGACG00000008988"
##  [3025] "ENSGACG00000008993" "ENSGACG00000009009" "ENSGACG00000025975"
##  [3028] "ENSGACG00000025487" "ENSGACG00000009035" "ENSGACG00000009060"
##  [3031] "ENSGACG00000009072" "ENSGACG00000010711" "ENSGACG00000010730"
##  [3034] "ENSGACG00000010737" "ENSGACG00000010741" "ENSGACG00000035937"
##  [3037] "ENSGACG00000010789" "ENSGACG00000032855" "ENSGACG00000010797"
##  [3040] "ENSGACG00000030440" "ENSGACG00000010818" "ENSGACG00000010833"
##  [3043] "ENSGACG00000005918" "ENSGACG00000010843" "ENSGACG00000005929"
##  [3046] "ENSGACG00000036214" "ENSGACG00000011447" "ENSGACG00000034036"
##  [3049] "ENSGACG00000011454" "ENSGACG00000011460" "ENSGACG00000023719"
##  [3052] "ENSGACG00000011469" "ENSGACG00000005955" "ENSGACG00000005962"
##  [3055] "ENSGACG00000030017" "ENSGACG00000009089" "ENSGACG00000009097"
##  [3058] "ENSGACG00000009109" "ENSGACG00000027363" "ENSGACG00000009125"
##  [3061] "ENSGACG00000009131" "ENSGACG00000009158" "ENSGACG00000009165"
##  [3064] "ENSGACG00000035336" "ENSGACG00000009176" "ENSGACG00000009184"
##  [3067] "ENSGACG00000009193" "ENSGACG00000026044" "ENSGACG00000009235"
##  [3070] "ENSGACG00000006331" "ENSGACG00000006344" "ENSGACG00000006348"
##  [3073] "ENSGACG00000006375" "ENSGACG00000006407" "ENSGACG00000006638"
##  [3076] "ENSGACG00000006415" "ENSGACG00000006419" "ENSGACG00000028491"
##  [3079] "ENSGACG00000009247" "ENSGACG00000009256" "ENSGACG00000009257"
##  [3082] "ENSGACG00000009314" "ENSGACG00000009336" "ENSGACG00000009349"
##  [3085] "ENSGACG00000035071" "ENSGACG00000009369" "ENSGACG00000029513"
##  [3088] "ENSGACG00000027517" "ENSGACG00000009383" "ENSGACG00000009417"
##  [3091] "ENSGACG00000009420" "ENSGACG00000024393" "ENSGACG00000009426"
##  [3094] "ENSGACG00000029112" "ENSGACG00000011496" "ENSGACG00000009437"
##  [3097] "ENSGACG00000028759" "ENSGACG00000011521" "ENSGACG00000010497"
##  [3100] "ENSGACG00000010514" "ENSGACG00000013384" "ENSGACG00000032556"
##  [3103] "ENSGACG00000009446" "ENSGACG00000013083" "ENSGACG00000028527"
##  [3106] "ENSGACG00000013085" "ENSGACG00000028257" "ENSGACG00000009494"
##  [3109] "ENSGACG00000009502" "ENSGACG00000027591" "ENSGACG00000013091"
##  [3112] "ENSGACG00000009539" "ENSGACG00000009589" "ENSGACG00000009594"
##  [3115] "ENSGACG00000009600" "ENSGACG00000009604" "ENSGACG00000023074"
##  [3118] "ENSGACG00000007161" "ENSGACG00000013115" "ENSGACG00000007171"
##  [3121] "ENSGACG00000007177" "ENSGACG00000029730" "ENSGACG00000007181"
##  [3124] "ENSGACG00000013140" "ENSGACG00000013147" "ENSGACG00000013152"
##  [3127] "ENSGACG00000034777" "ENSGACG00000007230" "ENSGACG00000013172"
##  [3130] "ENSGACG00000007267" "ENSGACG00000007272" "ENSGACG00000007276"
##  [3133] "ENSGACG00000008121" "ENSGACG00000013178" "ENSGACG00000013185"
##  [3136] "ENSGACG00000008129" "ENSGACG00000013192" "ENSGACG00000035798"
##  [3139] "ENSGACG00000013203" "ENSGACG00000023523" "ENSGACG00000013228"
##  [3142] "ENSGACG00000031416" "ENSGACG00000029899" "ENSGACG00000013257"
##  [3145] "ENSGACG00000013263" "ENSGACG00000013269" "ENSGACG00000013281"
##  [3148] "ENSGACG00000036410" "ENSGACG00000013311" "ENSGACG00000013325"
##  [3151] "ENSGACG00000034990" "ENSGACG00000013361" "ENSGACG00000013367"
##  [3154] "ENSGACG00000026740" "ENSGACG00000008154" "ENSGACG00000026590"
##  [3157] "ENSGACG00000011373" "ENSGACG00000008161" "ENSGACG00000008165"
##  [3160] "ENSGACG00000008191" "ENSGACG00000008193" "ENSGACG00000024191"
##  [3163] "ENSGACG00000008207" "ENSGACG00000008224" "ENSGACG00000028660"
##  [3166] "ENSGACG00000008238" "ENSGACG00000008258" "ENSGACG00000008263"
##  [3169] "ENSGACG00000008284" "ENSGACG00000024087" "ENSGACG00000008325"
##  [3172] "ENSGACG00000008339" "ENSGACG00000031703" "ENSGACG00000008351"
##  [3175] "ENSGACG00000032021" "ENSGACG00000008355" "ENSGACG00000008625"
##  [3178] "ENSGACG00000008658" "ENSGACG00000038128" "ENSGACG00000007332"
##  [3181] "ENSGACG00000007361" "ENSGACG00000007362" "ENSGACG00000007374"
##  [3184] "ENSGACG00000007388" "ENSGACG00000035550" "ENSGACG00000007395"
##  [3187] "ENSGACG00000007428" "ENSGACG00000007440" "ENSGACG00000007444"
##  [3190] "ENSGACG00000007464" "ENSGACG00000037388" "ENSGACG00000031618"
##  [3193] "ENSGACG00000007520" "ENSGACG00000007587" "ENSGACG00000030970"
##  [3196] "ENSGACG00000007598" "ENSGACG00000007608" "ENSGACG00000007613"
##  [3199] "ENSGACG00000007630" "ENSGACG00000007649" "ENSGACG00000036603"
##  [3202] "ENSGACG00000009826" "ENSGACG00000037551" "ENSGACG00000011383"
##  [3205] "ENSGACG00000007672" "ENSGACG00000007693" "ENSGACG00000007695"
##  [3208] "ENSGACG00000034920" "ENSGACG00000007700" "ENSGACG00000031554"
##  [3211] "ENSGACG00000007710" "ENSGACG00000007721" "ENSGACG00000007728"
##  [3214] "ENSGACG00000007754" "ENSGACG00000007783" "ENSGACG00000023157"
##  [3217] "ENSGACG00000007795" "ENSGACG00000036156" "ENSGACG00000036324"
##  [3220] "ENSGACG00000007837" "ENSGACG00000007841" "ENSGACG00000007856"
##  [3223] "ENSGACG00000007861" "ENSGACG00000007877" "ENSGACG00000007887"
##  [3226] "ENSGACG00000032089" "ENSGACG00000007960" "ENSGACG00000007968"
##  [3229] "ENSGACG00000011405" "ENSGACG00000007985" "ENSGACG00000008046"
##  [3232] "ENSGACG00000008058" "ENSGACG00000008066" "ENSGACG00000008068"
##  [3235] "ENSGACG00000006478" "ENSGACG00000006486" "ENSGACG00000030105"
##  [3238] "ENSGACG00000033766" "ENSGACG00000028872" "ENSGACG00000009629"
##  [3241] "ENSGACG00000031293" "ENSGACG00000009641" "ENSGACG00000009656"
##  [3244] "ENSGACG00000009668" "ENSGACG00000009693" "ENSGACG00000009721"
##  [3247] "ENSGACG00000009732" "ENSGACG00000008387" "ENSGACG00000008413"
##  [3250] "ENSGACG00000008439" "ENSGACG00000011413" "ENSGACG00000011416"
##  [3253] "ENSGACG00000011438" "ENSGACG00000009838" "ENSGACG00000033331"
##  [3256] "ENSGACG00000009863" "ENSGACG00000009864" "ENSGACG00000025367"
##  [3259] "ENSGACG00000009879" "ENSGACG00000027960" "ENSGACG00000034963"
##  [3262] "ENSGACG00000029582" "ENSGACG00000005639" "ENSGACG00000024529"
##  [3265] "ENSGACG00000005645" "ENSGACG00000010482" "ENSGACG00000010488"
##  [3268] "ENSGACG00000005662" "ENSGACG00000005680" "ENSGACG00000026760"
##  [3271] "ENSGACG00000005684" "ENSGACG00000005715" "ENSGACG00000005725"
##  [3274] "ENSGACG00000005732" "ENSGACG00000026225" "ENSGACG00000005747"
##  [3277] "ENSGACG00000026052" "ENSGACG00000012569" "ENSGACG00000036998"
##  [3280] "ENSGACG00000012605" "ENSGACG00000012622" "ENSGACG00000023798"
##  [3283] "ENSGACG00000012628" "ENSGACG00000023561" "ENSGACG00000012646"
##  [3286] "ENSGACG00000005762" "ENSGACG00000030478" "ENSGACG00000005766"
##  [3289] "ENSGACG00000024883" "ENSGACG00000005793" "ENSGACG00000005807"
##  [3292] "ENSGACG00000005815" "ENSGACG00000005820" "ENSGACG00000012684"
##  [3295] "ENSGACG00000012687" "ENSGACG00000012689" "ENSGACG00000012692"
##  [3298] "ENSGACG00000032239" "ENSGACG00000012697" "ENSGACG00000005825"
##  [3301] "ENSGACG00000029323" "ENSGACG00000012742" "ENSGACG00000012759"
##  [3304] "ENSGACG00000012798" "ENSGACG00000033068" "ENSGACG00000005843"
##  [3307] "ENSGACG00000012821" "ENSGACG00000005869" "ENSGACG00000012840"
##  [3310] "ENSGACG00000035688" "ENSGACG00000012909" "ENSGACG00000005985"
##  [3313] "ENSGACG00000006013" "ENSGACG00000006022" "ENSGACG00000032766"
##  [3316] "ENSGACG00000012917" "ENSGACG00000006034" "ENSGACG00000006068"
##  [3319] "ENSGACG00000006079" "ENSGACG00000012940" "ENSGACG00000006112"
##  [3322] "ENSGACG00000006128" "ENSGACG00000006132" "ENSGACG00000025808"
##  [3325] "ENSGACG00000033911" "ENSGACG00000026439" "ENSGACG00000006150"
##  [3328] "ENSGACG00000006157" "ENSGACG00000006174" "ENSGACG00000032295"
##  [3331] "ENSGACG00000006182" "ENSGACG00000037627" "ENSGACG00000006192"
##  [3334] "ENSGACG00000006201" "ENSGACG00000029414" "ENSGACG00000036170"
##  [3337] "ENSGACG00000006227" "ENSGACG00000006236" "ENSGACG00000006254"
##  [3340] "ENSGACG00000006274" "ENSGACG00000006296" "ENSGACG00000006300"
##  [3343] "ENSGACG00000006321" "ENSGACG00000006518" "ENSGACG00000004822"
##  [3346] "ENSGACG00000004811" "ENSGACG00000031015" "ENSGACG00000034850"
##  [3349] "ENSGACG00000004800" "ENSGACG00000028950" "ENSGACG00000004748"
##  [3352] "ENSGACG00000029137" "ENSGACG00000004741" "ENSGACG00000004731"
##  [3355] "ENSGACG00000004718" "ENSGACG00000004711" "ENSGACG00000004699"
##  [3358] "ENSGACG00000028638" "ENSGACG00000004682" "ENSGACG00000004656"
##  [3361] "ENSGACG00000004650" "ENSGACG00000004639" "ENSGACG00000004631"
##  [3364] "ENSGACG00000031895" "ENSGACG00000004619" "ENSGACG00000033746"
##  [3367] "ENSGACG00000030681" "ENSGACG00000037132" "ENSGACG00000038046"
##  [3370] "ENSGACG00000034717" "ENSGACG00000004570" "ENSGACG00000004539"
##  [3373] "ENSGACG00000004524" "ENSGACG00000004504" "ENSGACG00000004499"
##  [3376] "ENSGACG00000004487" "ENSGACG00000025960" "ENSGACG00000026746"
##  [3379] "ENSGACG00000004458" "ENSGACG00000004417" "ENSGACG00000004413"
##  [3382] "ENSGACG00000029440" "ENSGACG00000025771" "ENSGACG00000004395"
##  [3385] "ENSGACG00000025294" "ENSGACG00000001374" "ENSGACG00000033126"
##  [3388] "ENSGACG00000029235" "ENSGACG00000025673" "ENSGACG00000001387"
##  [3391] "ENSGACG00000001389" "ENSGACG00000001419" "ENSGACG00000004866"
##  [3394] "ENSGACG00000004875" "ENSGACG00000004876" "ENSGACG00000004890"
##  [3397] "ENSGACG00000004898" "ENSGACG00000004907" "ENSGACG00000034031"
##  [3400] "ENSGACG00000025717" "ENSGACG00000028306" "ENSGACG00000004934"
##  [3403] "ENSGACG00000030525" "ENSGACG00000029982" "ENSGACG00000004964"
##  [3406] "ENSGACG00000004996" "ENSGACG00000005004" "ENSGACG00000005038"
##  [3409] "ENSGACG00000035238" "ENSGACG00000024483" "ENSGACG00000029496"
##  [3412] "ENSGACG00000005051" "ENSGACG00000005115" "ENSGACG00000005148"
##  [3415] "ENSGACG00000005161" "ENSGACG00000005173" "ENSGACG00000005183"
##  [3418] "ENSGACG00000005214" "ENSGACG00000005228" "ENSGACG00000026901"
##  [3421] "ENSGACG00000005257" "ENSGACG00000005259" "ENSGACG00000005271"
##  [3424] "ENSGACG00000005285" "ENSGACG00000033866" "ENSGACG00000005302"
##  [3427] "ENSGACG00000005321" "ENSGACG00000005329" "ENSGACG00000005337"
##  [3430] "ENSGACG00000005362" "ENSGACG00000005366" "ENSGACG00000005375"
##  [3433] "ENSGACG00000005384" "ENSGACG00000005396" "ENSGACG00000005426"
##  [3436] "ENSGACG00000005430" "ENSGACG00000027140" "ENSGACG00000025612"
##  [3439] "ENSGACG00000005438" "ENSGACG00000005457" "ENSGACG00000005469"
##  [3442] "ENSGACG00000005511" "ENSGACG00000024996" "ENSGACG00000005523"
##  [3445] "ENSGACG00000005540" "ENSGACG00000000206" "ENSGACG00000034486"
##  [3448] "ENSGACG00000033622" "ENSGACG00000025208" "ENSGACG00000000209"
##  [3451] "ENSGACG00000000212" "ENSGACG00000000218" "ENSGACG00000000219"
##  [3454] "ENSGACG00000000225" "ENSGACG00000000230" "ENSGACG00000000242"
##  [3457] "ENSGACG00000036750" "ENSGACG00000000261" "ENSGACG00000000252"
##  [3460] "ENSGACG00000031324" "ENSGACG00000026401" "ENSGACG00000012986"
##  [3463] "ENSGACG00000012993" "ENSGACG00000013006" "ENSGACG00000013015"
##  [3466] "ENSGACG00000013023" "ENSGACG00000013042" "ENSGACG00000013044"
##  [3469] "ENSGACG00000009741" "ENSGACG00000009761" "ENSGACG00000009773"
##  [3472] "ENSGACG00000037662" "ENSGACG00000027269" "ENSGACG00000009792"
##  [3475] "ENSGACG00000009801" "ENSGACG00000000101" "ENSGACG00000036636"
##  [3478] "ENSGACG00000005559" "ENSGACG00000010850" "ENSGACG00000023785"
##  [3481] "ENSGACG00000028149" "ENSGACG00000005634" "ENSGACG00000010856"
##  [3484] "ENSGACG00000005621" "ENSGACG00000010870" "ENSGACG00000034153"
##  [3487] "ENSGACG00000010876" "ENSGACG00000010917" "ENSGACG00000024332"
##  [3490] "ENSGACG00000023354" "ENSGACG00000004992" "ENSGACG00000036707"
##  [3493] "ENSGACG00000024238" "ENSGACG00000033028" "ENSGACG00000024222"
##  [3496] "ENSGACG00000027067" "ENSGACG00000031436" "ENSGACG00000037752"
##  [3499] "ENSGACG00000010940" "ENSGACG00000026473" "ENSGACG00000023322"
##  [3502] "ENSGACG00000032296" "ENSGACG00000029306" "ENSGACG00000035212"
##  [3505] "ENSGACG00000029493" "ENSGACG00000037501" "ENSGACG00000026144"
##  [3508] "ENSGACG00000010951" "ENSGACG00000028191" "ENSGACG00000034556"
##  [3511] "ENSGACG00000035627" "ENSGACG00000036957" "ENSGACG00000026598"
##  [3514] "ENSGACG00000010966" "ENSGACG00000010971" "ENSGACG00000010984"
##  [3517] "ENSGACG00000011002" "ENSGACG00000025833" "ENSGACG00000037451"
##  [3520] "ENSGACG00000037277" "ENSGACG00000037018" "ENSGACG00000011018"
##  [3523] "ENSGACG00000023423" "ENSGACG00000027020" "ENSGACG00000031101"
##  [3526] "ENSGACG00000031358" "ENSGACG00000023182" "ENSGACG00000011096"
##  [3529] "ENSGACG00000036237" "ENSGACG00000012966" "ENSGACG00000024149"
##  [3532] "ENSGACG00000027981" "ENSGACG00000031328" "ENSGACG00000030001"
##  [3535] "ENSGACG00000030833" "ENSGACG00000034343" "ENSGACG00000034184"
##  [3538] "ENSGACG00000023009" "ENSGACG00000035115" "ENSGACG00000037065"
##  [3541] "ENSGACG00000029228" "ENSGACG00000022916" "ENSGACG00000027415"
##  [3544] "ENSGACG00000030867" "ENSGACG00000025354" "ENSGACG00000031535"
##  [3547] "ENSGACG00000012973" "ENSGACG00000034688" "ENSGACG00000030659"
##  [3550] "ENSGACG00000028154" "ENSGACG00000026653" "ENSGACG00000037446"
##  [3553] "ENSGACG00000023264" "ENSGACG00000033921" "ENSGACG00000036241"
##  [3556] "ENSGACG00000023259" "ENSGACG00000023349" "ENSGACG00000022793"
##  [3559] "ENSGACG00000022780" "ENSGACG00000008772" "ENSGACG00000005892"
##  [3562] "ENSGACG00000005965" "ENSGACG00000011112" "ENSGACG00000006467"
##  [3565] "ENSGACG00000006472" "ENSGACG00000005779" "ENSGACG00000006140"
##  [3568] "ENSGACG00000001107" "ENSGACG00000004405" "ENSGACG00000005067"
##  [3571] "ENSGACG00000005585" "ENSGACG00000025274" "ENSGACG00000011119"
##  [3574] "ENSGACG00000031361" "ENSGACG00000011167" "ENSGACG00000011181"
##  [3577] "ENSGACG00000011190" "ENSGACG00000037611" "ENSGACG00000032256"
##  [3580] "ENSGACG00000011259" "ENSGACG00000011266" "ENSGACG00000011281"
##  [3583] "ENSGACG00000011291" "ENSGACG00000011316" "ENSGACG00000011330"
##  [3586] "ENSGACG00000011337" "ENSGACG00000012981" "ENSGACG00000012983"
##  [3589] "ENSGACG00000033421" "ENSGACG00000013401" "ENSGACG00000013416"
##  [3592] "ENSGACG00000034215" "ENSGACG00000013425" "ENSGACG00000013438"
##  [3595] "ENSGACG00000013443" "ENSGACG00000035667" "ENSGACG00000036661"
##  [3598] "ENSGACG00000013467" "ENSGACG00000013476" "ENSGACG00000013482"
##  [3601] "ENSGACG00000013486" "ENSGACG00000013496" "ENSGACG00000013509"
##  [3604] "ENSGACG00000013524" "ENSGACG00000033262" "ENSGACG00000032935"
##  [3607] "ENSGACG00000013540" "ENSGACG00000028478" "ENSGACG00000013551"
##  [3610] "ENSGACG00000013556" "ENSGACG00000013558" "ENSGACG00000013565"
##  [3613] "ENSGACG00000013575" "ENSGACG00000013586" "ENSGACG00000013605"
##  [3616] "ENSGACG00000013614" "ENSGACG00000035069" "ENSGACG00000013633"
##  [3619] "ENSGACG00000013641" "ENSGACG00000013651" "ENSGACG00000009936"
##  [3622] "ENSGACG00000025626" "ENSGACG00000030618" "ENSGACG00000009951"
##  [3625] "ENSGACG00000009957" "ENSGACG00000009989" "ENSGACG00000009999"
##  [3628] "ENSGACG00000010019" "ENSGACG00000010023" "ENSGACG00000010028"
##  [3631] "ENSGACG00000010092" "ENSGACG00000029885" "ENSGACG00000010105"
##  [3634] "ENSGACG00000031323" "ENSGACG00000010151" "ENSGACG00000010115"
##  [3637] "ENSGACG00000032981" "ENSGACG00000010161" "ENSGACG00000010173"
##  [3640] "ENSGACG00000010208" "ENSGACG00000010210" "ENSGACG00000010220"
##  [3643] "ENSGACG00000028533" "ENSGACG00000010238" "ENSGACG00000010246"
##  [3646] "ENSGACG00000035150" "ENSGACG00000010253" "ENSGACG00000023418"
##  [3649] "ENSGACG00000033630" "ENSGACG00000010268" "ENSGACG00000010276"
##  [3652] "ENSGACG00000010302" "ENSGACG00000010305" "ENSGACG00000010329"
##  [3655] "ENSGACG00000010371" "ENSGACG00000010386" "ENSGACG00000010389"
##  [3658] "ENSGACG00000010410" "ENSGACG00000033242" "ENSGACG00000010435"
##  [3661] "ENSGACG00000010440" "ENSGACG00000010444" "ENSGACG00000011549"
##  [3664] "ENSGACG00000032093" "ENSGACG00000017185" "ENSGACG00000011579"
##  [3667] "ENSGACG00000011596" "ENSGACG00000030563" "ENSGACG00000011606"
##  [3670] "ENSGACG00000011660" "ENSGACG00000011668" "ENSGACG00000011679"
##  [3673] "ENSGACG00000022923" "ENSGACG00000011689" "ENSGACG00000011697"
##  [3676] "ENSGACG00000011703" "ENSGACG00000011717" "ENSGACG00000011727"
##  [3679] "ENSGACG00000011753" "ENSGACG00000011755" "ENSGACG00000036079"
##  [3682] "ENSGACG00000014464" "ENSGACG00000014459" "ENSGACG00000037100"
##  [3685] "ENSGACG00000026805" "ENSGACG00000014437" "ENSGACG00000009159"
##  [3688] "ENSGACG00000014429" "ENSGACG00000011798" "ENSGACG00000011804"
##  [3691] "ENSGACG00000037159" "ENSGACG00000014424" "ENSGACG00000025896"
##  [3694] "ENSGACG00000026157" "ENSGACG00000011816" "ENSGACG00000014419"
##  [3697] "ENSGACG00000030927" "ENSGACG00000011821" "ENSGACG00000014407"
##  [3700] "ENSGACG00000011861" "ENSGACG00000011908" "ENSGACG00000011911"
##  [3703] "ENSGACG00000036546" "ENSGACG00000011922" "ENSGACG00000028056"
##  [3706] "ENSGACG00000033187" "ENSGACG00000011950" "ENSGACG00000011959"
##  [3709] "ENSGACG00000037320" "ENSGACG00000011976" "ENSGACG00000011980"
##  [3712] "ENSGACG00000011982" "ENSGACG00000012003" "ENSGACG00000028271"
##  [3715] "ENSGACG00000012026" "ENSGACG00000012069" "ENSGACG00000014396"
##  [3718] "ENSGACG00000014388" "ENSGACG00000014378" "ENSGACG00000014473"
##  [3721] "ENSGACG00000035325" "ENSGACG00000014373" "ENSGACG00000014367"
##  [3724] "ENSGACG00000012085" "ENSGACG00000014358" "ENSGACG00000027994"
##  [3727] "ENSGACG00000027265" "ENSGACG00000034027" "ENSGACG00000012121"
##  [3730] "ENSGACG00000035329" "ENSGACG00000024071" "ENSGACG00000012139"
##  [3733] "ENSGACG00000026078" "ENSGACG00000014324" "ENSGACG00000012155"
##  [3736] "ENSGACG00000012162" "ENSGACG00000012165" "ENSGACG00000012176"
##  [3739] "ENSGACG00000032411" "ENSGACG00000012236" "ENSGACG00000012245"
##  [3742] "ENSGACG00000027997" "ENSGACG00000012248" "ENSGACG00000012258"
##  [3745] "ENSGACG00000012278" "ENSGACG00000012279" "ENSGACG00000012283"
##  [3748] "ENSGACG00000012293" "ENSGACG00000028607" "ENSGACG00000012302"
##  [3751] "ENSGACG00000012311" "ENSGACG00000012321" "ENSGACG00000012327"
##  [3754] "ENSGACG00000012331" "ENSGACG00000028400" "ENSGACG00000025443"
##  [3757] "ENSGACG00000012342" "ENSGACG00000012374" "ENSGACG00000034040"
##  [3760] "ENSGACG00000012383" "ENSGACG00000012407" "ENSGACG00000012419"
##  [3763] "ENSGACG00000012427" "ENSGACG00000012485" "ENSGACG00000012515"
##  [3766] "ENSGACG00000037904" "ENSGACG00000027366" "ENSGACG00000012524"
##  [3769] "ENSGACG00000015026" "ENSGACG00000014323" "ENSGACG00000014313"
##  [3772] "ENSGACG00000025642" "ENSGACG00000014294" "ENSGACG00000014289"
##  [3775] "ENSGACG00000012552" "ENSGACG00000023524" "ENSGACG00000027007"
##  [3778] "ENSGACG00000025525" "ENSGACG00000014280" "ENSGACG00000024713"
##  [3781] "ENSGACG00000031942" "ENSGACG00000014275" "ENSGACG00000031519"
##  [3784] "ENSGACG00000026041" "ENSGACG00000030405" "ENSGACG00000033814"
##  [3787] "ENSGACG00000028719" "ENSGACG00000025433" "ENSGACG00000036372"
##  [3790] "ENSGACG00000014268" "ENSGACG00000025040" "ENSGACG00000037683"
##  [3793] "ENSGACG00000035616" "ENSGACG00000024057" "ENSGACG00000024381"
##  [3796] "ENSGACG00000031666" "ENSGACG00000029878" "ENSGACG00000035285"
##  [3799] "ENSGACG00000030764" "ENSGACG00000026962" "ENSGACG00000023367"
##  [3802] "ENSGACG00000038097" "ENSGACG00000030141" "ENSGACG00000025894"
##  [3805] "ENSGACG00000033233" "ENSGACG00000023386" "ENSGACG00000036032"
##  [3808] "ENSGACG00000023260" "ENSGACG00000029628" "ENSGACG00000029133"
##  [3811] "ENSGACG00000032718" "ENSGACG00000028902" "ENSGACG00000037294"
##  [3814] "ENSGACG00000026533" "ENSGACG00000029249" "ENSGACG00000023755"
##  [3817] "ENSGACG00000028717" "ENSGACG00000032169" "ENSGACG00000029928"
##  [3820] "ENSGACG00000030221" "ENSGACG00000034351" "ENSGACG00000034629"
##  [3823] "ENSGACG00000035655" "ENSGACG00000031470" "ENSGACG00000030883"
##  [3826] "ENSGACG00000033680" "ENSGACG00000025856" "ENSGACG00000036922"
##  [3829] "ENSGACG00000025863" "ENSGACG00000023150" "ENSGACG00000032869"
##  [3832] "ENSGACG00000035206" "ENSGACG00000029967" "ENSGACG00000029729"
##  [3835] "ENSGACG00000037826" "ENSGACG00000026855" "ENSGACG00000024586"
##  [3838] "ENSGACG00000022124" "ENSGACG00000031523" "ENSGACG00000032912"
##  [3841] "ENSGACG00000013315" "ENSGACG00000031521" "ENSGACG00000012756"
##  [3844] "ENSGACG00000010927" "ENSGACG00000011555" "ENSGACG00000014256"
##  [3847] "ENSGACG00000014248" "ENSGACG00000014244" "ENSGACG00000014241"
##  [3850] "ENSGACG00000014231" "ENSGACG00000014228" "ENSGACG00000014225"
##  [3853] "ENSGACG00000014216" "ENSGACG00000014203" "ENSGACG00000025119"
##  [3856] "ENSGACG00000014198" "ENSGACG00000014196" "ENSGACG00000014174"
##  [3859] "ENSGACG00000014167" "ENSGACG00000037731" "ENSGACG00000014150"
##  [3862] "ENSGACG00000014143" "ENSGACG00000013663" "ENSGACG00000013667"
##  [3865] "ENSGACG00000013682" "ENSGACG00000013689" "ENSGACG00000013701"
##  [3868] "ENSGACG00000026326" "ENSGACG00000035405" "ENSGACG00000035632"
##  [3871] "ENSGACG00000013703" "ENSGACG00000000413" "ENSGACG00000000415"
##  [3874] "ENSGACG00000032528" "ENSGACG00000037724" "ENSGACG00000031315"
##  [3877] "ENSGACG00000033339" "ENSGACG00000037855" "ENSGACG00000000422"
##  [3880] "ENSGACG00000015357" "ENSGACG00000015349" "ENSGACG00000036834"
##  [3883] "ENSGACG00000032293" "ENSGACG00000015339" "ENSGACG00000015336"
##  [3886] "ENSGACG00000015333" "ENSGACG00000015330" "ENSGACG00000015321"
##  [3889] "ENSGACG00000028308" "ENSGACG00000015312" "ENSGACG00000015303"
##  [3892] "ENSGACG00000015295" "ENSGACG00000015289" "ENSGACG00000015286"
##  [3895] "ENSGACG00000015285" "ENSGACG00000015284" "ENSGACG00000015279"
##  [3898] "ENSGACG00000015276" "ENSGACG00000024166" "ENSGACG00000024941"
##  [3901] "ENSGACG00000015263" "ENSGACG00000015253" "ENSGACG00000025042"
##  [3904] "ENSGACG00000015240" "ENSGACG00000037898" "ENSGACG00000034322"
##  [3907] "ENSGACG00000025012" "ENSGACG00000015234" "ENSGACG00000015223"
##  [3910] "ENSGACG00000015217" "ENSGACG00000015213" "ENSGACG00000015210"
##  [3913] "ENSGACG00000015207" "ENSGACG00000015201" "ENSGACG00000015198"
##  [3916] "ENSGACG00000015197" "ENSGACG00000015192" "ENSGACG00000015188"
##  [3919] "ENSGACG00000015181" "ENSGACG00000023533" "ENSGACG00000015177"
##  [3922] "ENSGACG00000015172" "ENSGACG00000015169" "ENSGACG00000025827"
##  [3925] "ENSGACG00000015159" "ENSGACG00000025243" "ENSGACG00000015152"
##  [3928] "ENSGACG00000036477" "ENSGACG00000015151" "ENSGACG00000015146"
##  [3931] "ENSGACG00000015143" "ENSGACG00000036130" "ENSGACG00000015135"
##  [3934] "ENSGACG00000015132" "ENSGACG00000015126" "ENSGACG00000015122"
##  [3937] "ENSGACG00000015116" "ENSGACG00000015105" "ENSGACG00000023295"
##  [3940] "ENSGACG00000015089" "ENSGACG00000015087" "ENSGACG00000024301"
##  [3943] "ENSGACG00000015081" "ENSGACG00000015076" "ENSGACG00000030198"
##  [3946] "ENSGACG00000015070" "ENSGACG00000015068" "ENSGACG00000024711"
##  [3949] "ENSGACG00000015062" "ENSGACG00000015058" "ENSGACG00000015061"
##  [3952] "ENSGACG00000015056" "ENSGACG00000015038" "ENSGACG00000015035"
##  [3955] "ENSGACG00000015030" "ENSGACG00000033823" "ENSGACG00000030052"
##  [3958] "ENSGACG00000015024" "ENSGACG00000015007" "ENSGACG00000031135"
##  [3961] "ENSGACG00000014968" "ENSGACG00000014966" "ENSGACG00000014964"
##  [3964] "ENSGACG00000014961" "ENSGACG00000014957" "ENSGACG00000033330"
##  [3967] "ENSGACG00000014951" "ENSGACG00000035442" "ENSGACG00000014949"
##  [3970] "ENSGACG00000027328" "ENSGACG00000014941" "ENSGACG00000014937"
##  [3973] "ENSGACG00000014934" "ENSGACG00000034768" "ENSGACG00000014932"
##  [3976] "ENSGACG00000014926" "ENSGACG00000014925" "ENSGACG00000014917"
##  [3979] "ENSGACG00000014909" "ENSGACG00000029061" "ENSGACG00000014901"
##  [3982] "ENSGACG00000014898" "ENSGACG00000014895" "ENSGACG00000014889"
##  [3985] "ENSGACG00000014886" "ENSGACG00000014883" "ENSGACG00000014869"
##  [3988] "ENSGACG00000013900" "ENSGACG00000014853" "ENSGACG00000014851"
##  [3991] "ENSGACG00000032537" "ENSGACG00000014843" "ENSGACG00000014838"
##  [3994] "ENSGACG00000014831" "ENSGACG00000014827" "ENSGACG00000014821"
##  [3997] "ENSGACG00000023110" "ENSGACG00000026878" "ENSGACG00000014817"
##  [4000] "ENSGACG00000014810" "ENSGACG00000014804" "ENSGACG00000014799"
##  [4003] "ENSGACG00000014790" "ENSGACG00000014773" "ENSGACG00000034516"
##  [4006] "ENSGACG00000014754" "ENSGACG00000014752" "ENSGACG00000014735"
##  [4009] "ENSGACG00000014727" "ENSGACG00000031242" "ENSGACG00000014720"
##  [4012] "ENSGACG00000014712" "ENSGACG00000037262" "ENSGACG00000023410"
##  [4015] "ENSGACG00000014493" "ENSGACG00000014669" "ENSGACG00000014645"
##  [4018] "ENSGACG00000014643" "ENSGACG00000035946" "ENSGACG00000014627"
##  [4021] "ENSGACG00000014605" "ENSGACG00000014601" "ENSGACG00000014600"
##  [4024] "ENSGACG00000014598" "ENSGACG00000034144" "ENSGACG00000014592"
##  [4027] "ENSGACG00000014580" "ENSGACG00000026013" "ENSGACG00000030522"
##  [4030] "ENSGACG00000014573" "ENSGACG00000014564" "ENSGACG00000014546"
##  [4033] "ENSGACG00000033297" "ENSGACG00000014542" "ENSGACG00000014540"
##  [4036] "ENSGACG00000014503" "ENSGACG00000035117" "ENSGACG00000014498"
##  [4039] "ENSGACG00000014496" "ENSGACG00000014485" "ENSGACG00000014137"
##  [4042] "ENSGACG00000014135" "ENSGACG00000014133" "ENSGACG00000014119"
##  [4045] "ENSGACG00000014115" "ENSGACG00000014108" "ENSGACG00000014102"
##  [4048] "ENSGACG00000014091" "ENSGACG00000014087" "ENSGACG00000027332"
##  [4051] "ENSGACG00000022964" "ENSGACG00000014074" "ENSGACG00000024715"
##  [4054] "ENSGACG00000014050" "ENSGACG00000014031" "ENSGACG00000014014"
##  [4057] "ENSGACG00000014013" "ENSGACG00000013999" "ENSGACG00000013989"
##  [4060] "ENSGACG00000001329" "ENSGACG00000024974" "ENSGACG00000013980"
##  [4063] "ENSGACG00000013952" "ENSGACG00000035142" "ENSGACG00000013970"
##  [4066] "ENSGACG00000013961" "ENSGACG00000013941" "ENSGACG00000013935"
##  [4069] "ENSGACG00000036689" "ENSGACG00000026080" "ENSGACG00000034593"
##  [4072] "ENSGACG00000013923" "ENSGACG00000036893" "ENSGACG00000013922"
##  [4075] "ENSGACG00000013910" "ENSGACG00000013901" "ENSGACG00000013884"
##  [4078] "ENSGACG00000013867" "ENSGACG00000013848" "ENSGACG00000013831"
##  [4081] "ENSGACG00000013821" "ENSGACG00000013817" "ENSGACG00000013811"
##  [4084] "ENSGACG00000013778" "ENSGACG00000013746" "ENSGACG00000030077"
##  [4087] "ENSGACG00000027657" "ENSGACG00000024169" "ENSGACG00000013728"
##  [4090] "ENSGACG00000013725" "ENSGACG00000013712" "ENSGACG00000032594"
##  [4093] "ENSGACG00000013709" "ENSGACG00000023528" "ENSGACG00000014876"
##  [4096] "ENSGACG00000035170" "ENSGACG00000014896" "ENSGACG00000014899"
##  [4099] "ENSGACG00000014904" "ENSGACG00000033264" "ENSGACG00000035184"
##  [4102] "ENSGACG00000014916" "ENSGACG00000014923" "ENSGACG00000014928"
##  [4105] "ENSGACG00000014939" "ENSGACG00000014945" "ENSGACG00000027928"
##  [4108] "ENSGACG00000014955" "ENSGACG00000029295" "ENSGACG00000014958"
##  [4111] "ENSGACG00000014962" "ENSGACG00000029734" "ENSGACG00000014992"
##  [4114] "ENSGACG00000014974" "ENSGACG00000014986" "ENSGACG00000014988"
##  [4117] "ENSGACG00000014995" "ENSGACG00000014998" "ENSGACG00000015002"
##  [4120] "ENSGACG00000015016" "ENSGACG00000015020" "ENSGACG00000015031"
##  [4123] "ENSGACG00000015033" "ENSGACG00000015036" "ENSGACG00000031080"
##  [4126] "ENSGACG00000015043" "ENSGACG00000015048" "ENSGACG00000031960"
##  [4129] "ENSGACG00000015057" "ENSGACG00000015060" "ENSGACG00000015071"
##  [4132] "ENSGACG00000015074" "ENSGACG00000015083" "ENSGACG00000015660"
##  [4135] "ENSGACG00000032694" "ENSGACG00000015657" "ENSGACG00000030256"
##  [4138] "ENSGACG00000015646" "ENSGACG00000015642" "ENSGACG00000015629"
##  [4141] "ENSGACG00000015622" "ENSGACG00000015619" "ENSGACG00000015617"
##  [4144] "ENSGACG00000015615" "ENSGACG00000028562" "ENSGACG00000015610"
##  [4147] "ENSGACG00000015609" "ENSGACG00000015599" "ENSGACG00000015589"
##  [4150] "ENSGACG00000015587" "ENSGACG00000015585" "ENSGACG00000034654"
##  [4153] "ENSGACG00000015579" "ENSGACG00000033666" "ENSGACG00000001802"
##  [4156] "ENSGACG00000015569" "ENSGACG00000015565" "ENSGACG00000036062"
##  [4159] "ENSGACG00000015560" "ENSGACG00000030151" "ENSGACG00000015556"
##  [4162] "ENSGACG00000015553" "ENSGACG00000015537" "ENSGACG00000015535"
##  [4165] "ENSGACG00000037357" "ENSGACG00000015532" "ENSGACG00000030100"
##  [4168] "ENSGACG00000001785" "ENSGACG00000015516" "ENSGACG00000015506"
##  [4171] "ENSGACG00000032846" "ENSGACG00000026507" "ENSGACG00000015371"
##  [4174] "ENSGACG00000015380" "ENSGACG00000015383" "ENSGACG00000015398"
##  [4177] "ENSGACG00000015399" "ENSGACG00000015402" "ENSGACG00000029373"
##  [4180] "ENSGACG00000015409" "ENSGACG00000015420" "ENSGACG00000015430"
##  [4183] "ENSGACG00000015448" "ENSGACG00000015443" "ENSGACG00000015453"
##  [4186] "ENSGACG00000015457" "ENSGACG00000026269" "ENSGACG00000015489"
##  [4189] "ENSGACG00000031407" "ENSGACG00000015491" "ENSGACG00000015494"
##  [4192] "ENSGACG00000015497" "ENSGACG00000025114" "ENSGACG00000015498"
##  [4195] "ENSGACG00000037691" "ENSGACG00000026618" "ENSGACG00000037458"
##  [4198] "ENSGACG00000028713" "ENSGACG00000035869" "ENSGACG00000024387"
##  [4201] "ENSGACG00000024280" "ENSGACG00000027310" "ENSGACG00000026018"
##  [4204] "ENSGACG00000033335" "ENSGACG00000033490" "ENSGACG00000026493"
##  [4207] "ENSGACG00000028361" "ENSGACG00000029356" "ENSGACG00000029518"
##  [4210] "ENSGACG00000028702" "ENSGACG00000037227" "ENSGACG00000035873"
##  [4213] "ENSGACG00000033269" "ENSGACG00000023076" "ENSGACG00000031709"
##  [4216] "ENSGACG00000032040" "ENSGACG00000027411" "ENSGACG00000036978"
##  [4219] "ENSGACG00000014576" "ENSGACG00000030014" "ENSGACG00000030296"
##  [4222] "ENSGACG00000027189" "ENSGACG00000034025" "ENSGACG00000023263"
##  [4225] "ENSGACG00000029438" "ENSGACG00000033265" "ENSGACG00000028993"
##  [4228] "ENSGACG00000031595" "ENSGACG00000028454" "ENSGACG00000024520"
##  [4231] "ENSGACG00000024016" "ENSGACG00000036668" "ENSGACG00000033042"
##  [4234] "ENSGACG00000025222" "ENSGACG00000037836" "ENSGACG00000031671"
##  [4237] "ENSGACG00000028195" "ENSGACG00000037974" "ENSGACG00000037038"
##  [4240] "ENSGACG00000025501" "ENSGACG00000035709" "ENSGACG00000024405"
##  [4243] "ENSGACG00000035048" "ENSGACG00000030061" "ENSGACG00000033963"
##  [4246] "ENSGACG00000015229" "ENSGACG00000015050" "ENSGACG00000014983"
##  [4249] "ENSGACG00000014833" "ENSGACG00000013933" "ENSGACG00000013914"
##  [4252] "ENSGACG00000032540" "ENSGACG00000031447" "ENSGACG00000016313"
##  [4255] "ENSGACG00000031111" "ENSGACG00000028837" "ENSGACG00000016317"
##  [4258] "ENSGACG00000016334" "ENSGACG00000016280" "ENSGACG00000016281"
##  [4261] "ENSGACG00000016288" "ENSGACG00000033703" "ENSGACG00000036758"
##  [4264] "ENSGACG00000016298" "ENSGACG00000036124" "ENSGACG00000016301"
##  [4267] "ENSGACG00000016307" "ENSGACG00000016884" "ENSGACG00000016891"
##  [4270] "ENSGACG00000016894" "ENSGACG00000016895" "ENSGACG00000016897"
##  [4273] "ENSGACG00000016909" "ENSGACG00000016913" "ENSGACG00000016920"
##  [4276] "ENSGACG00000016926" "ENSGACG00000016931" "ENSGACG00000016932"
##  [4279] "ENSGACG00000016936" "ENSGACG00000016944" "ENSGACG00000016952"
##  [4282] "ENSGACG00000016953" "ENSGACG00000016957" "ENSGACG00000016961"
##  [4285] "ENSGACG00000016705" "ENSGACG00000016710" "ENSGACG00000016702"
##  [4288] "ENSGACG00000027193" "ENSGACG00000015982" "ENSGACG00000015987"
##  [4291] "ENSGACG00000015990" "ENSGACG00000015995" "ENSGACG00000015997"
##  [4294] "ENSGACG00000016001" "ENSGACG00000016002" "ENSGACG00000016006"
##  [4297] "ENSGACG00000016009" "ENSGACG00000016025" "ENSGACG00000016030"
##  [4300] "ENSGACG00000016034" "ENSGACG00000016035" "ENSGACG00000016045"
##  [4303] "ENSGACG00000016041" "ENSGACG00000016339" "ENSGACG00000031455"
##  [4306] "ENSGACG00000016355" "ENSGACG00000028881" "ENSGACG00000016359"
##  [4309] "ENSGACG00000015875" "ENSGACG00000026366" "ENSGACG00000015877"
##  [4312] "ENSGACG00000035962" "ENSGACG00000015882" "ENSGACG00000015883"
##  [4315] "ENSGACG00000016365" "ENSGACG00000034741" "ENSGACG00000016368"
##  [4318] "ENSGACG00000016394" "ENSGACG00000035984" "ENSGACG00000037420"
##  [4321] "ENSGACG00000016405" "ENSGACG00000016415" "ENSGACG00000016413"
##  [4324] "ENSGACG00000016966" "ENSGACG00000016986" "ENSGACG00000016992"
##  [4327] "ENSGACG00000016994" "ENSGACG00000015929" "ENSGACG00000015931"
##  [4330] "ENSGACG00000015933" "ENSGACG00000016336" "ENSGACG00000015700"
##  [4333] "ENSGACG00000015701" "ENSGACG00000031504" "ENSGACG00000017000"
##  [4336] "ENSGACG00000017012" "ENSGACG00000017021" "ENSGACG00000017030"
##  [4339] "ENSGACG00000017034" "ENSGACG00000017038" "ENSGACG00000035204"
##  [4342] "ENSGACG00000017046" "ENSGACG00000017047" "ENSGACG00000017056"
##  [4345] "ENSGACG00000016694" "ENSGACG00000031951" "ENSGACG00000015637"
##  [4348] "ENSGACG00000015638" "ENSGACG00000015640" "ENSGACG00000015643"
##  [4351] "ENSGACG00000015645" "ENSGACG00000015648" "ENSGACG00000015653"
##  [4354] "ENSGACG00000015654" "ENSGACG00000023108" "ENSGACG00000015658"
##  [4357] "ENSGACG00000015661" "ENSGACG00000015663" "ENSGACG00000015667"
##  [4360] "ENSGACG00000015671" "ENSGACG00000015676" "ENSGACG00000015677"
##  [4363] "ENSGACG00000015680" "ENSGACG00000016700" "ENSGACG00000034051"
##  [4366] "ENSGACG00000016446" "ENSGACG00000016451" "ENSGACG00000016457"
##  [4369] "ENSGACG00000016462" "ENSGACG00000016467" "ENSGACG00000016481"
##  [4372] "ENSGACG00000028025" "ENSGACG00000016718" "ENSGACG00000016722"
##  [4375] "ENSGACG00000016727" "ENSGACG00000016729" "ENSGACG00000016737"
##  [4378] "ENSGACG00000016741" "ENSGACG00000026110" "ENSGACG00000016748"
##  [4381] "ENSGACG00000031186" "ENSGACG00000023134" "ENSGACG00000016752"
##  [4384] "ENSGACG00000016759" "ENSGACG00000037236" "ENSGACG00000016765"
##  [4387] "ENSGACG00000016770" "ENSGACG00000016773" "ENSGACG00000023695"
##  [4390] "ENSGACG00000016786" "ENSGACG00000016790" "ENSGACG00000016809"
##  [4393] "ENSGACG00000016813" "ENSGACG00000016834" "ENSGACG00000028627"
##  [4396] "ENSGACG00000016836" "ENSGACG00000027931" "ENSGACG00000016845"
##  [4399] "ENSGACG00000016860" "ENSGACG00000016866" "ENSGACG00000016871"
##  [4402] "ENSGACG00000016872" "ENSGACG00000016875" "ENSGACG00000016878"
##  [4405] "ENSGACG00000025435" "ENSGACG00000016484" "ENSGACG00000037334"
##  [4408] "ENSGACG00000035051" "ENSGACG00000016485" "ENSGACG00000016490"
##  [4411] "ENSGACG00000016492" "ENSGACG00000015694" "ENSGACG00000030576"
##  [4414] "ENSGACG00000015698" "ENSGACG00000016430" "ENSGACG00000016438"
##  [4417] "ENSGACG00000016441" "ENSGACG00000025803" "ENSGACG00000015937"
##  [4420] "ENSGACG00000015940" "ENSGACG00000015943" "ENSGACG00000015948"
##  [4423] "ENSGACG00000028524" "ENSGACG00000015953" "ENSGACG00000024171"
##  [4426] "ENSGACG00000028531" "ENSGACG00000015958" "ENSGACG00000015961"
##  [4429] "ENSGACG00000015963" "ENSGACG00000015967" "ENSGACG00000015970"
##  [4432] "ENSGACG00000024819" "ENSGACG00000015975" "ENSGACG00000015976"
##  [4435] "ENSGACG00000015977" "ENSGACG00000023833" "ENSGACG00000016494"
##  [4438] "ENSGACG00000016500" "ENSGACG00000016685" "ENSGACG00000016505"
##  [4441] "ENSGACG00000016512" "ENSGACG00000029023" "ENSGACG00000024808"
##  [4444] "ENSGACG00000016525" "ENSGACG00000016527" "ENSGACG00000016534"
##  [4447] "ENSGACG00000016539" "ENSGACG00000023477" "ENSGACG00000016549"
##  [4450] "ENSGACG00000016547" "ENSGACG00000030669" "ENSGACG00000016555"
##  [4453] "ENSGACG00000016557" "ENSGACG00000016559" "ENSGACG00000016569"
##  [4456] "ENSGACG00000016575" "ENSGACG00000016578" "ENSGACG00000016579"
##  [4459] "ENSGACG00000016583" "ENSGACG00000032499" "ENSGACG00000016590"
##  [4462] "ENSGACG00000016594" "ENSGACG00000016600" "ENSGACG00000016620"
##  [4465] "ENSGACG00000016626" "ENSGACG00000029102" "ENSGACG00000016629"
##  [4468] "ENSGACG00000016630" "ENSGACG00000016637" "ENSGACG00000016648"
##  [4471] "ENSGACG00000016670" "ENSGACG00000016678" "ENSGACG00000027434"
##  [4474] "ENSGACG00000016682" "ENSGACG00000016050" "ENSGACG00000016059"
##  [4477] "ENSGACG00000016060" "ENSGACG00000016061" "ENSGACG00000023437"
##  [4480] "ENSGACG00000034056" "ENSGACG00000016068" "ENSGACG00000032641"
##  [4483] "ENSGACG00000016072" "ENSGACG00000016077" "ENSGACG00000016082"
##  [4486] "ENSGACG00000017267" "ENSGACG00000016084" "ENSGACG00000017273"
##  [4489] "ENSGACG00000017279" "ENSGACG00000016103" "ENSGACG00000016105"
##  [4492] "ENSGACG00000026918" "ENSGACG00000016111" "ENSGACG00000032901"
##  [4495] "ENSGACG00000017603" "ENSGACG00000016118" "ENSGACG00000017600"
##  [4498] "ENSGACG00000017254" "ENSGACG00000016120" "ENSGACG00000025135"
##  [4501] "ENSGACG00000017256" "ENSGACG00000016129" "ENSGACG00000031887"
##  [4504] "ENSGACG00000017591" "ENSGACG00000016131" "ENSGACG00000016137"
##  [4507] "ENSGACG00000017581" "ENSGACG00000016139" "ENSGACG00000016143"
##  [4510] "ENSGACG00000016144" "ENSGACG00000016147" "ENSGACG00000016150"
##  [4513] "ENSGACG00000016151" "ENSGACG00000016158" "ENSGACG00000017572"
##  [4516] "ENSGACG00000016164" "ENSGACG00000017570" "ENSGACG00000016166"
##  [4519] "ENSGACG00000016168" "ENSGACG00000017562" "ENSGACG00000017552"
##  [4522] "ENSGACG00000033885" "ENSGACG00000017544" "ENSGACG00000028237"
##  [4525] "ENSGACG00000031573" "ENSGACG00000017531" "ENSGACG00000017527"
##  [4528] "ENSGACG00000016176" "ENSGACG00000017523" "ENSGACG00000033095"
##  [4531] "ENSGACG00000016182" "ENSGACG00000017522" "ENSGACG00000017520"
##  [4534] "ENSGACG00000017516" "ENSGACG00000016185" "ENSGACG00000017514"
##  [4537] "ENSGACG00000016190" "ENSGACG00000017513" "ENSGACG00000017512"
##  [4540] "ENSGACG00000016198" "ENSGACG00000017508" "ENSGACG00000034744"
##  [4543] "ENSGACG00000016202" "ENSGACG00000034298" "ENSGACG00000022985"
##  [4546] "ENSGACG00000016215" "ENSGACG00000016221" "ENSGACG00000016226"
##  [4549] "ENSGACG00000016236" "ENSGACG00000033133" "ENSGACG00000016239"
##  [4552] "ENSGACG00000016241" "ENSGACG00000016243" "ENSGACG00000016245"
##  [4555] "ENSGACG00000016247" "ENSGACG00000016253" "ENSGACG00000016256"
##  [4558] "ENSGACG00000016259" "ENSGACG00000016261" "ENSGACG00000016262"
##  [4561] "ENSGACG00000025775" "ENSGACG00000015885" "ENSGACG00000015895"
##  [4564] "ENSGACG00000015897" "ENSGACG00000024446" "ENSGACG00000015906"
##  [4567] "ENSGACG00000025942" "ENSGACG00000015911" "ENSGACG00000035721"
##  [4570] "ENSGACG00000015919" "ENSGACG00000015923" "ENSGACG00000025961"
##  [4573] "ENSGACG00000016275" "ENSGACG00000015683" "ENSGACG00000017307"
##  [4576] "ENSGACG00000017310" "ENSGACG00000029382" "ENSGACG00000017313"
##  [4579] "ENSGACG00000017315" "ENSGACG00000024044" "ENSGACG00000017319"
##  [4582] "ENSGACG00000017321" "ENSGACG00000017325" "ENSGACG00000026945"
##  [4585] "ENSGACG00000017331" "ENSGACG00000017333" "ENSGACG00000008520"
##  [4588] "ENSGACG00000017338" "ENSGACG00000017339" "ENSGACG00000017343"
##  [4591] "ENSGACG00000028240" "ENSGACG00000017352" "ENSGACG00000032432"
##  [4594] "ENSGACG00000017358" "ENSGACG00000030785" "ENSGACG00000017384"
##  [4597] "ENSGACG00000017391" "ENSGACG00000017394" "ENSGACG00000017423"
##  [4600] "ENSGACG00000017403" "ENSGACG00000017413" "ENSGACG00000017419"
##  [4603] "ENSGACG00000017426" "ENSGACG00000025754" "ENSGACG00000017491"
##  [4606] "ENSGACG00000025939" "ENSGACG00000034137" "ENSGACG00000017438"
##  [4609] "ENSGACG00000029006" "ENSGACG00000032430" "ENSGACG00000017448"
##  [4612] "ENSGACG00000017453" "ENSGACG00000025372" "ENSGACG00000017465"
##  [4615] "ENSGACG00000017469" "ENSGACG00000017471" "ENSGACG00000017472"
##  [4618] "ENSGACG00000017476" "ENSGACG00000017479" "ENSGACG00000017487"
##  [4621] "ENSGACG00000017488" "ENSGACG00000024618" "ENSGACG00000034661"
##  [4624] "ENSGACG00000030730" "ENSGACG00000017506" "ENSGACG00000017060"
##  [4627] "ENSGACG00000017063" "ENSGACG00000037660" "ENSGACG00000017073"
##  [4630] "ENSGACG00000017075" "ENSGACG00000017086" "ENSGACG00000017087"
##  [4633] "ENSGACG00000027598" "ENSGACG00000017092" "ENSGACG00000017094"
##  [4636] "ENSGACG00000017096" "ENSGACG00000015264" "ENSGACG00000015270"
##  [4639] "ENSGACG00000017098" "ENSGACG00000015262" "ENSGACG00000014322"
##  [4642] "ENSGACG00000014347" "ENSGACG00000023342" "ENSGACG00000014364"
##  [4645] "ENSGACG00000014764" "ENSGACG00000014778" "ENSGACG00000014372"
##  [4648] "ENSGACG00000014375" "ENSGACG00000014379" "ENSGACG00000014385"
##  [4651] "ENSGACG00000014390" "ENSGACG00000014393" "ENSGACG00000036444"
##  [4654] "ENSGACG00000017100" "ENSGACG00000034186" "ENSGACG00000014406"
##  [4657] "ENSGACG00000014415" "ENSGACG00000015704" "ENSGACG00000031674"
##  [4660] "ENSGACG00000015708" "ENSGACG00000014422" "ENSGACG00000015715"
##  [4663] "ENSGACG00000037058" "ENSGACG00000014479" "ENSGACG00000014482"
##  [4666] "ENSGACG00000017105" "ENSGACG00000029947" "ENSGACG00000038129"
##  [4669] "ENSGACG00000014495" "ENSGACG00000017112" "ENSGACG00000032777"
##  [4672] "ENSGACG00000014499" "ENSGACG00000017116" "ENSGACG00000017121"
##  [4675] "ENSGACG00000015725" "ENSGACG00000015726" "ENSGACG00000015749"
##  [4678] "ENSGACG00000015728" "ENSGACG00000014504" "ENSGACG00000014510"
##  [4681] "ENSGACG00000034976" "ENSGACG00000015734" "ENSGACG00000014526"
##  [4684] "ENSGACG00000015742" "ENSGACG00000028074" "ENSGACG00000015747"
##  [4687] "ENSGACG00000024682" "ENSGACG00000015752" "ENSGACG00000015754"
##  [4690] "ENSGACG00000015773" "ENSGACG00000015774" "ENSGACG00000014530"
##  [4693] "ENSGACG00000015776" "ENSGACG00000017125" "ENSGACG00000014541"
##  [4696] "ENSGACG00000014543" "ENSGACG00000017635" "ENSGACG00000014550"
##  [4699] "ENSGACG00000015779" "ENSGACG00000015783" "ENSGACG00000014552"
##  [4702] "ENSGACG00000014568" "ENSGACG00000014572" "ENSGACG00000015786"
##  [4705] "ENSGACG00000030619" "ENSGACG00000035631" "ENSGACG00000014575"
##  [4708] "ENSGACG00000014582" "ENSGACG00000030839" "ENSGACG00000015791"
##  [4711] "ENSGACG00000015795" "ENSGACG00000034320" "ENSGACG00000025346"
##  [4714] "ENSGACG00000015799" "ENSGACG00000015802" "ENSGACG00000017639"
##  [4717] "ENSGACG00000036928" "ENSGACG00000031950" "ENSGACG00000034794"
##  [4720] "ENSGACG00000029004" "ENSGACG00000014615" "ENSGACG00000014619"
##  [4723] "ENSGACG00000017645" "ENSGACG00000032222" "ENSGACG00000032918"
##  [4726] "ENSGACG00000014624" "ENSGACG00000014631" "ENSGACG00000014651"
##  [4729] "ENSGACG00000033878" "ENSGACG00000037300" "ENSGACG00000033546"
##  [4732] "ENSGACG00000014659" "ENSGACG00000015811" "ENSGACG00000014311"
##  [4735] "ENSGACG00000031249" "ENSGACG00000037370" "ENSGACG00000014242"
##  [4738] "ENSGACG00000015814" "ENSGACG00000014247" "ENSGACG00000015817"
##  [4741] "ENSGACG00000030402" "ENSGACG00000015819" "ENSGACG00000014257"
##  [4744] "ENSGACG00000014264" "ENSGACG00000014272" "ENSGACG00000014281"
##  [4747] "ENSGACG00000014285" "ENSGACG00000014291" "ENSGACG00000014306"
##  [4750] "ENSGACG00000015821" "ENSGACG00000014301" "ENSGACG00000015823"
##  [4753] "ENSGACG00000015432" "ENSGACG00000015825" "ENSGACG00000014782"
##  [4756] "ENSGACG00000014786" "ENSGACG00000030291" "ENSGACG00000026546"
##  [4759] "ENSGACG00000014795" "ENSGACG00000015829" "ENSGACG00000015830"
##  [4762] "ENSGACG00000026380" "ENSGACG00000014807" "ENSGACG00000015831"
##  [4765] "ENSGACG00000015832" "ENSGACG00000015833" "ENSGACG00000015835"
##  [4768] "ENSGACG00000015838" "ENSGACG00000015840" "ENSGACG00000017613"
##  [4771] "ENSGACG00000015845" "ENSGACG00000015850" "ENSGACG00000015853"
##  [4774] "ENSGACG00000036270" "ENSGACG00000015855" "ENSGACG00000015858"
##  [4777] "ENSGACG00000025115" "ENSGACG00000014815" "ENSGACG00000015863"
##  [4780] "ENSGACG00000014823" "ENSGACG00000015865" "ENSGACG00000015869"
##  [4783] "ENSGACG00000015872" "ENSGACG00000013981" "ENSGACG00000015879"
##  [4786] "ENSGACG00000015714" "ENSGACG00000027665" "ENSGACG00000029230"
##  [4789] "ENSGACG00000029846" "ENSGACG00000033317" "ENSGACG00000031475"
##  [4792] "ENSGACG00000028409" "ENSGACG00000027934" "ENSGACG00000030335"
##  [4795] "ENSGACG00000032689" "ENSGACG00000029718" "ENSGACG00000033146"
##  [4798] "ENSGACG00000027974" "ENSGACG00000037798" "ENSGACG00000024274"
##  [4801] "ENSGACG00000014828" "ENSGACG00000027901" "ENSGACG00000030136"
##  [4804] "ENSGACG00000023807" "ENSGACG00000032062" "ENSGACG00000014832"
##  [4807] "ENSGACG00000014835" "ENSGACG00000033429" "ENSGACG00000037817"
##  [4810] "ENSGACG00000024594" "ENSGACG00000030932" "ENSGACG00000032670"
##  [4813] "ENSGACG00000033200" "ENSGACG00000017169" "ENSGACG00000017171"
##  [4816] "ENSGACG00000033664" "ENSGACG00000014848" "ENSGACG00000017175"
##  [4819] "ENSGACG00000032709" "ENSGACG00000027042" "ENSGACG00000026491"
##  [4822] "ENSGACG00000033379" "ENSGACG00000032726" "ENSGACG00000014856"
##  [4825] "ENSGACG00000029142" "ENSGACG00000026796" "ENSGACG00000024107"
##  [4828] "ENSGACG00000029631" "ENSGACG00000014858" "ENSGACG00000036784"
##  [4831] "ENSGACG00000028793" "ENSGACG00000033596" "ENSGACG00000036245"
##  [4834] "ENSGACG00000026140" "ENSGACG00000032824" "ENSGACG00000026359"
##  [4837] "ENSGACG00000032216" "ENSGACG00000017182" "ENSGACG00000035595"
##  [4840] "ENSGACG00000035483" "ENSGACG00000024202" "ENSGACG00000016327"
##  [4843] "ENSGACG00000031514" "ENSGACG00000025499" "ENSGACG00000016032"
##  [4846] "ENSGACG00000017200" "ENSGACG00000017236" "ENSGACG00000023868"
##  [4849] "ENSGACG00000016476" "ENSGACG00000014864" "ENSGACG00000024354"
##  [4852] "ENSGACG00000037513" "ENSGACG00000015936" "ENSGACG00000014868"
##  [4855] "ENSGACG00000025028" "ENSGACG00000016509" "ENSGACG00000017243"
##  [4858] "ENSGACG00000014873" "ENSGACG00000031292" "ENSGACG00000015871"
##  [4861] "ENSGACG00000017247" "ENSGACG00000014887" "ENSGACG00000014888"
##  [4864] "ENSGACG00000014900" "ENSGACG00000014902" "ENSGACG00000014907"
##  [4867] "ENSGACG00000014918" "ENSGACG00000014920" "ENSGACG00000014922"
##  [4870] "ENSGACG00000014671" "ENSGACG00000014674" "ENSGACG00000014688"
##  [4873] "ENSGACG00000014696" "ENSGACG00000031376" "ENSGACG00000017616"
##  [4876] "ENSGACG00000033308" "ENSGACG00000025663" "ENSGACG00000014702"
##  [4879] "ENSGACG00000014725" "ENSGACG00000014729" "ENSGACG00000028754"
##  [4882] "ENSGACG00000017622" "ENSGACG00000023363" "ENSGACG00000033640"
##  [4885] "ENSGACG00000017631" "ENSGACG00000017634" "ENSGACG00000024768"
##  [4888] "ENSGACG00000024903" "ENSGACG00000017131" "ENSGACG00000014734"
##  [4891] "ENSGACG00000035183" "ENSGACG00000017137" "ENSGACG00000014748"
##  [4894] "ENSGACG00000017142" "ENSGACG00000017149" "ENSGACG00000035278"
##  [4897] "ENSGACG00000015100" "ENSGACG00000026235" "ENSGACG00000017155"
##  [4900] "ENSGACG00000031177" "ENSGACG00000017159" "ENSGACG00000036679"
##  [4903] "ENSGACG00000034401" "ENSGACG00000017162" "ENSGACG00000017165"
##  [4906] "ENSGACG00000033802" "ENSGACG00000015113" "ENSGACG00000015118"
##  [4909] "ENSGACG00000015124" "ENSGACG00000015128" "ENSGACG00000015127"
##  [4912] "ENSGACG00000017167" "ENSGACG00000015131" "ENSGACG00000015133"
##  [4915] "ENSGACG00000015134" "ENSGACG00000036227" "ENSGACG00000015139"
##  [4918] "ENSGACG00000037330" "ENSGACG00000035900" "ENSGACG00000027387"
##  [4921] "ENSGACG00000024039" "ENSGACG00000015147" "ENSGACG00000023992"
##  [4924] "ENSGACG00000017597" "ENSGACG00000015160" "ENSGACG00000015439"
##  [4927] "ENSGACG00000028764" "ENSGACG00000015445" "ENSGACG00000037879"
##  [4930] "ENSGACG00000015447" "ENSGACG00000031644" "ENSGACG00000028777"
##  [4933] "ENSGACG00000035109" "ENSGACG00000025436" "ENSGACG00000023136"
##  [4936] "ENSGACG00000031594" "ENSGACG00000036871" "ENSGACG00000025173"
##  [4939] "ENSGACG00000031432" "ENSGACG00000028811" "ENSGACG00000024629"
##  [4942] "ENSGACG00000026104" "ENSGACG00000023486" "ENSGACG00000024189"
##  [4945] "ENSGACG00000032298" "ENSGACG00000031493" "ENSGACG00000023142"
##  [4948] "ENSGACG00000023388" "ENSGACG00000036171" "ENSGACG00000017547"
##  [4951] "ENSGACG00000017663" "ENSGACG00000029076" "ENSGACG00000015452"
##  [4954] "ENSGACG00000015459" "ENSGACG00000015466" "ENSGACG00000015470"
##  [4957] "ENSGACG00000015478" "ENSGACG00000015483" "ENSGACG00000015484"
##  [4960] "ENSGACG00000026669" "ENSGACG00000029777" "ENSGACG00000015507"
##  [4963] "ENSGACG00000032300" "ENSGACG00000015514" "ENSGACG00000015517"
##  [4966] "ENSGACG00000015161" "ENSGACG00000015164" "ENSGACG00000015167"
##  [4969] "ENSGACG00000015174" "ENSGACG00000015179" "ENSGACG00000015183"
##  [4972] "ENSGACG00000015184" "ENSGACG00000015187" "ENSGACG00000015193"
##  [4975] "ENSGACG00000015200" "ENSGACG00000015204" "ENSGACG00000015208"
##  [4978] "ENSGACG00000015216" "ENSGACG00000015218" "ENSGACG00000032091"
##  [4981] "ENSGACG00000015251" "ENSGACG00000015254" "ENSGACG00000029085"
##  [4984] "ENSGACG00000025795" "ENSGACG00000015260" "ENSGACG00000015595"
##  [4987] "ENSGACG00000015600" "ENSGACG00000030169" "ENSGACG00000015603"
##  [4990] "ENSGACG00000015605" "ENSGACG00000015616" "ENSGACG00000015620"
##  [4993] "ENSGACG00000015626" "ENSGACG00000015632" "ENSGACG00000015635"
##  [4996] "ENSGACG00000026832" "ENSGACG00000015525" "ENSGACG00000015530"
##  [4999] "ENSGACG00000015539" "ENSGACG00000025737" "ENSGACG00000015543"
##  [5002] "ENSGACG00000015544" "ENSGACG00000015547" "ENSGACG00000015550"
##  [5005] "ENSGACG00000015551" "ENSGACG00000023035" "ENSGACG00000015558"
##  [5008] "ENSGACG00000015566" "ENSGACG00000015570" "ENSGACG00000015573"
##  [5011] "ENSGACG00000015578" "ENSGACG00000015592" "ENSGACG00000015275"
##  [5014] "ENSGACG00000037869" "ENSGACG00000015277" "ENSGACG00000015278"
##  [5017] "ENSGACG00000035489" "ENSGACG00000015281" "ENSGACG00000015283"
##  [5020] "ENSGACG00000015292" "ENSGACG00000032101" "ENSGACG00000015297"
##  [5023] "ENSGACG00000015300" "ENSGACG00000015302" "ENSGACG00000015305"
##  [5026] "ENSGACG00000015310" "ENSGACG00000015319" "ENSGACG00000015331"
##  [5029] "ENSGACG00000015337" "ENSGACG00000015338" "ENSGACG00000015345"
##  [5032] "ENSGACG00000015350" "ENSGACG00000015356" "ENSGACG00000015359"
##  [5035] "ENSGACG00000015363" "ENSGACG00000015368" "ENSGACG00000015376"
##  [5038] "ENSGACG00000015374" "ENSGACG00000031108" "ENSGACG00000015378"
##  [5041] "ENSGACG00000037492" "ENSGACG00000015384" "ENSGACG00000034303"
##  [5044] "ENSGACG00000015390" "ENSGACG00000015391" "ENSGACG00000015396"
##  [5047] "ENSGACG00000015403" "ENSGACG00000015417" "ENSGACG00000015419"
##  [5050] "ENSGACG00000015421" "ENSGACG00000027548" "ENSGACG00000015424"
##  [5053] "ENSGACG00000015428" "ENSGACG00000034284" "ENSGACG00000037090"
##  [5056] "ENSGACG00000032921" "ENSGACG00000028571" "ENSGACG00000027025"
##  [5059] "ENSGACG00000014981" "ENSGACG00000014985" "ENSGACG00000014989"
##  [5062] "ENSGACG00000014999" "ENSGACG00000015004" "ENSGACG00000015008"
##  [5065] "ENSGACG00000015010" "ENSGACG00000015018" "ENSGACG00000015021"
##  [5068] "ENSGACG00000015025" "ENSGACG00000015028" "ENSGACG00000015037"
##  [5071] "ENSGACG00000015040" "ENSGACG00000015044" "ENSGACG00000015047"
##  [5074] "ENSGACG00000015051" "ENSGACG00000030137" "ENSGACG00000015053"
##  [5077] "ENSGACG00000015059" "ENSGACG00000015069" "ENSGACG00000015072"
##  [5080] "ENSGACG00000015078" "ENSGACG00000030517" "ENSGACG00000015084"
##  [5083] "ENSGACG00000015091" "ENSGACG00000015093" "ENSGACG00000015096"
##  [5086] "ENSGACG00000015098" "ENSGACG00000012660" "ENSGACG00000012626"
##  [5089] "ENSGACG00000012591" "ENSGACG00000012573" "ENSGACG00000031205"
##  [5092] "ENSGACG00000012549" "ENSGACG00000012531" "ENSGACG00000025292"
##  [5095] "ENSGACG00000012495" "ENSGACG00000012477" "ENSGACG00000036842"
##  [5098] "ENSGACG00000012452" "ENSGACG00000028130" "ENSGACG00000014226"
##  [5101] "ENSGACG00000014217" "ENSGACG00000014211" "ENSGACG00000014187"
##  [5104] "ENSGACG00000031149" "ENSGACG00000014182" "ENSGACG00000014165"
##  [5107] "ENSGACG00000014160" "ENSGACG00000014155" "ENSGACG00000028108"
##  [5110] "ENSGACG00000037268" "ENSGACG00000014146" "ENSGACG00000014130"
##  [5113] "ENSGACG00000014127" "ENSGACG00000026781" "ENSGACG00000014114"
##  [5116] "ENSGACG00000014109" "ENSGACG00000014099" "ENSGACG00000014092"
##  [5119] "ENSGACG00000014089" "ENSGACG00000014084" "ENSGACG00000033722"
##  [5122] "ENSGACG00000014066" "ENSGACG00000013986" "ENSGACG00000013982"
##  [5125] "ENSGACG00000032656" "ENSGACG00000013974" "ENSGACG00000013959"
##  [5128] "ENSGACG00000013947" "ENSGACG00000013938" "ENSGACG00000013926"
##  [5131] "ENSGACG00000014234" "ENSGACG00000037811" "ENSGACG00000026516"
##  [5134] "ENSGACG00000030310" "ENSGACG00000033305" "ENSGACG00000031640"
##  [5137] "ENSGACG00000029568" "ENSGACG00000033192" "ENSGACG00000034682"
##  [5140] "ENSGACG00000031316" "ENSGACG00000028145" "ENSGACG00000029673"
##  [5143] "ENSGACG00000033139" "ENSGACG00000025051" "ENSGACG00000037673"
##  [5146] "ENSGACG00000033478" "ENSGACG00000033575" "ENSGACG00000030627"
##  [5149] "ENSGACG00000025927" "ENSGACG00000030961" "ENSGACG00000025555"
##  [5152] "ENSGACG00000036719" "ENSGACG00000025837" "ENSGACG00000028176"
##  [5155] "ENSGACG00000034633" "ENSGACG00000035062" "ENSGACG00000029550"
##  [5158] "ENSGACG00000028026" "ENSGACG00000034592" "ENSGACG00000025841"
##  [5161] "ENSGACG00000024579" "ENSGACG00000029072" "ENSGACG00000030844"
##  [5164] "ENSGACG00000023514" "ENSGACG00000024979" "ENSGACG00000036801"
##  [5167] "ENSGACG00000027211" "ENSGACG00000031468" "ENSGACG00000024584"
##  [5170] "ENSGACG00000031797" "ENSGACG00000035884" "ENSGACG00000034148"
##  [5173] "ENSGACG00000014706" "ENSGACG00000015140" "ENSGACG00000015375"
##  [5176] "ENSGACG00000014973" "ENSGACG00000028597" "ENSGACG00000012463"
##  [5179] "ENSGACG00000014193" "ENSGACG00000037766" "ENSGACG00000001847"
##  [5182] "ENSGACG00000020021" "ENSGACG00000020014" "ENSGACG00000020011"
##  [5185] "ENSGACG00000020010" "ENSGACG00000020004" "ENSGACG00000020002"
##  [5188] "ENSGACG00000020000" "ENSGACG00000019998" "ENSGACG00000029812"
##  [5191] "ENSGACG00000032418" "ENSGACG00000019994" "ENSGACG00000037748"
##  [5194] "ENSGACG00000019985" "ENSGACG00000019944" "ENSGACG00000019932"
##  [5197] "ENSGACG00000019926" "ENSGACG00000019918" "ENSGACG00000035455"
##  [5200] "ENSGACG00000019916" "ENSGACG00000019915" "ENSGACG00000019910"
##  [5203] "ENSGACG00000019906" "ENSGACG00000019899" "ENSGACG00000032593"
##  [5206] "ENSGACG00000019890" "ENSGACG00000019887" "ENSGACG00000029194"
##  [5209] "ENSGACG00000026450" "ENSGACG00000019882" "ENSGACG00000019878"
##  [5212] "ENSGACG00000031239" "ENSGACG00000033458" "ENSGACG00000019874"
##  [5215] "ENSGACG00000033081" "ENSGACG00000019871" "ENSGACG00000019868"
##  [5218] "ENSGACG00000019856" "ENSGACG00000033191" "ENSGACG00000019854"
##  [5221] "ENSGACG00000029857" "ENSGACG00000023183" "ENSGACG00000033001"
##  [5224] "ENSGACG00000019842" "ENSGACG00000019846" "ENSGACG00000030969"
##  [5227] "ENSGACG00000036163" "ENSGACG00000028157" "ENSGACG00000023360"
##  [5230] "ENSGACG00000025081" "ENSGACG00000034299" "ENSGACG00000033968"
##  [5233] "ENSGACG00000035415" "ENSGACG00000025577" "ENSGACG00000026048"
##  [5236] "ENSGACG00000019940" "ENSGACG00000035044" "ENSGACG00000018401"
##  [5239] "ENSGACG00000018405" "ENSGACG00000018398" "ENSGACG00000018396"
##  [5242] "ENSGACG00000018392" "ENSGACG00000018382" "ENSGACG00000018380"
##  [5245] "ENSGACG00000034353" "ENSGACG00000018377" "ENSGACG00000018376"
##  [5248] "ENSGACG00000018366" "ENSGACG00000018364" "ENSGACG00000032636"
##  [5251] "ENSGACG00000018360" "ENSGACG00000025778" "ENSGACG00000018356"
##  [5254] "ENSGACG00000018347" "ENSGACG00000018346" "ENSGACG00000026725"
##  [5257] "ENSGACG00000018341" "ENSGACG00000032798" "ENSGACG00000018336"
##  [5260] "ENSGACG00000018333" "ENSGACG00000035602" "ENSGACG00000023616"
##  [5263] "ENSGACG00000016775" "ENSGACG00000016774" "ENSGACG00000017387"
##  [5266] "ENSGACG00000017767" "ENSGACG00000017761" "ENSGACG00000017760"
##  [5269] "ENSGACG00000017708" "ENSGACG00000018409" "ENSGACG00000016769"
##  [5272] "ENSGACG00000016767" "ENSGACG00000016762" "ENSGACG00000016758"
##  [5275] "ENSGACG00000028850" "ENSGACG00000016754" "ENSGACG00000016753"
##  [5278] "ENSGACG00000016738" "ENSGACG00000016733" "ENSGACG00000016726"
##  [5281] "ENSGACG00000016723" "ENSGACG00000016711" "ENSGACG00000016708"
##  [5284] "ENSGACG00000016703" "ENSGACG00000016696" "ENSGACG00000016687"
##  [5287] "ENSGACG00000016666" "ENSGACG00000016663" "ENSGACG00000022932"
##  [5290] "ENSGACG00000016657" "ENSGACG00000016636" "ENSGACG00000036345"
##  [5293] "ENSGACG00000033513" "ENSGACG00000016324" "ENSGACG00000016635"
##  [5296] "ENSGACG00000033918" "ENSGACG00000016632" "ENSGACG00000016622"
##  [5299] "ENSGACG00000016328" "ENSGACG00000019454" "ENSGACG00000036529"
##  [5302] "ENSGACG00000034985" "ENSGACG00000029555" "ENSGACG00000001673"
##  [5305] "ENSGACG00000031166" "ENSGACG00000016621" "ENSGACG00000016616"
##  [5308] "ENSGACG00000023930" "ENSGACG00000016610" "ENSGACG00000019448"
##  [5311] "ENSGACG00000019446" "ENSGACG00000025375" "ENSGACG00000016607"
##  [5314] "ENSGACG00000016604" "ENSGACG00000034738" "ENSGACG00000016593"
##  [5317] "ENSGACG00000019442" "ENSGACG00000016589" "ENSGACG00000016585"
##  [5320] "ENSGACG00000019424" "ENSGACG00000037296" "ENSGACG00000016580"
##  [5323] "ENSGACG00000015888" "ENSGACG00000016568" "ENSGACG00000016566"
##  [5326] "ENSGACG00000019421" "ENSGACG00000016561" "ENSGACG00000016535"
##  [5329] "ENSGACG00000019419" "ENSGACG00000019412" "ENSGACG00000015890"
##  [5332] "ENSGACG00000019410" "ENSGACG00000015891" "ENSGACG00000015893"
##  [5335] "ENSGACG00000019407" "ENSGACG00000015899" "ENSGACG00000037466"
##  [5338] "ENSGACG00000016530" "ENSGACG00000024932" "ENSGACG00000017771"
##  [5341] "ENSGACG00000025404" "ENSGACG00000019475" "ENSGACG00000016832"
##  [5344] "ENSGACG00000019469" "ENSGACG00000025046" "ENSGACG00000019462"
##  [5347] "ENSGACG00000016827" "ENSGACG00000036517" "ENSGACG00000016815"
##  [5350] "ENSGACG00000016342" "ENSGACG00000016812" "ENSGACG00000016806"
##  [5353] "ENSGACG00000034105" "ENSGACG00000016361" "ENSGACG00000016364"
##  [5356] "ENSGACG00000016374" "ENSGACG00000016800" "ENSGACG00000016375"
##  [5359] "ENSGACG00000016377" "ENSGACG00000027902" "ENSGACG00000016797"
##  [5362] "ENSGACG00000016792" "ENSGACG00000016383" "ENSGACG00000016785"
##  [5365] "ENSGACG00000016386" "ENSGACG00000034800" "ENSGACG00000016390"
##  [5368] "ENSGACG00000017796" "ENSGACG00000034217" "ENSGACG00000026417"
##  [5371] "ENSGACG00000016412" "ENSGACG00000028210" "ENSGACG00000018066"
##  [5374] "ENSGACG00000016421" "ENSGACG00000018065" "ENSGACG00000018060"
##  [5377] "ENSGACG00000018056" "ENSGACG00000018054" "ENSGACG00000016427"
##  [5380] "ENSGACG00000018051" "ENSGACG00000016432" "ENSGACG00000018047"
##  [5383] "ENSGACG00000016436" "ENSGACG00000018037" "ENSGACG00000018034"
##  [5386] "ENSGACG00000016440" "ENSGACG00000016442" "ENSGACG00000016448"
##  [5389] "ENSGACG00000018031" "ENSGACG00000018028" "ENSGACG00000026015"
##  [5392] "ENSGACG00000018024" "ENSGACG00000016465" "ENSGACG00000030203"
##  [5395] "ENSGACG00000016839" "ENSGACG00000016837" "ENSGACG00000030252"
##  [5398] "ENSGACG00000016475" "ENSGACG00000035661" "ENSGACG00000016482"
##  [5401] "ENSGACG00000018230" "ENSGACG00000016493" "ENSGACG00000018238"
##  [5404] "ENSGACG00000018233" "ENSGACG00000016499" "ENSGACG00000019500"
##  [5407] "ENSGACG00000017494" "ENSGACG00000019492" "ENSGACG00000017492"
##  [5410] "ENSGACG00000017462" "ENSGACG00000032422" "ENSGACG00000017458"
##  [5413] "ENSGACG00000017450" "ENSGACG00000017443" "ENSGACG00000017439"
##  [5416] "ENSGACG00000017429" "ENSGACG00000019491" "ENSGACG00000017412"
##  [5419] "ENSGACG00000019479" "ENSGACG00000033673" "ENSGACG00000019477"
##  [5422] "ENSGACG00000016029" "ENSGACG00000016033" "ENSGACG00000017405"
##  [5425] "ENSGACG00000017401" "ENSGACG00000016526" "ENSGACG00000016520"
##  [5428] "ENSGACG00000016515" "ENSGACG00000016508" "ENSGACG00000032030"
##  [5431] "ENSGACG00000036107" "ENSGACG00000017502" "ENSGACG00000017501"
##  [5434] "ENSGACG00000036097" "ENSGACG00000025965" "ENSGACG00000017804"
##  [5437] "ENSGACG00000032938" "ENSGACG00000018410" "ENSGACG00000018022"
##  [5440] "ENSGACG00000018013" "ENSGACG00000018009" "ENSGACG00000035015"
##  [5443] "ENSGACG00000017994" "ENSGACG00000017985" "ENSGACG00000025337"
##  [5446] "ENSGACG00000029918" "ENSGACG00000017932" "ENSGACG00000016046"
##  [5449] "ENSGACG00000029386" "ENSGACG00000017922" "ENSGACG00000017906"
##  [5452] "ENSGACG00000017903" "ENSGACG00000016047" "ENSGACG00000017900"
##  [5455] "ENSGACG00000017879" "ENSGACG00000017887" "ENSGACG00000033197"
##  [5458] "ENSGACG00000016874" "ENSGACG00000031987" "ENSGACG00000016863"
##  [5461] "ENSGACG00000026623" "ENSGACG00000016861" "ENSGACG00000016857"
##  [5464] "ENSGACG00000016851" "ENSGACG00000016846" "ENSGACG00000017872"
##  [5467] "ENSGACG00000017870" "ENSGACG00000017861" "ENSGACG00000017858"
##  [5470] "ENSGACG00000017850" "ENSGACG00000016048" "ENSGACG00000016052"
##  [5473] "ENSGACG00000016056" "ENSGACG00000016057" "ENSGACG00000016063"
##  [5476] "ENSGACG00000025955" "ENSGACG00000034993" "ENSGACG00000017841"
##  [5479] "ENSGACG00000017840" "ENSGACG00000017826" "ENSGACG00000017809"
##  [5482] "ENSGACG00000033453" "ENSGACG00000017390" "ENSGACG00000018330"
##  [5485] "ENSGACG00000018328" "ENSGACG00000018327" "ENSGACG00000018310"
##  [5488] "ENSGACG00000018303" "ENSGACG00000018297" "ENSGACG00000018295"
##  [5491] "ENSGACG00000033174" "ENSGACG00000018278" "ENSGACG00000018276"
##  [5494] "ENSGACG00000018274" "ENSGACG00000018272" "ENSGACG00000033415"
##  [5497] "ENSGACG00000018262" "ENSGACG00000018261" "ENSGACG00000018247"
##  [5500] "ENSGACG00000018244" "ENSGACG00000017707" "ENSGACG00000017662"
##  [5503] "ENSGACG00000017658" "ENSGACG00000017657" "ENSGACG00000017653"
##  [5506] "ENSGACG00000017646" "ENSGACG00000026123" "ENSGACG00000017633"
##  [5509] "ENSGACG00000017630" "ENSGACG00000022941" "ENSGACG00000017615"
##  [5512] "ENSGACG00000017614" "ENSGACG00000031021" "ENSGACG00000017608"
##  [5515] "ENSGACG00000017601" "ENSGACG00000033039" "ENSGACG00000017587"
##  [5518] "ENSGACG00000031964" "ENSGACG00000030174" "ENSGACG00000027314"
##  [5521] "ENSGACG00000017558" "ENSGACG00000017556" "ENSGACG00000017550"
##  [5524] "ENSGACG00000017555" "ENSGACG00000017548" "ENSGACG00000033112"
##  [5527] "ENSGACG00000017536" "ENSGACG00000017529" "ENSGACG00000017519"
##  [5530] "ENSGACG00000023825" "ENSGACG00000023900" "ENSGACG00000037968"
##  [5533] "ENSGACG00000019717" "ENSGACG00000032982" "ENSGACG00000016504"
##  [5536] "ENSGACG00000028152" "ENSGACG00000019510" "ENSGACG00000019501"
##  [5539] "ENSGACG00000019507" "ENSGACG00000019511" "ENSGACG00000019513"
##  [5542] "ENSGACG00000019516" "ENSGACG00000019526" "ENSGACG00000019529"
##  [5545] "ENSGACG00000019531" "ENSGACG00000025109" "ENSGACG00000024257"
##  [5548] "ENSGACG00000019548" "ENSGACG00000019550" "ENSGACG00000019551"
##  [5551] "ENSGACG00000019552" "ENSGACG00000037144" "ENSGACG00000027399"
##  [5554] "ENSGACG00000019559" "ENSGACG00000034456" "ENSGACG00000019561"
##  [5557] "ENSGACG00000025098" "ENSGACG00000019564" "ENSGACG00000023151"
##  [5560] "ENSGACG00000019567" "ENSGACG00000015912" "ENSGACG00000019571"
##  [5563] "ENSGACG00000015921" "ENSGACG00000019577" "ENSGACG00000019579"
##  [5566] "ENSGACG00000023305" "ENSGACG00000019714" "ENSGACG00000019583"
##  [5569] "ENSGACG00000019584" "ENSGACG00000015922" "ENSGACG00000015925"
##  [5572] "ENSGACG00000019589" "ENSGACG00000016075" "ENSGACG00000019591"
##  [5575] "ENSGACG00000034579" "ENSGACG00000030144" "ENSGACG00000019600"
##  [5578] "ENSGACG00000019603" "ENSGACG00000019730" "ENSGACG00000019729"
##  [5581] "ENSGACG00000019726" "ENSGACG00000032058" "ENSGACG00000019719"
##  [5584] "ENSGACG00000001040" "ENSGACG00000035971" "ENSGACG00000019711"
##  [5587] "ENSGACG00000019707" "ENSGACG00000035803" "ENSGACG00000019695"
##  [5590] "ENSGACG00000019693" "ENSGACG00000019689" "ENSGACG00000019684"
##  [5593] "ENSGACG00000019682" "ENSGACG00000031029" "ENSGACG00000019678"
##  [5596] "ENSGACG00000031038" "ENSGACG00000032558" "ENSGACG00000019669"
##  [5599] "ENSGACG00000036486" "ENSGACG00000015939" "ENSGACG00000033569"
##  [5602] "ENSGACG00000019665" "ENSGACG00000019664" "ENSGACG00000019661"
##  [5605] "ENSGACG00000015944" "ENSGACG00000019642" "ENSGACG00000019641"
##  [5608] "ENSGACG00000026248" "ENSGACG00000032850" "ENSGACG00000031558"
##  [5611] "ENSGACG00000001034" "ENSGACG00000019626" "ENSGACG00000019620"
##  [5614] "ENSGACG00000015946" "ENSGACG00000029519" "ENSGACG00000029022"
##  [5617] "ENSGACG00000015951" "ENSGACG00000015959" "ENSGACG00000019607"
##  [5620] "ENSGACG00000030439" "ENSGACG00000015962" "ENSGACG00000036771"
##  [5623] "ENSGACG00000015968" "ENSGACG00000026840" "ENSGACG00000026595"
##  [5626] "ENSGACG00000019606" "ENSGACG00000015974" "ENSGACG00000032326"
##  [5629] "ENSGACG00000015979" "ENSGACG00000015986" "ENSGACG00000034638"
##  [5632] "ENSGACG00000015988" "ENSGACG00000034244" "ENSGACG00000029432"
##  [5635] "ENSGACG00000019735" "ENSGACG00000029454" "ENSGACG00000019736"
##  [5638] "ENSGACG00000033202" "ENSGACG00000015996" "ENSGACG00000033964"
##  [5641] "ENSGACG00000030865" "ENSGACG00000037708" "ENSGACG00000035581"
##  [5644] "ENSGACG00000016011" "ENSGACG00000016012" "ENSGACG00000037321"
##  [5647] "ENSGACG00000030034" "ENSGACG00000016017" "ENSGACG00000016019"
##  [5650] "ENSGACG00000019739" "ENSGACG00000016026" "ENSGACG00000019749"
##  [5653] "ENSGACG00000019752" "ENSGACG00000036854" "ENSGACG00000019756"
##  [5656] "ENSGACG00000016316" "ENSGACG00000019761" "ENSGACG00000028235"
##  [5659] "ENSGACG00000019769" "ENSGACG00000019771" "ENSGACG00000019775"
##  [5662] "ENSGACG00000001023" "ENSGACG00000033426" "ENSGACG00000023144"
##  [5665] "ENSGACG00000019778" "ENSGACG00000019779" "ENSGACG00000019780"
##  [5668] "ENSGACG00000030119" "ENSGACG00000029450" "ENSGACG00000029010"
##  [5671] "ENSGACG00000000656" "ENSGACG00000000645" "ENSGACG00000019811"
##  [5674] "ENSGACG00000016322" "ENSGACG00000019810" "ENSGACG00000016086"
##  [5677] "ENSGACG00000016087" "ENSGACG00000031706" "ENSGACG00000016093"
##  [5680] "ENSGACG00000016096" "ENSGACG00000016100" "ENSGACG00000019809"
##  [5683] "ENSGACG00000016107" "ENSGACG00000016110" "ENSGACG00000019808"
##  [5686] "ENSGACG00000033084" "ENSGACG00000016112" "ENSGACG00000019798"
##  [5689] "ENSGACG00000019788" "ENSGACG00000019786" "ENSGACG00000025270"
##  [5692] "ENSGACG00000026430" "ENSGACG00000016119" "ENSGACG00000019784"
##  [5695] "ENSGACG00000016135" "ENSGACG00000016136" "ENSGACG00000019782"
##  [5698] "ENSGACG00000016141" "ENSGACG00000016145" "ENSGACG00000016149"
##  [5701] "ENSGACG00000016153" "ENSGACG00000016165" "ENSGACG00000016178"
##  [5704] "ENSGACG00000036087" "ENSGACG00000029075" "ENSGACG00000016184"
##  [5707] "ENSGACG00000016187" "ENSGACG00000027546" "ENSGACG00000002054"
##  [5710] "ENSGACG00000033867" "ENSGACG00000028915" "ENSGACG00000027620"
##  [5713] "ENSGACG00000025672" "ENSGACG00000032487" "ENSGACG00000000196"
##  [5716] "ENSGACG00000019815" "ENSGACG00000019818" "ENSGACG00000024904"
##  [5719] "ENSGACG00000016196" "ENSGACG00000031289" "ENSGACG00000035033"
##  [5722] "ENSGACG00000037978" "ENSGACG00000036884" "ENSGACG00000017375"
##  [5725] "ENSGACG00000017371" "ENSGACG00000017366" "ENSGACG00000017347"
##  [5728] "ENSGACG00000017342" "ENSGACG00000017335" "ENSGACG00000017328"
##  [5731] "ENSGACG00000017322" "ENSGACG00000017317" "ENSGACG00000017308"
##  [5734] "ENSGACG00000030858" "ENSGACG00000017291" "ENSGACG00000017277"
##  [5737] "ENSGACG00000017275" "ENSGACG00000017274" "ENSGACG00000017271"
##  [5740] "ENSGACG00000017255" "ENSGACG00000017250" "ENSGACG00000017248"
##  [5743] "ENSGACG00000017238" "ENSGACG00000031567" "ENSGACG00000016204"
##  [5746] "ENSGACG00000031491" "ENSGACG00000017233" "ENSGACG00000029128"
##  [5749] "ENSGACG00000016213" "ENSGACG00000016219" "ENSGACG00000016223"
##  [5752] "ENSGACG00000027828" "ENSGACG00000017231" "ENSGACG00000016229"
##  [5755] "ENSGACG00000017230" "ENSGACG00000024243" "ENSGACG00000016232"
##  [5758] "ENSGACG00000016248" "ENSGACG00000017228" "ENSGACG00000017225"
##  [5761] "ENSGACG00000017223" "ENSGACG00000036102" "ENSGACG00000017219"
##  [5764] "ENSGACG00000017214" "ENSGACG00000017206" "ENSGACG00000016251"
##  [5767] "ENSGACG00000017201" "ENSGACG00000017193" "ENSGACG00000037947"
##  [5770] "ENSGACG00000017187" "ENSGACG00000016278" "ENSGACG00000016255"
##  [5773] "ENSGACG00000016263" "ENSGACG00000016265" "ENSGACG00000016266"
##  [5776] "ENSGACG00000033801" "ENSGACG00000016272" "ENSGACG00000016274"
##  [5779] "ENSGACG00000034635" "ENSGACG00000016291" "ENSGACG00000033698"
##  [5782] "ENSGACG00000029431" "ENSGACG00000019200" "ENSGACG00000019195"
##  [5785] "ENSGACG00000019191" "ENSGACG00000019187" "ENSGACG00000019181"
##  [5788] "ENSGACG00000036857" "ENSGACG00000019175" "ENSGACG00000019172"
##  [5791] "ENSGACG00000017172" "ENSGACG00000037973" "ENSGACG00000032564"
##  [5794] "ENSGACG00000019168" "ENSGACG00000017164" "ENSGACG00000017147"
##  [5797] "ENSGACG00000017145" "ENSGACG00000028854" "ENSGACG00000024501"
##  [5800] "ENSGACG00000017134" "ENSGACG00000017136" "ENSGACG00000017133"
##  [5803] "ENSGACG00000017130" "ENSGACG00000017124" "ENSGACG00000017099"
##  [5806] "ENSGACG00000017044" "ENSGACG00000019165" "ENSGACG00000019164"
##  [5809] "ENSGACG00000019162" "ENSGACG00000017039" "ENSGACG00000019158"
##  [5812] "ENSGACG00000019157" "ENSGACG00000019154" "ENSGACG00000019151"
##  [5815] "ENSGACG00000017035" "ENSGACG00000017033" "ENSGACG00000016990"
##  [5818] "ENSGACG00000016989" "ENSGACG00000016987" "ENSGACG00000019149"
##  [5821] "ENSGACG00000016984" "ENSGACG00000016968" "ENSGACG00000019146"
##  [5824] "ENSGACG00000019144" "ENSGACG00000019143" "ENSGACG00000036508"
##  [5827] "ENSGACG00000016964" "ENSGACG00000035463" "ENSGACG00000019135"
##  [5830] "ENSGACG00000019131" "ENSGACG00000019122" "ENSGACG00000016962"
##  [5833] "ENSGACG00000016960" "ENSGACG00000016955" "ENSGACG00000019112"
##  [5836] "ENSGACG00000016951" "ENSGACG00000019109" "ENSGACG00000016941"
##  [5839] "ENSGACG00000016293" "ENSGACG00000016303" "ENSGACG00000016306"
##  [5842] "ENSGACG00000016310" "ENSGACG00000019106" "ENSGACG00000016899"
##  [5845] "ENSGACG00000019103" "ENSGACG00000019100" "ENSGACG00000019097"
##  [5848] "ENSGACG00000019093" "ENSGACG00000019091" "ENSGACG00000019074"
##  [5851] "ENSGACG00000019065" "ENSGACG00000019062" "ENSGACG00000019060"
##  [5854] "ENSGACG00000019059" "ENSGACG00000030512" "ENSGACG00000019054"
##  [5857] "ENSGACG00000019406" "ENSGACG00000023601" "ENSGACG00000019401"
##  [5860] "ENSGACG00000016888" "ENSGACG00000019397" "ENSGACG00000016881"
##  [5863] "ENSGACG00000027463" "ENSGACG00000028635" "ENSGACG00000019394"
##  [5866] "ENSGACG00000036126" "ENSGACG00000018228" "ENSGACG00000036256"
##  [5869] "ENSGACG00000019385" "ENSGACG00000019382" "ENSGACG00000029580"
##  [5872] "ENSGACG00000019372" "ENSGACG00000019374" "ENSGACG00000018226"
##  [5875] "ENSGACG00000026467" "ENSGACG00000035506" "ENSGACG00000018223"
##  [5878] "ENSGACG00000019359" "ENSGACG00000018222" "ENSGACG00000018220"
##  [5881] "ENSGACG00000035493" "ENSGACG00000019353" "ENSGACG00000036167"
##  [5884] "ENSGACG00000018216" "ENSGACG00000019351" "ENSGACG00000018215"
##  [5887] "ENSGACG00000018212" "ENSGACG00000019335" "ENSGACG00000018210"
##  [5890] "ENSGACG00000031237" "ENSGACG00000018202" "ENSGACG00000018198"
##  [5893] "ENSGACG00000018197" "ENSGACG00000019332" "ENSGACG00000036506"
##  [5896] "ENSGACG00000018193" "ENSGACG00000025686" "ENSGACG00000034904"
##  [5899] "ENSGACG00000035024" "ENSGACG00000019317" "ENSGACG00000018185"
##  [5902] "ENSGACG00000023768" "ENSGACG00000035677" "ENSGACG00000025773"
##  [5905] "ENSGACG00000018178" "ENSGACG00000034575" "ENSGACG00000018170"
##  [5908] "ENSGACG00000018168" "ENSGACG00000019310" "ENSGACG00000018163"
##  [5911] "ENSGACG00000019303" "ENSGACG00000018160" "ENSGACG00000018155"
##  [5914] "ENSGACG00000019298" "ENSGACG00000037173" "ENSGACG00000018153"
##  [5917] "ENSGACG00000018151" "ENSGACG00000018135" "ENSGACG00000018128"
##  [5920] "ENSGACG00000035191" "ENSGACG00000031682" "ENSGACG00000018124"
##  [5923] "ENSGACG00000029416" "ENSGACG00000018120" "ENSGACG00000018114"
##  [5926] "ENSGACG00000018112" "ENSGACG00000018110" "ENSGACG00000018108"
##  [5929] "ENSGACG00000018104" "ENSGACG00000018097" "ENSGACG00000018090"
##  [5932] "ENSGACG00000018088" "ENSGACG00000018071" "ENSGACG00000018430"
##  [5935] "ENSGACG00000018426" "ENSGACG00000018416" "ENSGACG00000027860"
##  [5938] "ENSGACG00000026057" "ENSGACG00000038137" "ENSGACG00000035449"
##  [5941] "ENSGACG00000032518" "ENSGACG00000035239" "ENSGACG00000028771"
##  [5944] "ENSGACG00000031312" "ENSGACG00000032987" "ENSGACG00000024801"
##  [5947] "ENSGACG00000027559" "ENSGACG00000029778" "ENSGACG00000036286"
##  [5950] "ENSGACG00000029799" "ENSGACG00000026079" "ENSGACG00000037347"
##  [5953] "ENSGACG00000029688" "ENSGACG00000024077" "ENSGACG00000030140"
##  [5956] "ENSGACG00000026054" "ENSGACG00000038010" "ENSGACG00000025002"
##  [5959] "ENSGACG00000030259" "ENSGACG00000033000" "ENSGACG00000031355"
##  [5962] "ENSGACG00000029577" "ENSGACG00000031159" "ENSGACG00000034445"
##  [5965] "ENSGACG00000035049" "ENSGACG00000034563" "ENSGACG00000026216"
##  [5968] "ENSGACG00000031280" "ENSGACG00000023068" "ENSGACG00000033738"
##  [5971] "ENSGACG00000029792" "ENSGACG00000035801" "ENSGACG00000027532"
##  [5974] "ENSGACG00000025420" "ENSGACG00000028790" "ENSGACG00000024697"
##  [5977] "ENSGACG00000028120" "ENSGACG00000034966" "ENSGACG00000030479"
##  [5980] "ENSGACG00000033688" "ENSGACG00000022909" "ENSGACG00000026266"
##  [5983] "ENSGACG00000028420" "ENSGACG00000031153" "ENSGACG00000030753"
##  [5986] "ENSGACG00000025113" "ENSGACG00000016638" "ENSGACG00000037033"
##  [5989] "ENSGACG00000018299" "ENSGACG00000018321" "ENSGACG00000031505"
##  [5992] "ENSGACG00000031096" "ENSGACG00000028699" "ENSGACG00000019296"
##  [5995] "ENSGACG00000019290" "ENSGACG00000025537" "ENSGACG00000001045"
##  [5998] "ENSGACG00000019523" "ENSGACG00000019284" "ENSGACG00000034564"
##  [6001] "ENSGACG00000027560" "ENSGACG00000001748" "ENSGACG00000017143"
##  [6004] "ENSGACG00000018208" "ENSGACG00000019283" "ENSGACG00000026502"
##  [6007] "ENSGACG00000019278" "ENSGACG00000030895" "ENSGACG00000019272"
##  [6010] "ENSGACG00000019268" "ENSGACG00000030676" "ENSGACG00000019260"
##  [6013] "ENSGACG00000019256" "ENSGACG00000026914" "ENSGACG00000019254"
##  [6016] "ENSGACG00000035954" "ENSGACG00000029046" "ENSGACG00000019246"
##  [6019] "ENSGACG00000019245" "ENSGACG00000033468" "ENSGACG00000019237"
##  [6022] "ENSGACG00000019235" "ENSGACG00000019231" "ENSGACG00000025647"
##  [6025] "ENSGACG00000019228" "ENSGACG00000019224" "ENSGACG00000038081"
##  [6028] "ENSGACG00000038007" "ENSGACG00000027071" "ENSGACG00000027563"
##  [6031] "ENSGACG00000019209" "ENSGACG00000019048" "ENSGACG00000019047"
##  [6034] "ENSGACG00000019046" "ENSGACG00000019043" "ENSGACG00000028708"
##  [6037] "ENSGACG00000037792" "ENSGACG00000019035" "ENSGACG00000019032"
##  [6040] "ENSGACG00000019015" "ENSGACG00000029980" "ENSGACG00000019010"
##  [6043] "ENSGACG00000019006" "ENSGACG00000034324" "ENSGACG00000019004"
##  [6046] "ENSGACG00000030163" "ENSGACG00000028714" "ENSGACG00000018999"
##  [6049] "ENSGACG00000018995" "ENSGACG00000018994" "ENSGACG00000035603"
##  [6052] "ENSGACG00000018992" "ENSGACG00000018989" "ENSGACG00000018987"
##  [6055] "ENSGACG00000036670" "ENSGACG00000018968" "ENSGACG00000018966"
##  [6058] "ENSGACG00000018961" "ENSGACG00000018952" "ENSGACG00000018946"
##  [6061] "ENSGACG00000018944" "ENSGACG00000018941" "ENSGACG00000034046"
##  [6064] "ENSGACG00000018937" "ENSGACG00000018936" "ENSGACG00000018928"
##  [6067] "ENSGACG00000018925" "ENSGACG00000025117" "ENSGACG00000018920"
##  [6070] "ENSGACG00000018915" "ENSGACG00000018906" "ENSGACG00000026545"
##  [6073] "ENSGACG00000018901" "ENSGACG00000035092" "ENSGACG00000018895"
##  [6076] "ENSGACG00000018890" "ENSGACG00000018889" "ENSGACG00000035203"
##  [6079] "ENSGACG00000035370" "ENSGACG00000025273" "ENSGACG00000035004"
##  [6082] "ENSGACG00000018871" "ENSGACG00000018868" "ENSGACG00000018863"
##  [6085] "ENSGACG00000018859" "ENSGACG00000025329" "ENSGACG00000027181"
##  [6088] "ENSGACG00000018837" "ENSGACG00000037997" "ENSGACG00000018832"
##  [6091] "ENSGACG00000027830" "ENSGACG00000018810" "ENSGACG00000018800"
##  [6094] "ENSGACG00000037896" "ENSGACG00000018797" "ENSGACG00000018792"
##  [6097] "ENSGACG00000035814" "ENSGACG00000018790" "ENSGACG00000018787"
##  [6100] "ENSGACG00000027694" "ENSGACG00000026178" "ENSGACG00000018780"
##  [6103] "ENSGACG00000018778" "ENSGACG00000018777" "ENSGACG00000018776"
##  [6106] "ENSGACG00000018771" "ENSGACG00000018773" "ENSGACG00000018768"
##  [6109] "ENSGACG00000029253" "ENSGACG00000018758" "ENSGACG00000018754"
##  [6112] "ENSGACG00000018747" "ENSGACG00000018744" "ENSGACG00000026607"
##  [6115] "ENSGACG00000036480" "ENSGACG00000018741" "ENSGACG00000030320"
##  [6118] "ENSGACG00000035619" "ENSGACG00000031068" "ENSGACG00000018721"
##  [6121] "ENSGACG00000018717" "ENSGACG00000018716" "ENSGACG00000025463"
##  [6124] "ENSGACG00000018713" "ENSGACG00000018708" "ENSGACG00000032042"
##  [6127] "ENSGACG00000018691" "ENSGACG00000024004" "ENSGACG00000018688"
##  [6130] "ENSGACG00000018686" "ENSGACG00000018681" "ENSGACG00000001260"
##  [6133] "ENSGACG00000018676" "ENSGACG00000036425" "ENSGACG00000025983"
##  [6136] "ENSGACG00000018670" "ENSGACG00000018666" "ENSGACG00000018657"
##  [6139] "ENSGACG00000018653" "ENSGACG00000035027" "ENSGACG00000018627"
##  [6142] "ENSGACG00000018622" "ENSGACG00000018621" "ENSGACG00000018612"
##  [6145] "ENSGACG00000018609" "ENSGACG00000018604" "ENSGACG00000018603"
##  [6148] "ENSGACG00000018597" "ENSGACG00000018592" "ENSGACG00000018589"
##  [6151] "ENSGACG00000018583" "ENSGACG00000029957" "ENSGACG00000018581"
##  [6154] "ENSGACG00000018576" "ENSGACG00000033937" "ENSGACG00000018568"
##  [6157] "ENSGACG00000028490" "ENSGACG00000025441" "ENSGACG00000018535"
##  [6160] "ENSGACG00000018530" "ENSGACG00000018528" "ENSGACG00000018521"
##  [6163] "ENSGACG00000030556" "ENSGACG00000018519" "ENSGACG00000018518"
##  [6166] "ENSGACG00000018516" "ENSGACG00000018513" "ENSGACG00000018507"
##  [6169] "ENSGACG00000029832" "ENSGACG00000018504" "ENSGACG00000031946"
##  [6172] "ENSGACG00000018497" "ENSGACG00000030059" "ENSGACG00000018491"
##  [6175] "ENSGACG00000018488" "ENSGACG00000018481" "ENSGACG00000018479"
##  [6178] "ENSGACG00000032911" "ENSGACG00000023480" "ENSGACG00000018473"
##  [6181] "ENSGACG00000026715" "ENSGACG00000018469" "ENSGACG00000018465"
##  [6184] "ENSGACG00000030246" "ENSGACG00000018459" "ENSGACG00000018454"
##  [6187] "ENSGACG00000018452" "ENSGACG00000018451" "ENSGACG00000018448"
##  [6190] "ENSGACG00000018436" "ENSGACG00000018434" "ENSGACG00000029964"
##  [6193] "ENSGACG00000016115" "ENSGACG00000034883" "ENSGACG00000023727"
##  [6196] "ENSGACG00000033214" "ENSGACG00000036591" "ENSGACG00000029487"
##  [6199] "ENSGACG00000033254" "ENSGACG00000023336" "ENSGACG00000029997"
##  [6202] "ENSGACG00000025191" "ENSGACG00000035475" "ENSGACG00000026641"
##  [6205] "ENSGACG00000030995" "ENSGACG00000034759" "ENSGACG00000030885"
##  [6208] "ENSGACG00000035327" "ENSGACG00000025571" "ENSGACG00000036134"
##  [6211] "ENSGACG00000023236" "ENSGACG00000030913" "ENSGACG00000029185"
##  [6214] "ENSGACG00000027677" "ENSGACG00000028119" "ENSGACG00000027917"
##  [6217] "ENSGACG00000037608" "ENSGACG00000032633" "ENSGACG00000033871"
##  [6220] "ENSGACG00000032888" "ENSGACG00000037232" "ENSGACG00000026035"
##  [6223] "ENSGACG00000023794" "ENSGACG00000034947" "ENSGACG00000028227"
##  [6226] "ENSGACG00000025659" "ENSGACG00000032970" "ENSGACG00000026930"
##  [6229] "ENSGACG00000024654" "ENSGACG00000033928" "ENSGACG00000026387"
##  [6232] "ENSGACG00000029468" "ENSGACG00000035887" "ENSGACG00000022965"
##  [6235] "ENSGACG00000028365" "ENSGACG00000036200" "ENSGACG00000024700"
##  [6238] "ENSGACG00000027921" "ENSGACG00000036736" "ENSGACG00000035035"
##  [6241] "ENSGACG00000036191" "ENSGACG00000034572" "ENSGACG00000032215"
##  [6244] "ENSGACG00000027689" "ENSGACG00000030495" "ENSGACG00000034984"
##  [6247] "ENSGACG00000037163" "ENSGACG00000024199" "ENSGACG00000026788"
##  [6250] "ENSGACG00000027977" "ENSGACG00000028069" "ENSGACG00000025524"
##  [6253] "ENSGACG00000033470" "ENSGACG00000035910" "ENSGACG00000015905"
##  [6256] "ENSGACG00000015927" "ENSGACG00000016125" "ENSGACG00000016201"
##  [6259] "ENSGACG00000019388" "ENSGACG00000019376" "ENSGACG00000018990"
##  [6262] "ENSGACG00000018563" "ENSGACG00000018500" "ENSGACG00000018506"
##  [6265] "ENSGACG00000001081" "ENSGACG00000001078" "ENSGACG00000001076"
##  [6268] "ENSGACG00000028055" "ENSGACG00000023620" "ENSGACG00000033459"
##  [6271] "ENSGACG00000001066" "ENSGACG00000001064" "ENSGACG00000001062"
##  [6274] "ENSGACG00000010613" "ENSGACG00000010626" "ENSGACG00000023016"
##  [6277] "ENSGACG00000032397" "ENSGACG00000010651" "ENSGACG00000010655"
##  [6280] "ENSGACG00000031318" "ENSGACG00000028017" "ENSGACG00000010691"
##  [6283] "ENSGACG00000010695" "ENSGACG00000010699" "ENSGACG00000010710"
##  [6286] "ENSGACG00000010735" "ENSGACG00000010748" "ENSGACG00000010759"
##  [6289] "ENSGACG00000030288" "ENSGACG00000035600" "ENSGACG00000029427"
##  [6292] "ENSGACG00000010772" "ENSGACG00000035779" "ENSGACG00000013503"
##  [6295] "ENSGACG00000013512" "ENSGACG00000001124" "ENSGACG00000001108"
##  [6298] "ENSGACG00000026459" "ENSGACG00000001103" "ENSGACG00000001098"
##  [6301] "ENSGACG00000028888" "ENSGACG00000032072" "ENSGACG00000023707"
##  [6304] "ENSGACG00000001092" "ENSGACG00000001091" "ENSGACG00000024503"
##  [6307] "ENSGACG00000028776" "ENSGACG00000034297" "ENSGACG00000025494"
##  [6310] "ENSGACG00000033571" "ENSGACG00000031019" "ENSGACG00000024550"
##  [6313] "ENSGACG00000030906" "ENSGACG00000010214" "ENSGACG00000032583"
##  [6316] "ENSGACG00000012817" "ENSGACG00000012837" "ENSGACG00000007298"
##  [6319] "ENSGACG00000030520" "ENSGACG00000007315" "ENSGACG00000007321"
##  [6322] "ENSGACG00000007327" "ENSGACG00000035748" "ENSGACG00000003912"
##  [6325] "ENSGACG00000003906" "ENSGACG00000026293" "ENSGACG00000003881"
##  [6328] "ENSGACG00000003876" "ENSGACG00000036414" "ENSGACG00000029361"
##  [6331] "ENSGACG00000024508" "ENSGACG00000003872" "ENSGACG00000009085"
##  [6334] "ENSGACG00000012851" "ENSGACG00000009100" "ENSGACG00000009114"
##  [6337] "ENSGACG00000034975" "ENSGACG00000009136" "ENSGACG00000004629"
##  [6340] "ENSGACG00000009001" "ENSGACG00000003569" "ENSGACG00000003560"
##  [6343] "ENSGACG00000009149" "ENSGACG00000030586" "ENSGACG00000009157"
##  [6346] "ENSGACG00000009166" "ENSGACG00000009196" "ENSGACG00000009207"
##  [6349] "ENSGACG00000010212" "ENSGACG00000027530" "ENSGACG00000012861"
##  [6352] "ENSGACG00000012865" "ENSGACG00000029308" "ENSGACG00000012807"
##  [6355] "ENSGACG00000011999" "ENSGACG00000012009" "ENSGACG00000012015"
##  [6358] "ENSGACG00000031931" "ENSGACG00000009215" "ENSGACG00000012019"
##  [6361] "ENSGACG00000009222" "ENSGACG00000034412" "ENSGACG00000012060"
##  [6364] "ENSGACG00000012089" "ENSGACG00000012112" "ENSGACG00000035235"
##  [6367] "ENSGACG00000012181" "ENSGACG00000012194" "ENSGACG00000012207"
##  [6370] "ENSGACG00000023003" "ENSGACG00000012220" "ENSGACG00000012233"
##  [6373] "ENSGACG00000011514" "ENSGACG00000010939" "ENSGACG00000010986"
##  [6376] "ENSGACG00000010993" "ENSGACG00000024945" "ENSGACG00000011014"
##  [6379] "ENSGACG00000011026" "ENSGACG00000011027" "ENSGACG00000032389"
##  [6382] "ENSGACG00000011041" "ENSGACG00000011049" "ENSGACG00000011056"
##  [6385] "ENSGACG00000011068" "ENSGACG00000024612" "ENSGACG00000011078"
##  [6388] "ENSGACG00000011085" "ENSGACG00000011111" "ENSGACG00000011126"
##  [6391] "ENSGACG00000023441" "ENSGACG00000025037" "ENSGACG00000011169"
##  [6394] "ENSGACG00000011172" "ENSGACG00000011184" "ENSGACG00000011889"
##  [6397] "ENSGACG00000011893" "ENSGACG00000031739" "ENSGACG00000011923"
##  [6400] "ENSGACG00000011932" "ENSGACG00000011927" "ENSGACG00000011938"
##  [6403] "ENSGACG00000011948" "ENSGACG00000011954" "ENSGACG00000011961"
##  [6406] "ENSGACG00000023370" "ENSGACG00000011979" "ENSGACG00000011877"
##  [6409] "ENSGACG00000029761" "ENSGACG00000027870" "ENSGACG00000012679"
##  [6412] "ENSGACG00000030501" "ENSGACG00000012710" "ENSGACG00000012715"
##  [6415] "ENSGACG00000032601" "ENSGACG00000026540" "ENSGACG00000012778"
##  [6418] "ENSGACG00000012789" "ENSGACG00000011299" "ENSGACG00000003539"
##  [6421] "ENSGACG00000011308" "ENSGACG00000011334" "ENSGACG00000027574"
##  [6424] "ENSGACG00000003520" "ENSGACG00000028030" "ENSGACG00000011374"
##  [6427] "ENSGACG00000003512" "ENSGACG00000003503" "ENSGACG00000003471"
##  [6430] "ENSGACG00000011380" "ENSGACG00000003456" "ENSGACG00000003446"
##  [6433] "ENSGACG00000003433" "ENSGACG00000003432" "ENSGACG00000003381"
##  [6436] "ENSGACG00000003353" "ENSGACG00000013606" "ENSGACG00000033035"
##  [6439] "ENSGACG00000011546" "ENSGACG00000011553" "ENSGACG00000012903"
##  [6442] "ENSGACG00000012921" "ENSGACG00000023450" "ENSGACG00000003335"
##  [6445] "ENSGACG00000012934" "ENSGACG00000036143" "ENSGACG00000012949"
##  [6448] "ENSGACG00000012952" "ENSGACG00000012965" "ENSGACG00000012975"
##  [6451] "ENSGACG00000012977" "ENSGACG00000013003" "ENSGACG00000013011"
##  [6454] "ENSGACG00000013061" "ENSGACG00000013057" "ENSGACG00000013062"
##  [6457] "ENSGACG00000023240" "ENSGACG00000013076" "ENSGACG00000013088"
##  [6460] "ENSGACG00000030284" "ENSGACG00000013106" "ENSGACG00000013109"
##  [6463] "ENSGACG00000036630" "ENSGACG00000033639" "ENSGACG00000008787"
##  [6466] "ENSGACG00000008801" "ENSGACG00000023205" "ENSGACG00000003326"
##  [6469] "ENSGACG00000003313" "ENSGACG00000003309" "ENSGACG00000029813"
##  [6472] "ENSGACG00000003228" "ENSGACG00000003927" "ENSGACG00000003986"
##  [6475] "ENSGACG00000023573" "ENSGACG00000003989" "ENSGACG00000028743"
##  [6478] "ENSGACG00000004060" "ENSGACG00000033831" "ENSGACG00000004070"
##  [6481] "ENSGACG00000004114" "ENSGACG00000004119" "ENSGACG00000004136"
##  [6484] "ENSGACG00000009265" "ENSGACG00000010413" "ENSGACG00000037186"
##  [6487] "ENSGACG00000010472" "ENSGACG00000034748" "ENSGACG00000036913"
##  [6490] "ENSGACG00000004146" "ENSGACG00000010433" "ENSGACG00000004160"
##  [6493] "ENSGACG00000004169" "ENSGACG00000011411" "ENSGACG00000010462"
##  [6496] "ENSGACG00000024968" "ENSGACG00000011448" "ENSGACG00000010487"
##  [6499] "ENSGACG00000009294" "ENSGACG00000009302" "ENSGACG00000004212"
##  [6502] "ENSGACG00000004218" "ENSGACG00000004226" "ENSGACG00000004240"
##  [6505] "ENSGACG00000004263" "ENSGACG00000004269" "ENSGACG00000004293"
##  [6508] "ENSGACG00000004300" "ENSGACG00000004374" "ENSGACG00000004390"
##  [6511] "ENSGACG00000004398" "ENSGACG00000004420" "ENSGACG00000011474"
##  [6514] "ENSGACG00000004447" "ENSGACG00000004452" "ENSGACG00000037177"
##  [6517] "ENSGACG00000010567" "ENSGACG00000026469" "ENSGACG00000004529"
##  [6520] "ENSGACG00000010571" "ENSGACG00000010576" "ENSGACG00000004543"
##  [6523] "ENSGACG00000013189" "ENSGACG00000024794" "ENSGACG00000013231"
##  [6526] "ENSGACG00000013234" "ENSGACG00000013273" "ENSGACG00000013280"
##  [6529] "ENSGACG00000013283" "ENSGACG00000013291" "ENSGACG00000013302"
##  [6532] "ENSGACG00000013294" "ENSGACG00000004583" "ENSGACG00000013316"
##  [6535] "ENSGACG00000004614" "ENSGACG00000033027" "ENSGACG00000004627"
##  [6538] "ENSGACG00000008581" "ENSGACG00000010584" "ENSGACG00000035660"
##  [6541] "ENSGACG00000010595" "ENSGACG00000013323" "ENSGACG00000029845"
##  [6544] "ENSGACG00000033475" "ENSGACG00000013341" "ENSGACG00000013355"
##  [6547] "ENSGACG00000013386" "ENSGACG00000024944" "ENSGACG00000013410"
##  [6550] "ENSGACG00000004641" "ENSGACG00000013417" "ENSGACG00000004678"
##  [6553] "ENSGACG00000004685" "ENSGACG00000004702" "ENSGACG00000023324"
##  [6556] "ENSGACG00000004715" "ENSGACG00000028236" "ENSGACG00000004719"
##  [6559] "ENSGACG00000035575" "ENSGACG00000004723" "ENSGACG00000033824"
##  [6562] "ENSGACG00000027338" "ENSGACG00000013431" "ENSGACG00000004754"
##  [6565] "ENSGACG00000013436" "ENSGACG00000004838" "ENSGACG00000013441"
##  [6568] "ENSGACG00000013448" "ENSGACG00000004853" "ENSGACG00000028750"
##  [6571] "ENSGACG00000013463" "ENSGACG00000013469" "ENSGACG00000013477"
##  [6574] "ENSGACG00000004909" "ENSGACG00000013491" "ENSGACG00000004921"
##  [6577] "ENSGACG00000013522" "ENSGACG00000013531" "ENSGACG00000029303"
##  [6580] "ENSGACG00000032783" "ENSGACG00000008603" "ENSGACG00000013534"
##  [6583] "ENSGACG00000008626" "ENSGACG00000008631" "ENSGACG00000008633"
##  [6586] "ENSGACG00000008660" "ENSGACG00000013548" "ENSGACG00000008696"
##  [6589] "ENSGACG00000013560" "ENSGACG00000035177" "ENSGACG00000008705"
##  [6592] "ENSGACG00000008716" "ENSGACG00000008754" "ENSGACG00000008769"
##  [6595] "ENSGACG00000006722" "ENSGACG00000006728" "ENSGACG00000013543"
##  [6598] "ENSGACG00000034201" "ENSGACG00000010604" "ENSGACG00000011510"
##  [6601] "ENSGACG00000006769" "ENSGACG00000011195" "ENSGACG00000030987"
##  [6604] "ENSGACG00000006791" "ENSGACG00000011206" "ENSGACG00000006793"
##  [6607] "ENSGACG00000011213" "ENSGACG00000006815" "ENSGACG00000011255"
##  [6610] "ENSGACG00000026925" "ENSGACG00000006824" "ENSGACG00000006838"
##  [6613] "ENSGACG00000006842" "ENSGACG00000006857" "ENSGACG00000006860"
##  [6616] "ENSGACG00000006892" "ENSGACG00000006905" "ENSGACG00000006913"
##  [6619] "ENSGACG00000026899" "ENSGACG00000011263" "ENSGACG00000012240"
##  [6622] "ENSGACG00000006939" "ENSGACG00000012244" "ENSGACG00000006978"
##  [6625] "ENSGACG00000012305" "ENSGACG00000006992" "ENSGACG00000012316"
##  [6628] "ENSGACG00000007012" "ENSGACG00000007029" "ENSGACG00000012328"
##  [6631] "ENSGACG00000007043" "ENSGACG00000007048" "ENSGACG00000012350"
##  [6634] "ENSGACG00000032922" "ENSGACG00000031522" "ENSGACG00000012376"
##  [6637] "ENSGACG00000012399" "ENSGACG00000012410" "ENSGACG00000007072"
##  [6640] "ENSGACG00000033864" "ENSGACG00000007106" "ENSGACG00000007111"
##  [6643] "ENSGACG00000007118" "ENSGACG00000037244" "ENSGACG00000007167"
##  [6646] "ENSGACG00000007191" "ENSGACG00000029808" "ENSGACG00000012418"
##  [6649] "ENSGACG00000031162" "ENSGACG00000023047" "ENSGACG00000012438"
##  [6652] "ENSGACG00000012454" "ENSGACG00000012469" "ENSGACG00000012479"
##  [6655] "ENSGACG00000012483" "ENSGACG00000007218" "ENSGACG00000007201"
##  [6658] "ENSGACG00000007252" "ENSGACG00000007279" "ENSGACG00000007292"
##  [6661] "ENSGACG00000012502" "ENSGACG00000032566" "ENSGACG00000012516"
##  [6664] "ENSGACG00000006670" "ENSGACG00000012522" "ENSGACG00000029691"
##  [6667] "ENSGACG00000006692" "ENSGACG00000012542" "ENSGACG00000006715"
##  [6670] "ENSGACG00000008560" "ENSGACG00000028091" "ENSGACG00000009902"
##  [6673] "ENSGACG00000012563" "ENSGACG00000009905" "ENSGACG00000012577"
##  [6676] "ENSGACG00000012596" "ENSGACG00000012602" "ENSGACG00000009930"
##  [6679] "ENSGACG00000012610" "ENSGACG00000009937" "ENSGACG00000030695"
##  [6682] "ENSGACG00000012620" "ENSGACG00000029916" "ENSGACG00000009941"
##  [6685] "ENSGACG00000029070" "ENSGACG00000012648" "ENSGACG00000011782"
##  [6688] "ENSGACG00000009965" "ENSGACG00000027153" "ENSGACG00000032200"
##  [6691] "ENSGACG00000013615" "ENSGACG00000013635" "ENSGACG00000008336"
##  [6694] "ENSGACG00000008350" "ENSGACG00000033168" "ENSGACG00000008367"
##  [6697] "ENSGACG00000009985" "ENSGACG00000013642" "ENSGACG00000013649"
##  [6700] "ENSGACG00000013660" "ENSGACG00000013652" "ENSGACG00000025071"
##  [6703] "ENSGACG00000004478" "ENSGACG00000025079" "ENSGACG00000013661"
##  [6706] "ENSGACG00000028858" "ENSGACG00000013669" "ENSGACG00000013677"
##  [6709] "ENSGACG00000003853" "ENSGACG00000032920" "ENSGACG00000003835"
##  [6712] "ENSGACG00000013690" "ENSGACG00000003826" "ENSGACG00000003804"
##  [6715] "ENSGACG00000034782" "ENSGACG00000031791" "ENSGACG00000003797"
##  [6718] "ENSGACG00000035826" "ENSGACG00000003784" "ENSGACG00000013696"
##  [6721] "ENSGACG00000003759" "ENSGACG00000013699" "ENSGACG00000013704"
##  [6724] "ENSGACG00000003734" "ENSGACG00000003714" "ENSGACG00000029739"
##  [6727] "ENSGACG00000037419" "ENSGACG00000003668" "ENSGACG00000013711"
##  [6730] "ENSGACG00000013715" "ENSGACG00000003653" "ENSGACG00000003644"
##  [6733] "ENSGACG00000032960" "ENSGACG00000003634" "ENSGACG00000010008"
##  [6736] "ENSGACG00000013734" "ENSGACG00000013735" "ENSGACG00000003622"
##  [6739] "ENSGACG00000013747" "ENSGACG00000027636" "ENSGACG00000003618"
##  [6742] "ENSGACG00000013757" "ENSGACG00000025385" "ENSGACG00000013765"
##  [6745] "ENSGACG00000010013" "ENSGACG00000013767" "ENSGACG00000013783"
##  [6748] "ENSGACG00000013789" "ENSGACG00000029713" "ENSGACG00000028615"
##  [6751] "ENSGACG00000013806" "ENSGACG00000003583" "ENSGACG00000013810"
##  [6754] "ENSGACG00000003576" "ENSGACG00000003574" "ENSGACG00000013819"
##  [6757] "ENSGACG00000028772" "ENSGACG00000010020" "ENSGACG00000010031"
##  [6760] "ENSGACG00000010037" "ENSGACG00000035950" "ENSGACG00000013826"
##  [6763] "ENSGACG00000013843" "ENSGACG00000028031" "ENSGACG00000013865"
##  [6766] "ENSGACG00000010070" "ENSGACG00000013872" "ENSGACG00000032363"
##  [6769] "ENSGACG00000027799" "ENSGACG00000033222" "ENSGACG00000013917"
##  [6772] "ENSGACG00000011382" "ENSGACG00000011278" "ENSGACG00000013609"
##  [6775] "ENSGACG00000011796" "ENSGACG00000011806" "ENSGACG00000011817"
##  [6778] "ENSGACG00000011824" "ENSGACG00000011826" "ENSGACG00000026270"
##  [6781] "ENSGACG00000011840" "ENSGACG00000011849" "ENSGACG00000011854"
##  [6784] "ENSGACG00000025152" "ENSGACG00000011404" "ENSGACG00000026286"
##  [6787] "ENSGACG00000010141" "ENSGACG00000010145" "ENSGACG00000010148"
##  [6790] "ENSGACG00000010152" "ENSGACG00000029551" "ENSGACG00000010177"
##  [6793] "ENSGACG00000037350" "ENSGACG00000004268" "ENSGACG00000008816"
##  [6796] "ENSGACG00000024339" "ENSGACG00000010919" "ENSGACG00000031538"
##  [6799] "ENSGACG00000008861" "ENSGACG00000008884" "ENSGACG00000010935"
##  [6802] "ENSGACG00000008917" "ENSGACG00000008931" "ENSGACG00000008939"
##  [6805] "ENSGACG00000008963" "ENSGACG00000008973" "ENSGACG00000008977"
##  [6808] "ENSGACG00000008374" "ENSGACG00000008412" "ENSGACG00000008416"
##  [6811] "ENSGACG00000011494" "ENSGACG00000008426" "ENSGACG00000008429"
##  [6814] "ENSGACG00000011556" "ENSGACG00000011569" "ENSGACG00000007338"
##  [6817] "ENSGACG00000024140" "ENSGACG00000007345" "ENSGACG00000011583"
##  [6820] "ENSGACG00000028749" "ENSGACG00000011758" "ENSGACG00000032857"
##  [6823] "ENSGACG00000011773" "ENSGACG00000010607" "ENSGACG00000010640"
##  [6826] "ENSGACG00000008451" "ENSGACG00000010656" "ENSGACG00000010668"
##  [6829] "ENSGACG00000024957" "ENSGACG00000008464" "ENSGACG00000010674"
##  [6832] "ENSGACG00000010690" "ENSGACG00000010702" "ENSGACG00000037943"
##  [6835] "ENSGACG00000010716" "ENSGACG00000010753" "ENSGACG00000010756"
##  [6838] "ENSGACG00000036334" "ENSGACG00000027048" "ENSGACG00000010796"
##  [6841] "ENSGACG00000010820" "ENSGACG00000010828" "ENSGACG00000010837"
##  [6844] "ENSGACG00000027457" "ENSGACG00000023276" "ENSGACG00000010881"
##  [6847] "ENSGACG00000010886" "ENSGACG00000010897" "ENSGACG00000012153"
##  [6850] "ENSGACG00000012169" "ENSGACG00000013937" "ENSGACG00000030584"
##  [6853] "ENSGACG00000013964" "ENSGACG00000013973" "ENSGACG00000007377"
##  [6856] "ENSGACG00000013983" "ENSGACG00000007382" "ENSGACG00000013987"
##  [6859] "ENSGACG00000007394" "ENSGACG00000007399" "ENSGACG00000007406"
##  [6862] "ENSGACG00000007420" "ENSGACG00000013992" "ENSGACG00000034094"
##  [6865] "ENSGACG00000007495" "ENSGACG00000007510" "ENSGACG00000007528"
##  [6868] "ENSGACG00000007535" "ENSGACG00000034789" "ENSGACG00000007564"
##  [6871] "ENSGACG00000007576" "ENSGACG00000007581" "ENSGACG00000037916"
##  [6874] "ENSGACG00000007625" "ENSGACG00000008499" "ENSGACG00000008502"
##  [6877] "ENSGACG00000008530" "ENSGACG00000032073" "ENSGACG00000009334"
##  [6880] "ENSGACG00000009355" "ENSGACG00000009399" "ENSGACG00000009410"
##  [6883] "ENSGACG00000009462" "ENSGACG00000014003" "ENSGACG00000035976"
##  [6886] "ENSGACG00000009498" "ENSGACG00000014007" "ENSGACG00000009510"
##  [6889] "ENSGACG00000009515" "ENSGACG00000014039" "ENSGACG00000014048"
##  [6892] "ENSGACG00000009529" "ENSGACG00000014056" "ENSGACG00000014058"
##  [6895] "ENSGACG00000014064" "ENSGACG00000014065" "ENSGACG00000009535"
##  [6898] "ENSGACG00000036696" "ENSGACG00000009537" "ENSGACG00000014070"
##  [6901] "ENSGACG00000014080" "ENSGACG00000034484" "ENSGACG00000026617"
##  [6904] "ENSGACG00000014094" "ENSGACG00000036965" "ENSGACG00000009556"
##  [6907] "ENSGACG00000014103" "ENSGACG00000030707" "ENSGACG00000009561"
##  [6910] "ENSGACG00000027284" "ENSGACG00000014106" "ENSGACG00000014111"
##  [6913] "ENSGACG00000014120" "ENSGACG00000014139" "ENSGACG00000009585"
##  [6916] "ENSGACG00000014158" "ENSGACG00000009595" "ENSGACG00000014163"
##  [6919] "ENSGACG00000009599" "ENSGACG00000009614" "ENSGACG00000009632"
##  [6922] "ENSGACG00000009634" "ENSGACG00000009640" "ENSGACG00000014173"
##  [6925] "ENSGACG00000009733" "ENSGACG00000009739" "ENSGACG00000014181"
##  [6928] "ENSGACG00000014205" "ENSGACG00000014209" "ENSGACG00000014215"
##  [6931] "ENSGACG00000014227" "ENSGACG00000014240" "ENSGACG00000010223"
##  [6934] "ENSGACG00000010225" "ENSGACG00000009749" "ENSGACG00000009754"
##  [6937] "ENSGACG00000024024" "ENSGACG00000010245" "ENSGACG00000009777"
##  [6940] "ENSGACG00000010257" "ENSGACG00000010270" "ENSGACG00000010275"
##  [6943] "ENSGACG00000010281" "ENSGACG00000010293" "ENSGACG00000010296"
##  [6946] "ENSGACG00000010311" "ENSGACG00000010315" "ENSGACG00000010369"
##  [6949] "ENSGACG00000034969" "ENSGACG00000010375" "ENSGACG00000009799"
##  [6952] "ENSGACG00000010385" "ENSGACG00000010397" "ENSGACG00000010402"
##  [6955] "ENSGACG00000014408" "ENSGACG00000032035" "ENSGACG00000009848"
##  [6958] "ENSGACG00000009870" "ENSGACG00000009878" "ENSGACG00000014405"
##  [6961] "ENSGACG00000014363" "ENSGACG00000014355" "ENSGACG00000014350"
##  [6964] "ENSGACG00000014334" "ENSGACG00000014341" "ENSGACG00000014333"
##  [6967] "ENSGACG00000009887" "ENSGACG00000024163" "ENSGACG00000034468"
##  [6970] "ENSGACG00000028730" "ENSGACG00000005045" "ENSGACG00000014318"
##  [6973] "ENSGACG00000038098" "ENSGACG00000036583" "ENSGACG00000027487"
##  [6976] "ENSGACG00000005069" "ENSGACG00000029341" "ENSGACG00000030610"
##  [6979] "ENSGACG00000014315" "ENSGACG00000034048" "ENSGACG00000026494"
##  [6982] "ENSGACG00000014309" "ENSGACG00000031219" "ENSGACG00000014286"
##  [6985] "ENSGACG00000034208" "ENSGACG00000005103" "ENSGACG00000005110"
##  [6988] "ENSGACG00000031510" "ENSGACG00000005114" "ENSGACG00000005187"
##  [6991] "ENSGACG00000024395" "ENSGACG00000032144" "ENSGACG00000005204"
##  [6994] "ENSGACG00000023585" "ENSGACG00000033326" "ENSGACG00000014785"
##  [6997] "ENSGACG00000005210" "ENSGACG00000005242" "ENSGACG00000033588"
##  [7000] "ENSGACG00000005251" "ENSGACG00000023330" "ENSGACG00000036823"
##  [7003] "ENSGACG00000014808" "ENSGACG00000014794" "ENSGACG00000005274"
##  [7006] "ENSGACG00000029597" "ENSGACG00000014787" "ENSGACG00000028978"
##  [7009] "ENSGACG00000005295" "ENSGACG00000033538" "ENSGACG00000005345"
##  [7012] "ENSGACG00000005324" "ENSGACG00000033440" "ENSGACG00000014783"
##  [7015] "ENSGACG00000005349" "ENSGACG00000014779" "ENSGACG00000005391"
##  [7018] "ENSGACG00000005395" "ENSGACG00000005404" "ENSGACG00000005409"
##  [7021] "ENSGACG00000014775" "ENSGACG00000005422" "ENSGACG00000014771"
##  [7024] "ENSGACG00000014767" "ENSGACG00000014757" "ENSGACG00000034434"
##  [7027] "ENSGACG00000014744" "ENSGACG00000014721" "ENSGACG00000005508"
##  [7030] "ENSGACG00000005462" "ENSGACG00000005515" "ENSGACG00000030919"
##  [7033] "ENSGACG00000005534" "ENSGACG00000005538" "ENSGACG00000005552"
##  [7036] "ENSGACG00000005569" "ENSGACG00000005574" "ENSGACG00000005625"
##  [7039] "ENSGACG00000005664" "ENSGACG00000034443" "ENSGACG00000036141"
##  [7042] "ENSGACG00000034317" "ENSGACG00000005689" "ENSGACG00000005703"
##  [7045] "ENSGACG00000005726" "ENSGACG00000005837" "ENSGACG00000005772"
##  [7048] "ENSGACG00000005887" "ENSGACG00000036428" "ENSGACG00000005902"
##  [7051] "ENSGACG00000036063" "ENSGACG00000005917" "ENSGACG00000033208"
##  [7054] "ENSGACG00000024455" "ENSGACG00000005927" "ENSGACG00000005945"
##  [7057] "ENSGACG00000005959" "ENSGACG00000005997" "ENSGACG00000027480"
##  [7060] "ENSGACG00000006008" "ENSGACG00000006024" "ENSGACG00000037484"
##  [7063] "ENSGACG00000006094" "ENSGACG00000006107" "ENSGACG00000006116"
##  [7066] "ENSGACG00000006134" "ENSGACG00000006143" "ENSGACG00000006149"
##  [7069] "ENSGACG00000006172" "ENSGACG00000006209" "ENSGACG00000006214"
##  [7072] "ENSGACG00000006220" "ENSGACG00000006242" "ENSGACG00000014719"
##  [7075] "ENSGACG00000014713" "ENSGACG00000014707" "ENSGACG00000023082"
##  [7078] "ENSGACG00000029449" "ENSGACG00000014701" "ENSGACG00000014690"
##  [7081] "ENSGACG00000014691" "ENSGACG00000014682" "ENSGACG00000014676"
##  [7084] "ENSGACG00000014675" "ENSGACG00000014673" "ENSGACG00000014252"
##  [7087] "ENSGACG00000034522" "ENSGACG00000014260" "ENSGACG00000014263"
##  [7090] "ENSGACG00000014266" "ENSGACG00000035522" "ENSGACG00000014599"
##  [7093] "ENSGACG00000014667" "ENSGACG00000014666" "ENSGACG00000014658"
##  [7096] "ENSGACG00000024300" "ENSGACG00000024708" "ENSGACG00000014642"
##  [7099] "ENSGACG00000029655" "ENSGACG00000014618" "ENSGACG00000028172"
##  [7102] "ENSGACG00000026971" "ENSGACG00000014611" "ENSGACG00000014606"
##  [7105] "ENSGACG00000014591" "ENSGACG00000006280" "ENSGACG00000034255"
##  [7108] "ENSGACG00000006305" "ENSGACG00000006295" "ENSGACG00000014571"
##  [7111] "ENSGACG00000006313" "ENSGACG00000037428" "ENSGACG00000006363"
##  [7114] "ENSGACG00000014527" "ENSGACG00000014520" "ENSGACG00000024287"
##  [7117] "ENSGACG00000006389" "ENSGACG00000006405" "ENSGACG00000006524"
##  [7120] "ENSGACG00000014517" "ENSGACG00000014514" "ENSGACG00000036637"
##  [7123] "ENSGACG00000014488" "ENSGACG00000014484" "ENSGACG00000029543"
##  [7126] "ENSGACG00000006513" "ENSGACG00000006542" "ENSGACG00000006548"
##  [7129] "ENSGACG00000023193" "ENSGACG00000024217" "ENSGACG00000006574"
##  [7132] "ENSGACG00000006585" "ENSGACG00000034819" "ENSGACG00000007638"
##  [7135] "ENSGACG00000007653" "ENSGACG00000007679" "ENSGACG00000007686"
##  [7138] "ENSGACG00000007696" "ENSGACG00000007706" "ENSGACG00000007732"
##  [7141] "ENSGACG00000026265" "ENSGACG00000028546" "ENSGACG00000014471"
##  [7144] "ENSGACG00000007745" "ENSGACG00000007757" "ENSGACG00000007765"
##  [7147] "ENSGACG00000007776" "ENSGACG00000007780" "ENSGACG00000032549"
##  [7150] "ENSGACG00000007806" "ENSGACG00000007900" "ENSGACG00000007925"
##  [7153] "ENSGACG00000026210" "ENSGACG00000025576" "ENSGACG00000026672"
##  [7156] "ENSGACG00000008029" "ENSGACG00000008043" "ENSGACG00000023954"
##  [7159] "ENSGACG00000008080" "ENSGACG00000008083" "ENSGACG00000008097"
##  [7162] "ENSGACG00000008111" "ENSGACG00000008122" "ENSGACG00000008128"
##  [7165] "ENSGACG00000008142" "ENSGACG00000008163" "ENSGACG00000008178"
##  [7168] "ENSGACG00000008197" "ENSGACG00000008222" "ENSGACG00000008235"
##  [7171] "ENSGACG00000008243" "ENSGACG00000029173" "ENSGACG00000008255"
##  [7174] "ENSGACG00000008257" "ENSGACG00000014452" "ENSGACG00000014447"
##  [7177] "ENSGACG00000014421" "ENSGACG00000008271" "ENSGACG00000008308"
##  [7180] "ENSGACG00000027797" "ENSGACG00000008274" "ENSGACG00000026205"
##  [7183] "ENSGACG00000033704" "ENSGACG00000034607" "ENSGACG00000026441"
##  [7186] "ENSGACG00000028216" "ENSGACG00000037275" "ENSGACG00000035245"
##  [7189] "ENSGACG00000007973" "ENSGACG00000029394" "ENSGACG00000036807"
##  [7192] "ENSGACG00000037871" "ENSGACG00000032953" "ENSGACG00000023012"
##  [7195] "ENSGACG00000028250" "ENSGACG00000032039" "ENSGACG00000025681"
##  [7198] "ENSGACG00000036855" "ENSGACG00000035626" "ENSGACG00000023048"
##  [7201] "ENSGACG00000036868" "ENSGACG00000033469" "ENSGACG00000025174"
##  [7204] "ENSGACG00000029154" "ENSGACG00000035589" "ENSGACG00000034786"
##  [7207] "ENSGACG00000023225" "ENSGACG00000030979" "ENSGACG00000025929"
##  [7210] "ENSGACG00000035365" "ENSGACG00000004635" "ENSGACG00000026575"
##  [7213] "ENSGACG00000036851" "ENSGACG00000026476" "ENSGACG00000025223"
##  [7216] "ENSGACG00000029123" "ENSGACG00000023691" "ENSGACG00000030020"
##  [7219] "ENSGACG00000026007" "ENSGACG00000032994" "ENSGACG00000033207"
##  [7222] "ENSGACG00000037110" "ENSGACG00000027980" "ENSGACG00000025893"
##  [7225] "ENSGACG00000024741" "ENSGACG00000027581" "ENSGACG00000028678"
##  [7228] "ENSGACG00000033685" "ENSGACG00000036530" "ENSGACG00000024826"
##  [7231] "ENSGACG00000027149" "ENSGACG00000036353" "ENSGACG00000028207"
##  [7234] "ENSGACG00000028817" "ENSGACG00000029097" "ENSGACG00000023395"
##  [7237] "ENSGACG00000032155" "ENSGACG00000026500" "ENSGACG00000031207"
##  [7240] "ENSGACG00000036967" "ENSGACG00000030565" "ENSGACG00000027802"
##  [7243] "ENSGACG00000033215" "ENSGACG00000035019" "ENSGACG00000034301"
##  [7246] "ENSGACG00000030600" "ENSGACG00000038087" "ENSGACG00000037055"
##  [7249] "ENSGACG00000025492" "ENSGACG00000031094" "ENSGACG00000033318"
##  [7252] "ENSGACG00000037079" "ENSGACG00000035542" "ENSGACG00000028495"
##  [7255] "ENSGACG00000034446" "ENSGACG00000032119" "ENSGACG00000029819"
##  [7258] "ENSGACG00000027958" "ENSGACG00000025285" "ENSGACG00000036181"
##  [7261] "ENSGACG00000034949" "ENSGACG00000037920" "ENSGACG00000035979"
##  [7264] "ENSGACG00000010004" "ENSGACG00000026471" "ENSGACG00000012704"
##  [7267] "ENSGACG00000003366" "ENSGACG00000003940" "ENSGACG00000013043"
##  [7270] "ENSGACG00000004289" "ENSGACG00000010419" "ENSGACG00000004500"
##  [7273] "ENSGACG00000033451" "ENSGACG00000028862" "ENSGACG00000028273"
##  [7276] "ENSGACG00000033853" "ENSGACG00000032164" "ENSGACG00000010866"
##  [7279] "ENSGACG00000026619" "ENSGACG00000037525" "ENSGACG00000001385"
##  [7282] "ENSGACG00000029434" "ENSGACG00000006876" "ENSGACG00000007129"
##  [7285] "ENSGACG00000008971" "ENSGACG00000023377" "ENSGACG00000030670"
##  [7288] "ENSGACG00000005630" "ENSGACG00000025379" "ENSGACG00000032127"
##  [7291] "ENSGACG00000006361" "ENSGACG00000023926" "ENSGACG00000009827"
##  [7294] "ENSGACG00000008103" "ENSGACG00000007524" "ENSGACG00000007486"
##  [7297] "ENSGACG00000007475" "ENSGACG00000007396" "ENSGACG00000031374"
##  [7300] "ENSGACG00000007376" "ENSGACG00000007367" "ENSGACG00000007356"
##  [7303] "ENSGACG00000028636" "ENSGACG00000034546" "ENSGACG00000007334"
##  [7306] "ENSGACG00000007325" "ENSGACG00000007313" "ENSGACG00000007303"
##  [7309] "ENSGACG00000007295" "ENSGACG00000007262" "ENSGACG00000030382"
##  [7312] "ENSGACG00000007248" "ENSGACG00000008630" "ENSGACG00000008620"
##  [7315] "ENSGACG00000008614" "ENSGACG00000008590" "ENSGACG00000008575"
##  [7318] "ENSGACG00000008546" "ENSGACG00000003742" "ENSGACG00000008770"
##  [7321] "ENSGACG00000008755" "ENSGACG00000008732" "ENSGACG00000008709"
##  [7324] "ENSGACG00000008681" "ENSGACG00000008662" "ENSGACG00000008645"
##  [7327] "ENSGACG00000007077" "ENSGACG00000007067" "ENSGACG00000007037"
##  [7330] "ENSGACG00000007017" "ENSGACG00000003739" "ENSGACG00000006991"
##  [7333] "ENSGACG00000006975" "ENSGACG00000036504" "ENSGACG00000006968"
##  [7336] "ENSGACG00000032247" "ENSGACG00000006953" "ENSGACG00000035178"
##  [7339] "ENSGACG00000028618" "ENSGACG00000006945" "ENSGACG00000006920"
##  [7342] "ENSGACG00000006919" "ENSGACG00000006914" "ENSGACG00000006841"
##  [7345] "ENSGACG00000008477" "ENSGACG00000008508" "ENSGACG00000008568"
##  [7348] "ENSGACG00000008549" "ENSGACG00000013098" "ENSGACG00000013073"
##  [7351] "ENSGACG00000005862" "ENSGACG00000005848" "ENSGACG00000005838"
##  [7354] "ENSGACG00000007527" "ENSGACG00000005827" "ENSGACG00000005796"
##  [7357] "ENSGACG00000027244" "ENSGACG00000005792" "ENSGACG00000005782"
##  [7360] "ENSGACG00000005775" "ENSGACG00000026344" "ENSGACG00000005768"
##  [7363] "ENSGACG00000005753" "ENSGACG00000005745" "ENSGACG00000029458"
##  [7366] "ENSGACG00000008449" "ENSGACG00000008441" "ENSGACG00000008427"
##  [7369] "ENSGACG00000008417" "ENSGACG00000030224" "ENSGACG00000008381"
##  [7372] "ENSGACG00000008357" "ENSGACG00000008347" "ENSGACG00000008311"
##  [7375] "ENSGACG00000008288" "ENSGACG00000008285" "ENSGACG00000008262"
##  [7378] "ENSGACG00000008208" "ENSGACG00000026299" "ENSGACG00000008244"
##  [7381] "ENSGACG00000008236" "ENSGACG00000035345" "ENSGACG00000008195"
##  [7384] "ENSGACG00000008157" "ENSGACG00000008138" "ENSGACG00000008145"
##  [7387] "ENSGACG00000008082" "ENSGACG00000008079" "ENSGACG00000008072"
##  [7390] "ENSGACG00000008035" "ENSGACG00000007932" "ENSGACG00000008023"
##  [7393] "ENSGACG00000030267" "ENSGACG00000032627" "ENSGACG00000007935"
##  [7396] "ENSGACG00000030098" "ENSGACG00000007913" "ENSGACG00000007907"
##  [7399] "ENSGACG00000032505" "ENSGACG00000007894" "ENSGACG00000007867"
##  [7402] "ENSGACG00000033601" "ENSGACG00000007846" "ENSGACG00000007830"
##  [7405] "ENSGACG00000027179" "ENSGACG00000033225" "ENSGACG00000007810"
##  [7408] "ENSGACG00000007741" "ENSGACG00000007705" "ENSGACG00000007691"
##  [7411] "ENSGACG00000007674" "ENSGACG00000007643" "ENSGACG00000007599"
##  [7414] "ENSGACG00000027018" "ENSGACG00000007575" "ENSGACG00000007572"
##  [7417] "ENSGACG00000007566" "ENSGACG00000007530" "ENSGACG00000007229"
##  [7420] "ENSGACG00000007205" "ENSGACG00000007164" "ENSGACG00000007121"
##  [7423] "ENSGACG00000007124" "ENSGACG00000007115" "ENSGACG00000007105"
##  [7426] "ENSGACG00000007082" "ENSGACG00000013129" "ENSGACG00000003721"
##  [7429] "ENSGACG00000025796" "ENSGACG00000003711" "ENSGACG00000003708"
##  [7432] "ENSGACG00000003706" "ENSGACG00000030148" "ENSGACG00000003663"
##  [7435] "ENSGACG00000003658" "ENSGACG00000003654" "ENSGACG00000034842"
##  [7438] "ENSGACG00000003630" "ENSGACG00000003625" "ENSGACG00000003620"
##  [7441] "ENSGACG00000003616" "ENSGACG00000003613" "ENSGACG00000003602"
##  [7444] "ENSGACG00000031699" "ENSGACG00000003596" "ENSGACG00000003580"
##  [7447] "ENSGACG00000028709" "ENSGACG00000012954" "ENSGACG00000012943"
##  [7450] "ENSGACG00000030355" "ENSGACG00000036450" "ENSGACG00000036164"
##  [7453] "ENSGACG00000012890" "ENSGACG00000012833" "ENSGACG00000033495"
##  [7456] "ENSGACG00000012822" "ENSGACG00000012816" "ENSGACG00000012800"
##  [7459] "ENSGACG00000012784" "ENSGACG00000027702" "ENSGACG00000013035"
##  [7462] "ENSGACG00000013018" "ENSGACG00000012751" "ENSGACG00000033352"
##  [7465] "ENSGACG00000012702" "ENSGACG00000013007" "ENSGACG00000013002"
##  [7468] "ENSGACG00000012997" "ENSGACG00000028318" "ENSGACG00000001234"
##  [7471] "ENSGACG00000001242" "ENSGACG00000030015" "ENSGACG00000032257"
##  [7474] "ENSGACG00000023594" "ENSGACG00000029183" "ENSGACG00000033671"
##  [7477] "ENSGACG00000022991" "ENSGACG00000024746" "ENSGACG00000012700"
##  [7480] "ENSGACG00000012683" "ENSGACG00000012673" "ENSGACG00000012662"
##  [7483] "ENSGACG00000032484" "ENSGACG00000012645" "ENSGACG00000012633"
##  [7486] "ENSGACG00000012574" "ENSGACG00000013354" "ENSGACG00000013344"
##  [7489] "ENSGACG00000027291" "ENSGACG00000027915" "ENSGACG00000013324"
##  [7492] "ENSGACG00000013312" "ENSGACG00000013289" "ENSGACG00000035724"
##  [7495] "ENSGACG00000027557" "ENSGACG00000013277" "ENSGACG00000013243"
##  [7498] "ENSGACG00000030997" "ENSGACG00000013239" "ENSGACG00000013224"
##  [7501] "ENSGACG00000013207" "ENSGACG00000031610" "ENSGACG00000036631"
##  [7504] "ENSGACG00000013176" "ENSGACG00000013173" "ENSGACG00000013165"
##  [7507] "ENSGACG00000013155" "ENSGACG00000013143" "ENSGACG00000035407"
##  [7510] "ENSGACG00000013136" "ENSGACG00000033567" "ENSGACG00000028729"
##  [7513] "ENSGACG00000029773" "ENSGACG00000013117" "ENSGACG00000026486"
##  [7516] "ENSGACG00000033116" "ENSGACG00000013110" "ENSGACG00000006835"
##  [7519] "ENSGACG00000006807" "ENSGACG00000037850" "ENSGACG00000026241"
##  [7522] "ENSGACG00000006798" "ENSGACG00000030597" "ENSGACG00000006774"
##  [7525] "ENSGACG00000030194" "ENSGACG00000006756" "ENSGACG00000035466"
##  [7528] "ENSGACG00000006736" "ENSGACG00000026824" "ENSGACG00000035628"
##  [7531] "ENSGACG00000006646" "ENSGACG00000006609" "ENSGACG00000006600"
##  [7534] "ENSGACG00000006584" "ENSGACG00000006567" "ENSGACG00000006547"
##  [7537] "ENSGACG00000006541" "ENSGACG00000028312" "ENSGACG00000006503"
##  [7540] "ENSGACG00000006473" "ENSGACG00000028476" "ENSGACG00000006464"
##  [7543] "ENSGACG00000006452" "ENSGACG00000006447" "ENSGACG00000026390"
##  [7546] "ENSGACG00000006435" "ENSGACG00000006426" "ENSGACG00000037415"
##  [7549] "ENSGACG00000006365" "ENSGACG00000006411" "ENSGACG00000025699"
##  [7552] "ENSGACG00000032087" "ENSGACG00000006324" "ENSGACG00000006318"
##  [7555] "ENSGACG00000006294" "ENSGACG00000006279" "ENSGACG00000025455"
##  [7558] "ENSGACG00000006271" "ENSGACG00000028390" "ENSGACG00000027616"
##  [7561] "ENSGACG00000006257" "ENSGACG00000006252" "ENSGACG00000006241"
##  [7564] "ENSGACG00000035781" "ENSGACG00000035379" "ENSGACG00000006199"
##  [7567] "ENSGACG00000006159" "ENSGACG00000006122" "ENSGACG00000006117"
##  [7570] "ENSGACG00000006084" "ENSGACG00000006074" "ENSGACG00000006063"
##  [7573] "ENSGACG00000006053" "ENSGACG00000006042" "ENSGACG00000006019"
##  [7576] "ENSGACG00000006005" "ENSGACG00000005995" "ENSGACG00000005980"
##  [7579] "ENSGACG00000005956" "ENSGACG00000026498" "ENSGACG00000034643"
##  [7582] "ENSGACG00000005905" "ENSGACG00000030991" "ENSGACG00000005730"
##  [7585] "ENSGACG00000005727" "ENSGACG00000005719" "ENSGACG00000005716"
##  [7588] "ENSGACG00000005710" "ENSGACG00000005697" "ENSGACG00000027556"
##  [7591] "ENSGACG00000005656" "ENSGACG00000005642" "ENSGACG00000029437"
##  [7594] "ENSGACG00000005619" "ENSGACG00000005604" "ENSGACG00000005593"
##  [7597] "ENSGACG00000005579" "ENSGACG00000005550" "ENSGACG00000005533"
##  [7600] "ENSGACG00000023901" "ENSGACG00000029089" "ENSGACG00000005516"
##  [7603] "ENSGACG00000035358" "ENSGACG00000005498" "ENSGACG00000030799"
##  [7606] "ENSGACG00000005490" "ENSGACG00000005485" "ENSGACG00000005474"
##  [7609] "ENSGACG00000005452" "ENSGACG00000005446" "ENSGACG00000032348"
##  [7612] "ENSGACG00000005412" "ENSGACG00000005356" "ENSGACG00000036875"
##  [7615] "ENSGACG00000032736" "ENSGACG00000005322" "ENSGACG00000027678"
##  [7618] "ENSGACG00000005294" "ENSGACG00000037340" "ENSGACG00000005273"
##  [7621] "ENSGACG00000005260" "ENSGACG00000005254" "ENSGACG00000005229"
##  [7624] "ENSGACG00000005221" "ENSGACG00000036988" "ENSGACG00000034822"
##  [7627] "ENSGACG00000005209" "ENSGACG00000025432" "ENSGACG00000005198"
##  [7630] "ENSGACG00000005177" "ENSGACG00000023221" "ENSGACG00000005166"
##  [7633] "ENSGACG00000005124" "ENSGACG00000005072" "ENSGACG00000005036"
##  [7636] "ENSGACG00000005027" "ENSGACG00000005021" "ENSGACG00000004981"
##  [7639] "ENSGACG00000004973" "ENSGACG00000004944" "ENSGACG00000004938"
##  [7642] "ENSGACG00000004924" "ENSGACG00000004911" "ENSGACG00000004910"
##  [7645] "ENSGACG00000004869" "ENSGACG00000004901" "ENSGACG00000004839"
##  [7648] "ENSGACG00000036651" "ENSGACG00000004807" "ENSGACG00000004780"
##  [7651] "ENSGACG00000031829" "ENSGACG00000036726" "ENSGACG00000004776"
##  [7654] "ENSGACG00000025332" "ENSGACG00000035742" "ENSGACG00000004763"
##  [7657] "ENSGACG00000004753" "ENSGACG00000004740" "ENSGACG00000004734"
##  [7660] "ENSGACG00000004732" "ENSGACG00000004709" "ENSGACG00000024686"
##  [7663] "ENSGACG00000024391" "ENSGACG00000004667" "ENSGACG00000004662"
##  [7666] "ENSGACG00000004655" "ENSGACG00000004646" "ENSGACG00000004620"
##  [7669] "ENSGACG00000004605" "ENSGACG00000004594" "ENSGACG00000004585"
##  [7672] "ENSGACG00000031727" "ENSGACG00000023474" "ENSGACG00000004553"
##  [7675] "ENSGACG00000004544" "ENSGACG00000030704" "ENSGACG00000004467"
##  [7678] "ENSGACG00000035736" "ENSGACG00000010473" "ENSGACG00000036745"
##  [7681] "ENSGACG00000027660" "ENSGACG00000035283" "ENSGACG00000004454"
##  [7684] "ENSGACG00000010460" "ENSGACG00000003024" "ENSGACG00000004451"
##  [7687] "ENSGACG00000003036" "ENSGACG00000004431" "ENSGACG00000028504"
##  [7690] "ENSGACG00000034776" "ENSGACG00000004361" "ENSGACG00000004338"
##  [7693] "ENSGACG00000004309" "ENSGACG00000024286" "ENSGACG00000004286"
##  [7696] "ENSGACG00000004283" "ENSGACG00000004260" "ENSGACG00000003042"
##  [7699] "ENSGACG00000023824" "ENSGACG00000004245" "ENSGACG00000004216"
##  [7702] "ENSGACG00000003056" "ENSGACG00000037416" "ENSGACG00000023720"
##  [7705] "ENSGACG00000004198" "ENSGACG00000004196" "ENSGACG00000004193"
##  [7708] "ENSGACG00000003068" "ENSGACG00000004190" "ENSGACG00000003075"
##  [7711] "ENSGACG00000004167" "ENSGACG00000027323" "ENSGACG00000003096"
##  [7714] "ENSGACG00000015086" "ENSGACG00000015092" "ENSGACG00000030746"
##  [7717] "ENSGACG00000015104" "ENSGACG00000015110" "ENSGACG00000015114"
##  [7720] "ENSGACG00000015119" "ENSGACG00000038015" "ENSGACG00000015123"
##  [7723] "ENSGACG00000015125" "ENSGACG00000015138" "ENSGACG00000015141"
##  [7726] "ENSGACG00000015148" "ENSGACG00000015157" "ENSGACG00000029572"
##  [7729] "ENSGACG00000029780" "ENSGACG00000030054" "ENSGACG00000026779"
##  [7732] "ENSGACG00000015178" "ENSGACG00000025047" "ENSGACG00000015209"
##  [7735] "ENSGACG00000015220" "ENSGACG00000015221" "ENSGACG00000035512"
##  [7738] "ENSGACG00000029464" "ENSGACG00000015226" "ENSGACG00000036953"
##  [7741] "ENSGACG00000015232" "ENSGACG00000015239" "ENSGACG00000015245"
##  [7744] "ENSGACG00000015249" "ENSGACG00000029163" "ENSGACG00000004155"
##  [7747] "ENSGACG00000031967" "ENSGACG00000003425" "ENSGACG00000010405"
##  [7750] "ENSGACG00000010370" "ENSGACG00000010366" "ENSGACG00000010359"
##  [7753] "ENSGACG00000028930" "ENSGACG00000010324" "ENSGACG00000032020"
##  [7756] "ENSGACG00000010303" "ENSGACG00000027592" "ENSGACG00000026189"
##  [7759] "ENSGACG00000010298" "ENSGACG00000026481" "ENSGACG00000004133"
##  [7762] "ENSGACG00000010294" "ENSGACG00000004125" "ENSGACG00000010283"
##  [7765] "ENSGACG00000004127" "ENSGACG00000010273" "ENSGACG00000004113"
##  [7768] "ENSGACG00000004087" "ENSGACG00000036512" "ENSGACG00000004040"
##  [7771] "ENSGACG00000004023" "ENSGACG00000004001" "ENSGACG00000003800"
##  [7774] "ENSGACG00000003787" "ENSGACG00000003777" "ENSGACG00000003767"
##  [7777] "ENSGACG00000010256" "ENSGACG00000003221" "ENSGACG00000003225"
##  [7780] "ENSGACG00000008474" "ENSGACG00000026662" "ENSGACG00000024920"
##  [7783] "ENSGACG00000036986" "ENSGACG00000030541" "ENSGACG00000031599"
##  [7786] "ENSGACG00000029679" "ENSGACG00000030387" "ENSGACG00000034228"
##  [7789] "ENSGACG00000030260" "ENSGACG00000034703" "ENSGACG00000026419"
##  [7792] "ENSGACG00000035163" "ENSGACG00000029762" "ENSGACG00000031422"
##  [7795] "ENSGACG00000025353" "ENSGACG00000032759" "ENSGACG00000033049"
##  [7798] "ENSGACG00000010216" "ENSGACG00000035732" "ENSGACG00000026371"
##  [7801] "ENSGACG00000029608" "ENSGACG00000032496" "ENSGACG00000035572"
##  [7804] "ENSGACG00000029601" "ENSGACG00000030590" "ENSGACG00000023527"
##  [7807] "ENSGACG00000034898" "ENSGACG00000037497" "ENSGACG00000026839"
##  [7810] "ENSGACG00000035428" "ENSGACG00000010196" "ENSGACG00000034397"
##  [7813] "ENSGACG00000035381" "ENSGACG00000035987" "ENSGACG00000029159"
##  [7816] "ENSGACG00000029399" "ENSGACG00000025293" "ENSGACG00000031507"
##  [7819] "ENSGACG00000030375" "ENSGACG00000035651" "ENSGACG00000010181"
##  [7822] "ENSGACG00000029542" "ENSGACG00000029232" "ENSGACG00000025696"
##  [7825] "ENSGACG00000010172" "ENSGACG00000036944" "ENSGACG00000037874"
##  [7828] "ENSGACG00000022900" "ENSGACG00000010164" "ENSGACG00000007468"
##  [7831] "ENSGACG00000031209" "ENSGACG00000031566" "ENSGACG00000005812"
##  [7834] "ENSGACG00000005808" "ENSGACG00000007655" "ENSGACG00000033931"
##  [7837] "ENSGACG00000013198" "ENSGACG00000027107" "ENSGACG00000027579"
##  [7840] "ENSGACG00000006131" "ENSGACG00000005566" "ENSGACG00000005361"
##  [7843] "ENSGACG00000004681" "ENSGACG00000004256" "ENSGACG00000004044"
##  [7846] "ENSGACG00000003794" "ENSGACG00000010158" "ENSGACG00000010153"
##  [7849] "ENSGACG00000010123" "ENSGACG00000010150" "ENSGACG00000010118"
##  [7852] "ENSGACG00000010102" "ENSGACG00000010078" "ENSGACG00000010076"
##  [7855] "ENSGACG00000009964" "ENSGACG00000009961" "ENSGACG00000009953"
##  [7858] "ENSGACG00000009938" "ENSGACG00000009922" "ENSGACG00000009911"
##  [7861] "ENSGACG00000009903" "ENSGACG00000009900" "ENSGACG00000009866"
##  [7864] "ENSGACG00000009844" "ENSGACG00000009843" "ENSGACG00000033761"
##  [7867] "ENSGACG00000035995" "ENSGACG00000009831" "ENSGACG00000034686"
##  [7870] "ENSGACG00000009806" "ENSGACG00000027803" "ENSGACG00000009758"
##  [7873] "ENSGACG00000009752" "ENSGACG00000003245" "ENSGACG00000003249"
##  [7876] "ENSGACG00000009743" "ENSGACG00000009730" "ENSGACG00000027441"
##  [7879] "ENSGACG00000009691" "ENSGACG00000009683" "ENSGACG00000009673"
##  [7882] "ENSGACG00000009663" "ENSGACG00000009660" "ENSGACG00000009654"
##  [7885] "ENSGACG00000009646" "ENSGACG00000034848" "ENSGACG00000003255"
##  [7888] "ENSGACG00000009645" "ENSGACG00000009636" "ENSGACG00000003275"
##  [7891] "ENSGACG00000009612" "ENSGACG00000003278" "ENSGACG00000003293"
##  [7894] "ENSGACG00000003298" "ENSGACG00000003301" "ENSGACG00000028469"
##  [7897] "ENSGACG00000030868" "ENSGACG00000003323" "ENSGACG00000009565"
##  [7900] "ENSGACG00000009463" "ENSGACG00000009569" "ENSGACG00000003339"
##  [7903] "ENSGACG00000029453" "ENSGACG00000029056" "ENSGACG00000003364"
##  [7906] "ENSGACG00000003369" "ENSGACG00000003388" "ENSGACG00000003392"
##  [7909] "ENSGACG00000003402" "ENSGACG00000027368" "ENSGACG00000009456"
##  [7912] "ENSGACG00000009444" "ENSGACG00000003112" "ENSGACG00000025110"
##  [7915] "ENSGACG00000009435" "ENSGACG00000009433" "ENSGACG00000009431"
##  [7918] "ENSGACG00000009430" "ENSGACG00000027840" "ENSGACG00000009416"
##  [7921] "ENSGACG00000024046" "ENSGACG00000009401" "ENSGACG00000009377"
##  [7924] "ENSGACG00000009372" "ENSGACG00000009356" "ENSGACG00000035100"
##  [7927] "ENSGACG00000009347" "ENSGACG00000009344" "ENSGACG00000009332"
##  [7930] "ENSGACG00000009275" "ENSGACG00000009263" "ENSGACG00000009227"
##  [7933] "ENSGACG00000009161" "ENSGACG00000009144" "ENSGACG00000009120"
##  [7936] "ENSGACG00000009119" "ENSGACG00000009116" "ENSGACG00000033647"
##  [7939] "ENSGACG00000009104" "ENSGACG00000009071" "ENSGACG00000029557"
##  [7942] "ENSGACG00000009038" "ENSGACG00000009013" "ENSGACG00000009008"
##  [7945] "ENSGACG00000008996" "ENSGACG00000008991" "ENSGACG00000003431"
##  [7948] "ENSGACG00000003438" "ENSGACG00000003444" "ENSGACG00000003450"
##  [7951] "ENSGACG00000003481" "ENSGACG00000003483" "ENSGACG00000030531"
##  [7954] "ENSGACG00000003505" "ENSGACG00000003535" "ENSGACG00000003565"
##  [7957] "ENSGACG00000012557" "ENSGACG00000012521" "ENSGACG00000003128"
##  [7960] "ENSGACG00000003194" "ENSGACG00000003197" "ENSGACG00000008964"
##  [7963] "ENSGACG00000035162" "ENSGACG00000033066" "ENSGACG00000008944"
##  [7966] "ENSGACG00000008938" "ENSGACG00000008927" "ENSGACG00000008900"
##  [7969] "ENSGACG00000008848" "ENSGACG00000008846" "ENSGACG00000008823"
##  [7972] "ENSGACG00000008789" "ENSGACG00000008976" "ENSGACG00000008972"
##  [7975] "ENSGACG00000008969" "ENSGACG00000034773" "ENSGACG00000000234"
##  [7978] "ENSGACG00000000239" "ENSGACG00000035702" "ENSGACG00000024054"
##  [7981] "ENSGACG00000000262" "ENSGACG00000031509" "ENSGACG00000000267"
##  [7984] "ENSGACG00000000270" "ENSGACG00000000273" "ENSGACG00000000278"
##  [7987] "ENSGACG00000027947" "ENSGACG00000000280" "ENSGACG00000000281"
##  [7990] "ENSGACG00000013407" "ENSGACG00000000286" "ENSGACG00000000288"
##  [7993] "ENSGACG00000000290" "ENSGACG00000029886" "ENSGACG00000000296"
##  [7996] "ENSGACG00000000297" "ENSGACG00000025418" "ENSGACG00000000521"
##  [7999] "ENSGACG00000000518" "ENSGACG00000000515" "ENSGACG00000024476"
##  [8002] "ENSGACG00000000511" "ENSGACG00000000505" "ENSGACG00000000504"
##  [8005] "ENSGACG00000000501" "ENSGACG00000000487" "ENSGACG00000000479"
##  [8008] "ENSGACG00000000466" "ENSGACG00000000454" "ENSGACG00000033691"
##  [8011] "ENSGACG00000000453" "ENSGACG00000000204" "ENSGACG00000000198"
##  [8014] "ENSGACG00000000195" "ENSGACG00000025259" "ENSGACG00000000189"
##  [8017] "ENSGACG00000031226" "ENSGACG00000023161" "ENSGACG00000001596"
##  [8020] "ENSGACG00000035697" "ENSGACG00000001608" "ENSGACG00000001639"
##  [8023] "ENSGACG00000030771" "ENSGACG00000002558" "ENSGACG00000037656"
##  [8026] "ENSGACG00000028904" "ENSGACG00000002569" "ENSGACG00000002576"
##  [8029] "ENSGACG00000002604" "ENSGACG00000002611" "ENSGACG00000002613"
##  [8032] "ENSGACG00000002622" "ENSGACG00000002624" "ENSGACG00000002635"
##  [8035] "ENSGACG00000002639" "ENSGACG00000002650" "ENSGACG00000032561"
##  [8038] "ENSGACG00000036133" "ENSGACG00000002655" "ENSGACG00000002661"
##  [8041] "ENSGACG00000002664" "ENSGACG00000002671" "ENSGACG00000034664"
##  [8044] "ENSGACG00000010929" "ENSGACG00000010925" "ENSGACG00000029694"
##  [8047] "ENSGACG00000010885" "ENSGACG00000010890" "ENSGACG00000031629"
##  [8050] "ENSGACG00000010877" "ENSGACG00000010851" "ENSGACG00000010849"
##  [8053] "ENSGACG00000010842" "ENSGACG00000010838" "ENSGACG00000010810"
##  [8056] "ENSGACG00000010800" "ENSGACG00000010788" "ENSGACG00000010770"
##  [8059] "ENSGACG00000010752" "ENSGACG00000010747" "ENSGACG00000024772"
##  [8062] "ENSGACG00000010722" "ENSGACG00000010714" "ENSGACG00000010703"
##  [8065] "ENSGACG00000010696" "ENSGACG00000010694" "ENSGACG00000037937"
##  [8068] "ENSGACG00000010685" "ENSGACG00000010644" "ENSGACG00000010636"
##  [8071] "ENSGACG00000010564" "ENSGACG00000010554" "ENSGACG00000010525"
##  [8074] "ENSGACG00000031353" "ENSGACG00000010511" "ENSGACG00000010506"
##  [8077] "ENSGACG00000034585" "ENSGACG00000010495" "ENSGACG00000010490"
##  [8080] "ENSGACG00000010484" "ENSGACG00000027912" "ENSGACG00000023884"
##  [8083] "ENSGACG00000002685" "ENSGACG00000002695" "ENSGACG00000002699"
##  [8086] "ENSGACG00000002707" "ENSGACG00000002717" "ENSGACG00000002750"
##  [8089] "ENSGACG00000024052" "ENSGACG00000002753" "ENSGACG00000026325"
##  [8092] "ENSGACG00000002762" "ENSGACG00000002777" "ENSGACG00000023834"
##  [8095] "ENSGACG00000002783" "ENSGACG00000002792" "ENSGACG00000002797"
##  [8098] "ENSGACG00000002806" "ENSGACG00000002815" "ENSGACG00000002844"
##  [8101] "ENSGACG00000002852" "ENSGACG00000002865" "ENSGACG00000002873"
##  [8104] "ENSGACG00000002881" "ENSGACG00000002887" "ENSGACG00000002899"
##  [8107] "ENSGACG00000034360" "ENSGACG00000027164" "ENSGACG00000002907"
##  [8110] "ENSGACG00000002926" "ENSGACG00000002941" "ENSGACG00000035443"
##  [8113] "ENSGACG00000002965" "ENSGACG00000002980" "ENSGACG00000002982"
##  [8116] "ENSGACG00000002985" "ENSGACG00000002990" "ENSGACG00000036717"
##  [8119] "ENSGACG00000032217" "ENSGACG00000024454" "ENSGACG00000000090"
##  [8122] "ENSGACG00000031596" "ENSGACG00000025327" "ENSGACG00000000086"
##  [8125] "ENSGACG00000000084" "ENSGACG00000036980" "ENSGACG00000000082"
##  [8128] "ENSGACG00000024161" "ENSGACG00000000079" "ENSGACG00000000070"
##  [8131] "ENSGACG00000000069" "ENSGACG00000000068" "ENSGACG00000000065"
##  [8134] "ENSGACG00000000064" "ENSGACG00000000063" "ENSGACG00000026645"
##  [8137] "ENSGACG00000000062" "ENSGACG00000000061" "ENSGACG00000000060"
##  [8140] "ENSGACG00000000059" "ENSGACG00000028695" "ENSGACG00000037982"
##  [8143] "ENSGACG00000012425" "ENSGACG00000012448" "ENSGACG00000012391"
##  [8146] "ENSGACG00000034724" "ENSGACG00000025137" "ENSGACG00000027991"
##  [8149] "ENSGACG00000037866" "ENSGACG00000037579" "ENSGACG00000012365"
##  [8152] "ENSGACG00000012362" "ENSGACG00000012359" "ENSGACG00000012353"
##  [8155] "ENSGACG00000012314" "ENSGACG00000012309" "ENSGACG00000031708"
##  [8158] "ENSGACG00000012263" "ENSGACG00000012237" "ENSGACG00000012228"
##  [8161] "ENSGACG00000012214" "ENSGACG00000024276" "ENSGACG00000012202"
##  [8164] "ENSGACG00000012184" "ENSGACG00000032414" "ENSGACG00000012163"
##  [8167] "ENSGACG00000025198" "ENSGACG00000012146" "ENSGACG00000012127"
##  [8170] "ENSGACG00000012123" "ENSGACG00000036213" "ENSGACG00000012097"
##  [8173] "ENSGACG00000012091" "ENSGACG00000012087" "ENSGACG00000012076"
##  [8176] "ENSGACG00000012061" "ENSGACG00000012057" "ENSGACG00000026694"
##  [8179] "ENSGACG00000012054" "ENSGACG00000012046" "ENSGACG00000012022"
##  [8182] "ENSGACG00000029071" "ENSGACG00000025843" "ENSGACG00000012014"
##  [8185] "ENSGACG00000028785" "ENSGACG00000011994" "ENSGACG00000032409"
##  [8188] "ENSGACG00000023554" "ENSGACG00000011977" "ENSGACG00000011967"
##  [8191] "ENSGACG00000011949" "ENSGACG00000027452" "ENSGACG00000011931"
##  [8194] "ENSGACG00000011924" "ENSGACG00000011912" "ENSGACG00000011910"
##  [8197] "ENSGACG00000011886" "ENSGACG00000011868" "ENSGACG00000011834"
##  [8200] "ENSGACG00000011828" "ENSGACG00000024967" "ENSGACG00000035705"
##  [8203] "ENSGACG00000011807" "ENSGACG00000011805" "ENSGACG00000024465"
##  [8206] "ENSGACG00000028900" "ENSGACG00000011799" "ENSGACG00000011783"
##  [8209] "ENSGACG00000011777" "ENSGACG00000037995" "ENSGACG00000011767"
##  [8212] "ENSGACG00000036584" "ENSGACG00000011751" "ENSGACG00000011730"
##  [8215] "ENSGACG00000011728" "ENSGACG00000011691" "ENSGACG00000011678"
##  [8218] "ENSGACG00000011650" "ENSGACG00000011642" "ENSGACG00000011605"
##  [8221] "ENSGACG00000011630" "ENSGACG00000011626" "ENSGACG00000011615"
##  [8224] "ENSGACG00000038070" "ENSGACG00000031738" "ENSGACG00000011519"
##  [8227] "ENSGACG00000011509" "ENSGACG00000026879" "ENSGACG00000011479"
##  [8230] "ENSGACG00000033449" "ENSGACG00000023400" "ENSGACG00000011456"
##  [8233] "ENSGACG00000011442" "ENSGACG00000011410" "ENSGACG00000011406"
##  [8236] "ENSGACG00000011384" "ENSGACG00000011378" "ENSGACG00000023796"
##  [8239] "ENSGACG00000027894" "ENSGACG00000011358" "ENSGACG00000011353"
##  [8242] "ENSGACG00000011349" "ENSGACG00000011338" "ENSGACG00000011309"
##  [8245] "ENSGACG00000011331" "ENSGACG00000027888" "ENSGACG00000011296"
##  [8248] "ENSGACG00000011283" "ENSGACG00000033708" "ENSGACG00000011282"
##  [8251] "ENSGACG00000032803" "ENSGACG00000011260" "ENSGACG00000011252"
##  [8254] "ENSGACG00000011244" "ENSGACG00000011240" "ENSGACG00000011217"
##  [8257] "ENSGACG00000011209" "ENSGACG00000011204" "ENSGACG00000011194"
##  [8260] "ENSGACG00000011193" "ENSGACG00000011187" "ENSGACG00000011186"
##  [8263] "ENSGACG00000011168" "ENSGACG00000011163" "ENSGACG00000011160"
##  [8266] "ENSGACG00000011155" "ENSGACG00000025846" "ENSGACG00000011148"
##  [8269] "ENSGACG00000037977" "ENSGACG00000037030" "ENSGACG00000011135"
##  [8272] "ENSGACG00000036347" "ENSGACG00000011124" "ENSGACG00000011089"
##  [8275] "ENSGACG00000011086" "ENSGACG00000011084" "ENSGACG00000033394"
##  [8278] "ENSGACG00000011061" "ENSGACG00000011047" "ENSGACG00000011044"
##  [8281] "ENSGACG00000011034" "ENSGACG00000011031" "ENSGACG00000011025"
##  [8284] "ENSGACG00000011017" "ENSGACG00000031072" "ENSGACG00000011007"
##  [8287] "ENSGACG00000011000" "ENSGACG00000037436" "ENSGACG00000010990"
##  [8290] "ENSGACG00000010945" "ENSGACG00000030902" "ENSGACG00000025649"
##  [8293] "ENSGACG00000032520" "ENSGACG00000026853" "ENSGACG00000024306"
##  [8296] "ENSGACG00000023103" "ENSGACG00000037073" "ENSGACG00000025745"
##  [8299] "ENSGACG00000027952" "ENSGACG00000025602" "ENSGACG00000030509"
##  [8302] "ENSGACG00000034867" "ENSGACG00000026367" "ENSGACG00000023827"
##  [8305] "ENSGACG00000037386" "ENSGACG00000026743" "ENSGACG00000024524"
##  [8308] "ENSGACG00000025731" "ENSGACG00000027972" "ENSGACG00000025883"
##  [8311] "ENSGACG00000036136" "ENSGACG00000037641" "ENSGACG00000035025"
##  [8314] "ENSGACG00000033370" "ENSGACG00000029319" "ENSGACG00000023143"
##  [8317] "ENSGACG00000023588" "ENSGACG00000034394" "ENSGACG00000030364"
##  [8320] "ENSGACG00000033030" "ENSGACG00000025156" "ENSGACG00000028569"
##  [8323] "ENSGACG00000026829" "ENSGACG00000034291" "ENSGACG00000026102"
##  [8326] "ENSGACG00000037892" "ENSGACG00000023211" "ENSGACG00000028682"
##  [8329] "ENSGACG00000032372" "ENSGACG00000023563" "ENSGACG00000025991"
##  [8332] "ENSGACG00000025315" "ENSGACG00000037389" "ENSGACG00000034442"
##  [8335] "ENSGACG00000027426" "ENSGACG00000026031" "ENSGACG00000036793"
##  [8338] "ENSGACG00000027057" "ENSGACG00000024165" "ENSGACG00000025204"
##  [8341] "ENSGACG00000031990" "ENSGACG00000022817" "ENSGACG00000015129"
##  [8344] "ENSGACG00000032979" "ENSGACG00000012510" "ENSGACG00000001632"
##  [8347] "ENSGACG00000002830" "ENSGACG00000029800" "ENSGACG00000000075"
##  [8350] "ENSGACG00000012439" "ENSGACG00000012157" "ENSGACG00000027307"
##  [8353] "ENSGACG00000032023" "ENSGACG00000024916" "ENSGACG00000011366"
##  [8356] "ENSGACG00000026907" "ENSGACG00000030392" "ENSGACG00000026372"
##  [8359] "ENSGACG00000010807" "ENSGACG00000010798" "ENSGACG00000010775"
##  [8362] "ENSGACG00000035159" "ENSGACG00000010762" "ENSGACG00000010094"
##  [8365] "ENSGACG00000010091" "ENSGACG00000010077" "ENSGACG00000010067"
##  [8368] "ENSGACG00000010054" "ENSGACG00000026136" "ENSGACG00000010032"
##  [8371] "ENSGACG00000010024" "ENSGACG00000010014" "ENSGACG00000027377"
##  [8374] "ENSGACG00000030776" "ENSGACG00000009959" "ENSGACG00000009934"
##  [8377] "ENSGACG00000009927" "ENSGACG00000009918" "ENSGACG00000028781"
##  [8380] "ENSGACG00000010746" "ENSGACG00000031953" "ENSGACG00000009892"
##  [8383] "ENSGACG00000009705" "ENSGACG00000009642" "ENSGACG00000009626"
##  [8386] "ENSGACG00000009605" "ENSGACG00000009572" "ENSGACG00000009562"
##  [8389] "ENSGACG00000034913" "ENSGACG00000026640" "ENSGACG00000009549"
##  [8392] "ENSGACG00000037047" "ENSGACG00000009454" "ENSGACG00000009418"
##  [8395] "ENSGACG00000009388" "ENSGACG00000009378" "ENSGACG00000009906"
##  [8398] "ENSGACG00000036797" "ENSGACG00000035149" "ENSGACG00000010734"
##  [8401] "ENSGACG00000010732" "ENSGACG00000037692" "ENSGACG00000010705"
##  [8404] "ENSGACG00000010689" "ENSGACG00000010682" "ENSGACG00000024038"
##  [8407] "ENSGACG00000027344" "ENSGACG00000036226" "ENSGACG00000010629"
##  [8410] "ENSGACG00000024420" "ENSGACG00000025999" "ENSGACG00000010509"
##  [8413] "ENSGACG00000031118" "ENSGACG00000010496" "ENSGACG00000037794"
##  [8416] "ENSGACG00000010459" "ENSGACG00000010453" "ENSGACG00000010443"
##  [8419] "ENSGACG00000010422" "ENSGACG00000010417" "ENSGACG00000036768"
##  [8422] "ENSGACG00000024763" "ENSGACG00000010239" "ENSGACG00000010230"
##  [8425] "ENSGACG00000010224" "ENSGACG00000010201" "ENSGACG00000035936"
##  [8428] "ENSGACG00000033583" "ENSGACG00000010186" "ENSGACG00000033118"
##  [8431] "ENSGACG00000010168" "ENSGACG00000010163" "ENSGACG00000010114"
##  [8434] "ENSGACG00000009776" "ENSGACG00000009764" "ENSGACG00000009748"
##  [8437] "ENSGACG00000009729" "ENSGACG00000009717" "ENSGACG00000009714"
##  [8440] "ENSGACG00000029833" "ENSGACG00000031057" "ENSGACG00000010827"
##  [8443] "ENSGACG00000010823" "ENSGACG00000004695" "ENSGACG00000004691"
##  [8446] "ENSGACG00000004680" "ENSGACG00000004675" "ENSGACG00000004670"
##  [8449] "ENSGACG00000004613" "ENSGACG00000004596" "ENSGACG00000004578"
##  [8452] "ENSGACG00000004555" "ENSGACG00000004528" "ENSGACG00000027985"
##  [8455] "ENSGACG00000004498" "ENSGACG00000037694" "ENSGACG00000004485"
##  [8458] "ENSGACG00000004439" "ENSGACG00000031579" "ENSGACG00000004409"
##  [8461] "ENSGACG00000037283" "ENSGACG00000004384" "ENSGACG00000004380"
##  [8464] "ENSGACG00000004357" "ENSGACG00000004350" "ENSGACG00000004333"
##  [8467] "ENSGACG00000030410" "ENSGACG00000006564" "ENSGACG00000031862"
##  [8470] "ENSGACG00000024919" "ENSGACG00000036882" "ENSGACG00000006576"
##  [8473] "ENSGACG00000004004" "ENSGACG00000023393" "ENSGACG00000003977"
##  [8476] "ENSGACG00000026455" "ENSGACG00000003947" "ENSGACG00000003928"
##  [8479] "ENSGACG00000003911" "ENSGACG00000003905" "ENSGACG00000003899"
##  [8482] "ENSGACG00000003874" "ENSGACG00000003854" "ENSGACG00000003840"
##  [8485] "ENSGACG00000003832" "ENSGACG00000003821" "ENSGACG00000003792"
##  [8488] "ENSGACG00000003761" "ENSGACG00000028249" "ENSGACG00000003757"
##  [8491] "ENSGACG00000026437" "ENSGACG00000003748" "ENSGACG00000003720"
##  [8494] "ENSGACG00000003703" "ENSGACG00000003688" "ENSGACG00000003661"
##  [8497] "ENSGACG00000003650" "ENSGACG00000003639" "ENSGACG00000003627"
##  [8500] "ENSGACG00000003609" "ENSGACG00000003597" "ENSGACG00000003589"
##  [8503] "ENSGACG00000037859" "ENSGACG00000032822" "ENSGACG00000034863"
##  [8506] "ENSGACG00000003554" "ENSGACG00000003546" "ENSGACG00000031874"
##  [8509] "ENSGACG00000003509" "ENSGACG00000036900" "ENSGACG00000003491"
##  [8512] "ENSGACG00000003487" "ENSGACG00000036787" "ENSGACG00000003482"
##  [8515] "ENSGACG00000003466" "ENSGACG00000037487" "ENSGACG00000003460"
##  [8518] "ENSGACG00000031919" "ENSGACG00000010936" "ENSGACG00000028648"
##  [8521] "ENSGACG00000010891" "ENSGACG00000010898" "ENSGACG00000016444"
##  [8524] "ENSGACG00000010863" "ENSGACG00000006624" "ENSGACG00000009885"
##  [8527] "ENSGACG00000026058" "ENSGACG00000009867" "ENSGACG00000009849"
##  [8530] "ENSGACG00000009803" "ENSGACG00000009794" "ENSGACG00000009783"
##  [8533] "ENSGACG00000027505" "ENSGACG00000004276" "ENSGACG00000004250"
##  [8536] "ENSGACG00000031664" "ENSGACG00000004247" "ENSGACG00000004229"
##  [8539] "ENSGACG00000004215" "ENSGACG00000023292" "ENSGACG00000004145"
##  [8542] "ENSGACG00000025598" "ENSGACG00000004129" "ENSGACG00000004122"
##  [8545] "ENSGACG00000004112" "ENSGACG00000004103" "ENSGACG00000004098"
##  [8548] "ENSGACG00000004088" "ENSGACG00000036279" "ENSGACG00000004063"
##  [8551] "ENSGACG00000004050" "ENSGACG00000004028" "ENSGACG00000004020"
##  [8554] "ENSGACG00000004325" "ENSGACG00000011437" "ENSGACG00000011381"
##  [8557] "ENSGACG00000011397" "ENSGACG00000011389" "ENSGACG00000011388"
##  [8560] "ENSGACG00000026456" "ENSGACG00000011372" "ENSGACG00000025631"
##  [8563] "ENSGACG00000011348" "ENSGACG00000011344" "ENSGACG00000011340"
##  [8566] "ENSGACG00000011311" "ENSGACG00000011270" "ENSGACG00000011262"
##  [8569] "ENSGACG00000011253" "ENSGACG00000023576" "ENSGACG00000033428"
##  [8572] "ENSGACG00000035166" "ENSGACG00000011188" "ENSGACG00000011179"
##  [8575] "ENSGACG00000011177" "ENSGACG00000010944" "ENSGACG00000036078"
##  [8578] "ENSGACG00000011141" "ENSGACG00000032900" "ENSGACG00000011130"
##  [8581] "ENSGACG00000011125" "ENSGACG00000011104" "ENSGACG00000030062"
##  [8584] "ENSGACG00000011093" "ENSGACG00000037906" "ENSGACG00000011062"
##  [8587] "ENSGACG00000031332" "ENSGACG00000034390" "ENSGACG00000028922"
##  [8590] "ENSGACG00000025215" "ENSGACG00000011004" "ENSGACG00000010988"
##  [8593] "ENSGACG00000010978" "ENSGACG00000010954" "ENSGACG00000010963"
##  [8596] "ENSGACG00000032004" "ENSGACG00000007525" "ENSGACG00000007499"
##  [8599] "ENSGACG00000007480" "ENSGACG00000024512" "ENSGACG00000007450"
##  [8602] "ENSGACG00000007434" "ENSGACG00000007425" "ENSGACG00000007393"
##  [8605] "ENSGACG00000007380" "ENSGACG00000007371" "ENSGACG00000007341"
##  [8608] "ENSGACG00000007291" "ENSGACG00000007281" "ENSGACG00000007256"
##  [8611] "ENSGACG00000030493" "ENSGACG00000007242" "ENSGACG00000007238"
##  [8614] "ENSGACG00000031750" "ENSGACG00000007210" "ENSGACG00000007186"
##  [8617] "ENSGACG00000025485" "ENSGACG00000027232" "ENSGACG00000007153"
##  [8620] "ENSGACG00000037806" "ENSGACG00000007114" "ENSGACG00000007109"
##  [8623] "ENSGACG00000007102" "ENSGACG00000007091" "ENSGACG00000007084"
##  [8626] "ENSGACG00000007054" "ENSGACG00000007047" "ENSGACG00000007033"
##  [8629] "ENSGACG00000006990" "ENSGACG00000006985" "ENSGACG00000029865"
##  [8632] "ENSGACG00000006950" "ENSGACG00000022952" "ENSGACG00000024574"
##  [8635] "ENSGACG00000006822" "ENSGACG00000024665" "ENSGACG00000034543"
##  [8638] "ENSGACG00000006810" "ENSGACG00000035754" "ENSGACG00000006806"
##  [8641] "ENSGACG00000006759" "ENSGACG00000006738" "ENSGACG00000006703"
##  [8644] "ENSGACG00000006701" "ENSGACG00000003348" "ENSGACG00000006687"
##  [8647] "ENSGACG00000030803" "ENSGACG00000003368" "ENSGACG00000035733"
##  [8650] "ENSGACG00000006659" "ENSGACG00000006649" "ENSGACG00000037799"
##  [8653] "ENSGACG00000035110" "ENSGACG00000007601" "ENSGACG00000027046"
##  [8656] "ENSGACG00000024851" "ENSGACG00000012191" "ENSGACG00000032626"
##  [8659] "ENSGACG00000004142" "ENSGACG00000031584" "ENSGACG00000035563"
##  [8662] "ENSGACG00000007588" "ENSGACG00000037545" "ENSGACG00000032166"
##  [8665] "ENSGACG00000012113" "ENSGACG00000032616" "ENSGACG00000012079"
##  [8668] "ENSGACG00000007560" "ENSGACG00000007545" "ENSGACG00000035223"
##  [8671] "ENSGACG00000012071" "ENSGACG00000037964" "ENSGACG00000006521"
##  [8674] "ENSGACG00000006393" "ENSGACG00000037401" "ENSGACG00000012053"
##  [8677] "ENSGACG00000012040" "ENSGACG00000012034" "ENSGACG00000012021"
##  [8680] "ENSGACG00000011996" "ENSGACG00000038027" "ENSGACG00000011975"
##  [8683] "ENSGACG00000011958" "ENSGACG00000031465" "ENSGACG00000034798"
##  [8686] "ENSGACG00000029988" "ENSGACG00000011906" "ENSGACG00000029326"
##  [8689] "ENSGACG00000026121" "ENSGACG00000031948" "ENSGACG00000011879"
##  [8692] "ENSGACG00000006502" "ENSGACG00000023667" "ENSGACG00000033719"
##  [8695] "ENSGACG00000028203" "ENSGACG00000011833" "ENSGACG00000011830"
##  [8698] "ENSGACG00000028968" "ENSGACG00000036224" "ENSGACG00000030155"
##  [8701] "ENSGACG00000006370" "ENSGACG00000027605" "ENSGACG00000035762"
##  [8704] "ENSGACG00000006315" "ENSGACG00000006281" "ENSGACG00000006260"
##  [8707] "ENSGACG00000006232" "ENSGACG00000006224" "ENSGACG00000011784"
##  [8710] "ENSGACG00000006219" "ENSGACG00000011771" "ENSGACG00000006202"
##  [8713] "ENSGACG00000036208" "ENSGACG00000013544" "ENSGACG00000013538"
##  [8716] "ENSGACG00000037564" "ENSGACG00000024236" "ENSGACG00000013499"
##  [8719] "ENSGACG00000026852" "ENSGACG00000025677" "ENSGACG00000023223"
##  [8722] "ENSGACG00000013487" "ENSGACG00000027290" "ENSGACG00000013483"
##  [8725] "ENSGACG00000013480" "ENSGACG00000013473" "ENSGACG00000013465"
##  [8728] "ENSGACG00000029939" "ENSGACG00000036325" "ENSGACG00000013433"
##  [8731] "ENSGACG00000036950" "ENSGACG00000037024" "ENSGACG00000013379"
##  [8734] "ENSGACG00000029654" "ENSGACG00000035808" "ENSGACG00000013365"
##  [8737] "ENSGACG00000013358" "ENSGACG00000034856" "ENSGACG00000025711"
##  [8740] "ENSGACG00000013333" "ENSGACG00000006163" "ENSGACG00000006158"
##  [8743] "ENSGACG00000006154" "ENSGACG00000006141" "ENSGACG00000006135"
##  [8746] "ENSGACG00000006120" "ENSGACG00000006110" "ENSGACG00000006101"
##  [8749] "ENSGACG00000032705" "ENSGACG00000006072" "ENSGACG00000029050"
##  [8752] "ENSGACG00000035074" "ENSGACG00000006025" "ENSGACG00000006001"
##  [8755] "ENSGACG00000013331" "ENSGACG00000013321" "ENSGACG00000013260"
##  [8758] "ENSGACG00000013258" "ENSGACG00000013246" "ENSGACG00000005996"
##  [8761] "ENSGACG00000005988" "ENSGACG00000005982" "ENSGACG00000024473"
##  [8764] "ENSGACG00000035889" "ENSGACG00000024410" "ENSGACG00000005957"
##  [8767] "ENSGACG00000032455" "ENSGACG00000005940" "ENSGACG00000005942"
##  [8770] "ENSGACG00000005915" "ENSGACG00000025627" "ENSGACG00000013238"
##  [8773] "ENSGACG00000014846" "ENSGACG00000005909" "ENSGACG00000027622"
##  [8776] "ENSGACG00000030921" "ENSGACG00000031362" "ENSGACG00000005899"
##  [8779] "ENSGACG00000011725" "ENSGACG00000025446" "ENSGACG00000025844"
##  [8782] "ENSGACG00000005889" "ENSGACG00000011708" "ENSGACG00000030063"
##  [8785] "ENSGACG00000005868" "ENSGACG00000028997" "ENSGACG00000011684"
##  [8788] "ENSGACG00000005853" "ENSGACG00000027403" "ENSGACG00000005842"
##  [8791] "ENSGACG00000011661" "ENSGACG00000031448" "ENSGACG00000030046"
##  [8794] "ENSGACG00000011647" "ENSGACG00000005809" "ENSGACG00000005806"
##  [8797] "ENSGACG00000011633" "ENSGACG00000005794" "ENSGACG00000035188"
##  [8800] "ENSGACG00000005752" "ENSGACG00000011591" "ENSGACG00000005737"
##  [8803] "ENSGACG00000011573" "ENSGACG00000011564" "ENSGACG00000011548"
##  [8806] "ENSGACG00000038031" "ENSGACG00000005733" "ENSGACG00000011538"
##  [8809] "ENSGACG00000011535" "ENSGACG00000011505" "ENSGACG00000005659"
##  [8812] "ENSGACG00000011484" "ENSGACG00000005632" "ENSGACG00000005618"
##  [8815] "ENSGACG00000005613" "ENSGACG00000005607" "ENSGACG00000005590"
##  [8818] "ENSGACG00000005561" "ENSGACG00000011478" "ENSGACG00000037061"
##  [8821] "ENSGACG00000005541" "ENSGACG00000031202" "ENSGACG00000023141"
##  [8824] "ENSGACG00000005521" "ENSGACG00000027908" "ENSGACG00000005514"
##  [8827] "ENSGACG00000005489" "ENSGACG00000028196" "ENSGACG00000033244"
##  [8830] "ENSGACG00000028634" "ENSGACG00000005468" "ENSGACG00000035022"
##  [8833] "ENSGACG00000029612" "ENSGACG00000005442" "ENSGACG00000005416"
##  [8836] "ENSGACG00000023064" "ENSGACG00000033371" "ENSGACG00000005365"
##  [8839] "ENSGACG00000005355" "ENSGACG00000005331" "ENSGACG00000005293"
##  [8842] "ENSGACG00000005255" "ENSGACG00000005252" "ENSGACG00000005226"
##  [8845] "ENSGACG00000005213" "ENSGACG00000013167" "ENSGACG00000013149"
##  [8848] "ENSGACG00000013135" "ENSGACG00000005181" "ENSGACG00000005176"
##  [8851] "ENSGACG00000005171" "ENSGACG00000005167" "ENSGACG00000005158"
##  [8854] "ENSGACG00000005119" "ENSGACG00000005092" "ENSGACG00000005085"
##  [8857] "ENSGACG00000005078" "ENSGACG00000005043" "ENSGACG00000033301"
##  [8860] "ENSGACG00000004903" "ENSGACG00000013122" "ENSGACG00000013120"
##  [8863] "ENSGACG00000013113" "ENSGACG00000022956" "ENSGACG00000013089"
##  [8866] "ENSGACG00000004885" "ENSGACG00000013060" "ENSGACG00000026596"
##  [8869] "ENSGACG00000004867" "ENSGACG00000013030" "ENSGACG00000012979"
##  [8872] "ENSGACG00000004859" "ENSGACG00000004827" "ENSGACG00000012971"
##  [8875] "ENSGACG00000004806" "ENSGACG00000023508" "ENSGACG00000004795"
##  [8878] "ENSGACG00000027122" "ENSGACG00000012946" "ENSGACG00000004744"
##  [8881] "ENSGACG00000012928" "ENSGACG00000038058" "ENSGACG00000035940"
##  [8884] "ENSGACG00000027648" "ENSGACG00000034311" "ENSGACG00000033742"
##  [8887] "ENSGACG00000033238" "ENSGACG00000031723" "ENSGACG00000012796"
##  [8890] "ENSGACG00000004772" "ENSGACG00000012787" "ENSGACG00000012770"
##  [8893] "ENSGACG00000023943" "ENSGACG00000004739" "ENSGACG00000004713"
##  [8896] "ENSGACG00000012746" "ENSGACG00000009373" "ENSGACG00000026593"
##  [8899] "ENSGACG00000009345" "ENSGACG00000024884" "ENSGACG00000009342"
##  [8902] "ENSGACG00000009335" "ENSGACG00000012711" "ENSGACG00000009309"
##  [8905] "ENSGACG00000029839" "ENSGACG00000009210" "ENSGACG00000009202"
##  [8908] "ENSGACG00000035859" "ENSGACG00000009190" "ENSGACG00000009107"
##  [8911] "ENSGACG00000031733" "ENSGACG00000029025" "ENSGACG00000034594"
##  [8914] "ENSGACG00000009061" "ENSGACG00000012647" "ENSGACG00000009039"
##  [8917] "ENSGACG00000033967" "ENSGACG00000029950" "ENSGACG00000012612"
##  [8920] "ENSGACG00000012608" "ENSGACG00000027587" "ENSGACG00000012580"
##  [8923] "ENSGACG00000012555" "ENSGACG00000012541" "ENSGACG00000034462"
##  [8926] "ENSGACG00000032343" "ENSGACG00000029352" "ENSGACG00000032515"
##  [8929] "ENSGACG00000012474" "ENSGACG00000030184" "ENSGACG00000032732"
##  [8932] "ENSGACG00000012412" "ENSGACG00000025679" "ENSGACG00000012381"
##  [8935] "ENSGACG00000012349" "ENSGACG00000012306" "ENSGACG00000009015"
##  [8938] "ENSGACG00000038011" "ENSGACG00000008981" "ENSGACG00000008957"
##  [8941] "ENSGACG00000012282" "ENSGACG00000012275" "ENSGACG00000012232"
##  [8944] "ENSGACG00000013611" "ENSGACG00000003385" "ENSGACG00000034328"
##  [8947] "ENSGACG00000003448" "ENSGACG00000002749" "ENSGACG00000014140"
##  [8950] "ENSGACG00000031963" "ENSGACG00000014112" "ENSGACG00000024874"
##  [8953] "ENSGACG00000014093" "ENSGACG00000014090" "ENSGACG00000028362"
##  [8956] "ENSGACG00000014081" "ENSGACG00000029908" "ENSGACG00000023731"
##  [8959] "ENSGACG00000023436" "ENSGACG00000032305" "ENSGACG00000014025"
##  [8962] "ENSGACG00000013996" "ENSGACG00000015873" "ENSGACG00000035205"
##  [8965] "ENSGACG00000037304" "ENSGACG00000013963" "ENSGACG00000013931"
##  [8968] "ENSGACG00000025680" "ENSGACG00000027282" "ENSGACG00000013899"
##  [8971] "ENSGACG00000013896" "ENSGACG00000030503" "ENSGACG00000032539"
##  [8974] "ENSGACG00000013883" "ENSGACG00000028386" "ENSGACG00000013858"
##  [8977] "ENSGACG00000037922" "ENSGACG00000013856" "ENSGACG00000029893"
##  [8980] "ENSGACG00000013845" "ENSGACG00000024552" "ENSGACG00000022920"
##  [8983] "ENSGACG00000013820" "ENSGACG00000013816" "ENSGACG00000013812"
##  [8986] "ENSGACG00000013805" "ENSGACG00000036502" "ENSGACG00000029247"
##  [8989] "ENSGACG00000038078" "ENSGACG00000013788" "ENSGACG00000027443"
##  [8992] "ENSGACG00000013779" "ENSGACG00000028769" "ENSGACG00000037938"
##  [8995] "ENSGACG00000029162" "ENSGACG00000008942" "ENSGACG00000013768"
##  [8998] "ENSGACG00000013763" "ENSGACG00000008935" "ENSGACG00000008928"
##  [9001] "ENSGACG00000008914" "ENSGACG00000013731" "ENSGACG00000008898"
##  [9004] "ENSGACG00000013724" "ENSGACG00000008891" "ENSGACG00000008880"
##  [9007] "ENSGACG00000013720" "ENSGACG00000013714" "ENSGACG00000033247"
##  [9010] "ENSGACG00000025916" "ENSGACG00000008843" "ENSGACG00000013705"
##  [9013] "ENSGACG00000013695" "ENSGACG00000008837" "ENSGACG00000026161"
##  [9016] "ENSGACG00000008779" "ENSGACG00000008783" "ENSGACG00000013687"
##  [9019] "ENSGACG00000023806" "ENSGACG00000008757" "ENSGACG00000027764"
##  [9022] "ENSGACG00000013670" "ENSGACG00000013655" "ENSGACG00000008743"
##  [9025] "ENSGACG00000023925" "ENSGACG00000028666" "ENSGACG00000008727"
##  [9028] "ENSGACG00000027449" "ENSGACG00000008678" "ENSGACG00000036917"
##  [9031] "ENSGACG00000008655" "ENSGACG00000013643" "ENSGACG00000024928"
##  [9034] "ENSGACG00000030904" "ENSGACG00000013631" "ENSGACG00000008638"
##  [9037] "ENSGACG00000013623" "ENSGACG00000013600" "ENSGACG00000008617"
##  [9040] "ENSGACG00000025855" "ENSGACG00000013564" "ENSGACG00000013552"
##  [9043] "ENSGACG00000013547" "ENSGACG00000002019" "ENSGACG00000000625"
##  [9046] "ENSGACG00000037823" "ENSGACG00000032018" "ENSGACG00000002020"
##  [9049] "ENSGACG00000002032" "ENSGACG00000031607" "ENSGACG00000008577"
##  [9052] "ENSGACG00000002033" "ENSGACG00000008550" "ENSGACG00000024625"
##  [9055] "ENSGACG00000028357" "ENSGACG00000008536" "ENSGACG00000008531"
##  [9058] "ENSGACG00000024330" "ENSGACG00000029300" "ENSGACG00000037985"
##  [9061] "ENSGACG00000008494" "ENSGACG00000002100" "ENSGACG00000002102"
##  [9064] "ENSGACG00000008453" "ENSGACG00000022531" "ENSGACG00000034219"
##  [9067] "ENSGACG00000000483" "ENSGACG00000008489" "ENSGACG00000026932"
##  [9070] "ENSGACG00000008384" "ENSGACG00000008376" "ENSGACG00000000482"
##  [9073] "ENSGACG00000031782" "ENSGACG00000036280" "ENSGACG00000000473"
##  [9076] "ENSGACG00000033114" "ENSGACG00000030010" "ENSGACG00000000459"
##  [9079] "ENSGACG00000030775" "ENSGACG00000023275" "ENSGACG00000008302"
##  [9082] "ENSGACG00000008240" "ENSGACG00000000452" "ENSGACG00000037750"
##  [9085] "ENSGACG00000008186" "ENSGACG00000002104" "ENSGACG00000008159"
##  [9088] "ENSGACG00000028792" "ENSGACG00000008153" "ENSGACG00000024157"
##  [9091] "ENSGACG00000008150" "ENSGACG00000029002" "ENSGACG00000008130"
##  [9094] "ENSGACG00000002114" "ENSGACG00000008086" "ENSGACG00000002120"
##  [9097] "ENSGACG00000008057" "ENSGACG00000028838" "ENSGACG00000008044"
##  [9100] "ENSGACG00000008030" "ENSGACG00000002134" "ENSGACG00000034258"
##  [9103] "ENSGACG00000002139" "ENSGACG00000025279" "ENSGACG00000002151"
##  [9106] "ENSGACG00000002154" "ENSGACG00000025666" "ENSGACG00000028277"
##  [9109] "ENSGACG00000024434" "ENSGACG00000008022" "ENSGACG00000008016"
##  [9112] "ENSGACG00000002184" "ENSGACG00000008007" "ENSGACG00000034495"
##  [9115] "ENSGACG00000007990" "ENSGACG00000023636" "ENSGACG00000007976"
##  [9118] "ENSGACG00000007967" "ENSGACG00000007956" "ENSGACG00000007952"
##  [9121] "ENSGACG00000007919" "ENSGACG00000035011" "ENSGACG00000007902"
##  [9124] "ENSGACG00000002194" "ENSGACG00000002219" "ENSGACG00000002221"
##  [9127] "ENSGACG00000002229" "ENSGACG00000002250" "ENSGACG00000035459"
##  [9130] "ENSGACG00000002265" "ENSGACG00000030952" "ENSGACG00000034527"
##  [9133] "ENSGACG00000007872" "ENSGACG00000024112" "ENSGACG00000035871"
##  [9136] "ENSGACG00000024689" "ENSGACG00000007833" "ENSGACG00000025566"
##  [9139] "ENSGACG00000007829" "ENSGACG00000007787" "ENSGACG00000029841"
##  [9142] "ENSGACG00000027191" "ENSGACG00000007744" "ENSGACG00000007738"
##  [9145] "ENSGACG00000007729" "ENSGACG00000007651" "ENSGACG00000007717"
##  [9148] "ENSGACG00000002280" "ENSGACG00000002282" "ENSGACG00000002294"
##  [9151] "ENSGACG00000007682" "ENSGACG00000028668" "ENSGACG00000024834"
##  [9154] "ENSGACG00000024554" "ENSGACG00000026786" "ENSGACG00000031907"
##  [9157] "ENSGACG00000027651" "ENSGACG00000030929" "ENSGACG00000030280"
##  [9160] "ENSGACG00000032075" "ENSGACG00000029113" "ENSGACG00000024635"
##  [9163] "ENSGACG00000033385" "ENSGACG00000036366" "ENSGACG00000034402"
##  [9166] "ENSGACG00000002305" "ENSGACG00000033280" "ENSGACG00000037267"
##  [9169] "ENSGACG00000034639" "ENSGACG00000002356" "ENSGACG00000024258"
##  [9172] "ENSGACG00000002360" "ENSGACG00000028429" "ENSGACG00000002363"
##  [9175] "ENSGACG00000025395" "ENSGACG00000031409" "ENSGACG00000027104"
##  [9178] "ENSGACG00000033071" "ENSGACG00000029594" "ENSGACG00000023644"
##  [9181] "ENSGACG00000035495" "ENSGACG00000030842" "ENSGACG00000002368"
##  [9184] "ENSGACG00000037789" "ENSGACG00000024866" "ENSGACG00000025873"
##  [9187] "ENSGACG00000027442" "ENSGACG00000034398" "ENSGACG00000002381"
##  [9190] "ENSGACG00000029933" "ENSGACG00000035320" "ENSGACG00000025869"
##  [9193] "ENSGACG00000038111" "ENSGACG00000023469" "ENSGACG00000028268"
##  [9196] "ENSGACG00000030680" "ENSGACG00000030429" "ENSGACG00000022681"
##  [9199] "ENSGACG00000022583" "ENSGACG00000029127" "ENSGACG00000010060"
##  [9202] "ENSGACG00000002398" "ENSGACG00000010749" "ENSGACG00000009450"
##  [9205] "ENSGACG00000004563" "ENSGACG00000003573" "ENSGACG00000030196"
##  [9208] "ENSGACG00000032399" "ENSGACG00000004724" "ENSGACG00000008589"
##  [9211] "ENSGACG00000007858" "ENSGACG00000027197" "ENSGACG00000002430"
##  [9214] "ENSGACG00000034707" "ENSGACG00000002434" "ENSGACG00000002437"
##  [9217] "ENSGACG00000002445" "ENSGACG00000002452" "ENSGACG00000033751"
##  [9220] "ENSGACG00000002456" "ENSGACG00000002457" "ENSGACG00000002476"
##  [9223] "ENSGACG00000034942" "ENSGACG00000002490" "ENSGACG00000030774"
##  [9226] "ENSGACG00000002497" "ENSGACG00000002504" "ENSGACG00000002509"
##  [9229] "ENSGACG00000002515" "ENSGACG00000002520" "ENSGACG00000002527"
##  [9232] "ENSGACG00000002529" "ENSGACG00000002537" "ENSGACG00000002538"
##  [9235] "ENSGACG00000037210" "ENSGACG00000002560" "ENSGACG00000002601"
##  [9238] "ENSGACG00000002606" "ENSGACG00000002623" "ENSGACG00000002626"
##  [9241] "ENSGACG00000037352" "ENSGACG00000002633" "ENSGACG00000002638"
##  [9244] "ENSGACG00000002644" "ENSGACG00000028058" "ENSGACG00000002646"
##  [9247] "ENSGACG00000002651" "ENSGACG00000002667" "ENSGACG00000002690"
##  [9250] "ENSGACG00000001261" "ENSGACG00000002867" "ENSGACG00000002874"
##  [9253] "ENSGACG00000002886" "ENSGACG00000029181" "ENSGACG00000035037"
##  [9256] "ENSGACG00000003013" "ENSGACG00000003069" "ENSGACG00000003089"
##  [9259] "ENSGACG00000003097" "ENSGACG00000003117" "ENSGACG00000025933"
##  [9262] "ENSGACG00000026905" "ENSGACG00000003130" "ENSGACG00000038038"
##  [9265] "ENSGACG00000029001" "ENSGACG00000037074" "ENSGACG00000031474"
##  [9268] "ENSGACG00000037753" "ENSGACG00000003198" "ENSGACG00000036938"
##  [9271] "ENSGACG00000003213" "ENSGACG00000036025" "ENSGACG00000003242"
##  [9274] "ENSGACG00000028733" "ENSGACG00000003257" "ENSGACG00000035886"
##  [9277] "ENSGACG00000003261" "ENSGACG00000003267" "ENSGACG00000003273"
##  [9280] "ENSGACG00000026111" "ENSGACG00000003286" "ENSGACG00000003312"
##  [9283] "ENSGACG00000003328" "ENSGACG00000003338" "ENSGACG00000036283"
##  [9286] "ENSGACG00000031008" "ENSGACG00000025730" "ENSGACG00000034732"
##  [9289] "ENSGACG00000032334" "ENSGACG00000033221" "ENSGACG00000034955"
##  [9292] "ENSGACG00000037522" "ENSGACG00000027814" "ENSGACG00000027000"
##  [9295] "ENSGACG00000024652" "ENSGACG00000026436" "ENSGACG00000037763"
##  [9298] "ENSGACG00000022929" "ENSGACG00000036531" "ENSGACG00000025496"
##  [9301] "ENSGACG00000023947" "ENSGACG00000037295" "ENSGACG00000023557"
##  [9304] "ENSGACG00000025770" "ENSGACG00000037902" "ENSGACG00000026751"
##  [9307] "ENSGACG00000026768" "ENSGACG00000032393" "ENSGACG00000037895"
##  [9310] "ENSGACG00000033629" "ENSGACG00000023045" "ENSGACG00000031036"
##  [9313] "ENSGACG00000036476" "ENSGACG00000029423" "ENSGACG00000030705"
##  [9316] "ENSGACG00000028206" "ENSGACG00000025102" "ENSGACG00000030519"
##  [9319] "ENSGACG00000036148" "ENSGACG00000034068" "ENSGACG00000028076"
##  [9322] "ENSGACG00000035302" "ENSGACG00000031945" "ENSGACG00000013517"
##  [9325] "ENSGACG00000026192" "ENSGACG00000028372" "ENSGACG00000013939"
##  [9328] "ENSGACG00000034073" "ENSGACG00000000464" "ENSGACG00000002541"
##  [9331] "ENSGACG00000002859" "ENSGACG00000032827" "ENSGACG00000003152"
##  [9334] "ENSGACG00000024596" "ENSGACG00000023633" "ENSGACG00000003852"
##  [9337] "ENSGACG00000003859" "ENSGACG00000004353" "ENSGACG00000004354"
##  [9340] "ENSGACG00000004360" "ENSGACG00000004383" "ENSGACG00000004397"
##  [9343] "ENSGACG00000038065" "ENSGACG00000004440" "ENSGACG00000004463"
##  [9346] "ENSGACG00000004497" "ENSGACG00000004502" "ENSGACG00000031373"
##  [9349] "ENSGACG00000004519" "ENSGACG00000004588" "ENSGACG00000004623"
##  [9352] "ENSGACG00000004648" "ENSGACG00000004688" "ENSGACG00000004712"
##  [9355] "ENSGACG00000033090" "ENSGACG00000028645" "ENSGACG00000004794"
##  [9358] "ENSGACG00000004812" "ENSGACG00000006527" "ENSGACG00000006258"
##  [9361] "ENSGACG00000006933" "ENSGACG00000033529" "ENSGACG00000025922"
##  [9364] "ENSGACG00000005791" "ENSGACG00000006100" "ENSGACG00000006519"
##  [9367] "ENSGACG00000004842" "ENSGACG00000004848" "ENSGACG00000004858"
##  [9370] "ENSGACG00000006104" "ENSGACG00000006125" "ENSGACG00000006138"
##  [9373] "ENSGACG00000006156" "ENSGACG00000031849" "ENSGACG00000006189"
##  [9376] "ENSGACG00000034268" "ENSGACG00000013592" "ENSGACG00000013599"
##  [9379] "ENSGACG00000025575" "ENSGACG00000029728" "ENSGACG00000013618"
##  [9382] "ENSGACG00000007278" "ENSGACG00000010240" "ENSGACG00000010250"
##  [9385] "ENSGACG00000036257" "ENSGACG00000010265" "ENSGACG00000007288"
##  [9388] "ENSGACG00000007309" "ENSGACG00000010289" "ENSGACG00000011869"
##  [9391] "ENSGACG00000011891" "ENSGACG00000011897" "ENSGACG00000011930"
##  [9394] "ENSGACG00000030304" "ENSGACG00000007373" "ENSGACG00000011943"
##  [9397] "ENSGACG00000029527" "ENSGACG00000010143" "ENSGACG00000011963"
##  [9400] "ENSGACG00000013629" "ENSGACG00000007384" "ENSGACG00000013636"
##  [9403] "ENSGACG00000013645" "ENSGACG00000013658" "ENSGACG00000025749"
##  [9406] "ENSGACG00000013647" "ENSGACG00000007456" "ENSGACG00000013681"
##  [9409] "ENSGACG00000013697" "ENSGACG00000013702" "ENSGACG00000013729"
##  [9412] "ENSGACG00000013744" "ENSGACG00000013748" "ENSGACG00000013753"
##  [9415] "ENSGACG00000013764" "ENSGACG00000013771" "ENSGACG00000034754"
##  [9418] "ENSGACG00000025697" "ENSGACG00000007505" "ENSGACG00000026283"
##  [9421] "ENSGACG00000007515" "ENSGACG00000007541" "ENSGACG00000007559"
##  [9424] "ENSGACG00000024325" "ENSGACG00000007614" "ENSGACG00000007641"
##  [9427] "ENSGACG00000031572" "ENSGACG00000013790" "ENSGACG00000013804"
##  [9430] "ENSGACG00000032183" "ENSGACG00000013824" "ENSGACG00000013833"
##  [9433] "ENSGACG00000013834" "ENSGACG00000007650" "ENSGACG00000013836"
##  [9436] "ENSGACG00000013838" "ENSGACG00000013844" "ENSGACG00000013847"
##  [9439] "ENSGACG00000013857" "ENSGACG00000007669" "ENSGACG00000007678"
##  [9442] "ENSGACG00000013868" "ENSGACG00000007681" "ENSGACG00000013892"
##  [9445] "ENSGACG00000007701" "ENSGACG00000007726" "ENSGACG00000007733"
##  [9448] "ENSGACG00000013911" "ENSGACG00000010528" "ENSGACG00000010560"
##  [9451] "ENSGACG00000007760" "ENSGACG00000007766" "ENSGACG00000008773"
##  [9454] "ENSGACG00000007771" "ENSGACG00000007777" "ENSGACG00000007785"
##  [9457] "ENSGACG00000007790" "ENSGACG00000007802" "ENSGACG00000006222"
##  [9460] "ENSGACG00000033374" "ENSGACG00000006238" "ENSGACG00000004877"
##  [9463] "ENSGACG00000004884" "ENSGACG00000004923" "ENSGACG00000004947"
##  [9466] "ENSGACG00000023415" "ENSGACG00000004957" "ENSGACG00000004966"
##  [9469] "ENSGACG00000004977" "ENSGACG00000005009" "ENSGACG00000005011"
##  [9472] "ENSGACG00000005016" "ENSGACG00000005020" "ENSGACG00000023547"
##  [9475] "ENSGACG00000005039" "ENSGACG00000005050" "ENSGACG00000033147"
##  [9478] "ENSGACG00000005076" "ENSGACG00000005088" "ENSGACG00000005100"
##  [9481] "ENSGACG00000025958" "ENSGACG00000029164" "ENSGACG00000037645"
##  [9484] "ENSGACG00000005129" "ENSGACG00000005151" "ENSGACG00000005180"
##  [9487] "ENSGACG00000005215" "ENSGACG00000005195" "ENSGACG00000032524"
##  [9490] "ENSGACG00000005246" "ENSGACG00000005282" "ENSGACG00000005320"
##  [9493] "ENSGACG00000005332" "ENSGACG00000005347" "ENSGACG00000027691"
##  [9496] "ENSGACG00000005357" "ENSGACG00000005364" "ENSGACG00000005402"
##  [9499] "ENSGACG00000028084" "ENSGACG00000005423" "ENSGACG00000005437"
##  [9502] "ENSGACG00000025705" "ENSGACG00000005567" "ENSGACG00000005636"
##  [9505] "ENSGACG00000033810" "ENSGACG00000005657" "ENSGACG00000005640"
##  [9508] "ENSGACG00000005672" "ENSGACG00000005701" "ENSGACG00000005708"
##  [9511] "ENSGACG00000032966" "ENSGACG00000005773" "ENSGACG00000006285"
##  [9514] "ENSGACG00000029788" "ENSGACG00000006320" "ENSGACG00000025632"
##  [9517] "ENSGACG00000006388" "ENSGACG00000006422" "ENSGACG00000006440"
##  [9520] "ENSGACG00000037437" "ENSGACG00000006461" "ENSGACG00000006451"
##  [9523] "ENSGACG00000006490" "ENSGACG00000006499" "ENSGACG00000006515"
##  [9526] "ENSGACG00000006934" "ENSGACG00000006949" "ENSGACG00000006964"
##  [9529] "ENSGACG00000007064" "ENSGACG00000023762" "ENSGACG00000003289"
##  [9532] "ENSGACG00000038073" "ENSGACG00000003316" "ENSGACG00000003317"
##  [9535] "ENSGACG00000003320" "ENSGACG00000003329" "ENSGACG00000035787"
##  [9538] "ENSGACG00000003347" "ENSGACG00000003362" "ENSGACG00000036464"
##  [9541] "ENSGACG00000003374" "ENSGACG00000031104" "ENSGACG00000003386"
##  [9544] "ENSGACG00000003529" "ENSGACG00000003537" "ENSGACG00000034574"
##  [9547] "ENSGACG00000003556" "ENSGACG00000003577" "ENSGACG00000033870"
##  [9550] "ENSGACG00000003623" "ENSGACG00000003637" "ENSGACG00000003659"
##  [9553] "ENSGACG00000003665" "ENSGACG00000003698" "ENSGACG00000003709"
##  [9556] "ENSGACG00000003718" "ENSGACG00000003726" "ENSGACG00000003746"
##  [9559] "ENSGACG00000003749" "ENSGACG00000003755" "ENSGACG00000003760"
##  [9562] "ENSGACG00000003775" "ENSGACG00000003029" "ENSGACG00000008804"
##  [9565] "ENSGACG00000008808" "ENSGACG00000008821" "ENSGACG00000008850"
##  [9568] "ENSGACG00000003046" "ENSGACG00000003052" "ENSGACG00000008858"
##  [9571] "ENSGACG00000003082" "ENSGACG00000003093" "ENSGACG00000003111"
##  [9574] "ENSGACG00000008863" "ENSGACG00000008878" "ENSGACG00000029571"
##  [9577] "ENSGACG00000003125" "ENSGACG00000003813" "ENSGACG00000003138"
##  [9580] "ENSGACG00000008887" "ENSGACG00000008896" "ENSGACG00000036418"
##  [9583] "ENSGACG00000008915" "ENSGACG00000003827" "ENSGACG00000003839"
##  [9586] "ENSGACG00000031119" "ENSGACG00000003843" "ENSGACG00000008966"
##  [9589] "ENSGACG00000008970" "ENSGACG00000003153" "ENSGACG00000023117"
##  [9592] "ENSGACG00000009002" "ENSGACG00000003158" "ENSGACG00000030465"
##  [9595] "ENSGACG00000009019" "ENSGACG00000024440" "ENSGACG00000009042"
##  [9598] "ENSGACG00000003193" "ENSGACG00000009051" "ENSGACG00000003196"
##  [9601] "ENSGACG00000011969" "ENSGACG00000011985" "ENSGACG00000022955"
##  [9604] "ENSGACG00000005845" "ENSGACG00000005854" "ENSGACG00000005873"
##  [9607] "ENSGACG00000036645" "ENSGACG00000005883" "ENSGACG00000024707"
##  [9610] "ENSGACG00000005908" "ENSGACG00000001733" "ENSGACG00000005920"
##  [9613] "ENSGACG00000005930" "ENSGACG00000012028" "ENSGACG00000007816"
##  [9616] "ENSGACG00000036330" "ENSGACG00000007843" "ENSGACG00000005939"
##  [9619] "ENSGACG00000005948" "ENSGACG00000007863" "ENSGACG00000007885"
##  [9622] "ENSGACG00000028008" "ENSGACG00000007908" "ENSGACG00000005999"
##  [9625] "ENSGACG00000032789" "ENSGACG00000006016" "ENSGACG00000007918"
##  [9628] "ENSGACG00000006030" "ENSGACG00000007929" "ENSGACG00000037607"
##  [9631] "ENSGACG00000007937" "ENSGACG00000006052" "ENSGACG00000007940"
##  [9634] "ENSGACG00000007958" "ENSGACG00000007974" "ENSGACG00000007983"
##  [9637] "ENSGACG00000022942" "ENSGACG00000008041" "ENSGACG00000036342"
##  [9640] "ENSGACG00000008047" "ENSGACG00000003263" "ENSGACG00000008078"
##  [9643] "ENSGACG00000003277" "ENSGACG00000029347" "ENSGACG00000003882"
##  [9646] "ENSGACG00000003886" "ENSGACG00000003908" "ENSGACG00000008140"
##  [9649] "ENSGACG00000003948" "ENSGACG00000003964" "ENSGACG00000036561"
##  [9652] "ENSGACG00000035480" "ENSGACG00000025127" "ENSGACG00000010107"
##  [9655] "ENSGACG00000010119" "ENSGACG00000003973" "ENSGACG00000032895"
##  [9658] "ENSGACG00000027350" "ENSGACG00000010129" "ENSGACG00000013339"
##  [9661] "ENSGACG00000013348" "ENSGACG00000004068" "ENSGACG00000013349"
##  [9664] "ENSGACG00000028865" "ENSGACG00000004090" "ENSGACG00000013373"
##  [9667] "ENSGACG00000013380" "ENSGACG00000025267" "ENSGACG00000004123"
##  [9670] "ENSGACG00000004130" "ENSGACG00000013385" "ENSGACG00000035032"
##  [9673] "ENSGACG00000037803" "ENSGACG00000030125" "ENSGACG00000035144"
##  [9676] "ENSGACG00000013428" "ENSGACG00000013444" "ENSGACG00000013497"
##  [9679] "ENSGACG00000013457" "ENSGACG00000013505" "ENSGACG00000013513"
##  [9682] "ENSGACG00000004224" "ENSGACG00000013532" "ENSGACG00000004230"
##  [9685] "ENSGACG00000037955" "ENSGACG00000033288" "ENSGACG00000033982"
##  [9688] "ENSGACG00000004241" "ENSGACG00000013542" "ENSGACG00000013582"
##  [9691] "ENSGACG00000013587" "ENSGACG00000023995" "ENSGACG00000036885"
##  [9694] "ENSGACG00000035546" "ENSGACG00000027502" "ENSGACG00000010040"
##  [9697] "ENSGACG00000010050" "ENSGACG00000010066" "ENSGACG00000004257"
##  [9700] "ENSGACG00000007086" "ENSGACG00000010075" "ENSGACG00000010082"
##  [9703] "ENSGACG00000031650" "ENSGACG00000031123" "ENSGACG00000010211"
##  [9706] "ENSGACG00000009062" "ENSGACG00000009069" "ENSGACG00000023861"
##  [9709] "ENSGACG00000007122" "ENSGACG00000007145" "ENSGACG00000024663"
##  [9712] "ENSGACG00000009086" "ENSGACG00000009134" "ENSGACG00000009139"
##  [9715] "ENSGACG00000009180" "ENSGACG00000009187" "ENSGACG00000009192"
##  [9718] "ENSGACG00000009198" "ENSGACG00000007168" "ENSGACG00000007183"
##  [9721] "ENSGACG00000028446" "ENSGACG00000007200" "ENSGACG00000009208"
##  [9724] "ENSGACG00000032747" "ENSGACG00000007207" "ENSGACG00000007240"
##  [9727] "ENSGACG00000035557" "ENSGACG00000007249" "ENSGACG00000007263"
##  [9730] "ENSGACG00000014346" "ENSGACG00000004264" "ENSGACG00000032870"
##  [9733] "ENSGACG00000028353" "ENSGACG00000024827" "ENSGACG00000004290"
##  [9736] "ENSGACG00000014359" "ENSGACG00000014370" "ENSGACG00000004301"
##  [9739] "ENSGACG00000014705" "ENSGACG00000004328" "ENSGACG00000014686"
##  [9742] "ENSGACG00000031181" "ENSGACG00000035810" "ENSGACG00000033795"
##  [9745] "ENSGACG00000004346" "ENSGACG00000014653" "ENSGACG00000036640"
##  [9748] "ENSGACG00000002004" "ENSGACG00000014646" "ENSGACG00000002006"
##  [9751] "ENSGACG00000014644" "ENSGACG00000014613" "ENSGACG00000014604"
##  [9754] "ENSGACG00000002016" "ENSGACG00000014587" "ENSGACG00000002021"
##  [9757] "ENSGACG00000002023" "ENSGACG00000014590" "ENSGACG00000030118"
##  [9760] "ENSGACG00000014574" "ENSGACG00000027649" "ENSGACG00000002028"
##  [9763] "ENSGACG00000014569" "ENSGACG00000002039" "ENSGACG00000033314"
##  [9766] "ENSGACG00000027758" "ENSGACG00000035179" "ENSGACG00000014565"
##  [9769] "ENSGACG00000036538" "ENSGACG00000014562" "ENSGACG00000002053"
##  [9772] "ENSGACG00000014545" "ENSGACG00000026365" "ENSGACG00000032322"
##  [9775] "ENSGACG00000014535" "ENSGACG00000002058" "ENSGACG00000014531"
##  [9778] "ENSGACG00000033882" "ENSGACG00000014528" "ENSGACG00000014467"
##  [9781] "ENSGACG00000014457" "ENSGACG00000002063" "ENSGACG00000009251"
##  [9784] "ENSGACG00000036391" "ENSGACG00000014445" "ENSGACG00000038103"
##  [9787] "ENSGACG00000023897" "ENSGACG00000002072" "ENSGACG00000009278"
##  [9790] "ENSGACG00000014431" "ENSGACG00000014428" "ENSGACG00000014412"
##  [9793] "ENSGACG00000028716" "ENSGACG00000002080" "ENSGACG00000014404"
##  [9796] "ENSGACG00000002084" "ENSGACG00000014380" "ENSGACG00000029705"
##  [9799] "ENSGACG00000014374" "ENSGACG00000025035" "ENSGACG00000002099"
##  [9802] "ENSGACG00000031628" "ENSGACG00000032618" "ENSGACG00000036701"
##  [9805] "ENSGACG00000029702" "ENSGACG00000031537" "ENSGACG00000024035"
##  [9808] "ENSGACG00000002117" "ENSGACG00000002121" "ENSGACG00000002126"
##  [9811] "ENSGACG00000002132" "ENSGACG00000002136" "ENSGACG00000002137"
##  [9814] "ENSGACG00000002150" "ENSGACG00000026320" "ENSGACG00000002928"
##  [9817] "ENSGACG00000009295" "ENSGACG00000026916" "ENSGACG00000009323"
##  [9820] "ENSGACG00000009337" "ENSGACG00000009346" "ENSGACG00000009375"
##  [9823] "ENSGACG00000009382" "ENSGACG00000036879" "ENSGACG00000009453"
##  [9826] "ENSGACG00000009457" "ENSGACG00000009483" "ENSGACG00000009490"
##  [9829] "ENSGACG00000035674" "ENSGACG00000002940" "ENSGACG00000002948"
##  [9832] "ENSGACG00000002952" "ENSGACG00000002976" "ENSGACG00000002994"
##  [9835] "ENSGACG00000009517" "ENSGACG00000009545" "ENSGACG00000003008"
##  [9838] "ENSGACG00000009588" "ENSGACG00000003021" "ENSGACG00000009607"
##  [9841] "ENSGACG00000006551" "ENSGACG00000023413" "ENSGACG00000006604"
##  [9844] "ENSGACG00000033726" "ENSGACG00000006617" "ENSGACG00000009671"
##  [9847] "ENSGACG00000025836" "ENSGACG00000009676" "ENSGACG00000009686"
##  [9850] "ENSGACG00000006695" "ENSGACG00000006704" "ENSGACG00000006699"
##  [9853] "ENSGACG00000006723" "ENSGACG00000009692" "ENSGACG00000009697"
##  [9856] "ENSGACG00000006730" "ENSGACG00000006754" "ENSGACG00000031816"
##  [9859] "ENSGACG00000006802" "ENSGACG00000029081" "ENSGACG00000006827"
##  [9862] "ENSGACG00000035313" "ENSGACG00000006853" "ENSGACG00000006859"
##  [9865] "ENSGACG00000009769" "ENSGACG00000006902" "ENSGACG00000006923"
##  [9868] "ENSGACG00000024911" "ENSGACG00000025613" "ENSGACG00000034483"
##  [9871] "ENSGACG00000037200" "ENSGACG00000037120" "ENSGACG00000027967"
##  [9874] "ENSGACG00000034164" "ENSGACG00000037773" "ENSGACG00000027114"
##  [9877] "ENSGACG00000027393" "ENSGACG00000024820" "ENSGACG00000032943"
##  [9880] "ENSGACG00000037445" "ENSGACG00000024099" "ENSGACG00000027688"
##  [9883] "ENSGACG00000033548" "ENSGACG00000031891" "ENSGACG00000033586"
##  [9886] "ENSGACG00000022974" "ENSGACG00000033367" "ENSGACG00000034064"
##  [9889] "ENSGACG00000035171" "ENSGACG00000033321" "ENSGACG00000023099"
##  [9892] "ENSGACG00000035767" "ENSGACG00000030188" "ENSGACG00000028239"
##  [9895] "ENSGACG00000029216" "ENSGACG00000030723" "ENSGACG00000031777"
##  [9898] "ENSGACG00000026687" "ENSGACG00000031196" "ENSGACG00000027907"
##  [9901] "ENSGACG00000031305" "ENSGACG00000036877" "ENSGACG00000037292"
##  [9904] "ENSGACG00000027087" "ENSGACG00000028292" "ENSGACG00000027709"
##  [9907] "ENSGACG00000032074" "ENSGACG00000026345" "ENSGACG00000030578"
##  [9910] "ENSGACG00000025593" "ENSGACG00000032925" "ENSGACG00000029452"
##  [9913] "ENSGACG00000032047" "ENSGACG00000028414" "ENSGACG00000022997"
##  [9916] "ENSGACG00000005463" "ENSGACG00000026049" "ENSGACG00000030787"
##  [9919] "ENSGACG00000007214" "ENSGACG00000026981" "ENSGACG00000006637"
##  [9922] "ENSGACG00000011570" "ENSGACG00000011585" "ENSGACG00000011588"
##  [9925] "ENSGACG00000035630" "ENSGACG00000011603" "ENSGACG00000011604"
##  [9928] "ENSGACG00000011611" "ENSGACG00000011634" "ENSGACG00000024406"
##  [9931] "ENSGACG00000011663" "ENSGACG00000011682" "ENSGACG00000011685"
##  [9934] "ENSGACG00000011702" "ENSGACG00000011711" "ENSGACG00000032495"
##  [9937] "ENSGACG00000023208" "ENSGACG00000011738" "ENSGACG00000011746"
##  [9940] "ENSGACG00000011760" "ENSGACG00000032310" "ENSGACG00000036731"
##  [9943] "ENSGACG00000011857" "ENSGACG00000011864" "ENSGACG00000011545"
##  [9946] "ENSGACG00000010568" "ENSGACG00000029806" "ENSGACG00000035294"
##  [9949] "ENSGACG00000037861" "ENSGACG00000010621" "ENSGACG00000010652"
##  [9952] "ENSGACG00000010658" "ENSGACG00000010666" "ENSGACG00000034346"
##  [9955] "ENSGACG00000037134" "ENSGACG00000010712" "ENSGACG00000010766"
##  [9958] "ENSGACG00000010782" "ENSGACG00000010822" "ENSGACG00000010846"
##  [9961] "ENSGACG00000010853" "ENSGACG00000025325" "ENSGACG00000010867"
##  [9964] "ENSGACG00000010884" "ENSGACG00000010924" "ENSGACG00000010969"
##  [9967] "ENSGACG00000010999" "ENSGACG00000011032" "ENSGACG00000011040"
##  [9970] "ENSGACG00000011065" "ENSGACG00000011083" "ENSGACG00000011114"
##  [9973] "ENSGACG00000011122" "ENSGACG00000011131" "ENSGACG00000011145"
##  [9976] "ENSGACG00000009802" "ENSGACG00000009808" "ENSGACG00000011152"
##  [9979] "ENSGACG00000009820" "ENSGACG00000009823" "ENSGACG00000009829"
##  [9982] "ENSGACG00000009841" "ENSGACG00000009857" "ENSGACG00000011171"
##  [9985] "ENSGACG00000009890" "ENSGACG00000011164" "ENSGACG00000011219"
##  [9988] "ENSGACG00000026550" "ENSGACG00000024224" "ENSGACG00000011182"
##  [9991] "ENSGACG00000030891" "ENSGACG00000011233" "ENSGACG00000009940"
##  [9994] "ENSGACG00000026753" "ENSGACG00000011239" "ENSGACG00000009931"
##  [9997] "ENSGACG00000029345" "ENSGACG00000009992" "ENSGACG00000027056"
## [10000] "ENSGACG00000009997" "ENSGACG00000010015" "ENSGACG00000026821"
## [10003] "ENSGACG00000011294" "ENSGACG00000011305" "ENSGACG00000010027"
## [10006] "ENSGACG00000011336" "ENSGACG00000028070" "ENSGACG00000011354"
## [10009] "ENSGACG00000011385" "ENSGACG00000029567" "ENSGACG00000033333"
## [10012] "ENSGACG00000023483" "ENSGACG00000011403" "ENSGACG00000011407"
## [10015] "ENSGACG00000011421" "ENSGACG00000011444" "ENSGACG00000028232"
## [10018] "ENSGACG00000011470" "ENSGACG00000011473" "ENSGACG00000034711"
## [10021] "ENSGACG00000011508" "ENSGACG00000011516" "ENSGACG00000027976"
## [10024] "ENSGACG00000024842" "ENSGACG00000011536" "ENSGACG00000011551"
## [10027] "ENSGACG00000028186" "ENSGACG00000029554" "ENSGACG00000023980"
## [10030] "ENSGACG00000010321" "ENSGACG00000010353" "ENSGACG00000025947"
## [10033] "ENSGACG00000036738" "ENSGACG00000010394" "ENSGACG00000023163"
## [10036] "ENSGACG00000010398" "ENSGACG00000010425" "ENSGACG00000010468"
## [10039] "ENSGACG00000010475" "ENSGACG00000037618" "ENSGACG00000010494"
## [10042] "ENSGACG00000010526" "ENSGACG00000036413" "ENSGACG00000033865"
## [10045] "ENSGACG00000008179" "ENSGACG00000008183" "ENSGACG00000008194"
## [10048] "ENSGACG00000008201" "ENSGACG00000008209" "ENSGACG00000008217"
## [10051] "ENSGACG00000008230" "ENSGACG00000008306" "ENSGACG00000008341"
## [10054] "ENSGACG00000008366" "ENSGACG00000008372" "ENSGACG00000008378"
## [10057] "ENSGACG00000008385" "ENSGACG00000008403" "ENSGACG00000008437"
## [10060] "ENSGACG00000026259" "ENSGACG00000008445" "ENSGACG00000030133"
## [10063] "ENSGACG00000008475" "ENSGACG00000034196" "ENSGACG00000008482"
## [10066] "ENSGACG00000008558" "ENSGACG00000008574" "ENSGACG00000008588"
## [10069] "ENSGACG00000035334" "ENSGACG00000008622" "ENSGACG00000030654"
## [10072] "ENSGACG00000012036" "ENSGACG00000036030" "ENSGACG00000012055"
## [10075] "ENSGACG00000012093" "ENSGACG00000012104" "ENSGACG00000027527"
## [10078] "ENSGACG00000012189" "ENSGACG00000012201" "ENSGACG00000012212"
## [10081] "ENSGACG00000012224" "ENSGACG00000028659" "ENSGACG00000008636"
## [10084] "ENSGACG00000012280" "ENSGACG00000008643" "ENSGACG00000008647"
## [10087] "ENSGACG00000012286" "ENSGACG00000012294" "ENSGACG00000012323"
## [10090] "ENSGACG00000012326" "ENSGACG00000012330" "ENSGACG00000012364"
## [10093] "ENSGACG00000037846" "ENSGACG00000012396" "ENSGACG00000012402"
## [10096] "ENSGACG00000012421" "ENSGACG00000012442" "ENSGACG00000008677"
## [10099] "ENSGACG00000008694" "ENSGACG00000008720" "ENSGACG00000008741"
## [10102] "ENSGACG00000008745" "ENSGACG00000008762" "ENSGACG00000012465"
## [10105] "ENSGACG00000012489" "ENSGACG00000012492" "ENSGACG00000012496"
## [10108] "ENSGACG00000012501" "ENSGACG00000012561" "ENSGACG00000012588"
## [10111] "ENSGACG00000012629" "ENSGACG00000012643" "ENSGACG00000038052"
## [10114] "ENSGACG00000012652" "ENSGACG00000037328" "ENSGACG00000012658"
## [10117] "ENSGACG00000012671" "ENSGACG00000012681" "ENSGACG00000012686"
## [10120] "ENSGACG00000032029" "ENSGACG00000012737" "ENSGACG00000031687"
## [10123] "ENSGACG00000012755" "ENSGACG00000012760" "ENSGACG00000035829"
## [10126] "ENSGACG00000012785" "ENSGACG00000012793" "ENSGACG00000012870"
## [10129] "ENSGACG00000012876" "ENSGACG00000035708" "ENSGACG00000012884"
## [10132] "ENSGACG00000025899" "ENSGACG00000012935" "ENSGACG00000012951"
## [10135] "ENSGACG00000029512" "ENSGACG00000012957" "ENSGACG00000012964"
## [10138] "ENSGACG00000012970" "ENSGACG00000012987" "ENSGACG00000012995"
## [10141] "ENSGACG00000013005" "ENSGACG00000013027" "ENSGACG00000013029"
## [10144] "ENSGACG00000013031" "ENSGACG00000032879" "ENSGACG00000013036"
## [10147] "ENSGACG00000013039" "ENSGACG00000013045" "ENSGACG00000013058"
## [10150] "ENSGACG00000013066" "ENSGACG00000013071" "ENSGACG00000013094"
## [10153] "ENSGACG00000013096" "ENSGACG00000013097" "ENSGACG00000013104"
## [10156] "ENSGACG00000013111" "ENSGACG00000013123" "ENSGACG00000013146"
## [10159] "ENSGACG00000013151" "ENSGACG00000013190" "ENSGACG00000013208"
## [10162] "ENSGACG00000013232" "ENSGACG00000013272" "ENSGACG00000023501"
## [10165] "ENSGACG00000013247" "ENSGACG00000032936" "ENSGACG00000033697"
## [10168] "ENSGACG00000013288" "ENSGACG00000013297" "ENSGACG00000013320"
## [10171] "ENSGACG00000013329" "ENSGACG00000032474" "ENSGACG00000024480"
## [10174] "ENSGACG00000013966" "ENSGACG00000034151" "ENSGACG00000029134"
## [10177] "ENSGACG00000030529" "ENSGACG00000013977" "ENSGACG00000013990"
## [10180] "ENSGACG00000030539" "ENSGACG00000014006" "ENSGACG00000014011"
## [10183] "ENSGACG00000014019" "ENSGACG00000014030" "ENSGACG00000014042"
## [10186] "ENSGACG00000014046" "ENSGACG00000014062" "ENSGACG00000014071"
## [10189] "ENSGACG00000014097" "ENSGACG00000014128" "ENSGACG00000014142"
## [10192] "ENSGACG00000027780" "ENSGACG00000014183" "ENSGACG00000014197"
## [10195] "ENSGACG00000027930" "ENSGACG00000014199" "ENSGACG00000014206"
## [10198] "ENSGACG00000014219" "ENSGACG00000026182" "ENSGACG00000014229"
## [10201] "ENSGACG00000014237" "ENSGACG00000030349" "ENSGACG00000014259"
## [10204] "ENSGACG00000014273" "ENSGACG00000014277" "ENSGACG00000014292"
## [10207] "ENSGACG00000014293" "ENSGACG00000014307" "ENSGACG00000025493"
## [10210] "ENSGACG00000014325" "ENSGACG00000014327" "ENSGACG00000026565"
## [10213] "ENSGACG00000014331" "ENSGACG00000024814" "ENSGACG00000014340"
## [10216] "ENSGACG00000031863" "ENSGACG00000001239" "ENSGACG00000001244"
## [10219] "ENSGACG00000038034" "ENSGACG00000034216" "ENSGACG00000026831"
## [10222] "ENSGACG00000034136" "ENSGACG00000027012" "ENSGACG00000037220"
## [10225] "ENSGACG00000023148" "ENSGACG00000031398" "ENSGACG00000026099"
## [10228] "ENSGACG00000024776" "ENSGACG00000023098" "ENSGACG00000024909"
## [10231] "ENSGACG00000028221" "ENSGACG00000033667" "ENSGACG00000025312"
## [10234] "ENSGACG00000036031" "ENSGACG00000037195" "ENSGACG00000024198"
## [10237] "ENSGACG00000033953" "ENSGACG00000034385" "ENSGACG00000025453"
## [10240] "ENSGACG00000037156" "ENSGACG00000029645" "ENSGACG00000029488"
## [10243] "ENSGACG00000034930" "ENSGACG00000024994" "ENSGACG00000037802"
## [10246] "ENSGACG00000032733" "ENSGACG00000029208" "ENSGACG00000036369"
## [10249] "ENSGACG00000028458" "ENSGACG00000030581" "ENSGACG00000030302"
## [10252] "ENSGACG00000027130" "ENSGACG00000023069" "ENSGACG00000033041"
## [10255] "ENSGACG00000031238" "ENSGACG00000023750" "ENSGACG00000034043"
## [10258] "ENSGACG00000037373" "ENSGACG00000032949" "ENSGACG00000031288"
## [10261] "ENSGACG00000030878" "ENSGACG00000037077" "ENSGACG00000023586"
## [10264] "ENSGACG00000026658" "ENSGACG00000032276" "ENSGACG00000027158"
## [10267] "ENSGACG00000023550" "ENSGACG00000025926" "ENSGACG00000010162"
## [10270] "ENSGACG00000030161" "ENSGACG00000009343" "ENSGACG00000011847"
## [10273] "ENSGACG00000011790" "ENSGACG00000010745" "ENSGACG00000033409"
## [10276] "ENSGACG00000011493" "ENSGACG00000008259" "ENSGACG00000008469"
## [10279] "ENSGACG00000012118" "ENSGACG00000012255" "ENSGACG00000012509"
## [10282] "ENSGACG00000012616" "ENSGACG00000013001" "ENSGACG00000013950"
## [10285] "ENSGACG00000029896" "ENSGACG00000036101" "ENSGACG00000027254"
## [10288] "ENSGACG00000014104" "ENSGACG00000014159" "ENSGACG00000014218"
## [10291] "ENSGACG00000030067" "ENSGACG00000023916" "ENSGACG00000032644"
## [10294] "ENSGACG00000035090" "ENSGACG00000036886" "ENSGACG00000036230"
## [10297] "ENSGACG00000035197" "ENSGACG00000013676" "ENSGACG00000013675"
## [10300] "ENSGACG00000013668" "ENSGACG00000013657" "ENSGACG00000037405"
## [10303] "ENSGACG00000030566" "ENSGACG00000013620" "ENSGACG00000013607"
## [10306] "ENSGACG00000013601" "ENSGACG00000027623" "ENSGACG00000026222"
## [10309] "ENSGACG00000013581" "ENSGACG00000013574" "ENSGACG00000033930"
## [10312] "ENSGACG00000013572" "ENSGACG00000013566" "ENSGACG00000027534"
## [10315] "ENSGACG00000013561" "ENSGACG00000013557" "ENSGACG00000013516"
## [10318] "ENSGACG00000013511" "ENSGACG00000013504" "ENSGACG00000013507"
## [10321] "ENSGACG00000013501" "ENSGACG00000013470" "ENSGACG00000013402"
## [10324] "ENSGACG00000013398" "ENSGACG00000013396" "ENSGACG00000013391"
## [10327] "ENSGACG00000013381" "ENSGACG00000025393" "ENSGACG00000013337"
## [10330] "ENSGACG00000013330" "ENSGACG00000013327" "ENSGACG00000030011"
## [10333] "ENSGACG00000013308" "ENSGACG00000013285" "ENSGACG00000026514"
## [10336] "ENSGACG00000013259" "ENSGACG00000013249" "ENSGACG00000031446"
## [10339] "ENSGACG00000013230" "ENSGACG00000026692" "ENSGACG00000013220"
## [10342] "ENSGACG00000024609" "ENSGACG00000036052" "ENSGACG00000029856"
## [10345] "ENSGACG00000013211" "ENSGACG00000029858" "ENSGACG00000013206"
## [10348] "ENSGACG00000013202" "ENSGACG00000013194" "ENSGACG00000023488"
## [10351] "ENSGACG00000035806" "ENSGACG00000013181" "ENSGACG00000013174"
## [10354] "ENSGACG00000013171" "ENSGACG00000013157" "ENSGACG00000033150"
## [10357] "ENSGACG00000013150" "ENSGACG00000013112" "ENSGACG00000013148"
## [10360] "ENSGACG00000013142" "ENSGACG00000013133" "ENSGACG00000013128"
## [10363] "ENSGACG00000013086" "ENSGACG00000012682" "ENSGACG00000013084"
## [10366] "ENSGACG00000013077" "ENSGACG00000027812" "ENSGACG00000013054"
## [10369] "ENSGACG00000013037" "ENSGACG00000013032" "ENSGACG00000031387"
## [10372] "ENSGACG00000013026" "ENSGACG00000013021" "ENSGACG00000013013"
## [10375] "ENSGACG00000013008" "ENSGACG00000012989" "ENSGACG00000012978"
## [10378] "ENSGACG00000012963" "ENSGACG00000012958" "ENSGACG00000012955"
## [10381] "ENSGACG00000012939" "ENSGACG00000012932" "ENSGACG00000012913"
## [10384] "ENSGACG00000012908" "ENSGACG00000012893" "ENSGACG00000012887"
## [10387] "ENSGACG00000023120" "ENSGACG00000027566" "ENSGACG00000012828"
## [10390] "ENSGACG00000030417" "ENSGACG00000012820" "ENSGACG00000012810"
## [10393] "ENSGACG00000012802" "ENSGACG00000012786" "ENSGACG00000012754"
## [10396] "ENSGACG00000012750" "ENSGACG00000012650" "ENSGACG00000025058"
## [10399] "ENSGACG00000025885" "ENSGACG00000012727" "ENSGACG00000033381"
## [10402] "ENSGACG00000012718" "ENSGACG00000031565" "ENSGACG00000012706"
## [10405] "ENSGACG00000012694" "ENSGACG00000012467" "ENSGACG00000033234"
## [10408] "ENSGACG00000012688" "ENSGACG00000012443" "ENSGACG00000023875"
## [10411] "ENSGACG00000012432" "ENSGACG00000012423" "ENSGACG00000025306"
## [10414] "ENSGACG00000012413" "ENSGACG00000027467" "ENSGACG00000027854"
## [10417] "ENSGACG00000023453" "ENSGACG00000008712" "ENSGACG00000008359"
## [10420] "ENSGACG00000031935" "ENSGACG00000008337" "ENSGACG00000012632"
## [10423] "ENSGACG00000012618" "ENSGACG00000012601" "ENSGACG00000031059"
## [10426] "ENSGACG00000023253" "ENSGACG00000024627" "ENSGACG00000012578"
## [10429] "ENSGACG00000031427" "ENSGACG00000012559" "ENSGACG00000012528"
## [10432] "ENSGACG00000012513" "ENSGACG00000012505" "ENSGACG00000031266"
## [10435] "ENSGACG00000038018" "ENSGACG00000030217" "ENSGACG00000012488"
## [10438] "ENSGACG00000012482" "ENSGACG00000012475" "ENSGACG00000008682"
## [10441] "ENSGACG00000024661" "ENSGACG00000033404" "ENSGACG00000008667"
## [10444] "ENSGACG00000008637" "ENSGACG00000034568" "ENSGACG00000008615"
## [10447] "ENSGACG00000008593" "ENSGACG00000008559" "ENSGACG00000033087"
## [10450] "ENSGACG00000008576" "ENSGACG00000008552" "ENSGACG00000008538"
## [10453] "ENSGACG00000008513" "ENSGACG00000008509" "ENSGACG00000008481"
## [10456] "ENSGACG00000008472" "ENSGACG00000008435" "ENSGACG00000028826"
## [10459] "ENSGACG00000032832" "ENSGACG00000035346" "ENSGACG00000008428"
## [10462] "ENSGACG00000025502" "ENSGACG00000008400" "ENSGACG00000013887"
## [10465] "ENSGACG00000013878" "ENSGACG00000013875" "ENSGACG00000013866"
## [10468] "ENSGACG00000013863" "ENSGACG00000030762" "ENSGACG00000013802"
## [10471] "ENSGACG00000013795" "ENSGACG00000013792" "ENSGACG00000026677"
## [10474] "ENSGACG00000013774" "ENSGACG00000013752" "ENSGACG00000034081"
## [10477] "ENSGACG00000013749" "ENSGACG00000013745" "ENSGACG00000033135"
## [10480] "ENSGACG00000013722" "ENSGACG00000013718" "ENSGACG00000013692"
## [10483] "ENSGACG00000013691" "ENSGACG00000013685" "ENSGACG00000013680"
## [10486] "ENSGACG00000035539" "ENSGACG00000028537" "ENSGACG00000008327"
## [10489] "ENSGACG00000008317" "ENSGACG00000008229" "ENSGACG00000008221"
## [10492] "ENSGACG00000008216" "ENSGACG00000008212" "ENSGACG00000008182"
## [10495] "ENSGACG00000008146" "ENSGACG00000008133" "ENSGACG00000008123"
## [10498] "ENSGACG00000008106" "ENSGACG00000008098" "ENSGACG00000023347"
## [10501] "ENSGACG00000003427" "ENSGACG00000003441" "ENSGACG00000036763"
## [10504] "ENSGACG00000027732" "ENSGACG00000003473" "ENSGACG00000027873"
## [10507] "ENSGACG00000003476" "ENSGACG00000014009" "ENSGACG00000014000"
## [10510] "ENSGACG00000027742" "ENSGACG00000013968" "ENSGACG00000032016"
## [10513] "ENSGACG00000029420" "ENSGACG00000013955" "ENSGACG00000013953"
## [10516] "ENSGACG00000013932" "ENSGACG00000013909" "ENSGACG00000013903"
## [10519] "ENSGACG00000013897" "ENSGACG00000029342" "ENSGACG00000008084"
## [10522] "ENSGACG00000008076" "ENSGACG00000026221" "ENSGACG00000030795"
## [10525] "ENSGACG00000037185" "ENSGACG00000008776" "ENSGACG00000008767"
## [10528] "ENSGACG00000008758" "ENSGACG00000008750" "ENSGACG00000009471"
## [10531] "ENSGACG00000009467" "ENSGACG00000009452" "ENSGACG00000009449"
## [10534] "ENSGACG00000009447" "ENSGACG00000025707" "ENSGACG00000009427"
## [10537] "ENSGACG00000009412" "ENSGACG00000009368" "ENSGACG00000009364"
## [10540] "ENSGACG00000009330" "ENSGACG00000000876" "ENSGACG00000000885"
## [10543] "ENSGACG00000000888" "ENSGACG00000000896" "ENSGACG00000027161"
## [10546] "ENSGACG00000000909" "ENSGACG00000000919" "ENSGACG00000000923"
## [10549] "ENSGACG00000000939" "ENSGACG00000000950" "ENSGACG00000032237"
## [10552] "ENSGACG00000025302" "ENSGACG00000028484" "ENSGACG00000031411"
## [10555] "ENSGACG00000017737" "ENSGACG00000032181" "ENSGACG00000029150"
## [10558] "ENSGACG00000017747" "ENSGACG00000023334" "ENSGACG00000017749"
## [10561] "ENSGACG00000017752" "ENSGACG00000027520" "ENSGACG00000037778"
## [10564] "ENSGACG00000017756" "ENSGACG00000017759" "ENSGACG00000017763"
## [10567] "ENSGACG00000017768" "ENSGACG00000017775" "ENSGACG00000017782"
## [10570] "ENSGACG00000025661" "ENSGACG00000002942" "ENSGACG00000002947"
## [10573] "ENSGACG00000002958" "ENSGACG00000025683" "ENSGACG00000002960"
## [10576] "ENSGACG00000002963" "ENSGACG00000002968" "ENSGACG00000002977"
## [10579] "ENSGACG00000027316" "ENSGACG00000003006" "ENSGACG00000003010"
## [10582] "ENSGACG00000026151" "ENSGACG00000003015" "ENSGACG00000003034"
## [10585] "ENSGACG00000003037" "ENSGACG00000035800" "ENSGACG00000003080"
## [10588] "ENSGACG00000003084" "ENSGACG00000032678" "ENSGACG00000003101"
## [10591] "ENSGACG00000003123" "ENSGACG00000003136" "ENSGACG00000025338"
## [10594] "ENSGACG00000030917" "ENSGACG00000003143" "ENSGACG00000003156"
## [10597] "ENSGACG00000026849" "ENSGACG00000003171" "ENSGACG00000003178"
## [10600] "ENSGACG00000003185" "ENSGACG00000003189" "ENSGACG00000024610"
## [10603] "ENSGACG00000003205" "ENSGACG00000029749" "ENSGACG00000003217"
## [10606] "ENSGACG00000003223" "ENSGACG00000032285" "ENSGACG00000034524"
## [10609] "ENSGACG00000001688" "ENSGACG00000001692" "ENSGACG00000001695"
## [10612] "ENSGACG00000001704" "ENSGACG00000001711" "ENSGACG00000001720"
## [10615] "ENSGACG00000037226" "ENSGACG00000001732" "ENSGACG00000001740"
## [10618] "ENSGACG00000036977" "ENSGACG00000001745" "ENSGACG00000001754"
## [10621] "ENSGACG00000035041" "ENSGACG00000001768" "ENSGACG00000001787"
## [10624] "ENSGACG00000027717" "ENSGACG00000001790" "ENSGACG00000001803"
## [10627] "ENSGACG00000001806" "ENSGACG00000001819" "ENSGACG00000029062"
## [10630] "ENSGACG00000031147" "ENSGACG00000003230" "ENSGACG00000003234"
## [10633] "ENSGACG00000003235" "ENSGACG00000003236" "ENSGACG00000003238"
## [10636] "ENSGACG00000003397" "ENSGACG00000037322" "ENSGACG00000003401"
## [10639] "ENSGACG00000003414" "ENSGACG00000009299" "ENSGACG00000009260"
## [10642] "ENSGACG00000029584" "ENSGACG00000009238" "ENSGACG00000037192"
## [10645] "ENSGACG00000031218" "ENSGACG00000009217" "ENSGACG00000029514"
## [10648] "ENSGACG00000037016" "ENSGACG00000009206" "ENSGACG00000009182"
## [10651] "ENSGACG00000031571" "ENSGACG00000009169" "ENSGACG00000009138"
## [10654] "ENSGACG00000009133" "ENSGACG00000009128" "ENSGACG00000009118"
## [10657] "ENSGACG00000009093" "ENSGACG00000009082" "ENSGACG00000031268"
## [10660] "ENSGACG00000009075" "ENSGACG00000009064" "ENSGACG00000009056"
## [10663] "ENSGACG00000009048" "ENSGACG00000032805" "ENSGACG00000009041"
## [10666] "ENSGACG00000009027" "ENSGACG00000009022" "ENSGACG00000029990"
## [10669] "ENSGACG00000009004" "ENSGACG00000008994" "ENSGACG00000008984"
## [10672] "ENSGACG00000008982" "ENSGACG00000008951" "ENSGACG00000008936"
## [10675] "ENSGACG00000008925" "ENSGACG00000008911" "ENSGACG00000008834"
## [10678] "ENSGACG00000008909" "ENSGACG00000030114" "ENSGACG00000024677"
## [10681] "ENSGACG00000012351" "ENSGACG00000012379" "ENSGACG00000012366"
## [10684] "ENSGACG00000012361" "ENSGACG00000037939" "ENSGACG00000012360"
## [10687] "ENSGACG00000035133" "ENSGACG00000012345" "ENSGACG00000012340"
## [10690] "ENSGACG00000027329" "ENSGACG00000012315" "ENSGACG00000012310"
## [10693] "ENSGACG00000012292" "ENSGACG00000012308" "ENSGACG00000012277"
## [10696] "ENSGACG00000024492" "ENSGACG00000012254" "ENSGACG00000035186"
## [10699] "ENSGACG00000012251" "ENSGACG00000012247" "ENSGACG00000012231"
## [10702] "ENSGACG00000028299" "ENSGACG00000026921" "ENSGACG00000012182"
## [10705] "ENSGACG00000012175" "ENSGACG00000028425" "ENSGACG00000033837"
## [10708] "ENSGACG00000012171" "ENSGACG00000012156" "ENSGACG00000035957"
## [10711] "ENSGACG00000012147" "ENSGACG00000012120" "ENSGACG00000030534"
## [10714] "ENSGACG00000012100" "ENSGACG00000036608" "ENSGACG00000012083"
## [10717] "ENSGACG00000012077" "ENSGACG00000012045" "ENSGACG00000025986"
## [10720] "ENSGACG00000012038" "ENSGACG00000012018" "ENSGACG00000012002"
## [10723] "ENSGACG00000011991" "ENSGACG00000011983" "ENSGACG00000029190"
## [10726] "ENSGACG00000011970" "ENSGACG00000027086" "ENSGACG00000029231"
## [10729] "ENSGACG00000029994" "ENSGACG00000011965" "ENSGACG00000011957"
## [10732] "ENSGACG00000011951" "ENSGACG00000011942" "ENSGACG00000011934"
## [10735] "ENSGACG00000011925" "ENSGACG00000011917" "ENSGACG00000011874"
## [10738] "ENSGACG00000031385" "ENSGACG00000011859" "ENSGACG00000011848"
## [10741] "ENSGACG00000011841" "ENSGACG00000011829" "ENSGACG00000011820"
## [10744] "ENSGACG00000035623" "ENSGACG00000011803" "ENSGACG00000032078"
## [10747] "ENSGACG00000011792" "ENSGACG00000011789" "ENSGACG00000011734"
## [10750] "ENSGACG00000011722" "ENSGACG00000011707" "ENSGACG00000011695"
## [10753] "ENSGACG00000011686" "ENSGACG00000011675" "ENSGACG00000011669"
## [10756] "ENSGACG00000011657" "ENSGACG00000011641" "ENSGACG00000011639"
## [10759] "ENSGACG00000036785" "ENSGACG00000011621" "ENSGACG00000027511"
## [10762] "ENSGACG00000011600" "ENSGACG00000011547" "ENSGACG00000028758"
## [10765] "ENSGACG00000011515" "ENSGACG00000036125" "ENSGACG00000011507"
## [10768] "ENSGACG00000026295" "ENSGACG00000011489" "ENSGACG00000011464"
## [10771] "ENSGACG00000026458" "ENSGACG00000032197" "ENSGACG00000011451"
## [10774] "ENSGACG00000011436" "ENSGACG00000010761" "ENSGACG00000010757"
## [10777] "ENSGACG00000010754" "ENSGACG00000028016" "ENSGACG00000010744"
## [10780] "ENSGACG00000031028" "ENSGACG00000010736" "ENSGACG00000010726"
## [10783] "ENSGACG00000010708" "ENSGACG00000010704" "ENSGACG00000010686"
## [10786] "ENSGACG00000031858" "ENSGACG00000035274" "ENSGACG00000027707"
## [10789] "ENSGACG00000010664" "ENSGACG00000034946" "ENSGACG00000010654"
## [10792] "ENSGACG00000010650" "ENSGACG00000010646" "ENSGACG00000010641"
## [10795] "ENSGACG00000026179" "ENSGACG00000031776" "ENSGACG00000010632"
## [10798] "ENSGACG00000010622" "ENSGACG00000028468" "ENSGACG00000010614"
## [10801] "ENSGACG00000010601" "ENSGACG00000023078" "ENSGACG00000032141"
## [10804] "ENSGACG00000010580" "ENSGACG00000034791" "ENSGACG00000010556"
## [10807] "ENSGACG00000027489" "ENSGACG00000031424" "ENSGACG00000010530"
## [10810] "ENSGACG00000010521" "ENSGACG00000026900" "ENSGACG00000033757"
## [10813] "ENSGACG00000010508" "ENSGACG00000010504" "ENSGACG00000035335"
## [10816] "ENSGACG00000010483" "ENSGACG00000010474" "ENSGACG00000010470"
## [10819] "ENSGACG00000010466" "ENSGACG00000010464" "ENSGACG00000010446"
## [10822] "ENSGACG00000010442" "ENSGACG00000010438" "ENSGACG00000010432"
## [10825] "ENSGACG00000010408" "ENSGACG00000010380" "ENSGACG00000010403"
## [10828] "ENSGACG00000010357" "ENSGACG00000010348" "ENSGACG00000022976"
## [10831] "ENSGACG00000026329" "ENSGACG00000010330" "ENSGACG00000010317"
## [10834] "ENSGACG00000023705" "ENSGACG00000010292" "ENSGACG00000010291"
## [10837] "ENSGACG00000010219" "ENSGACG00000010215" "ENSGACG00000010207"
## [10840] "ENSGACG00000010200" "ENSGACG00000031593" "ENSGACG00000034997"
## [10843] "ENSGACG00000037071" "ENSGACG00000025547" "ENSGACG00000010169"
## [10846] "ENSGACG00000035208" "ENSGACG00000010128" "ENSGACG00000026823"
## [10849] "ENSGACG00000010111" "ENSGACG00000007244" "ENSGACG00000007261"
## [10852] "ENSGACG00000007253" "ENSGACG00000032172" "ENSGACG00000005298"
## [10855] "ENSGACG00000037082" "ENSGACG00000025917" "ENSGACG00000005841"
## [10858] "ENSGACG00000010099" "ENSGACG00000005839" "ENSGACG00000030090"
## [10861] "ENSGACG00000005763" "ENSGACG00000006535" "ENSGACG00000006531"
## [10864] "ENSGACG00000007225" "ENSGACG00000007199" "ENSGACG00000006520"
## [10867] "ENSGACG00000006485" "ENSGACG00000006462" "ENSGACG00000023876"
## [10870] "ENSGACG00000037359" "ENSGACG00000028643" "ENSGACG00000006446"
## [10873] "ENSGACG00000006438" "ENSGACG00000006397" "ENSGACG00000006387"
## [10876] "ENSGACG00000006377" "ENSGACG00000006371" "ENSGACG00000034425"
## [10879] "ENSGACG00000006345" "ENSGACG00000006337" "ENSGACG00000006328"
## [10882] "ENSGACG00000006307" "ENSGACG00000010089" "ENSGACG00000038112"
## [10885] "ENSGACG00000010056" "ENSGACG00000010016" "ENSGACG00000026289"
## [10888] "ENSGACG00000032249" "ENSGACG00000009950" "ENSGACG00000009917"
## [10891] "ENSGACG00000009910" "ENSGACG00000009907" "ENSGACG00000009904"
## [10894] "ENSGACG00000009868" "ENSGACG00000009861" "ENSGACG00000009840"
## [10897] "ENSGACG00000024684" "ENSGACG00000009832" "ENSGACG00000009825"
## [10900] "ENSGACG00000009793" "ENSGACG00000009781" "ENSGACG00000009774"
## [10903] "ENSGACG00000009765" "ENSGACG00000009757" "ENSGACG00000009666"
## [10906] "ENSGACG00000009635" "ENSGACG00000033842" "ENSGACG00000009630"
## [10909] "ENSGACG00000028404" "ENSGACG00000035579" "ENSGACG00000009618"
## [10912] "ENSGACG00000009601" "ENSGACG00000009564" "ENSGACG00000026138"
## [10915] "ENSGACG00000009560" "ENSGACG00000009524" "ENSGACG00000006276"
## [10918] "ENSGACG00000006234" "ENSGACG00000006206" "ENSGACG00000032851"
## [10921] "ENSGACG00000006167" "ENSGACG00000006161" "ENSGACG00000006144"
## [10924] "ENSGACG00000006127" "ENSGACG00000006121" "ENSGACG00000029291"
## [10927] "ENSGACG00000006095" "ENSGACG00000031860" "ENSGACG00000006080"
## [10930] "ENSGACG00000006060" "ENSGACG00000031752" "ENSGACG00000006045"
## [10933] "ENSGACG00000006037" "ENSGACG00000006021" "ENSGACG00000006003"
## [10936] "ENSGACG00000023919" "ENSGACG00000026127" "ENSGACG00000005994"
## [10939] "ENSGACG00000005973" "ENSGACG00000005967" "ENSGACG00000005864"
## [10942] "ENSGACG00000005863" "ENSGACG00000037941" "ENSGACG00000005855"
## [10945] "ENSGACG00000005850" "ENSGACG00000005535" "ENSGACG00000005507"
## [10948] "ENSGACG00000025449" "ENSGACG00000005248" "ENSGACG00000005223"
## [10951] "ENSGACG00000005192" "ENSGACG00000023257" "ENSGACG00000005178"
## [10954] "ENSGACG00000031637" "ENSGACG00000005169" "ENSGACG00000005136"
## [10957] "ENSGACG00000003729" "ENSGACG00000037834" "ENSGACG00000003712"
## [10960] "ENSGACG00000003696" "ENSGACG00000003692" "ENSGACG00000003686"
## [10963] "ENSGACG00000003679" "ENSGACG00000003671" "ENSGACG00000003655"
## [10966] "ENSGACG00000025512" "ENSGACG00000003590" "ENSGACG00000036930"
## [10969] "ENSGACG00000003572" "ENSGACG00000005122" "ENSGACG00000022912"
## [10972] "ENSGACG00000003743" "ENSGACG00000003733" "ENSGACG00000024818"
## [10975] "ENSGACG00000005705" "ENSGACG00000005685" "ENSGACG00000028153"
## [10978] "ENSGACG00000005669" "ENSGACG00000005666" "ENSGACG00000005646"
## [10981] "ENSGACG00000005615" "ENSGACG00000005611" "ENSGACG00000006293"
## [10984] "ENSGACG00000005503" "ENSGACG00000005603" "ENSGACG00000005481"
## [10987] "ENSGACG00000005476" "ENSGACG00000033535" "ENSGACG00000005431"
## [10990] "ENSGACG00000009485" "ENSGACG00000009481" "ENSGACG00000027124"
## [10993] "ENSGACG00000028789" "ENSGACG00000036377" "ENSGACG00000026300"
## [10996] "ENSGACG00000024475" "ENSGACG00000031229" "ENSGACG00000029816"
## [10999] "ENSGACG00000017758" "ENSGACG00000032978" "ENSGACG00000024356"
## [11002] "ENSGACG00000032719" "ENSGACG00000023520" "ENSGACG00000028864"
## [11005] "ENSGACG00000024019" "ENSGACG00000023658" "ENSGACG00000033055"
## [11008] "ENSGACG00000024964" "ENSGACG00000027611" "ENSGACG00000024861"
## [11011] "ENSGACG00000028992" "ENSGACG00000033781" "ENSGACG00000034609"
## [11014] "ENSGACG00000031382" "ENSGACG00000023716" "ENSGACG00000032401"
## [11017] "ENSGACG00000038051" "ENSGACG00000034833" "ENSGACG00000024466"
## [11020] "ENSGACG00000028762" "ENSGACG00000024324" "ENSGACG00000029570"
## [11023] "ENSGACG00000024972" "ENSGACG00000038136" "ENSGACG00000027706"
## [11026] "ENSGACG00000036613" "ENSGACG00000036709" "ENSGACG00000033743"
## [11029] "ENSGACG00000032768" "ENSGACG00000037494" "ENSGACG00000037560"
## [11032] "ENSGACG00000028264" "ENSGACG00000034204" "ENSGACG00000028137"
## [11035] "ENSGACG00000036315" "ENSGACG00000024815" "ENSGACG00000029263"
## [11038] "ENSGACG00000025005" "ENSGACG00000027669" "ENSGACG00000003134"
## [11041] "ENSGACG00000030675" "ENSGACG00000031271" "ENSGACG00000037237"
## [11044] "ENSGACG00000025304" "ENSGACG00000036204" "ENSGACG00000028711"
## [11047] "ENSGACG00000029223" "ENSGACG00000012665" "ENSGACG00000012968"
## [11050] "ENSGACG00000012453" "ENSGACG00000005408" "ENSGACG00000023575"
## [11053] "ENSGACG00000005380" "ENSGACG00000008608" "ENSGACG00000031061"
## [11056] "ENSGACG00000034329" "ENSGACG00000038004" "ENSGACG00000002987"
## [11059] "ENSGACG00000005305" "ENSGACG00000012131" "ENSGACG00000031517"
## [11062] "ENSGACG00000025105" "ENSGACG00000003773" "ENSGACG00000028852"
## [11065] "ENSGACG00000003765" "ENSGACG00000003744" "ENSGACG00000006552"
## [11068] "ENSGACG00000003780" "ENSGACG00000007286" "ENSGACG00000003557"
## [11071] "ENSGACG00000003545" "ENSGACG00000027614" "ENSGACG00000024449"
## [11074] "ENSGACG00000003524" "ENSGACG00000003519" "ENSGACG00000003506"
## [11077] "ENSGACG00000003499" "ENSGACG00000003494" "ENSGACG00000014012"
## [11080] "ENSGACG00000014015" "ENSGACG00000032671" "ENSGACG00000014036"
## [11083] "ENSGACG00000014041" "ENSGACG00000014044" "ENSGACG00000014061"
## [11086] "ENSGACG00000014078" "ENSGACG00000014083" "ENSGACG00000014110"
## [11089] "ENSGACG00000014132" "ENSGACG00000014141" "ENSGACG00000014149"
## [11092] "ENSGACG00000029972" "ENSGACG00000014157" "ENSGACG00000014176"
## [11095] "ENSGACG00000037319" "ENSGACG00000014194" "ENSGACG00000014204"
## [11098] "ENSGACG00000014214" "ENSGACG00000036460" "ENSGACG00000028859"
## [11101] "ENSGACG00000026072" "ENSGACG00000006642" "ENSGACG00000027662"
## [11104] "ENSGACG00000006632" "ENSGACG00000006622" "ENSGACG00000037170"
## [11107] "ENSGACG00000006587" "ENSGACG00000033307" "ENSGACG00000006614"
## [11110] "ENSGACG00000007269" "ENSGACG00000006610" "ENSGACG00000006607"
## [11113] "ENSGACG00000025318" "ENSGACG00000006572" "ENSGACG00000005587"
## [11116] "ENSGACG00000005553" "ENSGACG00000005547" "ENSGACG00000005544"
## [11119] "ENSGACG00000007188" "ENSGACG00000007178" "ENSGACG00000007170"
## [11122] "ENSGACG00000007163" "ENSGACG00000007151" "ENSGACG00000007141"
## [11125] "ENSGACG00000007130" "ENSGACG00000033645" "ENSGACG00000007099"
## [11128] "ENSGACG00000007087" "ENSGACG00000007065" "ENSGACG00000007061"
## [11131] "ENSGACG00000007056" "ENSGACG00000007051" "ENSGACG00000034934"
## [11134] "ENSGACG00000007035" "ENSGACG00000007020" "ENSGACG00000007004"
## [11137] "ENSGACG00000006984" "ENSGACG00000027786" "ENSGACG00000006955"
## [11140] "ENSGACG00000006944" "ENSGACG00000006912" "ENSGACG00000006901"
## [11143] "ENSGACG00000006883" "ENSGACG00000006707" "ENSGACG00000006706"
## [11146] "ENSGACG00000006690" "ENSGACG00000027839" "ENSGACG00000008067"
## [11149] "ENSGACG00000008062" "ENSGACG00000008036" "ENSGACG00000008032"
## [11152] "ENSGACG00000008011" "ENSGACG00000007994" "ENSGACG00000007982"
## [11155] "ENSGACG00000007971" "ENSGACG00000007933" "ENSGACG00000007921"
## [11158] "ENSGACG00000035662" "ENSGACG00000034924" "ENSGACG00000007897"
## [11161] "ENSGACG00000007879" "ENSGACG00000029146" "ENSGACG00000007871"
## [11164] "ENSGACG00000007828" "ENSGACG00000007770" "ENSGACG00000032467"
## [11167] "ENSGACG00000007756" "ENSGACG00000026109" "ENSGACG00000027982"
## [11170] "ENSGACG00000007736" "ENSGACG00000007723" "ENSGACG00000007711"
## [11173] "ENSGACG00000007622" "ENSGACG00000007687" "ENSGACG00000007677"
## [11176] "ENSGACG00000007668" "ENSGACG00000038028" "ENSGACG00000007659"
## [11179] "ENSGACG00000007639" "ENSGACG00000007600" "ENSGACG00000007569"
## [11182] "ENSGACG00000007563" "ENSGACG00000036182" "ENSGACG00000023498"
## [11185] "ENSGACG00000024634" "ENSGACG00000023254" "ENSGACG00000030243"
## [11188] "ENSGACG00000007532" "ENSGACG00000007526" "ENSGACG00000007508"
## [11191] "ENSGACG00000025805" "ENSGACG00000032177" "ENSGACG00000024423"
## [11194] "ENSGACG00000032426" "ENSGACG00000027543" "ENSGACG00000030158"
## [11197] "ENSGACG00000025655" "ENSGACG00000025624" "ENSGACG00000029766"
## [11200] "ENSGACG00000034706" "ENSGACG00000030557" "ENSGACG00000007410"
## [11203] "ENSGACG00000028998" "ENSGACG00000029851" "ENSGACG00000031536"
## [11206] "ENSGACG00000007392" "ENSGACG00000029770" "ENSGACG00000023516"
## [11209] "ENSGACG00000007379" "ENSGACG00000007354" "ENSGACG00000030227"
## [11212] "ENSGACG00000007346" "ENSGACG00000007316" "ENSGACG00000000056"
## [11215] "ENSGACG00000005040" "ENSGACG00000005032" "ENSGACG00000005018"
## [11218] "ENSGACG00000005006" "ENSGACG00000004989" "ENSGACG00000004983"
## [11221] "ENSGACG00000004979" "ENSGACG00000004972" "ENSGACG00000004969"
## [11224] "ENSGACG00000023837" "ENSGACG00000004958" "ENSGACG00000004946"
## [11227] "ENSGACG00000004932" "ENSGACG00000004928" "ENSGACG00000004881"
## [11230] "ENSGACG00000004840" "ENSGACG00000004824" "ENSGACG00000004785"
## [11233] "ENSGACG00000004783" "ENSGACG00000004775" "ENSGACG00000004766"
## [11236] "ENSGACG00000004743" "ENSGACG00000004720" "ENSGACG00000004642"
## [11239] "ENSGACG00000004632" "ENSGACG00000004621" "ENSGACG00000004615"
## [11242] "ENSGACG00000004577" "ENSGACG00000036918" "ENSGACG00000004550"
## [11245] "ENSGACG00000004511" "ENSGACG00000004492" "ENSGACG00000004480"
## [11248] "ENSGACG00000036261" "ENSGACG00000026525" "ENSGACG00000004561"
## [11251] "ENSGACG00000004473" "ENSGACG00000004457" "ENSGACG00000004429"
## [11254] "ENSGACG00000004425" "ENSGACG00000004419" "ENSGACG00000004406"
## [11257] "ENSGACG00000004392" "ENSGACG00000004385" "ENSGACG00000004382"
## [11260] "ENSGACG00000035712" "ENSGACG00000004349" "ENSGACG00000004294"
## [11263] "ENSGACG00000004194" "ENSGACG00000004191" "ENSGACG00000004180"
## [11266] "ENSGACG00000004170" "ENSGACG00000004015" "ENSGACG00000028647"
## [11269] "ENSGACG00000003805" "ENSGACG00000003983" "ENSGACG00000003971"
## [11272] "ENSGACG00000003836" "ENSGACG00000003811" "ENSGACG00000003799"
## [11275] "ENSGACG00000003796" "ENSGACG00000022969" "ENSGACG00000003781"
## [11278] "ENSGACG00000035929" "ENSGACG00000036704" "ENSGACG00000032702"
## [11281] "ENSGACG00000029736" "ENSGACG00000036607" "ENSGACG00000029039"
## [11284] "ENSGACG00000037335" "ENSGACG00000023698" "ENSGACG00000034274"
## [11287] "ENSGACG00000031741" "ENSGACG00000023700" "ENSGACG00000034345"
## [11290] "ENSGACG00000036441" "ENSGACG00000024865" "ENSGACG00000035846"
## [11293] "ENSGACG00000030535" "ENSGACG00000035985" "ENSGACG00000024429"
## [11296] "ENSGACG00000028582" "ENSGACG00000025020" "ENSGACG00000033092"
## [11299] "ENSGACG00000030876" "ENSGACG00000036183" "ENSGACG00000036916"
## [11302] "ENSGACG00000037650" "ENSGACG00000037147" "ENSGACG00000036564"
## [11305] "ENSGACG00000029103" "ENSGACG00000028517" "ENSGACG00000029769"
## [11308] "ENSGACG00000037036" "ENSGACG00000025684" "ENSGACG00000030761"
## [11311] "ENSGACG00000025968" "ENSGACG00000034067" "ENSGACG00000030248"
## [11314] "ENSGACG00000029771" "ENSGACG00000033723" "ENSGACG00000031656"
## [11317] "ENSGACG00000032391" "ENSGACG00000036941" "ENSGACG00000025131"
## [11320] "ENSGACG00000028298" "ENSGACG00000026020" "ENSGACG00000031124"
## [11323] "ENSGACG00000033053" "ENSGACG00000007461" "ENSGACG00000004471"
## [11326] "ENSGACG00000003789" "ENSGACG00000006854" "ENSGACG00000000764"
## [11329] "ENSGACG00000000755" "ENSGACG00000000754" "ENSGACG00000000750"
## [11332] "ENSGACG00000030237" "ENSGACG00000000747" "ENSGACG00000003488"
## [11335] "ENSGACG00000003490" "ENSGACG00000003495" "ENSGACG00000003513"
## [11338] "ENSGACG00000003059" "ENSGACG00000001660" "ENSGACG00000027460"
## [11341] "ENSGACG00000001649" "ENSGACG00000001644" "ENSGACG00000029177"
## [11344] "ENSGACG00000006819" "ENSGACG00000004797" "ENSGACG00000004819"
## [11347] "ENSGACG00000006946" "ENSGACG00000031922" "ENSGACG00000033644"
## [11350] "ENSGACG00000006983" "ENSGACG00000006994" "ENSGACG00000006997"
## [11353] "ENSGACG00000007041" "ENSGACG00000007055" "ENSGACG00000033363"
## [11356] "ENSGACG00000007138" "ENSGACG00000007204" "ENSGACG00000012098"
## [11359] "ENSGACG00000007265" "ENSGACG00000012102" "ENSGACG00000026145"
## [11362] "ENSGACG00000031081" "ENSGACG00000012117" "ENSGACG00000007339"
## [11365] "ENSGACG00000036452" "ENSGACG00000007353" "ENSGACG00000012145"
## [11368] "ENSGACG00000007358" "ENSGACG00000012164" "ENSGACG00000012197"
## [11371] "ENSGACG00000007365" "ENSGACG00000012267" "ENSGACG00000007398"
## [11374] "ENSGACG00000012225" "ENSGACG00000027261" "ENSGACG00000007405"
## [11377] "ENSGACG00000007417" "ENSGACG00000037837" "ENSGACG00000007453"
## [11380] "ENSGACG00000007462" "ENSGACG00000007465" "ENSGACG00000007471"
## [11383] "ENSGACG00000007481" "ENSGACG00000035200" "ENSGACG00000037332"
## [11386] "ENSGACG00000012259" "ENSGACG00000007519" "ENSGACG00000031736"
## [11389] "ENSGACG00000012289" "ENSGACG00000007531" "ENSGACG00000012301"
## [11392] "ENSGACG00000007539" "ENSGACG00000028784" "ENSGACG00000029975"
## [11395] "ENSGACG00000001715" "ENSGACG00000001703" "ENSGACG00000022961"
## [11398] "ENSGACG00000001693" "ENSGACG00000029867" "ENSGACG00000001683"
## [11401] "ENSGACG00000001677" "ENSGACG00000001672" "ENSGACG00000001670"
## [11404] "ENSGACG00000001667" "ENSGACG00000001665" "ENSGACG00000003054"
## [11407] "ENSGACG00000036388" "ENSGACG00000003043" "ENSGACG00000003027"
## [11410] "ENSGACG00000003035" "ENSGACG00000032664" "ENSGACG00000026709"
## [11413] "ENSGACG00000003484" "ENSGACG00000003426" "ENSGACG00000033086"
## [11416] "ENSGACG00000036741" "ENSGACG00000003396" "ENSGACG00000036625"
## [11419] "ENSGACG00000003387" "ENSGACG00000003376" "ENSGACG00000003342"
## [11422] "ENSGACG00000003330" "ENSGACG00000003325" "ENSGACG00000003311"
## [11425] "ENSGACG00000003290" "ENSGACG00000003284" "ENSGACG00000031241"
## [11428] "ENSGACG00000003262" "ENSGACG00000003218" "ENSGACG00000003210"
## [11431] "ENSGACG00000003203" "ENSGACG00000003177" "ENSGACG00000003124"
## [11434] "ENSGACG00000003103" "ENSGACG00000003065" "ENSGACG00000030687"
## [11437] "ENSGACG00000000793" "ENSGACG00000000787" "ENSGACG00000000786"
## [11440] "ENSGACG00000000771" "ENSGACG00000000766" "ENSGACG00000004899"
## [11443] "ENSGACG00000004904" "ENSGACG00000004918" "ENSGACG00000034117"
## [11446] "ENSGACG00000004941" "ENSGACG00000004954" "ENSGACG00000004985"
## [11449] "ENSGACG00000005005" "ENSGACG00000029435" "ENSGACG00000036157"
## [11452] "ENSGACG00000005015" "ENSGACG00000005023" "ENSGACG00000005034"
## [11455] "ENSGACG00000005056" "ENSGACG00000005095" "ENSGACG00000005105"
## [11458] "ENSGACG00000005123" "ENSGACG00000005132" "ENSGACG00000005144"
## [11461] "ENSGACG00000025186" "ENSGACG00000031564" "ENSGACG00000024790"
## [11464] "ENSGACG00000004013" "ENSGACG00000026050" "ENSGACG00000004030"
## [11467] "ENSGACG00000028601" "ENSGACG00000004043" "ENSGACG00000004054"
## [11470] "ENSGACG00000031176" "ENSGACG00000004058" "ENSGACG00000004064"
## [11473] "ENSGACG00000004073" "ENSGACG00000004077" "ENSGACG00000004094"
## [11476] "ENSGACG00000004097" "ENSGACG00000023969" "ENSGACG00000004106"
## [11479] "ENSGACG00000004138" "ENSGACG00000004147" "ENSGACG00000004151"
## [11482] "ENSGACG00000004202" "ENSGACG00000004205" "ENSGACG00000004211"
## [11485] "ENSGACG00000004233" "ENSGACG00000004239" "ENSGACG00000004249"
## [11488] "ENSGACG00000004262" "ENSGACG00000004277" "ENSGACG00000028684"
## [11491] "ENSGACG00000004295" "ENSGACG00000003522" "ENSGACG00000033476"
## [11494] "ENSGACG00000003555" "ENSGACG00000023162" "ENSGACG00000003559"
## [11497] "ENSGACG00000003568" "ENSGACG00000003571" "ENSGACG00000028631"
## [11500] "ENSGACG00000026312" "ENSGACG00000036817" "ENSGACG00000003594"
## [11503] "ENSGACG00000027073" "ENSGACG00000003610" "ENSGACG00000003636"
## [11506] "ENSGACG00000025708" "ENSGACG00000031327" "ENSGACG00000003680"
## [11509] "ENSGACG00000003697" "ENSGACG00000029900" "ENSGACG00000026508"
## [11512] "ENSGACG00000003737" "ENSGACG00000003741" "ENSGACG00000003747"
## [11515] "ENSGACG00000003768" "ENSGACG00000003793" "ENSGACG00000003815"
## [11518] "ENSGACG00000003825" "ENSGACG00000003831" "ENSGACG00000003866"
## [11521] "ENSGACG00000003871" "ENSGACG00000027285" "ENSGACG00000003884"
## [11524] "ENSGACG00000003887" "ENSGACG00000003890" "ENSGACG00000027137"
## [11527] "ENSGACG00000003910" "ENSGACG00000003914" "ENSGACG00000003924"
## [11530] "ENSGACG00000003930" "ENSGACG00000003935" "ENSGACG00000030710"
## [11533] "ENSGACG00000003953" "ENSGACG00000003959" "ENSGACG00000003980"
## [11536] "ENSGACG00000001561" "ENSGACG00000001560" "ENSGACG00000027877"
## [11539] "ENSGACG00000031773" "ENSGACG00000032679" "ENSGACG00000034371"
## [11542] "ENSGACG00000004331" "ENSGACG00000004342" "ENSGACG00000024664"
## [11545] "ENSGACG00000025394" "ENSGACG00000033364" "ENSGACG00000026759"
## [11548] "ENSGACG00000004389" "ENSGACG00000001612" "ENSGACG00000001610"
## [11551] "ENSGACG00000001597" "ENSGACG00000004874" "ENSGACG00000004880"
## [11554] "ENSGACG00000004889" "ENSGACG00000001171" "ENSGACG00000001593"
## [11557] "ENSGACG00000001591" "ENSGACG00000033550" "ENSGACG00000027345"
## [11560] "ENSGACG00000001586" "ENSGACG00000001573" "ENSGACG00000001569"
## [11563] "ENSGACG00000024645" "ENSGACG00000001565" "ENSGACG00000026526"
## [11566] "ENSGACG00000004412" "ENSGACG00000005875" "ENSGACG00000004433"
## [11569] "ENSGACG00000026978" "ENSGACG00000005904" "ENSGACG00000005912"
## [11572] "ENSGACG00000025258" "ENSGACG00000004448" "ENSGACG00000004494"
## [11575] "ENSGACG00000004496" "ENSGACG00000004509" "ENSGACG00000004514"
## [11578] "ENSGACG00000033291" "ENSGACG00000035453" "ENSGACG00000004517"
## [11581] "ENSGACG00000004590" "ENSGACG00000004607" "ENSGACG00000004622"
## [11584] "ENSGACG00000004630" "ENSGACG00000004633" "ENSGACG00000004645"
## [11587] "ENSGACG00000004647" "ENSGACG00000004651" "ENSGACG00000004705"
## [11590] "ENSGACG00000004738" "ENSGACG00000004752" "ENSGACG00000004768"
## [11593] "ENSGACG00000004771" "ENSGACG00000004791" "ENSGACG00000004850"
## [11596] "ENSGACG00000001558" "ENSGACG00000037635" "ENSGACG00000001555"
## [11599] "ENSGACG00000033356" "ENSGACG00000001553" "ENSGACG00000001544"
## [11602] "ENSGACG00000001540" "ENSGACG00000026710" "ENSGACG00000001522"
## [11605] "ENSGACG00000001520" "ENSGACG00000001516" "ENSGACG00000001514"
## [11608] "ENSGACG00000029368" "ENSGACG00000001513" "ENSGACG00000023007"
## [11611] "ENSGACG00000001510" "ENSGACG00000001505" "ENSGACG00000027979"
## [11614] "ENSGACG00000028623" "ENSGACG00000001498" "ENSGACG00000025178"
## [11617] "ENSGACG00000001497" "ENSGACG00000001495" "ENSGACG00000001490"
## [11620] "ENSGACG00000001486" "ENSGACG00000001483" "ENSGACG00000001480"
## [11623] "ENSGACG00000001478" "ENSGACG00000027561" "ENSGACG00000025948"
## [11626] "ENSGACG00000001476" "ENSGACG00000023911" "ENSGACG00000001473"
## [11629] "ENSGACG00000000801" "ENSGACG00000001466" "ENSGACG00000001437"
## [11632] "ENSGACG00000001398" "ENSGACG00000035928" "ENSGACG00000001382"
## [11635] "ENSGACG00000001373" "ENSGACG00000024616" "ENSGACG00000001365"
## [11638] "ENSGACG00000024885" "ENSGACG00000001352" "ENSGACG00000001346"
## [11641] "ENSGACG00000001336" "ENSGACG00000001327" "ENSGACG00000001317"
## [11644] "ENSGACG00000036382" "ENSGACG00000037481" "ENSGACG00000005926"
## [11647] "ENSGACG00000005941" "ENSGACG00000005944" "ENSGACG00000005966"
## [11650] "ENSGACG00000006083" "ENSGACG00000006038" "ENSGACG00000006050"
## [11653] "ENSGACG00000006056" "ENSGACG00000006075" "ENSGACG00000006088"
## [11656] "ENSGACG00000006093" "ENSGACG00000006111" "ENSGACG00000006115"
## [11659] "ENSGACG00000006124" "ENSGACG00000027633" "ENSGACG00000006153"
## [11662] "ENSGACG00000006187" "ENSGACG00000006223" "ENSGACG00000035478"
## [11665] "ENSGACG00000006233" "ENSGACG00000006249" "ENSGACG00000006306"
## [11668] "ENSGACG00000026403" "ENSGACG00000006354" "ENSGACG00000025675"
## [11671] "ENSGACG00000006383" "ENSGACG00000006399" "ENSGACG00000006403"
## [11674] "ENSGACG00000006410" "ENSGACG00000035992" "ENSGACG00000006442"
## [11677] "ENSGACG00000006458" "ENSGACG00000027762" "ENSGACG00000006466"
## [11680] "ENSGACG00000006474" "ENSGACG00000029174" "ENSGACG00000006484"
## [11683] "ENSGACG00000006532" "ENSGACG00000006601" "ENSGACG00000006633"
## [11686] "ENSGACG00000006645" "ENSGACG00000024320" "ENSGACG00000024288"
## [11689] "ENSGACG00000038144" "ENSGACG00000006681" "ENSGACG00000006732"
## [11692] "ENSGACG00000006741" "ENSGACG00000001300" "ENSGACG00000024152"
## [11695] "ENSGACG00000000486" "ENSGACG00000000472" "ENSGACG00000023127"
## [11698] "ENSGACG00000001294" "ENSGACG00000033258" "ENSGACG00000001286"
## [11701] "ENSGACG00000001280" "ENSGACG00000001278" "ENSGACG00000001276"
## [11704] "ENSGACG00000001275" "ENSGACG00000001254" "ENSGACG00000035064"
## [11707] "ENSGACG00000026736" "ENSGACG00000023351" "ENSGACG00000001246"
## [11710] "ENSGACG00000001237" "ENSGACG00000001229" "ENSGACG00000001222"
## [11713] "ENSGACG00000001219" "ENSGACG00000001217" "ENSGACG00000001211"
## [11716] "ENSGACG00000033713" "ENSGACG00000024227" "ENSGACG00000001206"
## [11719] "ENSGACG00000001197" "ENSGACG00000000467" "ENSGACG00000000461"
## [11722] "ENSGACG00000001195" "ENSGACG00000000456" "ENSGACG00000035063"
## [11725] "ENSGACG00000001188" "ENSGACG00000001183" "ENSGACG00000001179"
## [11728] "ENSGACG00000001176" "ENSGACG00000000451" "ENSGACG00000000557"
## [11731] "ENSGACG00000005153" "ENSGACG00000037066" "ENSGACG00000029576"
## [11734] "ENSGACG00000027413" "ENSGACG00000000523" "ENSGACG00000037087"
## [11737] "ENSGACG00000011748" "ENSGACG00000030894" "ENSGACG00000031406"
## [11740] "ENSGACG00000032153" "ENSGACG00000005236" "ENSGACG00000005247"
## [11743] "ENSGACG00000005276" "ENSGACG00000005335" "ENSGACG00000005350"
## [11746] "ENSGACG00000005373" "ENSGACG00000005376" "ENSGACG00000035399"
## [11749] "ENSGACG00000005448" "ENSGACG00000005459" "ENSGACG00000005473"
## [11752] "ENSGACG00000005480" "ENSGACG00000011770" "ENSGACG00000011779"
## [11755] "ENSGACG00000011788" "ENSGACG00000011797" "ENSGACG00000034931"
## [11758] "ENSGACG00000011823" "ENSGACG00000034378" "ENSGACG00000011839"
## [11761] "ENSGACG00000011845" "ENSGACG00000011892" "ENSGACG00000011903"
## [11764] "ENSGACG00000011898" "ENSGACG00000011915" "ENSGACG00000011919"
## [11767] "ENSGACG00000011921" "ENSGACG00000033251" "ENSGACG00000005505"
## [11770] "ENSGACG00000027720" "ENSGACG00000005512" "ENSGACG00000011964"
## [11773] "ENSGACG00000023031" "ENSGACG00000037183" "ENSGACG00000011972"
## [11776] "ENSGACG00000005548" "ENSGACG00000011989" "ENSGACG00000025914"
## [11779] "ENSGACG00000005643" "ENSGACG00000005571" "ENSGACG00000005608"
## [11782] "ENSGACG00000033736" "ENSGACG00000034313" "ENSGACG00000005678"
## [11785] "ENSGACG00000005724" "ENSGACG00000005758" "ENSGACG00000011998"
## [11788] "ENSGACG00000012007" "ENSGACG00000005765" "ENSGACG00000009983"
## [11791] "ENSGACG00000005769" "ENSGACG00000005817" "ENSGACG00000005847"
## [11794] "ENSGACG00000001164" "ENSGACG00000001156" "ENSGACG00000026975"
## [11797] "ENSGACG00000001151" "ENSGACG00000001143" "ENSGACG00000001140"
## [11800] "ENSGACG00000001112" "ENSGACG00000023439" "ENSGACG00000001094"
## [11803] "ENSGACG00000027207" "ENSGACG00000001090" "ENSGACG00000001085"
## [11806] "ENSGACG00000031615" "ENSGACG00000001083" "ENSGACG00000001082"
## [11809] "ENSGACG00000001070" "ENSGACG00000001065" "ENSGACG00000001063"
## [11812] "ENSGACG00000024779" "ENSGACG00000031879" "ENSGACG00000009988"
## [11815] "ENSGACG00000001058" "ENSGACG00000001059" "ENSGACG00000036739"
## [11818] "ENSGACG00000001056" "ENSGACG00000001052" "ENSGACG00000032707"
## [11821] "ENSGACG00000010012" "ENSGACG00000001035" "ENSGACG00000000385"
## [11824] "ENSGACG00000035310" "ENSGACG00000001029" "ENSGACG00000000374"
## [11827] "ENSGACG00000001028" "ENSGACG00000001025" "ENSGACG00000025488"
## [11830] "ENSGACG00000001016" "ENSGACG00000001013" "ENSGACG00000027194"
## [11833] "ENSGACG00000001008" "ENSGACG00000001004" "ENSGACG00000000990"
## [11836] "ENSGACG00000000986" "ENSGACG00000000980" "ENSGACG00000000963"
## [11839] "ENSGACG00000000945" "ENSGACG00000027260" "ENSGACG00000000936"
## [11842] "ENSGACG00000000930" "ENSGACG00000000924" "ENSGACG00000000376"
## [11845] "ENSGACG00000000371" "ENSGACG00000000552" "ENSGACG00000000550"
## [11848] "ENSGACG00000000545" "ENSGACG00000037704" "ENSGACG00000000544"
## [11851] "ENSGACG00000000539" "ENSGACG00000029275" "ENSGACG00000000367"
## [11854] "ENSGACG00000023107" "ENSGACG00000000364" "ENSGACG00000000357"
## [11857] "ENSGACG00000000353" "ENSGACG00000035813" "ENSGACG00000000349"
## [11860] "ENSGACG00000000347" "ENSGACG00000000340" "ENSGACG00000000914"
## [11863] "ENSGACG00000022907" "ENSGACG00000034656" "ENSGACG00000024304"
## [11866] "ENSGACG00000000906" "ENSGACG00000000898" "ENSGACG00000000897"
## [11869] "ENSGACG00000000884" "ENSGACG00000000332" "ENSGACG00000000331"
## [11872] "ENSGACG00000028114" "ENSGACG00000000327" "ENSGACG00000000319"
## [11875] "ENSGACG00000028980" "ENSGACG00000000872" "ENSGACG00000000868"
## [11878] "ENSGACG00000000858" "ENSGACG00000000316" "ENSGACG00000000845"
## [11881] "ENSGACG00000000313" "ENSGACG00000000312" "ENSGACG00000000309"
## [11884] "ENSGACG00000030273" "ENSGACG00000000839" "ENSGACG00000000302"
## [11887] "ENSGACG00000028123" "ENSGACG00000000836" "ENSGACG00000007585"
## [11890] "ENSGACG00000000833" "ENSGACG00000034817" "ENSGACG00000000831"
## [11893] "ENSGACG00000000830" "ENSGACG00000034824" "ENSGACG00000031277"
## [11896] "ENSGACG00000035279" "ENSGACG00000000443" "ENSGACG00000000439"
## [11899] "ENSGACG00000032493" "ENSGACG00000023845" "ENSGACG00000000829"
## [11902] "ENSGACG00000000425" "ENSGACG00000000824" "ENSGACG00000025483"
## [11905] "ENSGACG00000000823" "ENSGACG00000000412" "ENSGACG00000025277"
## [11908] "ENSGACG00000000815" "ENSGACG00000000813" "ENSGACG00000000807"
## [11911] "ENSGACG00000000006" "ENSGACG00000026959" "ENSGACG00000035488"
## [11914] "ENSGACG00000033778" "ENSGACG00000007609" "ENSGACG00000003025"
## [11917] "ENSGACG00000000009" "ENSGACG00000028027" "ENSGACG00000028652"
## [11920] "ENSGACG00000000011" "ENSGACG00000000012" "ENSGACG00000000013"
## [11923] "ENSGACG00000026510" "ENSGACG00000000014" "ENSGACG00000035241"
## [11926] "ENSGACG00000007667" "ENSGACG00000000355" "ENSGACG00000035675"
## [11929] "ENSGACG00000000015" "ENSGACG00000000016" "ENSGACG00000007676"
## [11932] "ENSGACG00000000017" "ENSGACG00000000019" "ENSGACG00000000022"
## [11935] "ENSGACG00000000399" "ENSGACG00000033149" "ENSGACG00000027495"
## [11938] "ENSGACG00000000397" "ENSGACG00000027201" "ENSGACG00000030606"
## [11941] "ENSGACG00000028387" "ENSGACG00000007720" "ENSGACG00000000024"
## [11944] "ENSGACG00000000025" "ENSGACG00000037311" "ENSGACG00000000027"
## [11947] "ENSGACG00000000030" "ENSGACG00000025074" "ENSGACG00000000035"
## [11950] "ENSGACG00000000037" "ENSGACG00000034042" "ENSGACG00000029421"
## [11953] "ENSGACG00000007778" "ENSGACG00000007848" "ENSGACG00000007862"
## [11956] "ENSGACG00000000039" "ENSGACG00000007882" "ENSGACG00000033690"
## [11959] "ENSGACG00000007904" "ENSGACG00000026620" "ENSGACG00000007927"
## [11962] "ENSGACG00000007946" "ENSGACG00000025817" "ENSGACG00000007965"
## [11965] "ENSGACG00000007975" "ENSGACG00000007995" "ENSGACG00000008010"
## [11968] "ENSGACG00000008033" "ENSGACG00000008085" "ENSGACG00000008104"
## [11971] "ENSGACG00000024614" "ENSGACG00000023666" "ENSGACG00000008115"
## [11974] "ENSGACG00000000528" "ENSGACG00000032509" "ENSGACG00000011390"
## [11977] "ENSGACG00000008135" "ENSGACG00000000524" "ENSGACG00000034944"
## [11980] "ENSGACG00000000653" "ENSGACG00000000644" "ENSGACG00000008420"
## [11983] "ENSGACG00000032479" "ENSGACG00000008456" "ENSGACG00000008496"
## [11986] "ENSGACG00000008580" "ENSGACG00000008585" "ENSGACG00000034815"
## [11989] "ENSGACG00000008628" "ENSGACG00000033289" "ENSGACG00000008668"
## [11992] "ENSGACG00000008674" "ENSGACG00000008683" "ENSGACG00000008695"
## [11995] "ENSGACG00000008713" "ENSGACG00000008748" "ENSGACG00000008752"
## [11998] "ENSGACG00000008760" "ENSGACG00000008765" "ENSGACG00000008771"
## [12001] "ENSGACG00000008815" "ENSGACG00000008841" "ENSGACG00000008854"
## [12004] "ENSGACG00000027251" "ENSGACG00000025157" "ENSGACG00000037669"
## [12007] "ENSGACG00000008894" "ENSGACG00000008899" "ENSGACG00000035236"
## [12010] "ENSGACG00000008906" "ENSGACG00000003062" "ENSGACG00000007022"
## [12013] "ENSGACG00000023775" "ENSGACG00000035029" "ENSGACG00000000033"
## [12016] "ENSGACG00000008926" "ENSGACG00000036585" "ENSGACG00000008930"
## [12019] "ENSGACG00000035812" "ENSGACG00000023430" "ENSGACG00000033100"
## [12022] "ENSGACG00000027305" "ENSGACG00000008945" "ENSGACG00000026034"
## [12025] "ENSGACG00000008960" "ENSGACG00000024116" "ENSGACG00000030790"
## [12028] "ENSGACG00000033528" "ENSGACG00000009018" "ENSGACG00000027986"
## [12031] "ENSGACG00000025852" "ENSGACG00000037690" "ENSGACG00000030571"
## [12034] "ENSGACG00000034008" "ENSGACG00000027200" "ENSGACG00000035332"
## [12037] "ENSGACG00000023924" "ENSGACG00000026170" "ENSGACG00000024828"
## [12040] "ENSGACG00000030978" "ENSGACG00000036028" "ENSGACG00000032193"
## [12043] "ENSGACG00000034122" "ENSGACG00000037126" "ENSGACG00000006676"
## [12046] "ENSGACG00000024331" "ENSGACG00000035611" "ENSGACG00000037863"
## [12049] "ENSGACG00000023805" "ENSGACG00000030848" "ENSGACG00000035607"
## [12052] "ENSGACG00000034245" "ENSGACG00000026322" "ENSGACG00000035649"
## [12055] "ENSGACG00000023615" "ENSGACG00000009021" "ENSGACG00000024015"
## [12058] "ENSGACG00000009034" "ENSGACG00000037668" "ENSGACG00000033811"
## [12061] "ENSGACG00000031878" "ENSGACG00000023403" "ENSGACG00000023913"
## [12064] "ENSGACG00000028818" "ENSGACG00000023717" "ENSGACG00000028848"
## [12067] "ENSGACG00000000572" "ENSGACG00000027841" "ENSGACG00000027318"
## [12070] "ENSGACG00000000568" "ENSGACG00000031051" "ENSGACG00000022538"
## [12073] "ENSGACG00000007023" "ENSGACG00000007015" "ENSGACG00000034022"
## [12076] "ENSGACG00000000008" "ENSGACG00000010036" "ENSGACG00000004912"
## [12079] "ENSGACG00000003641" "ENSGACG00000003834" "ENSGACG00000000585"
## [12082] "ENSGACG00000008215" "ENSGACG00000029118" "ENSGACG00000028507"
## [12085] "ENSGACG00000001585" "ENSGACG00000008234" "ENSGACG00000008237"
## [12088] "ENSGACG00000008268" "ENSGACG00000025629" "ENSGACG00000028654"
## [12091] "ENSGACG00000008312" "ENSGACG00000006069" "ENSGACG00000029503"
## [12094] "ENSGACG00000006396" "ENSGACG00000025229" "ENSGACG00000001042"
## [12097] "ENSGACG00000008392" "ENSGACG00000000920" "ENSGACG00000000522"
## [12100] "ENSGACG00000000516" "ENSGACG00000000508" "ENSGACG00000000663"
## [12103] "ENSGACG00000000661" "ENSGACG00000000502" "ENSGACG00000000498"
## [12106] "ENSGACG00000000493" "ENSGACG00000011651" "ENSGACG00000011394"
## [12109] "ENSGACG00000011415" "ENSGACG00000029775" "ENSGACG00000032630"
## [12112] "ENSGACG00000011455" "ENSGACG00000011458" "ENSGACG00000011658"
## [12115] "ENSGACG00000025149" "ENSGACG00000011468" "ENSGACG00000011482"
## [12118] "ENSGACG00000011497" "ENSGACG00000011518" "ENSGACG00000011527"
## [12121] "ENSGACG00000011542" "ENSGACG00000033124" "ENSGACG00000011531"
## [12124] "ENSGACG00000011562" "ENSGACG00000011567" "ENSGACG00000011572"
## [12127] "ENSGACG00000011590" "ENSGACG00000011613" "ENSGACG00000011618"
## [12130] "ENSGACG00000000643" "ENSGACG00000000641" "ENSGACG00000000638"
## [12133] "ENSGACG00000000633" "ENSGACG00000025076" "ENSGACG00000000618"
## [12136] "ENSGACG00000034376" "ENSGACG00000000617" "ENSGACG00000032464"
## [12139] "ENSGACG00000000614" "ENSGACG00000000612" "ENSGACG00000023963"
## [12142] "ENSGACG00000000608" "ENSGACG00000000605" "ENSGACG00000000604"
## [12145] "ENSGACG00000000601" "ENSGACG00000011629" "ENSGACG00000034916"
## [12148] "ENSGACG00000011636" "ENSGACG00000009348" "ENSGACG00000009360"
## [12151] "ENSGACG00000009376" "ENSGACG00000009380" "ENSGACG00000009387"
## [12154] "ENSGACG00000009393" "ENSGACG00000024317" "ENSGACG00000009400"
## [12157] "ENSGACG00000009434" "ENSGACG00000009440" "ENSGACG00000009496"
## [12160] "ENSGACG00000034673" "ENSGACG00000009511" "ENSGACG00000000746"
## [12163] "ENSGACG00000000743" "ENSGACG00000024328" "ENSGACG00000000739"
## [12166] "ENSGACG00000000729" "ENSGACG00000000723" "ENSGACG00000009541"
## [12169] "ENSGACG00000009563" "ENSGACG00000034466" "ENSGACG00000009583"
## [12172] "ENSGACG00000009608" "ENSGACG00000000719" "ENSGACG00000000714"
## [12175] "ENSGACG00000000713" "ENSGACG00000000711" "ENSGACG00000000710"
## [12178] "ENSGACG00000027094" "ENSGACG00000029690" "ENSGACG00000000707"
## [12181] "ENSGACG00000029405" "ENSGACG00000024430" "ENSGACG00000000703"
## [12184] "ENSGACG00000026615" "ENSGACG00000000697" "ENSGACG00000000696"
## [12187] "ENSGACG00000000694" "ENSGACG00000000692" "ENSGACG00000000687"
## [12190] "ENSGACG00000000685" "ENSGACG00000000681" "ENSGACG00000000680"
## [12193] "ENSGACG00000000679" "ENSGACG00000000671" "ENSGACG00000000667"
## [12196] "ENSGACG00000000666" "ENSGACG00000000665" "ENSGACG00000000664"
## [12199] "ENSGACG00000009049" "ENSGACG00000009111" "ENSGACG00000009129"
## [12202] "ENSGACG00000009148" "ENSGACG00000009155" "ENSGACG00000009164"
## [12205] "ENSGACG00000009172" "ENSGACG00000027826" "ENSGACG00000037738"
## [12208] "ENSGACG00000009188" "ENSGACG00000009218" "ENSGACG00000009237"
## [12211] "ENSGACG00000009250" "ENSGACG00000009306" "ENSGACG00000009317"
## [12214] "ENSGACG00000009341" "ENSGACG00000000599" "ENSGACG00000012037"
## [12217] "ENSGACG00000012044" "ENSGACG00000012070" "ENSGACG00000009623"
## [12220] "ENSGACG00000009647" "ENSGACG00000009669" "ENSGACG00000009672"
## [12223] "ENSGACG00000009682" "ENSGACG00000025180" "ENSGACG00000009696"
## [12226] "ENSGACG00000035079" "ENSGACG00000009703" "ENSGACG00000009707"
## [12229] "ENSGACG00000009779" "ENSGACG00000009789" "ENSGACG00000009822"
## [12232] "ENSGACG00000009846" "ENSGACG00000009851" "ENSGACG00000033166"
## [12235] "ENSGACG00000009882" "ENSGACG00000009945" "ENSGACG00000032376"
## [12238] "ENSGACG00000009971" "ENSGACG00000009973" "ENSGACG00000009979"
## [12241] "ENSGACG00000008148" "ENSGACG00000033443" "ENSGACG00000024380"
## [12244] "ENSGACG00000008176" "ENSGACG00000025342" "ENSGACG00000008190"
## [12247] "ENSGACG00000008196" "ENSGACG00000008199" "ENSGACG00000010046"
## [12250] "ENSGACG00000010055" "ENSGACG00000031561" "ENSGACG00000010085"
## [12253] "ENSGACG00000010090" "ENSGACG00000026315" "ENSGACG00000010137"
## [12256] "ENSGACG00000033397" "ENSGACG00000036176" "ENSGACG00000010157"
## [12259] "ENSGACG00000010175" "ENSGACG00000030981" "ENSGACG00000010183"
## [12262] "ENSGACG00000026998" "ENSGACG00000010199" "ENSGACG00000010218"
## [12265] "ENSGACG00000010222" "ENSGACG00000010229" "ENSGACG00000010287"
## [12268] "ENSGACG00000010320" "ENSGACG00000010355" "ENSGACG00000027129"
## [12271] "ENSGACG00000010382" "ENSGACG00000010391" "ENSGACG00000010427"
## [12274] "ENSGACG00000010452" "ENSGACG00000031826" "ENSGACG00000010458"
## [12277] "ENSGACG00000010469" "ENSGACG00000010478" "ENSGACG00000010512"
## [12280] "ENSGACG00000034063" "ENSGACG00000010539" "ENSGACG00000010551"
## [12283] "ENSGACG00000010570" "ENSGACG00000010600" "ENSGACG00000010605"
## [12286] "ENSGACG00000010643" "ENSGACG00000010721" "ENSGACG00000028064"
## [12289] "ENSGACG00000010733" "ENSGACG00000010763" "ENSGACG00000010767"
## [12292] "ENSGACG00000010803" "ENSGACG00000010815" "ENSGACG00000031783"
## [12295] "ENSGACG00000010825" "ENSGACG00000010830" "ENSGACG00000010836"
## [12298] "ENSGACG00000010864" "ENSGACG00000010880" "ENSGACG00000036970"
## [12301] "ENSGACG00000010913" "ENSGACG00000031459" "ENSGACG00000035396"
## [12304] "ENSGACG00000010920" "ENSGACG00000010934" "ENSGACG00000010931"
## [12307] "ENSGACG00000038086" "ENSGACG00000010942" "ENSGACG00000010949"
## [12310] "ENSGACG00000010953" "ENSGACG00000010962" "ENSGACG00000010970"
## [12313] "ENSGACG00000029445" "ENSGACG00000010974" "ENSGACG00000010989"
## [12316] "ENSGACG00000010998" "ENSGACG00000011029" "ENSGACG00000036397"
## [12319] "ENSGACG00000032337" "ENSGACG00000011074" "ENSGACG00000011090"
## [12322] "ENSGACG00000025335" "ENSGACG00000011105" "ENSGACG00000011107"
## [12325] "ENSGACG00000011143" "ENSGACG00000011150" "ENSGACG00000011170"
## [12328] "ENSGACG00000032092" "ENSGACG00000011220" "ENSGACG00000036848"
## [12331] "ENSGACG00000011241" "ENSGACG00000011245" "ENSGACG00000011248"
## [12334] "ENSGACG00000011250" "ENSGACG00000027987" "ENSGACG00000011254"
## [12337] "ENSGACG00000035870" "ENSGACG00000011258" "ENSGACG00000025365"
## [12340] "ENSGACG00000011321" "ENSGACG00000011328" "ENSGACG00000011335"
## [12343] "ENSGACG00000011347" "ENSGACG00000011357" "ENSGACG00000011367"
## [12346] "ENSGACG00000011371" "ENSGACG00000011376" "ENSGACG00000011387"
## [12349] "ENSGACG00000011673" "ENSGACG00000011676" "ENSGACG00000011718"
## [12352] "ENSGACG00000011729" "ENSGACG00000030685" "ENSGACG00000011735"
## [12355] "ENSGACG00000026635" "ENSGACG00000023411" "ENSGACG00000030886"
## [12358] "ENSGACG00000031487" "ENSGACG00000034926" "ENSGACG00000031974"
## [12361] "ENSGACG00000035438" "ENSGACG00000031543" "ENSGACG00000023553"
## [12364] "ENSGACG00000034235" "ENSGACG00000027759" "ENSGACG00000036106"
## [12367] "ENSGACG00000022999" "ENSGACG00000028455" "ENSGACG00000032492"
## [12370] "ENSGACG00000029215" "ENSGACG00000024279" "ENSGACG00000027364"
## [12373] "ENSGACG00000035351" "ENSGACG00000024971" "ENSGACG00000031263"
## [12376] "ENSGACG00000029852" "ENSGACG00000027168" "ENSGACG00000029805"
## [12379] "ENSGACG00000026639" "ENSGACG00000023874" "ENSGACG00000025050"
## [12382] "ENSGACG00000026633" "ENSGACG00000033067" "ENSGACG00000027719"
## [12385] "ENSGACG00000036620" "ENSGACG00000035248" "ENSGACG00000035593"
## [12388] "ENSGACG00000036013" "ENSGACG00000028248" "ENSGACG00000023998"
## [12391] "ENSGACG00000036152" "ENSGACG00000037740" "ENSGACG00000023073"
## [12394] "ENSGACG00000032260" "ENSGACG00000030409" "ENSGACG00000029245"
## [12397] "ENSGACG00000027234" "ENSGACG00000031894" "ENSGACG00000035652"
## [12400] "ENSGACG00000028063" "ENSGACG00000030012" "ENSGACG00000035795"
## [12403] "ENSGACG00000032742" "ENSGACG00000000383" "ENSGACG00000025352"
## [12406] "ENSGACG00000008794" "ENSGACG00000000584" "ENSGACG00000000512"
## [12409] "ENSGACG00000011426" "ENSGACG00000011554" "ENSGACG00000000610"
## [12412] "ENSGACG00000009596" "ENSGACG00000009200" "ENSGACG00000026752"
## [12415] "ENSGACG00000027383" "ENSGACG00000002387" "ENSGACG00000002393"
## [12418] "ENSGACG00000002401" "ENSGACG00000002409" "ENSGACG00000002501"
## [12421] "ENSGACG00000030739" "ENSGACG00000002511" "ENSGACG00000002514"
## [12424] "ENSGACG00000038109" "ENSGACG00000032668" "ENSGACG00000002540"
## [12427] "ENSGACG00000002543" "ENSGACG00000002557" "ENSGACG00000002567"
## [12430] "ENSGACG00000002570" "ENSGACG00000002592" "ENSGACG00000002609"
## [12433] "ENSGACG00000002124" "ENSGACG00000002130" "ENSGACG00000002141"
## [12436] "ENSGACG00000002144" "ENSGACG00000002630" "ENSGACG00000026353"
## [12439] "ENSGACG00000004600" "ENSGACG00000004616" "ENSGACG00000004638"
## [12442] "ENSGACG00000004687" "ENSGACG00000004721" "ENSGACG00000004749"
## [12445] "ENSGACG00000004757" "ENSGACG00000004760" "ENSGACG00000003790"
## [12448] "ENSGACG00000003798" "ENSGACG00000003812" "ENSGACG00000003818"
## [12451] "ENSGACG00000003820" "ENSGACG00000030319" "ENSGACG00000003830"
## [12454] "ENSGACG00000003842" "ENSGACG00000003851" "ENSGACG00000003863"
## [12457] "ENSGACG00000003867" "ENSGACG00000003896" "ENSGACG00000003900"
## [12460] "ENSGACG00000003946" "ENSGACG00000034967" "ENSGACG00000003967"
## [12463] "ENSGACG00000003978" "ENSGACG00000003992" "ENSGACG00000004014"
## [12466] "ENSGACG00000004025" "ENSGACG00000004031" "ENSGACG00000004046"
## [12469] "ENSGACG00000034130" "ENSGACG00000004056" "ENSGACG00000004057"
## [12472] "ENSGACG00000004062" "ENSGACG00000004069" "ENSGACG00000025877"
## [12475] "ENSGACG00000029212" "ENSGACG00000002320" "ENSGACG00000002328"
## [12478] "ENSGACG00000002350" "ENSGACG00000002353" "ENSGACG00000024953"
## [12481] "ENSGACG00000002376" "ENSGACG00000002110" "ENSGACG00000002111"
## [12484] "ENSGACG00000002113" "ENSGACG00000002122" "ENSGACG00000008466"
## [12487] "ENSGACG00000004470" "ENSGACG00000004477" "ENSGACG00000008458"
## [12490] "ENSGACG00000023977" "ENSGACG00000004551" "ENSGACG00000006621"
## [12493] "ENSGACG00000006627" "ENSGACG00000027866" "ENSGACG00000035872"
## [12496] "ENSGACG00000032396" "ENSGACG00000026833" "ENSGACG00000002153"
## [12499] "ENSGACG00000006634" "ENSGACG00000006651" "ENSGACG00000006664"
## [12502] "ENSGACG00000006674" "ENSGACG00000006684" "ENSGACG00000006678"
## [12505] "ENSGACG00000002161" "ENSGACG00000006709" "ENSGACG00000002168"
## [12508] "ENSGACG00000002172" "ENSGACG00000026602" "ENSGACG00000006724"
## [12511] "ENSGACG00000006731" "ENSGACG00000002186" "ENSGACG00000033360"
## [12514] "ENSGACG00000006747" "ENSGACG00000025638" "ENSGACG00000032740"
## [12517] "ENSGACG00000002196" "ENSGACG00000025724" "ENSGACG00000034415"
## [12520] "ENSGACG00000035021" "ENSGACG00000002200" "ENSGACG00000023677"
## [12523] "ENSGACG00000006760" "ENSGACG00000007330" "ENSGACG00000002216"
## [12526] "ENSGACG00000007424" "ENSGACG00000028188" "ENSGACG00000002220"
## [12529] "ENSGACG00000002222" "ENSGACG00000002227" "ENSGACG00000032807"
## [12532] "ENSGACG00000002228" "ENSGACG00000025819" "ENSGACG00000034587"
## [12535] "ENSGACG00000007436" "ENSGACG00000034570" "ENSGACG00000027533"
## [12538] "ENSGACG00000031925" "ENSGACG00000007473" "ENSGACG00000002266"
## [12541] "ENSGACG00000007498" "ENSGACG00000031392" "ENSGACG00000006770"
## [12544] "ENSGACG00000002269" "ENSGACG00000002287" "ENSGACG00000002298"
## [12547] "ENSGACG00000002303" "ENSGACG00000006779" "ENSGACG00000028564"
## [12550] "ENSGACG00000023864" "ENSGACG00000002314" "ENSGACG00000031334"
## [12553] "ENSGACG00000001602" "ENSGACG00000023070" "ENSGACG00000032903"
## [12556] "ENSGACG00000006805" "ENSGACG00000004584" "ENSGACG00000006813"
## [12559] "ENSGACG00000001904" "ENSGACG00000006823" "ENSGACG00000003716"
## [12562] "ENSGACG00000006826" "ENSGACG00000003745" "ENSGACG00000037084"
## [12565] "ENSGACG00000006855" "ENSGACG00000004082" "ENSGACG00000006880"
## [12568] "ENSGACG00000028337" "ENSGACG00000035853" "ENSGACG00000030102"
## [12571] "ENSGACG00000028548" "ENSGACG00000004101" "ENSGACG00000006911"
## [12574] "ENSGACG00000029304" "ENSGACG00000004126" "ENSGACG00000027097"
## [12577] "ENSGACG00000004162" "ENSGACG00000004166" "ENSGACG00000004189"
## [12580] "ENSGACG00000027258" "ENSGACG00000004222" "ENSGACG00000004237"
## [12583] "ENSGACG00000034354" "ENSGACG00000028812" "ENSGACG00000006982"
## [12586] "ENSGACG00000007013" "ENSGACG00000007044" "ENSGACG00000007052"
## [12589] "ENSGACG00000007062" "ENSGACG00000007119" "ENSGACG00000007146"
## [12592] "ENSGACG00000007282" "ENSGACG00000007289" "ENSGACG00000007299"
## [12595] "ENSGACG00000028139" "ENSGACG00000007323" "ENSGACG00000024361"
## [12598] "ENSGACG00000004228" "ENSGACG00000007556" "ENSGACG00000007578"
## [12601] "ENSGACG00000004265" "ENSGACG00000008479" "ENSGACG00000033997"
## [12604] "ENSGACG00000038035" "ENSGACG00000007624" "ENSGACG00000007660"
## [12607] "ENSGACG00000007337" "ENSGACG00000007370" "ENSGACG00000007387"
## [12610] "ENSGACG00000008415" "ENSGACG00000004299" "ENSGACG00000036229"
## [12613] "ENSGACG00000004321" "ENSGACG00000004337" "ENSGACG00000004341"
## [12616] "ENSGACG00000004378" "ENSGACG00000004394" "ENSGACG00000004410"
## [12619] "ENSGACG00000024555" "ENSGACG00000004421" "ENSGACG00000004444"
## [12622] "ENSGACG00000001926" "ENSGACG00000001943" "ENSGACG00000036399"
## [12625] "ENSGACG00000001955" "ENSGACG00000027429" "ENSGACG00000036202"
## [12628] "ENSGACG00000001985" "ENSGACG00000028606" "ENSGACG00000001999"
## [12631] "ENSGACG00000037713" "ENSGACG00000002015" "ENSGACG00000002025"
## [12634] "ENSGACG00000002035" "ENSGACG00000002051" "ENSGACG00000002059"
## [12637] "ENSGACG00000035457" "ENSGACG00000034659" "ENSGACG00000002071"
## [12640] "ENSGACG00000035743" "ENSGACG00000002091" "ENSGACG00000002107"
## [12643] "ENSGACG00000001417" "ENSGACG00000024538" "ENSGACG00000001438"
## [12646] "ENSGACG00000001446" "ENSGACG00000008399" "ENSGACG00000001450"
## [12649] "ENSGACG00000001454" "ENSGACG00000035452" "ENSGACG00000001456"
## [12652] "ENSGACG00000001457" "ENSGACG00000001463" "ENSGACG00000008393"
## [12655] "ENSGACG00000001488" "ENSGACG00000001491" "ENSGACG00000001508"
## [12658] "ENSGACG00000028622" "ENSGACG00000001512" "ENSGACG00000001519"
## [12661] "ENSGACG00000008352" "ENSGACG00000023565" "ENSGACG00000031026"
## [12664] "ENSGACG00000031897" "ENSGACG00000024653" "ENSGACG00000027522"
## [12667] "ENSGACG00000023124" "ENSGACG00000008295" "ENSGACG00000008289"
## [12670] "ENSGACG00000027747" "ENSGACG00000008266" "ENSGACG00000008251"
## [12673] "ENSGACG00000001429" "ENSGACG00000001425" "ENSGACG00000001423"
## [12676] "ENSGACG00000001420" "ENSGACG00000001415" "ENSGACG00000001411"
## [12679] "ENSGACG00000001406" "ENSGACG00000037371" "ENSGACG00000001402"
## [12682] "ENSGACG00000001397" "ENSGACG00000028061" "ENSGACG00000031876"
## [12685] "ENSGACG00000001380" "ENSGACG00000001370" "ENSGACG00000006618"
## [12688] "ENSGACG00000023721" "ENSGACG00000001363" "ENSGACG00000001360"
## [12691] "ENSGACG00000001351" "ENSGACG00000001343" "ENSGACG00000001340"
## [12694] "ENSGACG00000007889" "ENSGACG00000032290" "ENSGACG00000001530"
## [12697] "ENSGACG00000007909" "ENSGACG00000001531" "ENSGACG00000001532"
## [12700] "ENSGACG00000007934" "ENSGACG00000001536" "ENSGACG00000025313"
## [12703] "ENSGACG00000007941" "ENSGACG00000001535" "ENSGACG00000007959"
## [12706] "ENSGACG00000001538" "ENSGACG00000028289" "ENSGACG00000026278"
## [12709] "ENSGACG00000034387" "ENSGACG00000007977" "ENSGACG00000029999"
## [12712] "ENSGACG00000007989" "ENSGACG00000001542" "ENSGACG00000001545"
## [12715] "ENSGACG00000008017" "ENSGACG00000030796" "ENSGACG00000008038"
## [12718] "ENSGACG00000008088" "ENSGACG00000001547" "ENSGACG00000008096"
## [12721] "ENSGACG00000008120" "ENSGACG00000008127" "ENSGACG00000008143"
## [12724] "ENSGACG00000008147" "ENSGACG00000008155" "ENSGACG00000008168"
## [12727] "ENSGACG00000001548" "ENSGACG00000001549" "ENSGACG00000008189"
## [12730] "ENSGACG00000001550" "ENSGACG00000008203" "ENSGACG00000008206"
## [12733] "ENSGACG00000008218" "ENSGACG00000008227" "ENSGACG00000008239"
## [12736] "ENSGACG00000008246" "ENSGACG00000009353" "ENSGACG00000026697"
## [12739] "ENSGACG00000009339" "ENSGACG00000009326" "ENSGACG00000009303"
## [12742] "ENSGACG00000001551" "ENSGACG00000001556" "ENSGACG00000001559"
## [12745] "ENSGACG00000001563" "ENSGACG00000001577" "ENSGACG00000001590"
## [12748] "ENSGACG00000001595" "ENSGACG00000002634" "ENSGACG00000009289"
## [12751] "ENSGACG00000032459" "ENSGACG00000002642" "ENSGACG00000002648"
## [12754] "ENSGACG00000009271" "ENSGACG00000037672" "ENSGACG00000009246"
## [12757] "ENSGACG00000002657" "ENSGACG00000009239" "ENSGACG00000009203"
## [12760] "ENSGACG00000009197" "ENSGACG00000009167" "ENSGACG00000027080"
## [12763] "ENSGACG00000032272" "ENSGACG00000009181" "ENSGACG00000032933"
## [12766] "ENSGACG00000009132" "ENSGACG00000009122" "ENSGACG00000009102"
## [12769] "ENSGACG00000009092" "ENSGACG00000028214" "ENSGACG00000009074"
## [12772] "ENSGACG00000009047" "ENSGACG00000004774" "ENSGACG00000004790"
## [12775] "ENSGACG00000004808" "ENSGACG00000004831" "ENSGACG00000004843"
## [12778] "ENSGACG00000037270" "ENSGACG00000002673" "ENSGACG00000004887"
## [12781] "ENSGACG00000002684" "ENSGACG00000022972" "ENSGACG00000004931"
## [12784] "ENSGACG00000036782" "ENSGACG00000005061" "ENSGACG00000005080"
## [12787] "ENSGACG00000005096" "ENSGACG00000005107" "ENSGACG00000005135"
## [12790] "ENSGACG00000005149" "ENSGACG00000005157" "ENSGACG00000005163"
## [12793] "ENSGACG00000005168" "ENSGACG00000005182" "ENSGACG00000005207"
## [12796] "ENSGACG00000005219" "ENSGACG00000005232" "ENSGACG00000005264"
## [12799] "ENSGACG00000030113" "ENSGACG00000005307" "ENSGACG00000005325"
## [12802] "ENSGACG00000005352" "ENSGACG00000024401" "ENSGACG00000005372"
## [12805] "ENSGACG00000037207" "ENSGACG00000005385" "ENSGACG00000005428"
## [12808] "ENSGACG00000005450" "ENSGACG00000002705" "ENSGACG00000002715"
## [12811] "ENSGACG00000002724" "ENSGACG00000002733" "ENSGACG00000002735"
## [12814] "ENSGACG00000002751" "ENSGACG00000002755" "ENSGACG00000027236"
## [12817] "ENSGACG00000027781" "ENSGACG00000030649" "ENSGACG00000002764"
## [12820] "ENSGACG00000002775" "ENSGACG00000002780" "ENSGACG00000002784"
## [12823] "ENSGACG00000026512" "ENSGACG00000002789" "ENSGACG00000002796"
## [12826] "ENSGACG00000005466" "ENSGACG00000002798" "ENSGACG00000005477"
## [12829] "ENSGACG00000005484" "ENSGACG00000032908" "ENSGACG00000002800"
## [12832] "ENSGACG00000002817" "ENSGACG00000024281" "ENSGACG00000002821"
## [12835] "ENSGACG00000024710" "ENSGACG00000030918" "ENSGACG00000005501"
## [12838] "ENSGACG00000031888" "ENSGACG00000005518" "ENSGACG00000002834"
## [12841] "ENSGACG00000002858" "ENSGACG00000030318" "ENSGACG00000002869"
## [12844] "ENSGACG00000002882" "ENSGACG00000005606" "ENSGACG00000005612"
## [12847] "ENSGACG00000030175" "ENSGACG00000002891" "ENSGACG00000005665"
## [12850] "ENSGACG00000031120" "ENSGACG00000005671" "ENSGACG00000002904"
## [12853] "ENSGACG00000002912" "ENSGACG00000029643" "ENSGACG00000027451"
## [12856] "ENSGACG00000002921" "ENSGACG00000026778" "ENSGACG00000005734"
## [12859] "ENSGACG00000002929" "ENSGACG00000002932" "ENSGACG00000028452"
## [12862] "ENSGACG00000002945" "ENSGACG00000002951" "ENSGACG00000002959"
## [12865] "ENSGACG00000031959" "ENSGACG00000002970" "ENSGACG00000037233"
## [12868] "ENSGACG00000023244" "ENSGACG00000003005" "ENSGACG00000003022"
## [12871] "ENSGACG00000003031" "ENSGACG00000003038" "ENSGACG00000003063"
## [12874] "ENSGACG00000023467" "ENSGACG00000003073" "ENSGACG00000003079"
## [12877] "ENSGACG00000003108" "ENSGACG00000003116" "ENSGACG00000003127"
## [12880] "ENSGACG00000003146" "ENSGACG00000023646" "ENSGACG00000003150"
## [12883] "ENSGACG00000003166" "ENSGACG00000003173" "ENSGACG00000003184"
## [12886] "ENSGACG00000003237" "ENSGACG00000003240" "ENSGACG00000003250"
## [12889] "ENSGACG00000003264" "ENSGACG00000003276" "ENSGACG00000034497"
## [12892] "ENSGACG00000003291" "ENSGACG00000003296" "ENSGACG00000023599"
## [12895] "ENSGACG00000003318" "ENSGACG00000003344" "ENSGACG00000005749"
## [12898] "ENSGACG00000003355" "ENSGACG00000005757" "ENSGACG00000003384"
## [12901] "ENSGACG00000003391" "ENSGACG00000003400" "ENSGACG00000003424"
## [12904] "ENSGACG00000007503" "ENSGACG00000008500" "ENSGACG00000003430"
## [12907] "ENSGACG00000003454" "ENSGACG00000003459" "ENSGACG00000003468"
## [12910] "ENSGACG00000003480" "ENSGACG00000031311" "ENSGACG00000003514"
## [12913] "ENSGACG00000027835" "ENSGACG00000003532" "ENSGACG00000028183"
## [12916] "ENSGACG00000032673" "ENSGACG00000009026" "ENSGACG00000009017"
## [12919] "ENSGACG00000009010" "ENSGACG00000033822" "ENSGACG00000008860"
## [12922] "ENSGACG00000027777" "ENSGACG00000008839" "ENSGACG00000003548"
## [12925] "ENSGACG00000003564" "ENSGACG00000008817" "ENSGACG00000003570"
## [12928] "ENSGACG00000024351" "ENSGACG00000033785" "ENSGACG00000003586"
## [12931] "ENSGACG00000003588" "ENSGACG00000003593" "ENSGACG00000003598"
## [12934] "ENSGACG00000003619" "ENSGACG00000024176" "ENSGACG00000003624"
## [12937] "ENSGACG00000003629" "ENSGACG00000003633" "ENSGACG00000003643"
## [12940] "ENSGACG00000003648" "ENSGACG00000036802" "ENSGACG00000037191"
## [12943] "ENSGACG00000026485" "ENSGACG00000035176" "ENSGACG00000035465"
## [12946] "ENSGACG00000033400" "ENSGACG00000003672" "ENSGACG00000003683"
## [12949] "ENSGACG00000028072" "ENSGACG00000003690" "ENSGACG00000003694"
## [12952] "ENSGACG00000003701" "ENSGACG00000003713" "ENSGACG00000001609"
## [12955] "ENSGACG00000008803" "ENSGACG00000034411" "ENSGACG00000031908"
## [12958] "ENSGACG00000001638" "ENSGACG00000001659" "ENSGACG00000001661"
## [12961] "ENSGACG00000001664" "ENSGACG00000001668" "ENSGACG00000001676"
## [12964] "ENSGACG00000037993" "ENSGACG00000001684" "ENSGACG00000034939"
## [12967] "ENSGACG00000034139" "ENSGACG00000001702" "ENSGACG00000001719"
## [12970] "ENSGACG00000029471" "ENSGACG00000001735" "ENSGACG00000032550"
## [12973] "ENSGACG00000026674" "ENSGACG00000026328" "ENSGACG00000031539"
## [12976] "ENSGACG00000001746" "ENSGACG00000008651" "ENSGACG00000026008"
## [12979] "ENSGACG00000022963" "ENSGACG00000025246" "ENSGACG00000008632"
## [12982] "ENSGACG00000008611" "ENSGACG00000008601" "ENSGACG00000008579"
## [12985] "ENSGACG00000008565" "ENSGACG00000027944" "ENSGACG00000008521"
## [12988] "ENSGACG00000004939" "ENSGACG00000004971" "ENSGACG00000004990"
## [12991] "ENSGACG00000022979" "ENSGACG00000005019" "ENSGACG00000005028"
## [12994] "ENSGACG00000005048" "ENSGACG00000005053" "ENSGACG00000004770"
## [12997] "ENSGACG00000009037" "ENSGACG00000007714" "ENSGACG00000025834"
## [13000] "ENSGACG00000007753" "ENSGACG00000007758" "ENSGACG00000007774"
## [13003] "ENSGACG00000007798" "ENSGACG00000007813" "ENSGACG00000007818"
## [13006] "ENSGACG00000007845" "ENSGACG00000030328" "ENSGACG00000005780"
## [13009] "ENSGACG00000005787" "ENSGACG00000005800" "ENSGACG00000033732"
## [13012] "ENSGACG00000005810" "ENSGACG00000005819" "ENSGACG00000005824"
## [13015] "ENSGACG00000036368" "ENSGACG00000005831" "ENSGACG00000032440"
## [13018] "ENSGACG00000005860" "ENSGACG00000026877" "ENSGACG00000005886"
## [13021] "ENSGACG00000005914" "ENSGACG00000005931" "ENSGACG00000005937"
## [13024] "ENSGACG00000033725" "ENSGACG00000005954" "ENSGACG00000001792"
## [13027] "ENSGACG00000024472" "ENSGACG00000025727" "ENSGACG00000033971"
## [13030] "ENSGACG00000025202" "ENSGACG00000005972" "ENSGACG00000001830"
## [13033] "ENSGACG00000005978" "ENSGACG00000005986" "ENSGACG00000005991"
## [13036] "ENSGACG00000001846" "ENSGACG00000001873" "ENSGACG00000035729"
## [13039] "ENSGACG00000006057" "ENSGACG00000001878" "ENSGACG00000029726"
## [13042] "ENSGACG00000006066" "ENSGACG00000006073" "ENSGACG00000006082"
## [13045] "ENSGACG00000006089" "ENSGACG00000006130" "ENSGACG00000024310"
## [13048] "ENSGACG00000006173" "ENSGACG00000037488" "ENSGACG00000001885"
## [13051] "ENSGACG00000001886" "ENSGACG00000025556" "ENSGACG00000001892"
## [13054] "ENSGACG00000001896" "ENSGACG00000028140" "ENSGACG00000001901"
## [13057] "ENSGACG00000025272" "ENSGACG00000023169" "ENSGACG00000027108"
## [13060] "ENSGACG00000032762" "ENSGACG00000026856" "ENSGACG00000027106"
## [13063] "ENSGACG00000032441" "ENSGACG00000002011" "ENSGACG00000033329"
## [13066] "ENSGACG00000037067" "ENSGACG00000034115" "ENSGACG00000036339"
## [13069] "ENSGACG00000036764" "ENSGACG00000026039" "ENSGACG00000033343"
## [13072] "ENSGACG00000024272" "ENSGACG00000024605" "ENSGACG00000024461"
## [13075] "ENSGACG00000034452" "ENSGACG00000032814" "ENSGACG00000034318"
## [13078] "ENSGACG00000037881" "ENSGACG00000025842" "ENSGACG00000031206"
## [13081] "ENSGACG00000031713" "ENSGACG00000030228" "ENSGACG00000022632"
## [13084] "ENSGACG00000033405" "ENSGACG00000002367" "ENSGACG00000001422"
## [13087] "ENSGACG00000001424" "ENSGACG00000001427" "ENSGACG00000028807"
## [13090] "ENSGACG00000036612" "ENSGACG00000031681" "ENSGACG00000001640"
## [13093] "ENSGACG00000006196" "ENSGACG00000006203" "ENSGACG00000006226"
## [13096] "ENSGACG00000006235" "ENSGACG00000006240" "ENSGACG00000006275"
## [13099] "ENSGACG00000035654" "ENSGACG00000037760" "ENSGACG00000006286"
## [13102] "ENSGACG00000006301" "ENSGACG00000037917" "ENSGACG00000006314"
## [13105] "ENSGACG00000032521" "ENSGACG00000006325" "ENSGACG00000006333"
## [13108] "ENSGACG00000006360" "ENSGACG00000006373" "ENSGACG00000006376"
## [13111] "ENSGACG00000006392" "ENSGACG00000038008" "ENSGACG00000031175"
## [13114] "ENSGACG00000006433" "ENSGACG00000006465" "ENSGACG00000006476"
## [13117] "ENSGACG00000030223" "ENSGACG00000006493" "ENSGACG00000029104"
## [13120] "ENSGACG00000006497" "ENSGACG00000006528" "ENSGACG00000006539"
## [13123] "ENSGACG00000030523" "ENSGACG00000034481" "ENSGACG00000024467"
## [13126] "ENSGACG00000006616" "ENSGACG00000033012" "ENSGACG00000001334"
## [13129] "ENSGACG00000030800" "ENSGACG00000001333" "ENSGACG00000001328"
## [13132] "ENSGACG00000001204" "ENSGACG00000001218" "ENSGACG00000001225"
## [13135] "ENSGACG00000033763" "ENSGACG00000001233" "ENSGACG00000001235"
## [13138] "ENSGACG00000001238" "ENSGACG00000000317" "ENSGACG00000000310"
## [13141] "ENSGACG00000034005" "ENSGACG00000000306" "ENSGACG00000000299"
## [13144] "ENSGACG00000000294" "ENSGACG00000036319" "ENSGACG00000000289"
## [13147] "ENSGACG00000033497" "ENSGACG00000000271" "ENSGACG00000037158"
## [13150] "ENSGACG00000000269" "ENSGACG00000000265" "ENSGACG00000000264"
## [13153] "ENSGACG00000000260" "ENSGACG00000000257" "ENSGACG00000000255"
## [13156] "ENSGACG00000000250" "ENSGACG00000000243" "ENSGACG00000000235"
## [13159] "ENSGACG00000026449" "ENSGACG00000022918" "ENSGACG00000000828"
## [13162] "ENSGACG00000028815" "ENSGACG00000000825" "ENSGACG00000000820"
## [13165] "ENSGACG00000000792" "ENSGACG00000026698" "ENSGACG00000026084"
## [13168] "ENSGACG00000025913" "ENSGACG00000034685" "ENSGACG00000030308"
## [13171] "ENSGACG00000029257" "ENSGACG00000028052" "ENSGACG00000029328"
## [13174] "ENSGACG00000025118" "ENSGACG00000023038" "ENSGACG00000025398"
## [13177] "ENSGACG00000033256" "ENSGACG00000030857" "ENSGACG00000033160"
## [13180] "ENSGACG00000028090" "ENSGACG00000036946" "ENSGACG00000023850"
## [13183] "ENSGACG00000035653" "ENSGACG00000026747" "ENSGACG00000023085"
## [13186] "ENSGACG00000026755" "ENSGACG00000023265" "ENSGACG00000035867"
## [13189] "ENSGACG00000024770" "ENSGACG00000034774" "ENSGACG00000027019"
## [13192] "ENSGACG00000028617" "ENSGACG00000036611" "ENSGACG00000033733"
## [13195] "ENSGACG00000030608" "ENSGACG00000030709" "ENSGACG00000037507"
## [13198] "ENSGACG00000029541" "ENSGACG00000034923" "ENSGACG00000024371"
## [13201] "ENSGACG00000026263" "ENSGACG00000030806" "ENSGACG00000023614"
## [13204] "ENSGACG00000037805" "ENSGACG00000028583" "ENSGACG00000029587"
## [13207] "ENSGACG00000006831" "ENSGACG00000008277" "ENSGACG00000031243"
## [13210] "ENSGACG00000005191" "ENSGACG00000005001" "ENSGACG00000028741"
## [13213] "ENSGACG00000006329" "ENSGACG00000006424" "ENSGACG00000028614"
## [13216] "ENSGACG00000029285" "ENSGACG00000034418" "ENSGACG00000013453"
## [13219] "ENSGACG00000037520" "ENSGACG00000013451" "ENSGACG00000027230"
## [13222] "ENSGACG00000013439" "ENSGACG00000013432" "ENSGACG00000013422"
## [13225] "ENSGACG00000013419" "ENSGACG00000027498" "ENSGACG00000004149"
## [13228] "ENSGACG00000026461" "ENSGACG00000004154" "ENSGACG00000004165"
## [13231] "ENSGACG00000004171" "ENSGACG00000004174" "ENSGACG00000004188"
## [13234] "ENSGACG00000004210" "ENSGACG00000002507" "ENSGACG00000002489"
## [13237] "ENSGACG00000029456" "ENSGACG00000002483" "ENSGACG00000002479"
## [13240] "ENSGACG00000002470" "ENSGACG00000002458" "ENSGACG00000037103"
## [13243] "ENSGACG00000002453" "ENSGACG00000029411" "ENSGACG00000002439"
## [13246] "ENSGACG00000002424" "ENSGACG00000024803" "ENSGACG00000036649"
## [13249] "ENSGACG00000002358" "ENSGACG00000002412" "ENSGACG00000032818"
## [13252] "ENSGACG00000002408" "ENSGACG00000002342" "ENSGACG00000005573"
## [13255] "ENSGACG00000029660" "ENSGACG00000005596" "ENSGACG00000031152"
## [13258] "ENSGACG00000005686" "ENSGACG00000005759" "ENSGACG00000005771"
## [13261] "ENSGACG00000005813" "ENSGACG00000005849" "ENSGACG00000030702"
## [13264] "ENSGACG00000005865" "ENSGACG00000005885" "ENSGACG00000005893"
## [13267] "ENSGACG00000005921" "ENSGACG00000005946" "ENSGACG00000005964"
## [13270] "ENSGACG00000026330" "ENSGACG00000002325" "ENSGACG00000033970"
## [13273] "ENSGACG00000002316" "ENSGACG00000005832" "ENSGACG00000003000"
## [13276] "ENSGACG00000002988" "ENSGACG00000002981" "ENSGACG00000002962"
## [13279] "ENSGACG00000002953" "ENSGACG00000027754" "ENSGACG00000002949"
## [13282] "ENSGACG00000002944" "ENSGACG00000002936" "ENSGACG00000002931"
## [13285] "ENSGACG00000002905" "ENSGACG00000002880" "ENSGACG00000029078"
## [13288] "ENSGACG00000002407" "ENSGACG00000002396" "ENSGACG00000003443"
## [13291] "ENSGACG00000003439" "ENSGACG00000003423" "ENSGACG00000003415"
## [13294] "ENSGACG00000026670" "ENSGACG00000003406" "ENSGACG00000026358"
## [13297] "ENSGACG00000003378" "ENSGACG00000003365" "ENSGACG00000026705"
## [13300] "ENSGACG00000003346" "ENSGACG00000003332" "ENSGACG00000003308"
## [13303] "ENSGACG00000003302" "ENSGACG00000003269" "ENSGACG00000005326"
## [13306] "ENSGACG00000004737" "ENSGACG00000004750" "ENSGACG00000025976"
## [13309] "ENSGACG00000036766" "ENSGACG00000003253" "ENSGACG00000003243"
## [13312] "ENSGACG00000003233" "ENSGACG00000003216" "ENSGACG00000023971"
## [13315] "ENSGACG00000003208" "ENSGACG00000003199" "ENSGACG00000003191"
## [13318] "ENSGACG00000003174" "ENSGACG00000003163" "ENSGACG00000003133"
## [13321] "ENSGACG00000003087" "ENSGACG00000003077" "ENSGACG00000003044"
## [13324] "ENSGACG00000034597" "ENSGACG00000032819" "ENSGACG00000003032"
## [13327] "ENSGACG00000003026" "ENSGACG00000026304" "ENSGACG00000003012"
## [13330] "ENSGACG00000003007" "ENSGACG00000004833" "ENSGACG00000034416"
## [13333] "ENSGACG00000004988" "ENSGACG00000029443" "ENSGACG00000005042"
## [13336] "ENSGACG00000005068" "ENSGACG00000037736" "ENSGACG00000005079"
## [13339] "ENSGACG00000005094" "ENSGACG00000005108" "ENSGACG00000024893"
## [13342] "ENSGACG00000023988" "ENSGACG00000005152" "ENSGACG00000005196"
## [13345] "ENSGACG00000005211" "ENSGACG00000026581" "ENSGACG00000005227"
## [13348] "ENSGACG00000002870" "ENSGACG00000002854" "ENSGACG00000004223"
## [13351] "ENSGACG00000004254" "ENSGACG00000004273" "ENSGACG00000004285"
## [13354] "ENSGACG00000004298" "ENSGACG00000004320" "ENSGACG00000004340"
## [13357] "ENSGACG00000004345" "ENSGACG00000004370" "ENSGACG00000027472"
## [13360] "ENSGACG00000004367" "ENSGACG00000004399" "ENSGACG00000004414"
## [13363] "ENSGACG00000004443" "ENSGACG00000004455" "ENSGACG00000004481"
## [13366] "ENSGACG00000036792" "ENSGACG00000004493" "ENSGACG00000005268"
## [13369] "ENSGACG00000005336" "ENSGACG00000037154" "ENSGACG00000005354"
## [13372] "ENSGACG00000005370" "ENSGACG00000005387" "ENSGACG00000005392"
## [13375] "ENSGACG00000005432" "ENSGACG00000005486" "ENSGACG00000005491"
## [13378] "ENSGACG00000005527" "ENSGACG00000034257" "ENSGACG00000035520"
## [13381] "ENSGACG00000004513" "ENSGACG00000004530" "ENSGACG00000004532"
## [13384] "ENSGACG00000034224" "ENSGACG00000004572" "ENSGACG00000036351"
## [13387] "ENSGACG00000035339" "ENSGACG00000024632" "ENSGACG00000004659"
## [13390] "ENSGACG00000004673" "ENSGACG00000006014" "ENSGACG00000006039"
## [13393] "ENSGACG00000006067" "ENSGACG00000006071" "ENSGACG00000006097"
## [13396] "ENSGACG00000006105" "ENSGACG00000006151" "ENSGACG00000037379"
## [13399] "ENSGACG00000033416" "ENSGACG00000006193" "ENSGACG00000037934"
## [13402] "ENSGACG00000006250" "ENSGACG00000006266" "ENSGACG00000006272"
## [13405] "ENSGACG00000006299" "ENSGACG00000006310" "ENSGACG00000030688"
## [13408] "ENSGACG00000006327" "ENSGACG00000006356" "ENSGACG00000006367"
## [13411] "ENSGACG00000006384" "ENSGACG00000006416" "ENSGACG00000006449"
## [13414] "ENSGACG00000006468" "ENSGACG00000006489" "ENSGACG00000006522"
## [13417] "ENSGACG00000006537" "ENSGACG00000006549" "ENSGACG00000029084"
## [13420] "ENSGACG00000036376" "ENSGACG00000006560" "ENSGACG00000006579"
## [13423] "ENSGACG00000006647" "ENSGACG00000006671" "ENSGACG00000006698"
## [13426] "ENSGACG00000006694" "ENSGACG00000006721" "ENSGACG00000006746"
## [13429] "ENSGACG00000006750" "ENSGACG00000030481" "ENSGACG00000025782"
## [13432] "ENSGACG00000028388" "ENSGACG00000027882" "ENSGACG00000006785"
## [13435] "ENSGACG00000006893" "ENSGACG00000023027" "ENSGACG00000006921"
## [13438] "ENSGACG00000006938" "ENSGACG00000006956" "ENSGACG00000006962"
## [13441] "ENSGACG00000006972" "ENSGACG00000007028" "ENSGACG00000023052"
## [13444] "ENSGACG00000033505" "ENSGACG00000002843" "ENSGACG00000002838"
## [13447] "ENSGACG00000002828" "ENSGACG00000029868" "ENSGACG00000002820"
## [13450] "ENSGACG00000002816" "ENSGACG00000002807" "ENSGACG00000002804"
## [13453] "ENSGACG00000002791" "ENSGACG00000002787" "ENSGACG00000002779"
## [13456] "ENSGACG00000032849" "ENSGACG00000030788" "ENSGACG00000002743"
## [13459] "ENSGACG00000002736" "ENSGACG00000002732" "ENSGACG00000032014"
## [13462] "ENSGACG00000002718" "ENSGACG00000023674" "ENSGACG00000002656"
## [13465] "ENSGACG00000002632" "ENSGACG00000002649" "ENSGACG00000036129"
## [13468] "ENSGACG00000002618" "ENSGACG00000002612" "ENSGACG00000002605"
## [13471] "ENSGACG00000028725" "ENSGACG00000002595" "ENSGACG00000027518"
## [13474] "ENSGACG00000034725" "ENSGACG00000031694" "ENSGACG00000030307"
## [13477] "ENSGACG00000003951" "ENSGACG00000024358" "ENSGACG00000003961"
## [13480] "ENSGACG00000003966" "ENSGACG00000003974" "ENSGACG00000003979"
## [13483] "ENSGACG00000003982" "ENSGACG00000004016" "ENSGACG00000002297"
## [13486] "ENSGACG00000026787" "ENSGACG00000002277" "ENSGACG00000002267"
## [13489] "ENSGACG00000002246" "ENSGACG00000002201" "ENSGACG00000004033"
## [13492] "ENSGACG00000023737" "ENSGACG00000004080" "ENSGACG00000004104"
## [13495] "ENSGACG00000004118" "ENSGACG00000025824" "ENSGACG00000025207"
## [13498] "ENSGACG00000004137" "ENSGACG00000002189" "ENSGACG00000002181"
## [13501] "ENSGACG00000035951" "ENSGACG00000031633" "ENSGACG00000002170"
## [13504] "ENSGACG00000002162" "ENSGACG00000025194" "ENSGACG00000014640"
## [13507] "ENSGACG00000014633" "ENSGACG00000014625" "ENSGACG00000024736"
## [13510] "ENSGACG00000014607" "ENSGACG00000014597" "ENSGACG00000035430"
## [13513] "ENSGACG00000003922" "ENSGACG00000033173" "ENSGACG00000003897"
## [13516] "ENSGACG00000003864" "ENSGACG00000003862" "ENSGACG00000034702"
## [13519] "ENSGACG00000003828" "ENSGACG00000023293" "ENSGACG00000003785"
## [13522] "ENSGACG00000025452" "ENSGACG00000003778" "ENSGACG00000003758"
## [13525] "ENSGACG00000003725" "ENSGACG00000003705" "ENSGACG00000026629"
## [13528] "ENSGACG00000003677" "ENSGACG00000028936" "ENSGACG00000032344"
## [13531] "ENSGACG00000003516" "ENSGACG00000003611" "ENSGACG00000029224"
## [13534] "ENSGACG00000003595" "ENSGACG00000003475" "ENSGACG00000003502"
## [13537] "ENSGACG00000037455" "ENSGACG00000003465" "ENSGACG00000003447"
## [13540] "ENSGACG00000007032" "ENSGACG00000007039" "ENSGACG00000007110"
## [13543] "ENSGACG00000007139" "ENSGACG00000032548" "ENSGACG00000007150"
## [13546] "ENSGACG00000007175" "ENSGACG00000007189" "ENSGACG00000007213"
## [13549] "ENSGACG00000024675" "ENSGACG00000007257" "ENSGACG00000007285"
## [13552] "ENSGACG00000035643" "ENSGACG00000007296" "ENSGACG00000007308"
## [13555] "ENSGACG00000007317" "ENSGACG00000002550" "ENSGACG00000002577"
## [13558] "ENSGACG00000036248" "ENSGACG00000002530" "ENSGACG00000002518"
## [13561] "ENSGACG00000030551" "ENSGACG00000035926" "ENSGACG00000023325"
## [13564] "ENSGACG00000027596" "ENSGACG00000024283" "ENSGACG00000036984"
## [13567] "ENSGACG00000030244" "ENSGACG00000029871" "ENSGACG00000033017"
## [13570] "ENSGACG00000028921" "ENSGACG00000023994" "ENSGACG00000035877"
## [13573] "ENSGACG00000033543" "ENSGACG00000028033" "ENSGACG00000022944"
## [13576] "ENSGACG00000033840" "ENSGACG00000036051" "ENSGACG00000037813"
## [13579] "ENSGACG00000033611" "ENSGACG00000027834" "ENSGACG00000032595"
## [13582] "ENSGACG00000031871" "ENSGACG00000035003" "ENSGACG00000025910"
## [13585] "ENSGACG00000030305" "ENSGACG00000035874" "ENSGACG00000032568"
## [13588] "ENSGACG00000029630" "ENSGACG00000037882" "ENSGACG00000030107"
## [13591] "ENSGACG00000005066" "ENSGACG00000037897" "ENSGACG00000034730"
## [13594] "ENSGACG00000024293" "ENSGACG00000025251" "ENSGACG00000031634"
## [13597] "ENSGACG00000030928" "ENSGACG00000032862" "ENSGACG00000026012"
## [13600] "ENSGACG00000038024" "ENSGACG00000032578" "ENSGACG00000029600"
## [13603] "ENSGACG00000033172" "ENSGACG00000023696" "ENSGACG00000031996"
## [13606] "ENSGACG00000023651" "ENSGACG00000031586" "ENSGACG00000031372"
## [13609] "ENSGACG00000027096" "ENSGACG00000035560" "ENSGACG00000005304"
## [13612] "ENSGACG00000003055" "ENSGACG00000030985" "ENSGACG00000004355"
## [13615] "ENSGACG00000004582" "ENSGACG00000006526" "ENSGACG00000006850"
## [13618] "ENSGACG00000036001" "ENSGACG00000003638" "ENSGACG00000009178"
## [13621] "ENSGACG00000009195" "ENSGACG00000009236" "ENSGACG00000009282"
## [13624] "ENSGACG00000009043" "ENSGACG00000009101" "ENSGACG00000030427"
## [13627] "ENSGACG00000009115" "ENSGACG00000009135" "ENSGACG00000009143"
## [13630] "ENSGACG00000024489" "ENSGACG00000009168" "ENSGACG00000027227"
## [13633] "ENSGACG00000033863" "ENSGACG00000011843" "ENSGACG00000011901"
## [13636] "ENSGACG00000029592" "ENSGACG00000011909" "ENSGACG00000011926"
## [13639] "ENSGACG00000030220" "ENSGACG00000034380" "ENSGACG00000011939"
## [13642] "ENSGACG00000011946" "ENSGACG00000011953" "ENSGACG00000011956"
## [13645] "ENSGACG00000011648" "ENSGACG00000011659" "ENSGACG00000011687"
## [13648] "ENSGACG00000026097" "ENSGACG00000011701" "ENSGACG00000011726"
## [13651] "ENSGACG00000011733" "ENSGACG00000022951" "ENSGACG00000011822"
## [13654] "ENSGACG00000011832" "ENSGACG00000011835" "ENSGACG00000037815"
## [13657] "ENSGACG00000011988" "ENSGACG00000012052" "ENSGACG00000012068"
## [13660] "ENSGACG00000012072" "ENSGACG00000012086" "ENSGACG00000012095"
## [13663] "ENSGACG00000012107" "ENSGACG00000024622" "ENSGACG00000029590"
## [13666] "ENSGACG00000028561" "ENSGACG00000031213" "ENSGACG00000025645"
## [13669] "ENSGACG00000012144" "ENSGACG00000034419" "ENSGACG00000012160"
## [13672] "ENSGACG00000012166" "ENSGACG00000012168" "ENSGACG00000029621"
## [13675] "ENSGACG00000012217" "ENSGACG00000012222" "ENSGACG00000032576"
## [13678] "ENSGACG00000011400" "ENSGACG00000011409" "ENSGACG00000011414"
## [13681] "ENSGACG00000011422" "ENSGACG00000037478" "ENSGACG00000011467"
## [13684] "ENSGACG00000027004" "ENSGACG00000012242" "ENSGACG00000037119"
## [13687] "ENSGACG00000012246" "ENSGACG00000012257" "ENSGACG00000012262"
## [13690] "ENSGACG00000012266" "ENSGACG00000012317" "ENSGACG00000012324"
## [13693] "ENSGACG00000012325" "ENSGACG00000025160" "ENSGACG00000032232"
## [13696] "ENSGACG00000012392" "ENSGACG00000012405" "ENSGACG00000012371"
## [13699] "ENSGACG00000035983" "ENSGACG00000001281" "ENSGACG00000001298"
## [13702] "ENSGACG00000001299" "ENSGACG00000001302" "ENSGACG00000001304"
## [13705] "ENSGACG00000001316" "ENSGACG00000001323" "ENSGACG00000001338"
## [13708] "ENSGACG00000036018" "ENSGACG00000029475" "ENSGACG00000001354"
## [13711] "ENSGACG00000001371" "ENSGACG00000010723" "ENSGACG00000010738"
## [13714] "ENSGACG00000010764" "ENSGACG00000010786" "ENSGACG00000023596"
## [13717] "ENSGACG00000010790" "ENSGACG00000010794" "ENSGACG00000010801"
## [13720] "ENSGACG00000010808" "ENSGACG00000010814" "ENSGACG00000010826"
## [13723] "ENSGACG00000010868" "ENSGACG00000010879" "ENSGACG00000031854"
## [13726] "ENSGACG00000026976" "ENSGACG00000009385" "ENSGACG00000007523"
## [13729] "ENSGACG00000007529" "ENSGACG00000007534" "ENSGACG00000009390"
## [13732] "ENSGACG00000009419" "ENSGACG00000009438" "ENSGACG00000009461"
## [13735] "ENSGACG00000029942" "ENSGACG00000009468" "ENSGACG00000009518"
## [13738] "ENSGACG00000023702" "ENSGACG00000009543" "ENSGACG00000009548"
## [13741] "ENSGACG00000009577" "ENSGACG00000009582" "ENSGACG00000009597"
## [13744] "ENSGACG00000009611" "ENSGACG00000009617" "ENSGACG00000009631"
## [13747] "ENSGACG00000030916" "ENSGACG00000009665" "ENSGACG00000009677"
## [13750] "ENSGACG00000028503" "ENSGACG00000009700" "ENSGACG00000009708"
## [13753] "ENSGACG00000009284" "ENSGACG00000029426" "ENSGACG00000009318"
## [13756] "ENSGACG00000023402" "ENSGACG00000034929" "ENSGACG00000009340"
## [13759] "ENSGACG00000009370" "ENSGACG00000011080" "ENSGACG00000010907"
## [13762] "ENSGACG00000010922" "ENSGACG00000010928" "ENSGACG00000028965"
## [13765] "ENSGACG00000010952" "ENSGACG00000026297" "ENSGACG00000010981"
## [13768] "ENSGACG00000010992" "ENSGACG00000037400" "ENSGACG00000011003"
## [13771] "ENSGACG00000011020" "ENSGACG00000011051" "ENSGACG00000011058"
## [13774] "ENSGACG00000024527" "ENSGACG00000029890" "ENSGACG00000011076"
## [13777] "ENSGACG00000011528" "ENSGACG00000034894" "ENSGACG00000011543"
## [13780] "ENSGACG00000024042" "ENSGACG00000011571" "ENSGACG00000032710"
## [13783] "ENSGACG00000011586" "ENSGACG00000025254" "ENSGACG00000036686"
## [13786] "ENSGACG00000011623" "ENSGACG00000011628" "ENSGACG00000011638"
## [13789] "ENSGACG00000030312" "ENSGACG00000011092" "ENSGACG00000011099"
## [13792] "ENSGACG00000024870" "ENSGACG00000011191" "ENSGACG00000024915"
## [13795] "ENSGACG00000011110" "ENSGACG00000025362" "ENSGACG00000027152"
## [13798] "ENSGACG00000011151" "ENSGACG00000011156" "ENSGACG00000034101"
## [13801] "ENSGACG00000038134" "ENSGACG00000011225" "ENSGACG00000033259"
## [13804] "ENSGACG00000011288" "ENSGACG00000011307" "ENSGACG00000011319"
## [13807] "ENSGACG00000011333" "ENSGACG00000026036" "ENSGACG00000010243"
## [13810] "ENSGACG00000037840" "ENSGACG00000010249" "ENSGACG00000010255"
## [13813] "ENSGACG00000010267" "ENSGACG00000023686" "ENSGACG00000037565"
## [13816] "ENSGACG00000010300" "ENSGACG00000010307" "ENSGACG00000010343"
## [13819] "ENSGACG00000010350" "ENSGACG00000010363" "ENSGACG00000010365"
## [13822] "ENSGACG00000037609" "ENSGACG00000010377" "ENSGACG00000026854"
## [13825] "ENSGACG00000010407" "ENSGACG00000026966" "ENSGACG00000010424"
## [13828] "ENSGACG00000029877" "ENSGACG00000010439" "ENSGACG00000010492"
## [13831] "ENSGACG00000010522" "ENSGACG00000010549" "ENSGACG00000010558"
## [13834] "ENSGACG00000026577" "ENSGACG00000010587" "ENSGACG00000035412"
## [13837] "ENSGACG00000010606" "ENSGACG00000010618" "ENSGACG00000034836"
## [13840] "ENSGACG00000010635" "ENSGACG00000010638" "ENSGACG00000010653"
## [13843] "ENSGACG00000010679" "ENSGACG00000027060" "ENSGACG00000011492"
## [13846] "ENSGACG00000011512" "ENSGACG00000032407" "ENSGACG00000007331"
## [13849] "ENSGACG00000007363" "ENSGACG00000007366" "ENSGACG00000007381"
## [13852] "ENSGACG00000007400" "ENSGACG00000007419" "ENSGACG00000030953"
## [13855] "ENSGACG00000007432" "ENSGACG00000007441" "ENSGACG00000007469"
## [13858] "ENSGACG00000032590" "ENSGACG00000036804" "ENSGACG00000007489"
## [13861] "ENSGACG00000007506" "ENSGACG00000007574" "ENSGACG00000007586"
## [13864] "ENSGACG00000007620" "ENSGACG00000007647" "ENSGACG00000031937"
## [13867] "ENSGACG00000007665" "ENSGACG00000037878" "ENSGACG00000036215"
## [13870] "ENSGACG00000007690" "ENSGACG00000007692" "ENSGACG00000007703"
## [13873] "ENSGACG00000007709" "ENSGACG00000007740" "ENSGACG00000007749"
## [13876] "ENSGACG00000007762" "ENSGACG00000007784" "ENSGACG00000031136"
## [13879] "ENSGACG00000007799" "ENSGACG00000007851" "ENSGACG00000007857"
## [13882] "ENSGACG00000007905" "ENSGACG00000033910" "ENSGACG00000031847"
## [13885] "ENSGACG00000033551" "ENSGACG00000025542" "ENSGACG00000008002"
## [13888] "ENSGACG00000008020" "ENSGACG00000023033" "ENSGACG00000008031"
## [13891] "ENSGACG00000008034" "ENSGACG00000008040" "ENSGACG00000008050"
## [13894] "ENSGACG00000008091" "ENSGACG00000008095" "ENSGACG00000008101"
## [13897] "ENSGACG00000036615" "ENSGACG00000008124" "ENSGACG00000008164"
## [13900] "ENSGACG00000008169" "ENSGACG00000008241" "ENSGACG00000008292"
## [13903] "ENSGACG00000008331" "ENSGACG00000008348" "ENSGACG00000008386"
## [13906] "ENSGACG00000008391" "ENSGACG00000008425" "ENSGACG00000008460"
## [13909] "ENSGACG00000008478" "ENSGACG00000008487" "ENSGACG00000008524"
## [13912] "ENSGACG00000008528" "ENSGACG00000026402" "ENSGACG00000008609"
## [13915] "ENSGACG00000008616" "ENSGACG00000008644" "ENSGACG00000032329"
## [13918] "ENSGACG00000008736" "ENSGACG00000008751" "ENSGACG00000029036"
## [13921] "ENSGACG00000033749" "ENSGACG00000008793" "ENSGACG00000008811"
## [13924] "ENSGACG00000008829" "ENSGACG00000029135" "ENSGACG00000008890"
## [13927] "ENSGACG00000031678" "ENSGACG00000008932" "ENSGACG00000036448"
## [13930] "ENSGACG00000037131" "ENSGACG00000036409" "ENSGACG00000032683"
## [13933] "ENSGACG00000008985" "ENSGACG00000008990" "ENSGACG00000009007"
## [13936] "ENSGACG00000033393" "ENSGACG00000036094" "ENSGACG00000035436"
## [13939] "ENSGACG00000009720" "ENSGACG00000009742" "ENSGACG00000009756"
## [13942] "ENSGACG00000009797" "ENSGACG00000009800" "ENSGACG00000009812"
## [13945] "ENSGACG00000009876" "ENSGACG00000009886" "ENSGACG00000009894"
## [13948] "ENSGACG00000009901" "ENSGACG00000009908" "ENSGACG00000009958"
## [13951] "ENSGACG00000009969" "ENSGACG00000010005" "ENSGACG00000024978"
## [13954] "ENSGACG00000034911" "ENSGACG00000010086" "ENSGACG00000033158"
## [13957] "ENSGACG00000026958" "ENSGACG00000028305" "ENSGACG00000010126"
## [13960] "ENSGACG00000010178" "ENSGACG00000010233" "ENSGACG00000034006"
## [13963] "ENSGACG00000024836" "ENSGACG00000037793" "ENSGACG00000033846"
## [13966] "ENSGACG00000025935" "ENSGACG00000030856" "ENSGACG00000036437"
## [13969] "ENSGACG00000035540" "ENSGACG00000031513" "ENSGACG00000023472"
## [13972] "ENSGACG00000029898" "ENSGACG00000037674" "ENSGACG00000025261"
## [13975] "ENSGACG00000026802" "ENSGACG00000033311" "ENSGACG00000030162"
## [13978] "ENSGACG00000034458" "ENSGACG00000035225" "ENSGACG00000037688"
## [13981] "ENSGACG00000027756" "ENSGACG00000024725" "ENSGACG00000035635"
## [13984] "ENSGACG00000031256" "ENSGACG00000036457" "ENSGACG00000026246"
## [13987] "ENSGACG00000035824" "ENSGACG00000036073" "ENSGACG00000028934"
## [13990] "ENSGACG00000035348" "ENSGACG00000028632" "ENSGACG00000026249"
## [13993] "ENSGACG00000032269" "ENSGACG00000033857" "ENSGACG00000035172"
## [13996] "ENSGACG00000030655" "ENSGACG00000025898" "ENSGACG00000009151"
## [13999] "ENSGACG00000031658" "ENSGACG00000027155" "ENSGACG00000025072"
## [14002] "ENSGACG00000012336" "ENSGACG00000010840" "ENSGACG00000009658"
## [14005] "ENSGACG00000009287" "ENSGACG00000010338" "ENSGACG00000007680"
## [14008] "ENSGACG00000026967" "ENSGACG00000025728" "ENSGACG00000010110"
## [14011] "ENSGACG00000002315" "ENSGACG00000002318" "ENSGACG00000002319"
## [14014] "ENSGACG00000037434" "ENSGACG00000002329" "ENSGACG00000002331"
## [14017] "ENSGACG00000002341" "ENSGACG00000033544" "ENSGACG00000004540"
## [14020] "ENSGACG00000034120" "ENSGACG00000025266" "ENSGACG00000004549"
## [14023] "ENSGACG00000032761" "ENSGACG00000000091" "ENSGACG00000000088"
## [14026] "ENSGACG00000004565" "ENSGACG00000004581" "ENSGACG00000031883"
## [14029] "ENSGACG00000002619" "ENSGACG00000031647" "ENSGACG00000002442"
## [14032] "ENSGACG00000002446" "ENSGACG00000002451" "ENSGACG00000002455"
## [14035] "ENSGACG00000002474" "ENSGACG00000002481" "ENSGACG00000002484"
## [14038] "ENSGACG00000002488" "ENSGACG00000002503" "ENSGACG00000002516"
## [14041] "ENSGACG00000037178" "ENSGACG00000002526" "ENSGACG00000002533"
## [14044] "ENSGACG00000002539" "ENSGACG00000002545" "ENSGACG00000002548"
## [14047] "ENSGACG00000002556" "ENSGACG00000002562" "ENSGACG00000002563"
## [14050] "ENSGACG00000002566" "ENSGACG00000004644" "ENSGACG00000037819"
## [14053] "ENSGACG00000031785" "ENSGACG00000029444" "ENSGACG00000004704"
## [14056] "ENSGACG00000004716" "ENSGACG00000032228" "ENSGACG00000027895"
## [14059] "ENSGACG00000024716" "ENSGACG00000004765" "ENSGACG00000029243"
## [14062] "ENSGACG00000004761" "ENSGACG00000031010" "ENSGACG00000004769"
## [14065] "ENSGACG00000004796" "ENSGACG00000004817" "ENSGACG00000004837"
## [14068] "ENSGACG00000004846" "ENSGACG00000004872" "ENSGACG00000004879"
## [14071] "ENSGACG00000004892" "ENSGACG00000004959" "ENSGACG00000004965"
## [14074] "ENSGACG00000004976" "ENSGACG00000004980" "ENSGACG00000004986"
## [14077] "ENSGACG00000004997" "ENSGACG00000005002" "ENSGACG00000005059"
## [14080] "ENSGACG00000033980" "ENSGACG00000005082" "ENSGACG00000005101"
## [14083] "ENSGACG00000023600" "ENSGACG00000005113" "ENSGACG00000005128"
## [14086] "ENSGACG00000005147" "ENSGACG00000023692" "ENSGACG00000005164"
## [14089] "ENSGACG00000005185" "ENSGACG00000025967" "ENSGACG00000005197"
## [14092] "ENSGACG00000005201" "ENSGACG00000005220" "ENSGACG00000030340"
## [14095] "ENSGACG00000028670" "ENSGACG00000035599" "ENSGACG00000005238"
## [14098] "ENSGACG00000005266" "ENSGACG00000005283" "ENSGACG00000005407"
## [14101] "ENSGACG00000005348" "ENSGACG00000028703" "ENSGACG00000005401"
## [14104] "ENSGACG00000028808" "ENSGACG00000005427" "ENSGACG00000002575"
## [14107] "ENSGACG00000026064" "ENSGACG00000002573" "ENSGACG00000035020"
## [14110] "ENSGACG00000002583" "ENSGACG00000002607" "ENSGACG00000002615"
## [14113] "ENSGACG00000033805" "ENSGACG00000002641" "ENSGACG00000002629"
## [14116] "ENSGACG00000002653" "ENSGACG00000002660" "ENSGACG00000002669"
## [14119] "ENSGACG00000002672" "ENSGACG00000026588" "ENSGACG00000033189"
## [14122] "ENSGACG00000032611" "ENSGACG00000027228" "ENSGACG00000002683"
## [14125] "ENSGACG00000002689" "ENSGACG00000002694" "ENSGACG00000002696"
## [14128] "ENSGACG00000002703" "ENSGACG00000002716" "ENSGACG00000033992"
## [14131] "ENSGACG00000002740" "ENSGACG00000002741" "ENSGACG00000002754"
## [14134] "ENSGACG00000002769" "ENSGACG00000002811" "ENSGACG00000002835"
## [14137] "ENSGACG00000002872" "ENSGACG00000002884" "ENSGACG00000002885"
## [14140] "ENSGACG00000002895" "ENSGACG00000023372" "ENSGACG00000032077"
## [14143] "ENSGACG00000002898" "ENSGACG00000028988" "ENSGACG00000002914"
## [14146] "ENSGACG00000002916" "ENSGACG00000031129" "ENSGACG00000002927"
## [14149] "ENSGACG00000002935" "ENSGACG00000023126" "ENSGACG00000002946"
## [14152] "ENSGACG00000002956" "ENSGACG00000002972" "ENSGACG00000030299"
## [14155] "ENSGACG00000002984" "ENSGACG00000002989" "ENSGACG00000028228"
## [14158] "ENSGACG00000002999" "ENSGACG00000003844" "ENSGACG00000003870"
## [14161] "ENSGACG00000003902" "ENSGACG00000028786" "ENSGACG00000003919"
## [14164] "ENSGACG00000032861" "ENSGACG00000003175" "ENSGACG00000003183"
## [14167] "ENSGACG00000003192" "ENSGACG00000027195" "ENSGACG00000003819"
## [14170] "ENSGACG00000004526" "ENSGACG00000004534" "ENSGACG00000004332"
## [14173] "ENSGACG00000036556" "ENSGACG00000003938" "ENSGACG00000003970"
## [14176] "ENSGACG00000003981" "ENSGACG00000003995" "ENSGACG00000004011"
## [14179] "ENSGACG00000004021" "ENSGACG00000004032" "ENSGACG00000035214"
## [14182] "ENSGACG00000010649" "ENSGACG00000004051" "ENSGACG00000004066"
## [14185] "ENSGACG00000023326" "ENSGACG00000030283" "ENSGACG00000004109"
## [14188] "ENSGACG00000004141" "ENSGACG00000004143" "ENSGACG00000004161"
## [14191] "ENSGACG00000032728" "ENSGACG00000034537" "ENSGACG00000032156"
## [14194] "ENSGACG00000025235" "ENSGACG00000004184" "ENSGACG00000004192"
## [14197] "ENSGACG00000004195" "ENSGACG00000004199" "ENSGACG00000004206"
## [14200] "ENSGACG00000004219" "ENSGACG00000004227" "ENSGACG00000004231"
## [14203] "ENSGACG00000004235" "ENSGACG00000004248" "ENSGACG00000004267"
## [14206] "ENSGACG00000004271" "ENSGACG00000004307" "ENSGACG00000028310"
## [14209] "ENSGACG00000004393" "ENSGACG00000004434" "ENSGACG00000004446"
## [14212] "ENSGACG00000004466" "ENSGACG00000004468" "ENSGACG00000004475"
## [14215] "ENSGACG00000025644" "ENSGACG00000030149" "ENSGACG00000004501"
## [14218] "ENSGACG00000031273" "ENSGACG00000025865" "ENSGACG00000027484"
## [14221] "ENSGACG00000027059" "ENSGACG00000002344" "ENSGACG00000026206"
## [14224] "ENSGACG00000002352" "ENSGACG00000032841" "ENSGACG00000027479"
## [14227] "ENSGACG00000002357" "ENSGACG00000002364" "ENSGACG00000002372"
## [14230] "ENSGACG00000002383" "ENSGACG00000028712" "ENSGACG00000002391"
## [14233] "ENSGACG00000002413" "ENSGACG00000002417" "ENSGACG00000003014"
## [14236] "ENSGACG00000028192" "ENSGACG00000037465" "ENSGACG00000003020"
## [14239] "ENSGACG00000025980" "ENSGACG00000003078" "ENSGACG00000023337"
## [14242] "ENSGACG00000003085" "ENSGACG00000003109" "ENSGACG00000037469"
## [14245] "ENSGACG00000003120" "ENSGACG00000003131" "ENSGACG00000003140"
## [14248] "ENSGACG00000003155" "ENSGACG00000029282" "ENSGACG00000028184"
## [14251] "ENSGACG00000003202" "ENSGACG00000003336" "ENSGACG00000026870"
## [14254] "ENSGACG00000003232" "ENSGACG00000003246" "ENSGACG00000003274"
## [14257] "ENSGACG00000003280" "ENSGACG00000003287" "ENSGACG00000029403"
## [14260] "ENSGACG00000023975" "ENSGACG00000003303" "ENSGACG00000003327"
## [14263] "ENSGACG00000038039" "ENSGACG00000003363" "ENSGACG00000003375"
## [14266] "ENSGACG00000026208" "ENSGACG00000027440" "ENSGACG00000003409"
## [14269] "ENSGACG00000023746" "ENSGACG00000031631" "ENSGACG00000030908"
## [14272] "ENSGACG00000037146" "ENSGACG00000003445" "ENSGACG00000003451"
## [14275] "ENSGACG00000024373" "ENSGACG00000003457" "ENSGACG00000003469"
## [14278] "ENSGACG00000003472" "ENSGACG00000003477" "ENSGACG00000003517"
## [14281] "ENSGACG00000026592" "ENSGACG00000003533" "ENSGACG00000033626"
## [14284] "ENSGACG00000003558" "ENSGACG00000034475" "ENSGACG00000003591"
## [14287] "ENSGACG00000003599" "ENSGACG00000024858" "ENSGACG00000003645"
## [14290] "ENSGACG00000033507" "ENSGACG00000003656" "ENSGACG00000003662"
## [14293] "ENSGACG00000003666" "ENSGACG00000003674" "ENSGACG00000003682"
## [14296] "ENSGACG00000003684" "ENSGACG00000003695" "ENSGACG00000029111"
## [14299] "ENSGACG00000031052" "ENSGACG00000003700" "ENSGACG00000003771"
## [14302] "ENSGACG00000003808" "ENSGACG00000003809" "ENSGACG00000001379"
## [14305] "ENSGACG00000001388" "ENSGACG00000001408" "ENSGACG00000001433"
## [14308] "ENSGACG00000038095" "ENSGACG00000001443" "ENSGACG00000001452"
## [14311] "ENSGACG00000001461" "ENSGACG00000001464" "ENSGACG00000036166"
## [14314] "ENSGACG00000035792" "ENSGACG00000001471" "ENSGACG00000034237"
## [14317] "ENSGACG00000026154" "ENSGACG00000001324" "ENSGACG00000001310"
## [14320] "ENSGACG00000001308" "ENSGACG00000001305" "ENSGACG00000001301"
## [14323] "ENSGACG00000001296" "ENSGACG00000026488" "ENSGACG00000023611"
## [14326] "ENSGACG00000001289" "ENSGACG00000023711" "ENSGACG00000034831"
## [14329] "ENSGACG00000001284" "ENSGACG00000009277" "ENSGACG00000025920"
## [14332] "ENSGACG00000036367" "ENSGACG00000009283" "ENSGACG00000000543"
## [14335] "ENSGACG00000000541" "ENSGACG00000000538" "ENSGACG00000009006"
## [14338] "ENSGACG00000000532" "ENSGACG00000000529" "ENSGACG00000000514"
## [14341] "ENSGACG00000000510" "ENSGACG00000000507" "ENSGACG00000025278"
## [14344] "ENSGACG00000034212" "ENSGACG00000034060" "ENSGACG00000030070"
## [14347] "ENSGACG00000000492" "ENSGACG00000034549" "ENSGACG00000002018"
## [14350] "ENSGACG00000009020" "ENSGACG00000032359" "ENSGACG00000026152"
## [14353] "ENSGACG00000009058" "ENSGACG00000009088" "ENSGACG00000009095"
## [14356] "ENSGACG00000009098" "ENSGACG00000025548" "ENSGACG00000009137"
## [14359] "ENSGACG00000029205" "ENSGACG00000000481" "ENSGACG00000000455"
## [14362] "ENSGACG00000009140" "ENSGACG00000030455" "ENSGACG00000036404"
## [14365] "ENSGACG00000027327" "ENSGACG00000009150" "ENSGACG00000029018"
## [14368] "ENSGACG00000037746" "ENSGACG00000000147" "ENSGACG00000029492"
## [14371] "ENSGACG00000029200" "ENSGACG00000000142" "ENSGACG00000009219"
## [14374] "ENSGACG00000009223" "ENSGACG00000026298" "ENSGACG00000030071"
## [14377] "ENSGACG00000027824" "ENSGACG00000004784" "ENSGACG00000009267"
## [14380] "ENSGACG00000009365" "ENSGACG00000028174" "ENSGACG00000009384"
## [14383] "ENSGACG00000025067" "ENSGACG00000009395" "ENSGACG00000001977"
## [14386] "ENSGACG00000001982" "ENSGACG00000001989" "ENSGACG00000001992"
## [14389] "ENSGACG00000014716" "ENSGACG00000001994" "ENSGACG00000000130"
## [14392] "ENSGACG00000000107" "ENSGACG00000027761" "ENSGACG00000027687"
## [14395] "ENSGACG00000025146" "ENSGACG00000000117" "ENSGACG00000029296"
## [14398] "ENSGACG00000010502" "ENSGACG00000026557" "ENSGACG00000034283"
## [14401] "ENSGACG00000035481" "ENSGACG00000034657" "ENSGACG00000017802"
## [14404] "ENSGACG00000005835" "ENSGACG00000000099" "ENSGACG00000032795"
## [14407] "ENSGACG00000034231" "ENSGACG00000005879" "ENSGACG00000005928"
## [14410] "ENSGACG00000005895" "ENSGACG00000005913" "ENSGACG00000000103"
## [14413] "ENSGACG00000033462" "ENSGACG00000005947" "ENSGACG00000005961"
## [14416] "ENSGACG00000005968" "ENSGACG00000000096" "ENSGACG00000005983"
## [14419] "ENSGACG00000000094" "ENSGACG00000000085" "ENSGACG00000027516"
## [14422] "ENSGACG00000000074" "ENSGACG00000035553" "ENSGACG00000024504"
## [14425] "ENSGACG00000005992" "ENSGACG00000000067" "ENSGACG00000005998"
## [14428] "ENSGACG00000006011" "ENSGACG00000006027" "ENSGACG00000006031"
## [14431] "ENSGACG00000006036" "ENSGACG00000027800" "ENSGACG00000001844"
## [14434] "ENSGACG00000001856" "ENSGACG00000031144" "ENSGACG00000001865"
## [14437] "ENSGACG00000006054" "ENSGACG00000006062" "ENSGACG00000001879"
## [14440] "ENSGACG00000006077" "ENSGACG00000001882" "ENSGACG00000026543"
## [14443] "ENSGACG00000026177" "ENSGACG00000030238" "ENSGACG00000006108"
## [14446] "ENSGACG00000005445" "ENSGACG00000028680" "ENSGACG00000036935"
## [14449] "ENSGACG00000036060" "ENSGACG00000005497" "ENSGACG00000001913"
## [14452] "ENSGACG00000001921" "ENSGACG00000001923" "ENSGACG00000028514"
## [14455] "ENSGACG00000029349" "ENSGACG00000032778" "ENSGACG00000001959"
## [14458] "ENSGACG00000001968" "ENSGACG00000031490" "ENSGACG00000001973"
## [14461] "ENSGACG00000001981" "ENSGACG00000028445" "ENSGACG00000001987"
## [14464] "ENSGACG00000035863" "ENSGACG00000001991" "ENSGACG00000001993"
## [14467] "ENSGACG00000026061" "ENSGACG00000006126" "ENSGACG00000005520"
## [14470] "ENSGACG00000005555" "ENSGACG00000005581" "ENSGACG00000005591"
## [14473] "ENSGACG00000005594" "ENSGACG00000005599" "ENSGACG00000005616"
## [14476] "ENSGACG00000005655" "ENSGACG00000005700" "ENSGACG00000005717"
## [14479] "ENSGACG00000005736" "ENSGACG00000005741" "ENSGACG00000005748"
## [14482] "ENSGACG00000005767" "ENSGACG00000030048" "ENSGACG00000005777"
## [14485] "ENSGACG00000005789" "ENSGACG00000002002" "ENSGACG00000005790"
## [14488] "ENSGACG00000002003" "ENSGACG00000005798" "ENSGACG00000005801"
## [14491] "ENSGACG00000002010" "ENSGACG00000005816" "ENSGACG00000002029"
## [14494] "ENSGACG00000034520" "ENSGACG00000030185" "ENSGACG00000002034"
## [14497] "ENSGACG00000036994" "ENSGACG00000009354" "ENSGACG00000009359"
## [14500] "ENSGACG00000033496" "ENSGACG00000031905" "ENSGACG00000009362"
## [14503] "ENSGACG00000002050" "ENSGACG00000006436" "ENSGACG00000031254"
## [14506] "ENSGACG00000002085" "ENSGACG00000006256" "ENSGACG00000006264"
## [14509] "ENSGACG00000002089" "ENSGACG00000002118" "ENSGACG00000006273"
## [14512] "ENSGACG00000002182" "ENSGACG00000002224" "ENSGACG00000033756"
## [14515] "ENSGACG00000002230" "ENSGACG00000026594" "ENSGACG00000031794"
## [14518] "ENSGACG00000002253" "ENSGACG00000006302" "ENSGACG00000002257"
## [14521] "ENSGACG00000002270" "ENSGACG00000002271" "ENSGACG00000006322"
## [14524] "ENSGACG00000002293" "ENSGACG00000006323" "ENSGACG00000023057"
## [14527] "ENSGACG00000006358" "ENSGACG00000006364" "ENSGACG00000002312"
## [14530] "ENSGACG00000002688" "ENSGACG00000023839" "ENSGACG00000026726"
## [14533] "ENSGACG00000034471" "ENSGACG00000025423" "ENSGACG00000025606"
## [14536] "ENSGACG00000038002" "ENSGACG00000032287" "ENSGACG00000030398"
## [14539] "ENSGACG00000029874" "ENSGACG00000032621" "ENSGACG00000037957"
## [14542] "ENSGACG00000026395" "ENSGACG00000023250" "ENSGACG00000030186"
## [14545] "ENSGACG00000027635" "ENSGACG00000023389" "ENSGACG00000036096"
## [14548] "ENSGACG00000037876" "ENSGACG00000027267" "ENSGACG00000032643"
## [14551] "ENSGACG00000034422" "ENSGACG00000025554" "ENSGACG00000027063"
## [14554] "ENSGACG00000026333" "ENSGACG00000025088" "ENSGACG00000027573"
## [14557] "ENSGACG00000028884" "ENSGACG00000034267" "ENSGACG00000033051"
## [14560] "ENSGACG00000025735" "ENSGACG00000034138" "ENSGACG00000027501"
## [14563] "ENSGACG00000035897" "ENSGACG00000034753" "ENSGACG00000006390"
## [14566] "ENSGACG00000029376" "ENSGACG00000036982" "ENSGACG00000033107"
## [14569] "ENSGACG00000006404" "ENSGACG00000029648" "ENSGACG00000028960"
## [14572] "ENSGACG00000033320" "ENSGACG00000031866" "ENSGACG00000029809"
## [14575] "ENSGACG00000036490" "ENSGACG00000023662" "ENSGACG00000027169"
## [14578] "ENSGACG00000023710" "ENSGACG00000024450" "ENSGACG00000034265"
## [14581] "ENSGACG00000028147" "ENSGACG00000006430" "ENSGACG00000006439"
## [14584] "ENSGACG00000036479" "ENSGACG00000006479" "ENSGACG00000031815"
## [14587] "ENSGACG00000006481" "ENSGACG00000036795" "ENSGACG00000024218"
## [14590] "ENSGACG00000032480" "ENSGACG00000029206" "ENSGACG00000006511"
## [14593] "ENSGACG00000033316" "ENSGACG00000029064" "ENSGACG00000036471"
## [14596] "ENSGACG00000022567" "ENSGACG00000036568" "ENSGACG00000003898"
## [14599] "ENSGACG00000036654" "ENSGACG00000004084" "ENSGACG00000026673"
## [14602] "ENSGACG00000003058" "ENSGACG00000003061" "ENSGACG00000003220"
## [14605] "ENSGACG00000003566" "ENSGACG00000029746" "ENSGACG00000001291"
## [14608] "ENSGACG00000006571" "ENSGACG00000006592" "ENSGACG00000006652"
## [14611] "ENSGACG00000006677" "ENSGACG00000006745" "ENSGACG00000000499"
## [14614] "ENSGACG00000037368" "ENSGACG00000000143" "ENSGACG00000000112"
## [14617] "ENSGACG00000036439" "ENSGACG00000000141" "ENSGACG00000006683"
## [14620] "ENSGACG00000002247" "ENSGACG00000006755" "ENSGACG00000006767"
## [14623] "ENSGACG00000027950" "ENSGACG00000007719" "ENSGACG00000027600"
## [14626] "ENSGACG00000007747" "ENSGACG00000025250" "ENSGACG00000007773"
## [14629] "ENSGACG00000007791" "ENSGACG00000007801" "ENSGACG00000018725"
## [14632] "ENSGACG00000018722" "ENSGACG00000020297" "ENSGACG00000018705"
## [14635] "ENSGACG00000018701" "ENSGACG00000007537" "ENSGACG00000033914"
## [14638] "ENSGACG00000007448" "ENSGACG00000007476" "ENSGACG00000007485"
## [14641] "ENSGACG00000025287" "ENSGACG00000037923" "ENSGACG00000007627"
## [14644] "ENSGACG00000007662" "ENSGACG00000026435" "ENSGACG00000007683"
## [14647] "ENSGACG00000007689" "ENSGACG00000023067" "ENSGACG00000007702"
## [14650] "ENSGACG00000007715" "ENSGACG00000024523" "ENSGACG00000025073"
## [14653] "ENSGACG00000038019" "ENSGACG00000008471" "ENSGACG00000023816"
## [14656] "ENSGACG00000008486" "ENSGACG00000008493" "ENSGACG00000008503"
## [14659] "ENSGACG00000030861" "ENSGACG00000008516" "ENSGACG00000027471"
## [14662] "ENSGACG00000025651" "ENSGACG00000008586" "ENSGACG00000008669"
## [14665] "ENSGACG00000008672" "ENSGACG00000008676" "ENSGACG00000008688"
## [14668] "ENSGACG00000008703" "ENSGACG00000008798" "ENSGACG00000008805"
## [14671] "ENSGACG00000007413" "ENSGACG00000008847" "ENSGACG00000008853"
## [14674] "ENSGACG00000008862" "ENSGACG00000008892" "ENSGACG00000023619"
## [14677] "ENSGACG00000008922" "ENSGACG00000008940" "ENSGACG00000008953"
## [14680] "ENSGACG00000037705" "ENSGACG00000008974" "ENSGACG00000033858"
## [14683] "ENSGACG00000009411" "ENSGACG00000009422" "ENSGACG00000009460"
## [14686] "ENSGACG00000027188" "ENSGACG00000009532" "ENSGACG00000009542"
## [14689] "ENSGACG00000009551" "ENSGACG00000009573" "ENSGACG00000009593"
## [14692] "ENSGACG00000009609" "ENSGACG00000009644" "ENSGACG00000026143"
## [14695] "ENSGACG00000037310" "ENSGACG00000005239" "ENSGACG00000009659"
## [14698] "ENSGACG00000009662" "ENSGACG00000009688" "ENSGACG00000009702"
## [14701] "ENSGACG00000009731" "ENSGACG00000020122" "ENSGACG00000034844"
## [14704] "ENSGACG00000022994" "ENSGACG00000009744" "ENSGACG00000009746"
## [14707] "ENSGACG00000024786" "ENSGACG00000029742" "ENSGACG00000035791"
## [14710] "ENSGACG00000037228" "ENSGACG00000025064" "ENSGACG00000007842"
## [14713] "ENSGACG00000030101" "ENSGACG00000028977" "ENSGACG00000026229"
## [14716] "ENSGACG00000007986" "ENSGACG00000007996" "ENSGACG00000007999"
## [14719] "ENSGACG00000008006" "ENSGACG00000008024" "ENSGACG00000008045"
## [14722] "ENSGACG00000037621" "ENSGACG00000008060" "ENSGACG00000008092"
## [14725] "ENSGACG00000008109" "ENSGACG00000008118" "ENSGACG00000024835"
## [14728] "ENSGACG00000033487" "ENSGACG00000029204" "ENSGACG00000036723"
## [14731] "ENSGACG00000029504" "ENSGACG00000008174" "ENSGACG00000009753"
## [14734] "ENSGACG00000009770" "ENSGACG00000009772" "ENSGACG00000009814"
## [14737] "ENSGACG00000009833" "ENSGACG00000009862" "ENSGACG00000028440"
## [14740] "ENSGACG00000033169" "ENSGACG00000029451" "ENSGACG00000009889"
## [14743] "ENSGACG00000029855" "ENSGACG00000009923" "ENSGACG00000009933"
## [14746] "ENSGACG00000009954" "ENSGACG00000009960" "ENSGACG00000032792"
## [14749] "ENSGACG00000009962" "ENSGACG00000009966" "ENSGACG00000009977"
## [14752] "ENSGACG00000009982" "ENSGACG00000010041" "ENSGACG00000026290"
## [14755] "ENSGACG00000029677" "ENSGACG00000026889" "ENSGACG00000010063"
## [14758] "ENSGACG00000010069" "ENSGACG00000010080" "ENSGACG00000008225"
## [14761] "ENSGACG00000034604" "ENSGACG00000008250" "ENSGACG00000027900"
## [14764] "ENSGACG00000025785" "ENSGACG00000008253" "ENSGACG00000030546"
## [14767] "ENSGACG00000036309" "ENSGACG00000008290" "ENSGACG00000025077"
## [14770] "ENSGACG00000008309" "ENSGACG00000033105" "ENSGACG00000008319"
## [14773] "ENSGACG00000010314" "ENSGACG00000008328" "ENSGACG00000008422"
## [14776] "ENSGACG00000008455" "ENSGACG00000010334" "ENSGACG00000010341"
## [14779] "ENSGACG00000010362" "ENSGACG00000035068" "ENSGACG00000010376"
## [14782] "ENSGACG00000027898" "ENSGACG00000029695" "ENSGACG00000010388"
## [14785] "ENSGACG00000010409" "ENSGACG00000010415" "ENSGACG00000010420"
## [14788] "ENSGACG00000010434" "ENSGACG00000006780" "ENSGACG00000028155"
## [14791] "ENSGACG00000006796" "ENSGACG00000006849" "ENSGACG00000006863"
## [14794] "ENSGACG00000037221" "ENSGACG00000029302" "ENSGACG00000006878"
## [14797] "ENSGACG00000006894" "ENSGACG00000006942" "ENSGACG00000035409"
## [14800] "ENSGACG00000006954" "ENSGACG00000006960" "ENSGACG00000006967"
## [14803] "ENSGACG00000035994" "ENSGACG00000007006" "ENSGACG00000007010"
## [14806] "ENSGACG00000007024" "ENSGACG00000007025" "ENSGACG00000007031"
## [14809] "ENSGACG00000007049" "ENSGACG00000038022" "ENSGACG00000007066"
## [14812] "ENSGACG00000007069" "ENSGACG00000026027" "ENSGACG00000026452"
## [14815] "ENSGACG00000035147" "ENSGACG00000007108" "ENSGACG00000028913"
## [14818] "ENSGACG00000007155" "ENSGACG00000007180" "ENSGACG00000030464"
## [14821] "ENSGACG00000007196" "ENSGACG00000007203" "ENSGACG00000007208"
## [14824] "ENSGACG00000007216" "ENSGACG00000024646" "ENSGACG00000007223"
## [14827] "ENSGACG00000007231" "ENSGACG00000007236" "ENSGACG00000007251"
## [14830] "ENSGACG00000007254" "ENSGACG00000007293" "ENSGACG00000007310"
## [14833] "ENSGACG00000030476" "ENSGACG00000007343" "ENSGACG00000007359"
## [14836] "ENSGACG00000007372" "ENSGACG00000023787" "ENSGACG00000029255"
## [14839] "ENSGACG00000033373" "ENSGACG00000031322" "ENSGACG00000027294"
## [14842] "ENSGACG00000037555" "ENSGACG00000030494" "ENSGACG00000034988"
## [14845] "ENSGACG00000029671" "ENSGACG00000024513" "ENSGACG00000035811"
## [14848] "ENSGACG00000036297" "ENSGACG00000035389" "ENSGACG00000032064"
## [14851] "ENSGACG00000035585" "ENSGACG00000026503" "ENSGACG00000032695"
## [14854] "ENSGACG00000025772" "ENSGACG00000030667" "ENSGACG00000030306"
## [14857] "ENSGACG00000024859" "ENSGACG00000031552" "ENSGACG00000023697"
## [14860] "ENSGACG00000034874" "ENSGACG00000038066" "ENSGACG00000034797"
## [14863] "ENSGACG00000037755" "ENSGACG00000029617" "ENSGACG00000027176"
## [14866] "ENSGACG00000033731" "ENSGACG00000026586" "ENSGACG00000036040"
## [14869] "ENSGACG00000023431" "ENSGACG00000028462" "ENSGACG00000026160"
## [14872] "ENSGACG00000037060" "ENSGACG00000030400" "ENSGACG00000030083"
## [14875] "ENSGACG00000037726" "ENSGACG00000033515" "ENSGACG00000026606"
## [14878] "ENSGACG00000031731" "ENSGACG00000031298" "ENSGACG00000030129"
## [14881] "ENSGACG00000030594" "ENSGACG00000031707" "ENSGACG00000035085"
## [14884] "ENSGACG00000023333" "ENSGACG00000023935" "ENSGACG00000032784"
## [14887] "ENSGACG00000037402" "ENSGACG00000029422" "ENSGACG00000028302"
## [14890] "ENSGACG00000029442" "ENSGACG00000024368" "ENSGACG00000028309"
## [14893] "ENSGACG00000031230" "ENSGACG00000009108" "ENSGACG00000009249"
## [14896] "ENSGACG00000005874" "ENSGACG00000007518" "ENSGACG00000007500"
## [14899] "ENSGACG00000007547" "ENSGACG00000031167" "ENSGACG00000007938"
## [14902] "ENSGACG00000010051" "ENSGACG00000008242" "ENSGACG00000033747"
## [14905] "ENSGACG00000034810" "ENSGACG00000034540" "ENSGACG00000014478"
## [14908] "ENSGACG00000014494" "ENSGACG00000014501" "ENSGACG00000014515"
## [14911] "ENSGACG00000014519" "ENSGACG00000014521" "ENSGACG00000033729"
## [14914] "ENSGACG00000014536" "ENSGACG00000030538" "ENSGACG00000013446"
## [14917] "ENSGACG00000028522" "ENSGACG00000013459" "ENSGACG00000013468"
## [14920] "ENSGACG00000032892" "ENSGACG00000013485" "ENSGACG00000014069"
## [14923] "ENSGACG00000030729" "ENSGACG00000014076" "ENSGACG00000014086"
## [14926] "ENSGACG00000014096" "ENSGACG00000014107" "ENSGACG00000033565"
## [14929] "ENSGACG00000034665" "ENSGACG00000012944" "ENSGACG00000012972"
## [14932] "ENSGACG00000012991" "ENSGACG00000012999" "ENSGACG00000013009"
## [14935] "ENSGACG00000013017" "ENSGACG00000013024" "ENSGACG00000013069"
## [14938] "ENSGACG00000029835" "ENSGACG00000013093" "ENSGACG00000013099"
## [14941] "ENSGACG00000013105" "ENSGACG00000013114" "ENSGACG00000013161"
## [14944] "ENSGACG00000013163" "ENSGACG00000013168" "ENSGACG00000013183"
## [14947] "ENSGACG00000013193" "ENSGACG00000013222" "ENSGACG00000013236"
## [14950] "ENSGACG00000013251" "ENSGACG00000035111" "ENSGACG00000013256"
## [14953] "ENSGACG00000013290" "ENSGACG00000013300" "ENSGACG00000013317"
## [14956] "ENSGACG00000013347" "ENSGACG00000013368" "ENSGACG00000013369"
## [14959] "ENSGACG00000013375" "ENSGACG00000013392" "ENSGACG00000013399"
## [14962] "ENSGACG00000013405" "ENSGACG00000013411" "ENSGACG00000013415"
## [14965] "ENSGACG00000014060" "ENSGACG00000030544" "ENSGACG00000024141"
## [14968] "ENSGACG00000013743" "ENSGACG00000014122" "ENSGACG00000015316"
## [14971] "ENSGACG00000014996" "ENSGACG00000015189" "ENSGACG00000015191"
## [14974] "ENSGACG00000015199" "ENSGACG00000015206" "ENSGACG00000026882"
## [14977] "ENSGACG00000015211" "ENSGACG00000026562" "ENSGACG00000015219"
## [14980] "ENSGACG00000015222" "ENSGACG00000015225" "ENSGACG00000036112"
## [14983] "ENSGACG00000015003" "ENSGACG00000015067" "ENSGACG00000015014"
## [14986] "ENSGACG00000025505" "ENSGACG00000015019" "ENSGACG00000015034"
## [14989] "ENSGACG00000015039" "ENSGACG00000015042" "ENSGACG00000015049"
## [14992] "ENSGACG00000015064" "ENSGACG00000015065" "ENSGACG00000032331"
## [14995] "ENSGACG00000015073" "ENSGACG00000015077" "ENSGACG00000015080"
## [14998] "ENSGACG00000015082" "ENSGACG00000015088" "ENSGACG00000015090"
## [15001] "ENSGACG00000015101" "ENSGACG00000035907" "ENSGACG00000028875"
## [15004] "ENSGACG00000015109" "ENSGACG00000015317" "ENSGACG00000015324"
## [15007] "ENSGACG00000015130" "ENSGACG00000015326" "ENSGACG00000015136"
## [15010] "ENSGACG00000025097" "ENSGACG00000015332" "ENSGACG00000015145"
## [15013] "ENSGACG00000015335" "ENSGACG00000015341" "ENSGACG00000027668"
## [15016] "ENSGACG00000015154" "ENSGACG00000015158" "ENSGACG00000015344"
## [15019] "ENSGACG00000015346" "ENSGACG00000015163" "ENSGACG00000036109"
## [15022] "ENSGACG00000015369" "ENSGACG00000036837" "ENSGACG00000015354"
## [15025] "ENSGACG00000014328" "ENSGACG00000014339" "ENSGACG00000014348"
## [15028] "ENSGACG00000014352" "ENSGACG00000015231" "ENSGACG00000015372"
## [15031] "ENSGACG00000015373" "ENSGACG00000015377" "ENSGACG00000015381"
## [15034] "ENSGACG00000030625" "ENSGACG00000025317" "ENSGACG00000032149"
## [15037] "ENSGACG00000031200" "ENSGACG00000015386" "ENSGACG00000014972"
## [15040] "ENSGACG00000013750" "ENSGACG00000025107" "ENSGACG00000013758"
## [15043] "ENSGACG00000013777" "ENSGACG00000013800" "ENSGACG00000013809"
## [15046] "ENSGACG00000013814" "ENSGACG00000013822" "ENSGACG00000013832"
## [15049] "ENSGACG00000013835" "ENSGACG00000013841" "ENSGACG00000013842"
## [15052] "ENSGACG00000028822" "ENSGACG00000037464" "ENSGACG00000013855"
## [15055] "ENSGACG00000013861" "ENSGACG00000013881" "ENSGACG00000013888"
## [15058] "ENSGACG00000027869" "ENSGACG00000029019" "ENSGACG00000013912"
## [15061] "ENSGACG00000034779" "ENSGACG00000034141" "ENSGACG00000013942"
## [15064] "ENSGACG00000013944" "ENSGACG00000013951" "ENSGACG00000014017"
## [15067] "ENSGACG00000014028" "ENSGACG00000014032" "ENSGACG00000014035"
## [15070] "ENSGACG00000014043" "ENSGACG00000014049" "ENSGACG00000015244"
## [15073] "ENSGACG00000015255" "ENSGACG00000029656" "ENSGACG00000015271"
## [15076] "ENSGACG00000015273" "ENSGACG00000015282" "ENSGACG00000031481"
## [15079] "ENSGACG00000015296" "ENSGACG00000015299" "ENSGACG00000015301"
## [15082] "ENSGACG00000015306" "ENSGACG00000015308" "ENSGACG00000015315"
## [15085] "ENSGACG00000014131" "ENSGACG00000014136" "ENSGACG00000027190"
## [15088] "ENSGACG00000014168" "ENSGACG00000027640" "ENSGACG00000014200"
## [15091] "ENSGACG00000014207" "ENSGACG00000014212" "ENSGACG00000023778"
## [15094] "ENSGACG00000014243" "ENSGACG00000014245" "ENSGACG00000014253"
## [15097] "ENSGACG00000014265" "ENSGACG00000023213" "ENSGACG00000014270"
## [15100] "ENSGACG00000014290" "ENSGACG00000014303" "ENSGACG00000032160"
## [15103] "ENSGACG00000028325" "ENSGACG00000014317" "ENSGACG00000014326"
## [15106] "ENSGACG00000035734" "ENSGACG00000014566" "ENSGACG00000024852"
## [15109] "ENSGACG00000014581" "ENSGACG00000014583" "ENSGACG00000014586"
## [15112] "ENSGACG00000014602" "ENSGACG00000014610" "ENSGACG00000014614"
## [15115] "ENSGACG00000026517" "ENSGACG00000014623" "ENSGACG00000014630"
## [15118] "ENSGACG00000014632" "ENSGACG00000014637" "ENSGACG00000014650"
## [15121] "ENSGACG00000028185" "ENSGACG00000014661" "ENSGACG00000014664"
## [15124] "ENSGACG00000014668" "ENSGACG00000014670" "ENSGACG00000014678"
## [15127] "ENSGACG00000014695" "ENSGACG00000014703" "ENSGACG00000014711"
## [15130] "ENSGACG00000014715" "ENSGACG00000014718" "ENSGACG00000014724"
## [15133] "ENSGACG00000014728" "ENSGACG00000014732" "ENSGACG00000014733"
## [15136] "ENSGACG00000014747" "ENSGACG00000025032" "ENSGACG00000014755"
## [15139] "ENSGACG00000037485" "ENSGACG00000014756" "ENSGACG00000033395"
## [15142] "ENSGACG00000014760" "ENSGACG00000014766" "ENSGACG00000014774"
## [15145] "ENSGACG00000027582" "ENSGACG00000014803" "ENSGACG00000014816"
## [15148] "ENSGACG00000014834" "ENSGACG00000023398" "ENSGACG00000014841"
## [15151] "ENSGACG00000014845" "ENSGACG00000014849" "ENSGACG00000014860"
## [15154] "ENSGACG00000014870" "ENSGACG00000014892" "ENSGACG00000014897"
## [15157] "ENSGACG00000014905" "ENSGACG00000014911" "ENSGACG00000035956"
## [15160] "ENSGACG00000014919" "ENSGACG00000014921" "ENSGACG00000014929"
## [15163] "ENSGACG00000035833" "ENSGACG00000014944" "ENSGACG00000029911"
## [15166] "ENSGACG00000014948" "ENSGACG00000028987" "ENSGACG00000014366"
## [15169] "ENSGACG00000014377" "ENSGACG00000014381" "ENSGACG00000037327"
## [15172] "ENSGACG00000014401" "ENSGACG00000014413" "ENSGACG00000025136"
## [15175] "ENSGACG00000014418" "ENSGACG00000014423" "ENSGACG00000031846"
## [15178] "ENSGACG00000014427" "ENSGACG00000026568" "ENSGACG00000014435"
## [15181] "ENSGACG00000014448" "ENSGACG00000014453" "ENSGACG00000014455"
## [15184] "ENSGACG00000035456" "ENSGACG00000014462" "ENSGACG00000014465"
## [15187] "ENSGACG00000013492" "ENSGACG00000013518" "ENSGACG00000013510"
## [15190] "ENSGACG00000027562" "ENSGACG00000013527" "ENSGACG00000028421"
## [15193] "ENSGACG00000016071" "ENSGACG00000028795" "ENSGACG00000016078"
## [15196] "ENSGACG00000013533" "ENSGACG00000013594" "ENSGACG00000013603"
## [15199] "ENSGACG00000013626" "ENSGACG00000013638" "ENSGACG00000013662"
## [15202] "ENSGACG00000013706" "ENSGACG00000013666" "ENSGACG00000013708"
## [15205] "ENSGACG00000013719" "ENSGACG00000013721" "ENSGACG00000032585"
## [15208] "ENSGACG00000013732" "ENSGACG00000015236" "ENSGACG00000015165"
## [15211] "ENSGACG00000015168" "ENSGACG00000027333" "ENSGACG00000015175"
## [15214] "ENSGACG00000015182" "ENSGACG00000016081" "ENSGACG00000016088"
## [15217] "ENSGACG00000029055" "ENSGACG00000016099" "ENSGACG00000016101"
## [15220] "ENSGACG00000016102" "ENSGACG00000016104" "ENSGACG00000016106"
## [15223] "ENSGACG00000024581" "ENSGACG00000028408" "ENSGACG00000034315"
## [15226] "ENSGACG00000029532" "ENSGACG00000016127" "ENSGACG00000016130"
## [15229] "ENSGACG00000016132" "ENSGACG00000016134" "ENSGACG00000016146"
## [15232] "ENSGACG00000016148" "ENSGACG00000016152" "ENSGACG00000016155"
## [15235] "ENSGACG00000016156" "ENSGACG00000016159" "ENSGACG00000016161"
## [15238] "ENSGACG00000037946" "ENSGACG00000016163" "ENSGACG00000016167"
## [15241] "ENSGACG00000016172" "ENSGACG00000016177" "ENSGACG00000016188"
## [15244] "ENSGACG00000030660" "ENSGACG00000016189" "ENSGACG00000016200"
## [15247] "ENSGACG00000035584" "ENSGACG00000036137" "ENSGACG00000016207"
## [15250] "ENSGACG00000016209" "ENSGACG00000016212" "ENSGACG00000016218"
## [15253] "ENSGACG00000031651" "ENSGACG00000016235" "ENSGACG00000016237"
## [15256] "ENSGACG00000016242" "ENSGACG00000016249" "ENSGACG00000024740"
## [15259] "ENSGACG00000016257" "ENSGACG00000016258" "ENSGACG00000016264"
## [15262] "ENSGACG00000016268" "ENSGACG00000026518" "ENSGACG00000016276"
## [15265] "ENSGACG00000016890" "ENSGACG00000016893" "ENSGACG00000016284"
## [15268] "ENSGACG00000016286" "ENSGACG00000036716" "ENSGACG00000016631"
## [15271] "ENSGACG00000016639" "ENSGACG00000016640" "ENSGACG00000016643"
## [15274] "ENSGACG00000016646" "ENSGACG00000032672" "ENSGACG00000016690"
## [15277] "ENSGACG00000035569" "ENSGACG00000026990" "ENSGACG00000033598"
## [15280] "ENSGACG00000012369" "ENSGACG00000036247" "ENSGACG00000012377"
## [15283] "ENSGACG00000016701" "ENSGACG00000016707" "ENSGACG00000012386"
## [15286] "ENSGACG00000012390" "ENSGACG00000012451" "ENSGACG00000025550"
## [15289] "ENSGACG00000038071" "ENSGACG00000012499" "ENSGACG00000012508"
## [15292] "ENSGACG00000012518" "ENSGACG00000016717" "ENSGACG00000012611"
## [15295] "ENSGACG00000016719" "ENSGACG00000012613" "ENSGACG00000028502"
## [15298] "ENSGACG00000016779" "ENSGACG00000016788" "ENSGACG00000016789"
## [15301] "ENSGACG00000024837" "ENSGACG00000016795" "ENSGACG00000016801"
## [15304] "ENSGACG00000026664" "ENSGACG00000016816" "ENSGACG00000016820"
## [15307] "ENSGACG00000032241" "ENSGACG00000016825" "ENSGACG00000029124"
## [15310] "ENSGACG00000016865" "ENSGACG00000016844" "ENSGACG00000016850"
## [15313] "ENSGACG00000016883" "ENSGACG00000016677" "ENSGACG00000012625"
## [15316] "ENSGACG00000016680" "ENSGACG00000023603" "ENSGACG00000012659"
## [15319] "ENSGACG00000016898" "ENSGACG00000012661" "ENSGACG00000012667"
## [15322] "ENSGACG00000016905" "ENSGACG00000016914" "ENSGACG00000012674"
## [15325] "ENSGACG00000016918" "ENSGACG00000012708" "ENSGACG00000016925"
## [15328] "ENSGACG00000016929" "ENSGACG00000012774" "ENSGACG00000012795"
## [15331] "ENSGACG00000012831" "ENSGACG00000012836" "ENSGACG00000012842"
## [15334] "ENSGACG00000012847" "ENSGACG00000012849" "ENSGACG00000023558"
## [15337] "ENSGACG00000012863" "ENSGACG00000012867" "ENSGACG00000012878"
## [15340] "ENSGACG00000012882" "ENSGACG00000012897" "ENSGACG00000012901"
## [15343] "ENSGACG00000012919" "ENSGACG00000015397" "ENSGACG00000015407"
## [15346] "ENSGACG00000015410" "ENSGACG00000015416" "ENSGACG00000015418"
## [15349] "ENSGACG00000015425" "ENSGACG00000015426" "ENSGACG00000015442"
## [15352] "ENSGACG00000015444" "ENSGACG00000015451" "ENSGACG00000016935"
## [15355] "ENSGACG00000015455" "ENSGACG00000016939" "ENSGACG00000025344"
## [15358] "ENSGACG00000025768" "ENSGACG00000015468" "ENSGACG00000016942"
## [15361] "ENSGACG00000016948" "ENSGACG00000016956" "ENSGACG00000016970"
## [15364] "ENSGACG00000016975" "ENSGACG00000016976" "ENSGACG00000016985"
## [15367] "ENSGACG00000016988" "ENSGACG00000034489" "ENSGACG00000026758"
## [15370] "ENSGACG00000017011" "ENSGACG00000023574" "ENSGACG00000024040"
## [15373] "ENSGACG00000017023" "ENSGACG00000017025" "ENSGACG00000017028"
## [15376] "ENSGACG00000017043" "ENSGACG00000017045" "ENSGACG00000015471"
## [15379] "ENSGACG00000015473" "ENSGACG00000015477" "ENSGACG00000015479"
## [15382] "ENSGACG00000015487" "ENSGACG00000015496" "ENSGACG00000017050"
## [15385] "ENSGACG00000015501" "ENSGACG00000015504" "ENSGACG00000017052"
## [15388] "ENSGACG00000017054" "ENSGACG00000017055" "ENSGACG00000017058"
## [15391] "ENSGACG00000017065" "ENSGACG00000017071" "ENSGACG00000028136"
## [15394] "ENSGACG00000015509" "ENSGACG00000015513" "ENSGACG00000015515"
## [15397] "ENSGACG00000017084" "ENSGACG00000017089" "ENSGACG00000017090"
## [15400] "ENSGACG00000017095" "ENSGACG00000015519" "ENSGACG00000015520"
## [15403] "ENSGACG00000015523" "ENSGACG00000015531" "ENSGACG00000036566"
## [15406] "ENSGACG00000015538" "ENSGACG00000015549" "ENSGACG00000036383"
## [15409] "ENSGACG00000017104" "ENSGACG00000015554" "ENSGACG00000017109"
## [15412] "ENSGACG00000032365" "ENSGACG00000017118" "ENSGACG00000030514"
## [15415] "ENSGACG00000017140" "ENSGACG00000015561" "ENSGACG00000033464"
## [15418] "ENSGACG00000015568" "ENSGACG00000017151" "ENSGACG00000015571"
## [15421] "ENSGACG00000017173" "ENSGACG00000015576" "ENSGACG00000017176"
## [15424] "ENSGACG00000017177" "ENSGACG00000017179" "ENSGACG00000015580"
## [15427] "ENSGACG00000015582" "ENSGACG00000015583" "ENSGACG00000015588"
## [15430] "ENSGACG00000017595" "ENSGACG00000015590" "ENSGACG00000027552"
## [15433] "ENSGACG00000016395" "ENSGACG00000016417" "ENSGACG00000016434"
## [15436] "ENSGACG00000016435" "ENSGACG00000037360" "ENSGACG00000015596"
## [15439] "ENSGACG00000015597" "ENSGACG00000016439" "ENSGACG00000015601"
## [15442] "ENSGACG00000015606" "ENSGACG00000015607" "ENSGACG00000024613"
## [15445] "ENSGACG00000015613" "ENSGACG00000015618" "ENSGACG00000015621"
## [15448] "ENSGACG00000030202" "ENSGACG00000015631" "ENSGACG00000015634"
## [15451] "ENSGACG00000016450" "ENSGACG00000016454" "ENSGACG00000016461"
## [15454] "ENSGACG00000028783" "ENSGACG00000016466" "ENSGACG00000016468"
## [15457] "ENSGACG00000016469" "ENSGACG00000016471" "ENSGACG00000016489"
## [15460] "ENSGACG00000016497" "ENSGACG00000016522" "ENSGACG00000016529"
## [15463] "ENSGACG00000016533" "ENSGACG00000015662" "ENSGACG00000016538"
## [15466] "ENSGACG00000015665" "ENSGACG00000034713" "ENSGACG00000016546"
## [15469] "ENSGACG00000016551" "ENSGACG00000016560" "ENSGACG00000015670"
## [15472] "ENSGACG00000016563" "ENSGACG00000016567" "ENSGACG00000016570"
## [15475] "ENSGACG00000016576" "ENSGACG00000015674" "ENSGACG00000016582"
## [15478] "ENSGACG00000015678" "ENSGACG00000015682" "ENSGACG00000016586"
## [15481] "ENSGACG00000015684" "ENSGACG00000015686" "ENSGACG00000016672"
## [15484] "ENSGACG00000015689" "ENSGACG00000016674" "ENSGACG00000015692"
## [15487] "ENSGACG00000015695" "ENSGACG00000015696" "ENSGACG00000015702"
## [15490] "ENSGACG00000026063" "ENSGACG00000015713" "ENSGACG00000016654"
## [15493] "ENSGACG00000031756" "ENSGACG00000016659" "ENSGACG00000015720"
## [15496] "ENSGACG00000016662" "ENSGACG00000015721" "ENSGACG00000037503"
## [15499] "ENSGACG00000016671" "ENSGACG00000016595" "ENSGACG00000015723"
## [15502] "ENSGACG00000016597" "ENSGACG00000015732" "ENSGACG00000015735"
## [15505] "ENSGACG00000015743" "ENSGACG00000016599" "ENSGACG00000035095"
## [15508] "ENSGACG00000015748" "ENSGACG00000016602" "ENSGACG00000015750"
## [15511] "ENSGACG00000015760" "ENSGACG00000025648" "ENSGACG00000016605"
## [15514] "ENSGACG00000015764" "ENSGACG00000015969" "ENSGACG00000015981"
## [15517] "ENSGACG00000015985" "ENSGACG00000028286" "ENSGACG00000015994"
## [15520] "ENSGACG00000015998" "ENSGACG00000033530" "ENSGACG00000026480"
## [15523] "ENSGACG00000027257" "ENSGACG00000024623" "ENSGACG00000023075"
## [15526] "ENSGACG00000016010" "ENSGACG00000031098" "ENSGACG00000016612"
## [15529] "ENSGACG00000016028" "ENSGACG00000016031" "ENSGACG00000016618"
## [15532] "ENSGACG00000016039" "ENSGACG00000016625" "ENSGACG00000016043"
## [15535] "ENSGACG00000016053" "ENSGACG00000024225" "ENSGACG00000016066"
## [15538] "ENSGACG00000016397" "ENSGACG00000016401" "ENSGACG00000016406"
## [15541] "ENSGACG00000028401" "ENSGACG00000031477" "ENSGACG00000037620"
## [15544] "ENSGACG00000032360" "ENSGACG00000023426" "ENSGACG00000031131"
## [15547] "ENSGACG00000034871" "ENSGACG00000032811" "ENSGACG00000016414"
## [15550] "ENSGACG00000032096" "ENSGACG00000024411" "ENSGACG00000035843"
## [15553] "ENSGACG00000030396" "ENSGACG00000030208" "ENSGACG00000014113"
## [15556] "ENSGACG00000023258" "ENSGACG00000026859" "ENSGACG00000033648"
## [15559] "ENSGACG00000024926" "ENSGACG00000002564" "ENSGACG00000024662"
## [15562] "ENSGACG00000031669" "ENSGACG00000014469" "ENSGACG00000037617"
## [15565] "ENSGACG00000035036" "ENSGACG00000029644" "ENSGACG00000033977"
## [15568] "ENSGACG00000030263" "ENSGACG00000026748" "ENSGACG00000031034"
## [15571] "ENSGACG00000026355" "ENSGACG00000016343" "ENSGACG00000031603"
## [15574] "ENSGACG00000026610" "ENSGACG00000032214" "ENSGACG00000036484"
## [15577] "ENSGACG00000026708" "ENSGACG00000016348" "ENSGACG00000037547"
## [15580] "ENSGACG00000031047" "ENSGACG00000036503" "ENSGACG00000016354"
## [15583] "ENSGACG00000023675" "ENSGACG00000026948" "ENSGACG00000032968"
## [15586] "ENSGACG00000032265" "ENSGACG00000029014" "ENSGACG00000032408"
## [15589] "ENSGACG00000033120" "ENSGACG00000030463" "ENSGACG00000016371"
## [15592] "ENSGACG00000034589" "ENSGACG00000016373" "ENSGACG00000023371"
## [15595] "ENSGACG00000029372" "ENSGACG00000028937" "ENSGACG00000028281"
## [15598] "ENSGACG00000032488" "ENSGACG00000031413" "ENSGACG00000034808"
## [15601] "ENSGACG00000033236" "ENSGACG00000010465" "ENSGACG00000016384"
## [15604] "ENSGACG00000016391" "ENSGACG00000016392" "ENSGACG00000014470"
## [15607] "ENSGACG00000014993" "ENSGACG00000016393" "ENSGACG00000014875"
## [15610] "ENSGACG00000023604" "ENSGACG00000014960" "ENSGACG00000036370"
## [15613] "ENSGACG00000017604" "ENSGACG00000016090" "ENSGACG00000016290"
## [15616] "ENSGACG00000012576" "ENSGACG00000015431" "ENSGACG00000030663"
## [15619] "ENSGACG00000017607" "ENSGACG00000017641" "ENSGACG00000017644"
## [15622] "ENSGACG00000017648" "ENSGACG00000017656" "ENSGACG00000034663"
## [15625] "ENSGACG00000017665" "ENSGACG00000017667" "ENSGACG00000017672"
## [15628] "ENSGACG00000035410" "ENSGACG00000017675" "ENSGACG00000023358"
## [15631] "ENSGACG00000017679" "ENSGACG00000017682" "ENSGACG00000017683"
## [15634] "ENSGACG00000017685" "ENSGACG00000024535" "ENSGACG00000017688"
## [15637] "ENSGACG00000000902" "ENSGACG00000000899" "ENSGACG00000017696"
## [15640] "ENSGACG00000017399" "ENSGACG00000037125" "ENSGACG00000023207"
## [15643] "ENSGACG00000029921" "ENSGACG00000037348" "ENSGACG00000017705"
## [15646] "ENSGACG00000024491" "ENSGACG00000030805" "ENSGACG00000032737"
## [15649] "ENSGACG00000036054" "ENSGACG00000029180" "ENSGACG00000017719"
## [15652] "ENSGACG00000025175" "ENSGACG00000017724" "ENSGACG00000023597"
## [15655] "ENSGACG00000017730" "ENSGACG00000017738" "ENSGACG00000017751"
## [15658] "ENSGACG00000017762" "ENSGACG00000030316" "ENSGACG00000017770"
## [15661] "ENSGACG00000017772" "ENSGACG00000026845" "ENSGACG00000037477"
## [15664] "ENSGACG00000017787" "ENSGACG00000033585" "ENSGACG00000038041"
## [15667] "ENSGACG00000034408" "ENSGACG00000017794" "ENSGACG00000017799"
## [15670] "ENSGACG00000017811" "ENSGACG00000017184" "ENSGACG00000017188"
## [15673] "ENSGACG00000017191" "ENSGACG00000017194" "ENSGACG00000017196"
## [15676] "ENSGACG00000017198" "ENSGACG00000017202" "ENSGACG00000034124"
## [15679] "ENSGACG00000017207" "ENSGACG00000017210" "ENSGACG00000017213"
## [15682] "ENSGACG00000017215" "ENSGACG00000017220" "ENSGACG00000017224"
## [15685] "ENSGACG00000017229" "ENSGACG00000017235" "ENSGACG00000032219"
## [15688] "ENSGACG00000017244" "ENSGACG00000017269" "ENSGACG00000031761"
## [15691] "ENSGACG00000017272" "ENSGACG00000017280" "ENSGACG00000017282"
## [15694] "ENSGACG00000017287" "ENSGACG00000017289" "ENSGACG00000017295"
## [15697] "ENSGACG00000017298" "ENSGACG00000017309" "ENSGACG00000017314"
## [15700] "ENSGACG00000017323" "ENSGACG00000017327" "ENSGACG00000017336"
## [15703] "ENSGACG00000017361" "ENSGACG00000017383" "ENSGACG00000017393"
## [15706] "ENSGACG00000030768" "ENSGACG00000023283" "ENSGACG00000025171"
## [15709] "ENSGACG00000017395" "ENSGACG00000017400" "ENSGACG00000017402"
## [15712] "ENSGACG00000033499" "ENSGACG00000017406" "ENSGACG00000034041"
## [15715] "ENSGACG00000025009" "ENSGACG00000032405" "ENSGACG00000032404"
## [15718] "ENSGACG00000017421" "ENSGACG00000017425" "ENSGACG00000017427"
## [15721] "ENSGACG00000017431" "ENSGACG00000023121" "ENSGACG00000017437"
## [15724] "ENSGACG00000025196" "ENSGACG00000037756" "ENSGACG00000017535"
## [15727] "ENSGACG00000017451" "ENSGACG00000036193" "ENSGACG00000017477"
## [15730] "ENSGACG00000017480" "ENSGACG00000017481" "ENSGACG00000017540"
## [15733] "ENSGACG00000017546" "ENSGACG00000017549" "ENSGACG00000017553"
## [15736] "ENSGACG00000017568" "ENSGACG00000032055" "ENSGACG00000017561"
## [15739] "ENSGACG00000017565" "ENSGACG00000017575" "ENSGACG00000017582"
## [15742] "ENSGACG00000030689" "ENSGACG00000017584" "ENSGACG00000017590"
## [15745] "ENSGACG00000001580" "ENSGACG00000001584" "ENSGACG00000001615"
## [15748] "ENSGACG00000001619" "ENSGACG00000017812" "ENSGACG00000017816"
## [15751] "ENSGACG00000036436" "ENSGACG00000017821" "ENSGACG00000017828"
## [15754] "ENSGACG00000017835" "ENSGACG00000017834" "ENSGACG00000017842"
## [15757] "ENSGACG00000017847" "ENSGACG00000017854" "ENSGACG00000017855"
## [15760] "ENSGACG00000017857" "ENSGACG00000028983" "ENSGACG00000017893"
## [15763] "ENSGACG00000017904" "ENSGACG00000031531" "ENSGACG00000024277"
## [15766] "ENSGACG00000017916" "ENSGACG00000017926" "ENSGACG00000017927"
## [15769] "ENSGACG00000017929" "ENSGACG00000017935" "ENSGACG00000017931"
## [15772] "ENSGACG00000017936" "ENSGACG00000037312" "ENSGACG00000017947"
## [15775] "ENSGACG00000017953" "ENSGACG00000017957" "ENSGACG00000017959"
## [15778] "ENSGACG00000017961" "ENSGACG00000025748" "ENSGACG00000017974"
## [15781] "ENSGACG00000017977" "ENSGACG00000017975" "ENSGACG00000017983"
## [15784] "ENSGACG00000017984" "ENSGACG00000035232" "ENSGACG00000017989"
## [15787] "ENSGACG00000017991" "ENSGACG00000036921" "ENSGACG00000037509"
## [15790] "ENSGACG00000018006" "ENSGACG00000029074" "ENSGACG00000023300"
## [15793] "ENSGACG00000018008" "ENSGACG00000015815" "ENSGACG00000036259"
## [15796] "ENSGACG00000015812" "ENSGACG00000034534" "ENSGACG00000024787"
## [15799] "ENSGACG00000024343" "ENSGACG00000015797" "ENSGACG00000015794"
## [15802] "ENSGACG00000015765" "ENSGACG00000033957" "ENSGACG00000029830"
## [15805] "ENSGACG00000015769" "ENSGACG00000001634" "ENSGACG00000033661"
## [15808] "ENSGACG00000001675" "ENSGACG00000034469" "ENSGACG00000001621"
## [15811] "ENSGACG00000025336" "ENSGACG00000025286" "ENSGACG00000001587"
## [15814] "ENSGACG00000001583" "ENSGACG00000001571" "ENSGACG00000023668"
## [15817] "ENSGACG00000027214" "ENSGACG00000026415" "ENSGACG00000028543"
## [15820] "ENSGACG00000001529" "ENSGACG00000001525" "ENSGACG00000001527"
## [15823] "ENSGACG00000001524" "ENSGACG00000001523" "ENSGACG00000029714"
## [15826] "ENSGACG00000001504" "ENSGACG00000001499" "ENSGACG00000001496"
## [15829] "ENSGACG00000001484" "ENSGACG00000001481" "ENSGACG00000002441"
## [15832] "ENSGACG00000034809" "ENSGACG00000002450" "ENSGACG00000002454"
## [15835] "ENSGACG00000002459" "ENSGACG00000002517" "ENSGACG00000002472"
## [15838] "ENSGACG00000002524" "ENSGACG00000002523" "ENSGACG00000029530"
## [15841] "ENSGACG00000033050" "ENSGACG00000032221" "ENSGACG00000028403"
## [15844] "ENSGACG00000032314" "ENSGACG00000036258" "ENSGACG00000023487"
## [15847] "ENSGACG00000028263" "ENSGACG00000036604" "ENSGACG00000031384"
## [15850] "ENSGACG00000024921" "ENSGACG00000037472" "ENSGACG00000037048"
## [15853] "ENSGACG00000030183" "ENSGACG00000023309" "ENSGACG00000033309"
## [15856] "ENSGACG00000037353" "ENSGACG00000026317" "ENSGACG00000026163"
## [15859] "ENSGACG00000030060" "ENSGACG00000027252" "ENSGACG00000026521"
## [15862] "ENSGACG00000034374" "ENSGACG00000028042" "ENSGACG00000036577"
## [15865] "ENSGACG00000037715" "ENSGACG00000027513" "ENSGACG00000036638"
## [15868] "ENSGACG00000034441" "ENSGACG00000036927" "ENSGACG00000035093"
## [15871] "ENSGACG00000023852" "ENSGACG00000035000" "ENSGACG00000028991"
## [15874] "ENSGACG00000016532" "ENSGACG00000035164" "ENSGACG00000022982"
## [15877] "ENSGACG00000017260" "ENSGACG00000017446" "ENSGACG00000035782"
## [15880] "ENSGACG00000033056" "ENSGACG00000002496" "ENSGACG00000014057"
## [15883] "ENSGACG00000013429" "ENSGACG00000013434" "ENSGACG00000027280"
## [15886] "ENSGACG00000034339" "ENSGACG00000013462" "ENSGACG00000014990"
## [15889] "ENSGACG00000014079" "ENSGACG00000034404" "ENSGACG00000014910"
## [15892] "ENSGACG00000023315" "ENSGACG00000014903" "ENSGACG00000014088"
## [15895] "ENSGACG00000037374" "ENSGACG00000035978" "ENSGACG00000013420"
## [15898] "ENSGACG00000012084" "ENSGACG00000013472" "ENSGACG00000014987"
## [15901] "ENSGACG00000014982" "ENSGACG00000014979" "ENSGACG00000014967"
## [15904] "ENSGACG00000014959" "ENSGACG00000019207" "ENSGACG00000037698"
## [15907] "ENSGACG00000014954" "ENSGACG00000014952" "ENSGACG00000014943"
## [15910] "ENSGACG00000014940" "ENSGACG00000014938" "ENSGACG00000036798"
## [15913] "ENSGACG00000014936" "ENSGACG00000014935" "ENSGACG00000014930"
## [15916] "ENSGACG00000014927" "ENSGACG00000014924" "ENSGACG00000014082"
## [15919] "ENSGACG00000031306" "ENSGACG00000013490" "ENSGACG00000013506"
## [15922] "ENSGACG00000027317" "ENSGACG00000027524" "ENSGACG00000014005"
## [15925] "ENSGACG00000029509" "ENSGACG00000014010" "ENSGACG00000023593"
## [15928] "ENSGACG00000025962" "ENSGACG00000036722" "ENSGACG00000014024"
## [15931] "ENSGACG00000014033" "ENSGACG00000014038" "ENSGACG00000025300"
## [15934] "ENSGACG00000014047" "ENSGACG00000036454" "ENSGACG00000013694"
## [15937] "ENSGACG00000011884" "ENSGACG00000025421" "ENSGACG00000033180"
## [15940] "ENSGACG00000011890" "ENSGACG00000011899" "ENSGACG00000011905"
## [15943] "ENSGACG00000011960" "ENSGACG00000028151" "ENSGACG00000012005"
## [15946] "ENSGACG00000012011" "ENSGACG00000012023" "ENSGACG00000012035"
## [15949] "ENSGACG00000012064" "ENSGACG00000035094" "ENSGACG00000012082"
## [15952] "ENSGACG00000012174" "ENSGACG00000012178" "ENSGACG00000012190"
## [15955] "ENSGACG00000012196" "ENSGACG00000012203" "ENSGACG00000012215"
## [15958] "ENSGACG00000012219" "ENSGACG00000012238" "ENSGACG00000032434"
## [15961] "ENSGACG00000032959" "ENSGACG00000012269" "ENSGACG00000012276"
## [15964] "ENSGACG00000012291" "ENSGACG00000030131" "ENSGACG00000024657"
## [15967] "ENSGACG00000012304" "ENSGACG00000027784" "ENSGACG00000012319"
## [15970] "ENSGACG00000026190" "ENSGACG00000012320" "ENSGACG00000012357"
## [15973] "ENSGACG00000012363" "ENSGACG00000012403" "ENSGACG00000032947"
## [15976] "ENSGACG00000012409" "ENSGACG00000012422" "ENSGACG00000012429"
## [15979] "ENSGACG00000012435" "ENSGACG00000012437" "ENSGACG00000012449"
## [15982] "ENSGACG00000012455" "ENSGACG00000012460" "ENSGACG00000032543"
## [15985] "ENSGACG00000012472" "ENSGACG00000031691" "ENSGACG00000012535"
## [15988] "ENSGACG00000033424" "ENSGACG00000032350" "ENSGACG00000001322"
## [15991] "ENSGACG00000012556" "ENSGACG00000012567" "ENSGACG00000034751"
## [15994] "ENSGACG00000012587" "ENSGACG00000012593" "ENSGACG00000012598"
## [15997] "ENSGACG00000034112" "ENSGACG00000024556" "ENSGACG00000012617"
## [16000] "ENSGACG00000012637" "ENSGACG00000012096" "ENSGACG00000028577"
## [16003] "ENSGACG00000012103" "ENSGACG00000035746" "ENSGACG00000012140"
## [16006] "ENSGACG00000013508" "ENSGACG00000027929" "ENSGACG00000029193"
## [16009] "ENSGACG00000013529" "ENSGACG00000013555" "ENSGACG00000013570"
## [16012] "ENSGACG00000013573" "ENSGACG00000027113" "ENSGACG00000013584"
## [16015] "ENSGACG00000034755" "ENSGACG00000034970" "ENSGACG00000013602"
## [16018] "ENSGACG00000013608" "ENSGACG00000033408" "ENSGACG00000013628"
## [16021] "ENSGACG00000031240" "ENSGACG00000013640" "ENSGACG00000032102"
## [16024] "ENSGACG00000013673" "ENSGACG00000013688" "ENSGACG00000028535"
## [16027] "ENSGACG00000011503" "ENSGACG00000011523" "ENSGACG00000011526"
## [16030] "ENSGACG00000011530" "ENSGACG00000026230" "ENSGACG00000011541"
## [16033] "ENSGACG00000011550" "ENSGACG00000027438" "ENSGACG00000011563"
## [16036] "ENSGACG00000011578" "ENSGACG00000011592" "ENSGACG00000011601"
## [16039] "ENSGACG00000035120" "ENSGACG00000011632" "ENSGACG00000011643"
## [16042] "ENSGACG00000011652" "ENSGACG00000034710" "ENSGACG00000011694"
## [16045] "ENSGACG00000011696" "ENSGACG00000011700" "ENSGACG00000011706"
## [16048] "ENSGACG00000011709" "ENSGACG00000011720" "ENSGACG00000011731"
## [16051] "ENSGACG00000031753" "ENSGACG00000011749" "ENSGACG00000011762"
## [16054] "ENSGACG00000011775" "ENSGACG00000011800" "ENSGACG00000011808"
## [16057] "ENSGACG00000011814" "ENSGACG00000011858" "ENSGACG00000033025"
## [16060] "ENSGACG00000013723" "ENSGACG00000025465" "ENSGACG00000013727"
## [16063] "ENSGACG00000013741" "ENSGACG00000034302" "ENSGACG00000013766"
## [16066] "ENSGACG00000013770" "ENSGACG00000031569" "ENSGACG00000013773"
## [16069] "ENSGACG00000013787" "ENSGACG00000013818" "ENSGACG00000013825"
## [16072] "ENSGACG00000013853" "ENSGACG00000013839" "ENSGACG00000026309"
## [16075] "ENSGACG00000030424" "ENSGACG00000013930" "ENSGACG00000036794"
## [16078] "ENSGACG00000013948" "ENSGACG00000013960" "ENSGACG00000013969"
## [16081] "ENSGACG00000028443" "ENSGACG00000025403" "ENSGACG00000013988"
## [16084] "ENSGACG00000013991" "ENSGACG00000013993" "ENSGACG00000033036"
## [16087] "ENSGACG00000026866" "ENSGACG00000011408" "ENSGACG00000011412"
## [16090] "ENSGACG00000027996" "ENSGACG00000036733" "ENSGACG00000011433"
## [16093] "ENSGACG00000025511" "ENSGACG00000011440" "ENSGACG00000011445"
## [16096] "ENSGACG00000026181" "ENSGACG00000014891" "ENSGACG00000031460"
## [16099] "ENSGACG00000014885" "ENSGACG00000014884" "ENSGACG00000014882"
## [16102] "ENSGACG00000026523" "ENSGACG00000028929" "ENSGACG00000024898"
## [16105] "ENSGACG00000014872" "ENSGACG00000014866" "ENSGACG00000014865"
## [16108] "ENSGACG00000014861" "ENSGACG00000035448" "ENSGACG00000014840"
## [16111] "ENSGACG00000014837" "ENSGACG00000023304" "ENSGACG00000014997"
## [16114] "ENSGACG00000024783" "ENSGACG00000015001" "ENSGACG00000027948"
## [16117] "ENSGACG00000015006" "ENSGACG00000026129" "ENSGACG00000015011"
## [16120] "ENSGACG00000015015" "ENSGACG00000015017" "ENSGACG00000015022"
## [16123] "ENSGACG00000015023" "ENSGACG00000015032" "ENSGACG00000012677"
## [16126] "ENSGACG00000032028" "ENSGACG00000012690" "ENSGACG00000012731"
## [16129] "ENSGACG00000012741" "ENSGACG00000029409" "ENSGACG00000012757"
## [16132] "ENSGACG00000027653" "ENSGACG00000012781" "ENSGACG00000012769"
## [16135] "ENSGACG00000032294" "ENSGACG00000012763" "ENSGACG00000025343"
## [16138] "ENSGACG00000027718" "ENSGACG00000012814" "ENSGACG00000028463"
## [16141] "ENSGACG00000012825" "ENSGACG00000012838" "ENSGACG00000012848"
## [16144] "ENSGACG00000028955" "ENSGACG00000036197" "ENSGACG00000012872"
## [16147] "ENSGACG00000012881" "ENSGACG00000012885" "ENSGACG00000012896"
## [16150] "ENSGACG00000012911" "ENSGACG00000012936" "ENSGACG00000012953"
## [16153] "ENSGACG00000012961" "ENSGACG00000012980" "ENSGACG00000034762"
## [16156] "ENSGACG00000035883" "ENSGACG00000012988" "ENSGACG00000012990"
## [16159] "ENSGACG00000012998" "ENSGACG00000013019" "ENSGACG00000037326"
## [16162] "ENSGACG00000030045" "ENSGACG00000033276" "ENSGACG00000013059"
## [16165] "ENSGACG00000023729" "ENSGACG00000034995" "ENSGACG00000013080"
## [16168] "ENSGACG00000013095" "ENSGACG00000013102" "ENSGACG00000013119"
## [16171] "ENSGACG00000013134" "ENSGACG00000013139" "ENSGACG00000013141"
## [16174] "ENSGACG00000023102" "ENSGACG00000013264" "ENSGACG00000013279"
## [16177] "ENSGACG00000013287" "ENSGACG00000013295" "ENSGACG00000027970"
## [16180] "ENSGACG00000030040" "ENSGACG00000013336" "ENSGACG00000013346"
## [16183] "ENSGACG00000013350" "ENSGACG00000026368" "ENSGACG00000013359"
## [16186] "ENSGACG00000011161" "ENSGACG00000031425" "ENSGACG00000011165"
## [16189] "ENSGACG00000011185" "ENSGACG00000025269" "ENSGACG00000011192"
## [16192] "ENSGACG00000036805" "ENSGACG00000011207" "ENSGACG00000011215"
## [16195] "ENSGACG00000011236" "ENSGACG00000011261" "ENSGACG00000030960"
## [16198] "ENSGACG00000011277" "ENSGACG00000011284" "ENSGACG00000011292"
## [16201] "ENSGACG00000011295" "ENSGACG00000029388" "ENSGACG00000011313"
## [16204] "ENSGACG00000031837" "ENSGACG00000011329" "ENSGACG00000035257"
## [16207] "ENSGACG00000011339" "ENSGACG00000011341" "ENSGACG00000011350"
## [16210] "ENSGACG00000011359" "ENSGACG00000031921" "ENSGACG00000027304"
## [16213] "ENSGACG00000011370" "ENSGACG00000035226" "ENSGACG00000037152"
## [16216] "ENSGACG00000014129" "ENSGACG00000014134" "ENSGACG00000014138"
## [16219] "ENSGACG00000014145" "ENSGACG00000014154" "ENSGACG00000014161"
## [16222] "ENSGACG00000014169" "ENSGACG00000025428" "ENSGACG00000014177"
## [16225] "ENSGACG00000023248" "ENSGACG00000025596" "ENSGACG00000014202"
## [16228] "ENSGACG00000014208" "ENSGACG00000014221" "ENSGACG00000014224"
## [16231] "ENSGACG00000014233" "ENSGACG00000014239" "ENSGACG00000014254"
## [16234] "ENSGACG00000014261" "ENSGACG00000028098" "ENSGACG00000033098"
## [16237] "ENSGACG00000014271" "ENSGACG00000014276" "ENSGACG00000030050"
## [16240] "ENSGACG00000014278" "ENSGACG00000014288" "ENSGACG00000014295"
## [16243] "ENSGACG00000014332" "ENSGACG00000014316" "ENSGACG00000014320"
## [16246] "ENSGACG00000014330" "ENSGACG00000031248" "ENSGACG00000036769"
## [16249] "ENSGACG00000014338" "ENSGACG00000014351" "ENSGACG00000014365"
## [16252] "ENSGACG00000033319" "ENSGACG00000033362" "ENSGACG00000014386"
## [16255] "ENSGACG00000029483" "ENSGACG00000014391" "ENSGACG00000038094"
## [16258] "ENSGACG00000025607" "ENSGACG00000012701" "ENSGACG00000012705"
## [16261] "ENSGACG00000012709" "ENSGACG00000030819" "ENSGACG00000012712"
## [16264] "ENSGACG00000012716" "ENSGACG00000012736" "ENSGACG00000012748"
## [16267] "ENSGACG00000032744" "ENSGACG00000012768" "ENSGACG00000012808"
## [16270] "ENSGACG00000014685" "ENSGACG00000014680" "ENSGACG00000014660"
## [16273] "ENSGACG00000014656" "ENSGACG00000014635" "ENSGACG00000025129"
## [16276] "ENSGACG00000014616" "ENSGACG00000014603" "ENSGACG00000014559"
## [16279] "ENSGACG00000037759" "ENSGACG00000014556" "ENSGACG00000014538"
## [16282] "ENSGACG00000014537" "ENSGACG00000014532" "ENSGACG00000014529"
## [16285] "ENSGACG00000014523" "ENSGACG00000014516" "ENSGACG00000032937"
## [16288] "ENSGACG00000030943" "ENSGACG00000014506" "ENSGACG00000014500"
## [16291] "ENSGACG00000014497" "ENSGACG00000026303" "ENSGACG00000031115"
## [16294] "ENSGACG00000014486" "ENSGACG00000014483" "ENSGACG00000037041"
## [16297] "ENSGACG00000014475" "ENSGACG00000029397" "ENSGACG00000014463"
## [16300] "ENSGACG00000014454" "ENSGACG00000014449" "ENSGACG00000032909"
## [16303] "ENSGACG00000031749" "ENSGACG00000014443" "ENSGACG00000014440"
## [16306] "ENSGACG00000014433" "ENSGACG00000026433" "ENSGACG00000014416"
## [16309] "ENSGACG00000014410" "ENSGACG00000014830" "ENSGACG00000014829"
## [16312] "ENSGACG00000014822" "ENSGACG00000014813" "ENSGACG00000014806"
## [16315] "ENSGACG00000028829" "ENSGACG00000014796" "ENSGACG00000014793"
## [16318] "ENSGACG00000014789" "ENSGACG00000014784" "ENSGACG00000014781"
## [16321] "ENSGACG00000014780" "ENSGACG00000031042" "ENSGACG00000014765"
## [16324] "ENSGACG00000014763" "ENSGACG00000036265" "ENSGACG00000014751"
## [16327] "ENSGACG00000014740" "ENSGACG00000025531" "ENSGACG00000014731"
## [16330] "ENSGACG00000037685" "ENSGACG00000014717" "ENSGACG00000026883"
## [16333] "ENSGACG00000032988" "ENSGACG00000014700" "ENSGACG00000014698"
## [16336] "ENSGACG00000014697" "ENSGACG00000014689" "ENSGACG00000014991"
## [16339] "ENSGACG00000025059" "ENSGACG00000011881" "ENSGACG00000026794"
## [16342] "ENSGACG00000024720" "ENSGACG00000029674" "ENSGACG00000024170"
## [16345] "ENSGACG00000030018" "ENSGACG00000031366" "ENSGACG00000032722"
## [16348] "ENSGACG00000026176" "ENSGACG00000024215" "ENSGACG00000035030"
## [16351] "ENSGACG00000037274" "ENSGACG00000025685" "ENSGACG00000035461"
## [16354] "ENSGACG00000033715" "ENSGACG00000030931" "ENSGACG00000024792"
## [16357] "ENSGACG00000025825" "ENSGACG00000026732" "ENSGACG00000022925"
## [16360] "ENSGACG00000027400" "ENSGACG00000026601" "ENSGACG00000030915"
## [16363] "ENSGACG00000034116" "ENSGACG00000035390" "ENSGACG00000030191"
## [16366] "ENSGACG00000023461" "ENSGACG00000027011" "ENSGACG00000034745"
## [16369] "ENSGACG00000031639" "ENSGACG00000026186" "ENSGACG00000028885"
## [16372] "ENSGACG00000037822" "ENSGACG00000026872" "ENSGACG00000037925"
## [16375] "ENSGACG00000023893" "ENSGACG00000023491" "ENSGACG00000024692"
## [16378] "ENSGACG00000029058" "ENSGACG00000037245" "ENSGACG00000033013"
## [16381] "ENSGACG00000023754" "ENSGACG00000028265" "ENSGACG00000027243"
## [16384] "ENSGACG00000036308" "ENSGACG00000033283" "ENSGACG00000027506"
## [16387] "ENSGACG00000023694" "ENSGACG00000026830" "ENSGACG00000031426"
## [16390] "ENSGACG00000035161" "ENSGACG00000026135" "ENSGACG00000027095"
## [16393] "ENSGACG00000026180" "ENSGACG00000022639" "ENSGACG00000033701"
## [16396] "ENSGACG00000028050" "ENSGACG00000033873" "ENSGACG00000011425"
## [16399] "ENSGACG00000024595" "ENSGACG00000012799" "ENSGACG00000012783"
## [16402] "ENSGACG00000033304" "ENSGACG00000011303" "ENSGACG00000014304"
## [16405] "ENSGACG00000025412" "ENSGACG00000014472" "ENSGACG00000014802"
## [16408] "ENSGACG00000010121" "ENSGACG00000010133" "ENSGACG00000010139"
## [16411] "ENSGACG00000010142" "ENSGACG00000033972" "ENSGACG00000010155"
## [16414] "ENSGACG00000010159" "ENSGACG00000010165" "ENSGACG00000010192"
## [16417] "ENSGACG00000026051" "ENSGACG00000010202" "ENSGACG00000023086"
## [16420] "ENSGACG00000010209" "ENSGACG00000010217" "ENSGACG00000010258"
## [16423] "ENSGACG00000010263" "ENSGACG00000010280" "ENSGACG00000010295"
## [16426] "ENSGACG00000010301" "ENSGACG00000010312" "ENSGACG00000010327"
## [16429] "ENSGACG00000005394" "ENSGACG00000005388" "ENSGACG00000005359"
## [16432] "ENSGACG00000005339" "ENSGACG00000005333" "ENSGACG00000005318"
## [16435] "ENSGACG00000024383" "ENSGACG00000005297" "ENSGACG00000005270"
## [16438] "ENSGACG00000005261" "ENSGACG00000037053" "ENSGACG00000005250"
## [16441] "ENSGACG00000005249" "ENSGACG00000005245" "ENSGACG00000031342"
## [16444] "ENSGACG00000005143" "ENSGACG00000026608" "ENSGACG00000037250"
## [16447] "ENSGACG00000023539" "ENSGACG00000031225" "ENSGACG00000026696"
## [16450] "ENSGACG00000005131" "ENSGACG00000036587" "ENSGACG00000005084"
## [16453] "ENSGACG00000005060" "ENSGACG00000005008" "ENSGACG00000005000"
## [16456] "ENSGACG00000026616" "ENSGACG00000005871" "ENSGACG00000027550"
## [16459] "ENSGACG00000005878" "ENSGACG00000005907" "ENSGACG00000031295"
## [16462] "ENSGACG00000005805" "ENSGACG00000023821" "ENSGACG00000005811"
## [16465] "ENSGACG00000005822" "ENSGACG00000005830" "ENSGACG00000005844"
## [16468] "ENSGACG00000010356" "ENSGACG00000010374" "ENSGACG00000010401"
## [16471] "ENSGACG00000009661" "ENSGACG00000009684" "ENSGACG00000037206"
## [16474] "ENSGACG00000037013" "ENSGACG00000009722" "ENSGACG00000009738"
## [16477] "ENSGACG00000009767" "ENSGACG00000009782" "ENSGACG00000032433"
## [16480] "ENSGACG00000009795" "ENSGACG00000035685" "ENSGACG00000009804"
## [16483] "ENSGACG00000009821" "ENSGACG00000009828" "ENSGACG00000009852"
## [16486] "ENSGACG00000009854" "ENSGACG00000009875" "ENSGACG00000009896"
## [16489] "ENSGACG00000009914" "ENSGACG00000009926" "ENSGACG00000009943"
## [16492] "ENSGACG00000009946" "ENSGACG00000009952" "ENSGACG00000034780"
## [16495] "ENSGACG00000009978" "ENSGACG00000030528" "ENSGACG00000037572"
## [16498] "ENSGACG00000036243" "ENSGACG00000010039" "ENSGACG00000035830"
## [16501] "ENSGACG00000010106" "ENSGACG00000005938" "ENSGACG00000005943"
## [16504] "ENSGACG00000005952" "ENSGACG00000005990" "ENSGACG00000034933"
## [16507] "ENSGACG00000006009" "ENSGACG00000006018" "ENSGACG00000006046"
## [16510] "ENSGACG00000006070" "ENSGACG00000005400" "ENSGACG00000004729"
## [16513] "ENSGACG00000004781" "ENSGACG00000004798" "ENSGACG00000004802"
## [16516] "ENSGACG00000024985" "ENSGACG00000004841" "ENSGACG00000004844"
## [16519] "ENSGACG00000004854" "ENSGACG00000004857" "ENSGACG00000004897"
## [16522] "ENSGACG00000004950" "ENSGACG00000004970" "ENSGACG00000005784"
## [16525] "ENSGACG00000005761" "ENSGACG00000032772" "ENSGACG00000005755"
## [16528] "ENSGACG00000026992" "ENSGACG00000005661" "ENSGACG00000025033"
## [16531] "ENSGACG00000005644" "ENSGACG00000024988" "ENSGACG00000036155"
## [16534] "ENSGACG00000005631" "ENSGACG00000005628" "ENSGACG00000005626"
## [16537] "ENSGACG00000034519" "ENSGACG00000034134" "ENSGACG00000032403"
## [16540] "ENSGACG00000024545" "ENSGACG00000005605" "ENSGACG00000005589"
## [16543] "ENSGACG00000005576" "ENSGACG00000005568" "ENSGACG00000005531"
## [16546] "ENSGACG00000005522" "ENSGACG00000005502" "ENSGACG00000028873"
## [16549] "ENSGACG00000005447" "ENSGACG00000005444" "ENSGACG00000005403"
## [16552] "ENSGACG00000008326" "ENSGACG00000037814" "ENSGACG00000008346"
## [16555] "ENSGACG00000008358" "ENSGACG00000008362" "ENSGACG00000008377"
## [16558] "ENSGACG00000008394" "ENSGACG00000035260" "ENSGACG00000008389"
## [16561] "ENSGACG00000035534" "ENSGACG00000029859" "ENSGACG00000032597"
## [16564] "ENSGACG00000009415" "ENSGACG00000009436" "ENSGACG00000009445"
## [16567] "ENSGACG00000009465" "ENSGACG00000009487" "ENSGACG00000009522"
## [16570] "ENSGACG00000023682" "ENSGACG00000009536" "ENSGACG00000034171"
## [16573] "ENSGACG00000009540" "ENSGACG00000006615" "ENSGACG00000006081"
## [16576] "ENSGACG00000024124" "ENSGACG00000006096" "ENSGACG00000006197"
## [16579] "ENSGACG00000006215" "ENSGACG00000027801" "ENSGACG00000006231"
## [16582] "ENSGACG00000024349" "ENSGACG00000006255" "ENSGACG00000023846"
## [16585] "ENSGACG00000006265" "ENSGACG00000006270" "ENSGACG00000006278"
## [16588] "ENSGACG00000006288" "ENSGACG00000006342" "ENSGACG00000006395"
## [16591] "ENSGACG00000024878" "ENSGACG00000027617" "ENSGACG00000025176"
## [16594] "ENSGACG00000030289" "ENSGACG00000006412" "ENSGACG00000029523"
## [16597] "ENSGACG00000006448" "ENSGACG00000006453" "ENSGACG00000006477"
## [16600] "ENSGACG00000006482" "ENSGACG00000006483" "ENSGACG00000006494"
## [16603] "ENSGACG00000006504" "ENSGACG00000006514" "ENSGACG00000009558"
## [16606] "ENSGACG00000006540" "ENSGACG00000031617" "ENSGACG00000009576"
## [16609] "ENSGACG00000034708" "ENSGACG00000009590" "ENSGACG00000009598"
## [16612] "ENSGACG00000009622" "ENSGACG00000006555" "ENSGACG00000006561"
## [16615] "ENSGACG00000006598" "ENSGACG00000028672" "ENSGACG00000006606"
## [16618] "ENSGACG00000028800" "ENSGACG00000029122" "ENSGACG00000009652"
## [16621] "ENSGACG00000009655" "ENSGACG00000033887" "ENSGACG00000006630"
## [16624] "ENSGACG00000006640" "ENSGACG00000025503" "ENSGACG00000006658"
## [16627] "ENSGACG00000006669" "ENSGACG00000033675" "ENSGACG00000006691"
## [16630] "ENSGACG00000006705" "ENSGACG00000006712" "ENSGACG00000006733"
## [16633] "ENSGACG00000024209" "ENSGACG00000006740" "ENSGACG00000006744"
## [16636] "ENSGACG00000006757" "ENSGACG00000006763" "ENSGACG00000006771"
## [16639] "ENSGACG00000006776" "ENSGACG00000006786" "ENSGACG00000033255"
## [16642] "ENSGACG00000026308" "ENSGACG00000036083" "ENSGACG00000006848"
## [16645] "ENSGACG00000006852" "ENSGACG00000006867" "ENSGACG00000006879"
## [16648] "ENSGACG00000030944" "ENSGACG00000006907" "ENSGACG00000006925"
## [16651] "ENSGACG00000007003" "ENSGACG00000007026" "ENSGACG00000007063"
## [16654] "ENSGACG00000007070" "ENSGACG00000007073" "ENSGACG00000007092"
## [16657] "ENSGACG00000007131" "ENSGACG00000024990" "ENSGACG00000007157"
## [16660] "ENSGACG00000025364" "ENSGACG00000030332" "ENSGACG00000007174"
## [16663] "ENSGACG00000007176" "ENSGACG00000024507" "ENSGACG00000022993"
## [16666] "ENSGACG00000025516" "ENSGACG00000007197" "ENSGACG00000034766"
## [16669] "ENSGACG00000033880" "ENSGACG00000007206" "ENSGACG00000028679"
## [16672] "ENSGACG00000007212" "ENSGACG00000007224" "ENSGACG00000007233"
## [16675] "ENSGACG00000036086" "ENSGACG00000007243" "ENSGACG00000007246"
## [16678] "ENSGACG00000007259" "ENSGACG00000030042" "ENSGACG00000007268"
## [16681] "ENSGACG00000007273" "ENSGACG00000023104" "ENSGACG00000030588"
## [16684] "ENSGACG00000007290" "ENSGACG00000007314" "ENSGACG00000026073"
## [16687] "ENSGACG00000007322" "ENSGACG00000025786" "ENSGACG00000007336"
## [16690] "ENSGACG00000007344" "ENSGACG00000007357" "ENSGACG00000007360"
## [16693] "ENSGACG00000007378" "ENSGACG00000007421" "ENSGACG00000007426"
## [16696] "ENSGACG00000007443" "ENSGACG00000024587" "ENSGACG00000030130"
## [16699] "ENSGACG00000007457" "ENSGACG00000034338" "ENSGACG00000028739"
## [16702] "ENSGACG00000007477" "ENSGACG00000030414" "ENSGACG00000007516"
## [16705] "ENSGACG00000007533" "ENSGACG00000007540" "ENSGACG00000007549"
## [16708] "ENSGACG00000007567" "ENSGACG00000030110" "ENSGACG00000007628"
## [16711] "ENSGACG00000007633" "ENSGACG00000007645" "ENSGACG00000007657"
## [16714] "ENSGACG00000007663" "ENSGACG00000007684" "ENSGACG00000007707"
## [16717] "ENSGACG00000007724" "ENSGACG00000007735" "ENSGACG00000007755"
## [16720] "ENSGACG00000007759" "ENSGACG00000007764" "ENSGACG00000007792"
## [16723] "ENSGACG00000007796" "ENSGACG00000007807" "ENSGACG00000007822"
## [16726] "ENSGACG00000007836" "ENSGACG00000007892" "ENSGACG00000029902"
## [16729] "ENSGACG00000007912" "ENSGACG00000007931" "ENSGACG00000007947"
## [16732] "ENSGACG00000007962" "ENSGACG00000007978" "ENSGACG00000007992"
## [16735] "ENSGACG00000029978" "ENSGACG00000030473" "ENSGACG00000008039"
## [16738] "ENSGACG00000029045" "ENSGACG00000008071" "ENSGACG00000008090"
## [16741] "ENSGACG00000024318" "ENSGACG00000008181" "ENSGACG00000008299"
## [16744] "ENSGACG00000008313" "ENSGACG00000025100" "ENSGACG00000026314"
## [16747] "ENSGACG00000008421" "ENSGACG00000008434" "ENSGACG00000037486"
## [16750] "ENSGACG00000008462" "ENSGACG00000008473" "ENSGACG00000008483"
## [16753] "ENSGACG00000008492" "ENSGACG00000023043" "ENSGACG00000024624"
## [16756] "ENSGACG00000024853" "ENSGACG00000008517" "ENSGACG00000008519"
## [16759] "ENSGACG00000032472" "ENSGACG00000008523" "ENSGACG00000008527"
## [16762] "ENSGACG00000008532" "ENSGACG00000008544" "ENSGACG00000008553"
## [16765] "ENSGACG00000008605" "ENSGACG00000008607" "ENSGACG00000008634"
## [16768] "ENSGACG00000008641" "ENSGACG00000031813" "ENSGACG00000008648"
## [16771] "ENSGACG00000008659" "ENSGACG00000008679" "ENSGACG00000008740"
## [16774] "ENSGACG00000008756" "ENSGACG00000008761" "ENSGACG00000008768"
## [16777] "ENSGACG00000008806" "ENSGACG00000008809" "ENSGACG00000008812"
## [16780] "ENSGACG00000008824" "ENSGACG00000008830" "ENSGACG00000008857"
## [16783] "ENSGACG00000008867" "ENSGACG00000008875" "ENSGACG00000008889"
## [16786] "ENSGACG00000008897" "ENSGACG00000008904" "ENSGACG00000032729"
## [16789] "ENSGACG00000033634" "ENSGACG00000026716" "ENSGACG00000008920"
## [16792] "ENSGACG00000008937" "ENSGACG00000024598" "ENSGACG00000037970"
## [16795] "ENSGACG00000008948" "ENSGACG00000009023" "ENSGACG00000009044"
## [16798] "ENSGACG00000009057" "ENSGACG00000027346" "ENSGACG00000009096"
## [16801] "ENSGACG00000025625" "ENSGACG00000009106" "ENSGACG00000035091"
## [16804] "ENSGACG00000036616" "ENSGACG00000027823" "ENSGACG00000009163"
## [16807] "ENSGACG00000035893" "ENSGACG00000009259" "ENSGACG00000009280"
## [16810] "ENSGACG00000009291" "ENSGACG00000009371" "ENSGACG00000030757"
## [16813] "ENSGACG00000009381" "ENSGACG00000028621" "ENSGACG00000035173"
## [16816] "ENSGACG00000000430" "ENSGACG00000000434" "ENSGACG00000030623"
## [16819] "ENSGACG00000000448" "ENSGACG00000000458" "ENSGACG00000000462"
## [16822] "ENSGACG00000000468" "ENSGACG00000000469" "ENSGACG00000000474"
## [16825] "ENSGACG00000000484" "ENSGACG00000000485" "ENSGACG00000024676"
## [16828] "ENSGACG00000000496" "ENSGACG00000000500" "ENSGACG00000000503"
## [16831] "ENSGACG00000000506" "ENSGACG00000000333" "ENSGACG00000000335"
## [16834] "ENSGACG00000004178" "ENSGACG00000004234" "ENSGACG00000004296"
## [16837] "ENSGACG00000004324" "ENSGACG00000004364" "ENSGACG00000004396"
## [16840] "ENSGACG00000004404" "ENSGACG00000004418" "ENSGACG00000004449"
## [16843] "ENSGACG00000004489" "ENSGACG00000004503" "ENSGACG00000004527"
## [16846] "ENSGACG00000004552" "ENSGACG00000004567" "ENSGACG00000004610"
## [16849] "ENSGACG00000004634" "ENSGACG00000004684" "ENSGACG00000004710"
## [16852] "ENSGACG00000010423" "ENSGACG00000010428" "ENSGACG00000023767"
## [16855] "ENSGACG00000010454" "ENSGACG00000010463" "ENSGACG00000033531"
## [16858] "ENSGACG00000010471" "ENSGACG00000010531" "ENSGACG00000010535"
## [16861] "ENSGACG00000032871" "ENSGACG00000010548" "ENSGACG00000010562"
## [16864] "ENSGACG00000010574" "ENSGACG00000010598" "ENSGACG00000010596"
## [16867] "ENSGACG00000035568" "ENSGACG00000010630" "ENSGACG00000026139"
## [16870] "ENSGACG00000028599" "ENSGACG00000010647" "ENSGACG00000010661"
## [16873] "ENSGACG00000010662" "ENSGACG00000010678" "ENSGACG00000010792"
## [16876] "ENSGACG00000031941" "ENSGACG00000010804" "ENSGACG00000032500"
## [16879] "ENSGACG00000026219" "ENSGACG00000027772" "ENSGACG00000010824"
## [16882] "ENSGACG00000010845" "ENSGACG00000010865" "ENSGACG00000010875"
## [16885] "ENSGACG00000037899" "ENSGACG00000026665" "ENSGACG00000029199"
## [16888] "ENSGACG00000030783" "ENSGACG00000037633" "ENSGACG00000035617"
## [16891] "ENSGACG00000010899" "ENSGACG00000032607" "ENSGACG00000010915"
## [16894] "ENSGACG00000010921" "ENSGACG00000010926" "ENSGACG00000010941"
## [16897] "ENSGACG00000029892" "ENSGACG00000010948" "ENSGACG00000010972"
## [16900] "ENSGACG00000010982" "ENSGACG00000010987" "ENSGACG00000010994"
## [16903] "ENSGACG00000011006" "ENSGACG00000011013" "ENSGACG00000034792"
## [16906] "ENSGACG00000011019" "ENSGACG00000011028" "ENSGACG00000011036"
## [16909] "ENSGACG00000011059" "ENSGACG00000011067" "ENSGACG00000011072"
## [16912] "ENSGACG00000011097" "ENSGACG00000011103" "ENSGACG00000011109"
## [16915] "ENSGACG00000011120" "ENSGACG00000024517" "ENSGACG00000011132"
## [16918] "ENSGACG00000036783" "ENSGACG00000034554" "ENSGACG00000031349"
## [16921] "ENSGACG00000031551" "ENSGACG00000038130" "ENSGACG00000033935"
## [16924] "ENSGACG00000036027" "ENSGACG00000026809" "ENSGACG00000037518"
## [16927] "ENSGACG00000035731" "ENSGACG00000034335" "ENSGACG00000026025"
## [16930] "ENSGACG00000037238" "ENSGACG00000032213" "ENSGACG00000026200"
## [16933] "ENSGACG00000027337" "ENSGACG00000028814" "ENSGACG00000036432"
## [16936] "ENSGACG00000033919" "ENSGACG00000024197" "ENSGACG00000027768"
## [16939] "ENSGACG00000033212" "ENSGACG00000028951" "ENSGACG00000026649"
## [16942] "ENSGACG00000025438" "ENSGACG00000029063" "ENSGACG00000026931"
## [16945] "ENSGACG00000031916" "ENSGACG00000032770" "ENSGACG00000026820"
## [16948] "ENSGACG00000026628" "ENSGACG00000029883" "ENSGACG00000033943"
## [16951] "ENSGACG00000035081" "ENSGACG00000029511" "ENSGACG00000025233"
## [16954] "ENSGACG00000036873" "ENSGACG00000023932" "ENSGACG00000033676"
## [16957] "ENSGACG00000031657" "ENSGACG00000029344" "ENSGACG00000032406"
## [16960] "ENSGACG00000028411" "ENSGACG00000029828" "ENSGACG00000028335"
## [16963] "ENSGACG00000023444" "ENSGACG00000038096" "ENSGACG00000026067"
## [16966] "ENSGACG00000030977" "ENSGACG00000035190" "ENSGACG00000032750"
## [16969] "ENSGACG00000028125" "ENSGACG00000034716" "ENSGACG00000034888"
## [16972] "ENSGACG00000028110" "ENSGACG00000025008" "ENSGACG00000023725"
## [16975] "ENSGACG00000030772" "ENSGACG00000033078" "ENSGACG00000033471"
## [16978] "ENSGACG00000023623" "ENSGACG00000022363" "ENSGACG00000034362"
## [16981] "ENSGACG00000036022" "ENSGACG00000005722" "ENSGACG00000006626"
## [16984] "ENSGACG00000037164" "ENSGACG00000008012" "ENSGACG00000033380"
## [16987] "ENSGACG00000000573" "ENSGACG00000000588" "ENSGACG00000000554"
## [16990] "ENSGACG00000000556" "ENSGACG00000000564" "ENSGACG00000000582"
## [16993] "ENSGACG00000027401" "ENSGACG00000001399" "ENSGACG00000000594"
## [16996] "ENSGACG00000034760" "ENSGACG00000023032" "ENSGACG00000003285"
## [16999] "ENSGACG00000029721" "ENSGACG00000003341" "ENSGACG00000003352"
## [17002] "ENSGACG00000027993" "ENSGACG00000003367" "ENSGACG00000025424"
## [17005] "ENSGACG00000003377" "ENSGACG00000003389" "ENSGACG00000003408"
## [17008] "ENSGACG00000026826" "ENSGACG00000003410" "ENSGACG00000003452"
## [17011] "ENSGACG00000003464" "ENSGACG00000003489" "ENSGACG00000003500"
## [17014] "ENSGACG00000003515" "ENSGACG00000003525" "ENSGACG00000003543"
## [17017] "ENSGACG00000026873" "ENSGACG00000002714" "ENSGACG00000003553"
## [17020] "ENSGACG00000023149" "ENSGACG00000025474" "ENSGACG00000034532"
## [17023] "ENSGACG00000003606" "ENSGACG00000003608" "ENSGACG00000029929"
## [17026] "ENSGACG00000003621" "ENSGACG00000003626" "ENSGACG00000026142"
## [17029] "ENSGACG00000003678" "ENSGACG00000026735" "ENSGACG00000003687"
## [17032] "ENSGACG00000003693" "ENSGACG00000003858" "ENSGACG00000003865"
## [17035] "ENSGACG00000032531" "ENSGACG00000037593" "ENSGACG00000003893"
## [17038] "ENSGACG00000003909" "ENSGACG00000003913" "ENSGACG00000003920"
## [17041] "ENSGACG00000003933" "ENSGACG00000003735" "ENSGACG00000003740"
## [17044] "ENSGACG00000022908" "ENSGACG00000003756" "ENSGACG00000003763"
## [17047] "ENSGACG00000003774" "ENSGACG00000029904" "ENSGACG00000003816"
## [17050] "ENSGACG00000003823" "ENSGACG00000003837" "ENSGACG00000003838"
## [17053] "ENSGACG00000036864" "ENSGACG00000030142" "ENSGACG00000036203"
## [17056] "ENSGACG00000029968" "ENSGACG00000002700" "ENSGACG00000002708"
## [17059] "ENSGACG00000002710" "ENSGACG00000005504" "ENSGACG00000027421"
## [17062] "ENSGACG00000005528" "ENSGACG00000005543" "ENSGACG00000005557"
## [17065] "ENSGACG00000034158" "ENSGACG00000032043" "ENSGACG00000005565"
## [17068] "ENSGACG00000005575" "ENSGACG00000034533" "ENSGACG00000034381"
## [17071] "ENSGACG00000034119" "ENSGACG00000031440" "ENSGACG00000032506"
## [17074] "ENSGACG00000012636" "ENSGACG00000002723" "ENSGACG00000002727"
## [17077] "ENSGACG00000002744" "ENSGACG00000002748" "ENSGACG00000002757"
## [17080] "ENSGACG00000002767" "ENSGACG00000002765" "ENSGACG00000031445"
## [17083] "ENSGACG00000032429" "ENSGACG00000002793" "ENSGACG00000002794"
## [17086] "ENSGACG00000002803" "ENSGACG00000037271" "ENSGACG00000002818"
## [17089] "ENSGACG00000002822" "ENSGACG00000033616" "ENSGACG00000002837"
## [17092] "ENSGACG00000002842" "ENSGACG00000002845" "ENSGACG00000002857"
## [17095] "ENSGACG00000002879" "ENSGACG00000002894" "ENSGACG00000002896"
## [17098] "ENSGACG00000002909" "ENSGACG00000002911" "ENSGACG00000002913"
## [17101] "ENSGACG00000002922" "ENSGACG00000002964" "ENSGACG00000002969"
## [17104] "ENSGACG00000002971" "ENSGACG00000002973" "ENSGACG00000002974"
## [17107] "ENSGACG00000002983" "ENSGACG00000002986" "ENSGACG00000002998"
## [17110] "ENSGACG00000003004" "ENSGACG00000003016" "ENSGACG00000003047"
## [17113] "ENSGACG00000003053" "ENSGACG00000003060" "ENSGACG00000003066"
## [17116] "ENSGACG00000003081" "ENSGACG00000003094" "ENSGACG00000003095"
## [17119] "ENSGACG00000026842" "ENSGACG00000003102" "ENSGACG00000003107"
## [17122] "ENSGACG00000003110" "ENSGACG00000003114" "ENSGACG00000036381"
## [17125] "ENSGACG00000003147" "ENSGACG00000003151" "ENSGACG00000032570"
## [17128] "ENSGACG00000003172" "ENSGACG00000034330" "ENSGACG00000003186"
## [17131] "ENSGACG00000033727" "ENSGACG00000035704" "ENSGACG00000003207"
## [17134] "ENSGACG00000003211" "ENSGACG00000003247" "ENSGACG00000003254"
## [17137] "ENSGACG00000003251" "ENSGACG00000003260" "ENSGACG00000003266"
## [17140] "ENSGACG00000003265" "ENSGACG00000037585" "ENSGACG00000012653"
## [17143] "ENSGACG00000027084" "ENSGACG00000012670" "ENSGACG00000031857"
## [17146] "ENSGACG00000012678" "ENSGACG00000028393" "ENSGACG00000012722"
## [17149] "ENSGACG00000012729" "ENSGACG00000012745" "ENSGACG00000012775"
## [17152] "ENSGACG00000012803" "ENSGACG00000012811" "ENSGACG00000012829"
## [17155] "ENSGACG00000012864" "ENSGACG00000012869" "ENSGACG00000036844"
## [17158] "ENSGACG00000012895" "ENSGACG00000034932" "ENSGACG00000030277"
## [17161] "ENSGACG00000001170" "ENSGACG00000001167" "ENSGACG00000030361"
## [17164] "ENSGACG00000001160" "ENSGACG00000030735" "ENSGACG00000001148"
## [17167] "ENSGACG00000033327" "ENSGACG00000001138" "ENSGACG00000029569"
## [17170] "ENSGACG00000018107" "ENSGACG00000018105" "ENSGACG00000018101"
## [17173] "ENSGACG00000018093" "ENSGACG00000024628" "ENSGACG00000018085"
## [17176] "ENSGACG00000018079" "ENSGACG00000003936" "ENSGACG00000003949"
## [17179] "ENSGACG00000004993" "ENSGACG00000023306" "ENSGACG00000004999"
## [17182] "ENSGACG00000005055" "ENSGACG00000023125" "ENSGACG00000005087"
## [17185] "ENSGACG00000005097" "ENSGACG00000029307" "ENSGACG00000005116"
## [17188] "ENSGACG00000005134" "ENSGACG00000005141" "ENSGACG00000005146"
## [17191] "ENSGACG00000005155" "ENSGACG00000005172" "ENSGACG00000035333"
## [17194] "ENSGACG00000005288" "ENSGACG00000031190" "ENSGACG00000023251"
## [17197] "ENSGACG00000005308" "ENSGACG00000005314" "ENSGACG00000005328"
## [17200] "ENSGACG00000005346" "ENSGACG00000026311" "ENSGACG00000029066"
## [17203] "ENSGACG00000005386" "ENSGACG00000024094" "ENSGACG00000002594"
## [17206] "ENSGACG00000002599" "ENSGACG00000002603" "ENSGACG00000002620"
## [17209] "ENSGACG00000002625" "ENSGACG00000002631" "ENSGACG00000002636"
## [17212] "ENSGACG00000002643" "ENSGACG00000002662" "ENSGACG00000002670"
## [17215] "ENSGACG00000034478" "ENSGACG00000002680" "ENSGACG00000002682"
## [17218] "ENSGACG00000002686" "ENSGACG00000024526" "ENSGACG00000003847"
## [17221] "ENSGACG00000003955" "ENSGACG00000003962" "ENSGACG00000005390"
## [17224] "ENSGACG00000005398" "ENSGACG00000005424" "ENSGACG00000005435"
## [17227] "ENSGACG00000005449" "ENSGACG00000005455" "ENSGACG00000005472"
## [17230] "ENSGACG00000005475" "ENSGACG00000005478" "ENSGACG00000005488"
## [17233] "ENSGACG00000005500" "ENSGACG00000003965" "ENSGACG00000003976"
## [17236] "ENSGACG00000038082" "ENSGACG00000003991" "ENSGACG00000003994"
## [17239] "ENSGACG00000004008" "ENSGACG00000004019" "ENSGACG00000004022"
## [17242] "ENSGACG00000004034" "ENSGACG00000004036" "ENSGACG00000033577"
## [17245] "ENSGACG00000004059" "ENSGACG00000004067" "ENSGACG00000004075"
## [17248] "ENSGACG00000004079" "ENSGACG00000004096" "ENSGACG00000004102"
## [17251] "ENSGACG00000004107" "ENSGACG00000004111" "ENSGACG00000004128"
## [17254] "ENSGACG00000004157" "ENSGACG00000004163" "ENSGACG00000004176"
## [17257] "ENSGACG00000004186" "ENSGACG00000004197" "ENSGACG00000004201"
## [17260] "ENSGACG00000004209" "ENSGACG00000004252" "ENSGACG00000004261"
## [17263] "ENSGACG00000004275" "ENSGACG00000004282" "ENSGACG00000004297"
## [17266] "ENSGACG00000004303" "ENSGACG00000004336" "ENSGACG00000004347"
## [17269] "ENSGACG00000031808" "ENSGACG00000028102" "ENSGACG00000004365"
## [17272] "ENSGACG00000004422" "ENSGACG00000031466" "ENSGACG00000032313"
## [17275] "ENSGACG00000004445" "ENSGACG00000024245" "ENSGACG00000004460"
## [17278] "ENSGACG00000004505" "ENSGACG00000004538" "ENSGACG00000004560"
## [17281] "ENSGACG00000004586" "ENSGACG00000004595" "ENSGACG00000004604"
## [17284] "ENSGACG00000031319" "ENSGACG00000004664" "ENSGACG00000030896"
## [17287] "ENSGACG00000026191" "ENSGACG00000023021" "ENSGACG00000034173"
## [17290] "ENSGACG00000004706" "ENSGACG00000004717" "ENSGACG00000004725"
## [17293] "ENSGACG00000004747" "ENSGACG00000004751" "ENSGACG00000030265"
## [17296] "ENSGACG00000004755" "ENSGACG00000004764" "ENSGACG00000004804"
## [17299] "ENSGACG00000004834" "ENSGACG00000004845" "ENSGACG00000031336"
## [17302] "ENSGACG00000004863" "ENSGACG00000004870" "ENSGACG00000004873"
## [17305] "ENSGACG00000004878" "ENSGACG00000004882" "ENSGACG00000004905"
## [17308] "ENSGACG00000036635" "ENSGACG00000029099" "ENSGACG00000004956"
## [17311] "ENSGACG00000004967" "ENSGACG00000004982" "ENSGACG00000004991"
## [17314] "ENSGACG00000005570" "ENSGACG00000028487" "ENSGACG00000036992"
## [17317] "ENSGACG00000030342" "ENSGACG00000030094" "ENSGACG00000027685"
## [17320] "ENSGACG00000031762" "ENSGACG00000024036" "ENSGACG00000024804"
## [17323] "ENSGACG00000035536" "ENSGACG00000029226" "ENSGACG00000036582"
## [17326] "ENSGACG00000024767" "ENSGACG00000024511" "ENSGACG00000034017"
## [17329] "ENSGACG00000023200" "ENSGACG00000034499" "ENSGACG00000029401"
## [17332] "ENSGACG00000030154" "ENSGACG00000028087" "ENSGACG00000025431"
## [17335] "ENSGACG00000030025" "ENSGACG00000033312" "ENSGACG00000030099"
## [17338] "ENSGACG00000035745" "ENSGACG00000023635" "ENSGACG00000036888"
## [17341] "ENSGACG00000029787" "ENSGACG00000033682" "ENSGACG00000032658"
## [17344] "ENSGACG00000026016" "ENSGACG00000036004" "ENSGACG00000022644"
## [17347] "ENSGACG00000031977" "ENSGACG00000031278" "ENSGACG00000002752"
## [17350] "ENSGACG00000002831" "ENSGACG00000029447" "ENSGACG00000032954"
## [17353] "ENSGACG00000005360" "ENSGACG00000004949" "ENSGACG00000024571"
## [17356] "ENSGACG00000002334" "ENSGACG00000030573" "ENSGACG00000002347"
## [17359] "ENSGACG00000002362" "ENSGACG00000002374" "ENSGACG00000002385"
## [17362] "ENSGACG00000002388" "ENSGACG00000002390" "ENSGACG00000031429"
## [17365] "ENSGACG00000002395" "ENSGACG00000023252" "ENSGACG00000002399"
## [17368] "ENSGACG00000030575" "ENSGACG00000002402" "ENSGACG00000002406"
## [17371] "ENSGACG00000002411" "ENSGACG00000033508" "ENSGACG00000002416"
## [17374] "ENSGACG00000002418" "ENSGACG00000002421" "ENSGACG00000030828"
## [17377] "ENSGACG00000002426" "ENSGACG00000002435" "ENSGACG00000031582"
## [17380] "ENSGACG00000002444" "ENSGACG00000002448" "ENSGACG00000028349"
## [17383] "ENSGACG00000002460" "ENSGACG00000002461" "ENSGACG00000001712"
## [17386] "ENSGACG00000001718" "ENSGACG00000025504" "ENSGACG00000024085"
## [17389] "ENSGACG00000031282" "ENSGACG00000001760" "ENSGACG00000033876"
## [17392] "ENSGACG00000002475" "ENSGACG00000002478" "ENSGACG00000002485"
## [17395] "ENSGACG00000002491" "ENSGACG00000002493" "ENSGACG00000002494"
## [17398] "ENSGACG00000002498" "ENSGACG00000002505" "ENSGACG00000028710"
## [17401] "ENSGACG00000031533" "ENSGACG00000002512" "ENSGACG00000002519"
## [17404] "ENSGACG00000002531" "ENSGACG00000002536" "ENSGACG00000002544"
## [17407] "ENSGACG00000002559" "ENSGACG00000035955" "ENSGACG00000002551"
## [17410] "ENSGACG00000002553" "ENSGACG00000030794" "ENSGACG00000016776"
## [17413] "ENSGACG00000002060" "ENSGACG00000002069" "ENSGACG00000002073"
## [17416] "ENSGACG00000002094" "ENSGACG00000030748" "ENSGACG00000002101"
## [17419] "ENSGACG00000002145" "ENSGACG00000002106" "ENSGACG00000002116"
## [17422] "ENSGACG00000002128" "ENSGACG00000032476" "ENSGACG00000002135"
## [17425] "ENSGACG00000031048" "ENSGACG00000001778" "ENSGACG00000001786"
## [17428] "ENSGACG00000001798" "ENSGACG00000001940" "ENSGACG00000001809"
## [17431] "ENSGACG00000024302" "ENSGACG00000001836" "ENSGACG00000001848"
## [17434] "ENSGACG00000001852" "ENSGACG00000001858" "ENSGACG00000001862"
## [17437] "ENSGACG00000026609" "ENSGACG00000001884" "ENSGACG00000001917"
## [17440] "ENSGACG00000028611" "ENSGACG00000001930" "ENSGACG00000001949"
## [17443] "ENSGACG00000001956" "ENSGACG00000001961" "ENSGACG00000001966"
## [17446] "ENSGACG00000001969" "ENSGACG00000033008" "ENSGACG00000002048"
## [17449] "ENSGACG00000001974" "ENSGACG00000001997" "ENSGACG00000002000"
## [17452] "ENSGACG00000002012" "ENSGACG00000028626" "ENSGACG00000002031"
## [17455] "ENSGACG00000023769" "ENSGACG00000002156" "ENSGACG00000002157"
## [17458] "ENSGACG00000002158" "ENSGACG00000002164" "ENSGACG00000028911"
## [17461] "ENSGACG00000002167" "ENSGACG00000024722" "ENSGACG00000002180"
## [17464] "ENSGACG00000002183" "ENSGACG00000034880" "ENSGACG00000002192"
## [17467] "ENSGACG00000002198" "ENSGACG00000035253" "ENSGACG00000029365"
## [17470] "ENSGACG00000023179" "ENSGACG00000035804" "ENSGACG00000035776"
## [17473] "ENSGACG00000035174" "ENSGACG00000002244" "ENSGACG00000035265"
## [17476] "ENSGACG00000028604" "ENSGACG00000027203" "ENSGACG00000036890"
## [17479] "ENSGACG00000002268" "ENSGACG00000002276" "ENSGACG00000028914"
## [17482] "ENSGACG00000002286" "ENSGACG00000002307" "ENSGACG00000002309"
## [17485] "ENSGACG00000002321" "ENSGACG00000028437" "ENSGACG00000031672"
## [17488] "ENSGACG00000002326" "ENSGACG00000030116" "ENSGACG00000037039"
## [17491] "ENSGACG00000024444" "ENSGACG00000029583" "ENSGACG00000001114"
## [17494] "ENSGACG00000034615" "ENSGACG00000000078" "ENSGACG00000000087"
## [17497] "ENSGACG00000032048" "ENSGACG00000036056" "ENSGACG00000000100"
## [17500] "ENSGACG00000000102" "ENSGACG00000035298" "ENSGACG00000000104"
## [17503] "ENSGACG00000000105" "ENSGACG00000026718" "ENSGACG00000000111"
## [17506] "ENSGACG00000028344" "ENSGACG00000035930" "ENSGACG00000035326"
## [17509] "ENSGACG00000014769" "ENSGACG00000001652" "ENSGACG00000023888"
## [17512] "ENSGACG00000000861" "ENSGACG00000000864" "ENSGACG00000032439"
## [17515] "ENSGACG00000000879" "ENSGACG00000028424" "ENSGACG00000028467"
## [17518] "ENSGACG00000000895" "ENSGACG00000000903" "ENSGACG00000025099"
## [17521] "ENSGACG00000000933" "ENSGACG00000000934" "ENSGACG00000030466"
## [17524] "ENSGACG00000000940" "ENSGACG00000000951" "ENSGACG00000034697"
## [17527] "ENSGACG00000000965" "ENSGACG00000000970" "ENSGACG00000000972"
## [17530] "ENSGACG00000000975" "ENSGACG00000000976" "ENSGACG00000000979"
## [17533] "ENSGACG00000031909" "ENSGACG00000000987" "ENSGACG00000000999"
## [17536] "ENSGACG00000001002" "ENSGACG00000001006" "ENSGACG00000001018"
## [17539] "ENSGACG00000030671" "ENSGACG00000001024" "ENSGACG00000001050"
## [17542] "ENSGACG00000001055" "ENSGACG00000026406" "ENSGACG00000001069"
## [17545] "ENSGACG00000001656" "ENSGACG00000001658" "ENSGACG00000036095"
## [17548] "ENSGACG00000001662" "ENSGACG00000001666" "ENSGACG00000001679"
## [17551] "ENSGACG00000001687" "ENSGACG00000001691" "ENSGACG00000028567"
## [17554] "ENSGACG00000001700" "ENSGACG00000026240" "ENSGACG00000031458"
## [17557] "ENSGACG00000024212" "ENSGACG00000033938" "ENSGACG00000024980"
## [17560] "ENSGACG00000034253" "ENSGACG00000027923" "ENSGACG00000037583"
## [17563] "ENSGACG00000029986" "ENSGACG00000023838" "ENSGACG00000036822"
## [17566] "ENSGACG00000023881" "ENSGACG00000030881" "ENSGACG00000024647"
## [17569] "ENSGACG00000035295" "ENSGACG00000032844" "ENSGACG00000032916"
## [17572] "ENSGACG00000024010" "ENSGACG00000015250" "ENSGACG00000032905"
## [17575] "ENSGACG00000029219" "ENSGACG00000028966" "ENSGACG00000030624"
## [17578] "ENSGACG00000027053" "ENSGACG00000036718" "ENSGACG00000028304"
## [17581] "ENSGACG00000023511" "ENSGACG00000036809" "ENSGACG00000037037"
## [17584] "ENSGACG00000026134" "ENSGACG00000037001" "ENSGACG00000026273"
## [17587] "ENSGACG00000023401" "ENSGACG00000030869" "ENSGACG00000034852"
## [17590] "ENSGACG00000037407" "ENSGACG00000025139" "ENSGACG00000001722"
## [17593] "ENSGACG00000002095" "ENSGACG00000001871" "ENSGACG00000001881"
## [17596] "ENSGACG00000001958" "ENSGACG00000029715" "ENSGACG00000026869"
## [17599] "ENSGACG00000031662" "ENSGACG00000031340" "ENSGACG00000036934"
## [17602] "ENSGACG00000024986" "ENSGACG00000007390" "ENSGACG00000007403"
## [17605] "ENSGACG00000007409" "ENSGACG00000007455" "ENSGACG00000007467"
## [17608] "ENSGACG00000007487" "ENSGACG00000007497" "ENSGACG00000034052"
## [17611] "ENSGACG00000007509" "ENSGACG00000007538" "ENSGACG00000007542"
## [17614] "ENSGACG00000007548" "ENSGACG00000025881" "ENSGACG00000007981"
## [17617] "ENSGACG00000007988" "ENSGACG00000009245" "ENSGACG00000009254"
## [17620] "ENSGACG00000009262" "ENSGACG00000009279" "ENSGACG00000036756"
## [17623] "ENSGACG00000023818" "ENSGACG00000009310" "ENSGACG00000024496"
## [17626] "ENSGACG00000009386" "ENSGACG00000036429" "ENSGACG00000024314"
## [17629] "ENSGACG00000009406" "ENSGACG00000024739" "ENSGACG00000009448"
## [17632] "ENSGACG00000009458" "ENSGACG00000035282" "ENSGACG00000009466"
## [17635] "ENSGACG00000009473" "ENSGACG00000023938" "ENSGACG00000009488"
## [17638] "ENSGACG00000024142" "ENSGACG00000008275" "ENSGACG00000007418"
## [17641] "ENSGACG00000008836" "ENSGACG00000008282" "ENSGACG00000008294"
## [17644] "ENSGACG00000008300" "ENSGACG00000035966" "ENSGACG00000008322"
## [17647] "ENSGACG00000008360" "ENSGACG00000008383" "ENSGACG00000008396"
## [17650] "ENSGACG00000008408" "ENSGACG00000008424" "ENSGACG00000007869"
## [17653] "ENSGACG00000007896" "ENSGACG00000037448" "ENSGACG00000007915"
## [17656] "ENSGACG00000025794" "ENSGACG00000008845" "ENSGACG00000008849"
## [17659] "ENSGACG00000008913" "ENSGACG00000008923" "ENSGACG00000032184"
## [17662] "ENSGACG00000008929" "ENSGACG00000008941" "ENSGACG00000008955"
## [17665] "ENSGACG00000008968" "ENSGACG00000008998" "ENSGACG00000009030"
## [17668] "ENSGACG00000009031" "ENSGACG00000009046" "ENSGACG00000009050"
## [17671] "ENSGACG00000023434" "ENSGACG00000009073" "ENSGACG00000009087"
## [17674] "ENSGACG00000009094" "ENSGACG00000009124" "ENSGACG00000031067"
## [17677] "ENSGACG00000029525" "ENSGACG00000025234" "ENSGACG00000009179"
## [17680] "ENSGACG00000035201" "ENSGACG00000009194" "ENSGACG00000009199"
## [17683] "ENSGACG00000009209" "ENSGACG00000009229" "ENSGACG00000009232"
## [17686] "ENSGACG00000008151" "ENSGACG00000008158" "ENSGACG00000008184"
## [17689] "ENSGACG00000008202" "ENSGACG00000008210" "ENSGACG00000036961"
## [17692] "ENSGACG00000008260" "ENSGACG00000030653" "ENSGACG00000007580"
## [17695] "ENSGACG00000007553" "ENSGACG00000007595" "ENSGACG00000010588"
## [17698] "ENSGACG00000024122" "ENSGACG00000037020" "ENSGACG00000007623"
## [17701] "ENSGACG00000029107" "ENSGACG00000007632" "ENSGACG00000027223"
## [17704] "ENSGACG00000007648" "ENSGACG00000007688" "ENSGACG00000007712"
## [17707] "ENSGACG00000029358" "ENSGACG00000037161" "ENSGACG00000007731"
## [17710] "ENSGACG00000007746" "ENSGACG00000009501" "ENSGACG00000029476"
## [17713] "ENSGACG00000009509" "ENSGACG00000009525" "ENSGACG00000009559"
## [17716] "ENSGACG00000009567" "ENSGACG00000009575" "ENSGACG00000009603"
## [17719] "ENSGACG00000027725" "ENSGACG00000009620" "ENSGACG00000009650"
## [17722] "ENSGACG00000005199" "ENSGACG00000036321" "ENSGACG00000027681"
## [17725] "ENSGACG00000009679" "ENSGACG00000009709" "ENSGACG00000009755"
## [17728] "ENSGACG00000032612" "ENSGACG00000024026" "ENSGACG00000009787"
## [17731] "ENSGACG00000009807" "ENSGACG00000034854" "ENSGACG00000009810"
## [17734] "ENSGACG00000009819" "ENSGACG00000031755" "ENSGACG00000031923"
## [17737] "ENSGACG00000009858" "ENSGACG00000009899" "ENSGACG00000030507"
## [17740] "ENSGACG00000036580" "ENSGACG00000032205" "ENSGACG00000008430"
## [17743] "ENSGACG00000008443" "ENSGACG00000008432" "ENSGACG00000008470"
## [17746] "ENSGACG00000007763" "ENSGACG00000008488" "ENSGACG00000008506"
## [17749] "ENSGACG00000007769" "ENSGACG00000007781" "ENSGACG00000026124"
## [17752] "ENSGACG00000008525" "ENSGACG00000007827" "ENSGACG00000007840"
## [17755] "ENSGACG00000028141" "ENSGACG00000007852" "ENSGACG00000007855"
## [17758] "ENSGACG00000008533" "ENSGACG00000008542" "ENSGACG00000008573"
## [17761] "ENSGACG00000008597" "ENSGACG00000025906" "ENSGACG00000008621"
## [17764] "ENSGACG00000008639" "ENSGACG00000008649" "ENSGACG00000008656"
## [17767] "ENSGACG00000008666" "ENSGACG00000008704" "ENSGACG00000008719"
## [17770] "ENSGACG00000010260" "ENSGACG00000010272" "ENSGACG00000010331"
## [17773] "ENSGACG00000025280" "ENSGACG00000026712" "ENSGACG00000010368"
## [17776] "ENSGACG00000010552" "ENSGACG00000010557" "ENSGACG00000010534"
## [17779] "ENSGACG00000010542" "ENSGACG00000006959" "ENSGACG00000006965"
## [17782] "ENSGACG00000006980" "ENSGACG00000006986" "ENSGACG00000031812"
## [17785] "ENSGACG00000030201" "ENSGACG00000033973" "ENSGACG00000007002"
## [17788] "ENSGACG00000006413" "ENSGACG00000007019" "ENSGACG00000029261"
## [17791] "ENSGACG00000007098" "ENSGACG00000024327" "ENSGACG00000007165"
## [17794] "ENSGACG00000035359" "ENSGACG00000027748" "ENSGACG00000006428"
## [17797] "ENSGACG00000005971" "ENSGACG00000006501" "ENSGACG00000006509"
## [17800] "ENSGACG00000036859" "ENSGACG00000006544" "ENSGACG00000006577"
## [17803] "ENSGACG00000006589" "ENSGACG00000006596" "ENSGACG00000024756"
## [17806] "ENSGACG00000006661" "ENSGACG00000006680" "ENSGACG00000033566"
## [17809] "ENSGACG00000034979" "ENSGACG00000030700" "ENSGACG00000006693"
## [17812] "ENSGACG00000031403" "ENSGACG00000027695" "ENSGACG00000029299"
## [17815] "ENSGACG00000006717" "ENSGACG00000006725" "ENSGACG00000032079"
## [17818] "ENSGACG00000026720" "ENSGACG00000032517" "ENSGACG00000006764"
## [17821] "ENSGACG00000006772" "ENSGACG00000006181" "ENSGACG00000024699"
## [17824] "ENSGACG00000006183" "ENSGACG00000006184" "ENSGACG00000030638"
## [17827] "ENSGACG00000006194" "ENSGACG00000006205" "ENSGACG00000034055"
## [17830] "ENSGACG00000006298" "ENSGACG00000006778" "ENSGACG00000006787"
## [17833] "ENSGACG00000006800" "ENSGACG00000006309" "ENSGACG00000006330"
## [17836] "ENSGACG00000006338" "ENSGACG00000030084" "ENSGACG00000027375"
## [17839] "ENSGACG00000024059" "ENSGACG00000006856" "ENSGACG00000006881"
## [17842] "ENSGACG00000006884" "ENSGACG00000035479" "ENSGACG00000023131"
## [17845] "ENSGACG00000006898" "ENSGACG00000006909" "ENSGACG00000006931"
## [17848] "ENSGACG00000005976" "ENSGACG00000025145" "ENSGACG00000037101"
## [17851] "ENSGACG00000005993" "ENSGACG00000006000" "ENSGACG00000006007"
## [17854] "ENSGACG00000006010" "ENSGACG00000006023" "ENSGACG00000031568"
## [17857] "ENSGACG00000006040" "ENSGACG00000006086" "ENSGACG00000025570"
## [17860] "ENSGACG00000033950" "ENSGACG00000006109" "ENSGACG00000006146"
## [17863] "ENSGACG00000037346" "ENSGACG00000024137" "ENSGACG00000024359"
## [17866] "ENSGACG00000006368" "ENSGACG00000026261" "ENSGACG00000006386"
## [17869] "ENSGACG00000006394" "ENSGACG00000025558" "ENSGACG00000004674"
## [17872] "ENSGACG00000027045" "ENSGACG00000024823" "ENSGACG00000004666"
## [17875] "ENSGACG00000034531" "ENSGACG00000004625" "ENSGACG00000035121"
## [17878] "ENSGACG00000004609" "ENSGACG00000004602" "ENSGACG00000004587"
## [17881] "ENSGACG00000004571" "ENSGACG00000004554" "ENSGACG00000004541"
## [17884] "ENSGACG00000004533" "ENSGACG00000004515" "ENSGACG00000004495"
## [17887] "ENSGACG00000029273" "ENSGACG00000004484" "ENSGACG00000036494"
## [17890] "ENSGACG00000004430" "ENSGACG00000004416" "ENSGACG00000004400"
## [17893] "ENSGACG00000004849" "ENSGACG00000004851" "ENSGACG00000004871"
## [17896] "ENSGACG00000004860" "ENSGACG00000033576" "ENSGACG00000004888"
## [17899] "ENSGACG00000004891" "ENSGACG00000004895" "ENSGACG00000004902"
## [17902] "ENSGACG00000026724" "ENSGACG00000031363" "ENSGACG00000004925"
## [17905] "ENSGACG00000004936" "ENSGACG00000004951" "ENSGACG00000004975"
## [17908] "ENSGACG00000004978" "ENSGACG00000005044" "ENSGACG00000005070"
## [17911] "ENSGACG00000007930" "ENSGACG00000007948" "ENSGACG00000031853"
## [17914] "ENSGACG00000010372" "ENSGACG00000010383" "ENSGACG00000012424"
## [17917] "ENSGACG00000012514" "ENSGACG00000012260" "ENSGACG00000027883"
## [17920] "ENSGACG00000012287" "ENSGACG00000012312" "ENSGACG00000029378"
## [17923] "ENSGACG00000012334" "ENSGACG00000024798" "ENSGACG00000012400"
## [17926] "ENSGACG00000027856" "ENSGACG00000029024" "ENSGACG00000010437"
## [17929] "ENSGACG00000012346" "ENSGACG00000032715" "ENSGACG00000031435"
## [17932] "ENSGACG00000010500" "ENSGACG00000029410" "ENSGACG00000032651"
## [17935] "ENSGACG00000010507" "ENSGACG00000011612" "ENSGACG00000010527"
## [17938] "ENSGACG00000031035" "ENSGACG00000032195" "ENSGACG00000006398"
## [17941] "ENSGACG00000007179" "ENSGACG00000007211" "ENSGACG00000007221"
## [17944] "ENSGACG00000007232" "ENSGACG00000024572" "ENSGACG00000012411"
## [17947] "ENSGACG00000007250" "ENSGACG00000023165" "ENSGACG00000007280"
## [17950] "ENSGACG00000011624" "ENSGACG00000025534" "ENSGACG00000007301"
## [17953] "ENSGACG00000011640" "ENSGACG00000007305" "ENSGACG00000037960"
## [17956] "ENSGACG00000011649" "ENSGACG00000007329" "ENSGACG00000011655"
## [17959] "ENSGACG00000035213" "ENSGACG00000007369" "ENSGACG00000007351"
## [17962] "ENSGACG00000011667" "ENSGACG00000007385" "ENSGACG00000026131"
## [17965] "ENSGACG00000008807" "ENSGACG00000008818" "ENSGACG00000011683"
## [17968] "ENSGACG00000024123" "ENSGACG00000024749" "ENSGACG00000008724"
## [17971] "ENSGACG00000008795" "ENSGACG00000034038" "ENSGACG00000008800"
## [17974] "ENSGACG00000011698" "ENSGACG00000011704" "ENSGACG00000011710"
## [17977] "ENSGACG00000010180" "ENSGACG00000026699" "ENSGACG00000011719"
## [17980] "ENSGACG00000011736" "ENSGACG00000011743" "ENSGACG00000011747"
## [17983] "ENSGACG00000012545" "ENSGACG00000012585" "ENSGACG00000012589"
## [17986] "ENSGACG00000028015" "ENSGACG00000012619" "ENSGACG00000012635"
## [17989] "ENSGACG00000012698" "ENSGACG00000012707" "ENSGACG00000012713"
## [17992] "ENSGACG00000012761" "ENSGACG00000012771" "ENSGACG00000037962"
## [17995] "ENSGACG00000012773" "ENSGACG00000012782" "ENSGACG00000012788"
## [17998] "ENSGACG00000010025" "ENSGACG00000010029" "ENSGACG00000010236"
## [18001] "ENSGACG00000010242" "ENSGACG00000033303" "ENSGACG00000010072"
## [18004] "ENSGACG00000010083" "ENSGACG00000010109" "ENSGACG00000010116"
## [18007] "ENSGACG00000024769" "ENSGACG00000010122" "ENSGACG00000028918"
## [18010] "ENSGACG00000010131" "ENSGACG00000034952" "ENSGACG00000010149"
## [18013] "ENSGACG00000012000" "ENSGACG00000010156" "ENSGACG00000012008"
## [18016] "ENSGACG00000010166" "ENSGACG00000025410" "ENSGACG00000012025"
## [18019] "ENSGACG00000004683" "ENSGACG00000012041" "ENSGACG00000004676"
## [18022] "ENSGACG00000010581" "ENSGACG00000010578" "ENSGACG00000010583"
## [18025] "ENSGACG00000012063" "ENSGACG00000010586" "ENSGACG00000031079"
## [18028] "ENSGACG00000010590" "ENSGACG00000010602" "ENSGACG00000027138"
## [18031] "ENSGACG00000010608" "ENSGACG00000007997" "ENSGACG00000008015"
## [18034] "ENSGACG00000008025" "ENSGACG00000008042" "ENSGACG00000008048"
## [18037] "ENSGACG00000008087" "ENSGACG00000008094" "ENSGACG00000008105"
## [18040] "ENSGACG00000037205" "ENSGACG00000008116" "ENSGACG00000008119"
## [18043] "ENSGACG00000009924" "ENSGACG00000009929" "ENSGACG00000036598"
## [18046] "ENSGACG00000009955" "ENSGACG00000009968" "ENSGACG00000009976"
## [18049] "ENSGACG00000009986" "ENSGACG00000009994" "ENSGACG00000009995"
## [18052] "ENSGACG00000010002" "ENSGACG00000010017" "ENSGACG00000010021"
## [18055] "ENSGACG00000005291" "ENSGACG00000005081" "ENSGACG00000005118"
## [18058] "ENSGACG00000005133" "ENSGACG00000025189" "ENSGACG00000005170"
## [18061] "ENSGACG00000012819" "ENSGACG00000012823" "ENSGACG00000012839"
## [18064] "ENSGACG00000037772" "ENSGACG00000005190" "ENSGACG00000032731"
## [18067] "ENSGACG00000005202" "ENSGACG00000012116" "ENSGACG00000005212"
## [18070] "ENSGACG00000012122" "ENSGACG00000012130" "ENSGACG00000012135"
## [18073] "ENSGACG00000005262" "ENSGACG00000005272" "ENSGACG00000012149"
## [18076] "ENSGACG00000012167" "ENSGACG00000005303" "ENSGACG00000005327"
## [18079] "ENSGACG00000012183" "ENSGACG00000024525" "ENSGACG00000012186"
## [18082] "ENSGACG00000012192" "ENSGACG00000012195" "ENSGACG00000005344"
## [18085] "ENSGACG00000012204" "ENSGACG00000029425" "ENSGACG00000005358"
## [18088] "ENSGACG00000012416" "ENSGACG00000012527" "ENSGACG00000005363"
## [18091] "ENSGACG00000005369" "ENSGACG00000011995" "ENSGACG00000005393"
## [18094] "ENSGACG00000012223" "ENSGACG00000005421" "ENSGACG00000005493"
## [18097] "ENSGACG00000012234" "ENSGACG00000026125" "ENSGACG00000032175"
## [18100] "ENSGACG00000011761" "ENSGACG00000011769" "ENSGACG00000005536"
## [18103] "ENSGACG00000031428" "ENSGACG00000011778" "ENSGACG00000029191"
## [18106] "ENSGACG00000034058" "ENSGACG00000005564" "ENSGACG00000011787"
## [18109] "ENSGACG00000011791" "ENSGACG00000005582" "ENSGACG00000011795"
## [18112] "ENSGACG00000005647" "ENSGACG00000005673" "ENSGACG00000024207"
## [18115] "ENSGACG00000027372" "ENSGACG00000005698" "ENSGACG00000030782"
## [18118] "ENSGACG00000005714" "ENSGACG00000005723" "ENSGACG00000005738"
## [18121] "ENSGACG00000011810" "ENSGACG00000037693" "ENSGACG00000005770"
## [18124] "ENSGACG00000005774" "ENSGACG00000005788" "ENSGACG00000028234"
## [18127] "ENSGACG00000011842" "ENSGACG00000005814" "ENSGACG00000011850"
## [18130] "ENSGACG00000011856" "ENSGACG00000011862" "ENSGACG00000011867"
## [18133] "ENSGACG00000005826" "ENSGACG00000011876" "ENSGACG00000011880"
## [18136] "ENSGACG00000010619" "ENSGACG00000010625" "ENSGACG00000005856"
## [18139] "ENSGACG00000010634" "ENSGACG00000005867" "ENSGACG00000005882"
## [18142] "ENSGACG00000005888" "ENSGACG00000005896" "ENSGACG00000010657"
## [18145] "ENSGACG00000029854" "ENSGACG00000005932" "ENSGACG00000010673"
## [18148] "ENSGACG00000010688" "ENSGACG00000036064" "ENSGACG00000032457"
## [18151] "ENSGACG00000031350" "ENSGACG00000012843" "ENSGACG00000005935"
## [18154] "ENSGACG00000005951" "ENSGACG00000012868" "ENSGACG00000012877"
## [18157] "ENSGACG00000012898" "ENSGACG00000012880" "ENSGACG00000012907"
## [18160] "ENSGACG00000031654" "ENSGACG00000012914" "ENSGACG00000012929"
## [18163] "ENSGACG00000012956" "ENSGACG00000012959" "ENSGACG00000004373"
## [18166] "ENSGACG00000012967" "ENSGACG00000012974" "ENSGACG00000013000"
## [18169] "ENSGACG00000037201" "ENSGACG00000004368" "ENSGACG00000024506"
## [18172] "ENSGACG00000004351" "ENSGACG00000013022" "ENSGACG00000038056"
## [18175] "ENSGACG00000035422" "ENSGACG00000031488" "ENSGACG00000002001"
## [18178] "ENSGACG00000025351" "ENSGACG00000004323" "ENSGACG00000001996"
## [18181] "ENSGACG00000036601" "ENSGACG00000001995" "ENSGACG00000001988"
## [18184] "ENSGACG00000028805" "ENSGACG00000001983" "ENSGACG00000001963"
## [18187] "ENSGACG00000004311" "ENSGACG00000001957" "ENSGACG00000035224"
## [18190] "ENSGACG00000036343" "ENSGACG00000004181" "ENSGACG00000001946"
## [18193] "ENSGACG00000001939" "ENSGACG00000001915" "ENSGACG00000001898"
## [18196] "ENSGACG00000004177" "ENSGACG00000032138" "ENSGACG00000004172"
## [18199] "ENSGACG00000004158" "ENSGACG00000004148" "ENSGACG00000004071"
## [18202] "ENSGACG00000004038" "ENSGACG00000004018" "ENSGACG00000031722"
## [18205] "ENSGACG00000023877" "ENSGACG00000004005" "ENSGACG00000003990"
## [18208] "ENSGACG00000037342" "ENSGACG00000030013" "ENSGACG00000003968"
## [18211] "ENSGACG00000003943" "ENSGACG00000025241" "ENSGACG00000026254"
## [18214] "ENSGACG00000003916" "ENSGACG00000003907" "ENSGACG00000001891"
## [18217] "ENSGACG00000013366" "ENSGACG00000003885" "ENSGACG00000003883"
## [18220] "ENSGACG00000003879" "ENSGACG00000003868" "ENSGACG00000013343"
## [18223] "ENSGACG00000010805" "ENSGACG00000030353" "ENSGACG00000036492"
## [18226] "ENSGACG00000013319" "ENSGACG00000010813" "ENSGACG00000031220"
## [18229] "ENSGACG00000022980" "ENSGACG00000029355" "ENSGACG00000010841"
## [18232] "ENSGACG00000010844" "ENSGACG00000034172" "ENSGACG00000027885"
## [18235] "ENSGACG00000004847" "ENSGACG00000025599" "ENSGACG00000010860"
## [18238] "ENSGACG00000000520" "ENSGACG00000023632" "ENSGACG00000004823"
## [18241] "ENSGACG00000004835" "ENSGACG00000004809" "ENSGACG00000004792"
## [18244] "ENSGACG00000013305" "ENSGACG00000013299" "ENSGACG00000013286"
## [18247] "ENSGACG00000013274" "ENSGACG00000013267" "ENSGACG00000004786"
## [18250] "ENSGACG00000013261" "ENSGACG00000004779" "ENSGACG00000004758"
## [18253] "ENSGACG00000030181" "ENSGACG00000013255" "ENSGACG00000004742"
## [18256] "ENSGACG00000013250" "ENSGACG00000027599" "ENSGACG00000013212"
## [18259] "ENSGACG00000036519" "ENSGACG00000013180" "ENSGACG00000013169"
## [18262] "ENSGACG00000013166" "ENSGACG00000013158" "ENSGACG00000013156"
## [18265] "ENSGACG00000023278" "ENSGACG00000013137" "ENSGACG00000013132"
## [18268] "ENSGACG00000013126" "ENSGACG00000032340" "ENSGACG00000013081"
## [18271] "ENSGACG00000013067" "ENSGACG00000013056" "ENSGACG00000032745"
## [18274] "ENSGACG00000013047" "ENSGACG00000013038" "ENSGACG00000013033"
## [18277] "ENSGACG00000020130" "ENSGACG00000020131" "ENSGACG00000020134"
## [18280] "ENSGACG00000025384" "ENSGACG00000037747" "ENSGACG00000033427"
## [18283] "ENSGACG00000024132" "ENSGACG00000020138" "ENSGACG00000020140"
## [18286] "ENSGACG00000028413" "ENSGACG00000035684" "ENSGACG00000020143"
## [18289] "ENSGACG00000004730" "ENSGACG00000020147" "ENSGACG00000030241"
## [18292] "ENSGACG00000004700" "ENSGACG00000030428" "ENSGACG00000020150"
## [18295] "ENSGACG00000012922" "ENSGACG00000037051" "ENSGACG00000025252"
## [18298] "ENSGACG00000037127" "ENSGACG00000036730" "ENSGACG00000012428"
## [18301] "ENSGACG00000032800" "ENSGACG00000031371" "ENSGACG00000026876"
## [18304] "ENSGACG00000023726" "ENSGACG00000012431" "ENSGACG00000029745"
## [18307] "ENSGACG00000012444" "ENSGACG00000004815" "ENSGACG00000036039"
## [18310] "ENSGACG00000023671" "ENSGACG00000029992" "ENSGACG00000012459"
## [18313] "ENSGACG00000023456" "ENSGACG00000027499" "ENSGACG00000023972"
## [18316] "ENSGACG00000025700" "ENSGACG00000024494" "ENSGACG00000027369"
## [18319] "ENSGACG00000035277" "ENSGACG00000035749" "ENSGACG00000012476"
## [18322] "ENSGACG00000023261" "ENSGACG00000030239" "ENSGACG00000037138"
## [18325] "ENSGACG00000012481" "ENSGACG00000034625" "ENSGACG00000024733"
## [18328] "ENSGACG00000025265" "ENSGACG00000032928" "ENSGACG00000037083"
## [18331] "ENSGACG00000027017" "ENSGACG00000029429" "ENSGACG00000032868"
## [18334] "ENSGACG00000026767" "ENSGACG00000032238" "ENSGACG00000031317"
## [18337] "ENSGACG00000033933" "ENSGACG00000032227" "ENSGACG00000032749"
## [18340] "ENSGACG00000033396" "ENSGACG00000023625" "ENSGACG00000027069"
## [18343] "ENSGACG00000024353" "ENSGACG00000028460" "ENSGACG00000010731"
## [18346] "ENSGACG00000033282" "ENSGACG00000025376" "ENSGACG00000036147"
## [18349] "ENSGACG00000033477" "ENSGACG00000023842" "ENSGACG00000037578"
## [18352] "ENSGACG00000027864" "ENSGACG00000032034" "ENSGACG00000037528"
## [18355] "ENSGACG00000031585" "ENSGACG00000035439" "ENSGACG00000010755"
## [18358] "ENSGACG00000010760" "ENSGACG00000010777" "ENSGACG00000009413"
## [18361] "ENSGACG00000010787" "ENSGACG00000007550" "ENSGACG00000010829"
## [18364] "ENSGACG00000009591" "ENSGACG00000007821" "ENSGACG00000010835"
## [18367] "ENSGACG00000010839" "ENSGACG00000029040" "ENSGACG00000025451"
## [18370] "ENSGACG00000030480" "ENSGACG00000024214" "ENSGACG00000036951"
## [18373] "ENSGACG00000004987" "ENSGACG00000007266" "ENSGACG00000005156"
## [18376] "ENSGACG00000005379" "ENSGACG00000005451" "ENSGACG00000010847"
## [18379] "ENSGACG00000026836" "ENSGACG00000010895" "ENSGACG00000010923"
## [18382] "ENSGACG00000010930" "ENSGACG00000010937" "ENSGACG00000010956"
## [18385] "ENSGACG00000010958" "ENSGACG00000010967" "ENSGACG00000010985"
## [18388] "ENSGACG00000035148" "ENSGACG00000011010" "ENSGACG00000011024"
## [18391] "ENSGACG00000011030" "ENSGACG00000011037" "ENSGACG00000023245"
## [18394] "ENSGACG00000011048" "ENSGACG00000036116" "ENSGACG00000011055"
## [18397] "ENSGACG00000011100" "ENSGACG00000035819" "ENSGACG00000011133"
## [18400] "ENSGACG00000011144" "ENSGACG00000011153" "ENSGACG00000011166"
## [18403] "ENSGACG00000011196" "ENSGACG00000011235" "ENSGACG00000011264"
## [18406] "ENSGACG00000037500" "ENSGACG00000011275" "ENSGACG00000035136"
## [18409] "ENSGACG00000011289" "ENSGACG00000025995" "ENSGACG00000036915"
## [18412] "ENSGACG00000011304" "ENSGACG00000011310" "ENSGACG00000011314"
## [18415] "ENSGACG00000011361" "ENSGACG00000011377" "ENSGACG00000011386"
## [18418] "ENSGACG00000011391" "ENSGACG00000011401" "ENSGACG00000011419"
## [18421] "ENSGACG00000011463" "ENSGACG00000011476" "ENSGACG00000030820"
## [18424] "ENSGACG00000011481" "ENSGACG00000011487" "ENSGACG00000011498"
## [18427] "ENSGACG00000032867" "ENSGACG00000011511" "ENSGACG00000011566"
## [18430] "ENSGACG00000011576" "ENSGACG00000011577" "ENSGACG00000011582"
## [18433] "ENSGACG00000011598" "ENSGACG00000038047" "ENSGACG00000028366"
## [18436] "ENSGACG00000026307" "ENSGACG00000033323" "ENSGACG00000023722"
## [18439] "ENSGACG00000033898" "ENSGACG00000036554" "ENSGACG00000029820"
## [18442] "ENSGACG00000024681" "ENSGACG00000036715" "ENSGACG00000037395"
## [18445] "ENSGACG00000024384" "ENSGACG00000034271" "ENSGACG00000030330"
## [18448] "ENSGACG00000029220" "ENSGACG00000029151" "ENSGACG00000023844"
## [18451] "ENSGACG00000037496" "ENSGACG00000036683" "ENSGACG00000029369"
## [18454] "ENSGACG00000037697" "ENSGACG00000028180" "ENSGACG00000026769"
## [18457] "ENSGACG00000023341" "ENSGACG00000037456" "ENSGACG00000034316"
## [18460] "ENSGACG00000031805" "ENSGACG00000035566" "ENSGACG00000033480"
## [18463] "ENSGACG00000032291" "ENSGACG00000033908" "ENSGACG00000027273"
## [18466] "ENSGACG00000026056" "ENSGACG00000025523" "ENSGACG00000012056"
## [18469] "ENSGACG00000012540" "ENSGACG00000020144" "ENSGACG00000018106"
## [18472] "ENSGACG00000018113" "ENSGACG00000018118" "ENSGACG00000018121"
## [18475] "ENSGACG00000018018" "ENSGACG00000030552" "ENSGACG00000017820"
## [18478] "ENSGACG00000017822" "ENSGACG00000027778" "ENSGACG00000037123"
## [18481] "ENSGACG00000018531" "ENSGACG00000018526" "ENSGACG00000018522"
## [18484] "ENSGACG00000018520" "ENSGACG00000018517" "ENSGACG00000018515"
## [18487] "ENSGACG00000018512" "ENSGACG00000028385" "ENSGACG00000023928"
## [18490] "ENSGACG00000017899" "ENSGACG00000017908" "ENSGACG00000017918"
## [18493] "ENSGACG00000017924" "ENSGACG00000025635" "ENSGACG00000023028"
## [18496] "ENSGACG00000017943" "ENSGACG00000017948" "ENSGACG00000017950"
## [18499] "ENSGACG00000017952" "ENSGACG00000017956" "ENSGACG00000038021"
## [18502] "ENSGACG00000017970" "ENSGACG00000017973" "ENSGACG00000017986"
## [18505] "ENSGACG00000018010" "ENSGACG00000018012" "ENSGACG00000018014"
## [18508] "ENSGACG00000018510" "ENSGACG00000018505" "ENSGACG00000018502"
## [18511] "ENSGACG00000018499" "ENSGACG00000018495" "ENSGACG00000018492"
## [18514] "ENSGACG00000017859" "ENSGACG00000017864" "ENSGACG00000017867"
## [18517] "ENSGACG00000031467" "ENSGACG00000017876" "ENSGACG00000017878"
## [18520] "ENSGACG00000017884" "ENSGACG00000017886" "ENSGACG00000017890"
## [18523] "ENSGACG00000031313" "ENSGACG00000023661" "ENSGACG00000018021"
## [18526] "ENSGACG00000024781" "ENSGACG00000018063" "ENSGACG00000018061"
## [18529] "ENSGACG00000018069" "ENSGACG00000018073" "ENSGACG00000018078"
## [18532] "ENSGACG00000032498" "ENSGACG00000018087" "ENSGACG00000018094"
## [18535] "ENSGACG00000034621" "ENSGACG00000018100" "ENSGACG00000017824"
## [18538] "ENSGACG00000017829" "ENSGACG00000017839" "ENSGACG00000018486"
## [18541] "ENSGACG00000018482" "ENSGACG00000018470" "ENSGACG00000018468"
## [18544] "ENSGACG00000018462" "ENSGACG00000018460" "ENSGACG00000018458"
## [18547] "ENSGACG00000018446" "ENSGACG00000018437" "ENSGACG00000018428"
## [18550] "ENSGACG00000018427" "ENSGACG00000029108" "ENSGACG00000018424"
## [18553] "ENSGACG00000018404" "ENSGACG00000018403" "ENSGACG00000018402"
## [18556] "ENSGACG00000018399" "ENSGACG00000018397" "ENSGACG00000018395"
## [18559] "ENSGACG00000026438" "ENSGACG00000018388" "ENSGACG00000037602"
## [18562] "ENSGACG00000018381" "ENSGACG00000018375" "ENSGACG00000023115"
## [18565] "ENSGACG00000018369" "ENSGACG00000018368" "ENSGACG00000018363"
## [18568] "ENSGACG00000018353" "ENSGACG00000018349" "ENSGACG00000018343"
## [18571] "ENSGACG00000018342" "ENSGACG00000026443" "ENSGACG00000018335"
## [18574] "ENSGACG00000018329" "ENSGACG00000018323" "ENSGACG00000018317"
## [18577] "ENSGACG00000018308" "ENSGACG00000018309" "ENSGACG00000018307"
## [18580] "ENSGACG00000018306" "ENSGACG00000018305" "ENSGACG00000018302"
## [18583] "ENSGACG00000024014" "ENSGACG00000018304" "ENSGACG00000018301"
## [18586] "ENSGACG00000029547" "ENSGACG00000018294" "ENSGACG00000018284"
## [18589] "ENSGACG00000018282" "ENSGACG00000034409" "ENSGACG00000018266"
## [18592] "ENSGACG00000035118" "ENSGACG00000018277" "ENSGACG00000018269"
## [18595] "ENSGACG00000025908" "ENSGACG00000018259" "ENSGACG00000024540"
## [18598] "ENSGACG00000018248" "ENSGACG00000018241" "ENSGACG00000036525"
## [18601] "ENSGACG00000000651" "ENSGACG00000000648" "ENSGACG00000000637"
## [18604] "ENSGACG00000000631" "ENSGACG00000000628" "ENSGACG00000000626"
## [18607] "ENSGACG00000036077" "ENSGACG00000028109" "ENSGACG00000027398"
## [18610] "ENSGACG00000018125" "ENSGACG00000018132" "ENSGACG00000018134"
## [18613] "ENSGACG00000018136" "ENSGACG00000018142" "ENSGACG00000018145"
## [18616] "ENSGACG00000018146" "ENSGACG00000018150" "ENSGACG00000018156"
## [18619] "ENSGACG00000018162" "ENSGACG00000018166" "ENSGACG00000018172"
## [18622] "ENSGACG00000018173" "ENSGACG00000018174" "ENSGACG00000029818"
## [18625] "ENSGACG00000018179" "ENSGACG00000018180" "ENSGACG00000018182"
## [18628] "ENSGACG00000018189" "ENSGACG00000018192" "ENSGACG00000018232"
## [18631] "ENSGACG00000018199" "ENSGACG00000018201" "ENSGACG00000018203"
## [18634] "ENSGACG00000018204" "ENSGACG00000018217" "ENSGACG00000018227"
## [18637] "ENSGACG00000027300" "ENSGACG00000018235" "ENSGACG00000018236"
## [18640] "ENSGACG00000018237" "ENSGACG00000036206" "ENSGACG00000018240"
## [18643] "ENSGACG00000018673" "ENSGACG00000018667" "ENSGACG00000018661"
## [18646] "ENSGACG00000028600" "ENSGACG00000018654" "ENSGACG00000018650"
## [18649] "ENSGACG00000018644" "ENSGACG00000018641" "ENSGACG00000018635"
## [18652] "ENSGACG00000018632" "ENSGACG00000018624" "ENSGACG00000030334"
## [18655] "ENSGACG00000018619" "ENSGACG00000018599" "ENSGACG00000018584"
## [18658] "ENSGACG00000018575" "ENSGACG00000018569" "ENSGACG00000018562"
## [18661] "ENSGACG00000030545" "ENSGACG00000018551" "ENSGACG00000018549"
## [18664] "ENSGACG00000018538" "ENSGACG00000028747" "ENSGACG00000034432"
## [18667] "ENSGACG00000024516" "ENSGACG00000023985" "ENSGACG00000032883"
## [18670] "ENSGACG00000035286" "ENSGACG00000029616" "ENSGACG00000024840"
## [18673] "ENSGACG00000032199" "ENSGACG00000034837" "ENSGACG00000025256"
## [18676] "ENSGACG00000035597" "ENSGACG00000037651" "ENSGACG00000035317"
## [18679] "ENSGACG00000035140" "ENSGACG00000026130" "ENSGACG00000030447"
## [18682] "ENSGACG00000034077" "ENSGACG00000034806" "ENSGACG00000029030"
## [18685] "ENSGACG00000035364" "ENSGACG00000033413" "ENSGACG00000025409"
## [18688] "ENSGACG00000030843" "ENSGACG00000024396" "ENSGACG00000024966"
## [18691] "ENSGACG00000027554" "ENSGACG00000027731" "ENSGACG00000037069"
## [18694] "ENSGACG00000023873" "ENSGACG00000018441" "ENSGACG00000018130"
## [18697] "ENSGACG00000016793" "ENSGACG00000016799" "ENSGACG00000015920"
## [18700] "ENSGACG00000032532" "ENSGACG00000015926" "ENSGACG00000025597"
## [18703] "ENSGACG00000015930" "ENSGACG00000015932" "ENSGACG00000015934"
## [18706] "ENSGACG00000015938" "ENSGACG00000036426" "ENSGACG00000017647"
## [18709] "ENSGACG00000017659" "ENSGACG00000015918" "ENSGACG00000015942"
## [18712] "ENSGACG00000016297" "ENSGACG00000015230" "ENSGACG00000016425"
## [18715] "ENSGACG00000016429" "ENSGACG00000035537" "ENSGACG00000016453"
## [18718] "ENSGACG00000016456" "ENSGACG00000016460" "ENSGACG00000031002"
## [18721] "ENSGACG00000016463" "ENSGACG00000016085" "ENSGACG00000034956"
## [18724] "ENSGACG00000016089" "ENSGACG00000016094" "ENSGACG00000016097"
## [18727] "ENSGACG00000016123" "ENSGACG00000024752" "ENSGACG00000029393"
## [18730] "ENSGACG00000024009" "ENSGACG00000027610" "ENSGACG00000016142"
## [18733] "ENSGACG00000028883" "ENSGACG00000016157" "ENSGACG00000016160"
## [18736] "ENSGACG00000016162" "ENSGACG00000016169" "ENSGACG00000031626"
## [18739] "ENSGACG00000016171" "ENSGACG00000034941" "ENSGACG00000016478"
## [18742] "ENSGACG00000016173" "ENSGACG00000016480" "ENSGACG00000016180"
## [18745] "ENSGACG00000016186" "ENSGACG00000030096" "ENSGACG00000016487"
## [18748] "ENSGACG00000016211" "ENSGACG00000016491" "ENSGACG00000016503"
## [18751] "ENSGACG00000016216" "ENSGACG00000016510" "ENSGACG00000016222"
## [18754] "ENSGACG00000016514" "ENSGACG00000016225" "ENSGACG00000016233"
## [18757] "ENSGACG00000016240" "ENSGACG00000016244" "ENSGACG00000016260"
## [18760] "ENSGACG00000016270" "ENSGACG00000016038" "ENSGACG00000023156"
## [18763] "ENSGACG00000016051" "ENSGACG00000016058" "ENSGACG00000016062"
## [18766] "ENSGACG00000016074" "ENSGACG00000016079" "ENSGACG00000016083"
## [18769] "ENSGACG00000017660" "ENSGACG00000017668" "ENSGACG00000016302"
## [18772] "ENSGACG00000016802" "ENSGACG00000017670" "ENSGACG00000017673"
## [18775] "ENSGACG00000033195" "ENSGACG00000017680" "ENSGACG00000017684"
## [18778] "ENSGACG00000025811" "ENSGACG00000017687" "ENSGACG00000030325"
## [18781] "ENSGACG00000017695" "ENSGACG00000017699" "ENSGACG00000016805"
## [18784] "ENSGACG00000016817" "ENSGACG00000036149" "ENSGACG00000016831"
## [18787] "ENSGACG00000026626" "ENSGACG00000016833" "ENSGACG00000016835"
## [18790] "ENSGACG00000016842" "ENSGACG00000016849" "ENSGACG00000017711"
## [18793] "ENSGACG00000016852" "ENSGACG00000017714" "ENSGACG00000017729"
## [18796] "ENSGACG00000028075" "ENSGACG00000016858" "ENSGACG00000016864"
## [18799] "ENSGACG00000031069" "ENSGACG00000016867" "ENSGACG00000017739"
## [18802] "ENSGACG00000031287" "ENSGACG00000034118" "ENSGACG00000017745"
## [18805] "ENSGACG00000016869" "ENSGACG00000017750" "ENSGACG00000017757"
## [18808] "ENSGACG00000017774" "ENSGACG00000017776" "ENSGACG00000017780"
## [18811] "ENSGACG00000033300" "ENSGACG00000033728" "ENSGACG00000017783"
## [18814] "ENSGACG00000017786" "ENSGACG00000036929" "ENSGACG00000017793"
## [18817] "ENSGACG00000017805" "ENSGACG00000029613" "ENSGACG00000017807"
## [18820] "ENSGACG00000016305" "ENSGACG00000016308" "ENSGACG00000016312"
## [18823] "ENSGACG00000016330" "ENSGACG00000016333" "ENSGACG00000030315"
## [18826] "ENSGACG00000016351" "ENSGACG00000016369" "ENSGACG00000016376"
## [18829] "ENSGACG00000016396" "ENSGACG00000034455" "ENSGACG00000032017"
## [18832] "ENSGACG00000016410" "ENSGACG00000032032" "ENSGACG00000015771"
## [18835] "ENSGACG00000017360" "ENSGACG00000034305" "ENSGACG00000017364"
## [18838] "ENSGACG00000017285" "ENSGACG00000017293" "ENSGACG00000017301"
## [18841] "ENSGACG00000017305" "ENSGACG00000033249" "ENSGACG00000017311"
## [18844] "ENSGACG00000017329" "ENSGACG00000017340" "ENSGACG00000017341"
## [18847] "ENSGACG00000017344" "ENSGACG00000017345" "ENSGACG00000017348"
## [18850] "ENSGACG00000017355" "ENSGACG00000035521" "ENSGACG00000035774"
## [18853] "ENSGACG00000016628" "ENSGACG00000016633" "ENSGACG00000016644"
## [18856] "ENSGACG00000016647" "ENSGACG00000016651" "ENSGACG00000027728"
## [18859] "ENSGACG00000016655" "ENSGACG00000016660" "ENSGACG00000016668"
## [18862] "ENSGACG00000016704" "ENSGACG00000029032" "ENSGACG00000016714"
## [18865] "ENSGACG00000016724" "ENSGACG00000016728" "ENSGACG00000031506"
## [18868] "ENSGACG00000037266" "ENSGACG00000034555" "ENSGACG00000032240"
## [18871] "ENSGACG00000016294" "ENSGACG00000035013" "ENSGACG00000016879"
## [18874] "ENSGACG00000016885" "ENSGACG00000016887" "ENSGACG00000016889"
## [18877] "ENSGACG00000016908" "ENSGACG00000029167" "ENSGACG00000016911"
## [18880] "ENSGACG00000016912" "ENSGACG00000016916" "ENSGACG00000016921"
## [18883] "ENSGACG00000016928" "ENSGACG00000033916" "ENSGACG00000027324"
## [18886] "ENSGACG00000016940" "ENSGACG00000016943" "ENSGACG00000016945"
## [18889] "ENSGACG00000016947" "ENSGACG00000016949" "ENSGACG00000016950"
## [18892] "ENSGACG00000016954" "ENSGACG00000016963" "ENSGACG00000016965"
## [18895] "ENSGACG00000032535" "ENSGACG00000016973" "ENSGACG00000031222"
## [18898] "ENSGACG00000016981" "ENSGACG00000025281" "ENSGACG00000032368"
## [18901] "ENSGACG00000016983" "ENSGACG00000016993" "ENSGACG00000016995"
## [18904] "ENSGACG00000017002" "ENSGACG00000036178" "ENSGACG00000017007"
## [18907] "ENSGACG00000017020" "ENSGACG00000034290" "ENSGACG00000017026"
## [18910] "ENSGACG00000032665" "ENSGACG00000036146" "ENSGACG00000017041"
## [18913] "ENSGACG00000017048" "ENSGACG00000017057" "ENSGACG00000026890"
## [18916] "ENSGACG00000017068" "ENSGACG00000030452" "ENSGACG00000017070"
## [18919] "ENSGACG00000017132" "ENSGACG00000017085" "ENSGACG00000017088"
## [18922] "ENSGACG00000027395" "ENSGACG00000017152" "ENSGACG00000017154"
## [18925] "ENSGACG00000017091" "ENSGACG00000017156" "ENSGACG00000017163"
## [18928] "ENSGACG00000017166" "ENSGACG00000017180" "ENSGACG00000031588"
## [18931] "ENSGACG00000017192" "ENSGACG00000017093" "ENSGACG00000023224"
## [18934] "ENSGACG00000017101" "ENSGACG00000029789" "ENSGACG00000017197"
## [18937] "ENSGACG00000017120" "ENSGACG00000011885" "ENSGACG00000037096"
## [18940] "ENSGACG00000017126" "ENSGACG00000024250" "ENSGACG00000017222"
## [18943] "ENSGACG00000017227" "ENSGACG00000017232" "ENSGACG00000017239"
## [18946] "ENSGACG00000017246" "ENSGACG00000017249" "ENSGACG00000032438"
## [18949] "ENSGACG00000031765" "ENSGACG00000026741" "ENSGACG00000035262"
## [18952] "ENSGACG00000029925" "ENSGACG00000016743" "ENSGACG00000016747"
## [18955] "ENSGACG00000016756" "ENSGACG00000025830" "ENSGACG00000034517"
## [18958] "ENSGACG00000031498" "ENSGACG00000016778" "ENSGACG00000016783"
## [18961] "ENSGACG00000015575" "ENSGACG00000015584" "ENSGACG00000015591"
## [18964] "ENSGACG00000015598" "ENSGACG00000015604" "ENSGACG00000029629"
## [18967] "ENSGACG00000035799" "ENSGACG00000015623" "ENSGACG00000024402"
## [18970] "ENSGACG00000015627" "ENSGACG00000025450" "ENSGACG00000015633"
## [18973] "ENSGACG00000015636" "ENSGACG00000015639" "ENSGACG00000015641"
## [18976] "ENSGACG00000016517" "ENSGACG00000033286" "ENSGACG00000016518"
## [18979] "ENSGACG00000016523" "ENSGACG00000016528" "ENSGACG00000016531"
## [18982] "ENSGACG00000016537" "ENSGACG00000016540" "ENSGACG00000016541"
## [18985] "ENSGACG00000016543" "ENSGACG00000016548" "ENSGACG00000025459"
## [18988] "ENSGACG00000016554" "ENSGACG00000016565" "ENSGACG00000016572"
## [18991] "ENSGACG00000026026" "ENSGACG00000016588" "ENSGACG00000016591"
## [18994] "ENSGACG00000016592" "ENSGACG00000016596" "ENSGACG00000016598"
## [18997] "ENSGACG00000030323" "ENSGACG00000016603" "ENSGACG00000016611"
## [19000] "ENSGACG00000025184" "ENSGACG00000016615" "ENSGACG00000000996"
## [19003] "ENSGACG00000034341" "ENSGACG00000034681" "ENSGACG00000015775"
## [19006] "ENSGACG00000015777" "ENSGACG00000015778" "ENSGACG00000015780"
## [19009] "ENSGACG00000015784" "ENSGACG00000015800" "ENSGACG00000028028"
## [19012] "ENSGACG00000015803" "ENSGACG00000015806" "ENSGACG00000015808"
## [19015] "ENSGACG00000015813" "ENSGACG00000015820" "ENSGACG00000023679"
## [19018] "ENSGACG00000029935" "ENSGACG00000032211" "ENSGACG00000033219"
## [19021] "ENSGACG00000034089" "ENSGACG00000015828" "ENSGACG00000015834"
## [19024] "ENSGACG00000029106" "ENSGACG00000015839" "ENSGACG00000015841"
## [19027] "ENSGACG00000015842" "ENSGACG00000015843" "ENSGACG00000015844"
## [19030] "ENSGACG00000015847" "ENSGACG00000015852" "ENSGACG00000038055"
## [19033] "ENSGACG00000015854" "ENSGACG00000015949" "ENSGACG00000015856"
## [19036] "ENSGACG00000015957" "ENSGACG00000015983" "ENSGACG00000026943"
## [19039] "ENSGACG00000015857" "ENSGACG00000015989" "ENSGACG00000015859"
## [19042] "ENSGACG00000015991" "ENSGACG00000015860" "ENSGACG00000024718"
## [19045] "ENSGACG00000015864" "ENSGACG00000016008" "ENSGACG00000016013"
## [19048] "ENSGACG00000016016" "ENSGACG00000016018" "ENSGACG00000016021"
## [19051] "ENSGACG00000016022" "ENSGACG00000016027" "ENSGACG00000034429"
## [19054] "ENSGACG00000015866" "ENSGACG00000015867" "ENSGACG00000015870"
## [19057] "ENSGACG00000015874" "ENSGACG00000015881" "ENSGACG00000015884"
## [19060] "ENSGACG00000036092" "ENSGACG00000015886" "ENSGACG00000015887"
## [19063] "ENSGACG00000015889" "ENSGACG00000015892" "ENSGACG00000029187"
## [19066] "ENSGACG00000015896" "ENSGACG00000015898" "ENSGACG00000015902"
## [19069] "ENSGACG00000015903" "ENSGACG00000022938" "ENSGACG00000030164"
## [19072] "ENSGACG00000032596" "ENSGACG00000015914" "ENSGACG00000015915"
## [19075] "ENSGACG00000015916" "ENSGACG00000015917" "ENSGACG00000017365"
## [19078] "ENSGACG00000017368" "ENSGACG00000017369" "ENSGACG00000017388"
## [19081] "ENSGACG00000036558" "ENSGACG00000017377" "ENSGACG00000034887"
## [19084] "ENSGACG00000017392" "ENSGACG00000017396" "ENSGACG00000017404"
## [19087] "ENSGACG00000035719" "ENSGACG00000033989" "ENSGACG00000017409"
## [19090] "ENSGACG00000017420" "ENSGACG00000017442" "ENSGACG00000017447"
## [19093] "ENSGACG00000017449" "ENSGACG00000017455" "ENSGACG00000017460"
## [19096] "ENSGACG00000017474" "ENSGACG00000017484" "ENSGACG00000017495"
## [19099] "ENSGACG00000029930" "ENSGACG00000017505" "ENSGACG00000017507"
## [19102] "ENSGACG00000017517" "ENSGACG00000017525" "ENSGACG00000017528"
## [19105] "ENSGACG00000017533" "ENSGACG00000029838" "ENSGACG00000017537"
## [19108] "ENSGACG00000017543" "ENSGACG00000017566" "ENSGACG00000017586"
## [19111] "ENSGACG00000017593" "ENSGACG00000017598" "ENSGACG00000017602"
## [19114] "ENSGACG00000017611" "ENSGACG00000017617" "ENSGACG00000017640"
## [19117] "ENSGACG00000028718" "ENSGACG00000001888" "ENSGACG00000001889"
## [19120] "ENSGACG00000001899" "ENSGACG00000001905" "ENSGACG00000018072"
## [19123] "ENSGACG00000001914" "ENSGACG00000001920" "ENSGACG00000033455"
## [19126] "ENSGACG00000001938" "ENSGACG00000034940" "ENSGACG00000015555"
## [19129] "ENSGACG00000015552" "ENSGACG00000037508" "ENSGACG00000015546"
## [19132] "ENSGACG00000015545" "ENSGACG00000035634" "ENSGACG00000015540"
## [19135] "ENSGACG00000023776" "ENSGACG00000015533" "ENSGACG00000015526"
## [19138] "ENSGACG00000015522" "ENSGACG00000015518" "ENSGACG00000015512"
## [19141] "ENSGACG00000015490" "ENSGACG00000036217" "ENSGACG00000025442"
## [19144] "ENSGACG00000015485" "ENSGACG00000015481" "ENSGACG00000033210"
## [19147] "ENSGACG00000015480" "ENSGACG00000034098" "ENSGACG00000015476"
## [19150] "ENSGACG00000015475" "ENSGACG00000015467" "ENSGACG00000030596"
## [19153] "ENSGACG00000015461" "ENSGACG00000015458" "ENSGACG00000033179"
## [19156] "ENSGACG00000015456" "ENSGACG00000015449" "ENSGACG00000015446"
## [19159] "ENSGACG00000015440" "ENSGACG00000038014" "ENSGACG00000028241"
## [19162] "ENSGACG00000027436" "ENSGACG00000000847" "ENSGACG00000000851"
## [19165] "ENSGACG00000028536" "ENSGACG00000000855" "ENSGACG00000000856"
## [19168] "ENSGACG00000030595" "ENSGACG00000000860" "ENSGACG00000027787"
## [19171] "ENSGACG00000000392" "ENSGACG00000000372" "ENSGACG00000000366"
## [19174] "ENSGACG00000037615" "ENSGACG00000000363" "ENSGACG00000038116"
## [19177] "ENSGACG00000000354" "ENSGACG00000000351" "ENSGACG00000000342"
## [19180] "ENSGACG00000037439" "ENSGACG00000000338" "ENSGACG00000000334"
## [19183] "ENSGACG00000035218" "ENSGACG00000000321" "ENSGACG00000015564"
## [19186] "ENSGACG00000015567" "ENSGACG00000015644" "ENSGACG00000015647"
## [19189] "ENSGACG00000033558" "ENSGACG00000028984" "ENSGACG00000015651"
## [19192] "ENSGACG00000030582" "ENSGACG00000015669" "ENSGACG00000015673"
## [19195] "ENSGACG00000031695" "ENSGACG00000015685" "ENSGACG00000015693"
## [19198] "ENSGACG00000015697" "ENSGACG00000015705" "ENSGACG00000015707"
## [19201] "ENSGACG00000015712" "ENSGACG00000015717" "ENSGACG00000015730"
## [19204] "ENSGACG00000015736" "ENSGACG00000037590" "ENSGACG00000015737"
## [19207] "ENSGACG00000015741" "ENSGACG00000015744" "ENSGACG00000015751"
## [19210] "ENSGACG00000029711" "ENSGACG00000015755" "ENSGACG00000034881"
## [19213] "ENSGACG00000037767" "ENSGACG00000015759" "ENSGACG00000015761"
## [19216] "ENSGACG00000015762" "ENSGACG00000023537" "ENSGACG00000017727"
## [19219] "ENSGACG00000037239" "ENSGACG00000016608" "ENSGACG00000024400"
## [19222] "ENSGACG00000025957" "ENSGACG00000037665" "ENSGACG00000034310"
## [19225] "ENSGACG00000025872" "ENSGACG00000028899" "ENSGACG00000033328"
## [19228] "ENSGACG00000036673" "ENSGACG00000033562" "ENSGACG00000027528"
## [19231] "ENSGACG00000022978" "ENSGACG00000036963" "ENSGACG00000031025"
## [19234] "ENSGACG00000035561" "ENSGACG00000029685" "ENSGACG00000027116"
## [19237] "ENSGACG00000034152" "ENSGACG00000037594" "ENSGACG00000032653"
## [19240] "ENSGACG00000024694" "ENSGACG00000037663" "ENSGACG00000037288"
## [19243] "ENSGACG00000025334" "ENSGACG00000032785" "ENSGACG00000029121"
## [19246] "ENSGACG00000027456" "ENSGACG00000031228" "ENSGACG00000037538"
## [19249] "ENSGACG00000025386" "ENSGACG00000036838" "ENSGACG00000025760"
## [19252] "ENSGACG00000037720" "ENSGACG00000023957" "ENSGACG00000034072"
## [19255] "ENSGACG00000026037" "ENSGACG00000033917" "ENSGACG00000031255"
## [19258] "ENSGACG00000023199" "ENSGACG00000024443" "ENSGACG00000029707"
## [19261] "ENSGACG00000026287" "ENSGACG00000030437" "ENSGACG00000035763"
## [19264] "ENSGACG00000033177" "ENSGACG00000024048" "ENSGACG00000029934"
## [19267] "ENSGACG00000022343" "ENSGACG00000016477" "ENSGACG00000016822"
## [19270] "ENSGACG00000030350" "ENSGACG00000024469" "ENSGACG00000017053"
## [19273] "ENSGACG00000017252" "ENSGACG00000017144" "ENSGACG00000017281"
## [19276] "ENSGACG00000025742" "ENSGACG00000016574" "ENSGACG00000015894"
## [19279] "ENSGACG00000017370" "ENSGACG00000017577" "ENSGACG00000036574"
## [19282] "ENSGACG00000026117" "ENSGACG00000012273" "ENSGACG00000012274"
## [19285] "ENSGACG00000012933" "ENSGACG00000012938" "ENSGACG00000012960"
## [19288] "ENSGACG00000024805" "ENSGACG00000012969" "ENSGACG00000012281"
## [19291] "ENSGACG00000029083" "ENSGACG00000011427" "ENSGACG00000011449"
## [19294] "ENSGACG00000011452" "ENSGACG00000011457" "ENSGACG00000036337"
## [19297] "ENSGACG00000037592" "ENSGACG00000011495" "ENSGACG00000011517"
## [19300] "ENSGACG00000011522" "ENSGACG00000011525" "ENSGACG00000011533"
## [19303] "ENSGACG00000011539" "ENSGACG00000036507" "ENSGACG00000011589"
## [19306] "ENSGACG00000037225" "ENSGACG00000011608" "ENSGACG00000024544"
## [19309] "ENSGACG00000011631" "ENSGACG00000011646" "ENSGACG00000011654"
## [19312] "ENSGACG00000011656" "ENSGACG00000025510" "ENSGACG00000011665"
## [19315] "ENSGACG00000011674" "ENSGACG00000011712" "ENSGACG00000031982"
## [19318] "ENSGACG00000028972" "ENSGACG00000011732" "ENSGACG00000011768"
## [19321] "ENSGACG00000011776" "ENSGACG00000011780" "ENSGACG00000024129"
## [19324] "ENSGACG00000011819" "ENSGACG00000011852" "ENSGACG00000011855"
## [19327] "ENSGACG00000011860" "ENSGACG00000011863" "ENSGACG00000025154"
## [19330] "ENSGACG00000024868" "ENSGACG00000011870" "ENSGACG00000011887"
## [19333] "ENSGACG00000011895" "ENSGACG00000011900" "ENSGACG00000011904"
## [19336] "ENSGACG00000027858" "ENSGACG00000011913" "ENSGACG00000011935"
## [19339] "ENSGACG00000011952" "ENSGACG00000011966" "ENSGACG00000011974"
## [19342] "ENSGACG00000033293" "ENSGACG00000035614" "ENSGACG00000011992"
## [19345] "ENSGACG00000012001" "ENSGACG00000012004" "ENSGACG00000012012"
## [19348] "ENSGACG00000012033" "ENSGACG00000012039" "ENSGACG00000012042"
## [19351] "ENSGACG00000012051" "ENSGACG00000012065" "ENSGACG00000024844"
## [19354] "ENSGACG00000012080" "ENSGACG00000012108" "ENSGACG00000012137"
## [19357] "ENSGACG00000036522" "ENSGACG00000012142" "ENSGACG00000012152"
## [19360] "ENSGACG00000012161" "ENSGACG00000012177" "ENSGACG00000012185"
## [19363] "ENSGACG00000027715" "ENSGACG00000012193" "ENSGACG00000012199"
## [19366] "ENSGACG00000026244" "ENSGACG00000012235" "ENSGACG00000012241"
## [19369] "ENSGACG00000012252" "ENSGACG00000012261" "ENSGACG00000023176"
## [19372] "ENSGACG00000012296" "ENSGACG00000012303" "ENSGACG00000033142"
## [19375] "ENSGACG00000012313" "ENSGACG00000026095" "ENSGACG00000012322"
## [19378] "ENSGACG00000012333" "ENSGACG00000012337" "ENSGACG00000012341"
## [19381] "ENSGACG00000012370" "ENSGACG00000012380" "ENSGACG00000012385"
## [19384] "ENSGACG00000012393" "ENSGACG00000012397" "ENSGACG00000012401"
## [19387] "ENSGACG00000012406" "ENSGACG00000012415" "ENSGACG00000012426"
## [19390] "ENSGACG00000012430" "ENSGACG00000012445" "ENSGACG00000012471"
## [19393] "ENSGACG00000012478" "ENSGACG00000012487" "ENSGACG00000012490"
## [19396] "ENSGACG00000012503" "ENSGACG00000012532" "ENSGACG00000012537"
## [19399] "ENSGACG00000012546" "ENSGACG00000012550" "ENSGACG00000032649"
## [19402] "ENSGACG00000012581" "ENSGACG00000012595" "ENSGACG00000012599"
## [19405] "ENSGACG00000012603" "ENSGACG00000012614" "ENSGACG00000012666"
## [19408] "ENSGACG00000023908" "ENSGACG00000023978" "ENSGACG00000012675"
## [19411] "ENSGACG00000012726" "ENSGACG00000012738" "ENSGACG00000032913"
## [19414] "ENSGACG00000012749" "ENSGACG00000037187" "ENSGACG00000012765"
## [19417] "ENSGACG00000012791" "ENSGACG00000012834" "ENSGACG00000012846"
## [19420] "ENSGACG00000012854" "ENSGACG00000031172" "ENSGACG00000012871"
## [19423] "ENSGACG00000012875" "ENSGACG00000012912" "ENSGACG00000036555"
## [19426] "ENSGACG00000023858" "ENSGACG00000034319" "ENSGACG00000012930"
## [19429] "ENSGACG00000037361" "ENSGACG00000012982" "ENSGACG00000012992"
## [19432] "ENSGACG00000012994" "ENSGACG00000013025" "ENSGACG00000013040"
## [19435] "ENSGACG00000037049" "ENSGACG00000013068" "ENSGACG00000013087"
## [19438] "ENSGACG00000013092" "ENSGACG00000013101" "ENSGACG00000013108"
## [19441] "ENSGACG00000023270" "ENSGACG00000013124" "ENSGACG00000013144"
## [19444] "ENSGACG00000013145" "ENSGACG00000025193" "ENSGACG00000025331"
## [19447] "ENSGACG00000013160" "ENSGACG00000013184" "ENSGACG00000013187"
## [19450] "ENSGACG00000034307" "ENSGACG00000013240" "ENSGACG00000013248"
## [19453] "ENSGACG00000013252" "ENSGACG00000013254" "ENSGACG00000013262"
## [19456] "ENSGACG00000013265" "ENSGACG00000033347" "ENSGACG00000013403"
## [19459] "ENSGACG00000013271" "ENSGACG00000013275" "ENSGACG00000013301"
## [19462] "ENSGACG00000035544" "ENSGACG00000013310" "ENSGACG00000013313"
## [19465] "ENSGACG00000013322" "ENSGACG00000013342" "ENSGACG00000013356"
## [19468] "ENSGACG00000032402" "ENSGACG00000013427" "ENSGACG00000013374"
## [19471] "ENSGACG00000013389" "ENSGACG00000013394" "ENSGACG00000013413"
## [19474] "ENSGACG00000036552" "ENSGACG00000013437" "ENSGACG00000013449"
## [19477] "ENSGACG00000013458" "ENSGACG00000013471" "ENSGACG00000013498"
## [19480] "ENSGACG00000013502" "ENSGACG00000029008" "ENSGACG00000013530"
## [19483] "ENSGACG00000013536" "ENSGACG00000032682" "ENSGACG00000013550"
## [19486] "ENSGACG00000013554" "ENSGACG00000013559" "ENSGACG00000013562"
## [19489] "ENSGACG00000013567" "ENSGACG00000013569" "ENSGACG00000013571"
## [19492] "ENSGACG00000013577" "ENSGACG00000013580" "ENSGACG00000028037"
## [19495] "ENSGACG00000010144" "ENSGACG00000010160" "ENSGACG00000010171"
## [19498] "ENSGACG00000010188" "ENSGACG00000010191" "ENSGACG00000010204"
## [19501] "ENSGACG00000010221" "ENSGACG00000010266" "ENSGACG00000033817"
## [19504] "ENSGACG00000010278" "ENSGACG00000010284" "ENSGACG00000026773"
## [19507] "ENSGACG00000010288" "ENSGACG00000010299" "ENSGACG00000010318"
## [19510] "ENSGACG00000010336" "ENSGACG00000010351" "ENSGACG00000010354"
## [19513] "ENSGACG00000010367" "ENSGACG00000025688" "ENSGACG00000010441"
## [19516] "ENSGACG00000010450" "ENSGACG00000010476" "ENSGACG00000034355"
## [19519] "ENSGACG00000010513" "ENSGACG00000010524" "ENSGACG00000010529"
## [19522] "ENSGACG00000026224" "ENSGACG00000010546" "ENSGACG00000010561"
## [19525] "ENSGACG00000010565" "ENSGACG00000007602" "ENSGACG00000025601"
## [19528] "ENSGACG00000010599" "ENSGACG00000034680" "ENSGACG00000010609"
## [19531] "ENSGACG00000010624" "ENSGACG00000010660" "ENSGACG00000010663"
## [19534] "ENSGACG00000010680" "ENSGACG00000010698" "ENSGACG00000010719"
## [19537] "ENSGACG00000030425" "ENSGACG00000010774" "ENSGACG00000032977"
## [19540] "ENSGACG00000010791" "ENSGACG00000010799" "ENSGACG00000027612"
## [19543] "ENSGACG00000010806" "ENSGACG00000033961" "ENSGACG00000010817"
## [19546] "ENSGACG00000010859" "ENSGACG00000029354" "ENSGACG00000010874"
## [19549] "ENSGACG00000010882" "ENSGACG00000010887" "ENSGACG00000010894"
## [19552] "ENSGACG00000010905" "ENSGACG00000029143" "ENSGACG00000031174"
## [19555] "ENSGACG00000010932" "ENSGACG00000010950" "ENSGACG00000010959"
## [19558] "ENSGACG00000010968" "ENSGACG00000010973" "ENSGACG00000011008"
## [19561] "ENSGACG00000011035" "ENSGACG00000011054" "ENSGACG00000011060"
## [19564] "ENSGACG00000011073" "ENSGACG00000011079" "ENSGACG00000011098"
## [19567] "ENSGACG00000011108" "ENSGACG00000011113" "ENSGACG00000011115"
## [19570] "ENSGACG00000011123" "ENSGACG00000011129" "ENSGACG00000011134"
## [19573] "ENSGACG00000011136" "ENSGACG00000011162" "ENSGACG00000011256"
## [19576] "ENSGACG00000011280" "ENSGACG00000011327" "ENSGACG00000011352"
## [19579] "ENSGACG00000011355" "ENSGACG00000030193" "ENSGACG00000011365"
## [19582] "ENSGACG00000025763" "ENSGACG00000011402" "ENSGACG00000037818"
## [19585] "ENSGACG00000026972" "ENSGACG00000035927" "ENSGACG00000036352"
## [19588] "ENSGACG00000031064" "ENSGACG00000032279" "ENSGACG00000034552"
## [19591] "ENSGACG00000023517" "ENSGACG00000025140" "ENSGACG00000029276"
## [19594] "ENSGACG00000024973" "ENSGACG00000033040" "ENSGACG00000035609"
## [19597] "ENSGACG00000023937" "ENSGACG00000028168" "ENSGACG00000031423"
## [19600] "ENSGACG00000030903" "ENSGACG00000030789" "ENSGACG00000024943"
## [19603] "ENSGACG00000036509" "ENSGACG00000036007" "ENSGACG00000033604"
## [19606] "ENSGACG00000029678" "ENSGACG00000035341" "ENSGACG00000025053"
## [19609] "ENSGACG00000032233" "ENSGACG00000034877" "ENSGACG00000027187"
## [19612] "ENSGACG00000035555" "ENSGACG00000034511" "ENSGACG00000012579"
## [19615] "ENSGACG00000036579" "ENSGACG00000036114" "ENSGACG00000028013"
## [19618] "ENSGACG00000035865" "ENSGACG00000027131" "ENSGACG00000032349"
## [19621] "ENSGACG00000035418" "ENSGACG00000027890" "ENSGACG00000033834"
## [19624] "ENSGACG00000028551" "ENSGACG00000035421" "ENSGACG00000036150"
## [19627] "ENSGACG00000032415" "ENSGACG00000028475" "ENSGACG00000035646"
## [19630] "ENSGACG00000011597" "ENSGACG00000011993" "ENSGACG00000012888"
## [19633] "ENSGACG00000013010" "ENSGACG00000025938" "ENSGACG00000004010"
## [19636] "ENSGACG00000004029" "ENSGACG00000004065" "ENSGACG00000037521"
## [19639] "ENSGACG00000004093" "ENSGACG00000031926" "ENSGACG00000004110"
## [19642] "ENSGACG00000004116" "ENSGACG00000033446" "ENSGACG00000004185"
## [19645] "ENSGACG00000004217" "ENSGACG00000004242" "ENSGACG00000032022"
## [19648] "ENSGACG00000025892" "ENSGACG00000004251" "ENSGACG00000004253"
## [19651] "ENSGACG00000004255" "ENSGACG00000004266" "ENSGACG00000004270"
## [19654] "ENSGACG00000037412" "ENSGACG00000024418" "ENSGACG00000004292"
## [19657] "ENSGACG00000004319" "ENSGACG00000023092" "ENSGACG00000024378"
## [19660] "ENSGACG00000004335" "ENSGACG00000025004" "ENSGACG00000004381"
## [19663] "ENSGACG00000004386" "ENSGACG00000004391" "ENSGACG00000004401"
## [19666] "ENSGACG00000004427" "ENSGACG00000035490" "ENSGACG00000004464"
## [19669] "ENSGACG00000004469" "ENSGACG00000025594" "ENSGACG00000026922"
## [19672] "ENSGACG00000004482" "ENSGACG00000004508" "ENSGACG00000004520"
## [19675] "ENSGACG00000004546" "ENSGACG00000004618" "ENSGACG00000004658"
## [19678] "ENSGACG00000004668" "ENSGACG00000004679" "ENSGACG00000004707"
## [19681] "ENSGACG00000037989" "ENSGACG00000004722" "ENSGACG00000004727"
## [19684] "ENSGACG00000035700" "ENSGACG00000031464" "ENSGACG00000004803"
## [19687] "ENSGACG00000004825" "ENSGACG00000004893" "ENSGACG00000035694"
## [19690] "ENSGACG00000036416" "ENSGACG00000004940" "ENSGACG00000036273"
## [19693] "ENSGACG00000033193" "ENSGACG00000034241" "ENSGACG00000022919"
## [19696] "ENSGACG00000031356" "ENSGACG00000006339" "ENSGACG00000006385"
## [19699] "ENSGACG00000006402" "ENSGACG00000006414" "ENSGACG00000006450"
## [19702] "ENSGACG00000006471" "ENSGACG00000006475" "ENSGACG00000033106"
## [19705] "ENSGACG00000006498" "ENSGACG00000006924" "ENSGACG00000006932"
## [19708] "ENSGACG00000006940" "ENSGACG00000006958" "ENSGACG00000006319"
## [19711] "ENSGACG00000006287" "ENSGACG00000033684" "ENSGACG00000005022"
## [19714] "ENSGACG00000005065" "ENSGACG00000005093" "ENSGACG00000031912"
## [19717] "ENSGACG00000005162" "ENSGACG00000005206" "ENSGACG00000032123"
## [19720] "ENSGACG00000005224" "ENSGACG00000005233" "ENSGACG00000005269"
## [19723] "ENSGACG00000005280" "ENSGACG00000005310" "ENSGACG00000005382"
## [19726] "ENSGACG00000005410" "ENSGACG00000005517" "ENSGACG00000005537"
## [19729] "ENSGACG00000005542" "ENSGACG00000005556" "ENSGACG00000005602"
## [19732] "ENSGACG00000005610" "ENSGACG00000034059" "ENSGACG00000005653"
## [19735] "ENSGACG00000005668" "ENSGACG00000005681" "ENSGACG00000005694"
## [19738] "ENSGACG00000005795" "ENSGACG00000005799" "ENSGACG00000030182"
## [19741] "ENSGACG00000005828" "ENSGACG00000030128" "ENSGACG00000005916"
## [19744] "ENSGACG00000005924" "ENSGACG00000005960" "ENSGACG00000033541"
## [19747] "ENSGACG00000005989" "ENSGACG00000006020" "ENSGACG00000006047"
## [19750] "ENSGACG00000006091" "ENSGACG00000006098" "ENSGACG00000006113"
## [19753] "ENSGACG00000006168" "ENSGACG00000006142" "ENSGACG00000006155"
## [19756] "ENSGACG00000006171" "ENSGACG00000037408" "ENSGACG00000006185"
## [19759] "ENSGACG00000006188" "ENSGACG00000031083" "ENSGACG00000006245"
## [19762] "ENSGACG00000006251" "ENSGACG00000006268" "ENSGACG00000007991"
## [19765] "ENSGACG00000007998" "ENSGACG00000008026" "ENSGACG00000008077"
## [19768] "ENSGACG00000008099" "ENSGACG00000036606" "ENSGACG00000034437"
## [19771] "ENSGACG00000024775" "ENSGACG00000008117" "ENSGACG00000008134"
## [19774] "ENSGACG00000025667" "ENSGACG00000008200" "ENSGACG00000008226"
## [19777] "ENSGACG00000008247" "ENSGACG00000008279" "ENSGACG00000008281"
## [19780] "ENSGACG00000031913" "ENSGACG00000035898" "ENSGACG00000008363"
## [19783] "ENSGACG00000029588" "ENSGACG00000008395" "ENSGACG00000008404"
## [19786] "ENSGACG00000025000" "ENSGACG00000008410" "ENSGACG00000008418"
## [19789] "ENSGACG00000008433" "ENSGACG00000027370" "ENSGACG00000008454"
## [19792] "ENSGACG00000006507" "ENSGACG00000006570" "ENSGACG00000006602"
## [19795] "ENSGACG00000026953" "ENSGACG00000006636" "ENSGACG00000006639"
## [19798] "ENSGACG00000006668" "ENSGACG00000006685" "ENSGACG00000006700"
## [19801] "ENSGACG00000006720" "ENSGACG00000006727" "ENSGACG00000026029"
## [19804] "ENSGACG00000006748" "ENSGACG00000006766" "ENSGACG00000006789"
## [19807] "ENSGACG00000006812" "ENSGACG00000006836" "ENSGACG00000023756"
## [19810] "ENSGACG00000031020" "ENSGACG00000006840" "ENSGACG00000006862"
## [19813] "ENSGACG00000024695" "ENSGACG00000006873" "ENSGACG00000006877"
## [19816] "ENSGACG00000006882" "ENSGACG00000031024" "ENSGACG00000006891"
## [19819] "ENSGACG00000006916" "ENSGACG00000034839" "ENSGACG00000036671"
## [19822] "ENSGACG00000006995" "ENSGACG00000007011" "ENSGACG00000007021"
## [19825] "ENSGACG00000007097" "ENSGACG00000007107" "ENSGACG00000007156"
## [19828] "ENSGACG00000007169" "ENSGACG00000007187" "ENSGACG00000007209"
## [19831] "ENSGACG00000007215" "ENSGACG00000007258" "ENSGACG00000007264"
## [19834] "ENSGACG00000007277" "ENSGACG00000031824" "ENSGACG00000007328"
## [19837] "ENSGACG00000007459" "ENSGACG00000007463" "ENSGACG00000007514"
## [19840] "ENSGACG00000007522" "ENSGACG00000007543" "ENSGACG00000007552"
## [19843] "ENSGACG00000032186" "ENSGACG00000007565" "ENSGACG00000007571"
## [19846] "ENSGACG00000007582" "ENSGACG00000007592" "ENSGACG00000007631"
## [19849] "ENSGACG00000007636" "ENSGACG00000007644" "ENSGACG00000007661"
## [19852] "ENSGACG00000007699" "ENSGACG00000007722" "ENSGACG00000007742"
## [19855] "ENSGACG00000007808" "ENSGACG00000007823" "ENSGACG00000007834"
## [19858] "ENSGACG00000007847" "ENSGACG00000007853" "ENSGACG00000007876"
## [19861] "ENSGACG00000007883" "ENSGACG00000007898" "ENSGACG00000025738"
## [19864] "ENSGACG00000007936" "ENSGACG00000007942" "ENSGACG00000008491"
## [19867] "ENSGACG00000008518" "ENSGACG00000008522" "ENSGACG00000008569"
## [19870] "ENSGACG00000008623" "ENSGACG00000008654" "ENSGACG00000008664"
## [19873] "ENSGACG00000008691" "ENSGACG00000030964" "ENSGACG00000008711"
## [19876] "ENSGACG00000008721" "ENSGACG00000026989" "ENSGACG00000008797"
## [19879] "ENSGACG00000035175" "ENSGACG00000032037" "ENSGACG00000008833"
## [19882] "ENSGACG00000008851" "ENSGACG00000008859" "ENSGACG00000008868"
## [19885] "ENSGACG00000008871" "ENSGACG00000008882" "ENSGACG00000008895"
## [19888] "ENSGACG00000008903" "ENSGACG00000008910" "ENSGACG00000008916"
## [19891] "ENSGACG00000008921" "ENSGACG00000008934" "ENSGACG00000008950"
## [19894] "ENSGACG00000008956" "ENSGACG00000008978" "ENSGACG00000009000"
## [19897] "ENSGACG00000009045" "ENSGACG00000009052" "ENSGACG00000009066"
## [19900] "ENSGACG00000009204" "ENSGACG00000009212" "ENSGACG00000009220"
## [19903] "ENSGACG00000009228" "ENSGACG00000031944" "ENSGACG00000009248"
## [19906] "ENSGACG00000035528" "ENSGACG00000009276" "ENSGACG00000009285"
## [19909] "ENSGACG00000009350" "ENSGACG00000032242" "ENSGACG00000009366"
## [19912] "ENSGACG00000035514" "ENSGACG00000009432" "ENSGACG00000009479"
## [19915] "ENSGACG00000029433" "ENSGACG00000009538" "ENSGACG00000026734"
## [19918] "ENSGACG00000009555" "ENSGACG00000009586" "ENSGACG00000009616"
## [19921] "ENSGACG00000009637" "ENSGACG00000009670" "ENSGACG00000009681"
## [19924] "ENSGACG00000009713" "ENSGACG00000009719" "ENSGACG00000009775"
## [19927] "ENSGACG00000009796" "ENSGACG00000009809" "ENSGACG00000009811"
## [19930] "ENSGACG00000031085" "ENSGACG00000009824" "ENSGACG00000009830"
## [19933] "ENSGACG00000009847" "ENSGACG00000009860" "ENSGACG00000009865"
## [19936] "ENSGACG00000024348" "ENSGACG00000031712" "ENSGACG00000009916"
## [19939] "ENSGACG00000009925" "ENSGACG00000009942" "ENSGACG00000009980"
## [19942] "ENSGACG00000009984" "ENSGACG00000009990" "ENSGACG00000009991"
## [19945] "ENSGACG00000010006" "ENSGACG00000010010" "ENSGACG00000010022"
## [19948] "ENSGACG00000010030" "ENSGACG00000033082" "ENSGACG00000027881"
## [19951] "ENSGACG00000034233" "ENSGACG00000010062" "ENSGACG00000010065"
## [19954] "ENSGACG00000010074" "ENSGACG00000010084" "ENSGACG00000010088"
## [19957] "ENSGACG00000010098" "ENSGACG00000010112" "ENSGACG00000010127"
## [19960] "ENSGACG00000010140" "ENSGACG00000031060" "ENSGACG00000029057"
## [19963] "ENSGACG00000024481" "ENSGACG00000033089" "ENSGACG00000025557"
## [19966] "ENSGACG00000034195" "ENSGACG00000032992" "ENSGACG00000038006"
## [19969] "ENSGACG00000027192" "ENSGACG00000025595" "ENSGACG00000026867"
## [19972] "ENSGACG00000025405" "ENSGACG00000025249" "ENSGACG00000030287"
## [19975] "ENSGACG00000029549" "ENSGACG00000026198" "ENSGACG00000035072"
## [19978] "ENSGACG00000035400" "ENSGACG00000033744" "ENSGACG00000025213"
## [19981] "ENSGACG00000036548" "ENSGACG00000037589" "ENSGACG00000036289"
## [19984] "ENSGACG00000025200" "ENSGACG00000028895" "ENSGACG00000028949"
## [19987] "ENSGACG00000027995" "ENSGACG00000027927" "ENSGACG00000037745"
## [19990] "ENSGACG00000035384" "ENSGACG00000026936" "ENSGACG00000034157"
## [19993] "ENSGACG00000036411" "ENSGACG00000009211" "ENSGACG00000025583"
## [19996] "ENSGACG00000027021" "ENSGACG00000030272" "ENSGACG00000025143"
## [19999] "ENSGACG00000036144" "ENSGACG00000032224" "ENSGACG00000035637"
## [20002] "ENSGACG00000034325" "ENSGACG00000036323" "ENSGACG00000027568"
## [20005] "ENSGACG00000027486" "ENSGACG00000037587" "ENSGACG00000032881"
## [20008] "ENSGACG00000038042" "ENSGACG00000023685" "ENSGACG00000023014"
## [20011] "ENSGACG00000036632" "ENSGACG00000034426" "ENSGACG00000036312"
## [20014] "ENSGACG00000007483" "ENSGACG00000009723" "ENSGACG00000006871"
## [20017] "ENSGACG00000011095" "ENSGACG00000031250" "ENSGACG00000025753"
## [20020] "ENSGACG00000011138" "ENSGACG00000033944" "ENSGACG00000027278"
## [20023] "ENSGACG00000024219" "ENSGACG00000035395" "ENSGACG00000035056"
## [20026] "ENSGACG00000031995" "ENSGACG00000025549" "ENSGACG00000025206"
## [20029] "ENSGACG00000027757" "ENSGACG00000029384" "ENSGACG00000025973"
## [20032] "ENSGACG00000022933" "ENSGACG00000037006" "ENSGACG00000031839"
## [20035] "ENSGACG00000032565" "ENSGACG00000038075" "ENSGACG00000011362"
## [20038] "ENSGACG00000031420" "ENSGACG00000026396" "ENSGACG00000036356"
## [20041] "ENSGACG00000035549" "ENSGACG00000035477" "ENSGACG00000028370"
## [20044] "ENSGACG00000030051" "ENSGACG00000032654" "ENSGACG00000032390"
## [20047] "ENSGACG00000011466" "ENSGACG00000026292" "ENSGACG00000032304"
## [20050] "ENSGACG00000032453" "ENSGACG00000028843" "ENSGACG00000034075"
## [20053] "ENSGACG00000028488" "ENSGACG00000034513" "ENSGACG00000024098"
## [20056] "ENSGACG00000026214" "ENSGACG00000029366" "ENSGACG00000035414"
## [20059] "ENSGACG00000030949" "ENSGACG00000023562" "ENSGACG00000025220"
## [20062] "ENSGACG00000036799" "ENSGACG00000032942" "ENSGACG00000031645"
## [20065] "ENSGACG00000029585" "ENSGACG00000033637" "ENSGACG00000011688"
## [20068] "ENSGACG00000011693" "ENSGACG00000032904" "ENSGACG00000011713"
## [20071] "ENSGACG00000029664" "ENSGACG00000036912" "ENSGACG00000037770"
## [20074] "ENSGACG00000011764" "ENSGACG00000028099" "ENSGACG00000033422"
## [20077] "ENSGACG00000011794" "ENSGACG00000023693" "ENSGACG00000035018"
## [20080] "ENSGACG00000011838" "ENSGACG00000036968" "ENSGACG00000011888"
## [20083] "ENSGACG00000011896" "ENSGACG00000011916" "ENSGACG00000011944"
## [20086] "ENSGACG00000034509" "ENSGACG00000031421" "ENSGACG00000034908"
## [20089] "ENSGACG00000030527" "ENSGACG00000025971" "ENSGACG00000033108"
## [20092] "ENSGACG00000024659" "ENSGACG00000028024" "ENSGACG00000012066"
## [20095] "ENSGACG00000029610" "ENSGACG00000026582" "ENSGACG00000034666"
## [20098] "ENSGACG00000029497" "ENSGACG00000034155" "ENSGACG00000012125"
## [20101] "ENSGACG00000025563" "ENSGACG00000012213" "ENSGACG00000012216"
## [20104] "ENSGACG00000037800" "ENSGACG00000024895" "ENSGACG00000026059"
## [20107] "ENSGACG00000012500" "ENSGACG00000028910" "ENSGACG00000027297"
## [20110] "ENSGACG00000026689" "ENSGACG00000036541" "ENSGACG00000035989"
## [20113] "ENSGACG00000023760" "ENSGACG00000012458" "ENSGACG00000031463"
## [20116] "ENSGACG00000012534" "ENSGACG00000030123" "ENSGACG00000012582"
## [20119] "ENSGACG00000031627" "ENSGACG00000035151" "ENSGACG00000023062"
## [20122] "ENSGACG00000012638" "ENSGACG00000034092" "ENSGACG00000033184"
## [20125] "ENSGACG00000012696" "ENSGACG00000012703" "ENSGACG00000024050"
## [20128] "ENSGACG00000012733" "ENSGACG00000035882" "ENSGACG00000012758"
## [20131] "ENSGACG00000035138" "ENSGACG00000035009" "ENSGACG00000031938"
## [20134] "ENSGACG00000025861" "ENSGACG00000012813" "ENSGACG00000012832"
## [20137] "ENSGACG00000012845" "ENSGACG00000012874" "ENSGACG00000033434"
## [20140] "ENSGACG00000028849" "ENSGACG00000025307" "ENSGACG00000013049"
## [20143] "ENSGACG00000023332" "ENSGACG00000030069" "ENSGACG00000031679"
## [20146] "ENSGACG00000025755" "ENSGACG00000029823" "ENSGACG00000033253"
## [20149] "ENSGACG00000013170" "ENSGACG00000013182" "ENSGACG00000013219"
## [20152] "ENSGACG00000013235" "ENSGACG00000026399" "ENSGACG00000033945"
## [20155] "ENSGACG00000037499" "ENSGACG00000032975" "ENSGACG00000034113"
## [20158] "ENSGACG00000024759" "ENSGACG00000037341" "ENSGACG00000037531"
## [20161] "ENSGACG00000035322" "ENSGACG00000010861" "ENSGACG00000010873"
## [20164] "ENSGACG00000029785" "ENSGACG00000037248" "ENSGACG00000027939"
## [20167] "ENSGACG00000030744" "ENSGACG00000036562" "ENSGACG00000029389"
## [20170] "ENSGACG00000032639" "ENSGACG00000027956" "ENSGACG00000023406"
## [20173] "ENSGACG00000035990" "ENSGACG00000030853" "ENSGACG00000031412"
## [20176] "ENSGACG00000037378" "ENSGACG00000030124" "ENSGACG00000033686"
## [20179] "ENSGACG00000029367" "ENSGACG00000035664" "ENSGACG00000026614"
## [20182] "ENSGACG00000023612" "ENSGACG00000028558" "ENSGACG00000024800"
## [20185] "ENSGACG00000032012" "ENSGACG00000035959" "ENSGACG00000036999"
## [20188] "ENSGACG00000030629" "ENSGACG00000027276" "ENSGACG00000031688"
## [20191] "ENSGACG00000027378" "ENSGACG00000037254" "ENSGACG00000032258"
## [20194] "ENSGACG00000023468" "ENSGACG00000036644" "ENSGACG00000024144"
## [20197] "ENSGACG00000011990" "ENSGACG00000012805" "ENSGACG00000034761"
## [20200] "ENSGACG00000026218" "ENSGACG00000026570" "ENSGACG00000026939"
## [20203] "ENSGACG00000023088" "ENSGACG00000024760" "ENSGACG00000032170"
## [20206] "ENSGACG00000027584" "ENSGACG00000029996" "ENSGACG00000035116"
## [20209] "ENSGACG00000023786" "ENSGACG00000030678" "ENSGACG00000036225"
## [20212] "ENSGACG00000029343" "ENSGACG00000023519" "ENSGACG00000035300"
## [20215] "ENSGACG00000034999" "ENSGACG00000027822" "ENSGACG00000006090"
## [20218] "ENSGACG00000027661" "ENSGACG00000024737" "ENSGACG00000027684"
## [20221] "ENSGACG00000024918" "ENSGACG00000037365" "ENSGACG00000031257"
## [20224] "ENSGACG00000033439" "ENSGACG00000037495" "ENSGACG00000004968"
## [20227] "ENSGACG00000031819" "ENSGACG00000032071" "ENSGACG00000031062"
## [20230] "ENSGACG00000006341" "ENSGACG00000006326" "ENSGACG00000026098"
## [20233] "ENSGACG00000030826" "ENSGACG00000032691" "ENSGACG00000033401"
## [20236] "ENSGACG00000028332" "ENSGACG00000030760" "ENSGACG00000035693"
## [20239] "ENSGACG00000028426" "ENSGACG00000034103" "ENSGACG00000024509"
## [20242] "ENSGACG00000030088" "ENSGACG00000004329" "ENSGACG00000036770"
## [20245] "ENSGACG00000027809" "ENSGACG00000009091" "ENSGACG00000029748"
## [20248] "ENSGACG00000029044" "ENSGACG00000004426" "ENSGACG00000017337"
## [20251] "ENSGACG00000027926" "ENSGACG00000034270" "ENSGACG00000030294"
## [20254] "ENSGACG00000023748" "ENSGACG00000028518" "ENSGACG00000001482"
## [20257] "ENSGACG00000008349" "ENSGACG00000007895" "ENSGACG00000030271"
## [20260] "ENSGACG00000026597" "ENSGACG00000037562" "ENSGACG00000034413"
## [20263] "ENSGACG00000010672" "ENSGACG00000025709" "ENSGACG00000032562"
## [20266] "ENSGACG00000028122" "ENSGACG00000034723" "ENSGACG00000035240"
## [20269] "ENSGACG00000010550" "ENSGACG00000031339" "ENSGACG00000030226"
## [20272] "ENSGACG00000024251" "ENSGACG00000025621" "ENSGACG00000026404"
## [20275] "ENSGACG00000025288" "ENSGACG00000026671" "ENSGACG00000036154"
## [20278] "ENSGACG00000006809" "ENSGACG00000033965" "ENSGACG00000028395"
## [20281] "ENSGACG00000033720" "ENSGACG00000036711" "ENSGACG00000026164"
## [20284] "ENSGACG00000036311" "ENSGACG00000036925" "ENSGACG00000027626"
## [20287] "ENSGACG00000026042" "ENSGACG00000026834" "ENSGACG00000028581"
## [20290] "ENSGACG00000033517" "ENSGACG00000006929" "ENSGACG00000005974"
## [20293] "ENSGACG00000007844" "ENSGACG00000028238" "ENSGACG00000028973"
## [20296] "ENSGACG00000007474" "ENSGACG00000031170" "ENSGACG00000032112"
## [20299] "ENSGACG00000031541" "ENSGACG00000027741" "ENSGACG00000023714"
## [20302] "ENSGACG00000024583" "ENSGACG00000013579" "ENSGACG00000030732"
## [20305] "ENSGACG00000028104" "ENSGACG00000034028" "ENSGACG00000023993"
## [20308] "ENSGACG00000013528" "ENSGACG00000036675" "ENSGACG00000026817"
## [20311] "ENSGACG00000030103" "ENSGACG00000013775" "ENSGACG00000032090"
## [20314] "ENSGACG00000034123" "ENSGACG00000037149" "ENSGACG00000013683"
## [20317] "ENSGACG00000026529" "ENSGACG00000026369" "ENSGACG00000013648"
## [20320] "ENSGACG00000035065" "ENSGACG00000036800" "ENSGACG00000006582"
## [20323] "ENSGACG00000037912" "ENSGACG00000033115" "ENSGACG00000013784"
## [20326] "ENSGACG00000036438" "ENSGACG00000024929" "ENSGACG00000038093"
## [20329] "ENSGACG00000013794" "ENSGACG00000028907" "ENSGACG00000013796"
## [20332] "ENSGACG00000027336" "ENSGACG00000027177" "ENSGACG00000025370"
## [20335] "ENSGACG00000024305" "ENSGACG00000024153" "ENSGACG00000013827"
## [20338] "ENSGACG00000030992" "ENSGACG00000013830" "ENSGACG00000035759"
## [20341] "ENSGACG00000023497" "ENSGACG00000013862" "ENSGACG00000031076"
## [20344] "ENSGACG00000026558" "ENSGACG00000033561" "ENSGACG00000033790"
## [20347] "ENSGACG00000026541" "ENSGACG00000027597" "ENSGACG00000027663"
## [20350] "ENSGACG00000032609" "ENSGACG00000013972" "ENSGACG00000023180"
## [20353] "ENSGACG00000025188" "ENSGACG00000014034" "ENSGACG00000014072"
## [20356] "ENSGACG00000036883" "ENSGACG00000031397" "ENSGACG00000028700"
## [20359] "ENSGACG00000035864" "ENSGACG00000023735" "ENSGACG00000014098"
## [20362] "ENSGACG00000030249" "ENSGACG00000003422" "ENSGACG00000028003"
## [20365] "ENSGACG00000028062" "ENSGACG00000003358" "ENSGACG00000024931"
## [20368] "ENSGACG00000034285" "ENSGACG00000036462" "ENSGACG00000024037"
## [20371] "ENSGACG00000025262" "ENSGACG00000029156" "ENSGACG00000025010"
## [20374] "ENSGACG00000037714" "ENSGACG00000029196" "ENSGACG00000037563"
## [20377] "ENSGACG00000037577" "ENSGACG00000031442" "ENSGACG00000003248"
## [20380] "ENSGACG00000003219" "ENSGACG00000033194" "ENSGACG00000003209"
## [20383] "ENSGACG00000028043" "ENSGACG00000028935" "ENSGACG00000026185"
## [20386] "ENSGACG00000003190" "ENSGACG00000036232" "ENSGACG00000035706"
## [20389] "ENSGACG00000003148" "ENSGACG00000037215" "ENSGACG00000023212"
## [20392] "ENSGACG00000032995" "ENSGACG00000033209" "ENSGACG00000025723"
## [20395] "ENSGACG00000026496" "ENSGACG00000033710" "ENSGACG00000037406"
## [20398] "ENSGACG00000031677" "ENSGACG00000035139" "ENSGACG00000028481"
## [20401] "ENSGACG00000010174" "ENSGACG00000035964" "ENSGACG00000030384"
## [20404] "ENSGACG00000024669" "ENSGACG00000032835" "ENSGACG00000029706"
## [20407] "ENSGACG00000030108" "ENSGACG00000013447" "ENSGACG00000031598"
## [20410] "ENSGACG00000013390" "ENSGACG00000013388" "ENSGACG00000027002"
## [20413] "ENSGACG00000028664" "ENSGACG00000025049" "ENSGACG00000013377"
## [20416] "ENSGACG00000034090" "ENSGACG00000035433" "ENSGACG00000026955"
## [20419] "ENSGACG00000024064" "ENSGACG00000025052" "ENSGACG00000024091"
## [20422] "ENSGACG00000033522" "ENSGACG00000029463" "ENSGACG00000011021"
## [20425] "ENSGACG00000037174" "ENSGACG00000031591" "ENSGACG00000011050"
## [20428] "ENSGACG00000011053" "ENSGACG00000034363" "ENSGACG00000011081"
## [20431] "ENSGACG00000032491" "ENSGACG00000029026" "ENSGACG00000028962"
## [20434] "ENSGACG00000032282" "ENSGACG00000030829" "ENSGACG00000011042"
## [20437] "ENSGACG00000032969" "ENSGACG00000032513" "ENSGACG00000027924"
## [20440] "ENSGACG00000029278" "ENSGACG00000023219" "ENSGACG00000026765"
## [20443] "ENSGACG00000029051" "ENSGACG00000031258" "ENSGACG00000027951"
## [20446] "ENSGACG00000029009" "ENSGACG00000033423" "ENSGACG00000027091"
## [20449] "ENSGACG00000023789" "ENSGACG00000030609" "ENSGACG00000028520"
## [20452] "ENSGACG00000026444" "ENSGACG00000036532" "ENSGACG00000027052"
## [20455] "ENSGACG00000029948" "ENSGACG00000025799" "ENSGACG00000022915"
## [20458] "ENSGACG00000035106" "ENSGACG00000027871" "ENSGACG00000037972"
## [20461] "ENSGACG00000037849" "ENSGACG00000028296" "ENSGACG00000028358"
## [20464] "ENSGACG00000028363" "ENSGACG00000030684" "ENSGACG00000030337"
## [20467] "ENSGACG00000024585" "ENSGACG00000035101" "ENSGACG00000024230"
## [20470] "ENSGACG00000031348" "ENSGACG00000035319" "ENSGACG00000007160"
## [20473] "ENSGACG00000007096" "ENSGACG00000007104" "ENSGACG00000032321"
## [20476] "ENSGACG00000007116" "ENSGACG00000008204" "ENSGACG00000035377"
## [20479] "ENSGACG00000008211" "ENSGACG00000008214" "ENSGACG00000008223"
## [20482] "ENSGACG00000029836" "ENSGACG00000036572" "ENSGACG00000008256"
## [20485] "ENSGACG00000026563" "ENSGACG00000008270" "ENSGACG00000008273"
## [20488] "ENSGACG00000008293" "ENSGACG00000008320" "ENSGACG00000008335"
## [20491] "ENSGACG00000008345" "ENSGACG00000008354" "ENSGACG00000027468"
## [20494] "ENSGACG00000008379" "ENSGACG00000008397" "ENSGACG00000034435"
## [20497] "ENSGACG00000008414" "ENSGACG00000008423" "ENSGACG00000008419"
## [20500] "ENSGACG00000008436" "ENSGACG00000008442" "ENSGACG00000008461"
## [20503] "ENSGACG00000008467" "ENSGACG00000027850" "ENSGACG00000028360"
## [20506] "ENSGACG00000009105" "ENSGACG00000009099" "ENSGACG00000009083"
## [20509] "ENSGACG00000009036" "ENSGACG00000034011" "ENSGACG00000009028"
## [20512] "ENSGACG00000033020" "ENSGACG00000009003" "ENSGACG00000008992"
## [20515] "ENSGACG00000008987" "ENSGACG00000032631" "ENSGACG00000008975"
## [20518] "ENSGACG00000023037" "ENSGACG00000008965" "ENSGACG00000008959"
## [20521] "ENSGACG00000032781" "ENSGACG00000008893" "ENSGACG00000008886"
## [20524] "ENSGACG00000008873" "ENSGACG00000008802" "ENSGACG00000008782"
## [20527] "ENSGACG00000025172" "ENSGACG00000035981" "ENSGACG00000030117"
## [20530] "ENSGACG00000008706" "ENSGACG00000008689" "ENSGACG00000008675"
## [20533] "ENSGACG00000030879" "ENSGACG00000008686" "ENSGACG00000029419"
## [20536] "ENSGACG00000008670" "ENSGACG00000008665" "ENSGACG00000008640"
## [20539] "ENSGACG00000008627" "ENSGACG00000008595" "ENSGACG00000024200"
## [20542] "ENSGACG00000008564" "ENSGACG00000037829" "ENSGACG00000035337"
## [20545] "ENSGACG00000032209" "ENSGACG00000008529" "ENSGACG00000025798"
## [20548] "ENSGACG00000008526" "ENSGACG00000008498" "ENSGACG00000030160"
## [20551] "ENSGACG00000009956" "ENSGACG00000009935" "ENSGACG00000009898"
## [20554] "ENSGACG00000031992" "ENSGACG00000009888" "ENSGACG00000007088"
## [20557] "ENSGACG00000002323" "ENSGACG00000002322" "ENSGACG00000009872"
## [20560] "ENSGACG00000009815" "ENSGACG00000009759" "ENSGACG00000009751"
## [20563] "ENSGACG00000009745" "ENSGACG00000009737" "ENSGACG00000009724"
## [20566] "ENSGACG00000009715" "ENSGACG00000031757" "ENSGACG00000009710"
## [20569] "ENSGACG00000009704" "ENSGACG00000023243" "ENSGACG00000009694"
## [20572] "ENSGACG00000009690" "ENSGACG00000025822" "ENSGACG00000009568"
## [20575] "ENSGACG00000009675" "ENSGACG00000009581" "ENSGACG00000009554"
## [20578] "ENSGACG00000009531" "ENSGACG00000009526" "ENSGACG00000028655"
## [20581] "ENSGACG00000009512" "ENSGACG00000009489" "ENSGACG00000032251"
## [20584] "ENSGACG00000009474" "ENSGACG00000009459" "ENSGACG00000009455"
## [20587] "ENSGACG00000009409" "ENSGACG00000024797" "ENSGACG00000025268"
## [20590] "ENSGACG00000009374" "ENSGACG00000009367" "ENSGACG00000024205"
## [20593] "ENSGACG00000009357" "ENSGACG00000002370" "ENSGACG00000002365"
## [20596] "ENSGACG00000002343" "ENSGACG00000002340" "ENSGACG00000002330"
## [20599] "ENSGACG00000006851" "ENSGACG00000006858" "ENSGACG00000006865"
## [20602] "ENSGACG00000006899" "ENSGACG00000006910" "ENSGACG00000006989"
## [20605] "ENSGACG00000028751" "ENSGACG00000007007" "ENSGACG00000007018"
## [20608] "ENSGACG00000007042" "ENSGACG00000007050" "ENSGACG00000007057"
## [20611] "ENSGACG00000007059" "ENSGACG00000029321" "ENSGACG00000029891"
## [20614] "ENSGACG00000007083" "ENSGACG00000002317" "ENSGACG00000002304"
## [20617] "ENSGACG00000002299" "ENSGACG00000002292" "ENSGACG00000002284"
## [20620] "ENSGACG00000002272" "ENSGACG00000002262" "ENSGACG00000002248"
## [20623] "ENSGACG00000035755" "ENSGACG00000002242" "ENSGACG00000002233"
## [20626] "ENSGACG00000002213" "ENSGACG00000002197" "ENSGACG00000026505"
## [20629] "ENSGACG00000031461" "ENSGACG00000002187" "ENSGACG00000002173"
## [20632] "ENSGACG00000002159" "ENSGACG00000002142" "ENSGACG00000002140"
## [20635] "ENSGACG00000002129" "ENSGACG00000002125" "ENSGACG00000029469"
## [20638] "ENSGACG00000002123" "ENSGACG00000002115" "ENSGACG00000002103"
## [20641] "ENSGACG00000002098" "ENSGACG00000036328" "ENSGACG00000023841"
## [20644] "ENSGACG00000005969" "ENSGACG00000028642" "ENSGACG00000006028"
## [20647] "ENSGACG00000006033" "ENSGACG00000006049" "ENSGACG00000006059"
## [20650] "ENSGACG00000006114" "ENSGACG00000006129" "ENSGACG00000006133"
## [20653] "ENSGACG00000006152" "ENSGACG00000006162" "ENSGACG00000023826"
## [20656] "ENSGACG00000006213" "ENSGACG00000006221" "ENSGACG00000006244"
## [20659] "ENSGACG00000006346" "ENSGACG00000006343" "ENSGACG00000034653"
## [20662] "ENSGACG00000024685" "ENSGACG00000006374" "ENSGACG00000006378"
## [20665] "ENSGACG00000037901" "ENSGACG00000006391" "ENSGACG00000006434"
## [20668] "ENSGACG00000006460" "ENSGACG00000006492" "ENSGACG00000006500"
## [20671] "ENSGACG00000006530" "ENSGACG00000006543" "ENSGACG00000037504"
## [20674] "ENSGACG00000006558" "ENSGACG00000006563" "ENSGACG00000006575"
## [20677] "ENSGACG00000006580" "ENSGACG00000006595" "ENSGACG00000006599"
## [20680] "ENSGACG00000006608" "ENSGACG00000006611" "ENSGACG00000006648"
## [20683] "ENSGACG00000024007" "ENSGACG00000006663" "ENSGACG00000006673"
## [20686] "ENSGACG00000006726" "ENSGACG00000006739" "ENSGACG00000006765"
## [20689] "ENSGACG00000026864" "ENSGACG00000006801" "ENSGACG00000006814"
## [20692] "ENSGACG00000006837" "ENSGACG00000006847" "ENSGACG00000009311"
## [20695] "ENSGACG00000030950" "ENSGACG00000029927" "ENSGACG00000009286"
## [20698] "ENSGACG00000009266" "ENSGACG00000009253" "ENSGACG00000009244"
## [20701] "ENSGACG00000009226" "ENSGACG00000034160" "ENSGACG00000009221"
## [20704] "ENSGACG00000033060" "ENSGACG00000009213" "ENSGACG00000009191"
## [20707] "ENSGACG00000009162" "ENSGACG00000009152" "ENSGACG00000009145"
## [20710] "ENSGACG00000009130" "ENSGACG00000009127" "ENSGACG00000014505"
## [20713] "ENSGACG00000031484" "ENSGACG00000014487" "ENSGACG00000014480"
## [20716] "ENSGACG00000014474" "ENSGACG00000014468" "ENSGACG00000014461"
## [20719] "ENSGACG00000014458" "ENSGACG00000037284" "ENSGACG00000014451"
## [20722] "ENSGACG00000032392" "ENSGACG00000023473" "ENSGACG00000014441"
## [20725] "ENSGACG00000025764" "ENSGACG00000014432" "ENSGACG00000014425"
## [20728] "ENSGACG00000014409" "ENSGACG00000026147" "ENSGACG00000014399"
## [20731] "ENSGACG00000031360" "ENSGACG00000031855" "ENSGACG00000014314"
## [20734] "ENSGACG00000014383" "ENSGACG00000014356" "ENSGACG00000014344"
## [20737] "ENSGACG00000014342" "ENSGACG00000014336" "ENSGACG00000014310"
## [20740] "ENSGACG00000035548" "ENSGACG00000027903" "ENSGACG00000009981"
## [20743] "ENSGACG00000025134" "ENSGACG00000037795" "ENSGACG00000010001"
## [20746] "ENSGACG00000031326" "ENSGACG00000010053" "ENSGACG00000010068"
## [20749] "ENSGACG00000036281" "ENSGACG00000010073" "ENSGACG00000010087"
## [20752] "ENSGACG00000010361" "ENSGACG00000010352" "ENSGACG00000010347"
## [20755] "ENSGACG00000010335" "ENSGACG00000010332" "ENSGACG00000010285"
## [20758] "ENSGACG00000010282" "ENSGACG00000010279" "ENSGACG00000010271"
## [20761] "ENSGACG00000010264" "ENSGACG00000030214" "ENSGACG00000028187"
## [20764] "ENSGACG00000010248" "ENSGACG00000010237" "ENSGACG00000010227"
## [20767] "ENSGACG00000010205" "ENSGACG00000010146" "ENSGACG00000010135"
## [20770] "ENSGACG00000036371" "ENSGACG00000015195" "ENSGACG00000015202"
## [20773] "ENSGACG00000010130" "ENSGACG00000024104" "ENSGACG00000015214"
## [20776] "ENSGACG00000015215" "ENSGACG00000010120" "ENSGACG00000015227"
## [20779] "ENSGACG00000015228" "ENSGACG00000015241" "ENSGACG00000025703"
## [20782] "ENSGACG00000004559" "ENSGACG00000037028" "ENSGACG00000004545"
## [20785] "ENSGACG00000004490" "ENSGACG00000010103" "ENSGACG00000010100"
## [20788] "ENSGACG00000033080" "ENSGACG00000029136" "ENSGACG00000004483"
## [20791] "ENSGACG00000004476" "ENSGACG00000037019" "ENSGACG00000004472"
## [20794] "ENSGACG00000007172" "ENSGACG00000007182" "ENSGACG00000004465"
## [20797] "ENSGACG00000031668" "ENSGACG00000007222" "ENSGACG00000025882"
## [20800] "ENSGACG00000025103" "ENSGACG00000037654" "ENSGACG00000028226"
## [20803] "ENSGACG00000023159" "ENSGACG00000004442" "ENSGACG00000004432"
## [20806] "ENSGACG00000027788" "ENSGACG00000004415" "ENSGACG00000033856"
## [20809] "ENSGACG00000002542" "ENSGACG00000002535" "ENSGACG00000007245"
## [20812] "ENSGACG00000002532" "ENSGACG00000007364" "ENSGACG00000023277"
## [20815] "ENSGACG00000036831" "ENSGACG00000002138" "ENSGACG00000007397"
## [20818] "ENSGACG00000036179" "ENSGACG00000037523" "ENSGACG00000002146"
## [20821] "ENSGACG00000007411" "ENSGACG00000002199" "ENSGACG00000007427"
## [20824] "ENSGACG00000007438" "ENSGACG00000002204" "ENSGACG00000007446"
## [20827] "ENSGACG00000007458" "ENSGACG00000007513" "ENSGACG00000037797"
## [20830] "ENSGACG00000007521" "ENSGACG00000007554" "ENSGACG00000007558"
## [20833] "ENSGACG00000007577" "ENSGACG00000002225" "ENSGACG00000007584"
## [20836] "ENSGACG00000002243" "ENSGACG00000002260" "ENSGACG00000007591"
## [20839] "ENSGACG00000002290" "ENSGACG00000007615" "ENSGACG00000002295"
## [20842] "ENSGACG00000033891" "ENSGACG00000002302" "ENSGACG00000007646"
## [20845] "ENSGACG00000007652" "ENSGACG00000007673" "ENSGACG00000007675"
## [20848] "ENSGACG00000007727" "ENSGACG00000037610" "ENSGACG00000007789"
## [20851] "ENSGACG00000030792" "ENSGACG00000007803" "ENSGACG00000027043"
## [20854] "ENSGACG00000007820" "ENSGACG00000002332" "ENSGACG00000007839"
## [20857] "ENSGACG00000007849" "ENSGACG00000033850" "ENSGACG00000007901"
## [20860] "ENSGACG00000002345" "ENSGACG00000035458" "ENSGACG00000002354"
## [20863] "ENSGACG00000033113" "ENSGACG00000002377" "ENSGACG00000007920"
## [20866] "ENSGACG00000030763" "ENSGACG00000003486" "ENSGACG00000007945"
## [20869] "ENSGACG00000003479" "ENSGACG00000037251" "ENSGACG00000023233"
## [20872] "ENSGACG00000007972" "ENSGACG00000003470" "ENSGACG00000003463"
## [20875] "ENSGACG00000003455" "ENSGACG00000003442" "ENSGACG00000007987"
## [20878] "ENSGACG00000003434" "ENSGACG00000003361" "ENSGACG00000008003"
## [20881] "ENSGACG00000008009" "ENSGACG00000008014" "ENSGACG00000008019"
## [20884] "ENSGACG00000036497" "ENSGACG00000008054" "ENSGACG00000008063"
## [20887] "ENSGACG00000024316" "ENSGACG00000008065" "ENSGACG00000025214"
## [20890] "ENSGACG00000031516" "ENSGACG00000008093" "ENSGACG00000008100"
## [20893] "ENSGACG00000008110" "ENSGACG00000008187" "ENSGACG00000031469"
## [20896] "ENSGACG00000031719" "ENSGACG00000023659" "ENSGACG00000037383"
## [20899] "ENSGACG00000025628" "ENSGACG00000031145" "ENSGACG00000034640"
## [20902] "ENSGACG00000035010" "ENSGACG00000024924" "ENSGACG00000033188"
## [20905] "ENSGACG00000036985" "ENSGACG00000029353" "ENSGACG00000024880"
## [20908] "ENSGACG00000024127" "ENSGACG00000028326" "ENSGACG00000037044"
## [20911] "ENSGACG00000031223" "ENSGACG00000026074" "ENSGACG00000023385"
## [20914] "ENSGACG00000025126" "ENSGACG00000007899" "ENSGACG00000033390"
## [20917] "ENSGACG00000025900" "ENSGACG00000034502" "ENSGACG00000025128"
## [20920] "ENSGACG00000031901" "ENSGACG00000026426" "ENSGACG00000026352"
## [20923] "ENSGACG00000035996" "ENSGACG00000024128" "ENSGACG00000024965"
## [20926] "ENSGACG00000026301" "ENSGACG00000024603" "ENSGACG00000032603"
## [20929] "ENSGACG00000034857" "ENSGACG00000028596" "ENSGACG00000030404"
## [20932] "ENSGACG00000034447" "ENSGACG00000024949" "ENSGACG00000037476"
## [20935] "ENSGACG00000030703" "ENSGACG00000026083" "ENSGACG00000022989"
## [20938] "ENSGACG00000028961" "ENSGACG00000008232" "ENSGACG00000008278"
## [20941] "ENSGACG00000008370" "ENSGACG00000008490" "ENSGACG00000008933"
## [20944] "ENSGACG00000027196" "ENSGACG00000025422" "ENSGACG00000031525"
## [20947] "ENSGACG00000006246" "ENSGACG00000014466" "ENSGACG00000037364"
## [20950] "ENSGACG00000023187" "ENSGACG00000007235" "ENSGACG00000008073"
## [20953] "ENSGACG00000036894" "ENSGACG00000003412" "ENSGACG00000003356"
## [20956] "ENSGACG00000003345" "ENSGACG00000003343" "ENSGACG00000003340"
## [20959] "ENSGACG00000029995" "ENSGACG00000034902" "ENSGACG00000025373"
## [20962] "ENSGACG00000037276" "ENSGACG00000003321" "ENSGACG00000002600"
## [20965] "ENSGACG00000002597" "ENSGACG00000036111" "ENSGACG00000004411"
## [20968] "ENSGACG00000035587" "ENSGACG00000031981" "ENSGACG00000004388"
## [20971] "ENSGACG00000004377" "ENSGACG00000004362" "ENSGACG00000004343"
## [20974] "ENSGACG00000004334" "ENSGACG00000004305" "ENSGACG00000004288"
## [20977] "ENSGACG00000004272" "ENSGACG00000004258" "ENSGACG00000004243"
## [20980] "ENSGACG00000004225" "ENSGACG00000004220" "ENSGACG00000030327"
## [20983] "ENSGACG00000031164" "ENSGACG00000031419" "ENSGACG00000027356"
## [20986] "ENSGACG00000004179" "ENSGACG00000004175" "ENSGACG00000004173"
## [20989] "ENSGACG00000004164" "ENSGACG00000004150" "ENSGACG00000004139"
## [20992] "ENSGACG00000004121" "ENSGACG00000004134" "ENSGACG00000024032"
## [20995] "ENSGACG00000004095" "ENSGACG00000004091" "ENSGACG00000002593"
## [20998] "ENSGACG00000002578" "ENSGACG00000002546" "ENSGACG00000002782"
## [21001] "ENSGACG00000023890" "ENSGACG00000002773" "ENSGACG00000002772"
## [21004] "ENSGACG00000002770" "ENSGACG00000002745" "ENSGACG00000002737"
## [21007] "ENSGACG00000002728" "ENSGACG00000002720" "ENSGACG00000015190"
## [21010] "ENSGACG00000030814" "ENSGACG00000002691" "ENSGACG00000002687"
## [21013] "ENSGACG00000002681" "ENSGACG00000002668" "ENSGACG00000002663"
## [21016] "ENSGACG00000002658" "ENSGACG00000002640" "ENSGACG00000002652"
## [21019] "ENSGACG00000002628" "ENSGACG00000002621" "ENSGACG00000002614"
## [21022] "ENSGACG00000002608" "ENSGACG00000005933" "ENSGACG00000005901"
## [21025] "ENSGACG00000029327" "ENSGACG00000005740" "ENSGACG00000005706"
## [21028] "ENSGACG00000005702" "ENSGACG00000005699" "ENSGACG00000005691"
## [21031] "ENSGACG00000036084" "ENSGACG00000005687" "ENSGACG00000005675"
## [21034] "ENSGACG00000028570" "ENSGACG00000028355" "ENSGACG00000005641"
## [21037] "ENSGACG00000037584" "ENSGACG00000005624" "ENSGACG00000005620"
## [21040] "ENSGACG00000005614" "ENSGACG00000005583" "ENSGACG00000005578"
## [21043] "ENSGACG00000005572" "ENSGACG00000005546" "ENSGACG00000005506"
## [21046] "ENSGACG00000005496" "ENSGACG00000033932" "ENSGACG00000005479"
## [21049] "ENSGACG00000005456" "ENSGACG00000005453" "ENSGACG00000031140"
## [21052] "ENSGACG00000005342" "ENSGACG00000005323" "ENSGACG00000005315"
## [21055] "ENSGACG00000032587" "ENSGACG00000005292" "ENSGACG00000005278"
## [21058] "ENSGACG00000005275" "ENSGACG00000005244" "ENSGACG00000005231"
## [21061] "ENSGACG00000005154" "ENSGACG00000005142" "ENSGACG00000005127"
## [21064] "ENSGACG00000005106" "ENSGACG00000005102" "ENSGACG00000005090"
## [21067] "ENSGACG00000031453" "ENSGACG00000005073" "ENSGACG00000005054"
## [21070] "ENSGACG00000005046" "ENSGACG00000030808" "ENSGACG00000005031"
## [21073] "ENSGACG00000037569" "ENSGACG00000025712" "ENSGACG00000005014"
## [21076] "ENSGACG00000035796" "ENSGACG00000029604" "ENSGACG00000004995"
## [21079] "ENSGACG00000004974" "ENSGACG00000004961" "ENSGACG00000004948"
## [21082] "ENSGACG00000004937" "ENSGACG00000004915" "ENSGACG00000004908"
## [21085] "ENSGACG00000004906" "ENSGACG00000004900" "ENSGACG00000026539"
## [21088] "ENSGACG00000024846" "ENSGACG00000029905" "ENSGACG00000004868"
## [21091] "ENSGACG00000037637" "ENSGACG00000026173" "ENSGACG00000004855"
## [21094] "ENSGACG00000003119" "ENSGACG00000004836" "ENSGACG00000003139"
## [21097] "ENSGACG00000003122" "ENSGACG00000003142" "ENSGACG00000026587"
## [21100] "ENSGACG00000015243" "ENSGACG00000024962" "ENSGACG00000015247"
## [21103] "ENSGACG00000015256" "ENSGACG00000015258" "ENSGACG00000015266"
## [21106] "ENSGACG00000015269" "ENSGACG00000015280" "ENSGACG00000015288"
## [21109] "ENSGACG00000033631" "ENSGACG00000015291" "ENSGACG00000015293"
## [21112] "ENSGACG00000015304" "ENSGACG00000023706" "ENSGACG00000028591"
## [21115] "ENSGACG00000015314" "ENSGACG00000015318" "ENSGACG00000015320"
## [21118] "ENSGACG00000028435" "ENSGACG00000015329" "ENSGACG00000015334"
## [21121] "ENSGACG00000027198" "ENSGACG00000015342" "ENSGACG00000015343"
## [21124] "ENSGACG00000015347" "ENSGACG00000015360" "ENSGACG00000015362"
## [21127] "ENSGACG00000023867" "ENSGACG00000015370" "ENSGACG00000015379"
## [21130] "ENSGACG00000004829" "ENSGACG00000004821" "ENSGACG00000004810"
## [21133] "ENSGACG00000031768" "ENSGACG00000024832" "ENSGACG00000015389"
## [21136] "ENSGACG00000015393" "ENSGACG00000015394" "ENSGACG00000004799"
## [21139] "ENSGACG00000004793" "ENSGACG00000015395" "ENSGACG00000025017"
## [21142] "ENSGACG00000004686" "ENSGACG00000015401" "ENSGACG00000023745"
## [21145] "ENSGACG00000004649" "ENSGACG00000026552" "ENSGACG00000015405"
## [21148] "ENSGACG00000004643" "ENSGACG00000004637" "ENSGACG00000015408"
## [21151] "ENSGACG00000004626" "ENSGACG00000023307" "ENSGACG00000004617"
## [21154] "ENSGACG00000015413" "ENSGACG00000004601" "ENSGACG00000034743"
## [21157] "ENSGACG00000004592" "ENSGACG00000033687" "ENSGACG00000004573"
## [21160] "ENSGACG00000004568" "ENSGACG00000015427" "ENSGACG00000015436"
## [21163] "ENSGACG00000003305" "ENSGACG00000003300" "ENSGACG00000032351"
## [21166] "ENSGACG00000003281" "ENSGACG00000003272" "ENSGACG00000003268"
## [21169] "ENSGACG00000003256" "ENSGACG00000003231" "ENSGACG00000003227"
## [21172] "ENSGACG00000033378" "ENSGACG00000027293" "ENSGACG00000003215"
## [21175] "ENSGACG00000003206" "ENSGACG00000003179" "ENSGACG00000003168"
## [21178] "ENSGACG00000003162" "ENSGACG00000032475" "ENSGACG00000003149"
## [21181] "ENSGACG00000003091" "ENSGACG00000003106" "ENSGACG00000003105"
## [21184] "ENSGACG00000003086" "ENSGACG00000003070" "ENSGACG00000003049"
## [21187] "ENSGACG00000003028" "ENSGACG00000002991" "ENSGACG00000002978"
## [21190] "ENSGACG00000023951" "ENSGACG00000002967" "ENSGACG00000002925"
## [21193] "ENSGACG00000002915" "ENSGACG00000002901" "ENSGACG00000002908"
## [21196] "ENSGACG00000030255" "ENSGACG00000002892" "ENSGACG00000028382"
## [21199] "ENSGACG00000002883" "ENSGACG00000002522" "ENSGACG00000002521"
## [21202] "ENSGACG00000002502" "ENSGACG00000002500" "ENSGACG00000037404"
## [21205] "ENSGACG00000002492" "ENSGACG00000028525" "ENSGACG00000002871"
## [21208] "ENSGACG00000002868" "ENSGACG00000002861" "ENSGACG00000002856"
## [21211] "ENSGACG00000002849" "ENSGACG00000002813" "ENSGACG00000024047"
## [21214] "ENSGACG00000002801" "ENSGACG00000002799" "ENSGACG00000002795"
## [21217] "ENSGACG00000002486" "ENSGACG00000002482" "ENSGACG00000002465"
## [21220] "ENSGACG00000002464" "ENSGACG00000002462" "ENSGACG00000002440"
## [21223] "ENSGACG00000030416" "ENSGACG00000002432" "ENSGACG00000002428"
## [21226] "ENSGACG00000002420" "ENSGACG00000002414" "ENSGACG00000002397"
## [21229] "ENSGACG00000002386" "ENSGACG00000030644" "ENSGACG00000002375"
## [21232] "ENSGACG00000002371" "ENSGACG00000004081" "ENSGACG00000004047"
## [21235] "ENSGACG00000004035" "ENSGACG00000004027" "ENSGACG00000024070"
## [21238] "ENSGACG00000003993" "ENSGACG00000003985" "ENSGACG00000003963"
## [21241] "ENSGACG00000003954" "ENSGACG00000003950" "ENSGACG00000028720"
## [21244] "ENSGACG00000003942" "ENSGACG00000003939" "ENSGACG00000003929"
## [21247] "ENSGACG00000024247" "ENSGACG00000026574" "ENSGACG00000029147"
## [21250] "ENSGACG00000003904" "ENSGACG00000003875" "ENSGACG00000034221"
## [21253] "ENSGACG00000003855" "ENSGACG00000003841" "ENSGACG00000003833"
## [21256] "ENSGACG00000003829" "ENSGACG00000003822" "ENSGACG00000003817"
## [21259] "ENSGACG00000003807" "ENSGACG00000003795" "ENSGACG00000003786"
## [21262] "ENSGACG00000028701" "ENSGACG00000003772" "ENSGACG00000003766"
## [21265] "ENSGACG00000003736" "ENSGACG00000003724" "ENSGACG00000003722"
## [21268] "ENSGACG00000034061" "ENSGACG00000036623" "ENSGACG00000030779"
## [21271] "ENSGACG00000003707" "ENSGACG00000003702" "ENSGACG00000003681"
## [21274] "ENSGACG00000035187" "ENSGACG00000003635" "ENSGACG00000003563"
## [21277] "ENSGACG00000003561" "ENSGACG00000003547" "ENSGACG00000003544"
## [21280] "ENSGACG00000003531" "ENSGACG00000003521" "ENSGACG00000027103"
## [21283] "ENSGACG00000030247" "ENSGACG00000003511" "ENSGACG00000003508"
## [21286] "ENSGACG00000003504" "ENSGACG00000028212" "ENSGACG00000003493"
## [21289] "ENSGACG00000031724" "ENSGACG00000024902" "ENSGACG00000036581"
## [21292] "ENSGACG00000031988" "ENSGACG00000024604" "ENSGACG00000034785"
## [21295] "ENSGACG00000025124" "ENSGACG00000036600" "ENSGACG00000032246"
## [21298] "ENSGACG00000023457" "ENSGACG00000025078" "ENSGACG00000028791"
## [21301] "ENSGACG00000023428" "ENSGACG00000035360" "ENSGACG00000036326"
## [21304] "ENSGACG00000023936" "ENSGACG00000030095" "ENSGACG00000035805"
## [21307] "ENSGACG00000032588" "ENSGACG00000024778" "ENSGACG00000028159"
## [21310] "ENSGACG00000036573" "ENSGACG00000033452" "ENSGACG00000031799"
## [21313] "ENSGACG00000003651" "ENSGACG00000035935" "ENSGACG00000028331"
## [21316] "ENSGACG00000024173" "ENSGACG00000035402" "ENSGACG00000024582"
## [21319] "ENSGACG00000025308" "ENSGACG00000034548" "ENSGACG00000031347"
## [21322] "ENSGACG00000026721" "ENSGACG00000036776" "ENSGACG00000027782"
## [21325] "ENSGACG00000024830" "ENSGACG00000025437" "ENSGACG00000034479"
## [21328] "ENSGACG00000033537" "ENSGACG00000037542" "ENSGACG00000032666"
## [21331] "ENSGACG00000027298" "ENSGACG00000036135" "ENSGACG00000024576"
## [21334] "ENSGACG00000028544" "ENSGACG00000023934" "ENSGACG00000023128"
## [21337] "ENSGACG00000024482" "ENSGACG00000029152" "ENSGACG00000029638"
## [21340] "ENSGACG00000027119" "ENSGACG00000025457" "ENSGACG00000023521"
## [21343] "ENSGACG00000005441" "ENSGACG00000003113" "ENSGACG00000036185"
## [21346] "ENSGACG00000036015" "ENSGACG00000003750" "ENSGACG00000030047"
## [21349] "ENSGACG00000001727" "ENSGACG00000032623" "ENSGACG00000032831"
## [21352] "ENSGACG00000013627" "ENSGACG00000023515" "ENSGACG00000013637"
## [21355] "ENSGACG00000013654" "ENSGACG00000013590" "ENSGACG00000034591"
## [21358] "ENSGACG00000013664" "ENSGACG00000013671" "ENSGACG00000013674"
## [21361] "ENSGACG00000013698" "ENSGACG00000035793" "ENSGACG00000030821"
## [21364] "ENSGACG00000025368" "ENSGACG00000010918" "ENSGACG00000010979"
## [21367] "ENSGACG00000029371" "ENSGACG00000010991" "ENSGACG00000011012"
## [21370] "ENSGACG00000011045" "ENSGACG00000011064" "ENSGACG00000036569"
## [21373] "ENSGACG00000011069" "ENSGACG00000011087" "ENSGACG00000011091"
## [21376] "ENSGACG00000032734" "ENSGACG00000011116" "ENSGACG00000000796"
## [21379] "ENSGACG00000027144" "ENSGACG00000000800" "ENSGACG00000024795"
## [21382] "ENSGACG00000023665" "ENSGACG00000025230" "ENSGACG00000000548"
## [21385] "ENSGACG00000036209" "ENSGACG00000035250" "ENSGACG00000025339"
## [21388] "ENSGACG00000033796" "ENSGACG00000026683" "ENSGACG00000030036"
## [21391] "ENSGACG00000030636" "ENSGACG00000035991" "ENSGACG00000010510"
## [21394] "ENSGACG00000010536" "ENSGACG00000010553" "ENSGACG00000010563"
## [21397] "ENSGACG00000010569" "ENSGACG00000036075" "ENSGACG00000012558"
## [21400] "ENSGACG00000032489" "ENSGACG00000038016" "ENSGACG00000012548"
## [21403] "ENSGACG00000035942" "ENSGACG00000037471" "ENSGACG00000031343"
## [21406] "ENSGACG00000036847" "ENSGACG00000030311" "ENSGACG00000037425"
## [21409] "ENSGACG00000026363" "ENSGACG00000035525" "ENSGACG00000014663"
## [21412] "ENSGACG00000014665" "ENSGACG00000014681" "ENSGACG00000026729"
## [21415] "ENSGACG00000000805" "ENSGACG00000000810" "ENSGACG00000038043"
## [21418] "ENSGACG00000025814" "ENSGACG00000025260" "ENSGACG00000025776"
## [21421] "ENSGACG00000025521" "ENSGACG00000015731" "ENSGACG00000015738"
## [21424] "ENSGACG00000033248" "ENSGACG00000023217" "ENSGACG00000027682"
## [21427] "ENSGACG00000032046" "ENSGACG00000000973" "ENSGACG00000000991"
## [21430] "ENSGACG00000001007" "ENSGACG00000030167" "ENSGACG00000013430"
## [21433] "ENSGACG00000013435" "ENSGACG00000013440" "ENSGACG00000013442"
## [21436] "ENSGACG00000013445" "ENSGACG00000037376" "ENSGACG00000035230"
## [21439] "ENSGACG00000036393" "ENSGACG00000001575" "ENSGACG00000034448"
## [21442] "ENSGACG00000001582" "ENSGACG00000026245" "ENSGACG00000035765"
## [21445] "ENSGACG00000029987" "ENSGACG00000032514" "ENSGACG00000033076"
## [21448] "ENSGACG00000030492" "ENSGACG00000036976" "ENSGACG00000036940"
## [21451] "ENSGACG00000000043" "ENSGACG00000038025" "ENSGACG00000000816"
## [21454] "ENSGACG00000000818" "ENSGACG00000036451" "ENSGACG00000023478"
## [21457] "ENSGACG00000029681" "ENSGACG00000035838" "ENSGACG00000026264"
## [21460] "ENSGACG00000001757" "ENSGACG00000034701" "ENSGACG00000024829"
## [21463] "ENSGACG00000026993" "ENSGACG00000034918" "ENSGACG00000013769"
## [21466] "ENSGACG00000033662" "ENSGACG00000031187" "ENSGACG00000001440"
## [21469] "ENSGACG00000001465" "ENSGACG00000001470" "ENSGACG00000035047"
## [21472] "ENSGACG00000001502" "ENSGACG00000027515" "ENSGACG00000023053"
## [21475] "ENSGACG00000001458" "ENSGACG00000001459" "ENSGACG00000001467"
## [21478] "ENSGACG00000001474" "ENSGACG00000025475" "ENSGACG00000028086"
## [21481] "ENSGACG00000036684" "ENSGACG00000030888" "ENSGACG00000036035"
## [21484] "ENSGACG00000026386" "ENSGACG00000024193" "ENSGACG00000000162"
## [21487] "ENSGACG00000024860" "ENSGACG00000031906" "ENSGACG00000001032"
## [21490] "ENSGACG00000000002" "ENSGACG00000031141" "ENSGACG00000029698"
## [21493] "ENSGACG00000034111" "ENSGACG00000029020" "ENSGACG00000035361"
## [21496] "ENSGACG00000033465" "ENSGACG00000000227" "ENSGACG00000000228"
## [21499] "ENSGACG00000000232" "ENSGACG00000029441" "ENSGACG00000035533"
## [21502] "ENSGACG00000013478" "ENSGACG00000013479" "ENSGACG00000028274"
## [21505] "ENSGACG00000015818" "ENSGACG00000012879" "ENSGACG00000012892"
## [21508] "ENSGACG00000012906" "ENSGACG00000012915" "ENSGACG00000012926"
## [21511] "ENSGACG00000027105" "ENSGACG00000025821" "ENSGACG00000002042"
## [21514] "ENSGACG00000025469" "ENSGACG00000001376" "ENSGACG00000032948"
## [21517] "ENSGACG00000023299" "ENSGACG00000027272" "ENSGACG00000026561"
## [21520] "ENSGACG00000015664" "ENSGACG00000031989" "ENSGACG00000026965"
## [21523] "ENSGACG00000026017" "ENSGACG00000028071" "ENSGACG00000027174"
## [21526] "ENSGACG00000026524" "ENSGACG00000019723" "ENSGACG00000001051"
## [21529] "ENSGACG00000001290" "ENSGACG00000001320" "ENSGACG00000035596"
## [21532] "ENSGACG00000030692" "ENSGACG00000026149" "ENSGACG00000034232"
## [21535] "ENSGACG00000027462" "ENSGACG00000034430" "ENSGACG00000026888"
## [21538] "ENSGACG00000026572" "ENSGACG00000030033" "ENSGACG00000025907"
## [21541] "ENSGACG00000034796" "ENSGACG00000025937" "ENSGACG00000031930"
## [21544] "ENSGACG00000032817" "ENSGACG00000004894" "ENSGACG00000035270"
## [21547] "ENSGACG00000033606" "ENSGACG00000024906" "ENSGACG00000026880"
## [21550] "ENSGACG00000035042" "ENSGACG00000024505" "ENSGACG00000030656"
## [21553] "ENSGACG00000031405" "ENSGACG00000001186" "ENSGACG00000025141"
## [21556] "ENSGACG00000001190" "ENSGACG00000001648" "ENSGACG00000001840"
## [21559] "ENSGACG00000029054" "ENSGACG00000024030" "ENSGACG00000024757"
## [21562] "ENSGACG00000032097" "ENSGACG00000032423" "ENSGACG00000001945"
## [21565] "ENSGACG00000029826" "ENSGACG00000023712" "ENSGACG00000035941"
## [21568] "ENSGACG00000000231" "ENSGACG00000000229" "ENSGACG00000035772"
## [21571] "ENSGACG00000026513" "ENSGACG00000036151" "ENSGACG00000026472"
## [21574] "ENSGACG00000031148" "ENSGACG00000029545" "ENSGACG00000026275"
## [21577] "ENSGACG00000036034" "ENSGACG00000026631" "ENSGACG00000035752"
## [21580] "ENSGACG00000030397" "ENSGACG00000032355" "ENSGACG00000012841"
## [21583] "ENSGACG00000012853" "ENSGACG00000001849" "ENSGACG00000001853"
## [21586] "ENSGACG00000031983" "ENSGACG00000036059" "ENSGACG00000015706"
## [21589] "ENSGACG00000028156" "ENSGACG00000032544" "ENSGACG00000026166"
## [21592] "ENSGACG00000025617" "ENSGACG00000023407" "ENSGACG00000030668"
## [21595] "ENSGACG00000036189" "ENSGACG00000000525" "ENSGACG00000030882"
## [21598] "ENSGACG00000028752" "ENSGACG00000025854" "ENSGACG00000000032"
## [21601] "ENSGACG00000029817" "ENSGACG00000026212" "ENSGACG00000000237"
## [21604] "ENSGACG00000001152" "ENSGACG00000028089" "ENSGACG00000000166"
## [21607] "ENSGACG00000032010" "ENSGACG00000030076" "ENSGACG00000029149"
## [21610] "ENSGACG00000037574" "ENSGACG00000036396" "ENSGACG00000033351"
## [21613] "ENSGACG00000024602" "ENSGACG00000014561" "ENSGACG00000014563"
## [21616] "ENSGACG00000029508" "ENSGACG00000027039" "ENSGACG00000036897"
## [21619] "ENSGACG00000030759" "ENSGACG00000023274" "ENSGACG00000001198"
## [21622] "ENSGACG00000014824" "ENSGACG00000014826" "ENSGACG00000027847"
## [21625] "ENSGACG00000035112" "ENSGACG00000035323" "ENSGACG00000014548"
## [21628] "ENSGACG00000014551" "ENSGACG00000030851" "ENSGACG00000024683"
## [21631] "ENSGACG00000023059" "ENSGACG00000029119" "ENSGACG00000031267"
## [21634] "ENSGACG00000001887" "ENSGACG00000001890" "ENSGACG00000001897"
## [21637] "ENSGACG00000001906" "ENSGACG00000001413" "ENSGACG00000000034"
## [21640] "ENSGACG00000025478" "ENSGACG00000001142" "ENSGACG00000001145"
## [21643] "ENSGACG00000032931" "ENSGACG00000027558" "ENSGACG00000013808"
## [21646] "ENSGACG00000013813" "ENSGACG00000027810" "ENSGACG00000032275"
## [21649] "ENSGACG00000031911" "ENSGACG00000025815" "ENSGACG00000035194"
## [21652] "ENSGACG00000028317" "ENSGACG00000034080" "ENSGACG00000032448"
## [21655] "ENSGACG00000032930" "ENSGACG00000031379" "ENSGACG00000000971"
## [21658] "ENSGACG00000028774" "ENSGACG00000027437" "ENSGACG00000011237"
## [21661] "ENSGACG00000013799" "ENSGACG00000037209" "ENSGACG00000027326"
## [21664] "ENSGACG00000028736" "ENSGACG00000028428" "ENSGACG00000030413"
## [21667] "ENSGACG00000030000" "ENSGACG00000001357" "ENSGACG00000035771"
## [21670] "ENSGACG00000032606" "ENSGACG00000037243" "ENSGACG00000001258"
## [21673] "ENSGACG00000029430" "ENSGACG00000023991" "ENSGACG00000015722"
## [21676] "ENSGACG00000015724" "ENSGACG00000031526" "ENSGACG00000001681"
## [21679] "ENSGACG00000035717" "ENSGACG00000001689" "ENSGACG00000033752"
## [21682] "ENSGACG00000036005" "ENSGACG00000002300" "ENSGACG00000037768"
## [21685] "ENSGACG00000028397" "ENSGACG00000037678" "ENSGACG00000014246"
## [21688] "ENSGACG00000029293" "ENSGACG00000010480" "ENSGACG00000031310"
## [21691] "ENSGACG00000001153" "ENSGACG00000035487" "ENSGACG00000037256"
## [21694] "ENSGACG00000030798" "ENSGACG00000010430" "ENSGACG00000024101"
## [21697] "ENSGACG00000032212" "ENSGACG00000028619" "ENSGACG00000033492"
## [21700] "ENSGACG00000032111" "ENSGACG00000013713" "ENSGACG00000025966"
## [21703] "ENSGACG00000002590" "ENSGACG00000032053" "ENSGACG00000032388"
## [21706] "ENSGACG00000037493" "ENSGACG00000020919" "ENSGACG00000034882"
## [21709] "ENSGACG00000022922" "ENSGACG00000001630" "ENSGACG00000000794"
## [21712] "ENSGACG00000032525" "ENSGACG00000000029" "ENSGACG00000035944"
## [21715] "ENSGACG00000001690" "ENSGACG00000034084" "ENSGACG00000024845"
## [21718] "ENSGACG00000001366" "ENSGACG00000028880" "ENSGACG00000028041"
## [21721] "ENSGACG00000001807" "ENSGACG00000027767" "ENSGACG00000024108"
## [21724] "ENSGACG00000031807" "ENSGACG00000028964" "ENSGACG00000026946"
## [21727] "ENSGACG00000001970" "ENSGACG00000033893" "ENSGACG00000023634"
## [21730] "ENSGACG00000033085" "ENSGACG00000037532" "ENSGACG00000032462"
## [21733] "ENSGACG00000002037" "ENSGACG00000029379" "ENSGACG00000029903"
## [21736] "ENSGACG00000036354" "ENSGACG00000000216" "ENSGACG00000036045"
## [21739] "ENSGACG00000033181" "ENSGACG00000033904" "ENSGACG00000032041"
## [21742] "ENSGACG00000037081" "ENSGACG00000028047" "ENSGACG00000026408"
## [21745] "ENSGACG00000031231" "ENSGACG00000033635" "ENSGACG00000023889"
## [21748] "ENSGACG00000001026" "ENSGACG00000032151" "ENSGACG00000037541"
## [21751] "ENSGACG00000030027" "ENSGACG00000029392" "ENSGACG00000027302"
## [21754] "ENSGACG00000000875" "ENSGACG00000000880" "ENSGACG00000023087"
## [21757] "ENSGACG00000030836" "ENSGACG00000028356" "ENSGACG00000030431"
## [21760] "ENSGACG00000001697" "ENSGACG00000001706" "ENSGACG00000005629"
## [21763] "ENSGACG00000035790" "ENSGACG00000031638" "ENSGACG00000029413"
## [21766] "ENSGACG00000031646" "ENSGACG00000030840" "ENSGACG00000032985"
## [21769] "ENSGACG00000001163" "ENSGACG00000029970" "ENSGACG00000036422"
## [21772] "ENSGACG00000031528" "ENSGACG00000033337" "ENSGACG00000034269"
## [21775] "ENSGACG00000001350" "ENSGACG00000001872" "ENSGACG00000037573"
## [21778] "ENSGACG00000030504" "ENSGACG00000025587" "ENSGACG00000030834"
## [21781] "ENSGACG00000031377" "ENSGACG00000035968" "ENSGACG00000028697"
## [21784] "ENSGACG00000031915" "ENSGACG00000023637" "ENSGACG00000023186"
## [21787] "ENSGACG00000033005" "ENSGACG00000033632" "ENSGACG00000000408"
## [21790] "ENSGACG00000034573" "ENSGACG00000001184" "ENSGACG00000033175"
## [21793] "ENSGACG00000033065" "ENSGACG00000037229" "ENSGACG00000030176"
## [21796] "ENSGACG00000032145" "ENSGACG00000001845" "ENSGACG00000027836"
## [21799] "ENSGACG00000023734" "ENSGACG00000034030" "ENSGACG00000028398"
## [21802] "ENSGACG00000028979" "ENSGACG00000023560" "ENSGACG00000005693"
## [21805] "ENSGACG00000000256" "ENSGACG00000034210" "ENSGACG00000025439"
## [21808] "ENSGACG00000001445" "ENSGACG00000028620" "ENSGACG00000001118"
## [21811] "ENSGACG00000036293" "ENSGACG00000023279" "ENSGACG00000010486"
## [21814] "ENSGACG00000034095" "ENSGACG00000035355" "ENSGACG00000033349"
## [21817] "ENSGACG00000028334" "ENSGACG00000025187" "ENSGACG00000023455"
## [21820] "ENSGACG00000037512" "ENSGACG00000033167" "ENSGACG00000037940"
## [21823] "ENSGACG00000035740" "ENSGACG00000015699" "ENSGACG00000036905"
## [21826] "ENSGACG00000005580" "ENSGACG00000005586" "ENSGACG00000023912"
## [21829] "ENSGACG00000029901" "ENSGACG00000030341" "ENSGACG00000025489"
## [21832] "ENSGACG00000028378" "ENSGACG00000027965" "ENSGACG00000030120"
## [21835] "ENSGACG00000036829" "ENSGACG00000030777" "ENSGACG00000022924"
## [21838] "ENSGACG00000026255" "ENSGACG00000001015" "ENSGACG00000032129"
## [21841] "ENSGACG00000034667" "ENSGACG00000000389" "ENSGACG00000034020"
## [21844] "ENSGACG00000001201" "ENSGACG00000029359" "ENSGACG00000000258"
## [21847] "ENSGACG00000031701" "ENSGACG00000013387" "ENSGACG00000031022"
## [21850] "ENSGACG00000000948" "ENSGACG00000035751" "ENSGACG00000026785"
## [21853] "ENSGACG00000023647" "ENSGACG00000028799" "ENSGACG00000035417"
## [21856] "ENSGACG00000038054" "ENSGACG00000035016" "ENSGACG00000027796"
## [21859] "ENSGACG00000001960" "ENSGACG00000001964" "ENSGACG00000030261"
## [21862] "ENSGACG00000000185" "ENSGACG00000026625" "ENSGACG00000002278"
## [21865] "ENSGACG00000033670" "ENSGACG00000029333" "ENSGACG00000032231"
## [21868] "ENSGACG00000032188" "ENSGACG00000015772" "ENSGACG00000037384"
## [21871] "ENSGACG00000013454" "ENSGACG00000000254" "ENSGACG00000034293"
## [21874] "ENSGACG00000018692" "ENSGACG00000034128" "ENSGACG00000023942"
## [21877] "ENSGACG00000000123" "ENSGACG00000001174" "ENSGACG00000030626"
## [21880] "ENSGACG00000026267" "ENSGACG00000033609" "ENSGACG00000035297"
## [21883] "ENSGACG00000028101" "ENSGACG00000036599" "ENSGACG00000024750"
## [21886] "ENSGACG00000031994" "ENSGACG00000036659" "ENSGACG00000036865"
## [21889] "ENSGACG00000014730" "ENSGACG00000031973" "ENSGACG00000033638"
## [21892] "ENSGACG00000024216" "ENSGACG00000015690" "ENSGACG00000000378"
## [21895] "ENSGACG00000001279" "ENSGACG00000029870" "ENSGACG00000001255"
## [21898] "ENSGACG00000030369" "ENSGACG00000013791" "ENSGACG00000029501"
## [21901] "ENSGACG00000015524" "ENSGACG00000029286" "ENSGACG00000028841"
## [21904] "ENSGACG00000032250" "ENSGACG00000035639" "ENSGACG00000026535"
## [21907] "ENSGACG00000027819" "ENSGACG00000024338" "ENSGACG00000001335"
## [21910] "ENSGACG00000030602" "ENSGACG00000023327" "ENSGACG00000024210"
## [21913] "ENSGACG00000001127" "ENSGACG00000031716" "ENSGACG00000035165"
## [21916] "ENSGACG00000000164" "ENSGACG00000034200" "ENSGACG00000028053"
## [21919] "ENSGACG00000005728" "ENSGACG00000031154" "ENSGACG00000032685"
## [21922] "ENSGACG00000024235" "ENSGACG00000001775" "ENSGACG00000025579"
## [21925] "ENSGACG00000025396" "ENSGACG00000031861" "ENSGACG00000028890"
## [21928] "ENSGACG00000023051" "ENSGACG00000036105" "ENSGACG00000037088"
## [21931] "ENSGACG00000030111" "ENSGACG00000023535" "ENSGACG00000023642"
## [21934] "ENSGACG00000030866" "ENSGACG00000038061" "ENSGACG00000018067"
## [21937] "ENSGACG00000031680" "ENSGACG00000027608" "ENSGACG00000034586"
## [21940] "ENSGACG00000026321" "ENSGACG00000031508" "ENSGACG00000033906"
## [21943] "ENSGACG00000025620" "ENSGACG00000000049" "ENSGACG00000023958"
## [21946] "ENSGACG00000006210" "ENSGACG00000032088" "ENSGACG00000036344"
## [21949] "ENSGACG00000024811" "ENSGACG00000022998" "ENSGACG00000032357"
## [21952] "ENSGACG00000034133" "ENSGACG00000035844" "ENSGACG00000001850"
## [21955] "ENSGACG00000030751" "ENSGACG00000024889" "ENSGACG00000028941"
## [21958] "ENSGACG00000032261" "ENSGACG00000037712" "ENSGACG00000001642"
## [21961] "ENSGACG00000026339" "ENSGACG00000025694" "ENSGACG00000015711"
## [21964] "ENSGACG00000035231" "ENSGACG00000026490" "ENSGACG00000028990"
## [21967] "ENSGACG00000033388" "ENSGACG00000028448" "ENSGACG00000001511"
## [21970] "ENSGACG00000035431" "ENSGACG00000000176" "ENSGACG00000031367"
## [21973] "ENSGACG00000037442" "ENSGACG00000036386" "ENSGACG00000025276"
## [21976] "ENSGACG00000034460" "ENSGACG00000011229" "ENSGACG00000026126"
## [21979] "ENSGACG00000031689" "ENSGACG00000028763" "ENSGACG00000013376"
## [21982] "ENSGACG00000013378" "ENSGACG00000028148" "ENSGACG00000034163"
## [21985] "ENSGACG00000034818" "ENSGACG00000027875" "ENSGACG00000038050"
## [21988] "ENSGACG00000026957" "ENSGACG00000036943" "ENSGACG00000026632"
## [21991] "ENSGACG00000034375" "ENSGACG00000034327" "ENSGACG00000033907"
## [21994] "ENSGACG00000028542" "ENSGACG00000033706" "ENSGACG00000000910"
## [21997] "ENSGACG00000000116" "ENSGACG00000037516" "ENSGACG00000015822"
## [22000] "ENSGACG00000035644" "ENSGACG00000030370" "ENSGACG00000030845"
## [22003] "ENSGACG00000001187" "ENSGACG00000036526" "ENSGACG00000034823"
## [22006] "ENSGACG00000010855" "ENSGACG00000034332" "ENSGACG00000014749"
## [22009] "ENSGACG00000034876" "ENSGACG00000037242" "ENSGACG00000001570"
## [22012] "ENSGACG00000027595" "ENSGACG00000000177" "ENSGACG00000033564"
## [22015] "ENSGACG00000001653" "ENSGACG00000001859" "ENSGACG00000030359"
## [22018] "ENSGACG00000001494" "ENSGACG00000000982" "ENSGACG00000024195"
## [22021] "ENSGACG00000037171" "ENSGACG00000033277" "ENSGACG00000023544"
## [22024] "ENSGACG00000033617" "ENSGACG00000005692" "ENSGACG00000028444"
## [22027] "ENSGACG00000028892" "ENSGACG00000029412" "ENSGACG00000033103"
## [22030] "ENSGACG00000000186" "ENSGACG00000030599" "ENSGACG00000025851"
## [22033] "ENSGACG00000001793" "ENSGACG00000001794" "ENSGACG00000038033"
## [22036] "ENSGACG00000035953" "ENSGACG00000001713" "ENSGACG00000037253"
## [22039] "ENSGACG00000027439" "ENSGACG00000029538" "ENSGACG00000026542"
## [22042] "ENSGACG00000034287" "ENSGACG00000024363" "ENSGACG00000026818"
## [22045] "ENSGACG00000024802" "ENSGACG00000000263" "ENSGACG00000022262"
## [22048] "ENSGACG00000030180" "ENSGACG00000010903" "ENSGACG00000025533"
## [22051] "ENSGACG00000026414" "ENSGACG00000002179" "ENSGACG00000017397"
## [22054] "ENSGACG00000038064" "ENSGACG00000001039" "ENSGACG00000032173"
## [22057] "ENSGACG00000025572" "ENSGACG00000024154" "ENSGACG00000001421"
## [22060] "ENSGACG00000018689" "ENSGACG00000000595" "ENSGACG00000028737"
## [22063] "ENSGACG00000028014" "ENSGACG00000034494" "ENSGACG00000033748"
## [22066] "ENSGACG00000024882" "ENSGACG00000028150" "ENSGACG00000030672"
## [22069] "ENSGACG00000037765" "ENSGACG00000030338" "ENSGACG00000026377"
## [22072] "ENSGACG00000032534" "ENSGACG00000031003" "ENSGACG00000033052"
## [22075] "ENSGACG00000002036" "ENSGACG00000029666" "ENSGACG00000031730"
## [22078] "ENSGACG00000001572" "ENSGACG00000037737" "ENSGACG00000030500"
## [22081] "ENSGACG00000024470" "ENSGACG00000001228" "ENSGACG00000001355"
## [22084] "ENSGACG00000035551" "ENSGACG00000035641" "ENSGACG00000023451"
## [22087] "ENSGACG00000030989" "ENSGACG00000029724" "ENSGACG00000025164"
## [22090] "ENSGACG00000002049" "ENSGACG00000030682" "ENSGACG00000038132"
## [22093] "ENSGACG00000026207" "ENSGACG00000036402" "ENSGACG00000032573"
## [22096] "ENSGACG00000036318" "ENSGACG00000037482" "ENSGACG00000000163"
## [22099] "ENSGACG00000036333" "ENSGACG00000028649" "ENSGACG00000035885"
## [22102] "ENSGACG00000033239" "ENSGACG00000001759" "ENSGACG00000014518"
## [22105] "ENSGACG00000026756" "ENSGACG00000035828" "ENSGACG00000000040"
## [22108] "ENSGACG00000027351" "ENSGACG00000024031" "ENSGACG00000024687"
## [22111] "ENSGACG00000025806" "ENSGACG00000013716" "ENSGACG00000034140"
## [22114] "ENSGACG00000030665" "ENSGACG00000027735" "ENSGACG00000032998"
## [22117] "ENSGACG00000035060" "ENSGACG00000028946" "ENSGACG00000032100"
## [22120] "ENSGACG00000023704" "ENSGACG00000035965" "ENSGACG00000027535"
## [22123] "ENSGACG00000032494" "ENSGACG00000024239" "ENSGACG00000035977"
```

```
# Running the analysis with Wald test, followed by shrinkage of log2 fold change to aid visualization

dds <- DESeq(dds)
```

```
## estimating size factors
##   Note: levels of factors in the design contain characters other than
##   letters, numbers, '_' and '.'. It is recommended (but not required) to use
##   only letters, numbers, and delimiters '_' or '.', as these are safe characters
##   for column names in R. [This is a message, not a warning or an error]
```

```
## estimating dispersions
```

```
## gene-wise dispersion estimates
```

```
## mean-dispersion relationship
```

```
##   Note: levels of factors in the design contain characters other than
##   letters, numbers, '_' and '.'. It is recommended (but not required) to use
##   only letters, numbers, and delimiters '_' or '.', as these are safe characters
##   for column names in R. [This is a message, not a warning or an error]
```

```
## final dispersion estimates
```

```
## fitting model and testing
```

```
res <- results(dds, alpha = 0.05)
resultsNames(dds)
```

```
## [1] "Intercept"                              
## [2] "Batch_2_vs_1"                           
## [3] "Batch_3_vs_1"                           
## [4] "Batch_4_vs_1"                           
## [5] "Lake_type_Pike_vs_No.Pike"              
## [6] "Parent_state_Parenting_vs_Non.parenting"
```

```
# Variance stabilizing transformation to improve clustering visualization

vsd <- vst(dds, blind = FALSE)
```

## Visualization

```
# Plotting vsd plots, before and after vst transformation

ntd <- normTransform(dds)
meanSdPlot(assay(ntd))
```

```
## Warning: `aes_string()` was deprecated in ggplot2 3.0.0.
## ℹ Please use tidy evaluation idioms with `aes()`.
## ℹ See also `vignette("ggplot2-in-packages")` for more information.
## ℹ The deprecated feature was likely used in the vsn package.
##   Please report the issue to the authors.
## This warning is displayed once per session.
## Call `lifecycle::last_lifecycle_warnings()` to see where this warning was
## generated.
```

```
meanSdPlot(assay(vsd))
```

```
# PC1 and PC2 for Lake type, Batch, Parent state, and Parent state + Lake type

plotPCA(vsd, intgroup = "Lake_type")
```

```
## using ntop=500 top features by variance
```

```
plotPCA(vsd, intgroup = "Batch")
```

```
## using ntop=500 top features by variance
```

```
plotPCA(vsd, intgroup = "Parent_state")
```

```
## using ntop=500 top features by variance
```

```
plotPCA(vsd, intgroup = c("Parent_state", "Lake_type"))
```

```
## using ntop=500 top features by variance
```

```
# Plotting PC1 and PC2 manually for Parent state and Lake type

pca_data <- plotPCA(vsd, intgroup = c("Parent_state", "Lake_type"), returnData = TRUE)
```

```
## using ntop=500 top features by variance
```

```
percentVar <- round(100 * attr(pca_data, "percentVar"))

ggplot(pca_data) +
  geom_point(aes(x = PC1, y = PC2, color = Parent_state, shape = Lake_type), size = 3.2) +
  xlab(paste0("PC1: ", percentVar[1], "% variance")) +
  ylab(paste0("PC2: ", percentVar[2], "% variance")) +
  ggtitle("Transcriptomic PCA") +
  theme_classic()
```

```
# Getting normalized counts

count_matrix <- counts(dds, normalized = TRUE)

# Making the results tables.

meta_table <- akmeta %>%
  rownames_to_column(var = "samplename") %>%
  as_tibble()

norm_counts <- count_matrix %>%
  data.frame() %>%
  rownames_to_column(var = "gene") %>%
  as_tibble()
```

## Results

```
# Setting thresholds for what constitutes a DEG.

padj.cutoff <- 0.05
lfc.cutoff <- 0.58

# Lake type results

lake_name <- resultsNames(dds)[grep("Lake_type", resultsNames(dds))]
res_lake <- results(dds, name = lake_name)
resLFC_lake <- lfcShrink(dds, coef = lake_name, type = "apeglm")
```

```
## using 'apeglm' for LFC shrinkage. If used in published research, please cite:
##     Zhu, A., Ibrahim, J.G., Love, M.I. (2018) Heavy-tailed prior distributions for
##     sequence count data: removing the noise and preserving large differences.
##     Bioinformatics. https://doi.org/10.1093/bioinformatics/bty895
```

```
summary(resLFC_lake)
```

```
## 
## out of 22125 with nonzero total read count
## adjusted p-value < 0.1
## LFC > 0 (up)       : 34, 0.15%
## LFC < 0 (down)     : 46, 0.21%
## outliers [1]       : 195, 0.88%
## low counts [2]     : 0, 0%
## (mean count < 2)
## [1] see 'cooksCutoff' argument of ?results
## [2] see 'independentFiltering' argument of ?results
```

```
# Parent state results

parent_name <- resultsNames(dds)[grep("Parent_state", resultsNames(dds))]
res_parent <- results(dds, name = parent_name)
resLFC_parent <- lfcShrink(dds, coef = parent_name, type = "apeglm")
```

```
## using 'apeglm' for LFC shrinkage. If used in published research, please cite:
##     Zhu, A., Ibrahim, J.G., Love, M.I. (2018) Heavy-tailed prior distributions for
##     sequence count data: removing the noise and preserving large differences.
##     Bioinformatics. https://doi.org/10.1093/bioinformatics/bty895
```

```
summary(resLFC_parent)
```

```
## 
## out of 22125 with nonzero total read count
## adjusted p-value < 0.1
## LFC > 0 (up)       : 1, 0.0045%
## LFC < 0 (down)     : 0, 0%
## outliers [1]       : 195, 0.88%
## low counts [2]     : 0, 0%
## (mean count < 2)
## [1] see 'cooksCutoff' argument of ?results
## [2] see 'independentFiltering' argument of ?results
```

```
# Batch results


batch_names <- resultsNames(dds)[grep("^Batch_", resultsNames(dds))]
res_batch <- lapply(batch_names, function(x) results(dds, name = x))
names(res_batch) <- batch_names
resLFC_batch <- lapply(batch_names, function(x) lfcShrink(dds, coef = x, type = "apeglm"))
```

```
## using 'apeglm' for LFC shrinkage. If used in published research, please cite:
##     Zhu, A., Ibrahim, J.G., Love, M.I. (2018) Heavy-tailed prior distributions for
##     sequence count data: removing the noise and preserving large differences.
##     Bioinformatics. https://doi.org/10.1093/bioinformatics/bty895
## using 'apeglm' for LFC shrinkage. If used in published research, please cite:
##     Zhu, A., Ibrahim, J.G., Love, M.I. (2018) Heavy-tailed prior distributions for
##     sequence count data: removing the noise and preserving large differences.
##     Bioinformatics. https://doi.org/10.1093/bioinformatics/bty895
## using 'apeglm' for LFC shrinkage. If used in published research, please cite:
##     Zhu, A., Ibrahim, J.G., Love, M.I. (2018) Heavy-tailed prior distributions for
##     sequence count data: removing the noise and preserving large differences.
##     Bioinformatics. https://doi.org/10.1093/bioinformatics/bty895
```

```
names(resLFC_batch) <- batch_names
lapply(resLFC_batch, summary)
```

```
## 
## out of 22125 with nonzero total read count
## adjusted p-value < 0.1
## LFC > 0 (up)       : 0, 0%
## LFC < 0 (down)     : 2, 0.009%
## outliers [1]       : 195, 0.88%
## low counts [2]     : 0, 0%
## (mean count < 2)
## [1] see 'cooksCutoff' argument of ?results
## [2] see 'independentFiltering' argument of ?results
## 
## 
## out of 22125 with nonzero total read count
## adjusted p-value < 0.1
## LFC > 0 (up)       : 2, 0.009%
## LFC < 0 (down)     : 5, 0.023%
## outliers [1]       : 195, 0.88%
## low counts [2]     : 0, 0%
## (mean count < 2)
## [1] see 'cooksCutoff' argument of ?results
## [2] see 'independentFiltering' argument of ?results
## 
## 
## out of 22125 with nonzero total read count
## adjusted p-value < 0.1
## LFC > 0 (up)       : 0, 0%
## LFC < 0 (down)     : 2, 0.009%
## outliers [1]       : 195, 0.88%
## low counts [2]     : 0, 0%
## (mean count < 2)
## [1] see 'cooksCutoff' argument of ?results
## [2] see 'independentFiltering' argument of ?results
```

```
## $Batch_2_vs_1
## NULL
## 
## $Batch_3_vs_1
## NULL
## 
## $Batch_4_vs_1
## NULL
```

```
# MA Plots before and after shrinkage

plotMA(res_lake, ylim = c(-3,3))
```

```
plotMA(resLFC_lake, ylim = c(-3,3))
```

```
plotMA(res_parent, ylim = c(-3,3))
```

```
plotMA(resLFC_parent, ylim = c(-3,3))
```

```
# Making res_table to eventually get DEGs

res_table_lake <- res_lake %>%
  data.frame() %>%
  rownames_to_column(var = "gene") %>%
  as_tibble()

res_table_parent <- res_parent %>%
  data.frame() %>%
  rownames_to_column(var = "gene") %>%
  as_tibble()

DEG_lake <- res_table_lake %>%
  filter(padj < padj.cutoff & abs(log2FoldChange) >= lfc.cutoff)

DEG_parent <- res_table_parent %>%
  filter(padj < padj.cutoff & abs(log2FoldChange) >= lfc.cutoff)
```

## Collecting and visualizing top 30 DEG by lake type

```
# Getting the top 30 DEG from the lake-type res_table and subset those out of the normalized counts

top30_DEG_lake <- res_table_lake %>%
  arrange(padj) %>%
  pull(gene) %>%
  head(n = 30)

top30_DEG_norm_lake <- norm_counts %>%
  filter(gene %in% top30_DEG_lake)

# Converting from wide to long format to plot

top30_DEG_norm_long_lake <- top30_DEG_norm_lake %>%
  gather(colnames(top30_DEG_norm_lake)[2:ncol(top30_DEG_norm_lake)], key = "samplename", value = "normalized_counts")

top30_DEG_norm_long_lake <- inner_join(meta_table, top30_DEG_norm_long_lake, by = "samplename")

# Plotting top 30 DEGs

ggplot(top30_DEG_norm_long_lake) +
  geom_point(aes(x = gene, y = normalized_counts, color = Lake_type_label)) +
  scale_y_log10() +
  xlab("Genes") +
  ylab("log10 Normalized Counts") +
  ggtitle("Top 30 Significant DE Genes") +
  theme_bw() +
  theme(axis.text.x = element_text(angle = 45, hjust = 1)) +
  theme(plot.title = element_text(hjust = 0.5))
```

```
## Warning in scale_y_log10(): log-10 transformation introduced infinite values.
```

```
# Preparing for volcano plots - need to have a column in the results table stating whether or not the gene is considered differentially expressed

res_table_lake <- res_table_lake %>%
  mutate(threshold = padj < 0.05 & abs(log2FoldChange) >= 0.58)

res_table_parent <- res_table_parent %>%
  mutate(threshold = padj < 0.05 & abs(log2FoldChange) >= 0.58)
```

## Volcano Plots

```
# Making the volcano plot showing the DEGs

ggplot(res_table_lake) +
  geom_point(aes(x = log2FoldChange, y = -log10(padj), colour = threshold)) +
  scale_color_manual(values = c("blue", "green")) +
  geom_hline(yintercept = 1.30, linetype = "dashed", color = "blue") +
  geom_vline(xintercept = c(-0.58,0.58), linetype = "dashed", color = "black") +
  xlab("log2 fold change") +
  ylab("-log10 adjusted p-value") +
  scale_y_continuous(expand = c(0, 0)) +
  theme_bw() +
  theme(
    legend.position = "none",
    plot.title = element_text(size = rel(1.5), hjust = 0.3),
    axis.title = element_text(size = rel(1.25))
  )
```

```
## Warning: Removed 195 rows containing missing values or values outside the scale range
## (`geom_point()`).
```

```
ggplot(res_table_parent) +
  geom_point(aes(x = log2FoldChange, y = -log10(padj), colour = threshold)) +
  scale_color_manual(values = c("blue", "green")) +
  geom_hline(yintercept = 1.30, linetype = "dashed", color = "blue") +
  geom_vline(xintercept = c(-0.58,0.58), linetype = "dashed", color = "black") +
  xlab("log2 fold change") +
  ylab("-log10 adjusted p-value") +
  scale_y_continuous(expand = c(0, 0)) +
  theme_bw() +
  theme(
    legend.position = "none",
    plot.title = element_text(size = rel(1.5), hjust = 0.3),
    axis.title = element_text(size = rel(1.25))
  )
```

```
## Warning: Removed 195 rows containing missing values or values outside the scale range
## (`geom_point()`).
```

```
# Volcano plot showing the top 10 DEGs

res_table_lake <- res_table_lake %>% arrange(padj) %>% mutate(genelabels = "")
res_table_lake$genelabels[1:min(10, nrow(res_table_lake))] <- res_table_lake$gene[1:min(10, nrow(res_table_lake))]

ggplot(res_table_lake, aes(x = log2FoldChange, y = -log10(padj))) +
  geom_point(aes(colour = threshold)) +
  geom_text_repel(aes(label = genelabels)) +
  scale_color_manual(values = c("black", "red3")) +
  scale_y_continuous(expand = c(0, 0)) +
  xlab("log2 fold change") +
  ylab("-log10 adjusted p-value") +
  theme_classic() +
  theme(
    legend.position = "none",
    plot.title = element_text(size = rel(1.5), hjust = 0.5),
    axis.title = element_text(size = rel(1.25))
  )
```

```
## Warning: Removed 195 rows containing missing values or values outside the scale range
## (`geom_point()`).
```

```
## Warning: Removed 195 rows containing missing values or values outside the scale range
## (`geom_text_repel()`).
```

## Summaries of DEGs

```
#  summaries 

count_deg <- function(res_obj, padj_cutoff = 0.05, lfc_cutoff = 0.58) {
  data.frame(
    padj_only = sum(!is.na(res_obj$padj) & res_obj$padj < padj_cutoff),
    padj_and_lfc = sum(!is.na(res_obj$padj) &
                         res_obj$padj < padj_cutoff &
                         abs(res_obj$log2FoldChange) >= lfc_cutoff)
  )
}

deg_summary <- bind_rows(
  count_deg(res_batch[[1]]) %>% mutate(Effect = names(res_batch)[1]),
  count_deg(res_batch[[2]]) %>% mutate(Effect = names(res_batch)[2]),
  count_deg(res_batch[[3]]) %>% mutate(Effect = names(res_batch)[3]),
  count_deg(res_lake) %>% mutate(Effect = "Lake type"),
  count_deg(res_parent) %>% mutate(Effect = "Parent state")
  ) %>%
  dplyr::select(Effect, padj_only, padj_and_lfc)

print(deg_summary)
```

```
##         Effect padj_only padj_and_lfc
## 1 Batch_2_vs_1         2            2
## 2 Batch_3_vs_1         5            5
## 3 Batch_4_vs_1         2            2
## 4    Lake type        53           47
## 5 Parent state         1            1
```

## Heatmap of lake-type DEG

```
# Heatmap of top 30 lake-type DEGs

deg_ids <- as.data.frame(res_lake) %>%
  rownames_to_column("gene") %>%
  filter(!is.na(padj), padj < 0.05, abs(log2FoldChange) >= 0.58) %>%
  arrange(padj) %>%
  slice_head(n = 30) %>%
  pull(gene)

deg_ids <- deg_ids[deg_ids %in% rownames(vsd)]

mat <- assay(vsd)[deg_ids, , drop = FALSE]

meta_heat <- akmeta[match(colnames(mat), rownames(akmeta)), , drop = FALSE]

meta_heat$Lake_type_label <- ifelse(
  meta_heat$Lake_type %in% c("No Pike", "No pike"),
  "Native predator-only",
  "Native + invasive predator"
)

meta_heat$Lake_type_label <- factor(
  meta_heat$Lake_type_label,
  levels = c("Native predator-only", "Native + invasive predator")
)

annotation_col <- data.frame(
  Lake_type = meta_heat$Lake_type_label
)
rownames(annotation_col) <- colnames(mat)

ann_colors <- list(
  Lake_type = c(
    "Native predator-only" = "#00BFC4",
    "Native + invasive predator" = "#F8766D"
  )
)

pheatmap(
  mat,
  scale = "row",
  annotation_col = annotation_col,
  annotation_colors = ann_colors,
  show_rownames = FALSE,
  fontsize_col = 10,
  main = "Top lake-type DEGs"
)
```

## PCAs by Lake Type

```
# PCA by lake

plotPCA(vsd, intgroup = "Lake")
```

```
## using ntop=500 top features by variance
```

```
pca_lake <- plotPCA(vsd, intgroup = c("Lake"), returnData = TRUE)
```

```
## using ntop=500 top features by variance
```

```
percentVar_lake <- round(100 * attr(pca_lake, "percentVar"))

ggplot(pca_lake, aes(PC1, PC2, color = Lake, label = name)) +
  geom_point(size = 3) +
  theme_classic(base_size = 14) +
  labs(
    title = "PCA by lake",
    x = paste0("PC1: ", percentVar_lake[1], "% variance"),
    y = paste0("PC2: ", percentVar_lake[2], "% variance")
  )
```

```
# GO/GSEA for lake-type contrast
##requires server connection, im going to make a csv with gene annotations and use this to help with reproducibility
#mart <- biomaRt::useEnsembl(
 # biomart = "genes",
  #dataset = "gaculeatus_gene_ensembl"
#)

all_genes <- rownames(dds)

#go_map <- biomaRt::getBM(
 # attributes = c("ensembl_gene_id", "go_id"),
  #filters = "ensembl_gene_id",
  #values = all_genes,
  #mart = mart
#) %>%
 # filter(go_id != "") %>%
  #distinct()

#TERM2GENE <- go_map %>%
  #dplyr::select(go_id, ensembl_gene_id)

#TERM2NAME <- AnnotationDbi::select(
 # GO.db,
  #keys = unique(TERM2GENE$go_id),
  #keytype = "GOID",
  #columns = c("TERM")
#) %>%
 # distinct(GOID, TERM) %>%
  #rename(go_id = GOID, term_name = TERM)

annotations <- read.csv("annotations.csv")
TERM2GENE <- read.csv("TERM2GENE.csv")
TERM2NAME <- read.csv("TERM2NAME.csv")

# Ranked gene list for GSEA

gene_list_lake <- res_lake$stat
names(gene_list_lake) <- rownames(res_lake)
gene_list_lake <- gene_list_lake[!is.na(gene_list_lake)]
gene_list_lake <- sort(gene_list_lake, decreasing = TRUE)

gsea_lake <- clusterProfiler::GSEA(
  geneList = gene_list_lake,
  TERM2GENE = TERM2GENE,
  TERM2NAME = TERM2NAME,
  pAdjustMethod = "BH",
  minGSSize = 10,
  maxGSSize = 500,
  verbose = FALSE
)
```

```
## Warning in fgseaMultilevel(pathways = pathways, stats = stats, minSize =
## minSize, : For some of the pathways the P-values were likely overestimated. For
## such pathways log2err is set to NA.
```

```
## Warning in fgseaMultilevel(pathways = pathways, stats = stats, minSize =
## minSize, : For some pathways, in reality P-values are less than 1e-10. You can
## set the `eps` argument to zero for better estimation.
```

```
gsea_lake_df <- as.data.frame(gsea_lake)
head(gsea_lake_df)
```

```
##                    ID                        Description setSize
## GO:0005840 GO:0005840                           ribosome     165
## GO:0003735 GO:0003735 structural constituent of ribosome     156
## GO:1990904 GO:1990904          ribonucleoprotein complex     261
## GO:0006412 GO:0006412                        translation     260
## GO:0006457 GO:0006457                    protein folding     105
## GO:0006955 GO:0006955                    immune response     173
##            enrichmentScore       NES       pvalue     p.adjust       qvalue
## GO:0005840       0.5542044  2.534461 1.000000e-10 4.117500e-08 3.684211e-08
## GO:0003735       0.5349275  2.428842 1.000000e-10 4.117500e-08 3.684211e-08
## GO:1990904       0.4523350  2.196223 1.000000e-10 4.117500e-08 3.684211e-08
## GO:0006412       0.4510014  2.189596 1.000000e-10 4.117500e-08 3.684211e-08
## GO:0006457       0.5416464  2.323703 1.744106e-09 5.745087e-07 5.140524e-07
## GO:0006955      -0.4726590 -2.090520 3.665006e-09 1.006044e-06 9.001768e-07
##            rank                   leading_edge
## GO:0005840 6280 tags=69%, list=28%, signal=50%
## GO:0003735 6912 tags=74%, list=31%, signal=51%
## GO:1990904 6658 tags=60%, list=30%, signal=42%
## GO:0006412 6280 tags=58%, list=28%, signal=42%
## GO:0006457 5537 tags=56%, list=25%, signal=42%
## GO:0006955 4088 tags=40%, list=18%, signal=33%
##                                                                                                                                                                                                                                                                                                                                                                                                                                                                                                                                                                                                                                                                                                                                                                                                                                                                                                                                                                                                                                                                                                                                                                                                                                                                                                                                                                                                                                                                                                                                                                                                                                                                                                                                                                                                                                                                                                                                                                                                                                                                                                                                                                                                                                                                                                                                                                                                                                                                                                                                                                                                                                                                                                                                                                                                                                                                                                                                                                                                                                                                                core_enrichment
## GO:0005840                                                                                                                                                                                                                                                                                                                                                                                                                                                                                                                                                                                                                                                                                                                                                                                                                               ENSGACG00000032871/ENSGACG00000003841/ENSGACG00000026821/ENSGACG00000033301/ENSGACG00000009280/ENSGACG00000019808/ENSGACG00000015484/ENSGACG00000015957/ENSGACG00000031654/ENSGACG00000033227/ENSGACG00000005000/ENSGACG00000003236/ENSGACG00000001897/ENSGACG00000000216/ENSGACG00000003336/ENSGACG00000033285/ENSGACG00000017491/ENSGACG00000029643/ENSGACG00000001379/ENSGACG00000012791/ENSGACG00000010761/ENSGACG00000018439/ENSGACG00000031174/ENSGACG00000006818/ENSGACG00000024893/ENSGACG00000013789/ENSGACG00000015089/ENSGACG00000014559/ENSGACG00000031440/ENSGACG00000006703/ENSGACG00000014154/ENSGACG00000026759/ENSGACG00000000656/ENSGACG00000002767/ENSGACG00000002035/ENSGACG00000034539/ENSGACG00000013073/ENSGACG00000002118/ENSGACG00000015549/ENSGACG00000014786/ENSGACG00000025107/ENSGACG00000007990/ENSGACG00000032351/ENSGACG00000008208/ENSGACG00000009641/ENSGACG00000014635/ENSGACG00000004699/ENSGACG00000013312/ENSGACG00000009310/ENSGACG00000006018/ENSGACG00000009814/ENSGACG00000036768/ENSGACG00000002835/ENSGACG00000013384/ENSGACG00000013435/ENSGACG00000024042/ENSGACG00000032960/ENSGACG00000017861/ENSGACG00000011913/ENSGACG00000016489/ENSGACG00000024818/ENSGACG00000017164/ENSGACG00000009248/ENSGACG00000034117/ENSGACG00000015169/ENSGACG00000015568/ENSGACG00000018103/ENSGACG00000015752/ENSGACG00000003250/ENSGACG00000000474/ENSGACG00000004968/ENSGACG00000016123/ENSGACG00000001940/ENSGACG00000003574/ENSGACG00000036745/ENSGACG00000019181/ENSGACG00000020625/ENSGACG00000011859/ENSGACG00000029403/ENSGACG00000017971/ENSGACG00000003995/ENSGACG00000027822/ENSGACG00000024858/ENSGACG00000028743/ENSGACG00000027741/ENSGACG00000016153/ENSGACG00000008496/ENSGACG00000032540/ENSGACG00000000590/ENSGACG00000008736/ENSGACG00000007041/ENSGACG00000022942/ENSGACG00000037855/ENSGACG00000017440/ENSGACG00000027137/ENSGACG00000004230/ENSGACG00000026156/ENSGACG00000020826/ENSGACG00000002699/ENSGACG00000027413/ENSGACG00000003476/ENSGACG00000004206/ENSGACG00000033326/ENSGACG00000019593/ENSGACG00000018720/ENSGACG00000025550/ENSGACG00000026724/ENSGACG00000017077/ENSGACG00000015363/ENSGACG00000003110/ENSGACG00000025785/ENSGACG00000006548/ENSGACG00000035223/ENSGACG00000013792
## GO:0003735                                                                                                                                                                                                                                                                                                                                                                                                                                                                                                                                                                                                                                                                                                                                                                                                            ENSGACG00000032871/ENSGACG00000026821/ENSGACG00000033301/ENSGACG00000009280/ENSGACG00000019808/ENSGACG00000015484/ENSGACG00000031654/ENSGACG00000033227/ENSGACG00000003236/ENSGACG00000000216/ENSGACG00000003336/ENSGACG00000003757/ENSGACG00000005634/ENSGACG00000033285/ENSGACG00000017491/ENSGACG00000029643/ENSGACG00000001379/ENSGACG00000012791/ENSGACG00000010761/ENSGACG00000018439/ENSGACG00000031174/ENSGACG00000006818/ENSGACG00000024893/ENSGACG00000013789/ENSGACG00000014559/ENSGACG00000031440/ENSGACG00000006703/ENSGACG00000014154/ENSGACG00000026759/ENSGACG00000002767/ENSGACG00000002035/ENSGACG00000034539/ENSGACG00000017799/ENSGACG00000013073/ENSGACG00000002118/ENSGACG00000015549/ENSGACG00000014786/ENSGACG00000025107/ENSGACG00000028965/ENSGACG00000007990/ENSGACG00000032351/ENSGACG00000008208/ENSGACG00000009641/ENSGACG00000014635/ENSGACG00000004699/ENSGACG00000013312/ENSGACG00000009310/ENSGACG00000006018/ENSGACG00000009814/ENSGACG00000036768/ENSGACG00000002835/ENSGACG00000013384/ENSGACG00000013435/ENSGACG00000024042/ENSGACG00000032960/ENSGACG00000017861/ENSGACG00000011913/ENSGACG00000016489/ENSGACG00000024818/ENSGACG00000017164/ENSGACG00000009248/ENSGACG00000034117/ENSGACG00000015169/ENSGACG00000015568/ENSGACG00000015752/ENSGACG00000003250/ENSGACG00000000474/ENSGACG00000004968/ENSGACG00000016123/ENSGACG00000001940/ENSGACG00000003574/ENSGACG00000036745/ENSGACG00000019181/ENSGACG00000020625/ENSGACG00000011859/ENSGACG00000029403/ENSGACG00000017971/ENSGACG00000003995/ENSGACG00000027822/ENSGACG00000024858/ENSGACG00000028743/ENSGACG00000027741/ENSGACG00000016153/ENSGACG00000008496/ENSGACG00000032540/ENSGACG00000000590/ENSGACG00000008736/ENSGACG00000007041/ENSGACG00000022942/ENSGACG00000037855/ENSGACG00000017440/ENSGACG00000027137/ENSGACG00000004230/ENSGACG00000026156/ENSGACG00000020826/ENSGACG00000002699/ENSGACG00000027413/ENSGACG00000003476/ENSGACG00000004206/ENSGACG00000019593/ENSGACG00000018720/ENSGACG00000025550/ENSGACG00000017077/ENSGACG00000015363/ENSGACG00000003110/ENSGACG00000025785/ENSGACG00000035223/ENSGACG00000013792/ENSGACG00000004104/ENSGACG00000011905/ENSGACG00000029705/ENSGACG00000030045/ENSGACG00000016089/ENSGACG00000036711/ENSGACG00000019780
## GO:1990904 ENSGACG00000016848/ENSGACG00000032871/ENSGACG00000033421/ENSGACG00000019603/ENSGACG00000015478/ENSGACG00000003841/ENSGACG00000026821/ENSGACG00000011665/ENSGACG00000009838/ENSGACG00000033301/ENSGACG00000019808/ENSGACG00000015484/ENSGACG00000015957/ENSGACG00000031654/ENSGACG00000033227/ENSGACG00000017223/ENSGACG00000005000/ENSGACG00000003236/ENSGACG00000001897/ENSGACG00000000216/ENSGACG00000003336/ENSGACG00000033285/ENSGACG00000017491/ENSGACG00000029643/ENSGACG00000032918/ENSGACG00000001379/ENSGACG00000012791/ENSGACG00000036914/ENSGACG00000012793/ENSGACG00000031703/ENSGACG00000011087/ENSGACG00000029168/ENSGACG00000010761/ENSGACG00000018439/ENSGACG00000031174/ENSGACG00000006818/ENSGACG00000013789/ENSGACG00000015089/ENSGACG00000014559/ENSGACG00000031440/ENSGACG00000006703/ENSGACG00000014154/ENSGACG00000026759/ENSGACG00000018352/ENSGACG00000000656/ENSGACG00000002767/ENSGACG00000002455/ENSGACG00000002035/ENSGACG00000034539/ENSGACG00000013073/ENSGACG00000036738/ENSGACG00000002118/ENSGACG00000015549/ENSGACG00000012453/ENSGACG00000014786/ENSGACG00000005107/ENSGACG00000025107/ENSGACG00000007990/ENSGACG00000032351/ENSGACG00000008208/ENSGACG00000009641/ENSGACG00000014635/ENSGACG00000004699/ENSGACG00000013312/ENSGACG00000009310/ENSGACG00000010920/ENSGACG00000020631/ENSGACG00000006018/ENSGACG00000009814/ENSGACG00000036768/ENSGACG00000002835/ENSGACG00000013384/ENSGACG00000013435/ENSGACG00000024042/ENSGACG00000032960/ENSGACG00000017861/ENSGACG00000008140/ENSGACG00000011913/ENSGACG00000016489/ENSGACG00000024818/ENSGACG00000017164/ENSGACG00000009248/ENSGACG00000035136/ENSGACG00000034117/ENSGACG00000005201/ENSGACG00000015169/ENSGACG00000015568/ENSGACG00000018103/ENSGACG00000008504/ENSGACG00000015752/ENSGACG00000003250/ENSGACG00000000474/ENSGACG00000004968/ENSGACG00000016123/ENSGACG00000001940/ENSGACG00000003574/ENSGACG00000005528/ENSGACG00000036745/ENSGACG00000019181/ENSGACG00000020625/ENSGACG00000011859/ENSGACG00000029403/ENSGACG00000017971/ENSGACG00000019682/ENSGACG00000003995/ENSGACG00000027822/ENSGACG00000024858/ENSGACG00000028743/ENSGACG00000027741/ENSGACG00000016153/ENSGACG00000008496/ENSGACG00000004110/ENSGACG00000032540/ENSGACG00000000590/ENSGACG00000008736/ENSGACG00000007041/ENSGACG00000004899/ENSGACG00000022942/ENSGACG00000017440/ENSGACG00000027137/ENSGACG00000004230/ENSGACG00000026156/ENSGACG00000012495/ENSGACG00000020826/ENSGACG00000002699/ENSGACG00000027413/ENSGACG00000003476/ENSGACG00000004206/ENSGACG00000033326/ENSGACG00000018720/ENSGACG00000017772/ENSGACG00000003155/ENSGACG00000025550/ENSGACG00000003262/ENSGACG00000026724/ENSGACG00000017077/ENSGACG00000007581/ENSGACG00000015363/ENSGACG00000006697/ENSGACG00000003110/ENSGACG00000003202/ENSGACG00000032529/ENSGACG00000025785/ENSGACG00000006548/ENSGACG00000016749/ENSGACG00000035223/ENSGACG00000013792/ENSGACG00000003488/ENSGACG00000017237/ENSGACG00000036631/ENSGACG00000003827/ENSGACG00000034260/ENSGACG00000004104/ENSGACG00000011905/ENSGACG00000017533/ENSGACG00000029705
## GO:0006412                                                                             ENSGACG00000020720/ENSGACG00000004044/ENSGACG00000005536/ENSGACG00000015551/ENSGACG00000011734/ENSGACG00000032871/ENSGACG00000007055/ENSGACG00000009413/ENSGACG00000017928/ENSGACG00000026821/ENSGACG00000004324/ENSGACG00000004505/ENSGACG00000004490/ENSGACG00000006278/ENSGACG00000013152/ENSGACG00000006474/ENSGACG00000003191/ENSGACG00000033301/ENSGACG00000015749/ENSGACG00000014921/ENSGACG00000006117/ENSGACG00000009280/ENSGACG00000004921/ENSGACG00000002182/ENSGACG00000019808/ENSGACG00000015484/ENSGACG00000020072/ENSGACG00000031654/ENSGACG00000003410/ENSGACG00000033227/ENSGACG00000009601/ENSGACG00000003236/ENSGACG00000018611/ENSGACG00000001897/ENSGACG00000000216/ENSGACG00000003336/ENSGACG00000020813/ENSGACG00000009864/ENSGACG00000033285/ENSGACG00000017491/ENSGACG00000000793/ENSGACG00000029643/ENSGACG00000001379/ENSGACG00000012791/ENSGACG00000012793/ENSGACG00000010761/ENSGACG00000014823/ENSGACG00000007146/ENSGACG00000032506/ENSGACG00000018439/ENSGACG00000031174/ENSGACG00000000697/ENSGACG00000024893/ENSGACG00000013789/ENSGACG00000014559/ENSGACG00000031440/ENSGACG00000004504/ENSGACG00000006703/ENSGACG00000014154/ENSGACG00000026759/ENSGACG00000002035/ENSGACG00000034539/ENSGACG00000017799/ENSGACG00000013073/ENSGACG00000000648/ENSGACG00000008569/ENSGACG00000015598/ENSGACG00000002118/ENSGACG00000015549/ENSGACG00000010536/ENSGACG00000002667/ENSGACG00000014631/ENSGACG00000025107/ENSGACG00000007990/ENSGACG00000017191/ENSGACG00000032351/ENSGACG00000008208/ENSGACG00000014635/ENSGACG00000004699/ENSGACG00000013312/ENSGACG00000009310/ENSGACG00000000009/ENSGACG00000006018/ENSGACG00000009814/ENSGACG00000036768/ENSGACG00000012678/ENSGACG00000002835/ENSGACG00000019994/ENSGACG00000013384/ENSGACG00000012961/ENSGACG00000013435/ENSGACG00000024042/ENSGACG00000032960/ENSGACG00000017861/ENSGACG00000011913/ENSGACG00000016489/ENSGACG00000012829/ENSGACG00000019587/ENSGACG00000024818/ENSGACG00000017164/ENSGACG00000009248/ENSGACG00000017501/ENSGACG00000015169/ENSGACG00000015568/ENSGACG00000015752/ENSGACG00000000474/ENSGACG00000015402/ENSGACG00000004968/ENSGACG00000016123/ENSGACG00000001940/ENSGACG00000003574/ENSGACG00000036745/ENSGACG00000019181/ENSGACG00000008010/ENSGACG00000020625/ENSGACG00000017702/ENSGACG00000029403/ENSGACG00000017971/ENSGACG00000018684/ENSGACG00000027822/ENSGACG00000028743/ENSGACG00000027741/ENSGACG00000016738/ENSGACG00000016153/ENSGACG00000032540/ENSGACG00000000590/ENSGACG00000006293/ENSGACG00000008736/ENSGACG00000007041/ENSGACG00000022942/ENSGACG00000008973/ENSGACG00000037855/ENSGACG00000017440/ENSGACG00000027137/ENSGACG00000004230/ENSGACG00000026156/ENSGACG00000007652/ENSGACG00000020826/ENSGACG00000002699/ENSGACG00000003476/ENSGACG00000004206/ENSGACG00000000261/ENSGACG00000019593/ENSGACG00000018720/ENSGACG00000025550/ENSGACG00000017077/ENSGACG00000015363/ENSGACG00000003110/ENSGACG00000008516/ENSGACG00000025785/ENSGACG00000035223/ENSGACG00000013792
## GO:0006457                                                                                                                                                                                                                                                                                                                                                                                                                                                                                                                                                                                                                                                                                                                                                                                                                                                                                                                                                                                                                                                                                                                                                                                                                                                                                                                                                                                                                                                                                                                                                                                                                                                                                                                                                                                                                                                                                                                    ENSGACG00000020732/ENSGACG00000012888/ENSGACG00000033276/ENSGACG00000010853/ENSGACG00000016892/ENSGACG00000011040/ENSGACG00000013773/ENSGACG00000018185/ENSGACG00000004625/ENSGACG00000016570/ENSGACG00000016157/ENSGACG00000013530/ENSGACG00000008860/ENSGACG00000014888/ENSGACG00000010517/ENSGACG00000009733/ENSGACG00000003052/ENSGACG00000017879/ENSGACG00000007033/ENSGACG00000016609/ENSGACG00000024763/ENSGACG00000030760/ENSGACG00000008508/ENSGACG00000016898/ENSGACG00000010635/ENSGACG00000019542/ENSGACG00000033822/ENSGACG00000016633/ENSGACG00000018171/ENSGACG00000026117/ENSGACG00000012561/ENSGACG00000019042/ENSGACG00000000958/ENSGACG00000008210/ENSGACG00000015962/ENSGACG00000027482/ENSGACG00000007662/ENSGACG00000011350/ENSGACG00000009047/ENSGACG00000019293/ENSGACG00000000681/ENSGACG00000004481/ENSGACG00000007238/ENSGACG00000019815/ENSGACG00000001108/ENSGACG00000020617/ENSGACG00000000860/ENSGACG00000019868/ENSGACG00000010067/ENSGACG00000010423/ENSGACG00000002512/ENSGACG00000017455/ENSGACG00000001906/ENSGACG00000010080/ENSGACG00000002309/ENSGACG00000006344/ENSGACG00000018188/ENSGACG00000020916/ENSGACG00000019847
## GO:0006955                                                                                                                                                                                                                                                                                                                                                                                                                                                                                                                                                                                                                                                                                                                                                                                                                                                                                                                                                                                                                                                                                                                                                                                                                                                                                                                                                                                                                                                                                                                                                                                                                                                                                                                      ENSGACG00000010581/ENSGACG00000000344/ENSGACG00000035814/ENSGACG00000029316/ENSGACG00000012556/ENSGACG00000025748/ENSGACG00000009673/ENSGACG00000017967/ENSGACG00000029163/ENSGACG00000001973/ENSGACG00000027816/ENSGACG00000003680/ENSGACG00000026753/ENSGACG00000035512/ENSGACG00000001322/ENSGACG00000036105/ENSGACG00000010801/ENSGACG00000006251/ENSGACG00000027463/ENSGACG00000033513/ENSGACG00000017958/ENSGACG00000033349/ENSGACG00000016874/ENSGACG00000000348/ENSGACG00000019055/ENSGACG00000007282/ENSGACG00000001913/ENSGACG00000025331/ENSGACG00000031262/ENSGACG00000033047/ENSGACG00000012278/ENSGACG00000023544/ENSGACG00000033906/ENSGACG00000009755/ENSGACG00000025164/ENSGACG00000023330/ENSGACG00000018669/ENSGACG00000018016/ENSGACG00000029800/ENSGACG00000024454/ENSGACG00000033802/ENSGACG00000032350/ENSGACG00000025686/ENSGACG00000001790/ENSGACG00000029691/ENSGACG00000028695/ENSGACG00000002280/ENSGACG00000028484/ENSGACG00000029915/ENSGACG00000017177/ENSGACG00000000116/ENSGACG00000027754/ENSGACG00000031200/ENSGACG00000023876/ENSGACG00000033677/ENSGACG00000032149/ENSGACG00000000330/ENSGACG00000035016/ENSGACG00000034360/ENSGACG00000020069/ENSGACG00000033617/ENSGACG00000030777/ENSGACG00000031915/ENSGACG00000033893/ENSGACG00000028941/ENSGACG00000030746/ENSGACG00000010301/ENSGACG00000018112/ENSGACG00000029780
```

## Dotplot and Ridgeplots

```
# Dotplot and ridgeplot

dotplot(gsea_lake, showCategory = 15)
```

```
ridgeplot(gsea_lake, showCategory = 10) +
  ggtitle("GSEA ridgeplot: lake-type differences") +
  labs(
    x = "Higher in native predator-only  <---- ranked gene statistic ---->  Higher in native + invasive predator lakes",
    y = NULL
  ) +
  theme_classic(base_size = 13)
```

```
## Picking joint bandwidth of 0.206
```

```
# GO enrichment using only strong lake-type DEGs

lake_deg_ids <- DEG_lake$gene

ego_lake <- clusterProfiler::enricher(
  gene = lake_deg_ids,
  universe = all_genes,
  TERM2GENE = TERM2GENE,
  TERM2NAME = TERM2NAME,
  pAdjustMethod = "BH"
)

if (!is.null(ego_lake) && nrow(as.data.frame(ego_lake)) > 0) {
  dotplot(ego_lake, showCategory = 15)
  head(as.data.frame(ego_lake))
} else {
  message("No significant DEG-based GO enrichment found for lake-type DEGs.")
}
```

```
##                    ID                      Description GeneRatio   BgRatio
## GO:0009897 GO:0009897 external side of plasma membrane      5/37 181/18816
## GO:0035869 GO:0035869          ciliary transition zone      2/37  22/18816
## GO:0060170 GO:0060170                 ciliary membrane      2/37  26/18816
## GO:1905515 GO:1905515       non-motile cilium assembly      2/37  27/18816
## GO:0019957 GO:0019957            C-C chemokine binding      2/37  36/18816
## GO:0016493 GO:0016493  C-C chemokine receptor activity      2/37  37/18816
##            RichFactor FoldEnrichment   zScore       pvalue   p.adjust
## GO:0009897 0.02762431       14.04808 7.829571 2.646699e-05 0.00272610
## GO:0035869 0.09090909       46.23096 9.422242 8.478471e-04 0.03296871
## GO:0060170 0.07692308       39.11850 8.633281 1.186967e-03 0.03296871
## GO:1905515 0.07407407       37.66967 8.463574 1.280338e-03 0.03296871
## GO:0019957 0.05555556       28.25225 7.264782 2.272605e-03 0.03530694
## GO:0016493 0.05405405       27.48868 7.158824 2.399501e-03 0.03530694
##                qvalue
## GO:0009897 0.00183876
## GO:0035869 0.02223746
## GO:0060170 0.02223746
## GO:1905515 0.02223746
## GO:0019957 0.02381459
## GO:0016493 0.02381459
##                                                                                                    geneID
## GO:0009897 ENSGACG00000006132/ENSGACG00000018112/ENSGACG00000029780/ENSGACG00000034997/ENSGACG00000000523
## GO:0035869                                                          ENSGACG00000011177/ENSGACG00000010954
## GO:0060170                                                          ENSGACG00000011177/ENSGACG00000010954
## GO:1905515                                                          ENSGACG00000011177/ENSGACG00000010954
## GO:0019957                                                          ENSGACG00000018112/ENSGACG00000000523
## GO:0016493                                                          ENSGACG00000018112/ENSGACG00000000523
##            Count
## GO:0009897     5
## GO:0035869     2
## GO:0060170     2
## GO:1905515     2
## GO:0019957     2
## GO:0016493     2
```

```
#  GSEA for parent state

gene_list_parent <- res_parent$stat
names(gene_list_parent) <- rownames(res_parent)
gene_list_parent <- gene_list_parent[!is.na(gene_list_parent)]
gene_list_parent <- sort(gene_list_parent, decreasing = TRUE)

gsea_parent <- clusterProfiler::GSEA(
  geneList = gene_list_parent,
  TERM2GENE = TERM2GENE,
  TERM2NAME = TERM2NAME,
  pAdjustMethod = "BH",
  minGSSize = 10,
  maxGSSize = 500,
  verbose = FALSE
)

gsea_parent_df <- as.data.frame(gsea_parent)
head(gsea_parent_df)
```

```
##                    ID                         Description setSize
## GO:0007601 GO:0007601                   visual perception      98
## GO:0007229 GO:0007229 integrin-mediated signaling pathway      41
## GO:0140359 GO:0140359       ABC-type transporter activity      49
## GO:0005730 GO:0005730                           nucleolus     255
## GO:0000398 GO:0000398      mRNA splicing, via spliceosome     102
## GO:0016323 GO:0016323         basolateral plasma membrane     117
##            enrichmentScore       NES       pvalue     p.adjust       qvalue
## GO:0007601       0.5627558  2.293789 8.405442e-10 1.384376e-06 1.200651e-06
## GO:0007229      -0.6728948 -2.358626 6.186403e-08 4.169034e-05 3.615748e-05
## GO:0140359      -0.6339012 -2.298011 7.593869e-08 4.169034e-05 3.615748e-05
## GO:0005730       0.3817204  1.799505 5.080930e-07 2.092073e-04 1.814427e-04
## GO:0000398       0.4898909  2.009565 1.964433e-06 3.759580e-04 3.260633e-04
## GO:0016323      -0.4603768 -1.983820 2.177837e-06 3.759580e-04 3.260633e-04
##            rank                   leading_edge
## GO:0007601 3066 tags=40%, list=14%, signal=34%
## GO:0007229 4104 tags=59%, list=19%, signal=48%
## GO:0140359 2129 tags=41%, list=10%, signal=37%
## GO:0005730 5859 tags=43%, list=26%, signal=32%
## GO:0000398 5362 tags=44%, list=24%, signal=34%
## GO:0016323 3927 tags=41%, list=18%, signal=34%
##                                                                                                                                                                                                                                                                                                                                                                                                                                                                                                                                                                                                                                                                                                                                                                                                                                                                                                                                                                                                                                                                                                                                                                                                                                                                                                                                                                                                                                                                                                                                                                                                                                                                                                                                                                                                                                                                                                                                                                                                                                                                                                                                                   core_enrichment
## GO:0007601                                                                                                                                                                                                                                                                                                                                                                                                                                                                                                                                                                                                                                                                                                                                                                                                                                                                                                                                                                                                                                                                                                                                                                                                                                                                                                                                                                                   ENSGACG00000003369/ENSGACG00000020277/ENSGACG00000010229/ENSGACG00000036771/ENSGACG00000010287/ENSGACG00000023234/ENSGACG00000030902/ENSGACG00000025749/ENSGACG00000020446/ENSGACG00000001398/ENSGACG00000017311/ENSGACG00000012301/ENSGACG00000025364/ENSGACG00000005061/ENSGACG00000014695/ENSGACG00000012325/ENSGACG00000017581/ENSGACG00000014432/ENSGACG00000016811/ENSGACG00000006407/ENSGACG00000017170/ENSGACG00000016885/ENSGACG00000014464/ENSGACG00000003256/ENSGACG00000013964/ENSGACG00000037100/ENSGACG00000000716/ENSGACG00000003215/ENSGACG00000015748/ENSGACG00000025645/ENSGACG00000008331/ENSGACG00000002128/ENSGACG00000014459/ENSGACG00000027434/ENSGACG00000036079/ENSGACG00000023363/ENSGACG00000001181/ENSGACG00000004859/ENSGACG00000005155
## GO:0007229                                                                                                                                                                                                                                                                                                                                                                                                                                                                                                                                                                                                                                                                                                                                                                                                                                                                                                                                                                                                                                                                                                                                                                                                                                                                                                                                                                                                                                                                                                                                                                                                                                                                                ENSGACG00000000260/ENSGACG00000010383/ENSGACG00000020423/ENSGACG00000006983/ENSGACG00000016225/ENSGACG00000037610/ENSGACG00000012478/ENSGACG00000001584/ENSGACG00000001519/ENSGACG00000008632/ENSGACG00000031992/ENSGACG00000017305/ENSGACG00000011820/ENSGACG00000006460/ENSGACG00000006439/ENSGACG00000005354/ENSGACG00000005054/ENSGACG00000018163/ENSGACG00000008445/ENSGACG00000016924/ENSGACG00000002051/ENSGACG00000008948/ENSGACG00000015418/ENSGACG00000004543
## GO:0140359                                                                                                                                                                                                                                                                                                                                                                                                                                                                                                                                                                                                                                                                                                                                                                                                                                                                                                                                                                                                                                                                                                                                                                                                                                                                                                                                                                                                                                                                                                                                                                                                                                                                                                                                                            ENSGACG00000008403/ENSGACG00000000434/ENSGACG00000018231/ENSGACG00000005285/ENSGACG00000000078/ENSGACG00000016636/ENSGACG00000009039/ENSGACG00000019493/ENSGACG00000005901/ENSGACG00000018590/ENSGACG00000017497/ENSGACG00000019172/ENSGACG00000013989/ENSGACG00000025067/ENSGACG00000016304/ENSGACG00000019810/ENSGACG00000027761/ENSGACG00000036404/ENSGACG00000000142/ENSGACG00000007419
## GO:0005730 ENSGACG00000012761/ENSGACG00000001470/ENSGACG00000007417/ENSGACG00000018481/ENSGACG00000025620/ENSGACG00000020229/ENSGACG00000001596/ENSGACG00000000557/ENSGACG00000010626/ENSGACG00000009462/ENSGACG00000029050/ENSGACG00000007898/ENSGACG00000018198/ENSGACG00000034260/ENSGACG00000017474/ENSGACG00000002618/ENSGACG00000003882/ENSGACG00000011343/ENSGACG00000009941/ENSGACG00000017887/ENSGACG00000015892/ENSGACG00000020319/ENSGACG00000002244/ENSGACG00000027429/ENSGACG00000007425/ENSGACG00000012710/ENSGACG00000023161/ENSGACG00000020624/ENSGACG00000014339/ENSGACG00000019931/ENSGACG00000004429/ENSGACG00000004993/ENSGACG00000030011/ENSGACG00000007092/ENSGACG00000014802/ENSGACG00000002633/ENSGACG00000018812/ENSGACG00000017026/ENSGACG00000006712/ENSGACG00000029342/ENSGACG00000033507/ENSGACG00000001040/ENSGACG00000016301/ENSGACG00000002615/ENSGACG00000033961/ENSGACG00000037169/ENSGACG00000010317/ENSGACG00000019481/ENSGACG00000019424/ENSGACG00000035166/ENSGACG00000014270/ENSGACG00000015998/ENSGACG00000037815/ENSGACG00000009164/ENSGACG00000008931/ENSGACG00000017435/ENSGACG00000003920/ENSGACG00000014510/ENSGACG00000013680/ENSGACG00000010843/ENSGACG00000001798/ENSGACG00000016888/ENSGACG00000017989/ENSGACG00000014724/ENSGACG00000010320/ENSGACG00000004125/ENSGACG00000000373/ENSGACG00000033529/ENSGACG00000013788/ENSGACG00000009951/ENSGACG00000014740/ENSGACG00000009686/ENSGACG00000007367/ENSGACG00000007043/ENSGACG00000009302/ENSGACG00000011280/ENSGACG00000006531/ENSGACG00000019603/ENSGACG00000016491/ENSGACG00000030299/ENSGACG00000015989/ENSGACG00000024224/ENSGACG00000017630/ENSGACG00000000856/ENSGACG00000013778/ENSGACG00000020889/ENSGACG00000015478/ENSGACG00000002926/ENSGACG00000016369/ENSGACG00000008065/ENSGACG00000017223/ENSGACG00000012843/ENSGACG00000009841/ENSGACG00000015540/ENSGACG00000008953/ENSGACG00000006679/ENSGACG00000003927/ENSGACG00000005774/ENSGACG00000017878/ENSGACG00000000969/ENSGACG00000000906/ENSGACG00000010391/ENSGACG00000005616/ENSGACG00000017236/ENSGACG00000034060/ENSGACG00000016805/ENSGACG00000020819/ENSGACG00000036438/ENSGACG00000000543
## GO:0000398                                                                                                                                                                                                                                                                                                                                                                                                                                                                                                                                                                                                                                                                                                                                                                                                                                                                                                                                                                                                                                                                                                                                                                                                                                                                 ENSGACG00000020601/ENSGACG00000010469/ENSGACG00000013725/ENSGACG00000017872/ENSGACG00000002071/ENSGACG00000001855/ENSGACG00000002048/ENSGACG00000008839/ENSGACG00000009787/ENSGACG00000000858/ENSGACG00000011539/ENSGACG00000035489/ENSGACG00000010993/ENSGACG00000018334/ENSGACG00000002576/ENSGACG00000005051/ENSGACG00000009978/ENSGACG00000011792/ENSGACG00000038071/ENSGACG00000003736/ENSGACG00000019174/ENSGACG00000020374/ENSGACG00000009247/ENSGACG00000025057/ENSGACG00000010600/ENSGACG00000020459/ENSGACG00000011261/ENSGACG00000007759/ENSGACG00000000826/ENSGACG00000015154/ENSGACG00000025403/ENSGACG00000020842/ENSGACG00000006697/ENSGACG00000006238/ENSGACG00000020889/ENSGACG00000025442/ENSGACG00000011852/ENSGACG00000017223/ENSGACG00000008165/ENSGACG00000005528/ENSGACG00000007305/ENSGACG00000008140/ENSGACG00000014605/ENSGACG00000009122/ENSGACG00000015069
## GO:0016323                                                                                                                                                                                                                                                                                                                                                                                                                                                                                                                                                                                                                                                                                                                                                                                                                                                                                                                                                                                                                                                                                                                                                                                                        ENSGACG00000009380/ENSGACG00000016710/ENSGACG00000013867/ENSGACG00000004664/ENSGACG00000013601/ENSGACG00000019161/ENSGACG00000017666/ENSGACG00000004150/ENSGACG00000001034/ENSGACG00000020209/ENSGACG00000013635/ENSGACG00000019116/ENSGACG00000014471/ENSGACG00000004850/ENSGACG00000002224/ENSGACG00000006498/ENSGACG00000019745/ENSGACG00000010483/ENSGACG00000005363/ENSGACG00000015467/ENSGACG00000034268/ENSGACG00000016343/ENSGACG00000008604/ENSGACG00000005400/ENSGACG00000000434/ENSGACG00000005457/ENSGACG00000003483/ENSGACG00000028370/ENSGACG00000017515/ENSGACG00000026437/ENSGACG00000001525/ENSGACG00000005924/ENSGACG00000020606/ENSGACG00000017497/ENSGACG00000010315/ENSGACG00000013989/ENSGACG00000030927/ENSGACG00000018635/ENSGACG00000010749/ENSGACG00000019810/ENSGACG00000001994/ENSGACG00000002199/ENSGACG00000001880/ENSGACG00000007018/ENSGACG00000009715/ENSGACG00000002728/ENSGACG00000003718/ENSGACG00000004552
```

```
if (nrow(gsea_parent_df) > 0) {
  dotplot(gsea_parent, showCategory = 15)
}
```

```
# Quick summary lines

cat("\nDEG summary:\n")
```

```
## 
## DEG summary:
```

```
print(deg_summary)
```

```
##         Effect padj_only padj_and_lfc
## 1 Batch_2_vs_1         2            2
## 2 Batch_3_vs_1         5            5
## 3 Batch_4_vs_1         2            2
## 4    Lake type        53           47
## 5 Parent state         1            1
```

```
cat("\nNumber of lake-type DEGs:\n")
```

```
## 
## Number of lake-type DEGs:
```

```
print(nrow(DEG_lake))
```

```
## [1] 47
```

```
cat("\nNumber of parent-state DEGs:\n")
```

```
## 
## Number of parent-state DEGs:
```

```
print(nrow(DEG_parent))
```

```
## [1] 1
```

# Plotting the sites

```
#Plotting the sites!

shelf(ggplot2, dplyr, sf, ggrepel, ggspatial, prettymapr, rnaturalearth, rnaturalearthdata)
```

```
## 
##   The 'cran_repo' argument in shelf() was not set, so it will use
##   cran_repo = 'https://cran.r-project.org' by default.
## 
##   To avoid this message, set the 'cran_repo' argument to a CRAN
##   mirror URL (see https://cran.r-project.org/mirrors.html) or set
##   'quiet = TRUE'.
```

```
# lake coordinates
lakes_map <- data.frame(
  Lake = c("South Rolly", "Crystal", "Finger", "Watson", "Hope", "Spirit"),
  Lake_type = c(
    "Native + invasive predator",
    "Native + invasive predator",
    "Native + invasive predator",
    "Native predators only",
    "Native predators only",
    "Native predators only"
  ),
  Longitude = c(
    -150.135689, -150.102865, -149.279200,
    -150.467000, -151.188278, -150.997664
  ),
  Latitude = c(
    61.667545, 61.710514, 61.605600,
    60.539000, 60.421528, 60.596104
  )
)

pal_lake_map <- c(
  "Native predators only" = "#0072B2",
  "Native + invasive predator" = "#CC79A7"
)

# convert lakes to sf object
lakes_sf <- st_as_sf(
  lakes_map,
  coords = c("Longitude", "Latitude"),
  crs = 4326
)

# transform to Web Mercator (required for map tiles)
lakes_sf_3857 <- st_transform(lakes_sf, 3857)

# plot
p_lake_map <- ggplot() +

  annotation_map_tile(
    type = "osm",
    zoom = 7
  ) +

  geom_sf(
    data = lakes_sf_3857,
    aes(color = Lake_type),
    size = 4
  ) +

  geom_sf_text(
    data = lakes_sf_3857,
    aes(label = Lake),
    size = 3.8,
    fontface = "bold",
    nudge_y = 15000
  ) +

  scale_color_manual(values = pal_lake_map) +

  coord_sf(
    crs = 3857,
    xlim = c(-17000000, -16500000),
    ylim = c(8400000, 8900000),
    expand = FALSE
  ) +

  # Scale bar
  annotation_scale(
    location = "bl",
    width_hint = 0.25,
    unit_category = "metric",
    text_cex = 0.8,
    line_width = 0.8
  ) +

  # North arrow
  annotation_north_arrow(
    location = "tr",
    which_north = "true",
    style = north_arrow_fancy_orienteering
  ) +

  labs(
    title = "Study lakes in southcentral Alaska",
    x = "Longitude",
    y = "Latitude",
    color = "Lake type"
  ) +

  theme_bw(base_size = 14) +

  theme(
    plot.title = element_text(
      face = "bold",
      hjust = 0.5,
      size = 16
    ),
    axis.title = element_text(face = "bold"),
    legend.position = "bottom",
    legend.title = element_text(face = "bold"),
    panel.grid.major = element_line(
      color = "grey70",
      linewidth = 0.3
    )
  )

print(p_lake_map)
```

```
## Zoom: 7
```

```
ggsave(
  filename = "study_lakes_map.png",
  plot = p_lake_map,
  width = 8,
  height = 6,
  units = "in",
  dpi = 600,
  bg = "white"
)
```

```
## Zoom: 7
```

```
# Session Information


``` r
sessionInfo()
```

```
## R version 4.5.2 (2025-10-31)
## Platform: x86_64-apple-darwin20
## Running under: macOS Ventura 13.7.8
## 
## Matrix products: default
## BLAS:   /Library/Frameworks/R.framework/Versions/4.5-x86_64/Resources/lib/libRblas.0.dylib 
## LAPACK: /Library/Frameworks/R.framework/Versions/4.5-x86_64/Resources/lib/libRlapack.dylib;  LAPACK version 3.12.1
## 
## locale:
## [1] en_US.UTF-8/en_US.UTF-8/en_US.UTF-8/C/en_US.UTF-8/en_US.UTF-8
## 
## time zone: America/Chicago
## tzcode source: internal
## 
## attached base packages:
## [1] stats4    stats     graphics  grDevices utils     datasets  methods  
## [8] base     
## 
## other attached packages:
##  [1] rnaturalearthdata_1.0.0     rnaturalearth_1.2.0        
##  [3] prettymapr_0.2.5            ggspatial_1.1.10           
##  [5] sf_1.1-1                    pheatmap_1.0.13            
##  [7] biomaRt_2.66.0              GO.db_3.22.0               
##  [9] enrichplot_1.30.4           clusterProfiler_4.18.4     
## [11] AnnotationDbi_1.72.0        ggrepel_0.9.7              
## [13] RColorBrewer_1.1-3          vsn_3.78.1                 
## [15] apeglm_1.32.0               DESeq2_1.50.2              
## [17] SummarizedExperiment_1.40.0 Biobase_2.70.0             
## [19] MatrixGenerics_1.22.0       matrixStats_1.5.0          
## [21] GenomicRanges_1.62.1        Seqinfo_1.0.0              
## [23] IRanges_2.44.0              S4Vectors_0.48.0           
## [25] BiocGenerics_0.56.0         generics_0.1.4             
## [27] genefilter_1.92.0           ggeffects_2.3.2            
## [29] sjPlot_2.9.0                effectsize_1.0.1           
## [31] MuMIn_1.48.11               performance_0.16.0         
## [33] car_3.1-5                   carData_3.0-6              
## [35] emmeans_2.0.2               lmerTest_3.2-1             
## [37] lme4_2.0-1                  Matrix_1.7-4               
## [39] broom.mixed_0.2.9.7         broom_1.0.12               
## [41] kableExtra_1.4.0            knitr_1.51                 
## [43] lubridate_1.9.5             forcats_1.0.1              
## [45] stringr_1.6.0               dplyr_1.2.0                
## [47] purrr_1.2.1                 readr_2.2.0                
## [49] tidyr_1.3.2                 tibble_3.3.1               
## [51] ggplot2_4.0.2               tidyverse_2.0.0            
## [53] librarian_1.8.1            
## 
## loaded via a namespace (and not attached):
##   [1] fs_1.6.6                httr_1.4.8              insight_1.4.6          
##   [4] numDeriv_2016.8-1.1     tools_4.5.2             backports_1.5.0        
##   [7] utf8_1.2.6              R6_2.6.1                lazyeval_0.2.2         
##  [10] withr_3.0.2             prettyunits_1.2.0       preprocessCore_1.72.0  
##  [13] cli_3.6.5               textshaping_1.0.4       scatterpie_0.2.6       
##  [16] sandwich_3.1-1          labeling_0.4.3          sass_0.4.10            
##  [19] mvtnorm_1.3-3           S7_0.2.1                proxy_0.4-29           
##  [22] ggridges_0.5.7          systemfonts_1.3.2       yulab.utils_0.2.4      
##  [25] gson_0.1.0              DOSE_4.4.0              svglite_2.2.2          
##  [28] R.utils_2.13.0          parallelly_1.46.1       bbmle_1.0.25.1         
##  [31] limma_3.66.0            rstudioapi_0.18.0       RSQLite_2.4.6          
##  [34] gridGraphics_0.5-1      abind_1.4-8             R.methodsS3_1.8.2      
##  [37] lifecycle_1.0.5         multcomp_1.4-29         yaml_2.3.12            
##  [40] BiocFileCache_3.0.0     qvalue_2.42.0           SparseArray_1.10.8     
##  [43] grid_4.5.2              blob_1.3.0              crayon_1.5.3           
##  [46] bdsmatrix_1.3-7         ggtangle_0.1.1          lattice_0.22-9         
##  [49] cowplot_1.2.0           annotate_1.88.0         KEGGREST_1.50.0        
##  [52] pillar_1.11.1           fgsea_1.36.2            boot_1.3-32            
##  [55] estimability_1.5.1      codetools_0.2-20        fastmatch_1.1-8        
##  [58] glue_1.8.0              ggiraph_0.9.6           ggfun_0.2.0            
##  [61] fontLiberation_0.1.0    data.table_1.18.2.1     vctrs_0.7.1            
##  [64] png_0.1-8               treeio_1.34.0           Rdpack_2.6.6           
##  [67] gtable_0.3.6            datawizard_1.3.0        emdbook_1.3.14         
##  [70] cachem_1.1.0            xfun_0.56               rbibutils_2.4.1        
##  [73] S4Arrays_1.10.1         rosm_0.3.1              coda_0.19-4.1          
##  [76] reformulas_0.4.4        survival_3.8-6          units_1.0-1            
##  [79] statmod_1.5.1           TH.data_1.1-5           nlme_3.1-168           
##  [82] pbkrtest_0.5.5          ggtree_4.0.4            bit64_4.6.0-1          
##  [85] fontquiver_0.2.1        progress_1.2.3          filelock_1.0.3         
##  [88] bslib_0.10.0            affyio_1.80.0           KernSmooth_2.23-26     
##  [91] DBI_1.3.0               tidyselect_1.2.1        curl_7.0.0             
##  [94] bit_4.6.0               compiler_4.5.2          httr2_1.2.2            
##  [97] xml2_1.5.2              fontBitstreamVera_0.1.1 DelayedArray_0.36.0    
## [100] bayestestR_0.17.0       scales_1.4.0            classInt_0.4-11        
## [103] hexbin_1.28.5           affy_1.88.0             rappdirs_0.3.4         
## [106] digest_0.6.39           minqa_1.2.8             rmarkdown_2.30         
## [109] XVector_0.50.0          htmltools_0.5.9         pkgconfig_2.0.3        
## [112] dbplyr_2.5.2            fastmap_1.2.0           rlang_1.1.7            
## [115] htmlwidgets_1.6.4       farver_2.1.2            jquerylib_0.1.4        
## [118] zoo_1.8-15              jsonlite_2.0.0          BiocParallel_1.44.0    
## [121] GOSemSim_2.36.0         R.oo_1.27.1             magrittr_2.0.4         
## [124] Formula_1.2-5           ggplotify_0.1.3         patchwork_1.3.2        
## [127] parameters_0.28.3       Rcpp_1.1.1              ape_5.8-1              
## [130] ggnewscale_0.5.2        gdtools_0.5.0           furrr_0.3.1            
## [133] stringi_1.8.7           MASS_7.3-65             plyr_1.8.9             
## [136] parallel_4.5.2          listenv_0.10.0          Biostrings_2.78.0      
## [139] splines_4.5.2           hms_1.1.4               locfit_1.5-9.12        
## [142] igraph_2.2.2            reshape2_1.4.5          XML_3.99-0.22          
## [145] evaluate_1.0.5          BiocManager_1.30.27     nloptr_2.2.1           
## [148] tzdb_0.5.0              tweenr_2.0.3            polyclip_1.10-7        
## [151] future_1.69.0           ggforce_0.5.0           xtable_1.8-8           
## [154] e1071_1.7-17            tidytree_0.4.7          tidydr_0.0.6           
## [157] class_7.3-23            viridisLite_0.4.3       ragg_1.5.0             
## [160] aplot_0.2.9             memoise_2.0.1           cluster_2.1.8.2        
## [163] timechange_0.4.0        globals_0.19.0
```
